# Stereoselective Alder-Ene Reactions of Bicyclo[1.1.0]butanes: Facile Synthesis of Cyclopropyl- and Aryl-Substituted Cyclobutenes

**DOI:** 10.1021/jacs.3c13080

**Published:** 2023-12-29

**Authors:** Ayan Dasgupta, Subrata Bhattacharjee, Zixuan Tong, Avishek Guin, Ryan E. McNamee, Kirsten E. Christensen, Akkattu T. Biju, Edward A. Anderson

**Affiliations:** † Chemistry Research Laboratory, Department of Chemistry, University of Oxford, 12 Mansfield Road, Oxford OX1 3TA, U.K.; ‡ Department of Organic Chemistry, Indian Institute of Science, Bangalore 560012, India

## Abstract

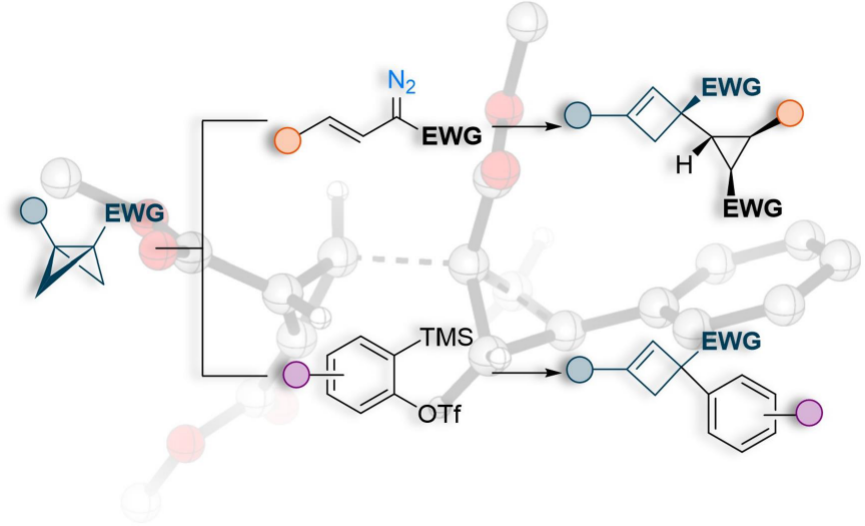

Bicyclo[1.1.0]butanes (BCBs), strained carbocycles comprising two fused cyclopropane rings, have become well-established building blocks in organic synthesis, medicinal chemistry, and chemical biology due to their diverse reactivity profile with radicals, nucleophiles, cations, and carbenes. The constraints of the bicyclic ring system confer high p-character on the interbridgehead C–C bond, leading to this broad reaction profile; however, the use of BCBs in pericyclic processes has to date been largely overlooked in favor of such stepwise, non-concerted additions. Here, we describe the use of BCBs as substrates for ene-like reactions with strained alkenes and alkynes, which give rise to cyclobutenes decorated with highly substituted cyclopropanes and arenes. The former products are obtained from highly stereoselective reactions with cyclopropenes, generated in situ from vinyl diazoacetates under blue light irradiation (440 nm). Cyclobutenes featuring a quaternary aryl-bearing carbon atom are prepared from equivalent reactions with arynes, which proceed in high yields under mild conditions. Mechanistic studies highlight the importance of electronic effects in this chemistry, while computational investigations support a concerted pathway and rationalize the excellent stereoselectivity of reactions with cyclopropenes.

## Introduction

**Figure 1:**
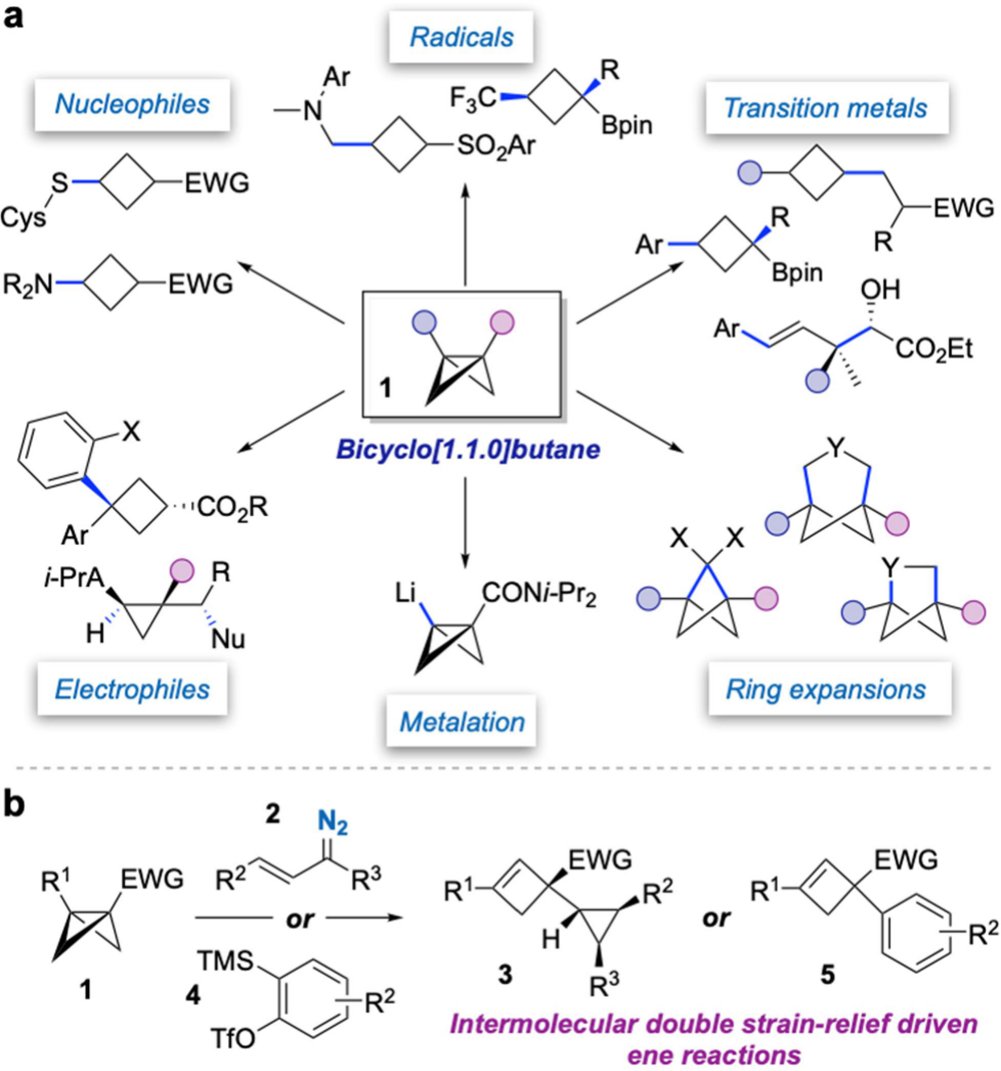
(a) Establishment of the reactivity of BCBs. (b) This work: stereoselective ene reactions of BCBs with cyclopropenes and arynes afford cyclopropyl- and aryl-substituted cyclobutenes.

Bicyclo[1.1.0]butanes (BCBs, **1**, Figure 1a) have emerged as versatile building blocks in organic synthesis due to the broad reactivity profile of their interbridgehead C1–C3 bond.^1^ With almost entirely p-character,^2^ ring-opening chemistry of this high-energy bond has been achieved using nucleophiles^3^ (including applications as bioconjugation agents),^3b,4^ radicals,^5^ electrophiles,^6^ and transition metals catalysts.^7^ In addition to such transformations, the ring expansion of BCBs to bicyclo[n.1.1]alkanes by formal one-,^8^ two-,^9^ or three-atom^10^ stepwise insertion processes has recently become a particularly valuable process due to the importance of these scaffolds in drug discovery.^11^ Finally, metalation of the acidic C–H bonds of BCBs provides further opportunities for scaffold diversification.^3f,12^

In contrast to this rich chemistry, the exploration of BCBs as components in pericyclic reactions is limited, which may be due to the ease with which the aforementioned chemistries can occur. Reports of such processes have to date been limited to [3 + 2] cycloadditions and aza-ene reactions of diimides^4b,13^ and other activated alkenes,^14^ intramolecular ene-type reactions of *N*-allylated BCBs to give spirocyclic cyclobutanes,^15^ and isolated examples of reactions between electron-rich BCBs and benzyne.^16^ In some cases, there is debate as to whether these reactions are truly concerted or proceed via stepwise diradical, or ionic, pathways. We questioned whether other strained π-bonds might also engage with BCBs, thus affording unique small-ring product architectures from these readily accessible building blocks. Here, we describe the strain-relief-driven Alder-ene reaction of polyfunctionalized BCBs with cyclopropenes, generated in situ from visible light-promoted decomposition of vinyl diazo compounds (**2**, Figure 1b),^17^ to give cyclopropyl-substituted cyclobutenes (**3**); and also, the development of highly efficient ene reactions of BCBs with a selection of arynes^18^ generated under mild reaction conditions from arylsilane triflates^19^**4** to give arylated cyclobutenes **5**. These reactions display a broad scope and high stereoselectivity; mechanistic studies support the involvement of an asynchronous concerted reaction pathway and highlight the importance of electronic effects in these transformations.

## Results and Discussion

Our studies began with photochemical carbene generation from the vinyl diazo compound **2a**, which is known to undergo spontaneous 2π-electrocyclization to the corresponding cyclopropene **6a** upon carbene formation (Table 1).^20,21^ UV–vis spectra first confirmed that **2a** is suitable for selective excitation in the presence of BCB **1a**, which does not absorb at 440 nm, and that cyclopropene **6a** was formed under these conditions.^22^ Pleasingly, irradiation of an equimolar quantity of **1a** and **2a** afforded the ene product **3a** in 67% yield as a single diastereomer (entry 1).^23^ Incomplete conversion was observed, which we ascribed to decomposition of **2a** over the 4 h reaction time. Increasing the equivalents of **2a**, and employing a slow addition protocol (2 h of addition time, 0.28 M solution of **2a**), improved the yield of **3a** to 88% (entry 2). Decreasing the reaction temperature resulted in poor conversion (along with significant recovery of **1a**), while no reaction was observed in the absence of light (entries 3, 4). Other solvents proved inferior to acetonitrile (entries 5–9).

**Table 1:**
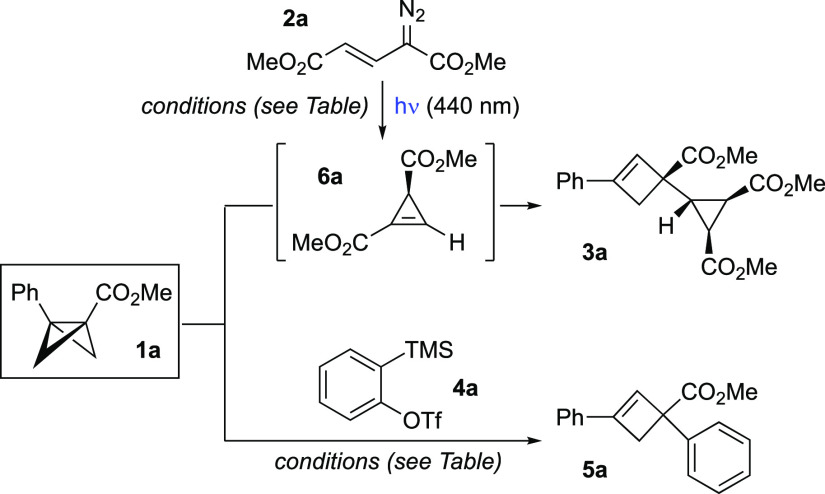
Optimization of Ene Reactions^a^

entry	substrate	conditions	time (h)	**2a**/**4a** (equiv)	yield **3a**/**5a** (%)
1	**2a**	MeCN	4	1	67
2	**2a**	MeCN	4	2	**88**
3	**2a**	MeCN^b^	8	2	21
4	**2a**	MeCN^c^	4	2	n.r.
5	**2a**	toluene	4	2	20
6	**2a**	Et_2_O	4	2	38
7	**2a**	CH_2_Cl_2_	4	2	38
8	**2a**	THF	3	2	31
9	**2a**	MeOH	3	2	44
10	**4a**	KF, THF^d^	12	1.2	85
11	**4a**	CsF, MeCN	12	1.2	82
12	**4a**	KF, THF^d^	12	1.5	96
13	**4a**	KF, THF^d^	4	1.5	**98**
14^e^	**4a**	KF, THF^d^	4	1.5	**98**

a Reactions were carried out using 0.1 mmol of **1a** (for reaction with **2a**) or 0.2 mmol scale of **1a** (for reaction with **4a**). Isolated yields are reported.

b Reaction conducted at 0 °C.

c Reaction carried out in the dark.

d Reaction conducted with 1 equiv 18-C-6 per equiv. KF.

e Performed on 1.0 mmol scale. n.r. = no reaction.

Encouraged by the success of the ene reaction using cyclopropene, we questioned whether other enophiles could engage with **1a** under similarly mild conditions. To this end, we were pleased to find that benzyne, produced in situ from 2-(trimethylsilyl)aryl triflate **4a** using KF and 18-crown-6, underwent a smooth ene reaction at room temperature, delivering phenyl-substituted cyclobutene **5a** in 85% yield (entry 10). Changing the fluoride source to CsF in CH_3_CN (to retard the rate of benzyne generation) did not improve the yield, but increasing the amount of benzyne precursor furnished **5a** in 96% yield (entries 11 and 12). Finally, reducing the reaction time to 4 h led to a slight improvement, with **5a** isolated in near quantitative yield on 0.2 and 1.0 mmol scale (98%, entries 13 and 14).

**Figure 2:**
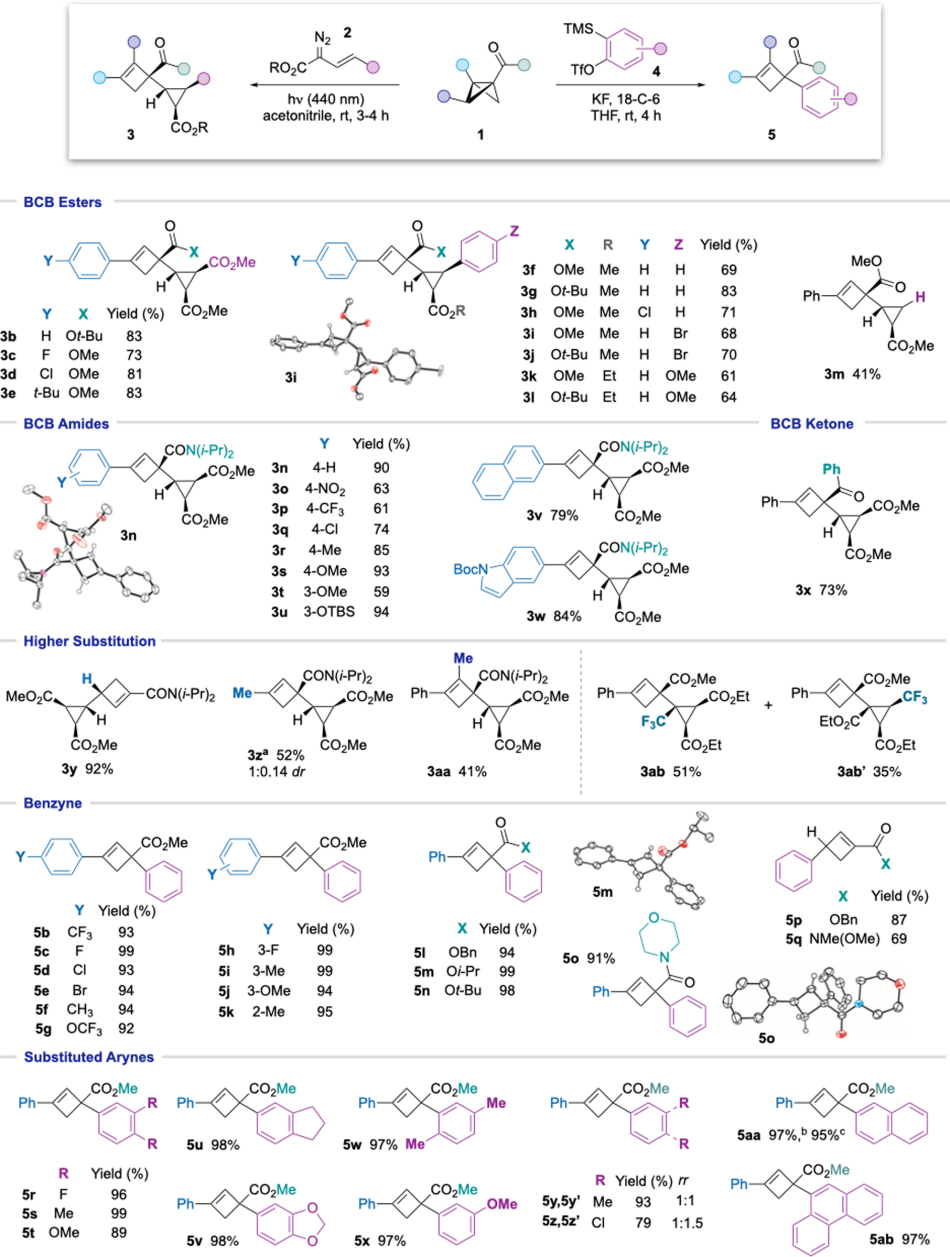
Substrate scope for the cyclopropene–BCB and aryne–BCB ene reactions. Thermal ellipsoids are shown at 50%. H atoms have been omitted for clarity. All reactions of vinyldiazo compounds were carried out on a 0.1 mmol scale and of aryne precursors on 0.2 mmol scale. Yields are isolated yields. ^a^**3z** was obtained in a 1:0.2 ratio with the regioisomeric ene product. ^b^Reaction of 1-naphthalyne. ^c^Reaction of 2-naphthalyne.

With optimized reaction conditions in hand for the reaction with both enophiles, we next explored the reaction scope (Figure 2). A variety of BCBs featuring ester, amide, and ketone groups were synthesized according to our previous studies^22^ and were combined with a selection of vinyl diazo esters (**2a**–**2e**). Pleasingly, good to excellent yields of cyclopropyl-substituted cyclobutenes were obtained (**3b**–**3v**, 41–93%), most as single diastereo- and regioisomers. We first found that reaction of C3-aryl substituted BCB esters **1b**–**1e** with diester or aryl/ester-substituted diazos **2a**–**2d** gave high yields of the product cyclobutenes **3b**–**3l** (61–83%). Use of a monosubstituted vinyl diazo ester **2e** gave a reduced yield of cyclobutene **3m**, the lower yield of which likely reflects the instability of the intermediate monosubstituted cyclopropene.^22^

The effect of α-substitution of the diazo compound was investigated using trifluoromethyl-substituted vinyldiazo ester **2h**.^24^ Interestingly, regioisomers **3ab** and **3ab′** were obtained in 51 and 35% yield, respectively, the structures of which were unequivocally assigned by X-ray crystallographic analysis. The formation of these isomers arises from ene reaction at either end of the intermediate cyclopropene, illustrating the importance of steric effects in controlling regioselectivity using other (α-unsubstituted) vinyldiazo compounds.

Variation of the BCB electron-withdrawing group and arene was well-tolerated: Aryl-substituted BCB amides afforded the corresponding cyclobutenes **3n**–**3u** in good to excellent yields (59–93%), with nitro, chloro, and aryl silyl ether groups all accommodated. Generally, electron-rich arenes were observed to give higher yields of ene products (e.g., **3u**, 94%). Bicyclic substituents were also successful, such as naphthyl (**3v**, 79%) and indole (**3w**, 84%) groups. A BCB ketone proved to be equally effective (**3x**, 73%).

Modification of the BCB bridgehead substituent led to interesting results: a monosubstituted BCB amide (R^1^, R^2^ = H) exclusively afforded the opposite regioisomer of ene product, with C–C bond formation now distal from the amide electron-withdrawing group (**3y**, 92%). In contrast to aryl-substituted BCBs, a bridgehead methyl-substituted BCB afforded a mixture of regioisomers in favor of C–C bond formation at the amide-bearing carbon (**3z**, 1:0.2 *rr*); the major product **3z** was isolated in 52% yield. Methyl substitution on the BCB bridge afforded a regioisomeric mixture of cyclobutenes, from which the major component **3aa**, featuring the more-substituted alkene, was isolated in 41% yield.

We next evaluated the scope of the reaction with arynes as enophiles. Under the optimized conditions, a wide variety of arylated BCB esters with substitution at the C4-position and C3-position of the arene gave excellent yields of the corresponding phenyl-substituted arylcyclobutenes **5b**–**j** (93–99%) on reaction with benzyne. Moreover, substitution at the C2-position of the BCB arene did not affect the reactivity (**5k**, 95%). Increasing the steric bulk of the BCB ester substituent and reaction with a BCB morpholine amide also proceeded with high efficiency (**5l**–**5o**, 91–98%). Interestingly, as observed with the cyclopropene ene reaction, an inversion of regioselectivity was observed in the reaction of benzyne with monosubstituted BCBs to give ester **5p** (87%) and Weinreb amide **5q** (69%).

The ene reaction also proceeded smoothly using an array of symmetrical and unsymmetrical-substituted arynes to give the corresponding arylated phenylcyclobutenes. The formation of **5r**–**5v** from symmetric arynes proceeded in excellent yields (89–99%), which were not compromised by *ortho* substituents (**5w**, 97%). Pleasingly, 3-methoxybenzyne delivered a single regioisomer **5x** in 97% yield;^12^ in contrast, 4-methyl- and 4-chlorobenzyne afforded mixtures of regioisomers with respect to the aryne (**5y** and **5z**), albeit in high yields. Interestingly, the symmetric and unsymmetric naphthalynes generated from the corresponding regioisomeric silyl triflate precursors produced the same naphthyl cyclobutene **5aa** in 95 and 97% yield, respectively. Finally, phenanthryne furnished the desired cyclobutene **5ab** in 97% yield. Arynes bearing electron-withdrawing groups such as NO_2_, and heterocyclic arynes such as pyridyne, did not afford the desired cyclobutene product under the optimized conditions.

**Figure 3:**
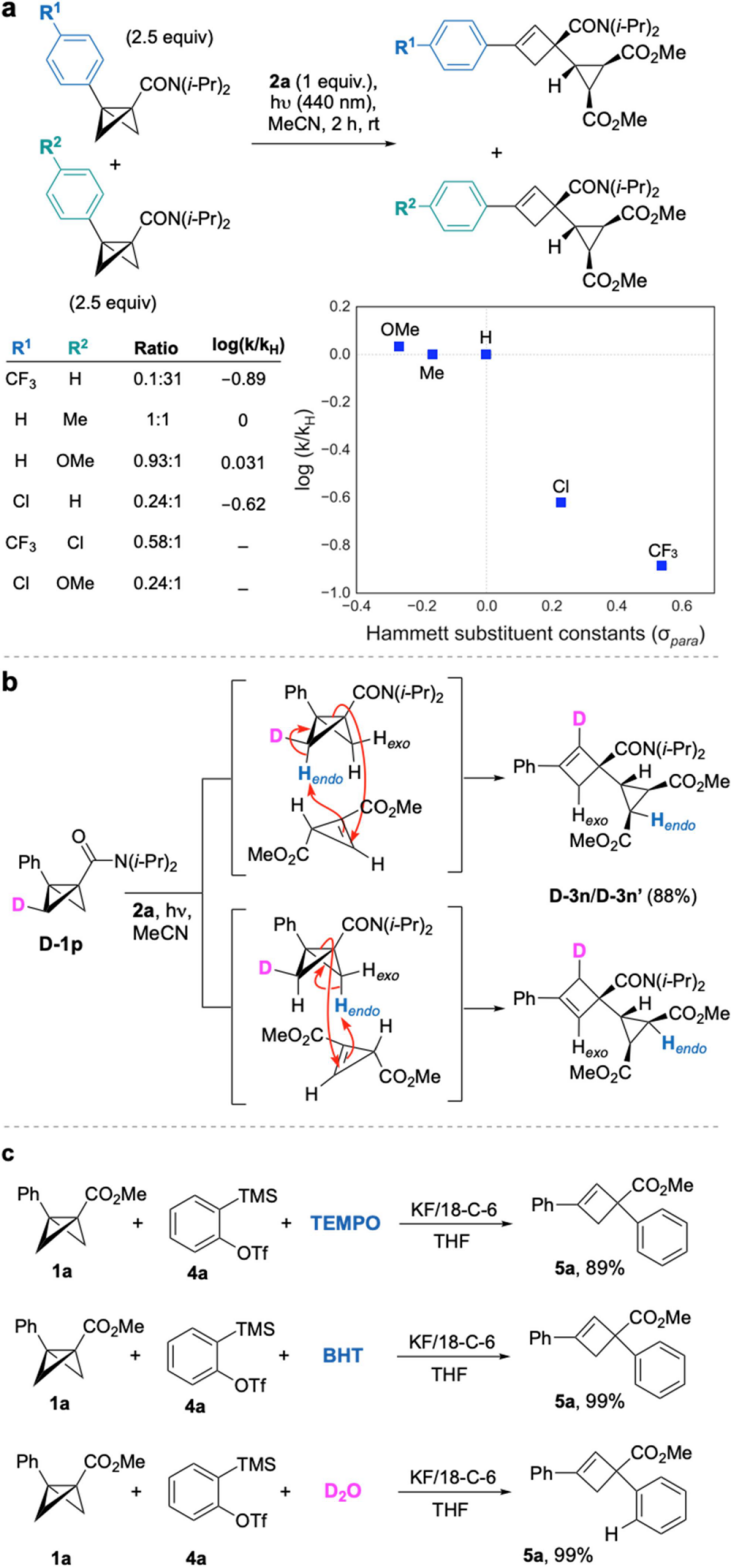
(a) Hammett study of the electronic effect of the bridgehead arene on the ene reaction; see the Supporting Information for details. (b) Deuterium labeling of the BCB bridge demonstrates exclusive *endo* C–H transfer to the cyclopropene. (c) Exploration of stepwise radical and ionic pathways for the aryne ene reaction.

To study the influence of electronic effects on the cyclopropene–BCB ene reaction, competition experiments were carried out between vinyl diazo **2a** and pairs of BCB amide substrates differing in the nature of the aryl group at the C3 position (using 2.5 equiv of each BCB relative to **2a**, Figure 3a). A plot of relative rate constants (log(*k*_X_/*k*_H_)) against the corresponding Hammett substituent constants revealed a surprising insensitivity to electron-donating groups (X = Me, OMe) but a dramatic retardation of reaction rate for electron-withdrawing substituents (Cl, CF_3_). We also subjected the bridge *exo-*deuterated BCB **D-1p** to reaction with **2a** (Figure 3b), which afforded a 1:1 mixture of ene products **D-3n** and **D-3n′** with the deuterium atom entirely retained on the cyclobutene ring. This confirms that the cyclopropene reacts exclusively with a C–H bond on the *endo* face of the BCB. We then explored the possibility that the aryne ene reaction could operate via stepwise radical or ionic pathways (Figure 3c). When the reaction was carried out in the presence of the radical scavengers TEMPO and BHT, **5a** was formed without significant detriment to the yield, suggesting the reaction is not radical-mediated.^25^ Moreover, treatment of BCB **1a** with aryne precursor **4a** under the optimized conditions in the presence of 2.0 equiv of D_2_O resulted in no incorporation of deuterium at the *ortho*-position (Figure 3c). This supports a concerted mechanism, rather than stepwise nucleophilic attack of the BCB on the aryne intermediate followed by protonation.

**Figure 4:**
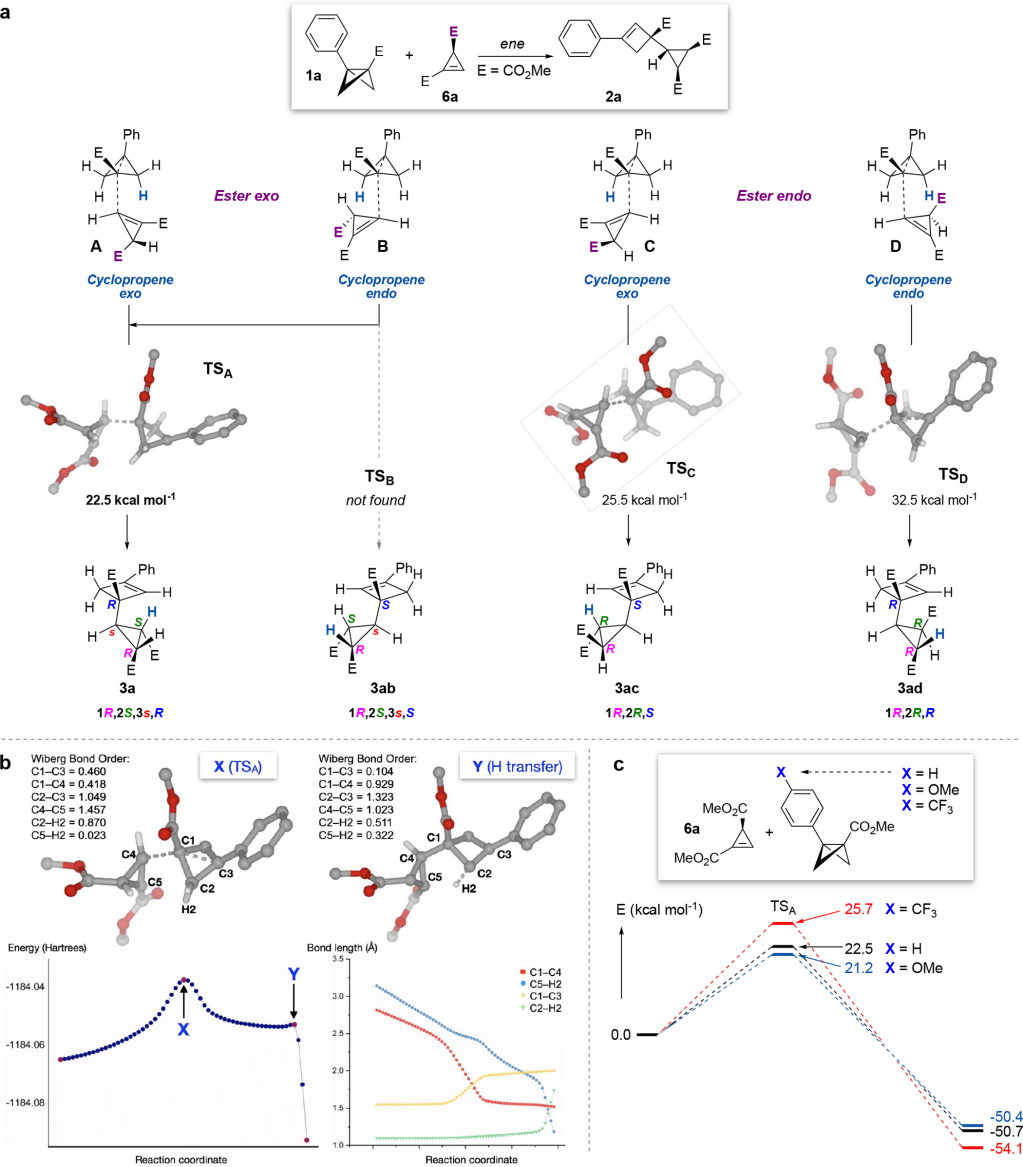
(a) Computation of conformations and ene transition states for the reaction of cyclopropene **3a** and cyclopropene **6a**. (b) Intrinsic reaction coordinate scan of the pathway via **TS**_**A**_ reveals a delayed C–H transfer in the ene reaction, and a ‘one step–two-stage’ concerted pathway. (c) Electronic effects on transition state energies (**TS**_**A**_). E = CO_2_Me.

To further explore the mechanism of the cyclopropene ene reaction^26^ and explain the stereoselectivity of the process, DFT calculations were carried out at the CPCM(acetonitrile)-DLPNO-CCSD(T)/def2-TZVPP//IEFPCM(acetonitrile)-B2PLYP(D3)/ def2-SVP level of theory at 298 K, using **1a** and **6a** as substrates (Figure 4a). Four possible arrangements of the cyclopropene and BCB were explored (**A**–**D**) in which the cyclopropene engages the BCB with the ester group on its sp^3^ carbon atom oriented *exo* (**A**, **B**) or *endo* (**C**, **D**). For each of these possibilities, the cyclopropene ring can then also orient *exo* (**A**, **C**) or *endo* (**B**, **D**) relative to the BCB. ‘Ester-*exo*’ conformations **A** and **B** would result in products **3a** (observed) and **3ab**, featuring a meso-cyclopropane group (i.e., ester groups *syn*) following transfer of the hydrogen atom from the BCB, and differ in the relative stereochemistry on the cyclobutene ring; ‘ester-*endo*’ conformations **C** and **D** lead to products **3ac** and **3ad** which also differ in the cyclobutene stereochemistry but now feature a chiral cyclopropane motif (ester groups *anti*).

We first identified the transition state **TS**_**A**_ which leads to the formation of the observed product **3a** with an energy barrier of 22.5 kcal mol^–1^. This TS shows that the ene reaction proceeds via a concerted but highly asynchronous mechanism in which C–H bond breaking/formation is delayed compared to C–C bond formation between the cyclopropene and the C3 bridgehead carbon atom (see discussion below). Two other transition states were identified: **TS**_**C**_ (25.5 kcal mol^–1^), and **TS**_**D**_ (32.5 kcal mol^–1^) that would lead to the (unobserved) (*R,R,S*)- and (*R,R,R*)-diastereomers **3ac** and **3ad**, respectively. A transition state corresponding to the fourth (*R,S,s,S*)-diastereomer **3ab** could not be located, with attempts to minimize conformation **B** instead converging on the presumably lower energy **TS**_**A**_ (which is effectively a rotamer of **TS**_**B**_ around the forming C–C bond).

The asynchronous nature of these transition states is consistent with previous calculations on cyclopropene ene reactions.^26^ To further explore this process, we performed an intrinsic reaction coordinate (IRC) scan (Figure 4b). This revealed an intriguing reaction profile that is characteristic of ‘one step–two stage’ asynchronous pericyclic processes:^24^**TS**_**A**_ (point **X**) is characterized by a C1–C4 bond length of 1.96 Å with a Mayer bond order of 0.418, which contrasts with a breaking C2–H2 bond length of 1.11 Å (bond order 0.870) and forming C5–H2 bond length of 1.52 Å (bond order 0.023). C–H bond formation is thus largely delayed until the molecule distorts sufficiently to bring H2 into proximity to C5 (C5–H2 bond order 0.322, point **Y**). At this point, the IRC scan reveals significant C–H transfer, developing cyclobutene double bond character, and rapid, exergonic H transfer.

Finally, we compared the relative transition state energies of BCB **1a** with those of equivalent BCB esters featuring electron-donating (OMe) and electron-withdrawing (CF_3_) substituents on the arene ring (Figure 4c). In comparison to **TS**_**A**_ (unsubstituted Ph ring, 22.5 kcal mol^–1^), these displayed barriers of 21.2 kcal mol^–1^ for **TS**_**A**_**(OMe)** and 25.7 kcal mol^–1^ for **TS**_**A**_**(CF**_**3**_**)**, which is in good agreement with the experimentally observed relative rates from the competition experiments (see Figure 3a).

**Figure 5:**
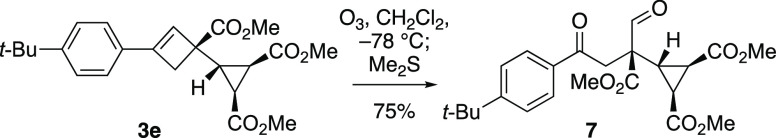
Product derivatization via the oxidative cleavage of cyclobutene **3e**.

The products of these ene reactions are rich in functionality and could be of use in other settings such as medicinal chemistry research, where highly substituted small ring systems are of importance. In terms of further chemistry, related cyclobutenes have recently been shown to be suitable substrates for (3 + 2) and (2 + 1) cycloaddition reactions to form rigid cyclobutane-fused ring systems.^27^ As an alternative to additional ring formation, we were able to demonstrate successful cyclobutene ring cleavage via ozonolysis of the double bond in **3e** (Figure 5), which afforded the complex cyclopropane-containing product **7** in 74% yield. Featuring three different carbonyl environments, this ketoaldehyde would be expected to undergo a variety of other chemistries toward stereochemically rich carbon backbones.

In conclusion, we have demonstrated highly regioselective and diastereoselective ene reactions of BCBs with cyclopropenes and arynes to afford cyclopropyl- and aryl-substituted cyclobutenes. The reactions proceed under mild conditions in good to near-quantitative yields. Experimental and DFT studies support an asynchronous concerted ‘one step–two-stage’ pathway, with exclusive reaction on the *endo*-face of the BCB. The cyclobutene products, which feature a quaternary carbon center to which the cyclopropane or arene is attached, are of potential use as small molecule building blocks in medicinal chemistry. Together, these methods further enhance the array of reactivities displayed by BCBs as valuable strain-relief building blocks in organic synthesis.

## Supporting Information Available

Experimental procedures, characterization data, coordinates, and crystallographic data of **3i**, **3n**, **3ab**, **5m**, **5o**, and **7** (PDF)
Coordinates (ZIP)

## References

[ref1] aGolfmann M.; Walker J. C. L. Bicyclobutanes as unusual building blocks for complexity generation in organic synthesis. Commun. Chem. 2023, 6, 9 10.1038/s42004-022-00811-3. 36697911 PMC9837078

[ref2] aSchulman J. M.; Newton M. D. Contributions to the nuclear spin-spin coupling constants of directly bonded carbons. J. Am. Chem. Soc. 1974, 96, 6295–6297. 10.1021/ja00827a009.

[ref3] aGuo L.; Noble A.; Aggarwal V. K. α-Selective Ring-Opening Reactions of Bicyclo[1.1.0]butyl Boronic Ester with Nucleophiles. Angew. Chem., Int. Ed. 2021, 60, 212–216. 10.1002/anie.202011739. 32956541

[ref4] aKaur A.; Lin W.; Dovhalyuk V.; Driutti L.; Di Martino M. L.; Vujasinovic M.; Löhr J. M.; Sellin M. E.; Globisch D. Chemoselective bicyclobutane-based mass spectrometric detection of biological thiols uncovers human and bacterial metabolites. Chem. Sci. 2023, 14, 5291–5301. 10.1039/D3SC00224A. 37234898 PMC10207876

[ref5] aPratt C. J.; Aycock R. A.; King M. D.; Jui N. T. Radical α-C–H Cyclobutylation of Aniline Derivatives. Synlett 2020, 31, 51–54. 10.1055/s-0039-1690197. 32103861 PMC7043791

[ref6] aGuin A.; Bhattacharjee S.; Harariya M. S.; Biju A. T. Lewis acid-catalyzed diastereoselective carbofunctionalization of bicyclobutanes employing naphthols. Chem. Sci. 2023, 14, 6585–6591. 10.1039/D3SC01373A. 37350821 PMC10284142

[ref7] aWölfl B.; Winter N.; Li J.; Noble A.; Aggarwal V. K. Strain-Release Driven Epoxidation and Aziridination of Bicyclo[1.1.0]butanes via Palladium Catalyzed σ-Bond Nucleopalladation. Angew. Chem., Int. Ed. 2023, 62, e202217064 10.1002/anie.202217064. PMC10107310 36507714

[ref8] aBychek R.; Mykhailiuk P. K. A Practical and Scalable Approach to Fluoro-Substituted Bicyclo[1.1.1]pentanes. Angew. Chem., Int. Ed. 2022, 61, e202205103 10.1002/anie.202205103. PMC9401599 35638404

[ref9] aAgasti S.; Beltran F.; Pye E.; Kaltsoyannis N.; Crisenza G. E. M.; Procter D. J. A catalytic alkene insertion approach to bicyclo[2.1.1]hexane bioisosteres. Nat. Chem. 2023, 15, 535–541. 10.1038/s41557-023-01135-y. 36781910

[ref10] aNguyen T. V. T.; Bossonnet A.; Waser J. Photocatalyzed [3σ + 2σ] and [3σ + 2π] cycloadditions for the synthesis of bicyclo[3.1.1]heptanes and cyclopentenes. ChemRxiv 2023, 10.26434/chemrxiv-2023-s8j30. 37934629

[ref11] aShire B. R.; Anderson E. A. Conquering the Synthesis and Functionalization of Bicyclo[1.1.1]pentanes. JACS Au 2023, 3, 1539–1553. 10.1021/jacsau.3c00014. 37388694 PMC10301682

[ref12] aMcNamee R. E.; Haugland M. M.; Nugent J.; Chan R.; Christensen K. E.; Anderson E. A. Synthesis of 1,3-disubstituted bicyclo[1.1.0]butanes via directed bridgehead functionalization. Chem. Sci. 2021, 12, 7480–7485. 10.1039/D1SC01836A. 34163838 PMC8171340

[ref13] aChang M. H.; Dougherty D. A. 2,3-Diazabicyclo[2.1.1]hex-2-ene. Synthesis and thermal decomposition. J. Org. Chem. 1981, 46, 4092–4093. 10.1021/jo00333a040.

[ref14] Cairncross A.; Blanchard E. P. Jr. Bicyclo[1.1.0]butane Chemistry. II. Cycloaddition Reactions of 3-Methylbicyclo[1.1.0]butanecarbonitriles. The Formation of Bicyclo[2.1.1]hexanes. J. Am. Chem. Soc. 1966, 88, 496–504. 10.1021/ja00955a021.

[ref15] aUeda M.; Walczak M. A. A.; Wipf P. Formal Alder-ene reaction of a bicyclo[1.1.0]butane in the synthesis of the tricyclic quaternary ammonium core of daphniglaucins. Tetrahedron Lett. 2008, 49, 5986–5989. 10.1016/j.tetlet.2008.07.179. 19129907 PMC2575373

[ref16] aPomerantz M.; Wilke R. N.; Gruber G. W.; Roy U. Electronic structure and reactivity of small ring compounds. V. Reaction of some bicyclobutanes with various dienophiles. J. Am. Chem. Soc. 1972, 94, 2752–2758. 10.1021/ja00763a037.

[ref17] aJurberg I. D.; Davies H. M. L. Blue light-promoted photolysis of aryldiazoacetates. Chem. Sci. 2018, 9, 5112–5118. 10.1039/C8SC01165F. 29938043 PMC5994880

[ref18] aJayanth T. T.; Jeganmohan M.; Cheng M.-J.; Chu S.-Y.; Cheng C.-H. Ene Reaction of Arynes with Alkynes. J. Am. Chem. Soc. 2006, 128, 2232–2233. 10.1021/ja058418q. 16478175

[ref19] aHimeshima Y.; Sonoda T.; Kobayashi H. Fluoride-induced 1,2-Elimination of *O*-trimethylsilylphenyl Triflate to Benzyne Under Mild Conditions. Chem. Lett. 1983, 12, 1211–1214. 10.1246/cl.1983.1211.

[ref20] Davies H. M. L.; Clark D. M.; Alligood D. B.; Eiband G. R. Mechanistic aspects of formal [3 + 4] cycloadditions between vinylcarbenoids and furans. Tetrahedron 1987, 43, 4265–4270. 10.1016/S0040-4020(01)90301-1.

[ref21] Hommelsheim R.; Guo Y.; Yang Z.; Empel C.; Koenigs R. M. Blue-Light-Induced Carbene-Transfer Reactions of Diazoalkanes. Angew. Chem., Int. Ed. 2019, 58, 1203–1207. 10.1002/anie.201811991. 30480350

[ref22] See the Supporting Information for details.

[ref23] The structure of the cyclopropane products was determined by NMR analysis of coupling constants and 2D NMR experiments, as well as by analogy with X-ray crystal structures for products **3i** and **3n**.

[ref24] Supurgibekov M. B.; Prakash G. K. S.; Nikolaev V. A. Two-Stage Synthesis of 3-(Perfluoroalkyl)-Substituted Vinyldiazocarbonyl Compounds and Their Nonfluorinated Counterparts: A Comparative Study. Synthesis 2013, 45, 1215–1226. 10.1055/s-0032-1318309.

[ref25] Scherübl M.; Daniliuc C. G.; Studer A. Arynes as Radical Acceptors: TEMPO-Mediated Cascades Comprising Addition, Cyclization, and Trapping. Angew. Chem., Int. Ed. 2021, 60, 711–715. 10.1002/anie.202012654. PMC7839731 33038065

[ref26] Deng Q.; Thomas B. E.; Houk K. N.; Dowd P. Transition Structures of the Ene Reactions of Cyclopropene. J. Am. Chem. Soc. 1997, 119, 6902–6908. 10.1021/ja963248q. 24236571

[ref27] Lin S.-L.; Chen Y.-H.; Liu H.-H.; Xiang S.-H.; Tan B. Enantioselective Synthesis of Chiral Cyclobutenes Enabled by Bro̷nsted Acid-Catalyzed Isomerization of BCBs. J. Am. Chem. Soc. 2023, 145, 21152–21158. 10.1021/jacs.3c06525. 37732875

