# Supplementary material for: Stereoselective Alder-Ene Reactions of Bicyclo[1.1.0]butanes: Facile Synthesis of Cyclopropyl- and Aryl-Substituted Cyclobutenes
Source: J Am Chem Soc. 2023 Dec 29;146(1):1196–203. doi: 10.1021/jacs.3c13080 (PMC10786042; doi:10.1021/jacs.3c13080)
Supplement: Supplementary file 1 — ja3c13080_si_001.pdf [file ja3c13080_si_001.pdf]

Supporting Information

Stereoselective Alder-ene Reactions of Bicyclo[1.1.0]butanes: Facile Synthesis of Cyclopropyl- and Aryl-substituted Cyclobutenes

Ayan Dasgupta,[a] Subrata Bhattacharjee,[b] Zixuan Tong,[a] Avishek Guin,[b] Ryan E. McNamee,[a] Kirsten E. Christensen,[a] Akkattu T. Biju[b]* and Edward A. Anderson[a]*

[a]Chemistry Research Laboratory, Department of Chemistry, University of Oxford, 12 Mansfield Road, Oxford, OX1 3TA, U.K

[b]Department of Organic Chemistry, Indian Institute of Science, Bangalore-560012, India

Table of Content

1	Experimental procedures	3
	1.1 General Statements	3
	1.2 List of BCBs and diazo esters employed for the Alder-ene reaction	4
	1.3 Synthesis and characterization of BCBs	5
	1.3.1 Synthesis of BCB-ester: General reaction procedure a	5
	1.3.2 Synthesis of BCB-amide: General reaction procedure b	6
	1.3.3 Synthesis of BCB-Ketone **1ae**	10
2	Optimization of Alder ene reaction (cyclopropene part)	11
	UV-Vis studies of diazo esters and BCB. 10^{-5} M acetonitrile solutions were used for the measurements	11
	2.1 Decomposition study of **2a**	11
	moles of **6a** (0.1, 0.2 and 0.3 mmol) calculated from the *in situ* ^{1}H NMR (AVX500, $CDCl_3$, 298 K) measure in 15, 30, 45, 95 and 185 min.	12
3	Synthesis and characterization of Alder-ene products (cyclopropene part)	13
	3.1 General reaction procedure c	13
	3.2 Synthesis and characterization of ene products	13
4	Competition Experiments	32
5	NMR Spectra (Cyclopropene part)	33
6	Optimization of the Alder-ene reaction (Aryne Part)	138
7	Synthesis and characterization of Alder-ene products (Aryne Part)	138
	7.1 General reaction procedure d	138
8	Mechanistic Experiments (Aryne Part)	152
9	NMR Spectra (Aryne Part)	155
10	Crystallographic Data	220
	10.1 Single crystal X-ray diffraction experimental	220
	10.2 Solid-state structure	221
	10.3 X-ray refinement data	224
11	Computational Data	230
	11.1 Computational details	230
	11.2 Evaluation of Level of Theory	231
	11.3 Intrinsic Reaction Coordinate (IRC) Calculation	235
	11.4 Cartesian coordinates and total energies for the calculated structures	237
12	References	266

1. Experimental procedures

1.1 General Statements:

NMR Spectroscopy: Proton (^{1}H), carbon (^{13}C), and fluorine (^{19}F) NMR spectra were recorded on Bruker AVIII HD 400, NEO 600, AVIII HD 500, AVII 500, AVII 600 or AVII 700 spectrometers (University of Oxford) and Bruker Ultrashield spectrometer (IISC India). ^{1}H, ^{13}C, and ^{19}F chemical shifts (δ) are quoted in parts per million (ppm). ^{1}H NMR spectra were recorded using an internal deuterium lock for the residual protons in chloroform-*d* (δ = 7.26) or acetonitrile-d_3 (δ = 1.94). ^{13}C NMR spectra were recorded using an internal deuterium lock in chloroform-*d* (δ = 77.16) or acetonitrile-d_3 (δ = 118.2). Assignments were determined either on the basis of unambiguous chemical shift or coupling patterns, COSY, HSQC, HMBC and/or NOESY experiments. Peak multiplicities are defined as s (singlet), d (doublet), t (triplet), q (quartet), p (quintet), sext (sextet), sept (septet), m (multiplet) and br (broad). Coupling constants (*J*) are reported to the nearest 0.1 Hz.

Mass Spectroscopy: High-resolution mass spectra (HRMS) were recorded by the Departmental Mass Spectrometry Service, University of Oxford on a Thermo Scientific Exactive Mass Spectrometer (Waters Equity autosampler and pump) for electrospray ionization (ESI) and an Agilent 7200 Accurate Mass QTOF GCMS (using a SIM Direct Insertion Probe) for electron ionization (EI) and chemical ionization (CI). HRMS (ESI) data were recorded on a Waters Xevo G2-XS Q-TOF instrument (IISC India). High-resolution values are calculated to 4 decimal places from the molecular formula, and all values are within a tolerance of 5 ppm.

Infrared Spectroscopy: Infrared spectra were obtained on a Bruker Tensor 27 FT-IR spectrometer, as a thin film by evaporation of a dichloromethane solution on a diamond ATR module. Wavelengths of maximum absorbance (υ_{max}) are quoted in cm^{-1}.

Chromatography: Column chromatography refers to normal phase column chromatography and was performed on silica gel obtained from Merck (Silica gel Si 60, 0.040–0.063 mm) under a positive pressure of nitrogen, using the stated solvent system. Analytical thin-layer chromatography was performed on pre-coated aluminium-backed plates (Merck Kieselgel 60 F_{254} plates) with visualization by ultraviolet light (254 nm) and/or by staining with potassium permanganate. Retention factors (R_f) are reported with the solvent system in parentheses.

Materials/procedures: All air- or moisture-sensitive reactions were carried out in anhydrous solvents in heat-gun-dried glassware under an inert atmosphere of argon or nitrogen. Light-sensitive reactions were carried out under aluminium foil protection. Heating was performed using an oil bath. Dry tetrahydrofuran, CH_2Cl_2, pyridine, triethylamine and diethyl ether were collected from an mBraun SPS-800 solvent purification system, having been passed through anhydrous alumina columns. All other commercially available reagents and solvents, where appropriate, were dried and purified before use, using standard procedures. Synthetic procedures and characterizations of all new compounds are listed below.

THF was freshly purified by distillation over Na-benzophenone and was transferred under nitrogen. 18-Crown-6 was recrystallized from dry CH_3CN, and KF was dried by heating at 110 °C for 12 h and left to cool under nitrogen, and stored in a nitrogen-filled glove box.

1.2 List of BCBs and diazo esters screened for the Alder-ene reaction.

Compounds **1a**, **1b**, **1c, 1d, 1f, 1g, 1h, 1i, 1j, 1l, 1k, 1l, 1m, 1n, 1o, 1p, 1q, 1s, 1aa, 1ab, 1ac, 1ad, 1af, and 1ag** was synthesized according the literature reports.[1,2,3,4] All the diazo esters **2a–2h**were synthesized following the literature procedure.[5,6] The 2-(trimethylsilyl) phenyl trifluoromethanesulfonate **4a** and the other symmetric and unsymmetrical aryne precursors **4b–4m** were prepared following the literature procedure.[7]

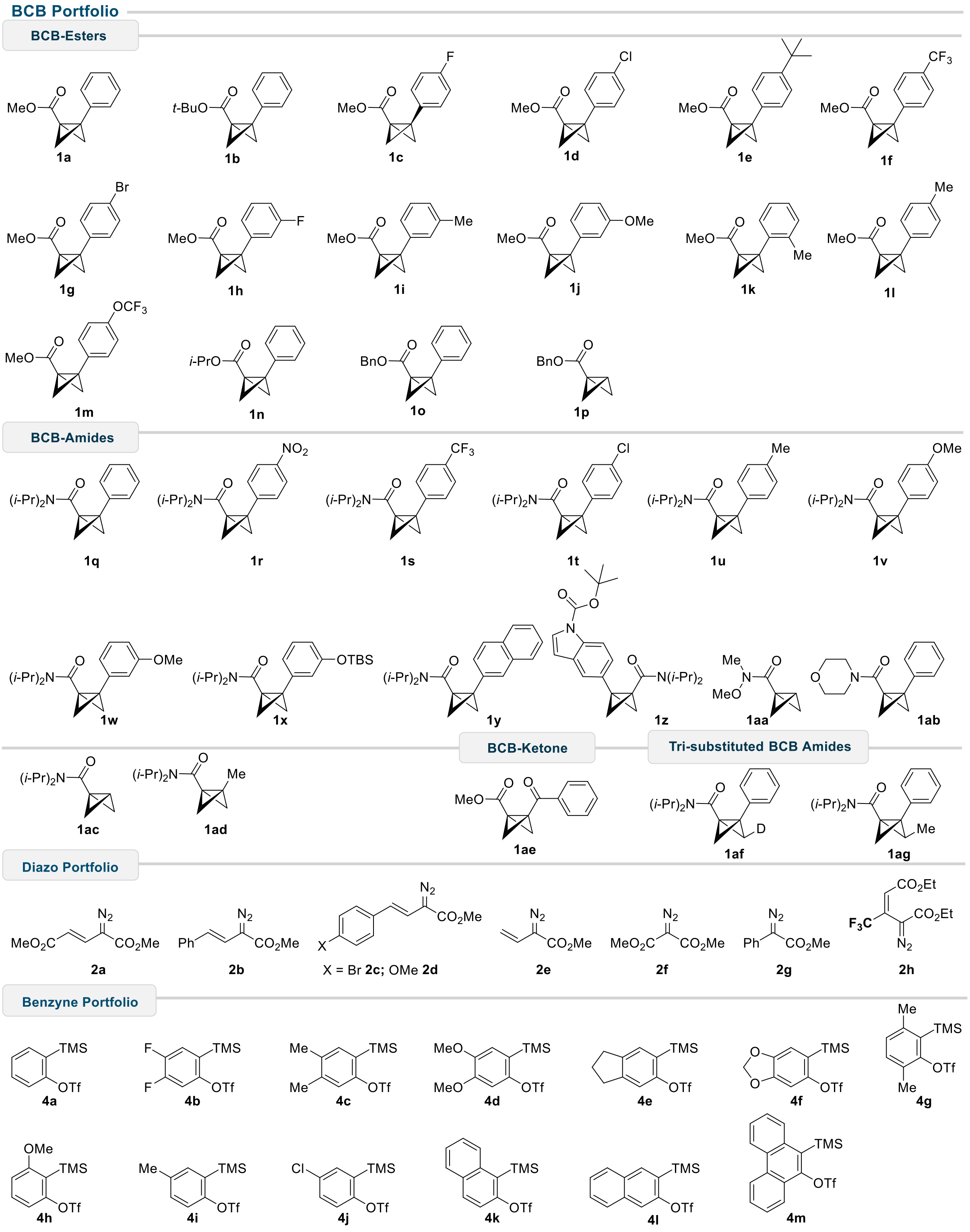

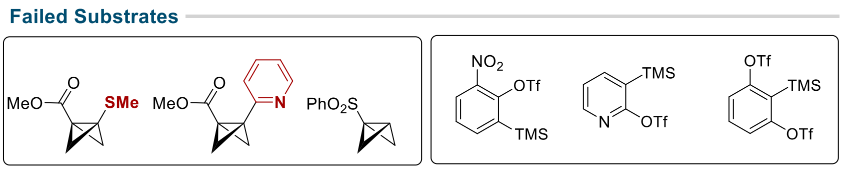

1.3 Synthesis and Characterization of BCBs:

1.3.1 Synthesis of BCB-ester: Reaction procedure a:[1,2]

The following reaction protocol was used to synthesize all the BCB esters using **I** and corresponding arylmagnesium bromides.

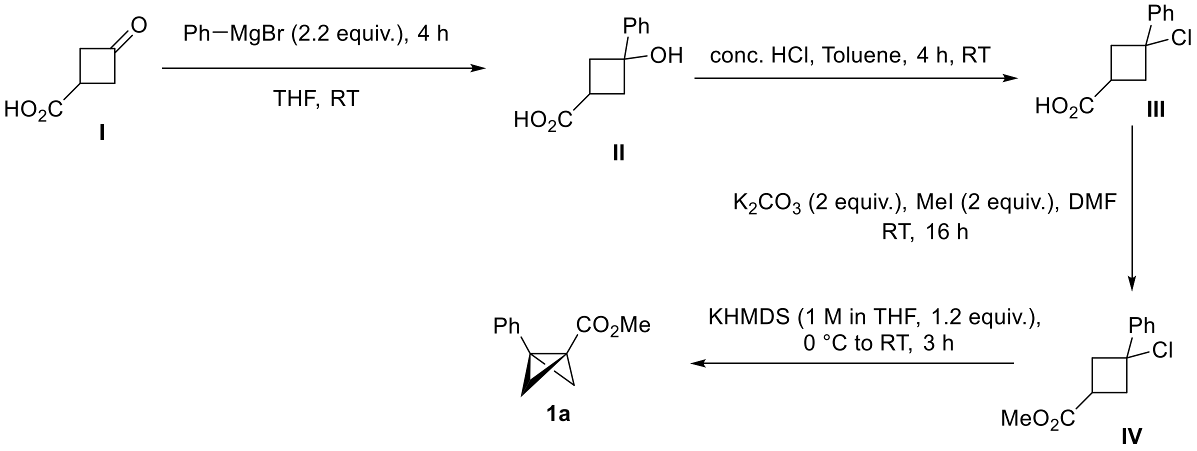

PhMgBr (3.0 M in THF, 106 mmol, 2.2 equiv.) was added dropwise to THF solution of **I** (5.50 g, 48 mmol, 1 equiv.) at 0 °C and stirred for 4 h under a nitrogen atmosphere. The reaction mixture was quenched with a saturated aqueous solution of NH_4Cl and extracted with ethyl acetate (3 × 30 mL). The combined organic layer was washed with brine (50 mL), dried over $MgSO_4$, and concentrated in a vacuum. A diastereomeric mixture (*syn*- and *anti*- isomers, 8 g, 42 mmol) was obtained and taken directly for the next step without further purification.

To a solution of **II** (8 g, 42 mmol, 1 equiv.) in toluene (100 mL), concentrated HCl (51 mL, 624 mmol, 15 equiv.) was added dropwise using a dropping funnel, and the reaction was stirred for 4 h at rt. The reaction mixture was diluted with 100 mL of brine solution, and the organic layer was extracted with EtOAc (3 × 100 mL). The combined organic phase was washed with water, $NaHCO_3$ (100 mL) and brine solution (100 mL), dried over $MgSO_4$, and concentrated in a vacuum. The residue (crude **III**) was taken into the next step without further purification.

To a solution of **III** (8.30 g, 39 mmol, 1 equiv.) in DMF (120 mL), K_2CO_3 (11 g, 79 mmol, 2 equiv.) was added portion wise, followed by the addition of MeI (4.90 mL, 79 mol, 2 equiv.). Then the reaction mixture was stirred for 16 h at rt, diluted with cold brine solution, and extracted with ethyl acetate (3 × 100 mL). The combined organic was dried over $MgSO_4$, and concentrated in a vacuum. The crude reaction mixture was purified using column chromatography using pentane/ethyl acetate (95:5; v/v).

To a solution of **IV** (5 g, 22 mmol, 1 equiv.) in toluene (150 mL) KHMDS (1.0 M in THF, 27 mL, 27 mmol, 1.2 equiv.) was added dropwise at 0 °C and stirred for the next 30 min and additional 2 h at room temperature. The reaction mixture was quenched with a saturated aqueous solution of NH_4Cl at 0 °C and the organic layer was extracted with ethyl acetate (3 × 50 mL). The combined organic phase was washed with brine (50 mL), dried over $MgSO_4$, and concentrated in a vacuum. The crude reaction mixture was purified by column chromatography on silica gel using pentane/ethyl acetate [5→10%] to afford **1a** (2.00 g, 10.46 mmol, 47%) as a colorless solid.

Characterization of **1e:**

Compound **1e** was synthesized according to the *reaction procedure a* using **I** (1 equiv.) and (4-(*tert*-butyl)phenyl)magnesium bromide (2.2 equiv.) as the starting material. The crude reaction mixture was purified by column chromatography on silica gel using pentane/ethyl acetate [5→10%] to afford **1e** (1.80 g, 7.48 mmol, 42%) as a colorless solid.

R_f = 0.4 (5% ethyl acetate/pentane).

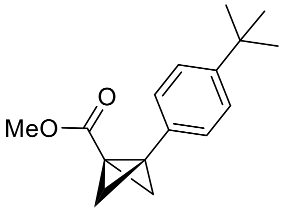

^{1}H NMR (500 MHz, $CDCl_3$, 298 K) δ 7.31 (d, *J* = 8.2 Hz, 2H, ArH), 7.22 (d, *J* = 8.5 Hz, 2H, ArH), 3.50 (s, 3H, CO_2Me), 2.90 (t, *J* = 1.2 Hz, 2H, H_{exo}), 1.58 (t, *J* = 1.2 Hz, 2H, H_{endo}), 1.30 (s, 9H, *t*-butyl); ^{13}C NMR (126 MHz, $CDCl_3$, 298 K) δ 170.5, 150.2, 130.5, 125.9, 125.6, 51.9, 35.9, 34.7, 33.4, 31.4, 23.0; IR υ_{max} (cm^{-1}): 2964, 1710, 1539, 1503, 1440, 1406, 1342, 1269, 1191, 1152, 1127, 1070, 1014; HRMS (ESI+) $[M+H]^+$ $[C_{16}H_{21}O_2]^+$: calculated 245.1542, found 245.1536.

1.3.2 Synthesis of BCB-amide: General reaction procedure b:[4]

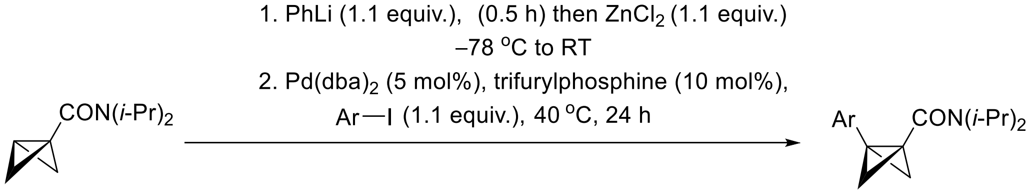

1ac

To a solution of **1ac** (91 mg, 0.50 mmol, 1 equiv.) in THF (1.50 mL) was added PhLi (0.29 mL, 1.9 M in *n*-Bu_2O, 0.55 mmol, 1.1 equiv.) dropwise at –78 °C. The mixture was stirred for 30 min before the addition of a THF solution of $ZnCl_2$ (75 mg, 0.55 mmol,1.1 equiv., 2 mL) at –78 °C. The reaction was warmed to rt and transferred *via* syringe to a vessel containing $Pd(dba)_2$ (14 mg, 0.02 mmol, 5 mol%) and tri-2-furylphosphine (12 mg, 0.05 mmol, 10 mol%). The mixture was stirred for 5 min before the addition of ArI (1.1 equiv.), then it was heated to 40 °C and stirred for 24 h. The reaction was cooled to rt, diluted with Et_2O (10 mL), and washed

with water (15 mL). The aqueous phase was extracted with Et_2O (3 × 25 mL), then the combined organic phases were dried over $MgSO_4$, filtered, and concentrated in a vacuum. The residue was purified *via* flash chromatography using diethyl ether/pentane to isolate the title compound. 1% NEt_3 was added to the eluent all the time to prevent the decomposition of the title compounds.

Characterization of **1r:**

Compound **1r** was synthesized according to the *General reaction procedure b* using **1ac** (91 mg, 0.50 mmol, 1 equiv.) and 1-iodo-4-nitrobenzene (137 mg, 0.55 mmol, 1.1 equiv.). The crude reaction mixture was purified *via* flash chromatography using diethyl ether/pentane (1% NEt_3, 30→50%) to yield **1r** (98 mg, 0.32 mmol, 65%) as a colorless solid.

R_f = 0.3 (30% diethyl ether/pentane).

^{1}H NMR (500 MHz, $CDCl_3$, 298 K) δ 8.14 (d, *J* = 8.9 Hz, 2H, ArH), 7.40 (d, *J* = 8.9 Hz, 2H, ArH), 4.67 (septet, *J* = 6.7 Hz, 1H, *iso*-propyl), 3.26 (septet, *J* = 6.8 Hz, 1H, *iso*-propyl), 2.87 (t, *J* = 0.9 Hz, 2H, H_{exo}), 1.66 (t, *J* = 0.9 Hz, 2H, H_{endo}), 1.22 (d, *J* = 6.7 Hz, 6H, *iso*-propyl), 1.13 (d, *J* = 6.8 Hz, 6H, *iso*-propyl); ^{13}C NMR (126 MHz, $CDCl_3$, 298 K) δ 166.2, 146.2, 143.7, 126.8, 123.5, 49.8, 46.3, 37.2, 29.1, 25.9, 21.6, 20.8; IR υ_{max} (cm^{-1}): 2967, 2936, 1619, 1442, 1369, 1348, 1289, 1263, 1238, 1210, 1155, 1122, 1038. HRMS (ESI+) $[M+H]^+$ $[C_{17}H_{23}N_2O_3]^+$: calculated 303.1709, found 303.1703.

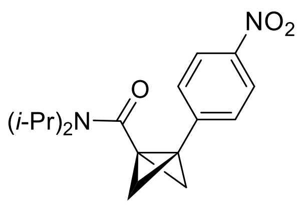

Characterization of **1t:**

Compound **1t** was synthesized according to the *General reaction procedure b* using **1ac** (91 mg, 0.50 mmol, 1 equiv.) and 1-chloro-4-iodobenzene (131 mg, 0.55 mmol, 1.1 equiv.). The crude reaction mixture was purified *via* flash chromatography using diethyl ether/pentane (1% NEt_3, 10→30%) to yield the **1t** (91 mg, 0.31 mmol, 62%) as a colorless solid.

R_f = 0.3 (10% diethyl ether/pentane).

^{1}H NMR (500 MHz, $CDCl_3$, 298 K) δ 7.33–7.12 (m, 4H, ArH), 4.69 (s, 1H, *iso*-propyl, br), 3.26 (s, 1H, *iso*-propyl, br), 2.75 (s, 2H, H_{exo}), 1.54 (s, *J* = 9.9 Hz, 2H, H_{endo}), 1.18 (d, *J* = 6.8 Hz, 12H, *iso*-propyl); ^{13}C NMR (126 MHz, $CDCl_3$, 298 K) δ 167.4, 133.4, 132.1, 128.4, 127.6, 49.5, 46.1, 36.5, 29.9, 29.5, 22.7, 21.6, 20.9; IR υ_{max} (cm^{-1}): 2966, 2936, 1621, 1439, 1369, 1347, 1288, 1263, 1210, 1155, 1122, 1097, 1038; HRMS (ESI+) $[M+H]^+$ $[C_{17}H_{23}ClNO]^+$: calculated 292.1468, found 292.1463.

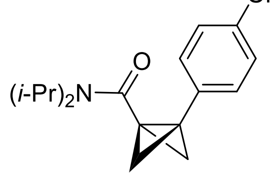

Characterization of **1u:**

Compound **1u** was synthesized according to the *General reaction procedure b* using **1ac** (91 mg, 0.50 mmol, 1 equiv.) and 1-iodo-4-methylbenzene (120 mg, 0.55 mmol, 1.1 equiv.). The crude reaction mixture was purified *via* flash chromatography using diethyl ether/pentane (1% NEt_3, 20→40%) to yield **1u** (90 mg, 0.33 mmol, 66%) as a colorless solid.

R_f = 0.4 (30% diethyl ether/pentane).

^{1}H NMR (500 MHz, $CDCl_3$, 298 K) δ 7.18 (d, *J* = 8.2 Hz, 2H, ArH), 7.10–7.06 (d, *J* = 8.2 Hz, 2H, ArH), 4.71 (s, 1H, *iso*-propyl, br), 3.25 (s, 1H, *iso*-propyl, br), 2.76 (s, 2H, H_{exo}), 2.29 (s, 3H, Me), 1.50 (s, 2H, H_{endo}), 1.18 1.07 (s, 12H, *iso*-propyl); ^{13}C NMR (126 MHz, $CDCl_3$, 298 K) δ 168.0, 135.9, 131.2, 129.0, 128.2, 127.6, 126.2, 49.5, 46.1, 36.5, 36.4, 30.4, 21.9, 21.6, 21.2, 21.0; IR υ_{max} (cm^{-1}): 2967, 2934, 1624, 1508, 1440, 1369, 1347, 1285, 1155, 1121, 1038; HRMS (ESI+) $[M+H]^+$ $[C_{18}H_{26}NO]^+$: calculated 272.2014, found 272.009.

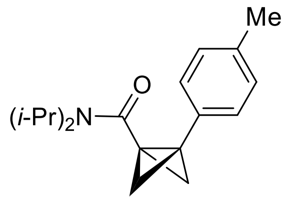

Characterization of **1v:**

Compound **1v** was synthesized according to the *General reaction procedure b* using **1ac** (91 mg, 0.50 mmol, 1 equiv.) and 1-iodo-4-methoxybenzene (129 mg, 0.55 mmol, 1.1 equiv.). The crude reaction mixture was purified *via* flash chromatography using diethyl ether and pentane (1% NEt_3, 30→50%) to yield **1v** (75 mg, 0.26 mmol, 52%) as a colorless solid.

R_f = 0.2 (20% diethyl ether/pentane).

^{1}H NMR (500 MHz, $CDCl_3$, 298 K) δ 7.22 (d, *J* = 8.8 Hz, 2H, ArH), 6.82 (d, *J* = 8.7 Hz, 2H, ArH), 4.70 (s, 1H, *iso*-propyl, br), 3.76 (s, 3H, OMe), 3.23 (s, 1H, *iso*-propyl, br), 2.74 (s, 2H, H_{exo}), 1.49 (s, 2H, H_{endo}), 1.17 (d, 12H, *iso*-propyl, br); ^{13}C NMR (126 MHz, $CDCl_3$) δ 168.1, 158.4, 127.4, 126.3, 113.9, 55.4, 49.5, 46.0, 36.4, 30.5, 21.6, 21.4, 21.0; IR υ_{max} (cm^{-1}): 2967, 1624, 1508, 1441, 1369, 1348, 1284, 1212, 1155, 1121, 1038; HRMS (ESI+) $[M+H]^+$ $[C_{18}H_{26}NO_2]^+$: calculated 288.1964, found 288.1964.

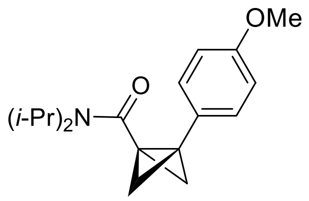

Characterization of **1w:**

Compound **1w** was synthesized according to the *General reaction procedure b* using **1ac** (91 mg, 0.50 mmol, 1 equiv.) and 1-iodo-3-methoxybenzene (129 mg, 0.55 mmol, 1.1 equiv.). The crude reaction mixture was purified *via* flash chromatography using diethyl ether and pentane (1% NEt_3, 30→40%) to yield **1w** (79 mg, 0.28 mmol, 55%) as a colorless solid.

R_f = 0.3 (30% diethyl ether/pentane)

^{1}H NMR (500 MHz, $CDCl_3$, 298 K) δ 7.19 (t, *J* = 8.0 Hz, 1H, ArH), 6.89 (dt, *J* = 7.7, 1.3 Hz, 1H,

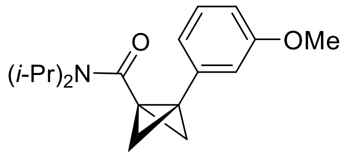

ArH), 6.90–6.82 (m, 1H, ArH), 6.72 (dd, *J* = 8.2, 2.6, 1H, ArH), 4.72 (s, 3H, OMe), 3.78 (s, 1H, *iso*-propyl, br), 3.25 (s, 1H, *iso*-propyl, br), 2.76 (s, 2H, H_{exo}), 1.50 (s, 2H, H_{endo}), 1.19 (br, 12H, *iso*-propyl)* (*broadening is due to slow C–C bond rotation, fast H exchange); ^{13}C NMR (126 MHz, $CDCl_3$, 298 K) δ 167.6, 159.6, 136.2, 129.2, 118.9, 112.3, 111.9, 55.3, 49.5, 46.1, 36.5, 30.3, 22.5, 21.6, 21.0; IR υ_{max} (cm^{-1}): 2982, 2941, 1627, 1514, 1424, 1349, 1264, 1211, 1175, 1141, 1038; HRMS (ESI+) $[M+H]^+$ $[C_{18}H_{26}NO_2]^+$: calculated 288.1964, found 288.1958.

Synthesis of **1x:***

Compound **1x** was synthesized according to the *General reaction procedure b* using **1ac** (91

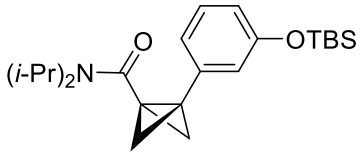

mg, 0.50 mmol, 1 equiv.) and *tert*-butyl(3-iodophenoxy)dimethylsilane (201 mg, 0.55 mmol, 1.1 equiv.). The crude reaction mixture was purified *via* flash chromatography using diethyl ether and pentane (1% NEt_3, 10→20%) to yield **1x** as a colorless oil.

R_f = 0.7 (10% diethyl ether/pentane).

*Due to the instability of the molecule, BCB-**1x** was synthesised using a standard literature report[4] and directly employed in the ene reaction.

Characterization of **1y:**

Compound **1y** was synthesized according to the *General reaction procedure b* using **1ac** (91 mg, 0.50 mmol, 1 equiv.) and 2-iodonaphthalene (140 mg, 0.55 mmol, 1.1 equiv.). The crude reaction mixture was purified *via* flash chromatography using diethyl ether/pentane (1% NEt_3, 10→30%) to yield **1y** (110 mg, 0.34 mmol, 72%) as a colorless solid.

R_f = 0.2 (10% diethyl ether/pentane).

^{1}H NMR (500 MHz, $CDCl_3$, 298 K) δ 7.78–7.74 (m, 4H, ArH), 7.43–7.37 (m, 3H, ArH), 4.76 (s,

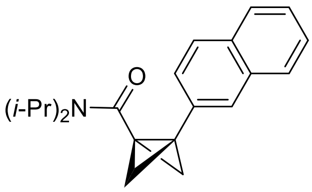

1H, *iso*-propyl, br), 3.24 (s, 1H, *iso*-propyl, br), 2.91 (s, 2H, H_{exo}), 1.60 (s, 2H, H_{endo}), 1.12 (d, 12H, *iso*-propyl); ^{13}C NMR (126 MHz, $CDCl_3$, 298 K) δ 167.6, 133.5, 132.37, 132.34, 127.8, 127.6, 126.0, 125.6, 125.3, 124.2, 49.5, 46.1, 36.7, 30.6, 22.7, 21.6, 20.9; IR υ_{max} (cm^{-1}): 2968, 2934, 1612, 1505, 1443, 1368, 1340, 1263, 1206, 1159, 1122, 1088, 1035; HRMS (ESI+) $[M+H]^+$ $[C_{21}H_{26}NO]^+$: calculated 308.2014, found 308.2009.

Characterization of **1z:**

Compound **1z** was synthesized according to the *General reaction procedure b* using **1ac** (36 mg, 0.20 mmol, 1 equiv.), PhLi (0.12 mL), $ZnCl_2$ (30 mg), $Pd(dba)_2$ (6 mg), tri(2-furyl)phosphine (5 mg) and *tert*-butyl 5-iodo-1H-indole-1-carboxylate (76 mg, 0.22 mmol, 1.1 equiv.). The crude reaction mixture was purified via flash chromatography using diethyl ether/pentane (1% NEt_3, 20→30%) to yield **1z** (58 mg, 0.15 mmol, 73%) as a colorless solid.

R_f = 0.3 (30% diethyl ether/pentane).

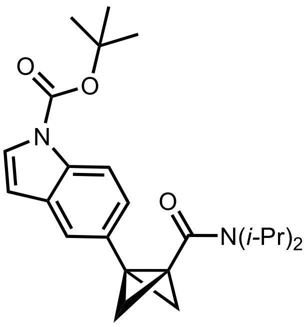

^{1}H NMR (500 MHz, C_6D_6) δ 8.44 (s, 1H, ArH), 7.70 (s, 1H, ArH), 7.49 (s, 1H, ArH), 7.45 (d, *J* = 9.4 Hz, 1H, Indole), 6.33 (d, *J* = 3.0 Hz, 1H, Indole), 4.52 (br, s, 1H, isopropyl), 2.89 (s, 2H, H_{exo}), 2.84 (s, 1H, isopropyl, br), 1.36 (s, 2H, H_{endo}), 1.33 (s, 9H, BOC), 1.19 (s, 6H, isopropyl, br), 0.84 (s, 6H, isopropyl, br); ^{13}C NMR (126 MHz, C_6D_6) δ 166.9, 149.8, 131.2, 129.4, 126.3, 123.6, 119.6, 115.3, 107.6, 82.9, 48.9, 46.1, 36.6, 34.4, 30.4, 27.9, 22.7, 22.0, 21.2, 14.3; IR υ_{max} (cm^{-1}): 2972, 1732, 1618, 1520, 1468, 1332, 1265, 1162, 1023; HRMS (ESI+) $[M+H]^+$ calculated for $[C_{24}H_{32}N_2O_3]^+$: 395.2340, found: 395.2321.

1.3.3 Synthesis and characterization of 1ae:

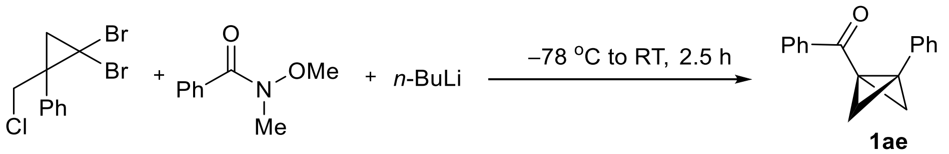

1ae

To a stirred THF (3 mL) solution of (2,2-dibromo-1-(chloromethyl)cyclopropyl)benzene (350 mg, 1.08 mmol, 1 equiv.) *n*-BuLi (2.5 M solution in Hexane, 0.9 mL, 2.38 mmol, 2.2 equiv.) was slowly added at –78 °C. After stirring for 30 min, THF solution (2 mL) of *N*-methoxy-*N*-methylbenzamide (214 mg, 1.30 mmol, 1.2 equiv.) was added dropwise to the reaction mixture. The reaction mixture was stirred at the same temperature for the next 1 h and slowly bring it to room temperature and allowed to stir for another 1 h at room temperature. And then quenched with sat NH_4Cl solution (3 mL). The aqueous phase was extracted with Et_2O (3 × 10 mL), then the combined organic phases were dried over $MgSO_4$, and concentrated in a vacuum. The crude reaction mixture was purified *via* flash chromatography using diethyl ether and pentane (1% NEt_3, 30→50%) to yield **1ae** (177 mg, 0.75 mmol, 70%) as a yellow solid.

R_f = 0.4 (30% diethyl ether/pentane).

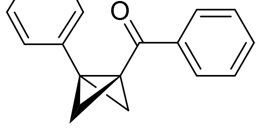

^{1}H NMR (500 MHz, $CDCl_3$, 298 K) δ 7.54 (dd, *J* = 8.3, 1.3 Hz, 2H, ArH), 7.48–7.43 (m, 1H, ArH), 7.37–7.31 (m, 2H, ArH), 7.25–7.19 (m, 3H, ArH), 7.17–7.10 (m, 2H, ArH), 3.17 (s, 2H, H_{exo}), 1.91 (s, 2H, H_{endo}); ^{13}C NMR (126 MHz, $CDCl_3$, 298 K) δ 196.7, 138.5, 133.0, 132.0, 128.5, 128.5, 128.2, 127.4,

126.2, 38.8, 37.8, 31.4; IR υ_{max} (cm^{-1}): 3060, 2936, 1732, 1599, 1577, 1516, 1482, 1447, 1399, 1345, 1265, 1211, 1175, 1131, 1062, 1036; HRMS (GC-EI+) $[M]^+$ $[C_{17}H_{14}O]^+$: calculated 234.10392, found 234.10447.

2 Optimization of Alder ene reaction (cyclopropene part)

UV-Vis studies of diazo esters and BCB (10^{-5} M acetonitrile solution).

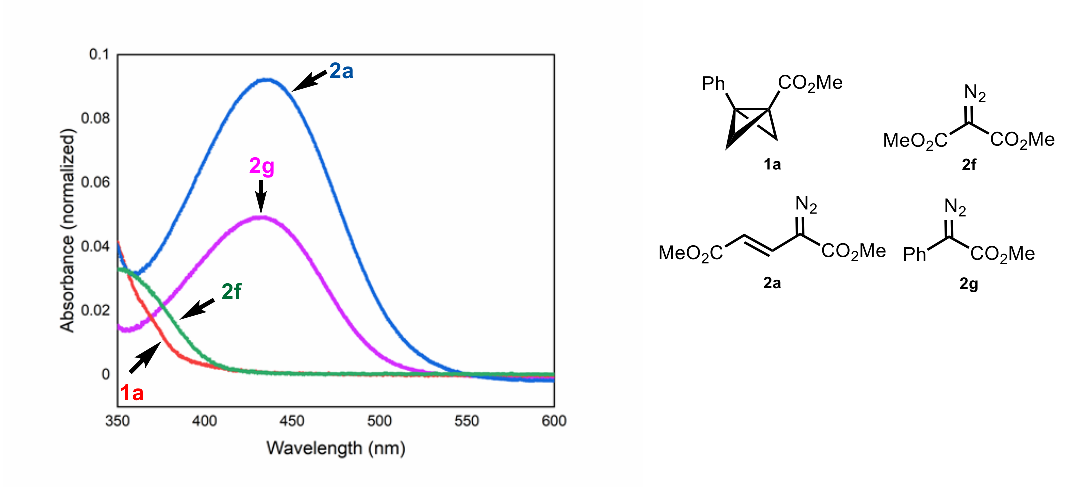

2.1 Decomposition study of 2a

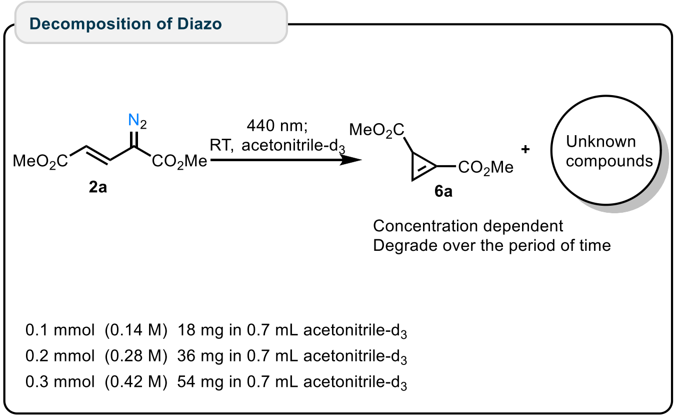

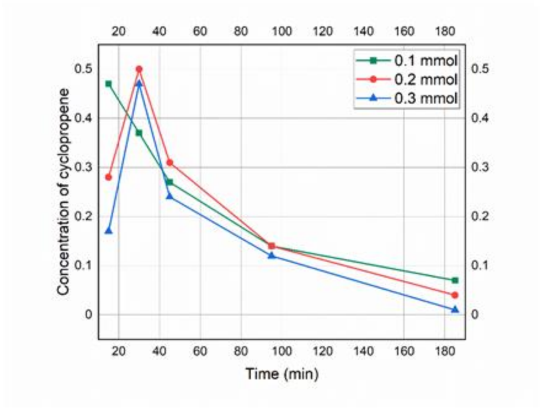

A time-dependent decomposition of **2a** was investigated using 3 different concentrations (0.14 M, 0.28 M and 0.42 M). All the reactions were carried out in the NMR tube. Acetonitrile-d3 solution of **2a** was irradiated using 440 nm light. The decomposition of **2a** was monitored using ^{1}H NMR at certain time intervals (15, 30, 45, 95 and 185 min). From the ^{1}H NMR of the crude reaction mixture complete conversion was observed to **6a**, in all cases. The ^{1}H NMR profile of the reaction showed increased levels of unidentifiable side reactivity with respect to concentration. Reaction at 0.2 M produced the lowest levels of decomposition relative to higher concentrations – in addition to maintaining good levels of Alder-ene reactivity.

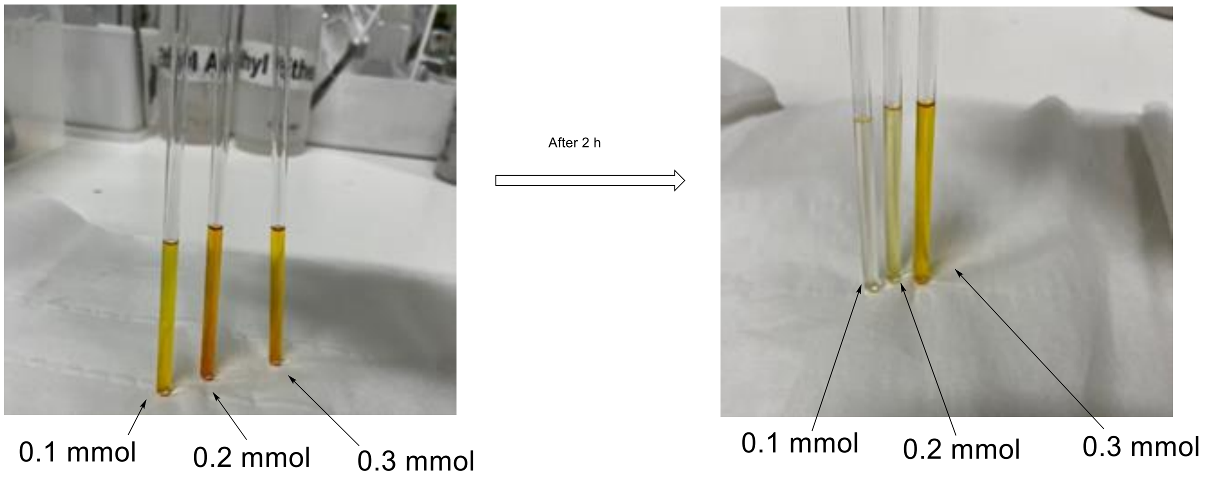

Moles of **6a** (0.1, 0.2 and 0.3 mmol) calculated from the *in situ* ^{1}H NMR (500 MHz, $CDCl_3$, 298 K) measured in 15, 30, 45, 95 and 185 min.

Entry	Time (min)	Moles of 6a		
		0.1 mmol (0.14 M)	0.2 mmol (0.28 M)	0.3 mmol (0.54 M)
1	15	0.47	0.28	0.17
2	30	0.37	0.50	0.47
3	45	0.27	0.31	0.24
4	95	0.14	0.14	0.12
5	185	0.07	0.04	0.01

3 Synthesis and characterization of Alder-ene products (cyclopropene part):

3.1 General reaction procedure c:

To a stirred acetonitrile solution of BCBs (0.1 mmol, 1 equiv., 0.5 mL), 1.5 mL acetonitrile solution of diazo esters (0.2 mmol, 2 equiv.) was added slowly through a syringe pump (1.5 / 2 h). The reaction was stirred at ambient temperature, under 440 nm light. All volatiles were removed *in a vacuum,* and the crude reaction mixture was purified *via* column chromatography using silica gel (Merck, 60 Å, 40–63 μm particle size) and pentane/diethyl ether unless otherwise stated.

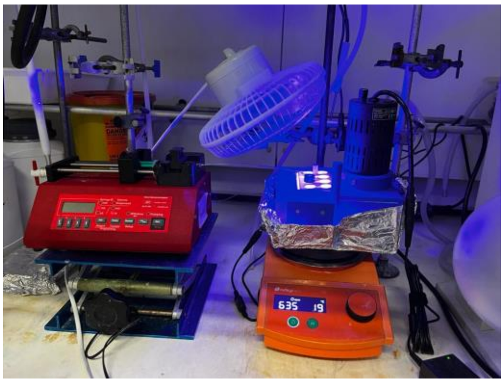

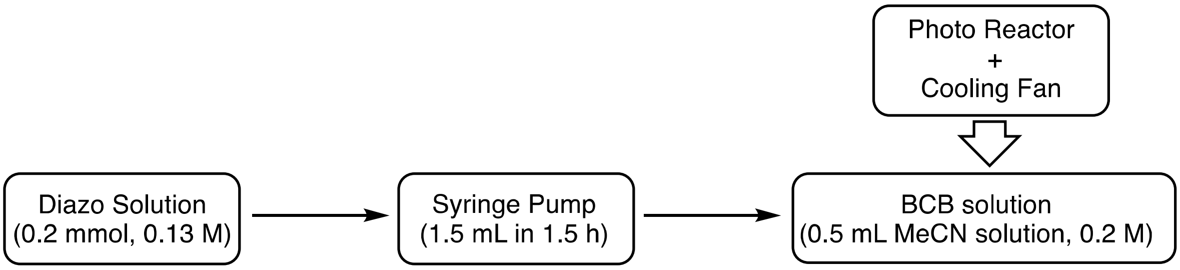

Photo Box: 8 X 4 mL vials photochemistry holder and HCK1006-01-016

Light-source: Kessil PR160L-440NM LED

Cooling Fan: 5 Star Fcl Clip-On Fan 6Inch White

3.2 Synthesis and characterization of ene products:

Synthesis and characterization of **3a:**

Synthesized in accordance with *General reaction procedure c* using BCB-**1a** (19 mg, 0.10 mmol, 1 equiv.) and diazo ester **2a** (37 mg, 0.20 mmol, 2 equiv.). All volatiles were removed in a vacuum, and the crude compound was purified *via* column chromatography using silica gel and pentane/diethyl ether (1:1 v/v; R_f = 0.5). The desired product **3a** was obtained as a colorless liquid. Yield: 30 mg, 0.09 mmol, 88%.

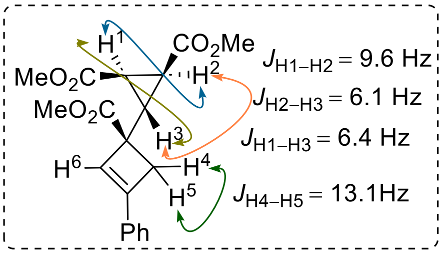

^{1}H NMR (500 MHz, $CDCl_3$, 298 K) δ: 7.37–7.30 (m, 5H, ArH), 6.05 (s, 1H, H6), 3.73 (s, 3H, CO_2Me), 3.71 (s, 3H, CO_2Me), 3.68 (s, 3H, CO_2Me), 3.24 (d, *J* = 13.1 Hz, 1H, H4), 2.73 (d, *J* = 13.1 Hz, 1H, H5), 2.71 (t, *J* = 6.2 Hz, 1H, H3), 2.14 (dd, *J* = 9.5, 6.4 Hz, 1H, H2), 2.09 (dd, *J* = 9.6, 6.1 Hz, 1H, H1); ^{13}C NMR (126 MHz, $CDCl_3$, 298 K) δ: 174.0, 170.0, 169.9, 150.1, 133.1, 129.3, 128.8, 125.3, 123.2, 52.7, 52.6, 49.5, 38.4, 29.7, 26.1, 24.1; IR υ_{max} (cm^{-1}): 2955, 1736,

1598, 1450, 1439, 1362, 1250, 1215, 1170, 1116, 1074; HRMS (ESI+) $[M+H]^+$ calculated for $[C_{19}H_{21}O_6]^+$: 345.1333, found: 342.1327.

Synthesis and characterization of **3b:**

Synthesized in accordance with *General reaction procedure c* using BCB-**1b** (23 mg, 0.10 mmol, 1 equiv.) and diazo ester **2a** (37 mg, 0.20 mmol, 2 equiv.). All volatiles were removed in a vacuum and the crude compound was purified *via* column chromatography using silica gel and pentane/diethyl ether (3:2 v/v; R_f = 0.7). The desired product **3b** was obtained as a colorless solid. Yield: 32 mg, 0.08 mmol, 83%.

^{1}H NMR (500 MHz, $CDCl_3$, 298 K) δ: 7.36–7.29 (m, 5H, ArH), 6.03 (s, 1H, H6), 3.70 (s, 3H, CO_2Me), 3.68 (s, 3H, CO_2Me), 3.21 (d, J = 13.1 Hz, 1H, H4), 2.65 (d, J = 12.5 Hz, 1H, H5), 2.63 (d, J = 5.7 Hz, 1H, H3), 2.13 (dd, J = 9.5, 6.4 Hz, 1H, H2), 2.09 (dd, J = 9.5, 6.2 Hz, 1H, H1), 1.46 (s, 9H, *t*-butyl); ^{13}C NMR (126 MHz, $CDCl_3$, 298 K) δ: 172.5, 170.0, 169.9, 149.8, 133.2, 129.0, 128.6, 125.2, 123.6, 81.5, 52.4, 52.3, 50.4, 37.7, 30.1, 28.1, 26.0, 24.1; IR $υ_{max}$ (cm^{-1}): 2979, 2953, 1728,1597, 1490, 1450, 1392, 1368, 1254, 1392, 1368, 1254, 1210, 1167, 1115, 1015; HRMS (ESI+) $[M+Na]^+$ calculated for $[C_{22}H_{26}O_6Na]^+$: 409.1622, found: 409.1608.

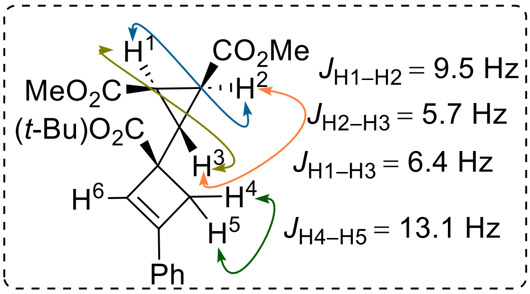

Synthesis and characterization of **3c:**

Synthesized in accordance with *General reaction procedure c* using BCB-**1c** (21 mg, 0.10 mmol, 1 equiv.) and diazo ester **2a** (37 mg, 0.20 mmol, 2 equiv.). All volatiles were removed in a vacuum and the crude compound was purified *via* column chromatography using silica gel and pentane/diethyl ether (1:1 v/v; R_f = 0.4). The desired product **3c** was obtained as a white solid. Yield: 26 mg, 0.07 mmol, 73%.

^{1}H NMR (500 MHz, $CDCl_3$, 298 K) δ: 7.32–7.30 (m, 2H, ArH), 7.05–7.02 (m, 2H, ArH), 5.98 (s, 1H, H6), 3.73 (s, 3H, CO_2Me), 3.710 (s, 3H, CO_2Me), 3.68 (s, 3H, CO_2Me), 3.22 (d, J = 13.1 Hz, 1H, H4), 2.71 (d, J = 13.4, Hz, 1H, H5), 2.72 (t, J = 6.4 Hz, 1H, H3), 2.12 (dd, J = 9.5, 6.4 Hz, 1H, H2), 2.09 (dd, J = 9.5, 6.2 Hz, 1H, H1); ^{13}C NMR (151 MHz, $CDCl_3$, 298 K) δ 173.8, 169.9, 169.7, 163.3 (d, J_{C-F} = 249.5 Hz), 148.8, 129.37, 129.35, 127.1 (d, J_{C-F} = 8.2 Hz), 122.6 (d, J_{C-F} = 2.6 Hz), 115.8, 115.7, 52.5, 52.42, 52.41, 49.3, 38.3, 29.5, 26.0, 24.0; ^{19}F NMR (470 MHz, $CDCl_3$, 298 K) δ –111.31 (ArF); IR $υ_{max}$ (cm^{-1}):

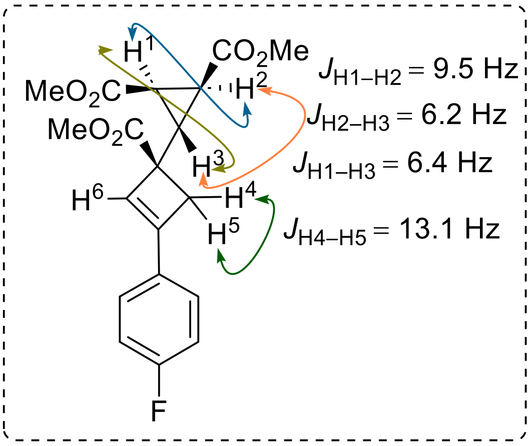

2954, 2926, 1732, 1601, 1506, 1438, 1360, 1257, 1217, 1168; HRMS (ESI+) $[M+Na]^+$ calculated for $[C_{19}H_{19}FO_6Na]^+$: 385.1058, found: 385.1044.

Synthesis and characterization of **3d:**

Synthesized in accordance with *General reaction procedure c* using BCB-**1d** (22 mg, 0.10 mmol, 1 equiv.) and diazo ester **2a** (37 mg, 0.20 mmol, 2 equiv.). All volatiles were removed *in a vacuum* and the crude compound was purified *via* column chromatography using silica gel and pentane/diethyl ether (1:1 v/v; R_f = 0.4). The desired product **3d** was obtained as a colorless liquid. Yield: 31 mg, 0.08 mmol, 81%.

^{1}H NMR (500 MHz, $CDCl_3$, 298 K) δ: 7.31 (d, *J* = 8.4 Hz, 2H, ArH), 7.26 (d, *J* = 8.4 Hz, 2H, ArH), 6.05 (s, 1H, H6), 3.73 (s, 3H, CO_2Me), 3.70 (s, 3H, CO_2Me), 3.68 (s, 3H, CO_2Me), 3.21 (d, *J* = 13.1 Hz, 1H, H4), 2.70 (d, *J* = 13.4 Hz, 1H, H5), 2.69 (t, *J* = 6.1, 1H, H3); 2.11 (dd, *J* = 8.4, 6.2 Hz, 1H, H2), 2.08 (dd, *J* = 8.1, 6.1 Hz, 1H, H1); ^{13}C NMR (126 MHz, $CDCl_3$, 298 K) δ: 173.6, 169.8, 169.7, 148.7, 135.0, 131.4, 128.9, 126.5, 123.9, 52.5, 52.40, 52.38, 49.4, 38.2, 29.4, 25.9, 24.0; IR υ_{max} (cm^{-1}): 2954, 1731,1591, 1489, 1454, 1437, 1404, 1361, 1305, 1250, 1210, 1168, 1115, 1090, 1014; HRMS (ESI+) $[M+Na]^+$ calculated for $[C_{19}H_{19}ClO_6Na]^+$: 401.0762, found: 401.0753.

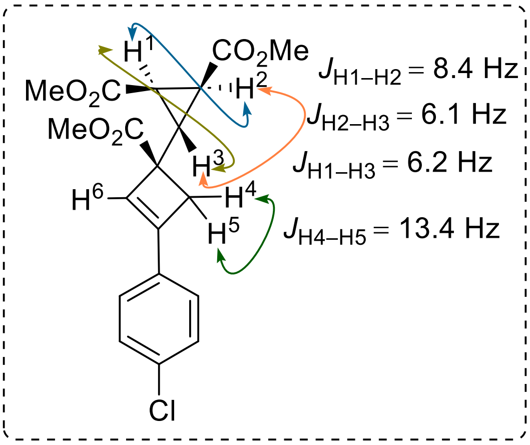

Synthesis and characterization of **3e:**

Synthesized in accordance with *General reaction procedure c* using BCB-**1e** (24 mg, 0.10 mmol, 1 equiv.) and diazo ester **2a** (37 mg, 0.20 mmol, 2 equiv.). All volatiles were removed *in a vacuum* and the crude compound was purified *via* column chromatography using silica gel and pentane/diethyl ether (1:1 v/v; R_f = 0.5). The desired product **3e** was obtained as a colorless liquid. Yield: 33 mg, 0.08 mmol, 83%.

^1H NMR (600 MHz, $CDCl_3$, 298 K) δ: 7.38 (d, 2H, J = 8.4 Hz, ArH), 7.29 (m, 2H, J = 8.4 Hz, ArH), 5.99 (s, 1H, H6), 3.73 (s, 3H, CO_2Me), 3.70 (s, 3H, CO_2Me), 3.67 (s, 3H, CO_2Me), 3.22 (d, J = 13.0 Hz, 1H, H4), 2.71 (d, J = 12.9 Hz, 1H. H5), 2.70 (t, J = 6.4 Hz, 1H, H3), 2.13 (dd, J = 9.3, 6.4 Hz, 1H, H2), 2.08 (dd, J = 9.5, 6.1 Hz, 1H, H1), 1.31 (s, 9H, *t*-butyl); ^{13}C NMR (151 MHz, $CDCl_3$, 298 K) δ: 174.0, 169.9, 169.8, 152.6, 149.9, 130.34, 125.6, 125.0, 122.1, 52.5, 52.4, 49.4, 38.3, 35.0, 31.4, 29.6, 26.0, 23.9; IR $υ_{max}$ (cm^{-1}): 2957, 1736, 1684, 1606, 1559, 1506, 1457, 1438, 1407, 1364, 1285, 1269, 1249, 1211, 1106; HRMS (ESI+) $[M+Na]^+$ calculated for $[C_{23}H_{28}O_6Na]^+$: 423.1778, found: 423.1758.

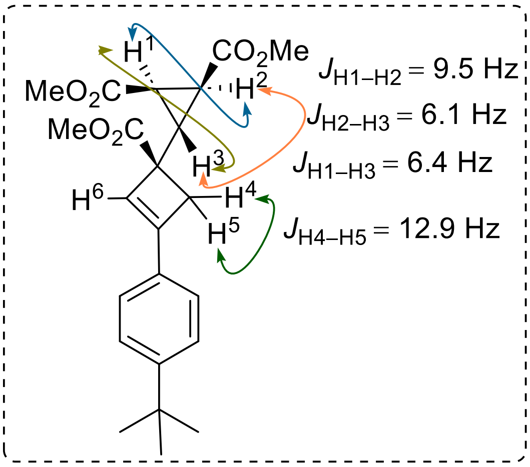

Synthesis and characterization of **3f:**

Synthesized in accordance with *General reaction procedure c* using BCB-**1a** (19 mg, 0.10 mmol, 1 equiv.) and diazo ester **2b** (40 mg, 0.20 mmol, 2 equiv.). All volatiles were removed in a vacuum and the crude compound was purified *via* column chromatography using silica gel and pentane/diethyl ether (1:1 v/v; R_f = 0.8). The desired product **3f** was obtained as a colorless liquid. Yield: 25 mg, 0.07 mmol, 69%.

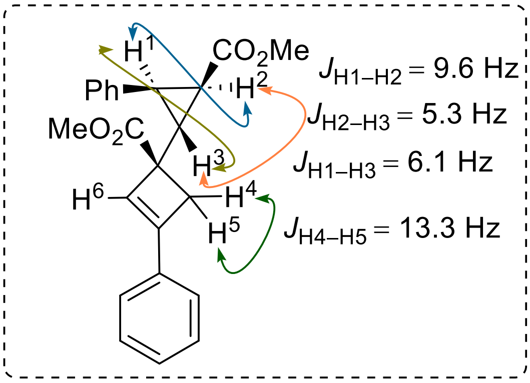

^1H NMR (500 MHz, $CDCl_3$, 298 K) δ: 7.38–7.37 (m, 5H, ArH), 7.30–7.28 (m, 4H, ArH), 7.23–7.19 (m, 1H, ArH), 6.17 (s, 1H, H6), 3.77 (s, 3H, CO_2Me), 3.43 (s, 3H, CO_2Me), 3.27 (d, J = 13.0 Hz, 1H, H4), 2.79 (t, J = 6.1 Hz, 1H, H3), 2.78 (d, J = 13.3 Hz, 1H, H5), 2.62 (dd, J = 9.6, 7.1 Hz, 1H, H2), 2.14 (dd, J = 9.6, 5.3 Hz, 1H, H1); ^{13}C NMR (126 MHz, $CDCl_3$, 298 K) δ: 174.8, 171.0, 149.6, 136.2, 133.3, 129.3, 129.0, 128.6, 128.2, 127.0, 125.2, 123.7, 52.4, 51.7, 50.0, 38.5, 30.6, 29.3, 23.7; IR $υ_{max}$ (cm^{-1}): 2952, 1731, 1603, 1499, 1446, 1363, 1245, 1201, 1164, 1113, 1028; HRMS (ESI+) $[M+Na]^+$ calculated for $[C_{23}H_{22}O_4Na]^+$: 385.1410, found: 385.1391.

Synthesis and characterization of **3g:**

Synthesized in accordance with *General reaction procedure c* using BCB-**1b** (23 mg, 0.10 mmol, 1 equiv.) and diazo ester **2b** (40 mg, 0.20 mmol, 2 equiv.). All volatiles were removed in a vacuum and the crude compound was purified *via* column chromatography using silica

gel and pentane/diethyl ether (1:1 v/v; R_f = 0.8). The desired product **3g** was obtained as a white solid. Yield: 33 mg, 0.08 mmol, 83%.

^{1}H NMR (600 MHz, $CDCl_3$, 298 K) δ: 7.41–7.34 (m, 7H, ArH), 7.33–7.30 (m, 1H, ArH), 7.17 (d, *J* = 8.4 Hz, 2H, ArH), 6.14 (s, 1H, H6), 3.46 (s, 3H, CO_2Me), 3.22 (d, *J* = 12.9 Hz, 1H, H4), 2.71 (d, *J* = 12.9 Hz, 1H, H5), 2.69 (t, *J* = 5.4 Hz, 1H, H3), 2.54 (dd, *J* = 9.5, 7.1 Hz, 1H, H2), 2.13 (dd, *J* = 9.5, 5.3 Hz, 1H, H1), 1.45 (s, 9H, *t*-butyl); ^{13}C NMR (151 MHz, $CDCl_3$, 298 K) δ: 173.3, 170.9, 149.6, 135.3, 133.4, 131.3, 131.0, 128.9, 128.6, 125.1, 123.8, 120.9, 81.3, 51.8, 50.81, 38.1, 30.1, 29.7, 28.2, 23.9; IR υ_{max} (cm^{-1}): 2952, 2925, 1731,1591, 1489, 1445, 1403, 1363, 1286, 1246, 1201, 1162, 1090, 1013.

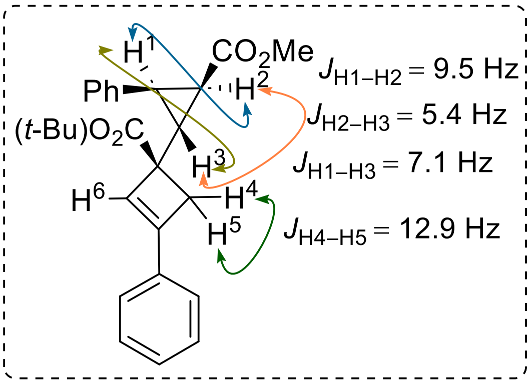

We couldn't obtain the HRMS for this compound. HRMS (ESI+) [M+Na]$^+$ / [M+H]$^+$/ [M]$^+$ calculated for [$C_{26}H_{28}O_4Na$]$^+$ / [$C_{26}H_{29}O_4$]$^+$ / [$C_{26}H_{28}O_4$]$^+$: 427.1885 / 405.2066 / 404.1988, not found

Synthesis and characterization of **3h:**

Synthesized in accordance with *General reaction procedure c* using BCB-**1d** (22 mg, 0.10 mmol, 1 equiv.) and diazo ester **2b** (40 mg, 0.20 mmol, 2 equiv.). All volatiles were removed in a vacuum and the crude compound was purified *via* column chromatography using silica gel and pentane/diethyl ether (3:2 v/v; R_f = 0.8). The desired product **3h** was obtained as a white solid. Yield: 28 mg, 0.07 mmol, 71%.

^{1}H NMR (600 MHz, $CDCl_3$, 298 K) δ: 7.34–7.32 (m, 2H, ArH), 7.30–7.27 (m, 6H, ArH), 7.23–7.19 (m, 1H, ArH), 6.17 (s, 1H, H6), 3.77 (s, 3H, CO_2Me), 3.43 (s, 3H, CO_2Me), 3.24 (d, *J* = 12.9 Hz, 1H, H4), 2.78 (dd, *J* = 7.1, 5.3 Hz, 1H, H3), 2.75 (d, *J* = 12.9 Hz, 1H, H5), 2.61 (dd, *J* = 9.6, 7.0 Hz, 1H, H2), 2.12 (dd, *J* = 9.6, 5.3 Hz, 1H, H1); ^{13}C NMR (151 MHz, $CDCl_3$, 298 K) δ: 174.5, 170.9, 148.5, 136.1, 134.8, 131.7, 129.3, 128.9, 128.3, 127.1, 126.5, 124.4, 52.5, 51.7, 50.1, 38.5, 30.6, 29.2, 23.8; IR υ_{max} (cm^{-1}): 2952, 2925, 1731,1591, 1489, 1445, 1403, 1363, 1286, 1246, 1201, 1162, 1114, 1090, 1013; HRMS (ESI+) [M+H]$^+$ calculated for [$C_{23}H_{22}ClO_4$]$^+$: 397.1201, found: 397.1204.

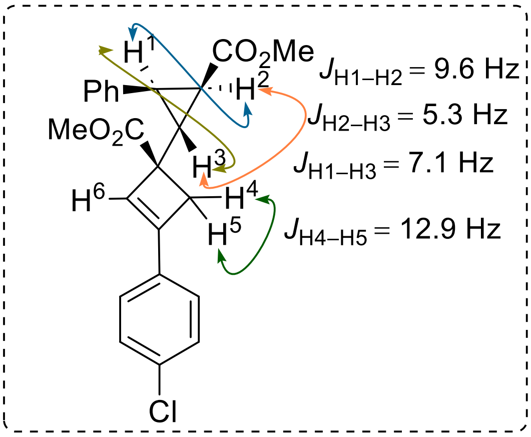

Synthesis and characterization of **3i**:

Synthesized in accordance with *General reaction procedure c* using BCB-**1a** (19 mg, 0.10 mmol, 1 equiv.) and diazo ester **2c** (56 mg, 0.20 mmol, 2 equiv.). All volatiles were removed in a vacuum and the crude compound was purified *via* column chromatography using silica gel and pentane/diethyl ether (3:2 v/v; R_f = 0.7). The desired product **3i** was obtained as a white solid. Yield: 30 mg, 0.07 mmol, 68%.

^{1}H NMR (600 MHz, $CDCl_3$, 298 K) δ: 7.44–7.30 (m, 7H, ArH), 7.17 (d, *J* = 9.6 Hz, 2H, ArH), 6.14 (s, 1H, H6), 3.76 (s, 3H, CO_2Me), 3.46 (s, 3H, CO_2Me), 3.27 (d, *J* = 12.9 Hz, 1H, H4), 2.76 (d, *J* = 12.7 Hz, 1H, H5), 2.73 (t, *J* = 4.3 Hz, 1H, H3), 2.53 (dd, *J* = 9.5, 7.0 Hz, 1H, H2), 2.13 (dd, *J* = 9.5, 6.2 Hz, 1H, H1);^{13}C NMR (151 MHz, $CDCl_3$, 298 K) δ: 174.7, 170.8, 149.9, 135.3, 133.2, 131.4, 131.1, 129.1, 128.6, 125.2, 123.3, 121.0, 52.5, 51.9, 49.9, 38.5, 30.0, 29.4, 23.7; IR υ_{max} (cm^{-1}): 3028, 2951, 1732, 1575, 1491, 1448, 1435, 1397, 1362, 1244, 1201, 1159, 1114, 1072; HRMS (ESI+) $[M+H]^+$ calculated for $[C_{23}H_{22}BrO_4]^+$: 441.0696, found: 441.0699.

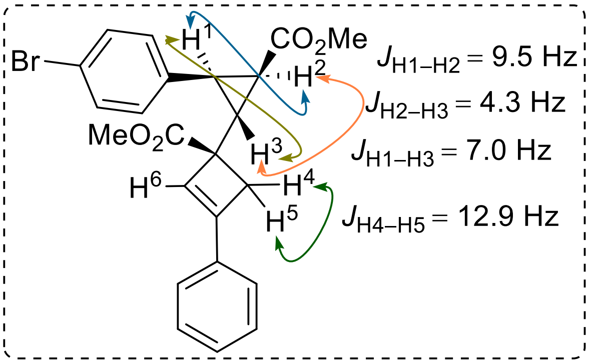

Synthesis and characterization of **3j**:

Synthesized in accordance with *General reaction procedure c* using BCB-**1b** (23 mg, 0.10 mmol, 1 equiv.) and diazo ester **2c** (56 mg, 0.20 mmol, 2 equiv.). All volatiles were removed in a vacuum and the crude compound was purified *via* column chromatography using silica gel and pentane/diethyl ether (1:1 v/v; R_f = 0.8). The desired product **3j** was obtained as a colorless solid. Yield: 34 mg, 0.07 mmol, 70%.

^{1}H NMR (600 MHz, $CDCl_3$, 298 K) δ: 7.39–7.34 (m, 4H, ArH), 7.33–7.27 (m, 4H, ArH), 7.22–7.19 (m, 1H, ArH), 6.17 (s, 1H, H6), 3.43 (s, 3H, CO_2Me), 3.22 (d, *J* = 12.9 Hz, 1H, H4), 2.75 (t, *J* = 7.0 Hz, 1H, H3), 2.72 (d, *J* = 12.4 Hz, 1H, H5), 2.63 (dd, *J* = 9.6, 7.1 Hz, 1H, H2), 2.13 (dd, *J* = 9.6, 5.3, 1H, H1), 1.47 (s, 9H, *t*-butyl); ^{13}C NMR (151 MHz, $CDCl_3$, 298 K) δ: 173.4, 171.1, 149.3, 136.2, 133.5, 129.3, 128.8, 128.6, 128.1, 126.9, 125.1, 124.2, 81.2, 51.7, 50.9, 38.2, 30.7, 29.6, 28.2, 24.0; IR υ_{max} (cm^{-1}): 2978, 2952, 1722,1604, 1576, 1499, 1490, 1446, 1392, 1367, 1254, 1200, 1167, 1028. HRMS (ESI+) $[M+Na]^+$ / $[M+H]^+$ calculated for $[C_{26}H_{28}BrO_4Na]^+$ / $[C_{26}H_{28}BrO_4]^+$**:** 505.0990 / 483.1171 not found.

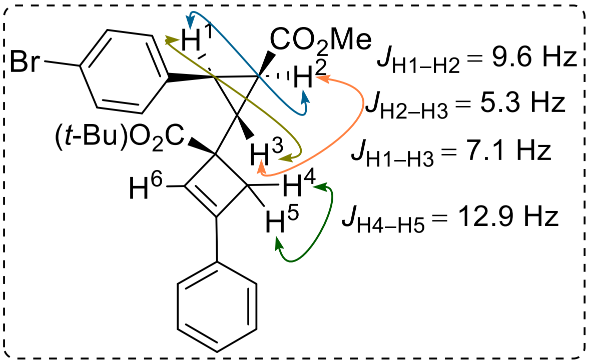

Synthesis and characterization of **3k**:

Synthesized in accordance with *General reaction procedure c* using BCB-**1a** (19 mg, 0.10 mmol, 1 equiv.) and diazo ester **2d** (49 mg, 0.20 mmol, 2 equiv.). All volatiles were removed in a vacuum and the crude compound was purified *via* column chromatography using silica gel and pentane/diethyl ether (3:2 v/v; R_f = 0.6). The desired product **3k** was obtained as a colorless liquid. Yield: 25 mg, 0.06 mmol, 61%.

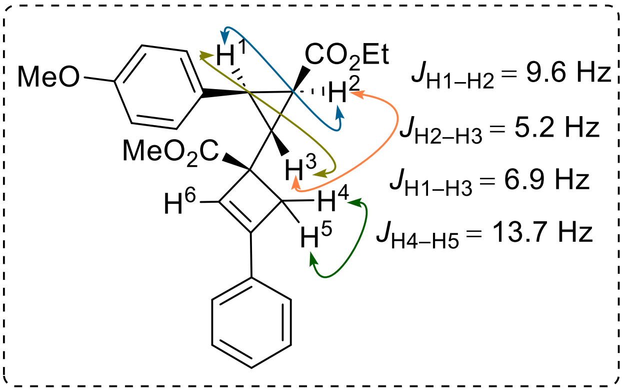

^{1}H NMR (600 MHz, $CDCl_3$, 298 K) δ 7.38–7.35 (m, 4H, ArH), 7.33–7.31 (m, 1H, ArH), 7.21 (d, *J* = 8.6 Hz, 2H, ArH), 6.81 (d, *J* = 8.6 Hz, 2H, ArH), 6.15 (s, 1H, H6), 3.88 (q, *J* = 7.8, 2H, Et), 3.78 (s, 3H, CO_2Me), 3.76 (s, 3H, CO_2Me), 3.25 (d, *J* = 13.7 Hz, 1H, H4), 2.77 (d, *J* = 13.0 Hz, 1H, H5), 2.73 (dd, *J* = 6.9, 5.2 Hz, 1H, H3), 2.56 (dd, *J* = 9.5, 6.9 Hz, 1H, H2), 2.07 (dd, *J* = 9.5, 5.2, 1H, H1), 1.00 (t, *J* = 7.1 Hz, 3H, Et); ^{13}C NMR (151 MHz, $CDCl_3$, 298 K) δ: 174.9, 170.6, 158.6, 149.6, 133.3, 130.4, 129.0, 128.6, 128.3, 125.2, 123.7, 113.6, 60.5, 55.4, 52.4, 50.1, 38.5, 29.9, 29.1, 23.8, 14.2; IR υ_{max} (cm^{-1}): 2984, 2837, 1730,1612, 1581, 1516, 1490, 1448, 1373, 1350, 1248, 1194, 1179, 1158, 1072; HRMS (ESI+) $[M+Na]^+$ calculated for $[C_{25}H_{26}O_5Na]^+$: 429.1673, found: 429.1663.

Synthesis and characterization of **3l**:

Synthesized in accordance with *General reaction procedure c* using BCB-**1b** (23 mg, 0.10 mmol, 1 equiv.) and diazo ester **2d** (49 mg, 0.20 mmol, 2 equiv.). All volatiles were removed in a vacuum and the crude compound was purified *via* column chromatography using silica gel and pentane/diethyl ether (1:1 v/v; R_f = 0.7). The desired product **3l** was obtained as a white solid. Yield: 29 mg, 0.06 mmol, 64%.

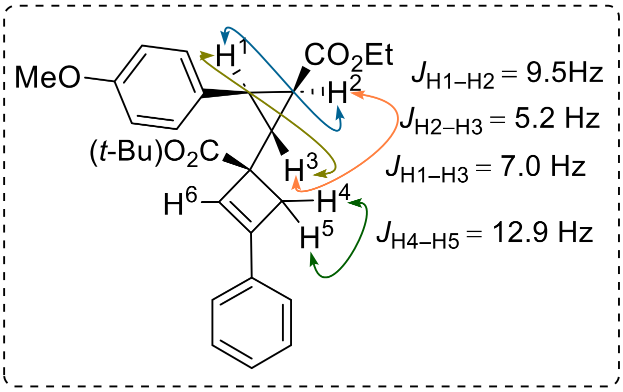

^{1}H NMR (500 MHz, $CDCl_3$, 298 K) δ 7.39–7.34 (m, 4H, ArH), 7.32–7.29 (m, 1H, ArH), 7.21 (d, *J* = 8.8 Hz, 2H, ArH), 6.81 (d, *J* = 8.7 Hz, 2H, ArH), 6.15 (s, 1H, H6), 3.88 (qd, *J* = 7.1, 1.3 Hz, 2H, Et), 3.78 (s, 3H, OMe), 3.21 (d, *J* = 12.9 Hz, 1H, H4), 2.72 (d, *J* = 12.9 Hz, 1H, H5), 2.69 (t, *J* = 5.2 Hz, 1H, H3), 2.57 (dd, *J* = 9.5, 7.0 Hz, 1H, H2), 2.06 (dd, *J* = 9.5, 5.2 Hz, 1H, H1), 1.00 (t, *J* = 7.1 Hz, 3H, Et); ^{13}C NMR (126 MHz, $CDCl_3$, 298 K) δ: 173.5, 170.7, 158.5, 149.2, 133.5, 130.4, 128.8, 128.5, 128.3, 125.1, 124.3,

113.5, 81.2, 60.4, 55.3, 51.0, 38.2, 30.0, 29.4, 28.2, 24.0, 14.2; IR υ_{max} (cm^{-1}): 2976, 2931, 2836, 1722,1613, 1581, 1516, 1490, 1448, 1392, 1369, 1349, 1296, 1250, 1173, 1071; HRMS (ESI+) $[M+H]^+$ calculated for $[C_{28}H_{33}O_5]^+$: 449.2323, found: 449.2333.

Synthesis and characterization of **3m**:

Synthesized in accordance with *General reaction procedure c* using BCB-**1a** (19 mg, 0.10 mmol, 1 equiv.) and diazo ester **2e** (25 mg, 0.20 mmol, 2 equiv.). All volatiles were removed in a vacuum and the crude compound was purified *via* column chromatography using silica gel and pentane/diethyl ether (3:2 v/v; R_f = 0.8). The desired product **3m** was obtained as a colorless liquid. Yield: 12 mg, 0.04 mmol, 41%.

^1H NMR (600 MHz, $CDCl_3$, 298 K) δ 7.37–7.33 (m, 4H, ArH), 7.32–7.29 (m, 1H, ArH), 6.08 (s, 1H, H6), 3.73 (s, 3H, CO_2Me), 3.66 (s, 3H, CO_2Me), 3.18 (d, *J* = 13.0 Hz, 1H, H4), 2.68 (d, *J* = 12.9 Hz, 1H, H5), 2.10 (ddd, *J* = 9.0, 6.5, 4.3 Hz, 1H, H3), 1.68 (dt, *J* = 8.5, 4.6 Hz, 1H, H2), 1.29 (dd, *J* = 9.2, 4.7 Hz, 1H, H1), 1.21 (d, *J* = 6.2 Hz, 1H, H1'); ^{13}C NMR (151 MHz, $CDCl_3$, 298 K) δ: 174.7, 174.5, 149.1, 133.3, 128.9, 128.6, 125.1, 124.1, 52.4, 52.0, 50.2, 38.4, 26.3, 15.9, 13.2; IR υ_{max} (cm^{-1}): 3016, 2954, 1732, 1599, 1548, 1514, 1449, 1402, 1275, 1259, 1214, 1177; HRMS (GC-EI+) $[M]^+$ calculated for $[C_{17}H_{18}O_4]^+$: 286.1205, found: 286.1199.

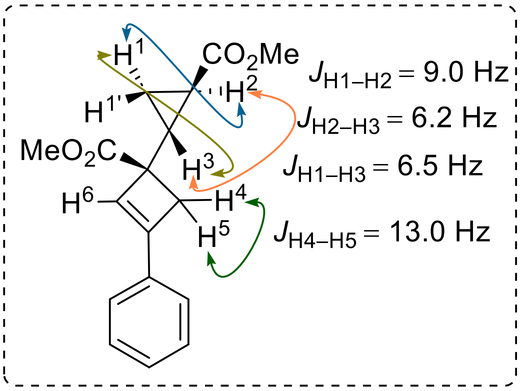

Synthesis and characterization of **3n**:

Synthesized in accordance with *General reaction procedure c* using BCB-**1q** (26 mg, 0.10 mmol, 1 equiv.) and diazo ester **2a** (37 mg, 0.20 mmol, 2 equiv.). All volatiles were removed in a vacuum and the crude compound was purified *via* column chromatography using silica gel and pentane/diethyl ether (1:1 v/v; R_f = 0.4). The desired product **3n** was obtained as a colorless solid. Yield: 37 mg, 0.09 mmol, 90%.

^{1}H NMR (500 MHz, $CDCl_3$, 298 K) δ 7.36–7.33 (m, 4H, ArH), 7.32–7.27 (m, 1H, ArH), 6.22 (s, 1H, H6), 4.03 (septet, *J* = 6.6 Hz, 1H, *iso*-propyl), 3.68 (s, 3H, CO_2Me), 3.67 (s, 3H, CO_2Me), 3.35 (septet, *J* = 6.8 Hz, 1H, *iso*-propyl), 3.17 (d, *J* = 12.4 Hz, 1H, H4), 2.84 (d, *J* = 12.4 Hz, 1H, H5), 2.54 (t, *J* = 6.2 Hz, 1H, H3), 2.23 (dd, *J* = 9.6, 6.3 Hz, 1H, H2), 2.18 (dd, *J* = 9.6, 6.0 Hz, 1H, H1), 1.41 (t, *J* = 7.0 Hz, 6H, *iso*-propyl), 1.33 (d, *J* = 6.7 Hz, 3H, *iso*-propyl), 1.28 (d, *J* = 6.5 Hz, 3H, *iso*-propyl); ^{13}C NMR (126 MHz, $CDCl_3$, 298 K) δ 171.5, 170.1, 169.8, 146.5, 133.2, 128.8, 128.5, 128.1, 127.9, 127.7, 125.3, 125.1, 52.4, 52.3, 50.2, 49.1, 46.4, 38.5, 30.0, 25.9, 24.3, 20.9, 20.8, 20.6, 20.2; IR $υ_{max}$ (cm^{-1}): 2968, 1742, 1623, 1490, 1438, 1370, 1331, 1370, 1331, 1295, 1215, 1165, 1105, 1063; HRMS (ESI+) $[M+H]^+$ calculated for $[C_{24}H_{32}NO_5]^+$: 414.2275, found: 414.2271.

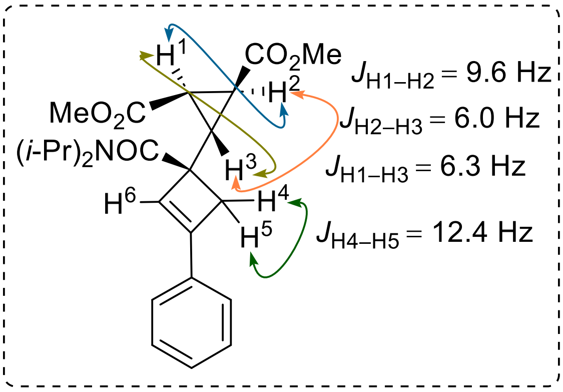

Synthesis and characterization of **3o**:

Synthesized in accordance with *General reaction procedure c* using BCB-**1r** (30 mg, 0.10 mmol, 1 equiv.) and diazo ester **2a** (37 mg, 0.20 mmol, 2 equiv.), All volatiles were removed in a vacuum and the crude compound was purified *via* column chromatography using silica gel and pentane/diethyl ether (1:1 v/v; R_f = 0.3). The desired product **3o** was obtained as a light-yellow solid. Yield: 29 mg, 0.06 mmol, 63%.

^{1}H NMR (600 MHz, $CDCl_3$, 298 K) δ 8.20 (d, *J* = 8.8 Hz, 2H, ArH), 7.46 (d, *J* = 8.7 Hz, 2H, ArH), 6.47 (s, 1H, H6), 3.99 (septet, *J* = 7.0 Hz, 1H, *iso*-propyl), 3.69 (s, 3H, CO_2Me), 3.67 (s, 3H, CO_2Me), 3.37 (septet, *J* = 7.1 Hz, 1H, *iso*-propyl), 3.21 (d, *J* = 12.3 Hz, 1H, H4), 2.91 (d, *J* = 12.4 Hz, 1H, H5), 2.55 (t, *J* = 6.2 Hz, 1H, H3), 2.24 (dd, *J* = 9.6, 6.3 Hz, 1H, H2), 2.15 (dd, *J* = 9.5, 6.1 Hz, 1H, H1), 1.41 (d, *J* = 8.8 Hz, 6H, *iso*-propyl), 1.34 (d, *J* = 6.5 Hz, 3H, *iso*-propyl), 1.29 (d, *J* = 6.5 Hz, 3H, *iso*-propyl); ^{13}C NMR (151 MHz, $CDCl_3$, 298 K) δ 170.7, 169.8, 169.5, 147.7, 144.4, 138.9, 131.3, 125.9, 124.0, 52.5, 52.4, 50.7, 49.2, 46.5, 38.5, 29.7, 25.8, 24.5, 20.87, 20.85, 20.6, 20.2; IR $υ_{max}$ (cm^{-1}): 2966, 1744, 1623, 1519, 1439, 1371, 1337 ,1296, 1253, 1215, 1166, 1109, 1063; HRMS (ESI+) $[M+H]^+$ calculated for $[C_{24}H_{31}N_2O_7]^+$: 459.2126, found: 459.2121.

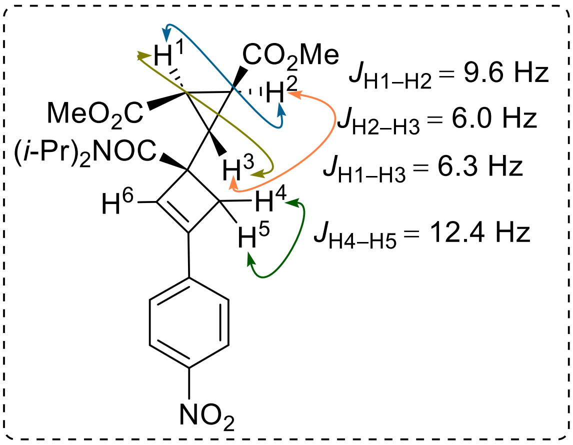

Synthesis and characterization of **3p**:

Synthesized in accordance with *General reaction procedure c* using BCB-**1s** (32 mg, 0.10 mmol, 1 equiv.) and diazo ester **2a** (37 mg, 0.20 mmol, 2 equiv.). All volatiles were removed in a vacuum and the crude compound was purified *via* column chromatography using silica gel and pentane/diethyl ether (1:1 v/v; R_f = 0.4). The desired product **3p** was obtained as a colorless liquid. Yield: 29 mg, 0.06 mmol, 61%.

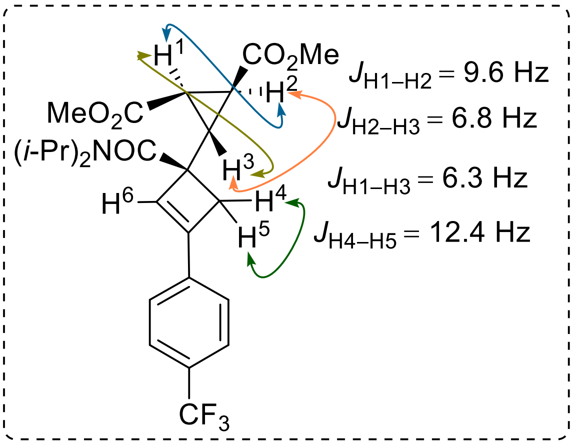

^{1}H NMR (500 MHz, $CDCl_3$, 298 K) δ 7.59 (d, *J* = 8.1 Hz, 2H, ArH), 7.43 (d, *J* = 8.0 Hz, 2H, ArH), 6.36 (s, 1H, H6), 4.01 (septet, *J* = 6.6 Hz, 1H, *iso*-propyl), 3.68 (s, 3H, CO_2Me), 3.67 (s, 3H, CO_2Me), 3.36 (septet, *J* = 6.8 Hz, 1H, *iso*-propyl), 3.18 (d, *J* = 12.4, Hz, 1H, H4), 2.87 (d, *J* = 12.4 Hz, 1H, H5), 2.55 (t, *J* = 6.2 Hz, 1H, H3), 2.23 (d, *J* = 9.6, 6.3 Hz, 1H, H2), 2.15 (dd, *J* = 9.6, 6.0 Hz, 1H, H1), 1.42 (d, *J* = 6.8 Hz, 6H, *iso*-propyl), 1.34 (d, *J* = 6.6 Hz, 3H, *iso*-propyl), 1.29 (d, *J* = 6.5 Hz, 3H, *iso*-propyl); ^{13}C NMR (126 MHz, $CDCl_3$, 298 K) δ 171.1, 169.9, 169.7, 169.6, 145.1, 130.6, 130.3, 128.6, 125.56 (q, *J* = 3.9 Hz, $ArCF_3$), 125.74, 123.1, 52.5, 52.3, 50.5, 49.2, 46. 4, 38.5, 29.8, 25.8, 24.4, 20.9, 20.6, 20.2; ^{19}F NMR (471 MHz, $CDCl_3$, 298 K) δ –62.64 ($ArCF_3$); IR $υ_{max}$ (cm^{-1}): 2971, 1743, 1619, 1440, 1371, 1325, 1298, 1251, 1214, 1168, 1129, 1109, 1068; HRMS (GC-EI+) $[M]^+$ calculated for $[C_{25}H_{31}F_3NO_5]^+$: 481.2076, found: 481.2093.

Synthesis and characterization of **3q**:

Synthesized in accordance with *General reaction procedure c* using BCB-**1t** (29 mg, 0.10 mmol, 1 equiv.) and diazo ester **2a** (37 mg, 0.20 mmol, 2 equiv.). All volatiles were removed in a vacuum and the crude compound was purified *via* column chromatography using silica gel and pentane/diethyl ether (1:1 v/v; R_f = 0.4). The desired product **3q** was obtained as a white solid Yield: 33 mg, 0.07 mmol, 74%.

^{1}H NMR (500 MHz, $CDCl_3$, 298 K) δ 7.30 (d, *J* = 8.5 Hz, 2H, ArH), 7.26 (d, *J* = 8.8 Hz, 2H, ArH), 6.22 (s, 1H, H6), 4.00 (septet, *J* = 6.7 Hz, 1H, *iso*-propyl), 3.68 (s, 3H, CO_2Me), 3.67 (s, 3H, CO_2Me), 3.35 (septet, *J* = 6.8 Hz, 1H, *iso*-propyl), 3.14 (d, *J* = 12.4 Hz, 1H, H4), 2.82 (d, *J* = 12.4 Hz, 1H, H5), 2.53 (t, *J* = 6.2 Hz, 1H, H3), 2.21 (dd, *J* = 9.6, 6.3 Hz, 1H, H2), 2.15 (dd, *J* = 9.1, 6.0 Hz, 1H, H1), 1.40 (d, *J* = 6.7 Hz, 6H, *iso*-propyl), 1.32 (d, *J* = 6.5 Hz, 3H, *iso*-propyl), 1.27 (d, *J* = 6.5 Hz, 3H, *iso*-propyl); ^{13}C NMR (126 MHz, $CDCl_3$, 298 K) δ 171.3, 170.0, 169.7, 145.3, 134.6, 131.7, 128.8, 126.5, 126.1, 52.4, 52.3, 50.2, 49.1, 46.4, 38.5, 29.9, 25.9, 24.3, 20.9, 20.6, 20.2; IR υ_{max} (cm^{-1}): 2969, 1744, 1624, 1550, 1489, 1439, 1403, 1371, 1297, 1249, 1215, 1165, 1089; HRMS (ESI+) $[M+H]^+$ calculated for $[C_{24}H_{31}ClNO_5]^+$: 448.1885, found: 448.1878.

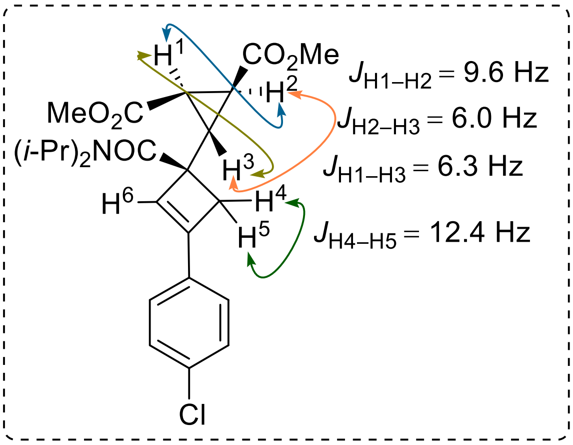

Synthesis and characterization of **3r**:

Synthesized in accordance with *General reaction procedure c* using BCB-**1u** (29 mg, 0.10 mmol, 1 equiv.) and diazo ester **2a** (37 mg, 0.20 mmol, 2 equiv.). All volatiles were removed in a vacuum and the crude compound was purified *via* column chromatography using silica gel and pentane/diethyl ether (1:1 v/v; R_f = 0.5). The desired product **3r** was obtained as a white solid Yield: 38 mg, 0.08 mmol, 85%.

^{1}H NMR (500 MHz, $CDCl_3$, 298 K) δ 7.24 (d, *J* = 8.2 Hz, 2H, ArH), 7.14 (d, *J* = 7.8 Hz, 2H, ArH), 6.14 (s, 1H, H6), 4.03 (septet, *J* = 6.6 Hz, 1H, *iso*-propyl), 3.67 (s, 3H, CO_2Me), 3.65 (s, 3H, CO_2Me), 3.33 (septet, *J* = 6.7 Hz, 1H, *iso*-propyl), 3.15 (d, *J* = 12.4 Hz, 1H, H4), 2.81 (d, *J* = 12.3 Hz, 1H, H5), 2.53 (t, *J* = 6.2 Hz, 1H, H3), 2.34 (s, 3H, Me), 2.21 (dd, *J* = 9.6, 6.3 Hz, 1H, H2), 2.17 (dd, *J* = 9.6, 6.0 Hz, 1H, H1), 1.41 (d, *J* = 6.7 Hz, 6H, *iso*-propyl), 1.33 (d, *J* = 6.7 Hz, 3H, *iso*-propyl), 1.26 (d, *J* = 6.3 Hz, 3H, *iso*-propyl); ^{13}C NMR (126 MHz, $CDCl_3$, 298 K) δ 171.7, 170.2, 169.8, 146.5, 138.8, 130.6, 129.2, 125.1, 124.0, 52.4, 50.1, 49.0, 46.3, 38.6, 30.0, 25.9, 24.2, 21.6, 20.84, 20.81, 20.6, 20.2; IR υ_{max} (cm^{-1}): 2966, 2928, 1745, 1691, 1627, 1551, 1510, 1439, 1370, 1331, 1298, 1276, 1253, 1214, 1166, 1038; HRMS (ESI+) $[M+H]^+$ calculated for $[C_{25}H_{34}NO_5]^+$: 428.2437, found: 428.2433.

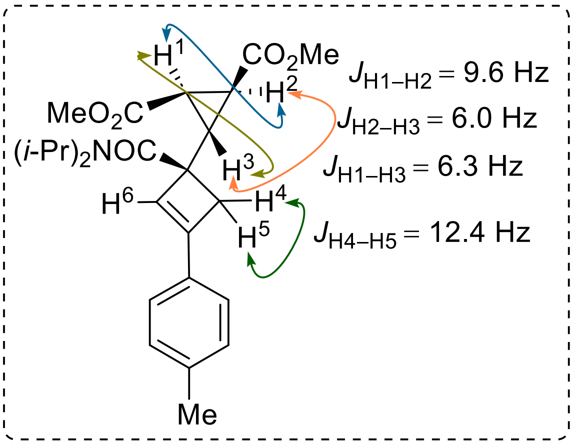

Synthesis and characterization of **3s**:

Synthesized in accordance with *General reaction procedure c* using BCB-**1v** (29 mg, 0.10 mmol, 1 equiv.) and diazo ester **2a** (37 mg, 0.20 mmol, 2 equiv.). All volatiles were removed in a vacuum and the crude compound was purified *via* column chromatography using silica gel and pentane/diethyl ether (1:1 v/v; R_f = 0.3). The desired product **3s** was obtained as a colorless liquid. Yield: 41 mg, 0.09 mmol, 93%.

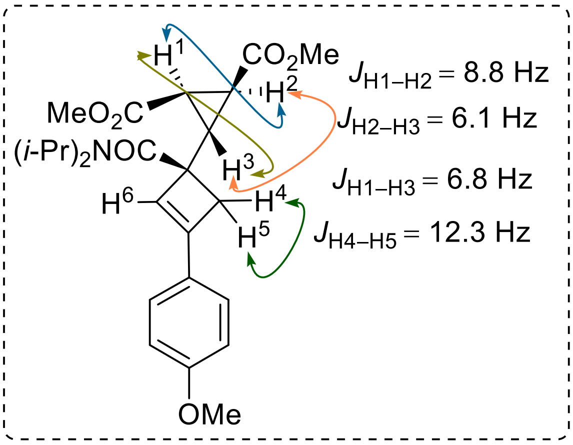

^{1}H NMR (500 MHz, $CDCl_3$, 298 K) δ 7.27 (d, *J* = 8.8 Hz, 2H, ArH), 6.86 (d, *J* = 8.7 Hz, 2H, ArH), 6.06 (s, 1H, H6), 4.02 (septet, *J* = 6.3 Hz, 1H, *iso*-propyl), 3.81 (s, 3H, OMe), 3.67 (s, 3H, CO_2Me), 3.66 (s, 3H, CO_2Me), 3.35 (septet, *J* = 6.9 Hz, 1H, *iso*-propyl), 3.14 (d, *J* = 12.3 Hz, 1H, H4), 2.80 (d, *J* = 12.3 Hz, 1H, H5), 2.52 (t, *J* = 6.1 Hz, 1H, H3), 2.21 (dd, *J* = 8.7, 5.4 Hz, 1H, H2), 2.18 (dd, *J* = 8.8, 5.1 Hz, 1H, H1), 1.41 (d, *J* = 6.8 Hz, 3H, *iso*-propyl), 1.40 (d, *J* = 6.7 Hz, 3H, *iso*-propyl),1.32 (d, *J* = 6.6 Hz, 3H, *iso*-propyl), 1.27 (d, *J* = 6.6 Hz, 3H, *iso*-propyl); ^{13}C NMR (126 MHz, $CDCl_3$, 298 K) δ 171.7, 170.2, 169.8, 160.1, 146.1, 130.9, 126.6, 126.3, 122.4, 113.9, 55.5, 52.4, 52.3, 50.0, 49.0, 46.3, 38.6, 30.1, 25.9, 24.2, 20.9, 20.6, 20.3; IR υ_{max} (cm^{-1}): 2967, 1743, 1625, 1575, 1509, 1439, 1370, 1330, 1252, 1214, 1171, 1109, 1061; HRMS (ESI+) $[M+H]^+$ calculated for $[C_{25}H_{34}NO_6]^+$: 444.2381, found: 444.2373.

Synthesis and characterization of **3t**:

Synthesized in accordance with *General reaction procedure c* using BCB-**1w** (29 mg, 0.10 mmol, 1 equiv.) and diazo ester **2a** (37 mg, 0.20 mmol, 2 equiv.). All volatiles were removed in a vacuum and the crude compound was purified *via* column chromatography using silica gel and pentane/diethyl ether (1:1 v/v; R_f = 0.3). The desired product **3t** was obtained as a colorless liquid. Yield: 26 mg, 0.06 mmol, 59%.

^1H NMR (500 MHz, $CDCl_3$, 298 K) δ 7.26–7.23 (m, 1H, ArH), 6.93 (dt, J = 7.6, 1.2 Hz, 1H, ArH), 6.88–6.81 (m, 2H, ArH), 6.22 (s, 1H, H6), 4.02 (septet, J = 6.6 Hz, 1H, *iso*-propyl), 3.80 (s, 3H, OMe), 3.67 (s, 3H, CO_2Me), 3.66 (s, 3H, CO_2Me), 3.35 (septet, J = 6.8 Hz, 1H, *iso*-propyl), 3.14 (d, J = 12.4 Hz, 1H, H4), 2.83 (d, J = 12.4 Hz, 1H, H5), 2.53 (t, J = 6.2 Hz, 1H, H3), 2.22 (dd, J = 9.6, 6.3 Hz, 1H, H2), 2.17 (dd, J = 9.6, 6.0 Hz, 1H, H1), 1.40 (d, J = 6.6 Hz, 6H, *iso*-propyl), 1.33 (d, J = 6.5 Hz, 3H, *iso*-propyl), 1.27 (d, J = 6.5 Hz, 3H, *iso*-propyl); ^{13}C NMR (126 MHz, $CDCl_3$, 298 K) δ 171.4, 170.1, 169.7, 159.8, 146.3, 134.6, 129.6, 125.7, 117.7, 114.7, 110.3, 55.4, 52.4, 52.3, 50.1, 49.1, 46.3, 38.6, 30.0, 25.9, 24.2, 20.8, 20.6, 20.2; IR υ_{max} (cm^{-1}): 2968, 1742, 1622, 1580, 1438, 1370 ,1328 ,1297, 1251, 1215, 1165, 1104, 1045; HRMS (GC-EI+) $[M]^+$ calculated for $[C_{25}H_{33}NO_6]^+$: 443.2307, found: 443.2302.

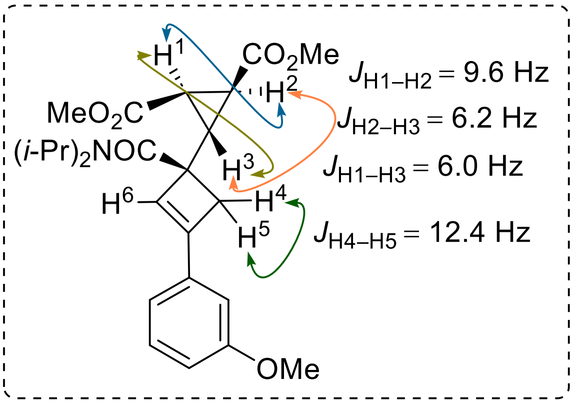

Synthesis and characterization of **3u:**

Synthesized in accordance with *General reaction procedure c* using BCB-**1x** (39 mg, 0.10 mmol, 1 equiv.) and diazo ester **2a** (37 mg, 0.20 mmol, 2 equiv.). All volatiles were removed in a vacuum and the crude compound was purified *via* column chromatography using silica gel and pentane/diethyl ether (4:1 v/v; R_f = 0.7). The desired product **3u** was obtained as a colorless liquid. Yield: 51 mg, 0.9 mmol, 94%.

^1H NMR (500 MHz, $CDCl_3$) δ 7.18 (t, J = 7.8 Hz, 1H, ArH), 6.93 (dt, J = 7.6, 1.3 Hz, 1H,ArH), 6.83–6.67 (m, 2H, ArH), 6.18 (s, 1H, H6), 4.02 (septet, J = 6.5 Hz, 1H, *iso-propyl*), 3.67 (s, 3H, CO_2Me), 3.66 (s, 3H, CO_2Me), 3.35 (septet, J = 6.8 Hz, 1H, *iso*-propyl), 3.13 (d, J = 12.3 Hz, 1H, H4), 2.81 (d, J = 12.4 Hz, 1H, H5), 2.52 (t, J = 6.2 Hz, 1H, H3), 2.21 (dd, J = 9.6, 6.3 Hz, 1H, H2), 2.17 (dd, J = 9.6, 6.1 Hz, 1H, H1), 1.40 (d, J = 8.6, 6H, *iso*-propyl), 1.32 (d, J = 6.6 Hz, 3H, *iso*-propyl), 1.27 (d, J = 6.5 Hz, 3H, *iso*-propyl), 0.98 (s, 9H, OTBS), 0.19 (s, 6H, OTBS); ^{13}C NMR (126 MHz, $CDCl_3$, 298 K) δ 171.5, 170.1, 169.8, 155.9, 146.3, 134.6, 129.5, 125.5, 120.6, 118.3, 116.6, 52.4, 52.3, 50.1, 49.0, 46.3, 38.5, 30.0, 25.8, 24.3, 20.84, 20.83 20.6, 20.2, 18.3, –4.24; IR υ_{max} (cm^{-1}): 2931, 2897, 1746, 1621, 1440, 1372 , 1299, 1258, 1216, 1165, 1003; HRMS (ESI+) $[M+H]^+$ calculated for $[C_{30}H_{46}NO_6Si]$+: 543.3089, found: 543.3076.

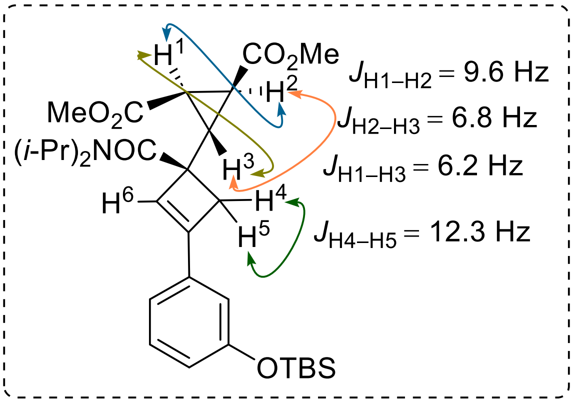

Synthesis and characterization of **3v:**

Synthesized in accordance with *General reaction procedure c* using BCB-**1y** (31 mg, 0.10 mmol, 1 equiv.) and diazo ester **2a** (37 mg, 0.20 mmol, 2 equiv.). All volatiles were removed in a vacuum and the crude compound was purified *via* column chromatography using silica gel and pentane/diethyl ether (1:1 v/v; R_f = 0.4). The desired product **3v** was obtained as an off-white solid. Yield: 36 mg, 0.07 mmol, 79%.

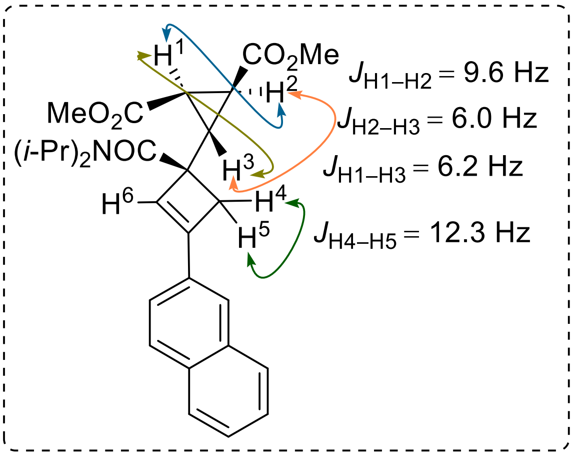

^{1}H NMR (500 MHz, $CDCl_3$, 298 K) δ 7.82–7.76 (m, 3H, ArH), 7.66 (s, 1H, ArH), 7.54 (dd, J = 8.4, 1.6 Hz, 1H, ArH), 7.48–7.46 (m, 2H, ArH), 6.33 (s, 1H, H6), 4.08 (septet, J = 6.6 Hz, 1H, *iso*-propyl), 3.69 (s, 3H, CO_2Me), 3.66 (s, 3H, CO_2Me), 3.37 (septet, J = 6.8 Hz, 1H, *iso*-propyl), 3.28 (d, J = 12.3 Hz, 1H, H4), 2.96 (d, J = 12.2 Hz, 1H, H5), 2.57 (t, J = 6.2 Hz, 1H, H3), 2.26 (dd, J = 9.6, 6.3 Hz, 1H, H2), 2.21 (dd, J = 9.6, 6.0 Hz, 1H, H1), 1.42 (d, J = 6.6 Hz, 6H, *iso*-propyl), 1.36 (d, J = 6.5 Hz, 3H, *iso*-propyl), 1.31 (d, J = 6.5 Hz, 3H, *iso*-propyl); ^{13}C NMR (126 MHz, $CDCl_3$, 298 K) δ 171.5, 170.1, 169.8, 146.5, 133.5, 133.3, 130.7, 128.3, 128.2, 128.0, 126.61, 126.60, 126.0, 124.5, 122.8, 52.4, 52.3, 50.3, 49.1, 46.4, 38.6, 30.1, 25.9, 24.3, 20.9, 20.6, 20.3; IR υ_{max} (cm^{-1}): 2968, 1743, 1623, 1506, 1438, 1370, 1331, 1296, 1252, 1214, 1165, 1100, 1061; HRMS (ESI+) $[M+Na]^+$ calculated for $[C_{28}H_{33}NO_5Na]^+$: 486.2251, found: 486.2241.

Synthesis and characterization of **3w:**

Synthesized in accordance with *General reaction procedure c* using BCB-**1z** (40 mg, 0.10 mmol, 1 equiv.) and diazo ester **2a** (37 mg, 0.20 mmol, 2 equiv.). All volatiles were removed in a vacuum and the crude compound was purified via column chromatography using silica gel and pentane/diethyl ether (4:1 v/v; R_f = 0.7). The desired products **3w** was obtained as a colorless solid. Yield: 46 mg, 0.08 mmol, 84%

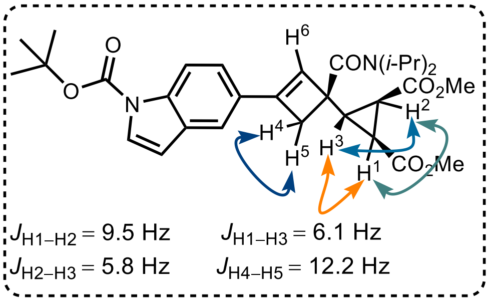

^{1}H NMR (500 MHz, $CDCl_3$) δ 8.09 (d, J = 8.6 Hz, 1H, indole), 7.58 (d, J = 3.7 Hz, 1H, ArH), 7.49 (s, 1H, ArH), 7.34 (dd, J = 8.6, 1.7 Hz, 1H, ArH), 6.54 (d, J = 3.7 Hz, 1H, indole), 6.18 (s, 1H, H6), 4.06 (septet, J = 6.6 Hz, 1H, *iso*-propyl), 3.68 (s, 3H, CO_2Me), 3.66 (s, 3H, CO_2Me), 3.36 (septet, J = 6.7 Hz, 1H, *iso*-propyl), 3.22 (d, J = 12.2 Hz, 1H, H4), 2.88 (d, J = 12.2 Hz, 1H, H5), 2.55 (t, J = 6.1 Hz, 1H, H3), 2.22 (t, J = 5.8 Hz, 2H, H1 and H2), 1.67 (s, 9H, *t*-butyl), 1.42

(d, J = 6.5 Hz, 6H, *iso*-propyl), 1.34 (d, J = 6.5 Hz, 3H, *iso*-propyl), 1.29 (d, J = 6.5 Hz, 3H, *iso*-propyl); ^{13}C NMR (126 MHz, $CDCl_3$) δ 171.7, 170.2, 169.9, 149.7, 146.9, 135.3, 130.6, 128.2, 126.8, 123.5, 121.6, 117.8, 115.2, 107.5, 84.1, 52.4, 52.3, 50.0, 49.1, 46.3, 38.8, 30.1, 28.3, 26, 24.2, 20.9, 20.9, 20.6, 20.3; IR $υ_{max}$ (cm^{-1}): 2976, 1734, 1620 ,1440, 1370, 1289, 1214, 1134, 1024; HRMS (ESI+) $[M+H]^+$ calculated for $[C_{31}H_{41}N_2O7]^+$: 553.2908, found: 551.2897.

Synthesis and characterization of **3x:**

Synthesized in accordance with *General reaction procedure c* using BCB-**1ae** (23 mg, 0.10 mmol, 1 equiv.) and diazo ester **2a** (37 mg, 0.20 mmol, 2 equiv.). All volatiles were removed in a vacuum and the crude compound was purified *via* column chromatography using silica gel and pentane/diethyl ether (1:1 v/v; R_f = 0.5). The desired product **3x** was obtained as a yellowish liquid. Yield: 28 mg, 0.07 mmol, 73%.

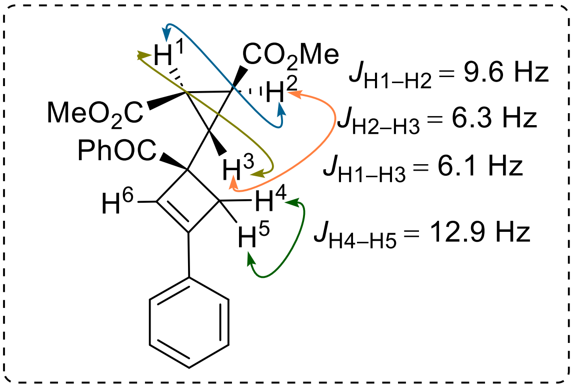

^{1}H NMR (500 MHz, $CDCl_3$, 298 K) δ 7.99 (d, J = 6.9 Hz, 2H, ArH), 7.59–7.55 (m, 1H, ArH), 7.49 (dd, J = 8.3, 7.0 Hz, 2H, ArH), 7.36–7.28 (m, 5H, ArH), 6.35 (s, 1H, H6), 3.68 (s, 3H, CO_2Me), 3.60 (s, 3H, CO_2Me), 3.44 (d, J = 12.9 Hz, 1H, H4), 3.06 (d, J = 13.0 Hz, 1H, H5), 2.82 (t, J = 6.2 Hz, 1H, H3), 2.26 (dd, J = 9.6, 6.3 Hz, 1H, H2), 2.15 (dd, J = 9.6, 6.1 Hz, 1H, H1); ^{13}C NMR (126 MHz, $CDCl_3$, 298 K) δ 200.3, 169.6, 148.5, 135.2, 133.2, 133.0, 130.7, 129.2, 129.1, 128.81, 128.6, 128.3, 128.1, 125.1, 124.5, 61.2, 54.4, 52.5, 52.3, 39.8, 30.7, 29.8, 26.4, 24.6; IR $υ_{max}$ (cm^{-1}): 2953, 1734, 1599, 1494, 1448, 1361, 1290, 1211, 1171, 1071; HRMS (ESI+) $[M+Na]^+$ calculated for $[C_{24}H_{22}O_5Na]^+$: 413.1360, found: 413.1361.

Synthesis and characterization of **3y:**

Synthesized in accordance with *General reaction procedure c* using BCB-**1ac** (18 mg, 0.10 mmol, 1 equiv.) and diazo ester **2a** (37 mg, 0.20 mmol, 2 equiv.). All volatiles were removed in a vacuum and the crude compound was purified *via* column chromatography using silica gel and pentane/diethyl ether (1:1 v/v; R_f = 0.3). The desired product **3y** was obtained as a yellowish liquid. Yield: 31 mg, 0.09 mmol, 92%.

^{1}H NMR (700 MHz, $CDCl_3$, 298 K) δ 6.15 (s, 1H, H7), 4.32 (s, 1H, *iso*-propyl, br), 3.68 (s, 3H, CO_2Me), 3.68 (s, 3H, CO_2Me), 3.42 (s, 1H, *iso*-propyl, br), 2.97 (dd, J = 13.4, 4.4 Hz, 1H, H4), 2.72 (t, J = 5.4 Hz, 1H, H3), 2.47 (d, J = 15.4 Hz, 1H, H6), 2.23 (q, J = 6.4 Hz, 1H, H5), 1.98 (dd, J = 9.4, 6.3 Hz, 1H, H2), 1.96 (dd, J = 9.5, 6.1 Hz, 1H, H1), 1.42 (s, 6H, *iso*-propyl, br), 1.20 (s, 6H, *iso*-propyl, br); ^{13}C NMR (176 MHz, $CDCl_3$, 298 K) δ 170.0, 169.99, 163.6, 143.7, 136.7, 52.3, 49.31, 46.2, 39.1, 36.5, 27.9, 26.5, 25.8, 21.2, 20.7; IR υ_{max} (cm^{-1}): 2970, 1736, 1620, 1440, 1370 ,1343, 1291, 1249, 1210, 1039; HRMS (ESI+) $[M+H]^+$ calculated for $[C_{18}H_{28}NO_5]^+$: 338.1962, found: 338.1958.

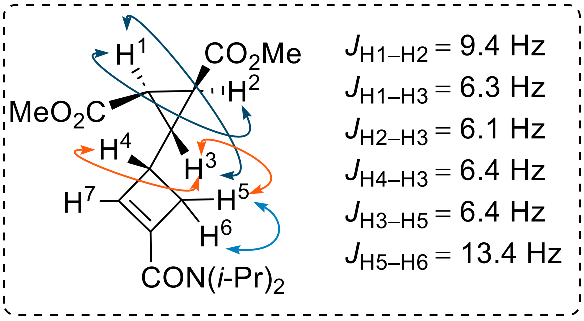

Synthesis and characterization of **3z (major isomer):**

Synthesized in accordance with *General reaction procedure c* using BCB-**1ad** (20 mg, 0.10 mmol, 1 equiv.) and diazo ester **2a** (37 mg, 0.20 mmol, 2 equiv.). All volatiles were removed in a vacuum and the crude compound was purified *via* column chromatography using silica gel and pentane/diethyl ether (1:1 v/v; R_f = 0.3). The desired product **3z** was obtained as a colorless liquid. Yield: 18 mg, 0.05 mmol, 52%.

^{1}H NMR (500 MHz, $CDCl_3$, 298 K) δ 5.65 (q, J = 1.5 Hz, 1H, H6), 3.90 (septet, J = 6.7 Hz, 1H, *iso*-propyl), 3.69 (s, 3H, CO_2Me), 3.65 (s, 3H, CO_2Me), 3.32 (septet, J = 6.7 Hz, 1H, *iso*-propyl), 2.78 (dd, J = 12.5, 1.3 Hz, 1H, H4), 2.44 (dd, J = 12.8, 1.4 Hz, 1H, H5), 2.42 (t, J = 6.2 Hz, 2H, H3), 2.13 (dd, J = 9.6, 6.2 Hz, 1H, H2), 2.10 (dd, J = 9.6, 6.2 Hz, 1H, H1), 1.71 (d, J = 1.4 Hz, 3H, Me), 1.42 (d, J = 6.8 Hz, 3H, *iso*-propyl), 1.37 (d, J = 6.7 Hz, 3H, *iso*-propyl), 1.28 (d, J = 6.7 Hz, 3H, *iso*-propyl), 1.21 (d, J = 6.5 Hz, 3H, *iso*-propyl); ^{13}C NMR (126 MHz, $CDCl_3$, 298 K) δ 172.1, 170.33, 169.9, 147.5, 127.4, 52.4, 52.2, 50.5, 48.9, 46.2, 42.3, 29.8, 25.9, 24.1, 20.9, 20.8, 20.5, 20.2, 16.7; IR υ_{max} (cm^{-1}): 2965, 1744, 1628, 1439, 1370, 1299, 1269, 1137, 1098, 1031; HRMS (ESI+) $[M+Na]^+$ calculated for $[C_{19}H_{29}NO_5Na]^+$: 374.1938, found: 374.1946.

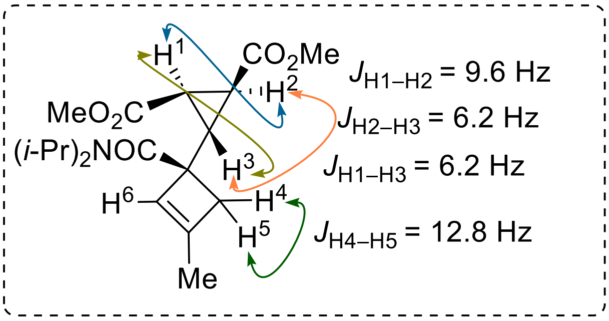

Characterization of **3z'** (3 mg, 8%, 0.008 mmol) **(minor isomer):**

^1H NMR (500 MHz, $CDCl_3$, 298 K) δ 6.08 (s, 1H, H6), 3.69 (s, 3H, CO_2Me), 3.69 (s, 3H, CO_2Me), 2.57 (d, J = 13.1 Hz, 1H, H4), 2.52 (d, J = 13.1 Hz, 1H, H5), 2.27 (t, J = 6.4 Hz, 1H, H3), 2.00 (dd, J = 9.5, 6.5 Hz, 1H, H2), 1.92 (dd, J = 9.4, 6.3 Hz, 1H, H1), 1.32 (s, 3H, Me); ^{13}C NMR (126 MHz, $CDCl_3$, 298 K) δ 170.3, 170. 1, 147.9, 142.2, 140.2, 137.9, 52.4, 42.9, 42.2, 32.9, 29.9, 25.6, 25.2, 23.5, 21.2, 20.7; IR υ_{max} (cm^{-1}): 2955, 2927, 1743, 1620, 1440, 1370, 1341, 1303, 1280, 1258, 1212, 1167, 1040; HRMS (ESI+) $[M+Na]^+$ calculated for $[C_{19}H_{29}NO_5Na]^+$: 374.1938, found: 374.1953.

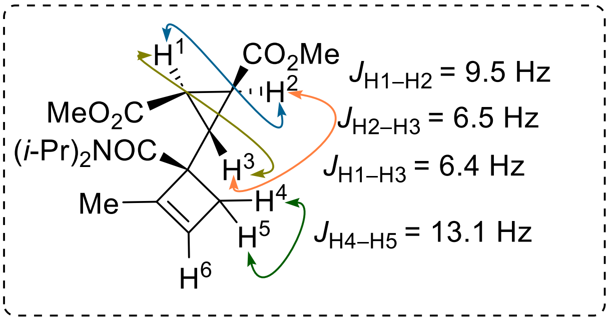

Synthesis and characterization of **3aa:**

Synthesized in accordance with *General reaction procedure c* using BCB-**1ag** (27 mg, 0.10 mmol, 1 equiv.) and diazo ester **2a** (37 mg, 0.20 mmol, 2 equiv.). All volatiles were removed in a vacuum and the crude compound was purified *via* column chromatography using silica gel and pentane/diethyl ether (1:1 v/v; R_f = 0.5). The desired product **3aa** was obtained as a colorless liquid. Yield: 18 mg, 0.04 mmol, 41%.

^1H NMR (500 MHz, $CDCl_3$, 298 K) δ 7.37–7.31 (m, 4H, ArH), 7.25–7.23 (m, 1H, ArH), 3.99 (septet, J = 6.6 Hz, 1H, *iso*-propyl), 3.69 (s, 3H, CO_2Me), 3.68 (s, 3H, CO_2Me), 3.34 (septet, J = 6.8 Hz, 1H, *iso*-propyl), 2.98 (dd, J = 11.8, 2.2 Hz, 1H, H4), 2.68 (dd, J = 11.8, 2.1 Hz, 1H, H5), 2.55 (t, J = 6.4 Hz, 1H, H3), 2.29 (dd, J = 9.6, 6.6 Hz, 1H, H2), 2.25 (dd, J = 9.5, 6.1 Hz, 1H, H1), 2.17 (t, J = 2.0 Hz, 3H, Me), 1.42 (d, J = 2.3 Hz, 3H, *iso*-propyl), 1.41 (d, J = 2.3 Hz, 3H, *iso*-propyl), 1.30 (d, J = 6.6 Hz, 3H, *iso*-propyl), 1.26 (d, J = 3.2 Hz, 3H, *iso*-propyl); ^{13}C NMR (126 MHz, $CDCl_3$, 298 K) δ 172.3, 170.0, 169.8, 140.1, 136.9, 134.7, 128.5, 127.6, 126.2, 52.4, 52.3, 51.1, 48.4, 46.2, 37.0, 30.5, 29.8, 25.9, 25.6, 21.0, 20.6, 20.3, 15.3; IR υ_{max} (cm^{-1}): 2918, 1740, 1626, 1494, 1439, 1369, 1327, 1295, 1249, 1214, 1166, 1034; HRMS (ESI+) $[M+Na]^+$ calculated for $[C_{25}H_{33}NO_5Na]^+$: 450.2251, found: 450.2252.

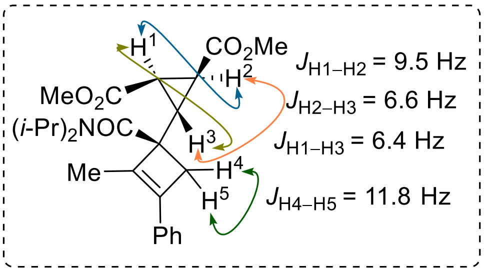

Synthesis and characterization of **3ab** and **3ab'**:

Synthesized in accordance with *General reaction procedure c* using BCB-**1a** (19 mg, 0.10 mmol, 1 equiv.) and diazo ester **2h** (56 mg, 0.20 mmol, 2 equiv.). All volatiles were removed in a vacuum and the crude compound was purified via column chromatography using silica gel and pentane/diethyl ether (3:2 v/v; R_f = 0.3). Two regio-isomers **3ab** and **3ab'** were obtained as the final products.

Yield: **3ab** - 23 mg (colorless solid), 0.05 mmol, 51% and **3ab'** (colourless oil), 15 mg, 0.04 mmol, 35%.

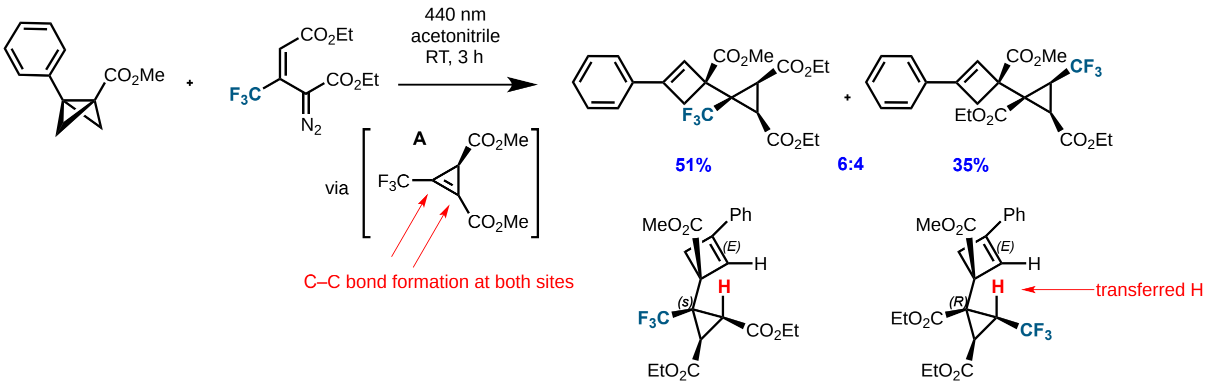

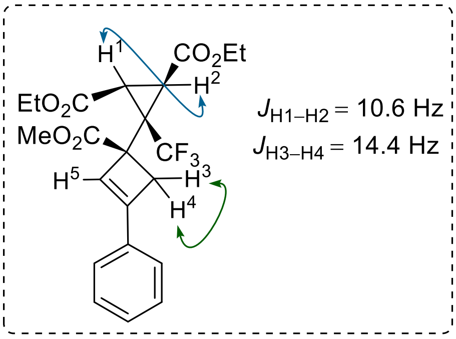

^{1}H NMR (500 MHz, $CDCl_3$) δ 7.38–7.37 (m, 5H, ArH), 5.92 (s, 1H, H5), 4.28–4.11 (m, 4H, CO_2**CH_2**CH_3), 3.77 (s, 3H, CO_2Me), 3.64 (d, *J* = 14.4 Hz, 1H, H4), 2.93 (d, *J* = 13.9 Hz, 1H, H3), 2.53 (d, *J* = 10.6 Hz, 1H, H1), 2.50 (d, *J* = 10.6 Hz, 1H, H2), 1.31 (t, *J* = 7.1 Hz, 3H, CO_2CH_2**CH_3**), 1.25 (t, *J* = 7.1 Hz, 3H, CO_2CH_2**CH_3**); ^{13}C NMR (126 MHz, $CDCl_3$) δ 171.8, 165.6, 165.4, 154.2, 132.4, 129.9, 128.8, 125.5, 121.5, 61.8, 61.7, 52.7, 51.9, 36.7, 28.2, 24.9, 14.1, 14.0; ^{19}F NMR (377 MHz, CDCl3) δ –58.39 (CF_3); IR υ_{max} (cm-1): 2987, 2848, 1746, 1646, 1525, 1491, 1449, 1397, 1259, 1122, 1028; HRMS (ESI+) $[M+H]^+$ calculated for $[C_{22}H_{24}F_3O_6]^+$: 441.1520, found: 441.1511.

^{1}H NMR (600 MHz, $CDCl_3$) δ 7.39–7.36 (m, 5H, ArH), 5.97 (s, 1H, H5), 4.27–4.19 (m, 4H, CO_2**CH_2**CH_3), 3.74 (s, 3H, CO_2Me), 3.50 (d, *J* = 13.7 Hz, 1H, H3), 2.91 (d, *J* = 13.8 Hz, 1H,H4), 2.37 (d, *J* = 9.8 Hz, 1H, H2), 2.12 (dq, *J* = 9.9, 8.3 Hz, 1H ,H1), 1.31 (t, *J* = 7.2 Hz, 3H, CO_2CH_2**CH_3**), 1.26 (t, *J* = 7.1 Hz, 3H, CO_2CH_2**CH_3**), ^{13}C NMR (151 MHz, $CDCl_3$) δ 172.4, 166.5, 165.8, 153.9, 132.5, 129.8, 128.8, 125.5, 121.3, 62.0, 61.6, 53.3, 52.6, 37.9, 29.9, 28.3, 25.5 (q, *J* = 38.8 Hz, CF_3), 14.1, 13.9; ^{19}F NMR (565 MHz, $CDCl_3$) δ –56.89 (CF_3); IR υ_{max} (cm^{-1}): 2998, 2851,

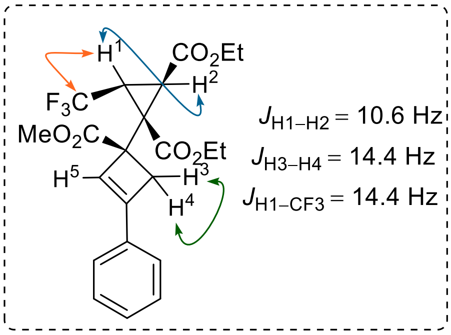

1751, 1677, 1543, 1435, 1401, 1388, 1245, 1125, 1028; HRMS (ESI+) $[M+H]^+$ calculated $[C_{22}H_{24}F_3O_6]^+$: 441.1520, found: 441.1513.

Synthesis and characterization of **D-3n:**

Synthesized in accordance with *General reaction procedure c* using BCB-**1af** (26 mg, 0.10 mmol, 1 equiv.) and diazo ester **2a** (37 mg, 0.20 mmol, 2 equiv.), All volatiles were removed in a vacuum and the crude compound was purified *via* column chromatography using silica gel and pentane/diethyl ether (1:1 v/v; R_f = 0.4). The desired product **D-3n** was obtained as a white solid. Yield: 36 mg, 0.09 mmol, 88%.

^{1}H NMR (500 MHz, $CDCl_3$, 298 K) δ 7.34–7.32 (m, 4H, ArH), 7.31–7.28 (m, 1H, ArH), 6.22 (s, 1H, H6), 4.03 (septet, *J* = 6.4 Hz, 1H, *iso*-propyl), 3.67 (s, 3H, CO_2Me), 3.66 (s, 3H, CO_2Me), 3.35 (septet, *J* = 6.6 Hz, 1H, *iso*-propyl), 3.17 (dd, *J* = 12.3, 1.7 Hz, 1H, H4), 2.83 (d, *J* = 13.2 Hz, 1H, H5), 2.53 (t, *J* = 6.2 Hz, 1H, H3), 2.22 (dd, *J* = 9.6, 6.3, 1H, H2), 2.18 (dd, *J* = 9.6, 6.0 Hz, 1H, H1), 1.41 (d, *J* = 7.1 Hz, 6H, *iso*-propyl), 1.33 (d, *J* = 6.6 Hz, 3H, *iso*-propyl), 1.28 (d, *J* = 6.5 Hz, 3H, *iso*-propyl); ^{13}C NMR (126 MHz, $CDCl_3$, 298 K) δ171.48, 170.1, 169.8, 146.4, 146.3, 133.2, 133.2, 128.8, 128.5, 125.3, 125.1, 52.4, 52.3, 50.1, 50.0, 49.0, 46.3, 38.5, 30.0, 25. 9, 24.3, 20.9, 20.8, 20.6, 20.2; IR υ_{max} (cm^{-1}): 2968, 1743, 1625, 1550, 1489, 1438, 1370, 1330, 1295, 1214, 1165, 1037; HRMS (ESI+) $[M+H]^+$ calculated for $[C_{24}H_{31}DNO_5]^+$: 415.2338, found: 415.2327.

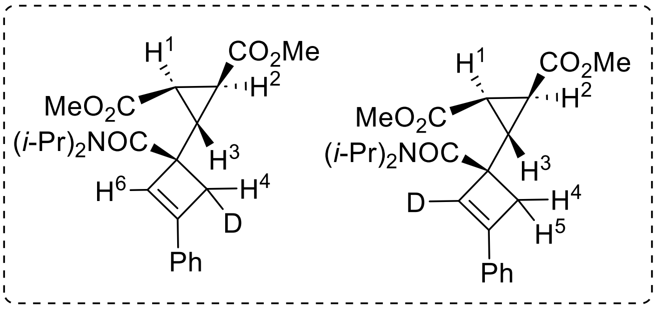

Synthesis and characterization of **7:**

A CH_2Cl_2 solution (10 mL) of **3e** (40 mg, 0.1 mmol) in a flame-dried flask was cooled to –78 °C. A stream of O_3/O_2 was flashed through a glass tube for a period of 4 to 5 min. The reaction mixture was sparged with O_2 and then N_2 for 10 min. The temperature of the reaction mixture was slowly brought to room temperature. To the crude reaction mixture, 40 µL Me_2S was added and stirred the reaction mixture at room temperature for 6 h. All the volatile was removed in a vacuum and the crude reaction mixture was purified via flash chromatography with ethyl acetate/pentane (9:1 v/v) to afford **7** as a colourless crystalline solid (32 mg, 0.08 mmol, 75%).

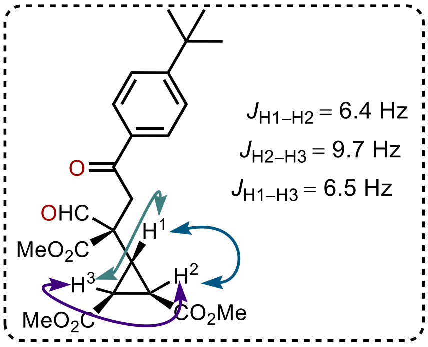

^{1}H NMR (400 MHz, C_6D_6) δ 10.35 (d, J = 1.1 Hz, 1H, CHO), 7.64 (d, J = 8.5 Hz, 2H, ArH), 7.08 (d, J = 8.5 Hz, 2H, ArH), 3.71 (d, J = 17.8 Hz, 1H, CH2), 3.62 (d, J = 17.8 Hz, 1H, CH2), 3.35 (s, 3H, CO_2Me), 3.33 (s, 3H, CO_2Me), 3.21 (s, 3H, CO_2Me), 2.69 (t, J = 6.4 Hz, 1H, H1), 2.37 (dd, J = 9.7, 6.4 Hz, 1H, H2), 2.18 (dd, J = 9.7, 6.5 Hz, 1H, H3), 1.11 (s, 9H, *t*-butyl); ^{13}C NMR (101 MHz, C_6D_6) δ 198.4, 196.1, 170.8, 168.8, 157.1, 133.4, 125.7, 56, 52.3, 52.1, 52.1, 45, 35, 31, 28.3, 25; IR $υ_{max}$ (cm^{-1}): 2956, 1728, 1672, 1604, 1541, 1397, 1317, 1265, 1071, 1023; HRMS (ESI+) $[M+H]^+$ calculated for $[C_{23}H_{29}O_8]^+$: 433.1857, found: 433.1850.

4 Competition Experiments:

To a stirred acetonitrile solution (0.50 mL) of BCBs (2.5 equiv., 0.25 mmol), 1.5 mL of an acetonitrile solution of **2a** (18 mg, 0.10 mmol, 1.0 equiv.) slowly added over 1.5 h using a syringe pump at room temperature. The reaction mixture was stirred additional 30 min at room temperature. All volatiles were removed in a vacuum. The ratios of the corresponding Alder-ene products were determined from the ^{1}H NMR spectra of the crude reaction mixture.

Competition reaction between BCBs and **2a.**

Competition Reaction: Hammett Studies

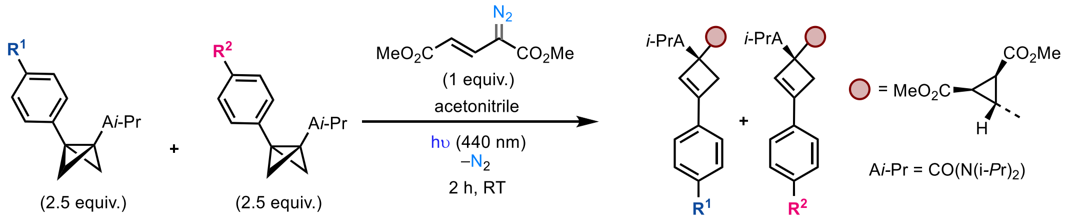

Entry	Substituents	Mole Ratio	log(k/k$_H$)
1	R^1 = CF_3 and R^2 = H	0.1:31	−2.040
2	R^1 = H and R^2 = Me	1:1	0
3	R^1 = H and R^2 = OMe	0.93:1	0.0725
4*	R^1 = Cl and R^2 = H	0.24:1	−0.6197
5	R^1 = CF_3 and R^2 = Cl	0.58:1	−0.236
6	R^1 = Cl and R^2 = OMe	0.24:1	−0.6197
7	R^1 = H and R^2 = H	-	0
8	R^1 = H and R^2 = 2-naphthyl	1:0.70	−0.6197

Calculation for k_{Cl}/k_H

k_{OMe}/k_H = 1:0.93
k_{Cl}/k_{OMe} = 0.24:1
k_{Cl}/k_H = 0.24/0.93
k_{Cl}/k_H = 0.258
log(0.258) = −0.5882

k_{CF3}/k_H = 0.13:1
k_{Cl}/k_{CF3} = 1:0.58
k_{Cl}/k_H = 0.13/0.58
k_{Cl}/k_H = 0.2241
log(0.2241) = −0.649

Therefore, average of these two values were taken for the Hammett plot (logk_{Cl}/k_H = −0.6197)

*The ratio between **3n** and **3h** was determined from entries 5 and 6 as the completion reaction between these two BCBs with **2a** afforded an inseparable mixture

5 NMR Spectra (Cyclopropene part)

^{1}H NMR (500 MHz, $CDCl_3$, 298 K) spectrum of **1e**.

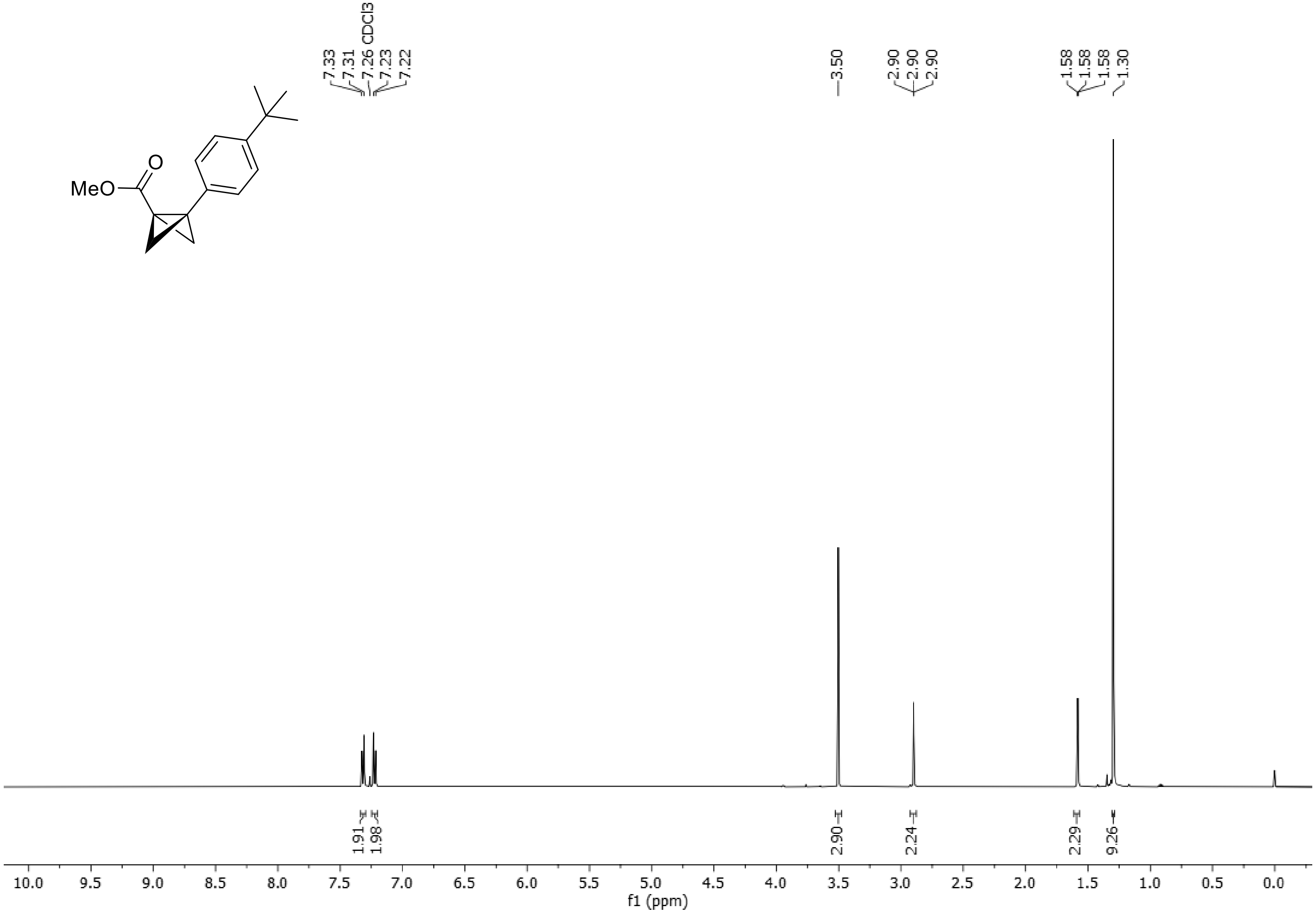

^{13}C NMR (126 MHz, $CDCl_3$, 298 K) spectrum of **1e**.

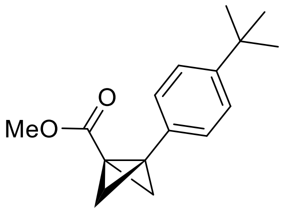

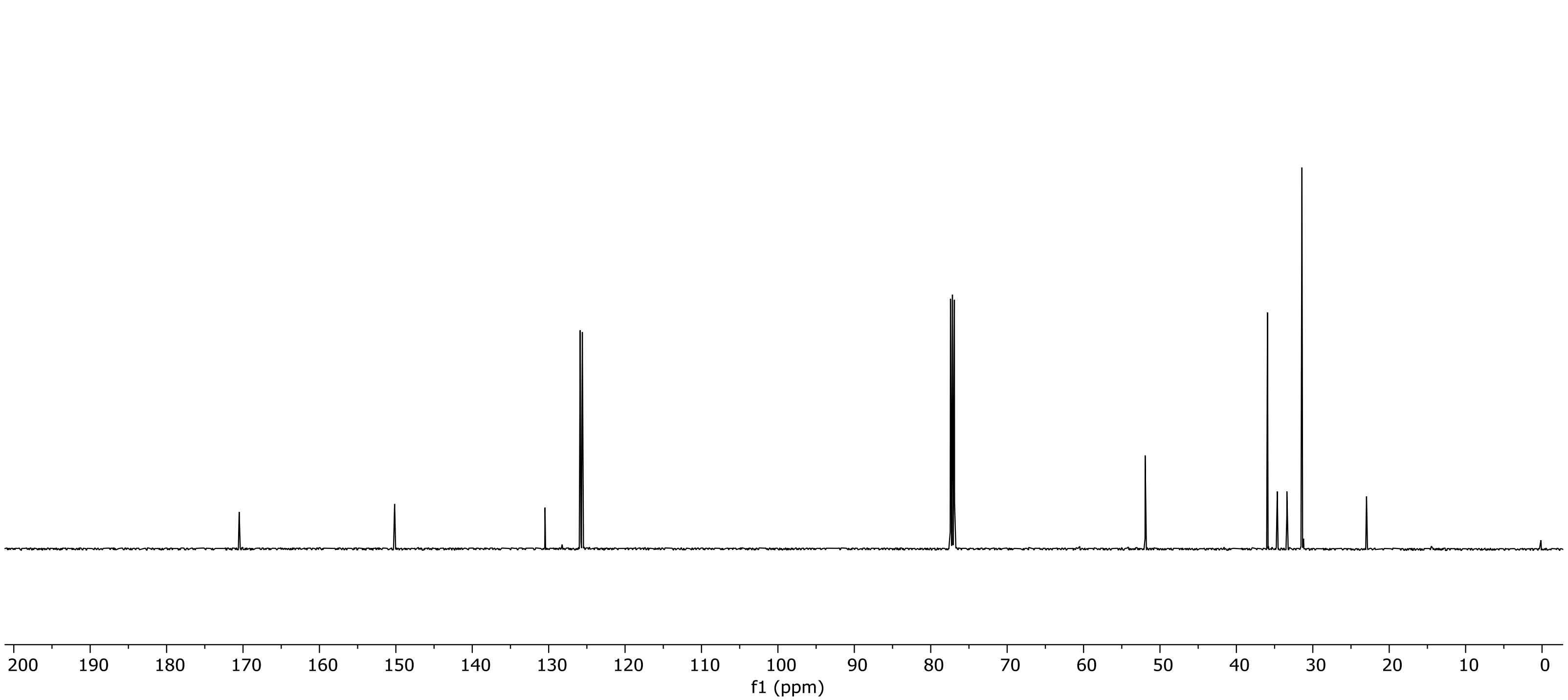

^{1}H NMR (500 MHz, $CDCl_3$, 298 K) spectrum of **1r**.

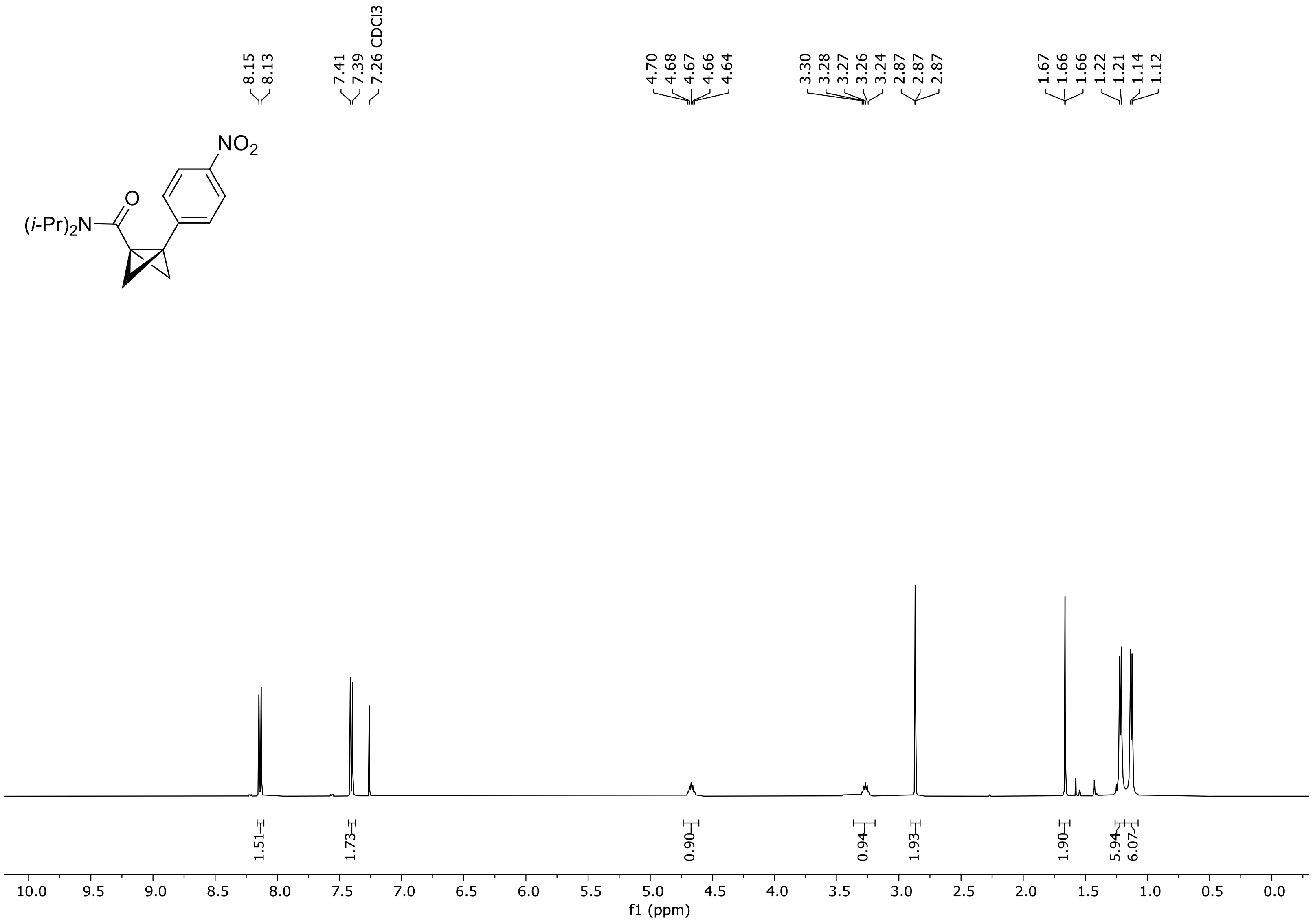

^{13}C NMR (126 MHz, $CDCl_3$, 298 K) spectrum of **1r**.

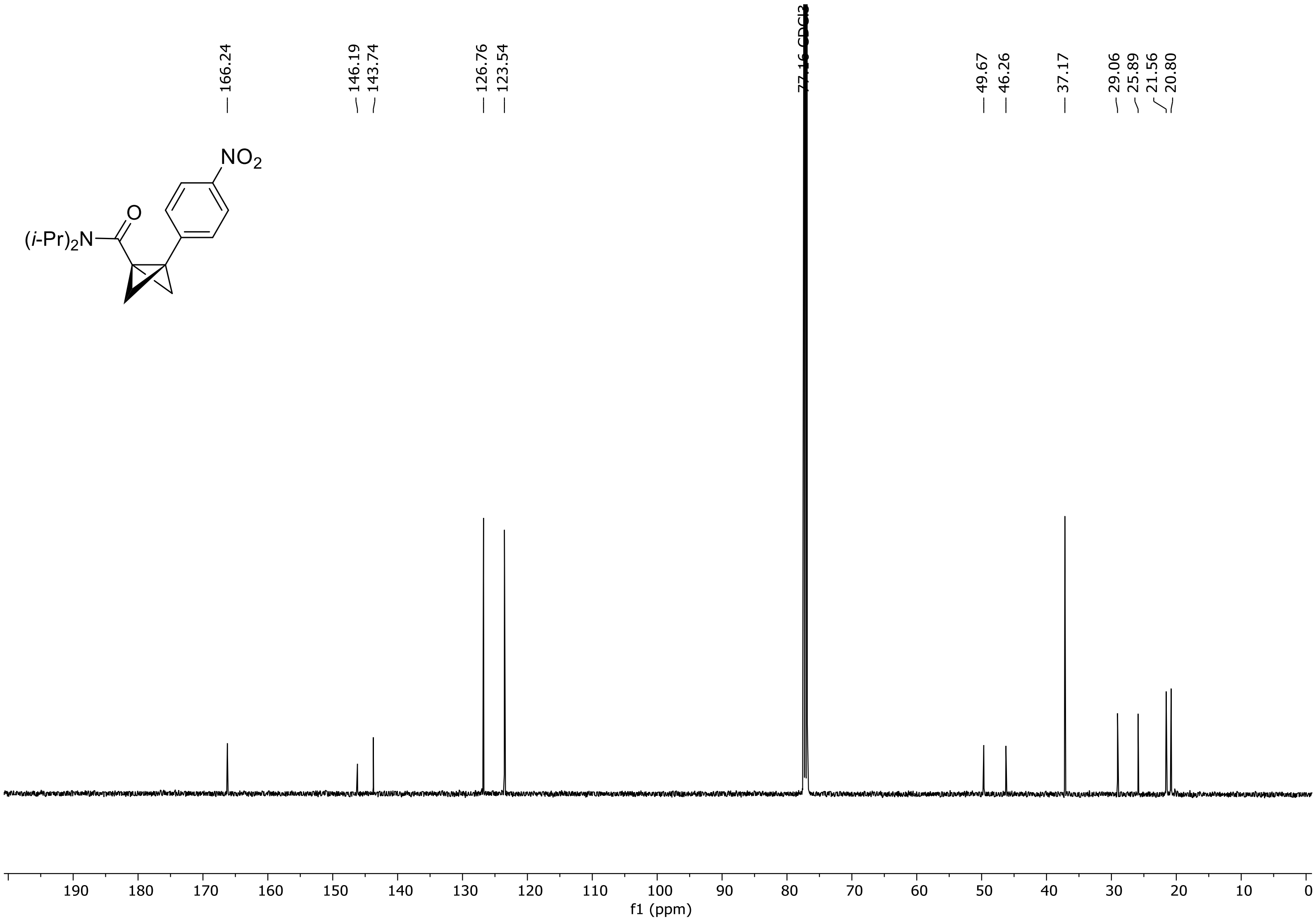

^{1}H NMR (500 MHz, $CDCl_3$, 298 K) spectrum of **1t**.

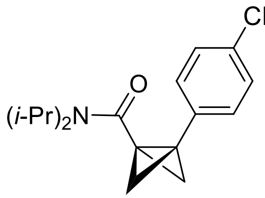

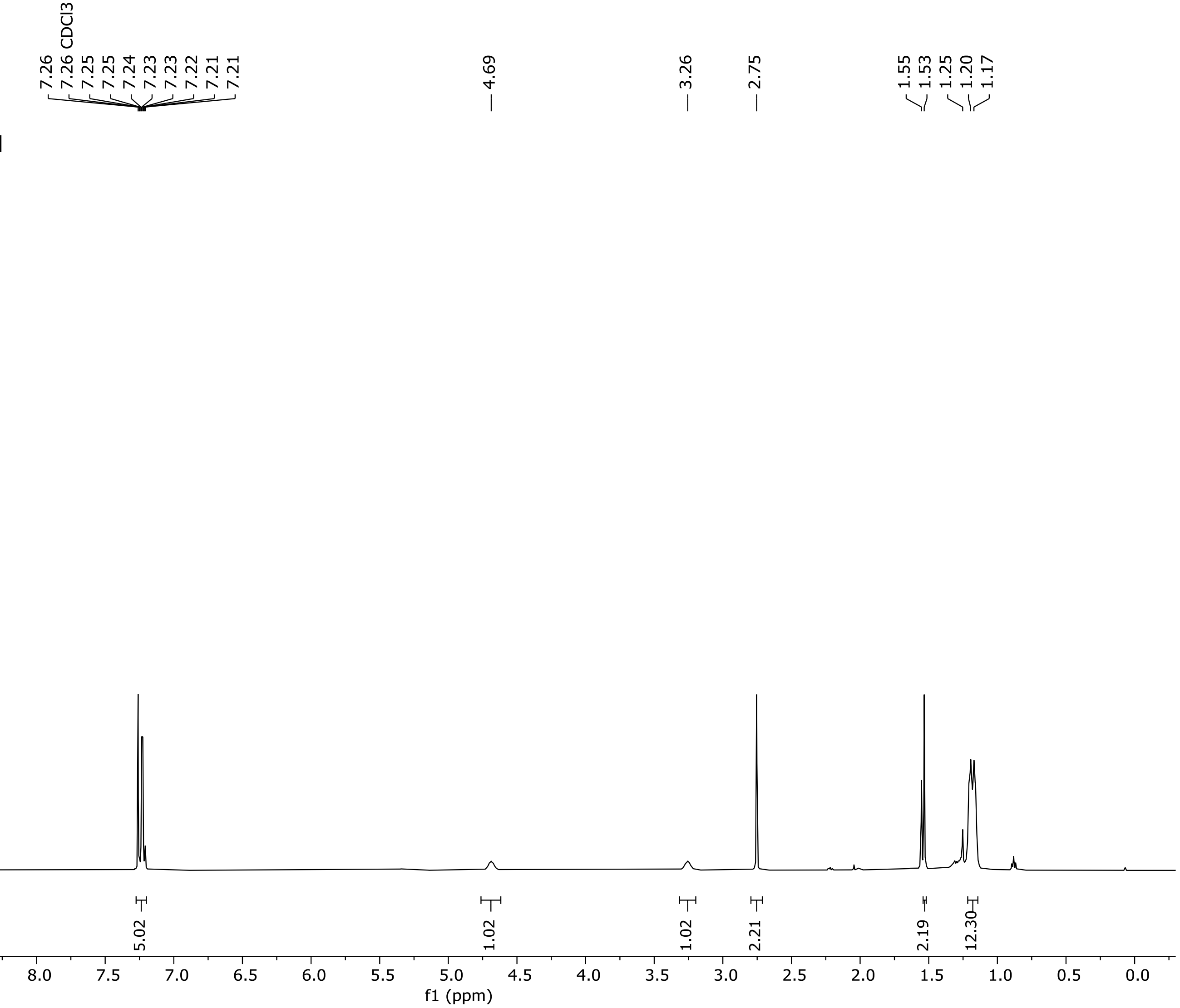

^{13}C NMR (126 MHz, $CDCl_3$, 298 K) spectrum of **1t**.

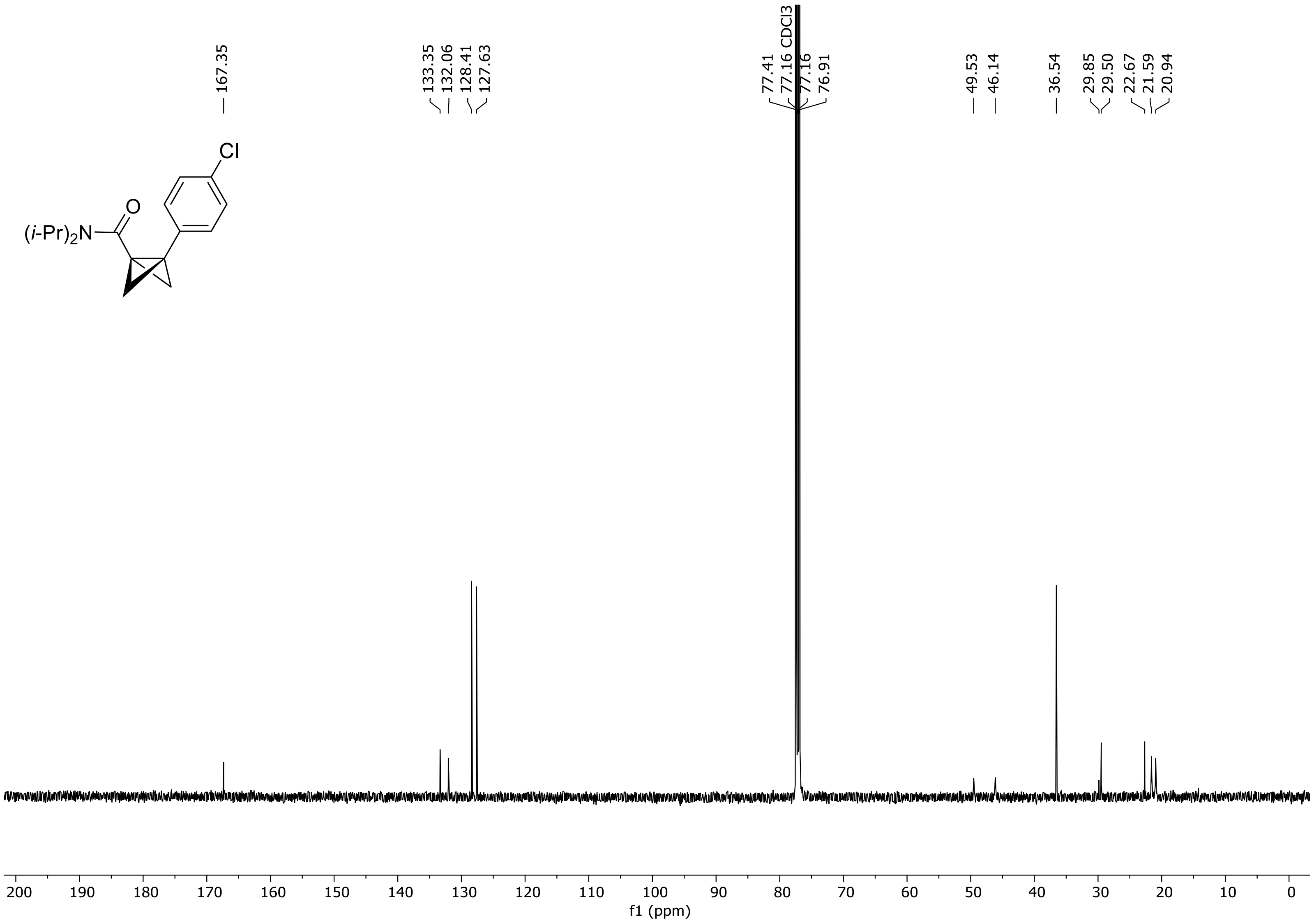

^{1}H NMR (500 MHz, $CDCl_3$, 298 K) spectrum of **1u**.

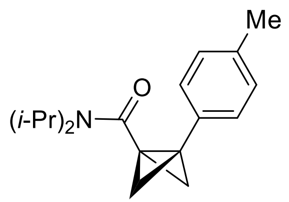

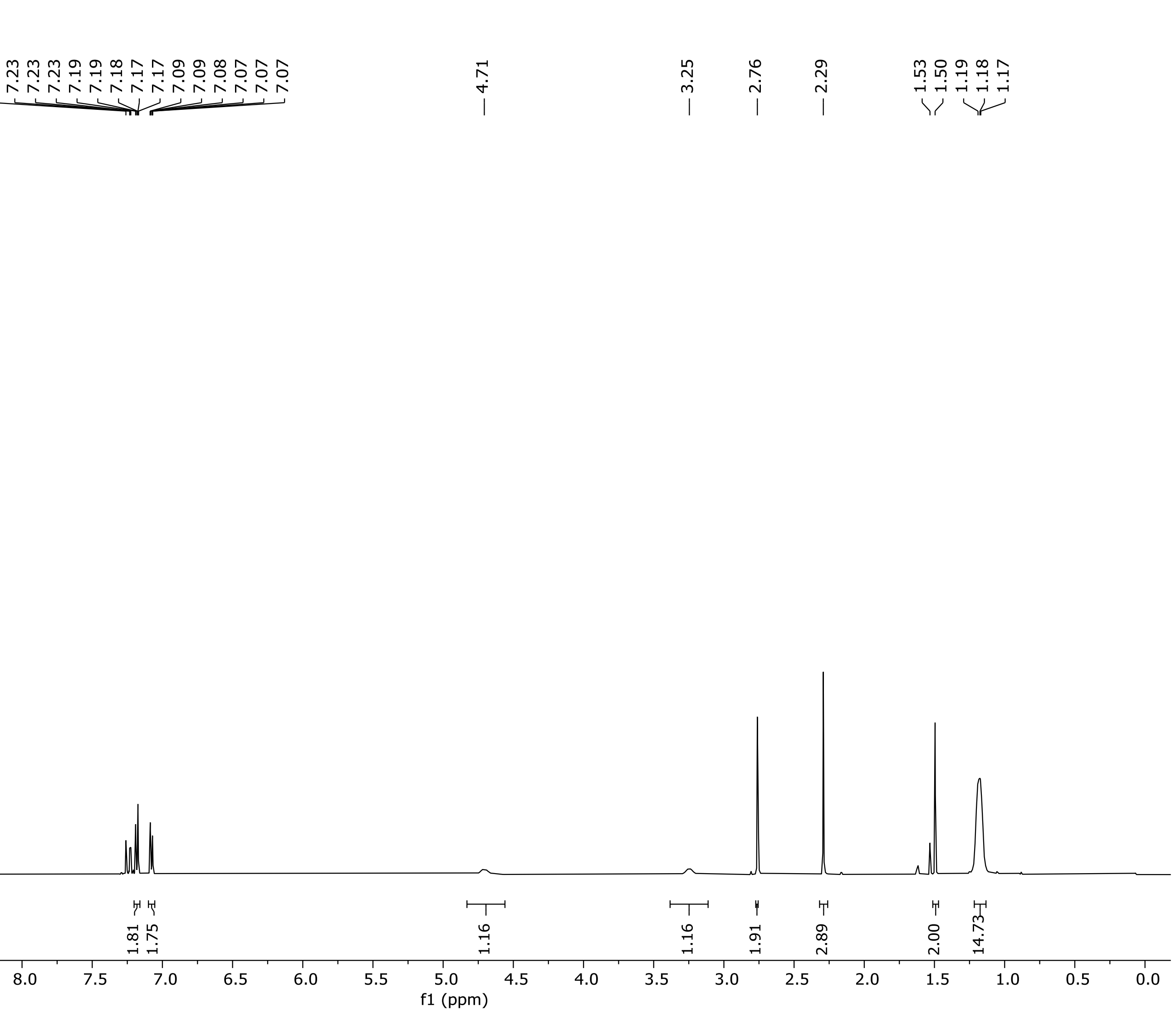

^{13}C NMR (126 MHz, $CDCl_3$, 298 K) spectrum of **1u**.

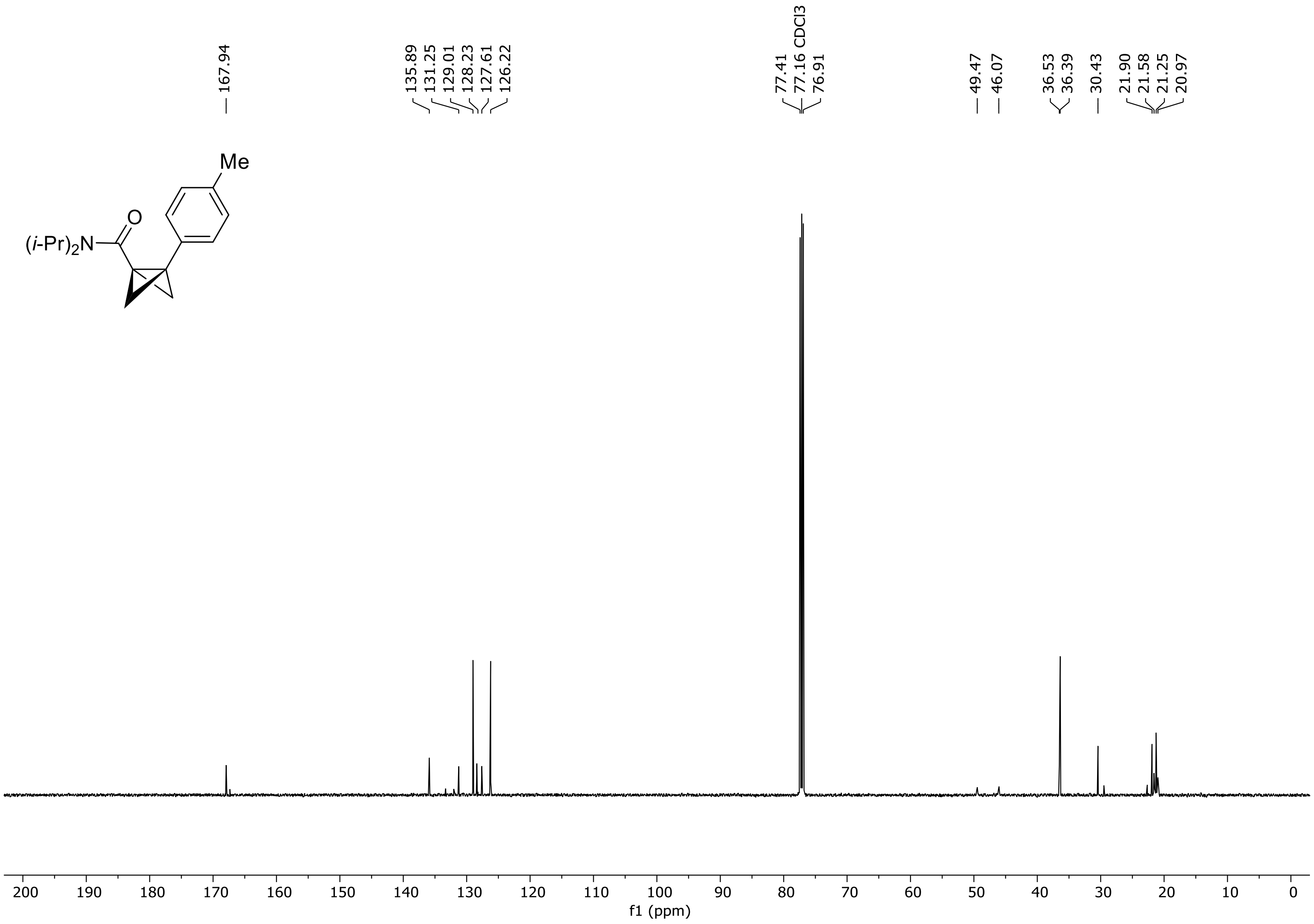

^1H NMR (500 MHz, $CDCl_3$, 298 K) spectrum of **1v**.

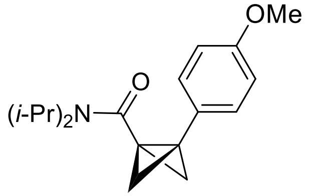

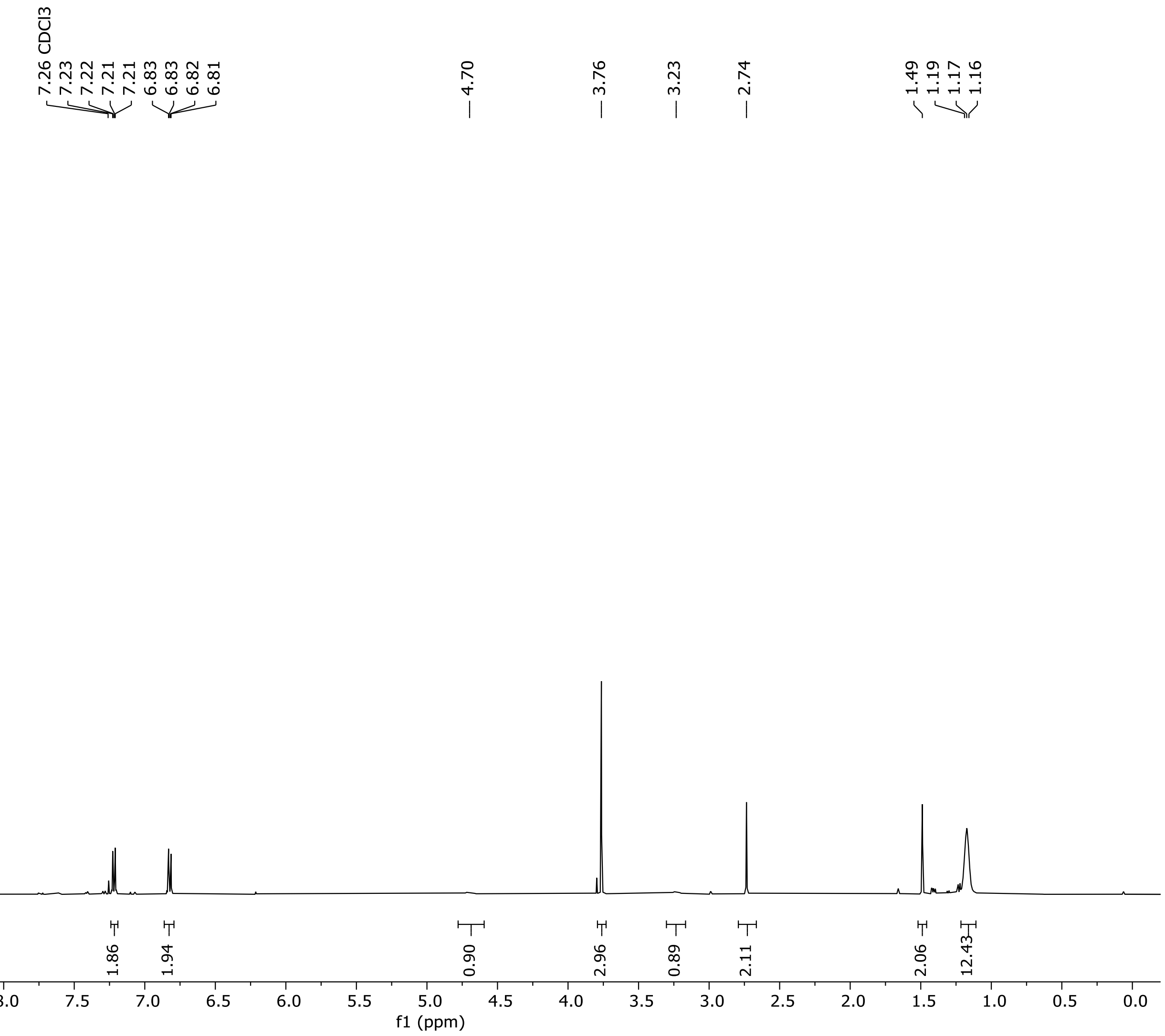

^{13}C NMR (126 MHz, $CDCl_3$, 298 K) spectrum of **1v**.

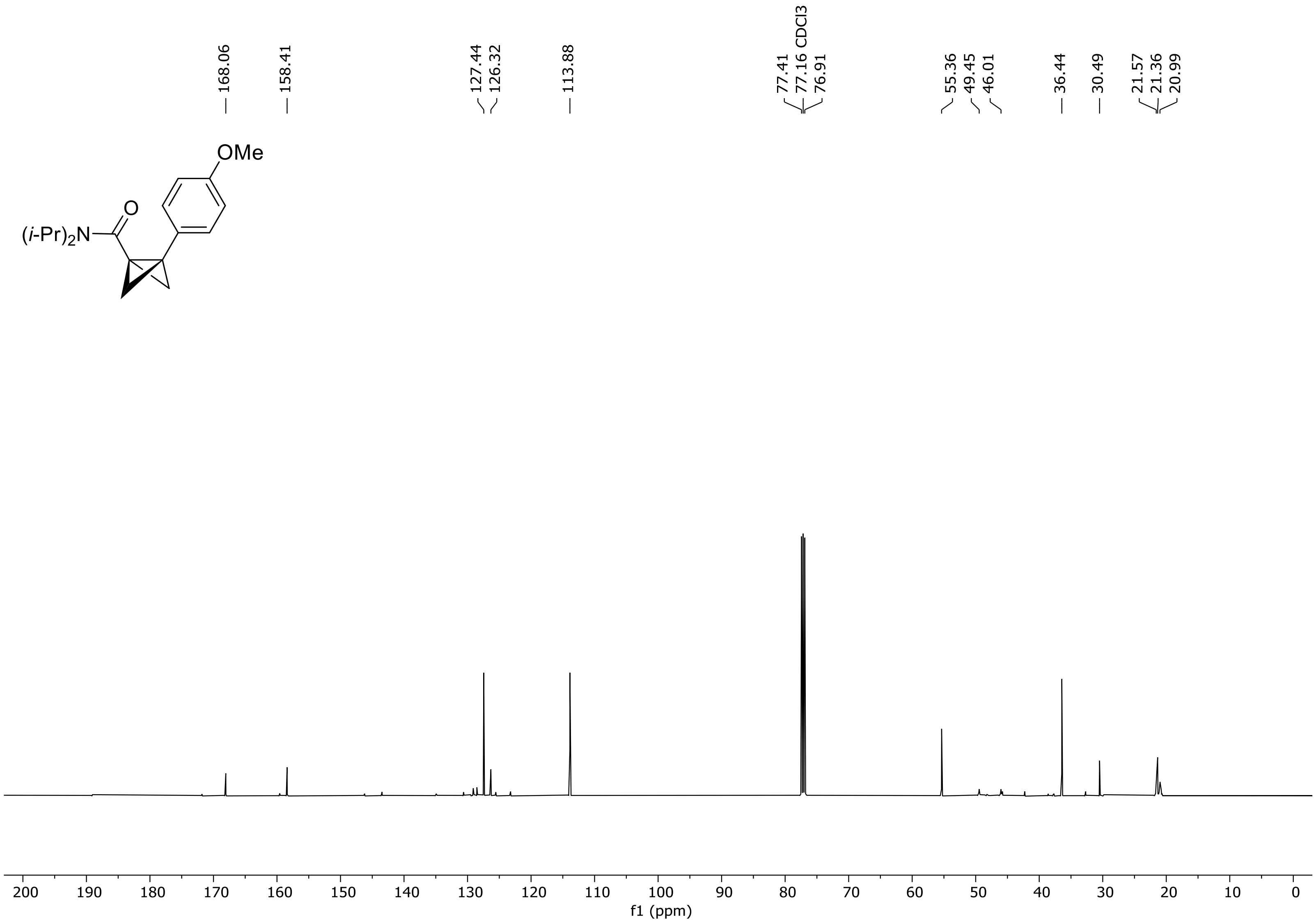

^{1}H NMR (500 MHz, $CDCl_3$, 298 K) spectrum of **1w**.

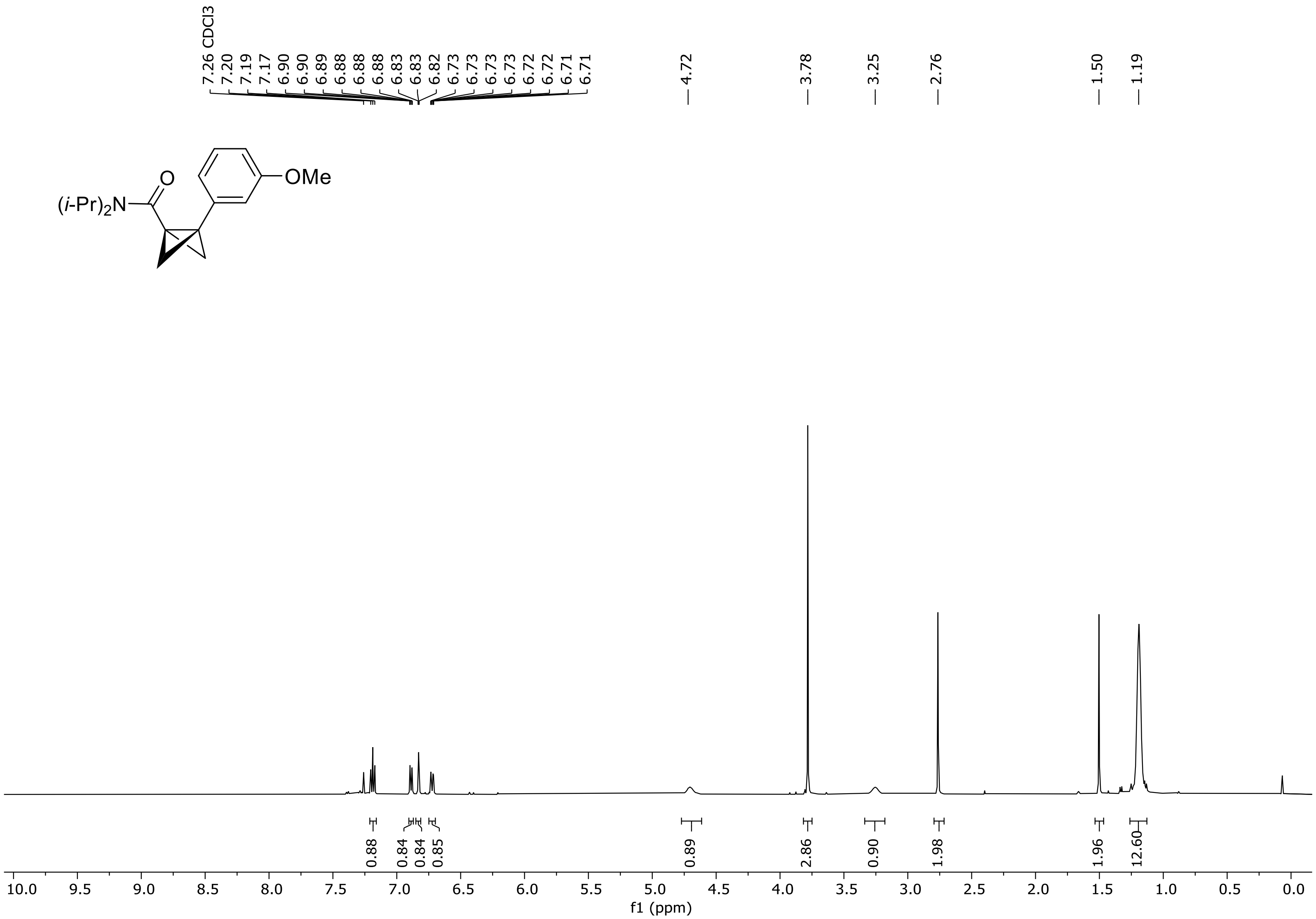

^{13}C NMR (126 MHz, $CDCl_3$, 298 K) spectrum of **1w**.

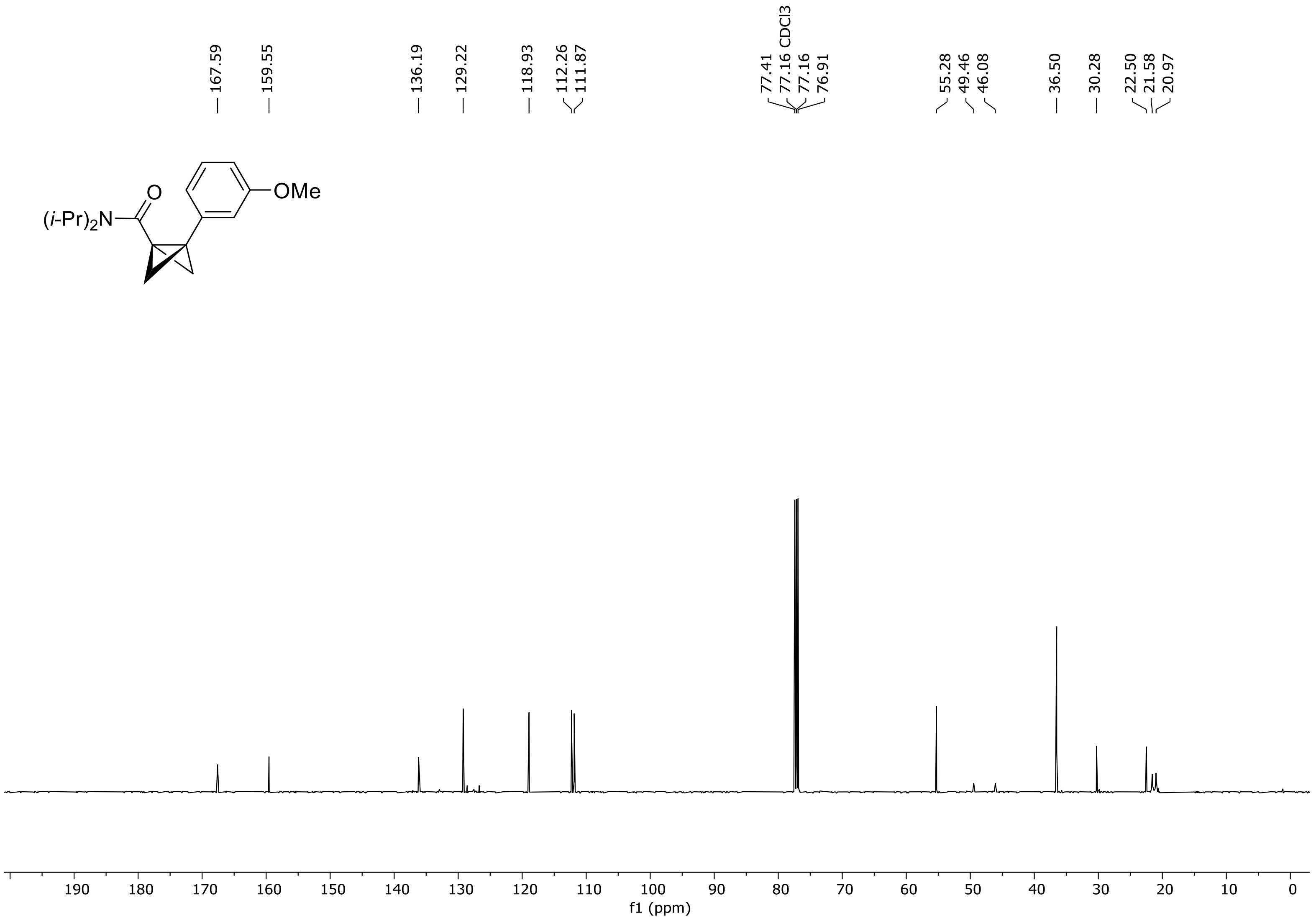

^{1}H NMR (500 MHz, $CDCl_3$, 298 K) spectrum of **1y**.

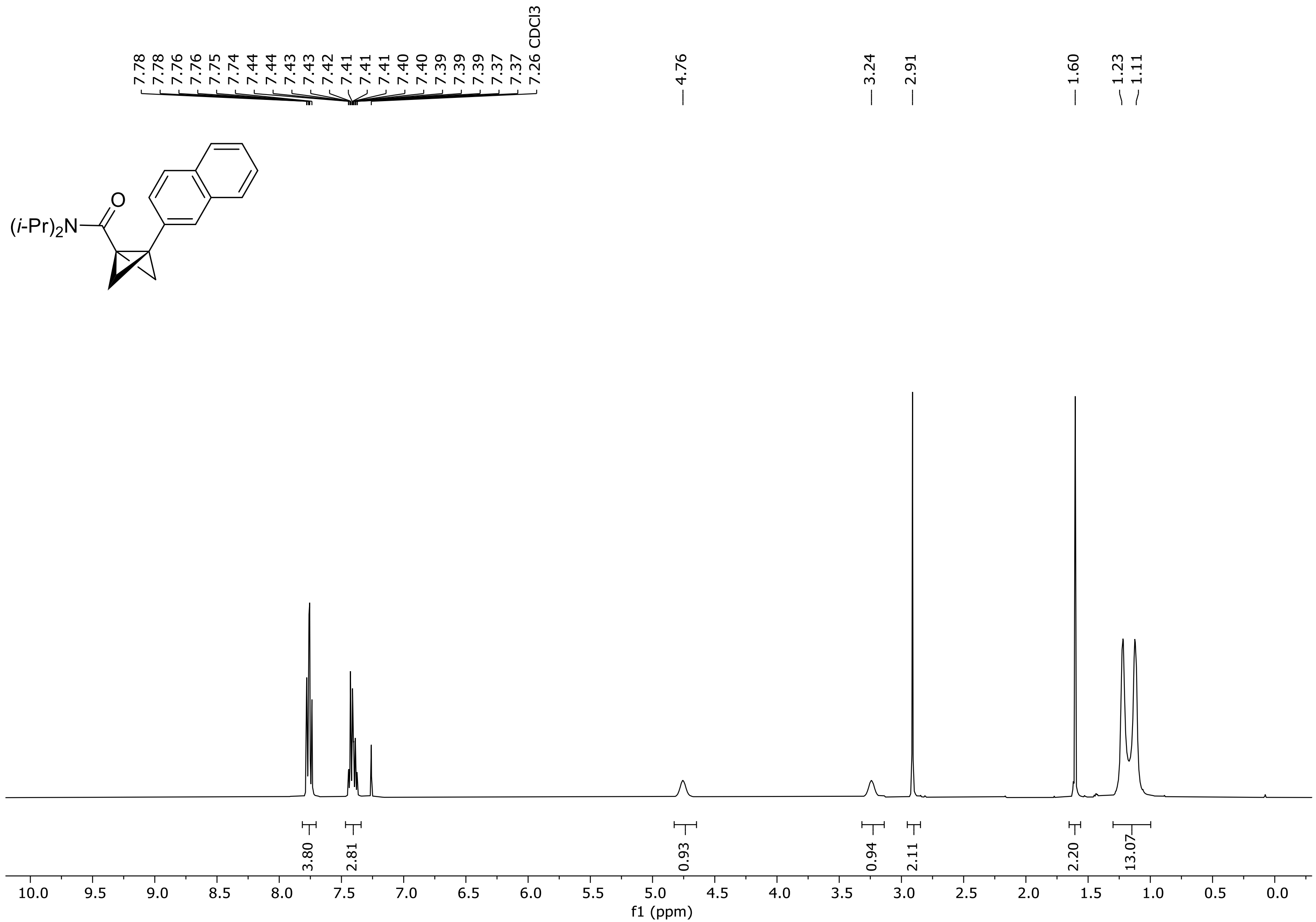

^{13}C NMR (126 MHz, $CDCl_3$, 298 K) spectrum of **1y**.

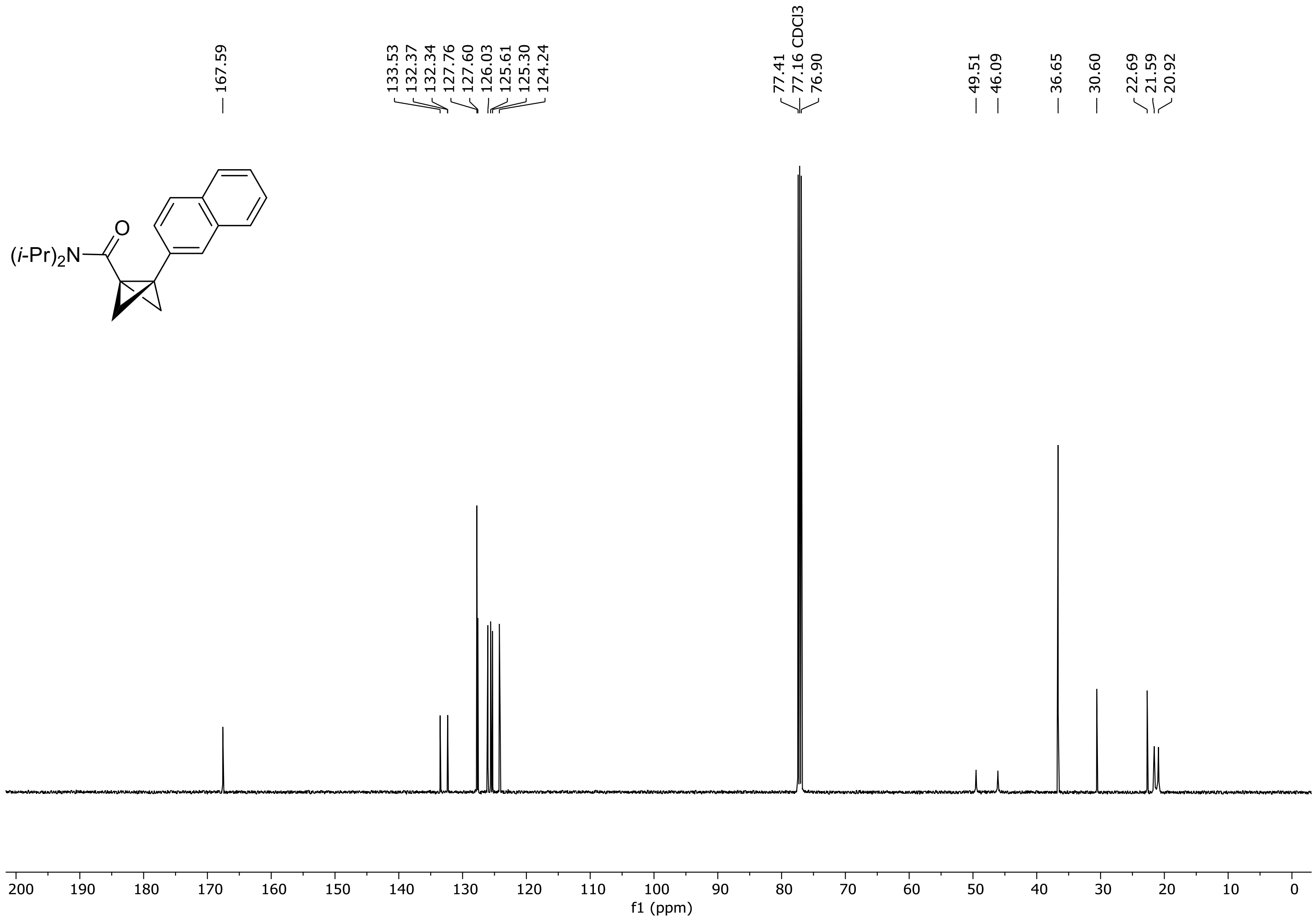

^{1}H NMR (500 MHz, C_6D_6, 298 K) spectrum of **1z**.

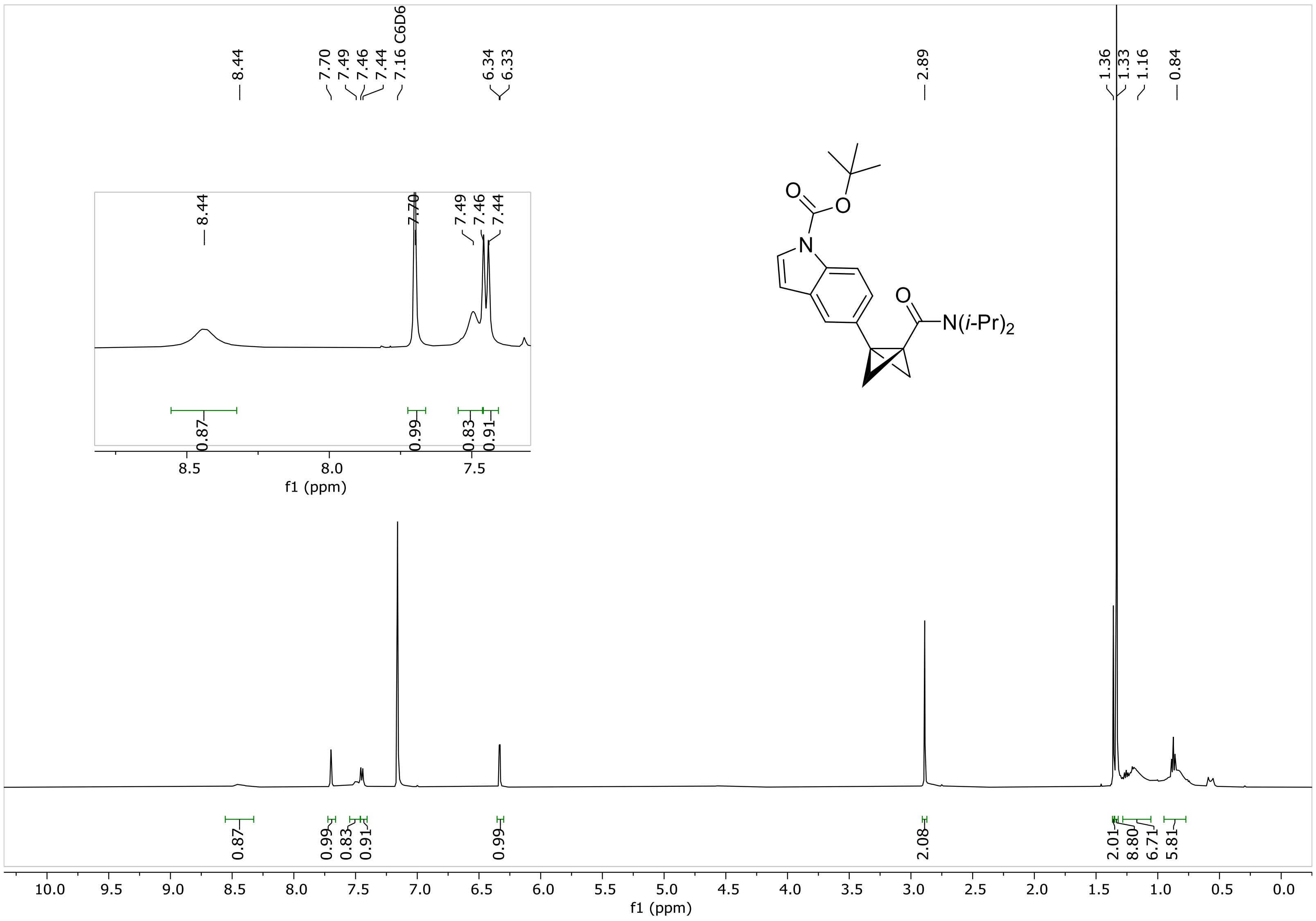

^{13}C NMR (126 MHz, C_6D_6, 298 K) spectrum of **1z**.

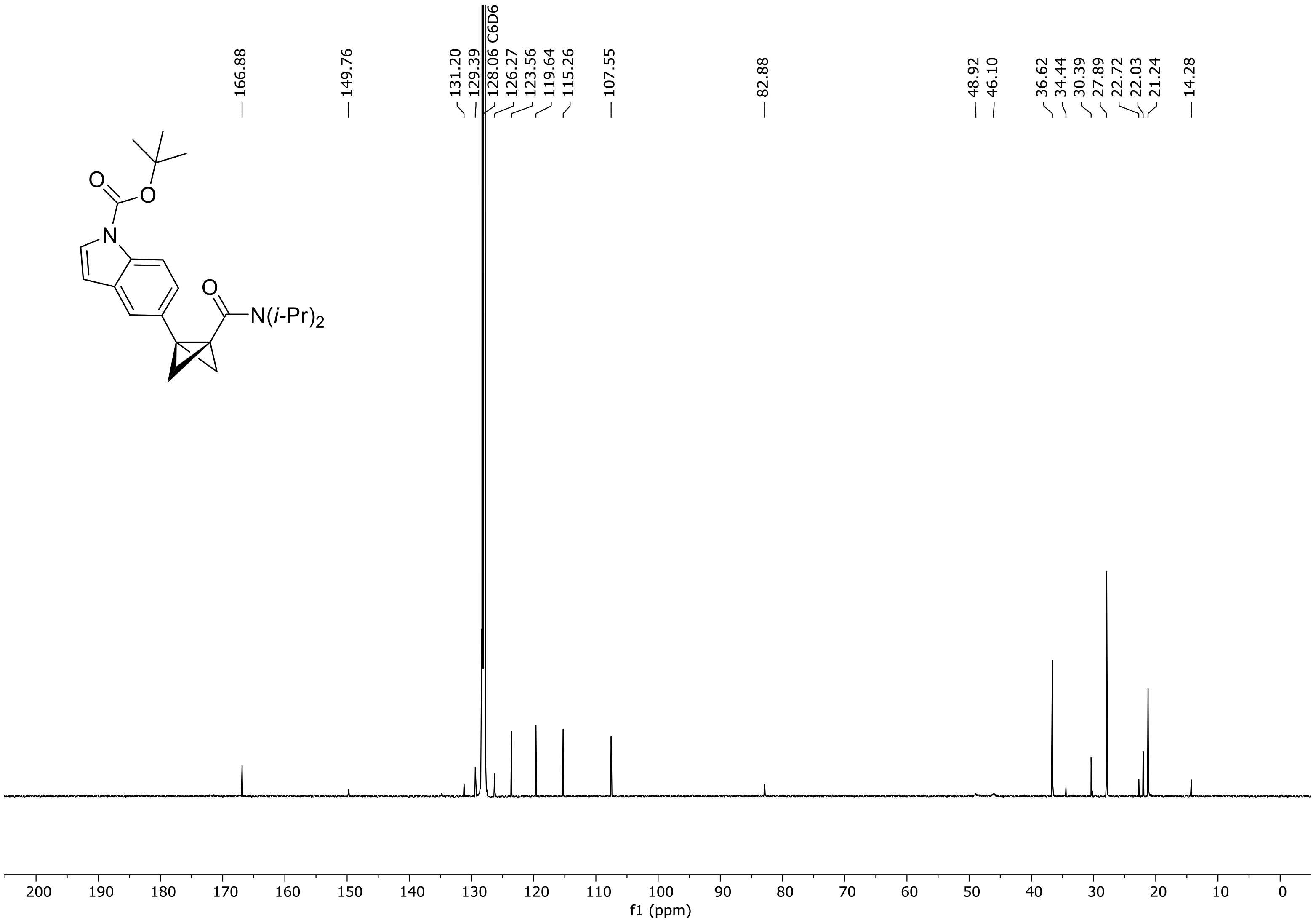

^{1}H NMR (500 MHz, $CDCl_3$, 298 K) spectrum of **1ae**.

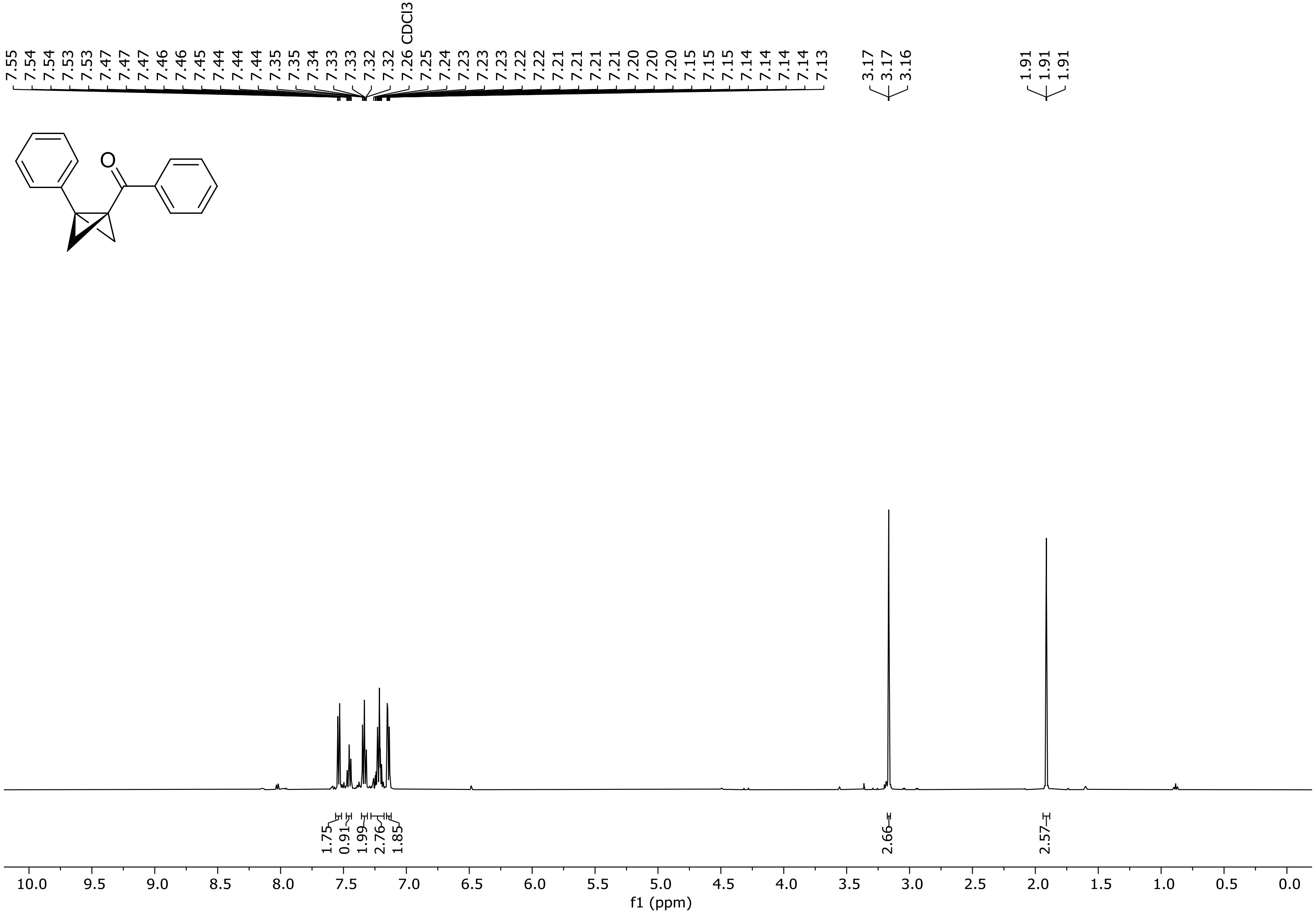

^{13}C NMR (126 MHz, $CDCl_3$, 298 K) spectrum of **1ae**.

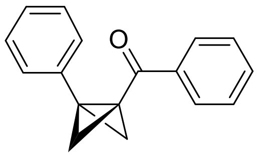

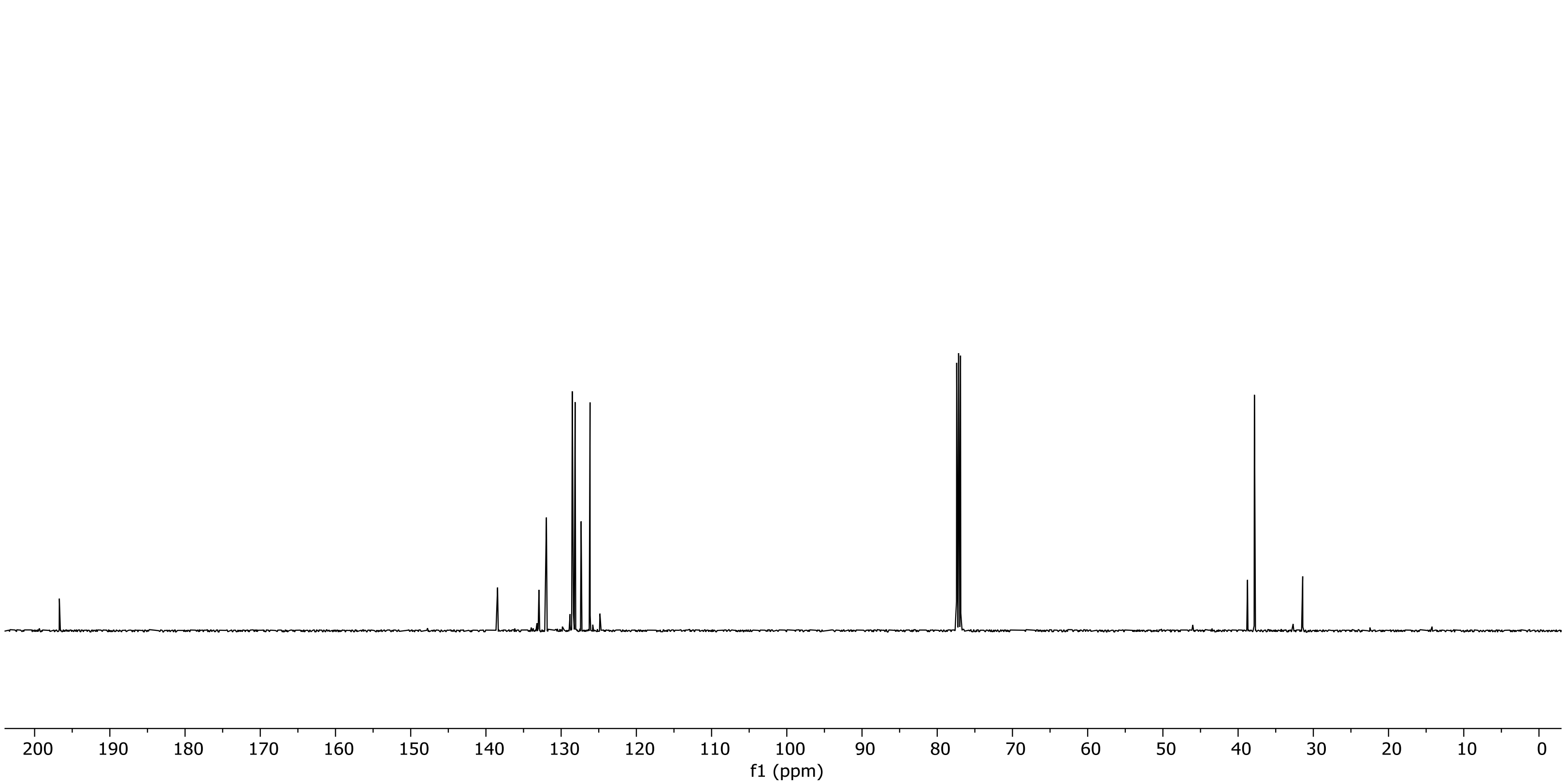

Stacked ^{1}H NMR (500 MHz, $CDCl_3$, 298 K) spectrum of 0.1 mmol (0.14 M) **2a** irradiated by 400 nm light for 1.5 h.

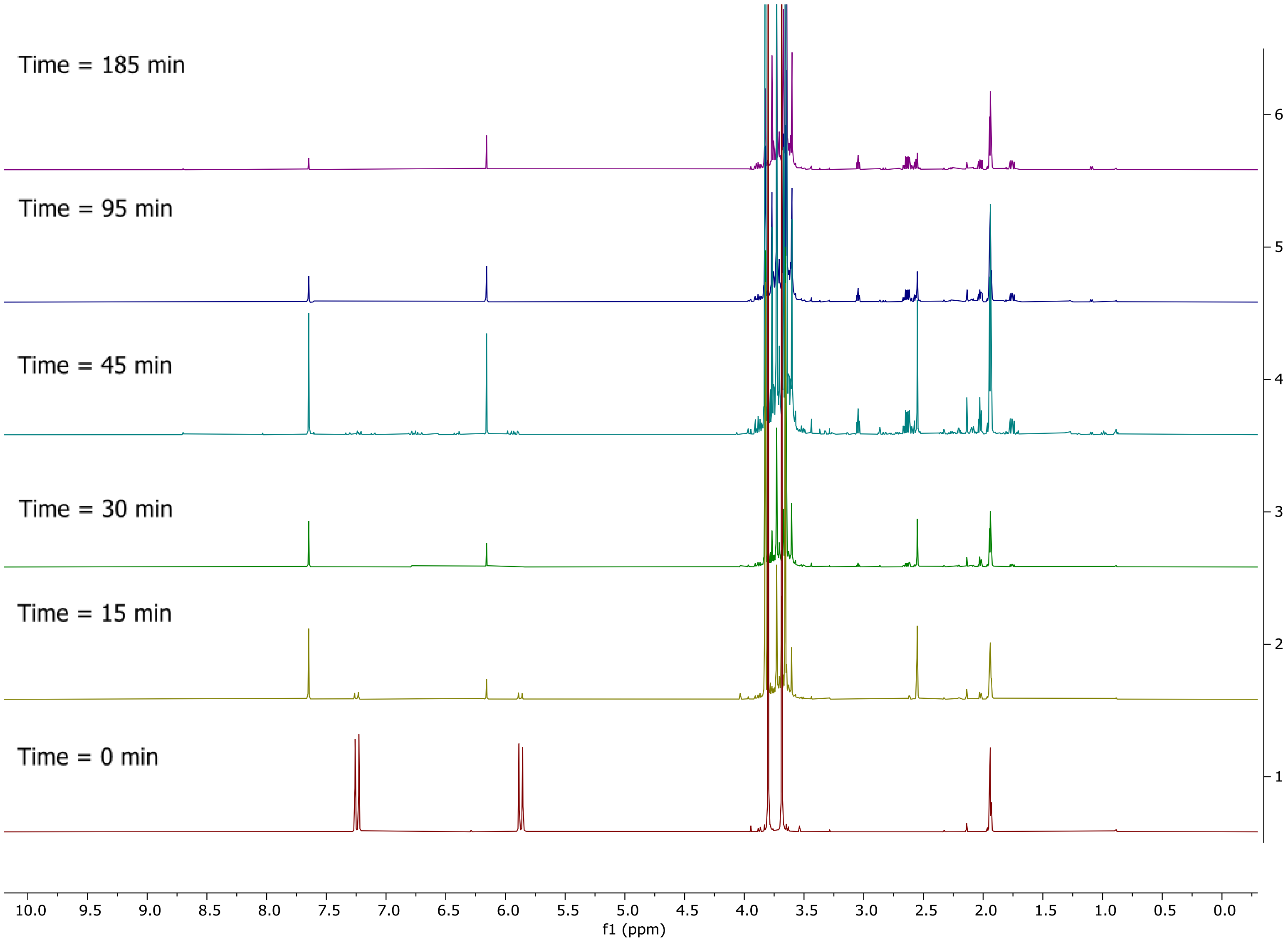

Stacked ^{1}H NMR (500 MHz, $CDCl_3$, 298 K) spectrum of 0.2 mmol (0.28 M) **2a** irradiated by 400 nm light for 1.5 h.

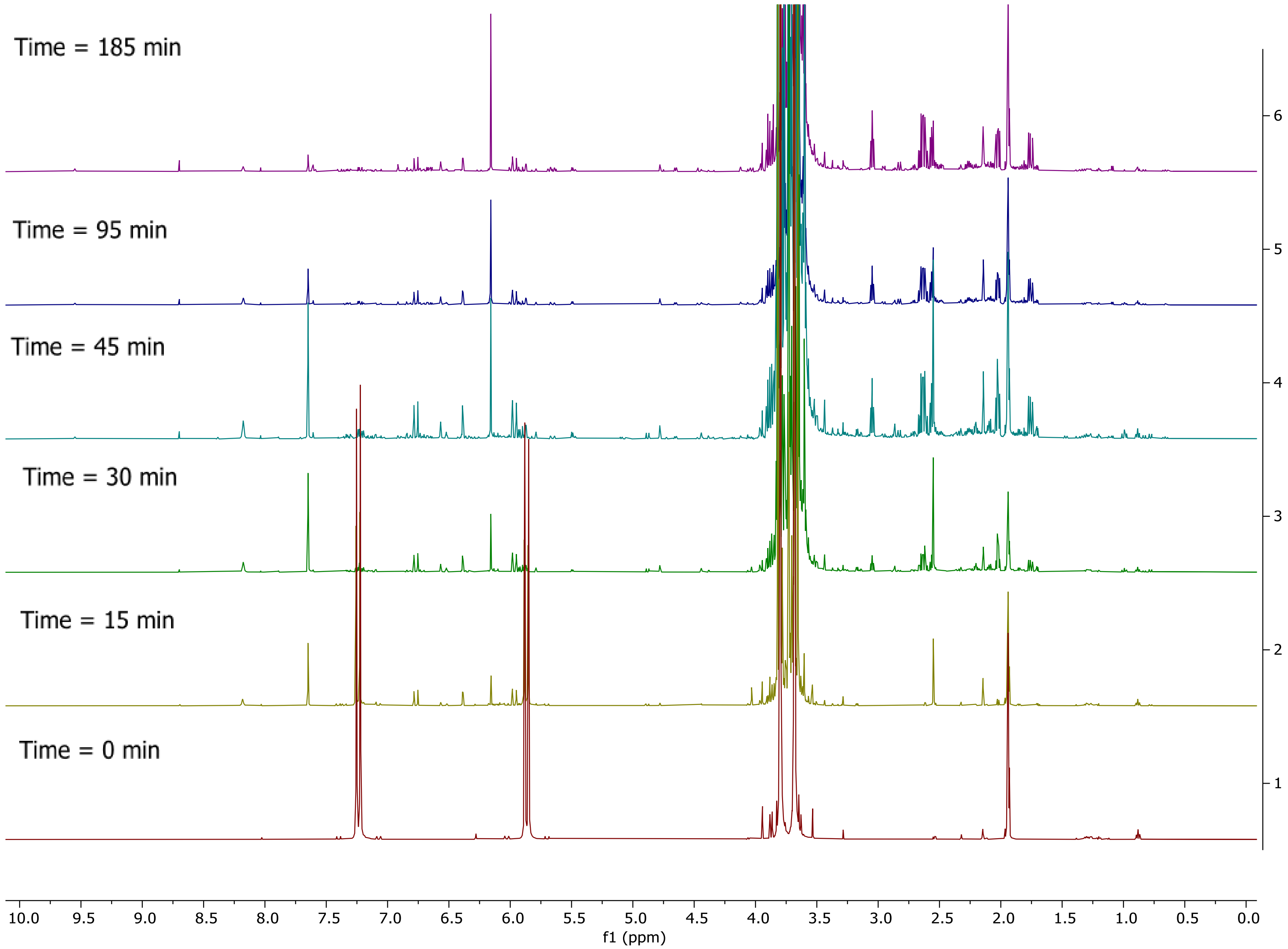

Stacked ^{1}H NMR (500 MHz, $CDCl_3$, 298 K) spectrum of 0.3 mmol (0.42 M) **2a** irradiated by 400 nm light for 1.5 h.

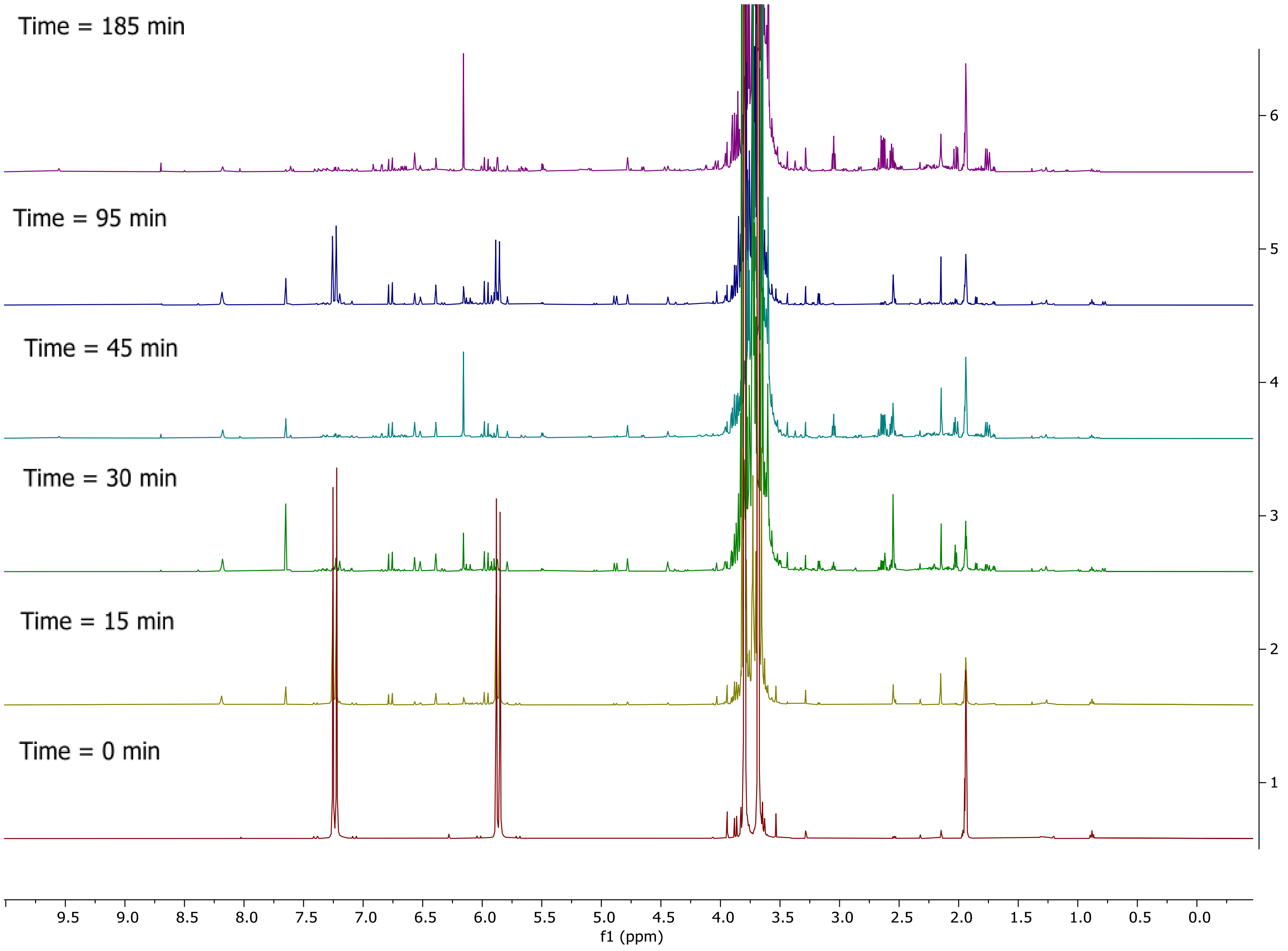

Stacked ^{1}H NMR (200 MHz, $CDCl_3$, 298 K) spectrum of **1q** irradiated by 400 nm light for 1.5 h.

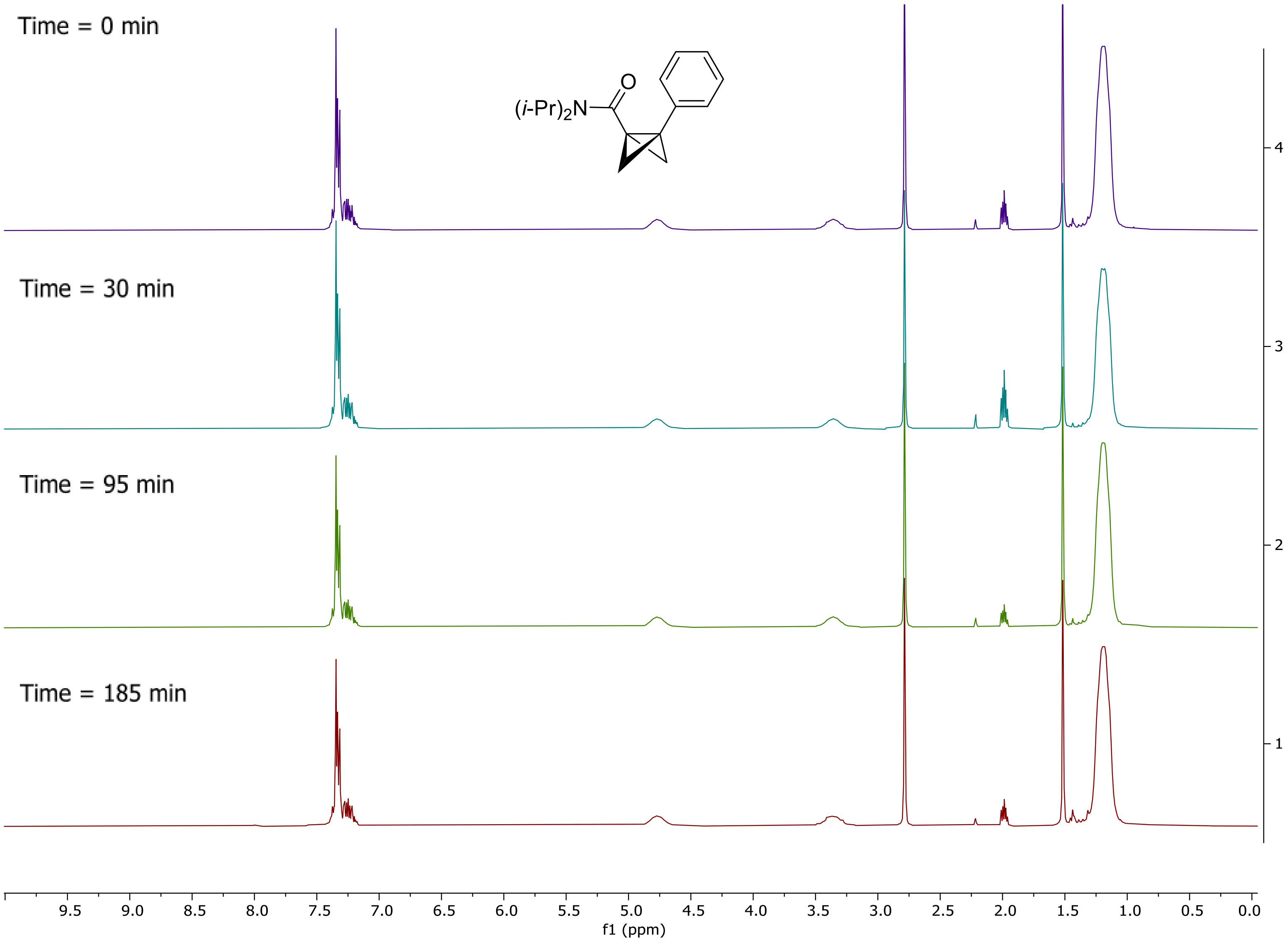

^1H NMR (500 MHz, $CDCl_3$, 298 K) spectrum of **6a** (crude reaction mixture).

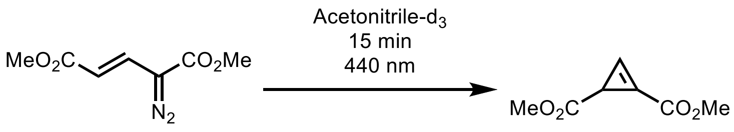

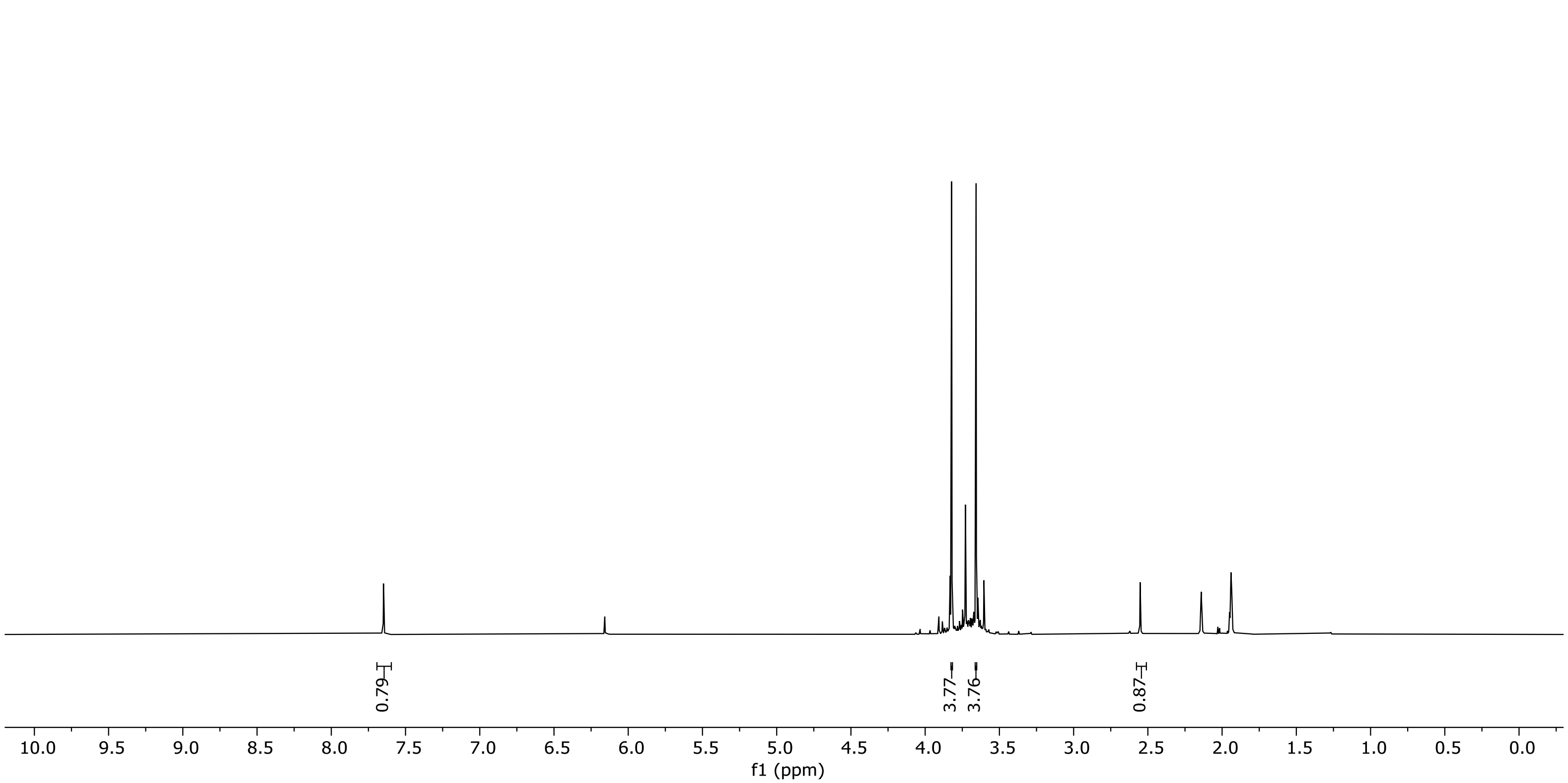

^{13}C NMR (500 MHz, $CDCl_3$, 298 K) spectrum of **6a** (Crude reaction mixture).

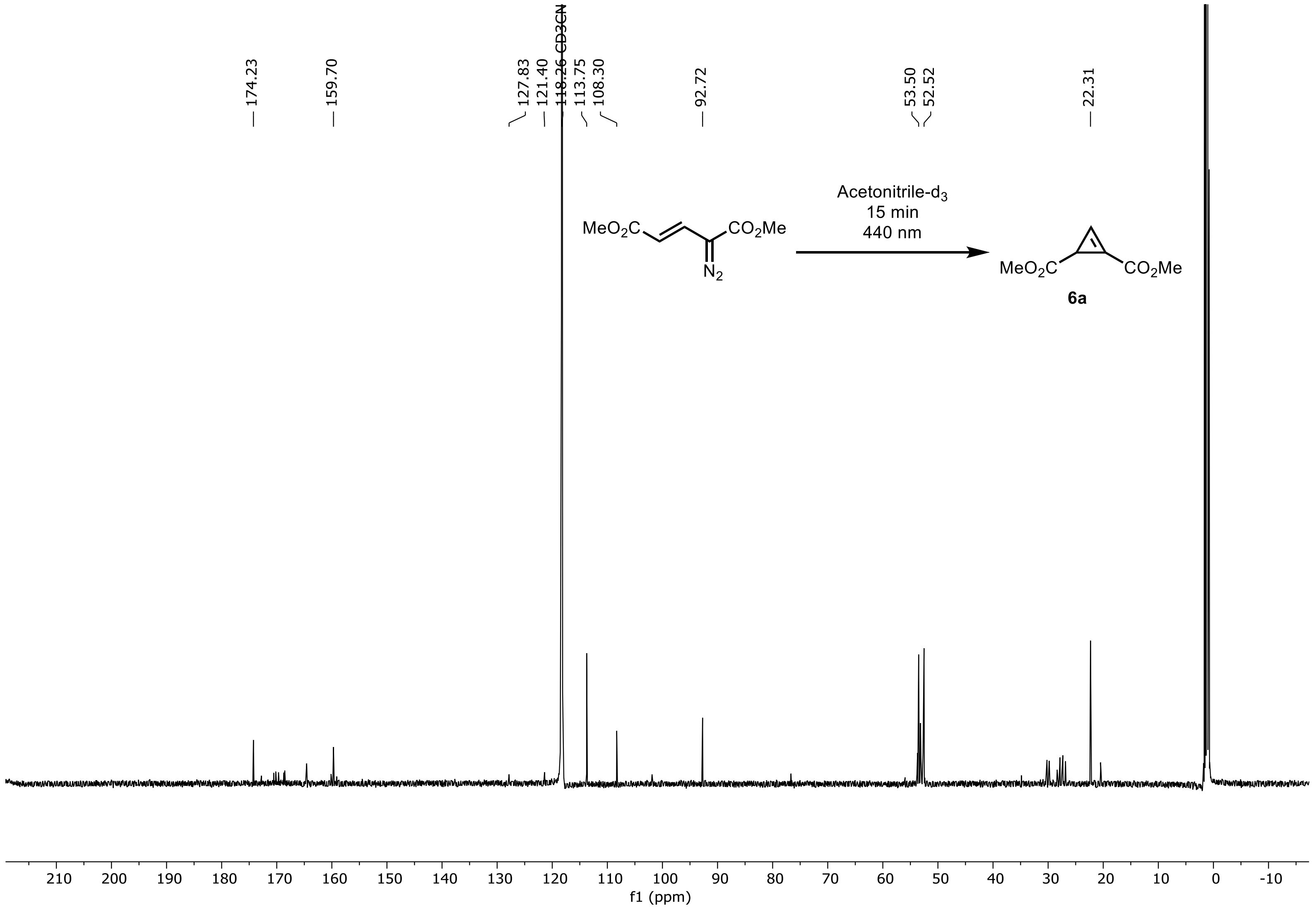

^{1}H NMR (500 MHz, $CDCl_3$, 298 K) spectrum of **6a'** (crude reaction mixture).

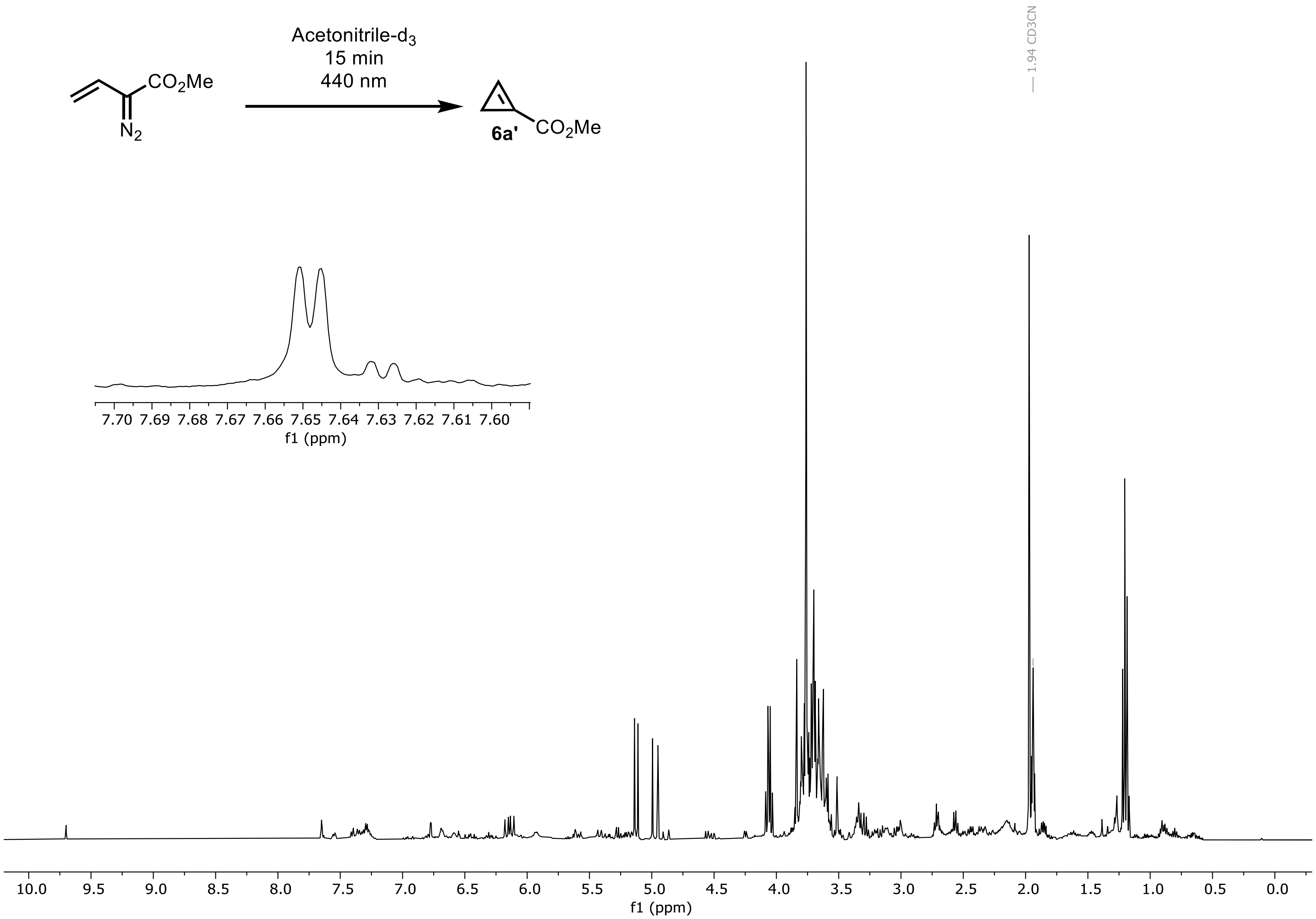

^1H NMR (500 MHz, $CDCl_3$, 298 K) spectrum of **3a**.

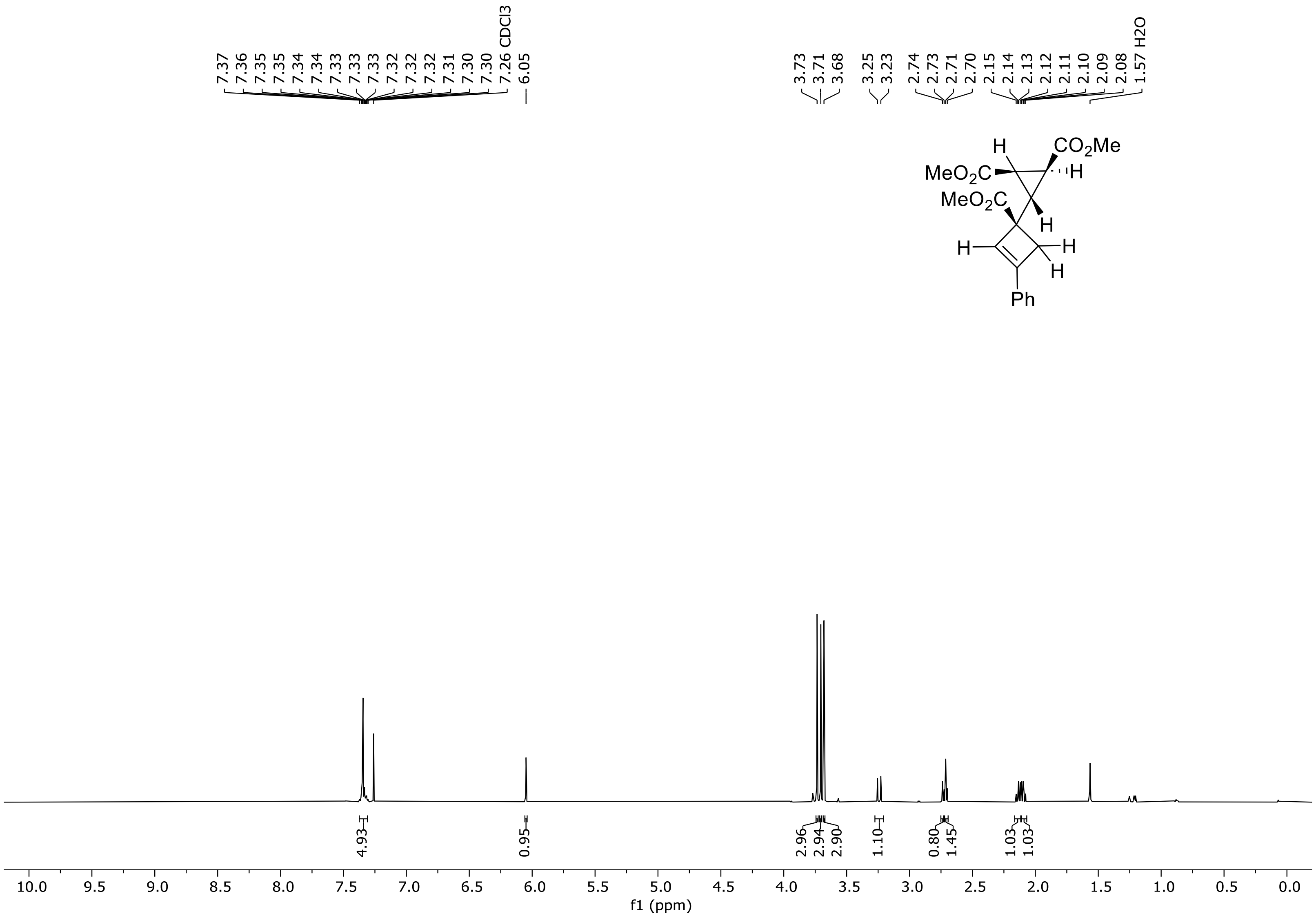

^{13}C NMR (126 MHz, $CDCl_3$, 298 K) spectrum of **3a**.

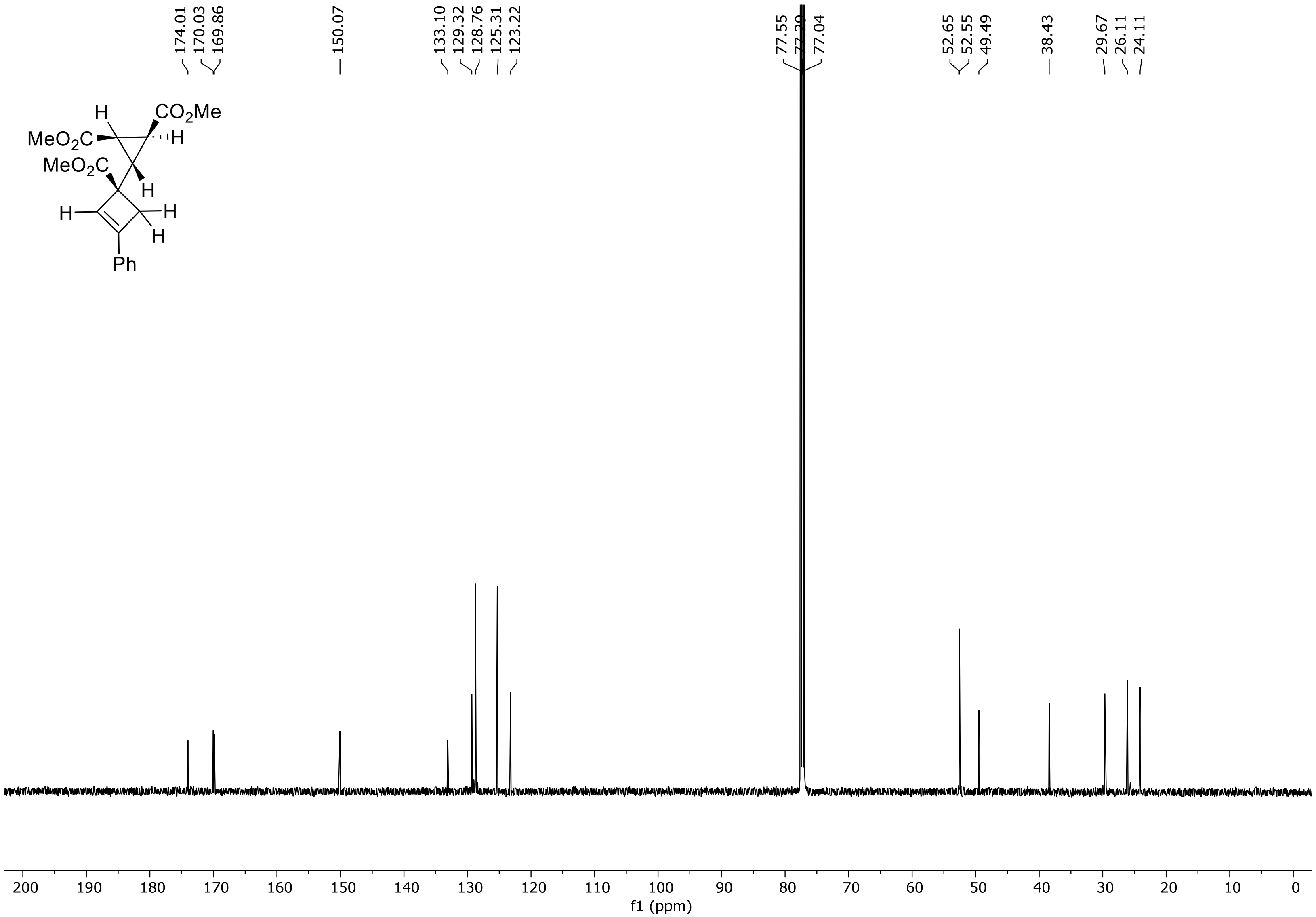

COSY (^{1}H–^{1}H) NMR (500 MHz, $CDCl_3$, 298 K) spectrum of **3a**.

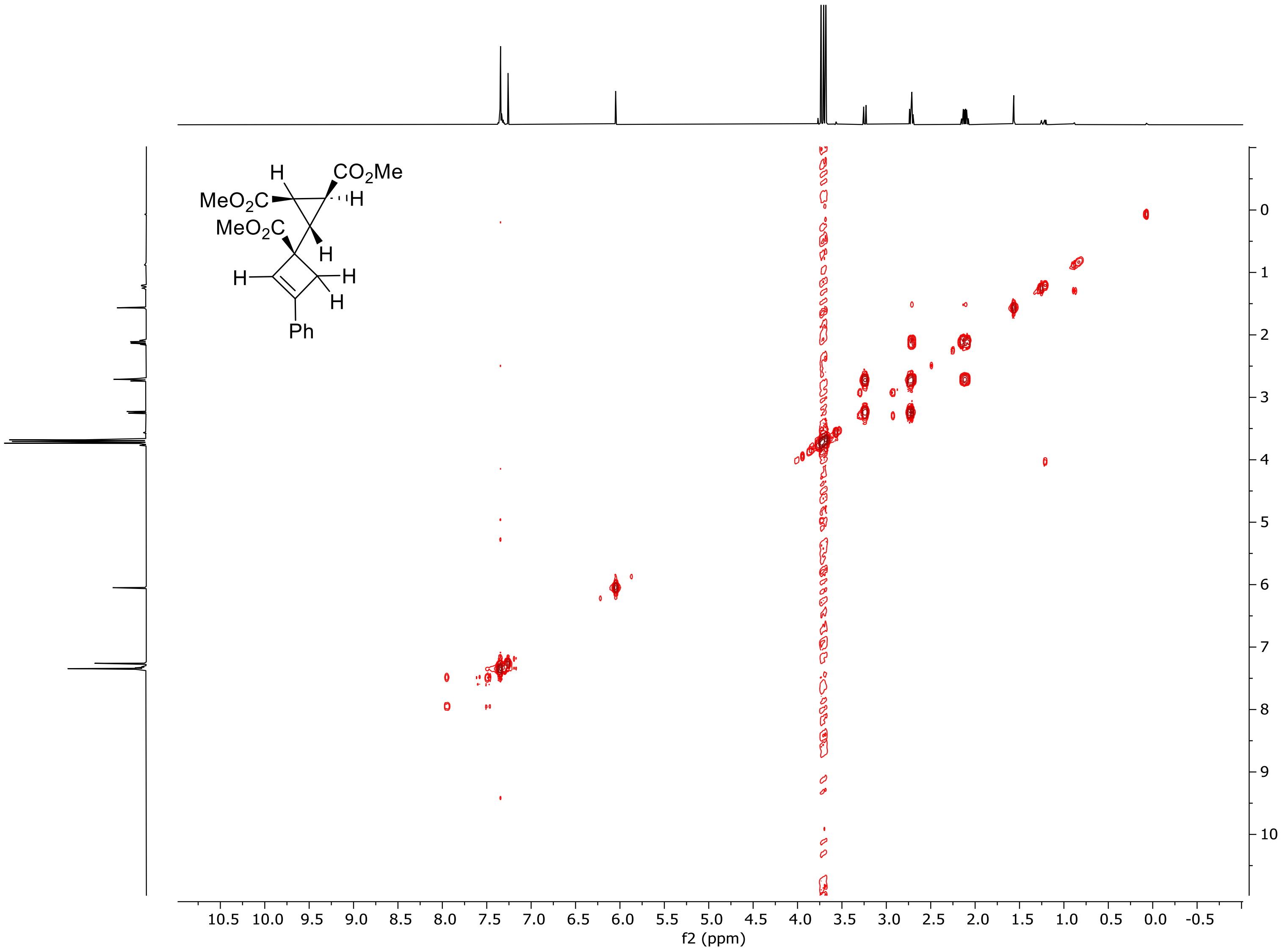

HSQC (^1H–^{13}C) NMR (500 MHz, $CDCl_3$, 298 K) spectrum of **3a**.

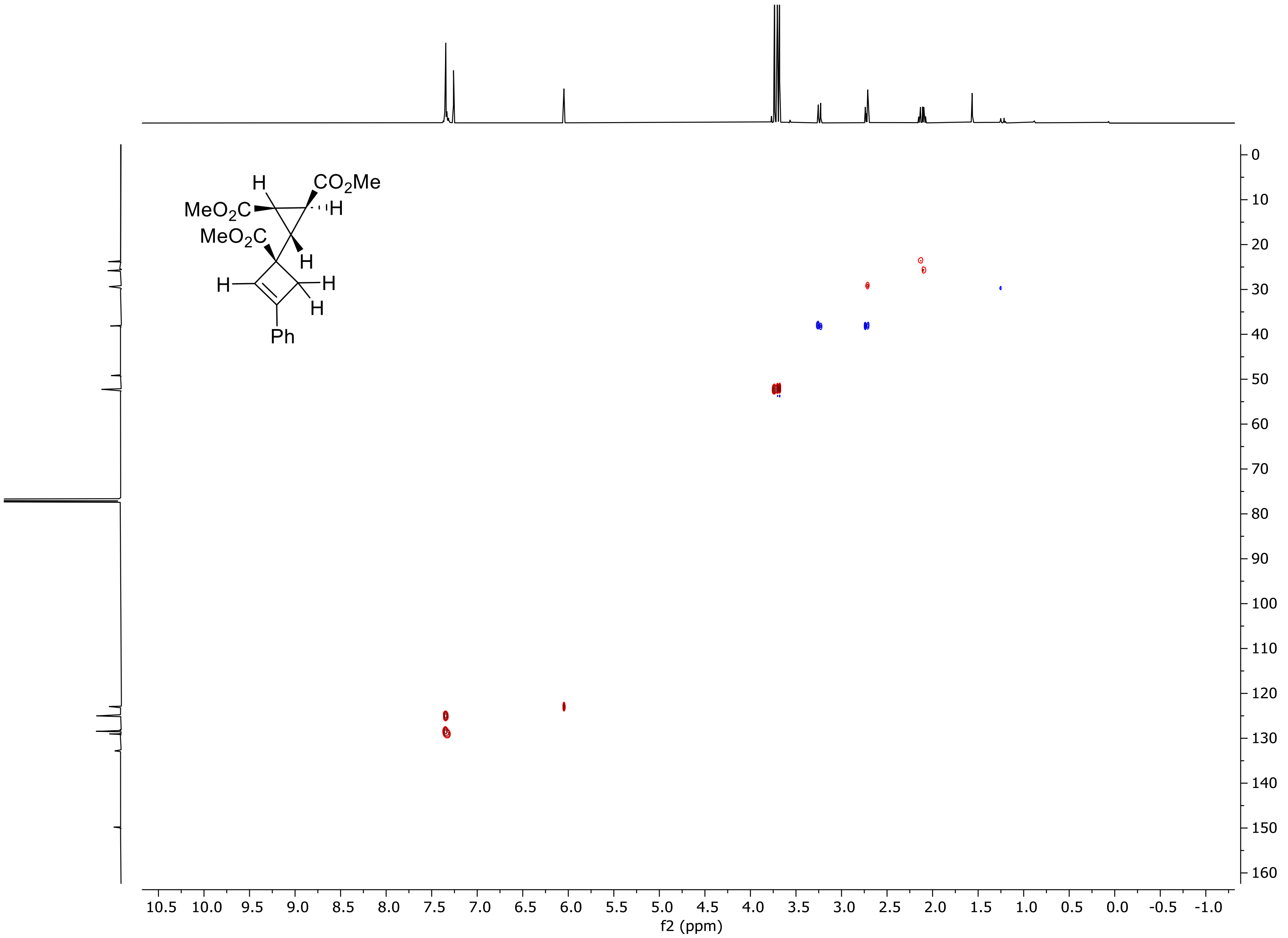

^{1}H NMR (500 MHz, $CDCl_3$, 298 K) spectrum of **3b**.

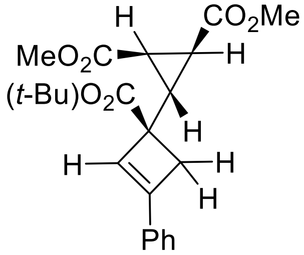

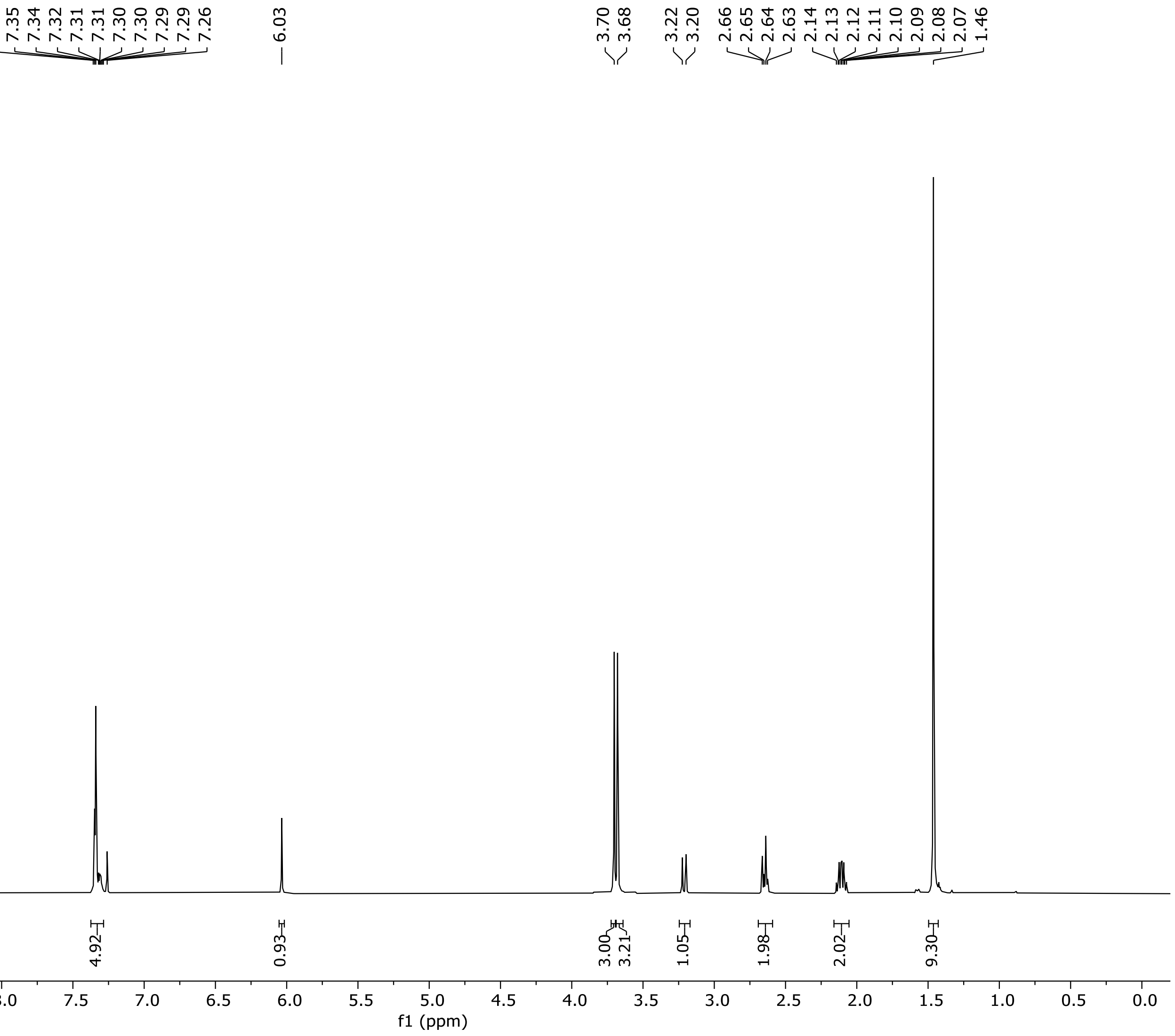

^{13}C NMR (126 MHz, $CDCl_3$, 298 K) spectrum of **3b**.

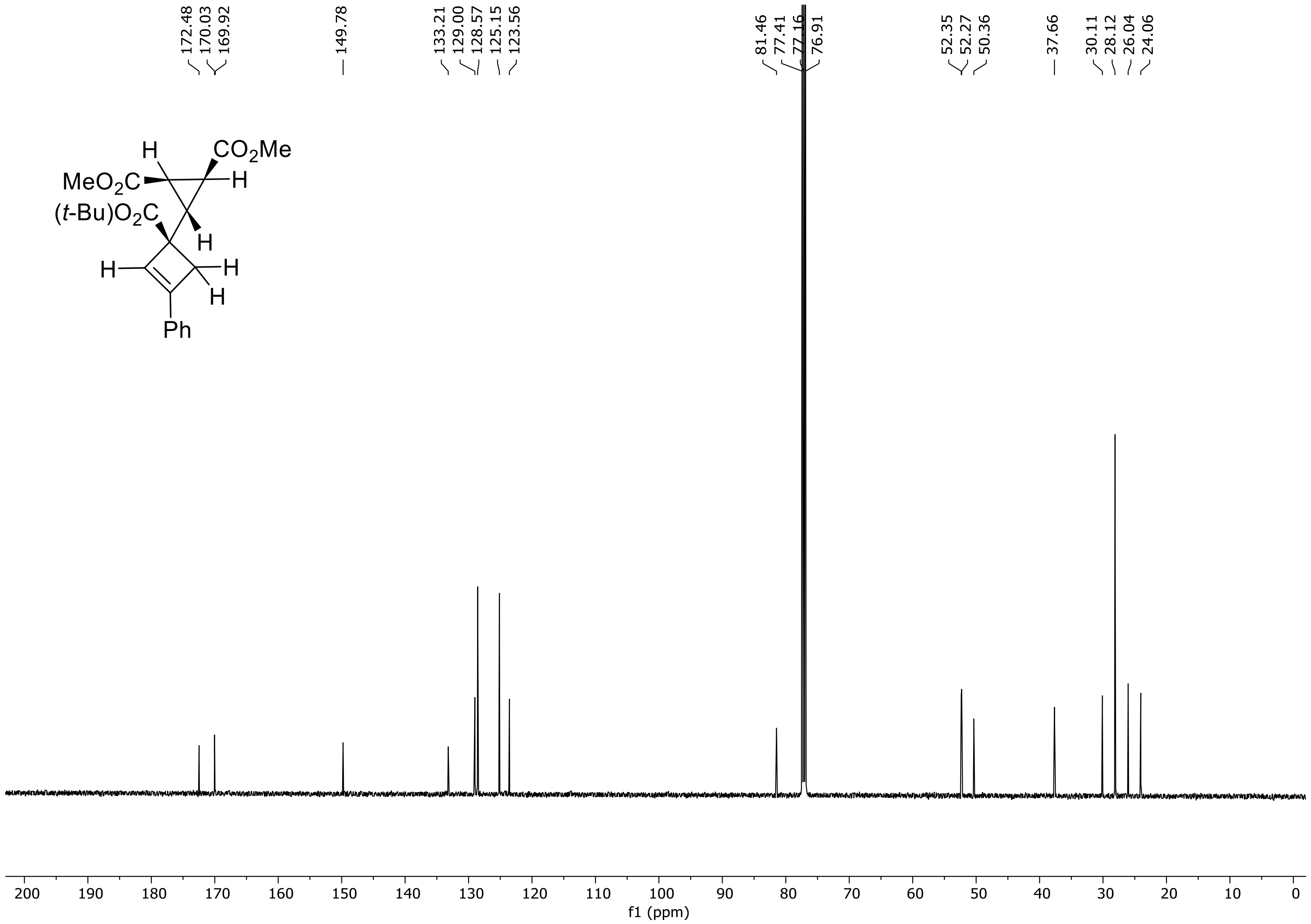

^{1}H NMR (500 MHz, $CDCl_3$, 298 K) spectrum of **3c**.

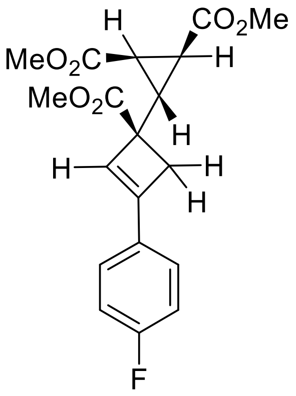

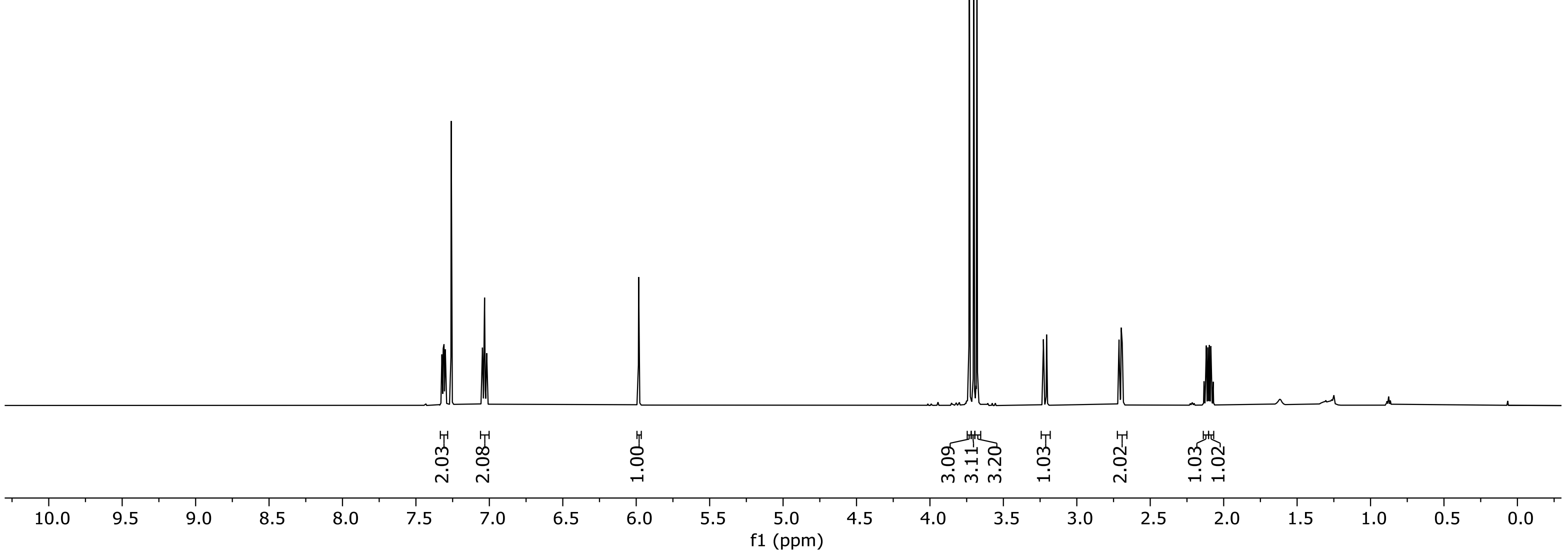

^{13}C NMR (126 MHz, $CDCl_3$, 298 K) spectrum of **3c**.

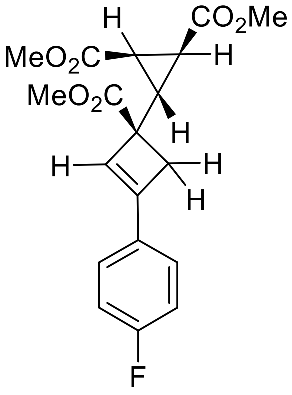

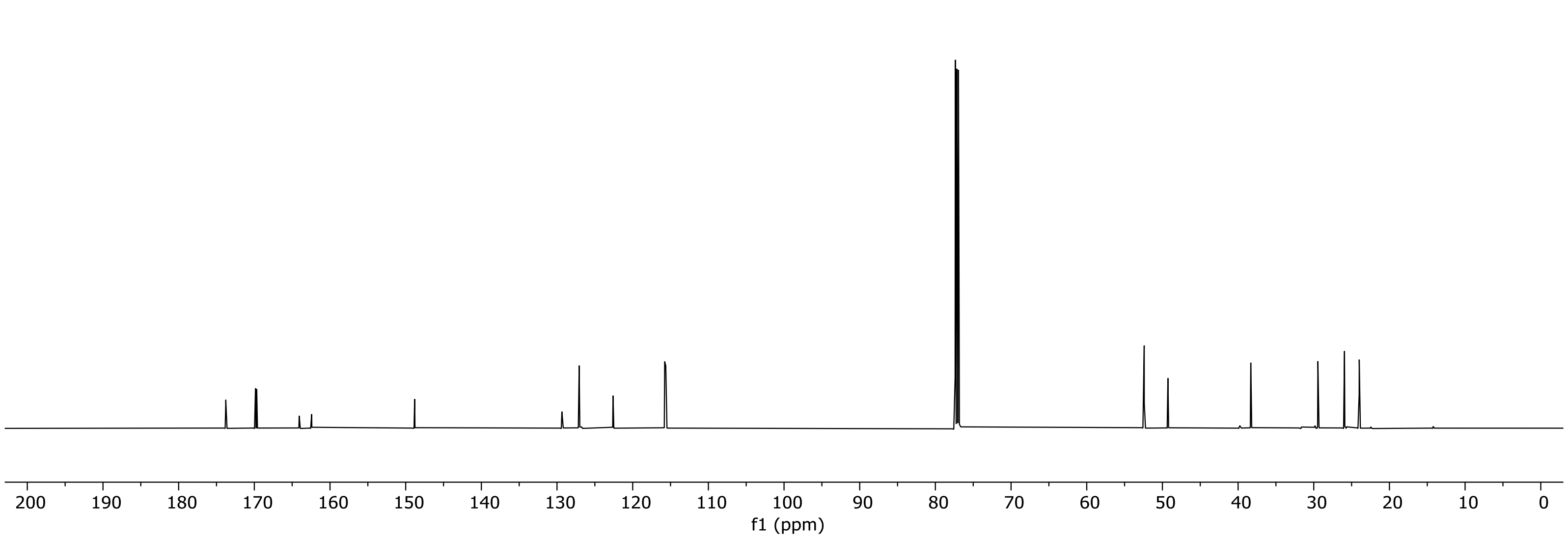

^{19}F NMR (470 MHz, $CDCl_3$, 298 K) spectrum of **3c**.

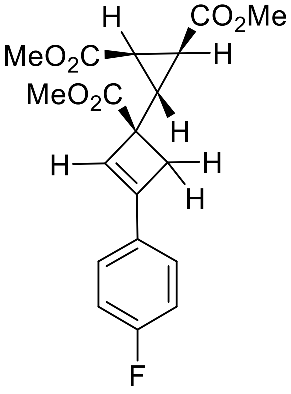

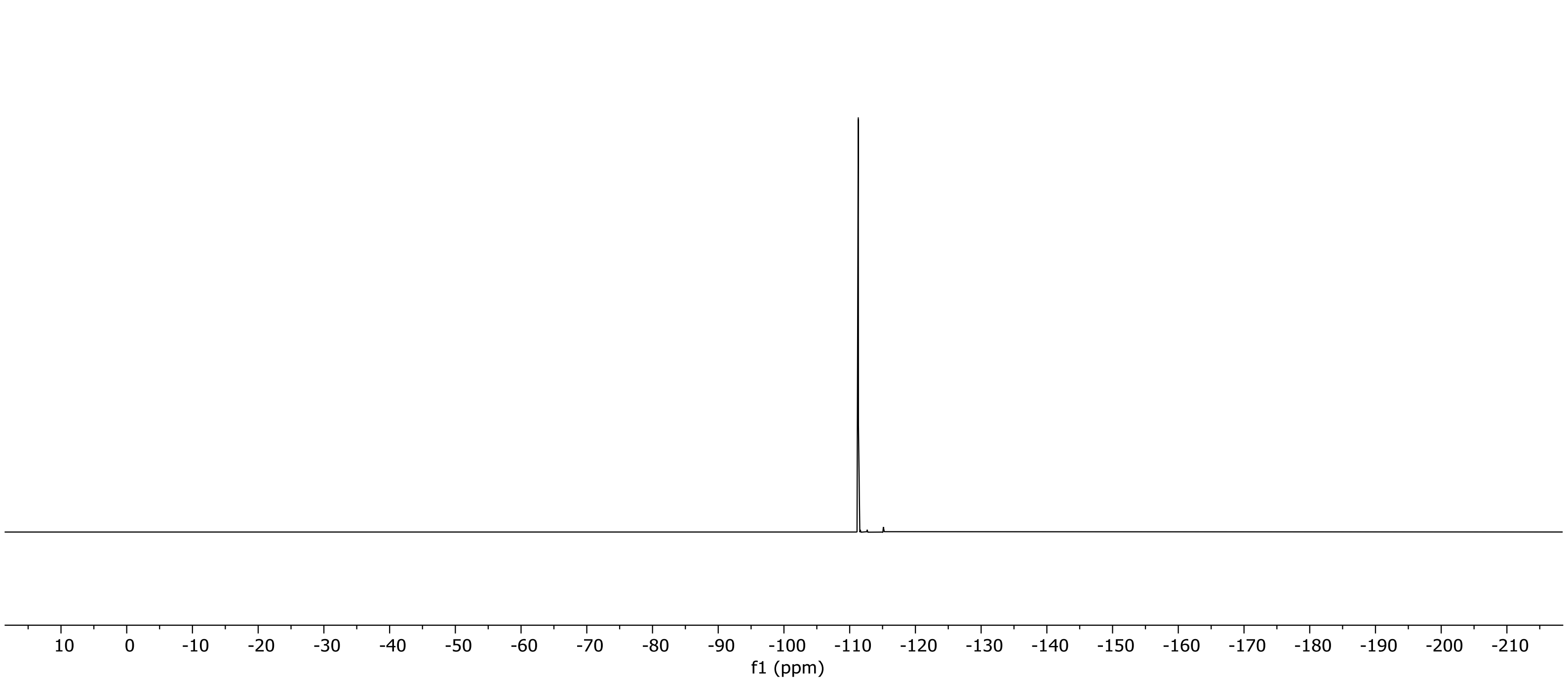

^{1}H NMR (500 MHz, $CDCl_3$, 298 K) spectrum of **3d**.

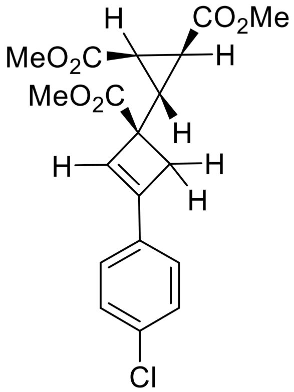

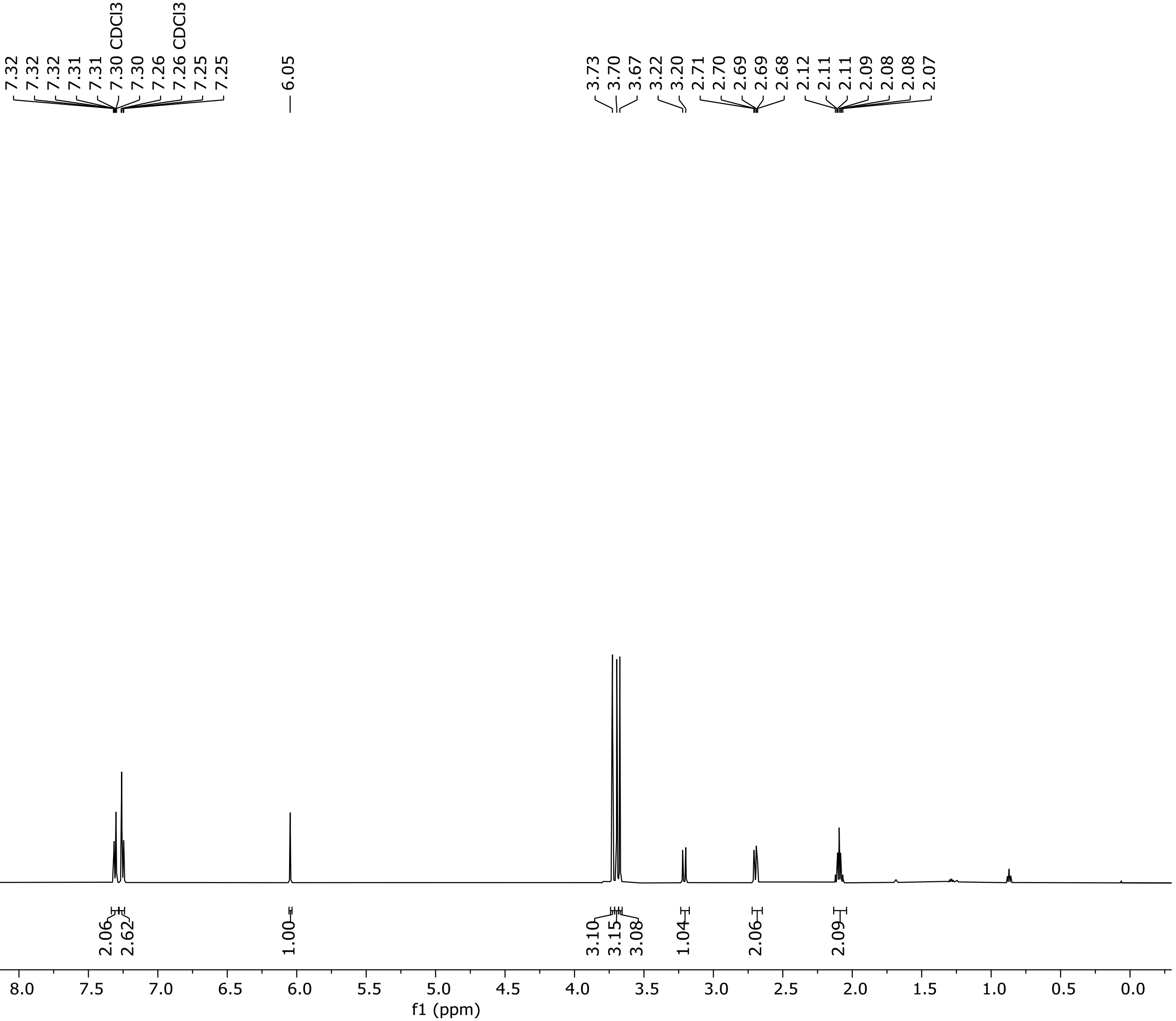

^{13}C NMR (126 MHz, $CDCl_3$, 298 K) spectrum of **3d**.

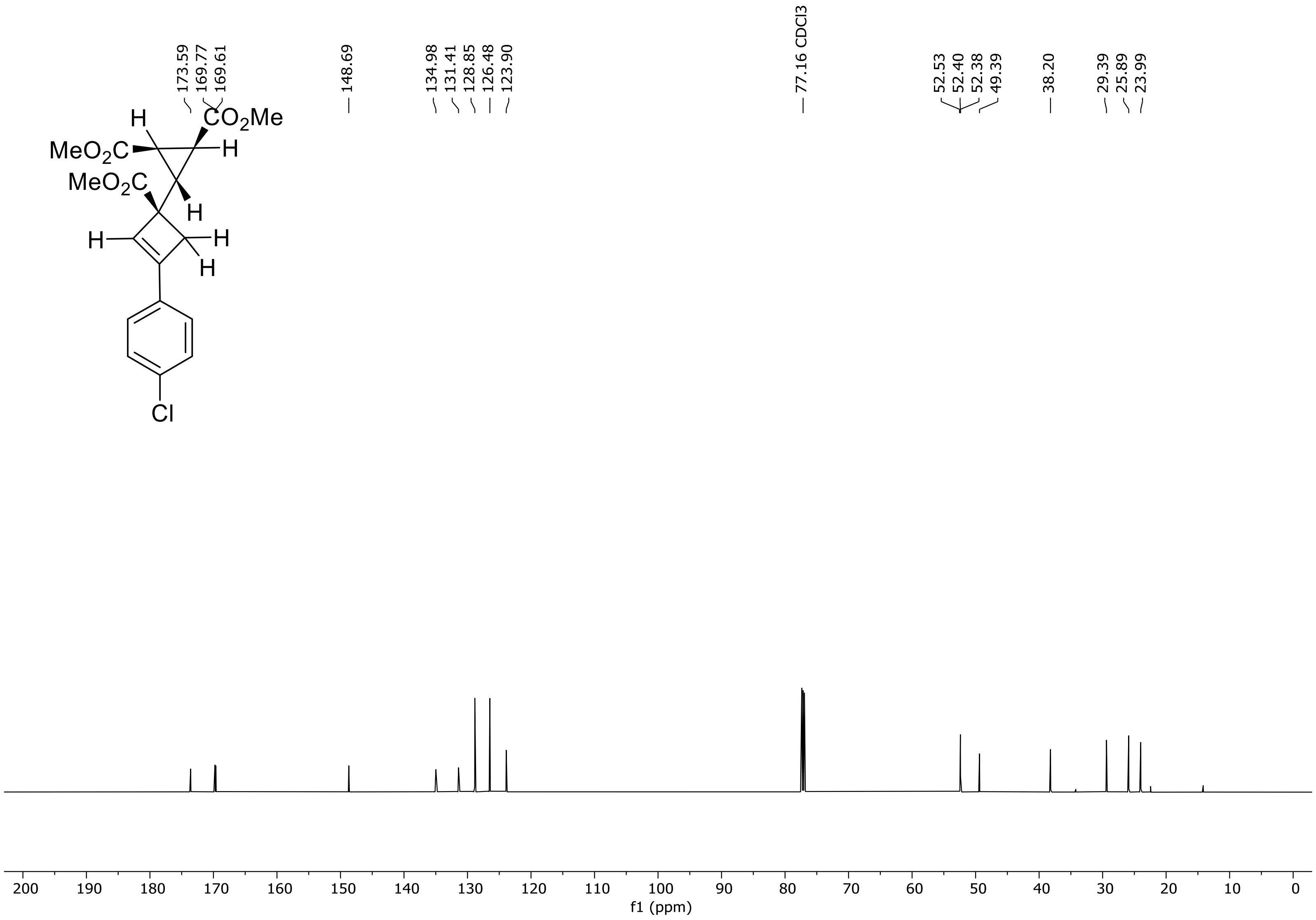

^{1}H NMR (600 MHz, $CDCl_3$, 298 K) spectrum of **3e**.

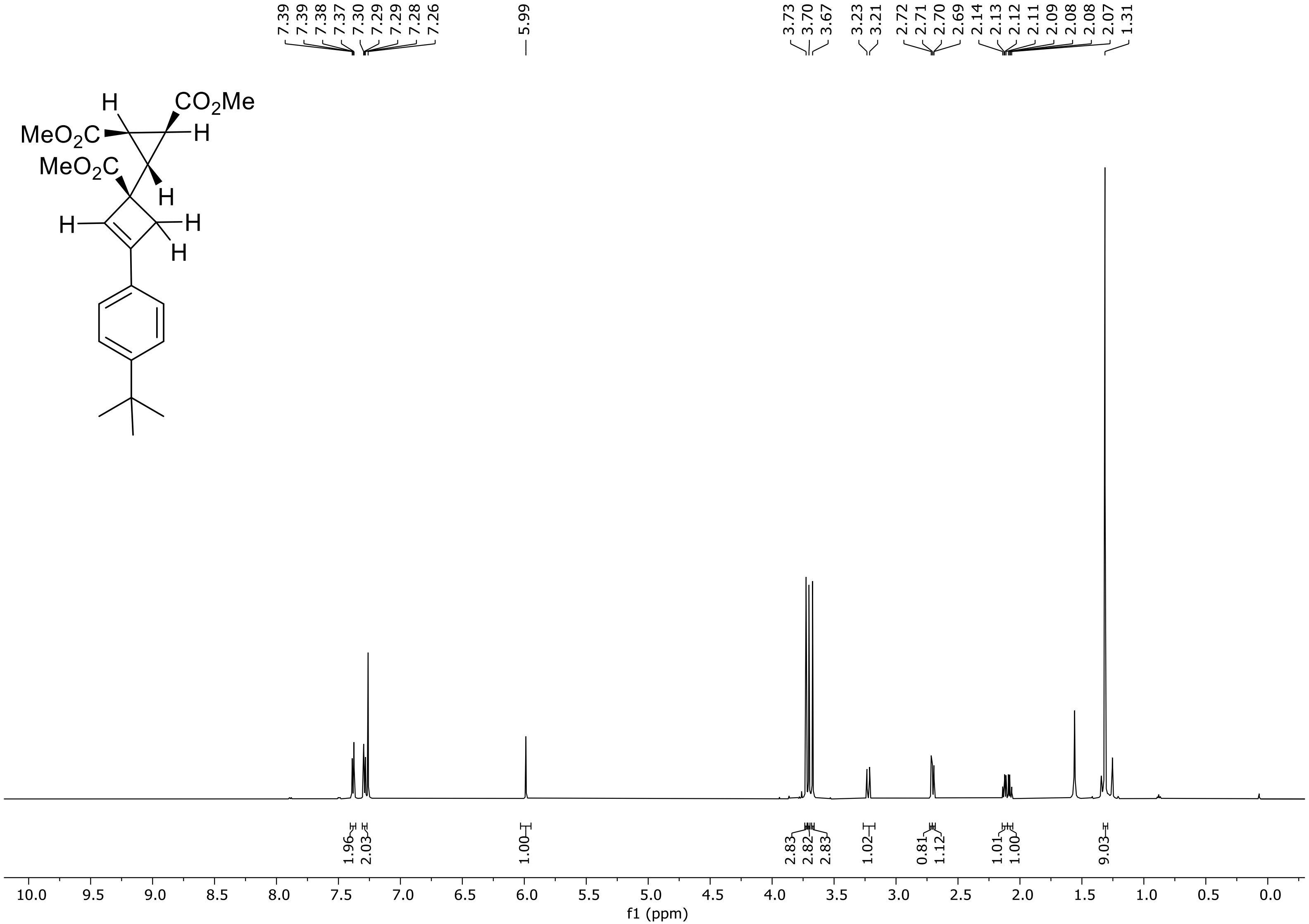

^{13}C NMR (151 MHz, $CDCl_3$, 298 K) spectrum of **3e**.

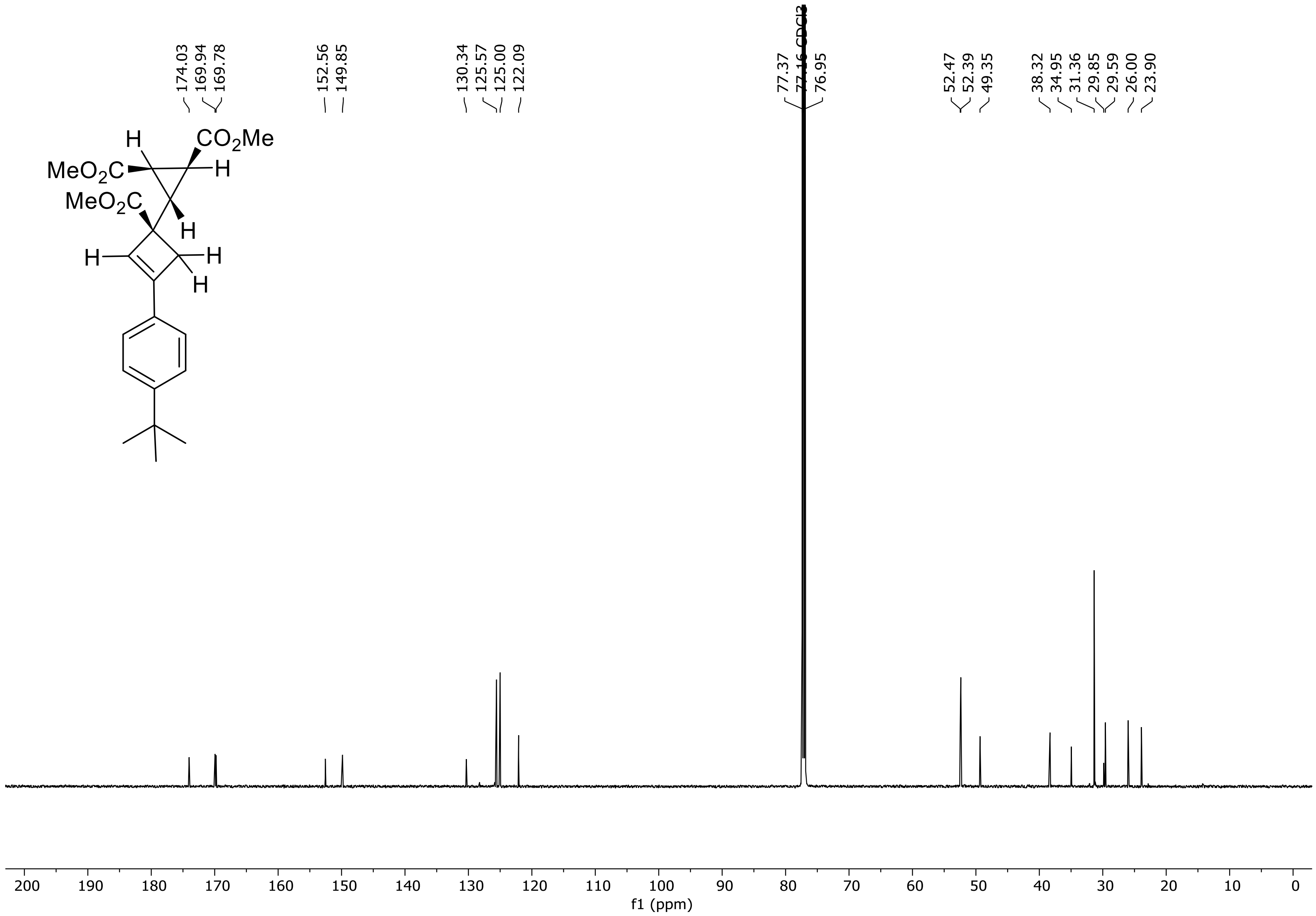

^{1}H NMR (500 MHz, $CDCl_3$, 298 K) spectrum of **3f**.

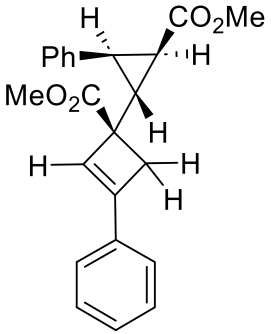

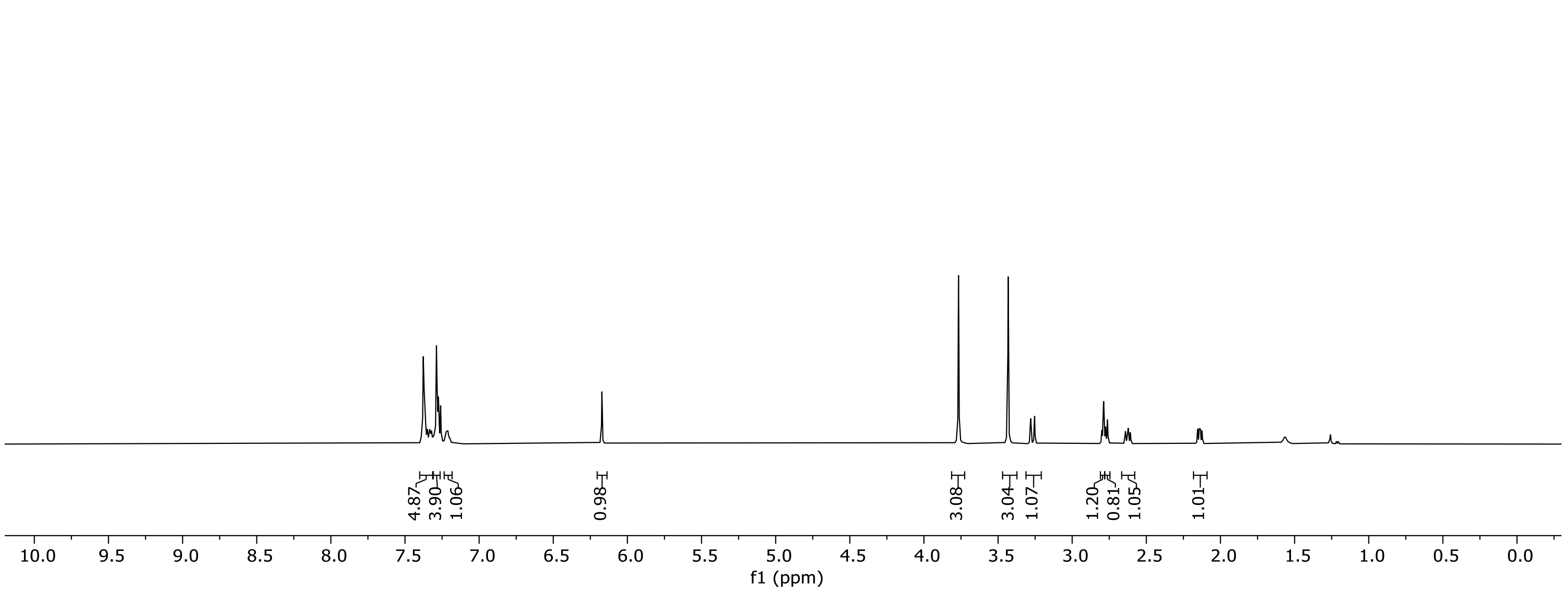

^{13}C NMR (126 MHz, $CDCl_3$, 298 K) spectrum of **3f**.

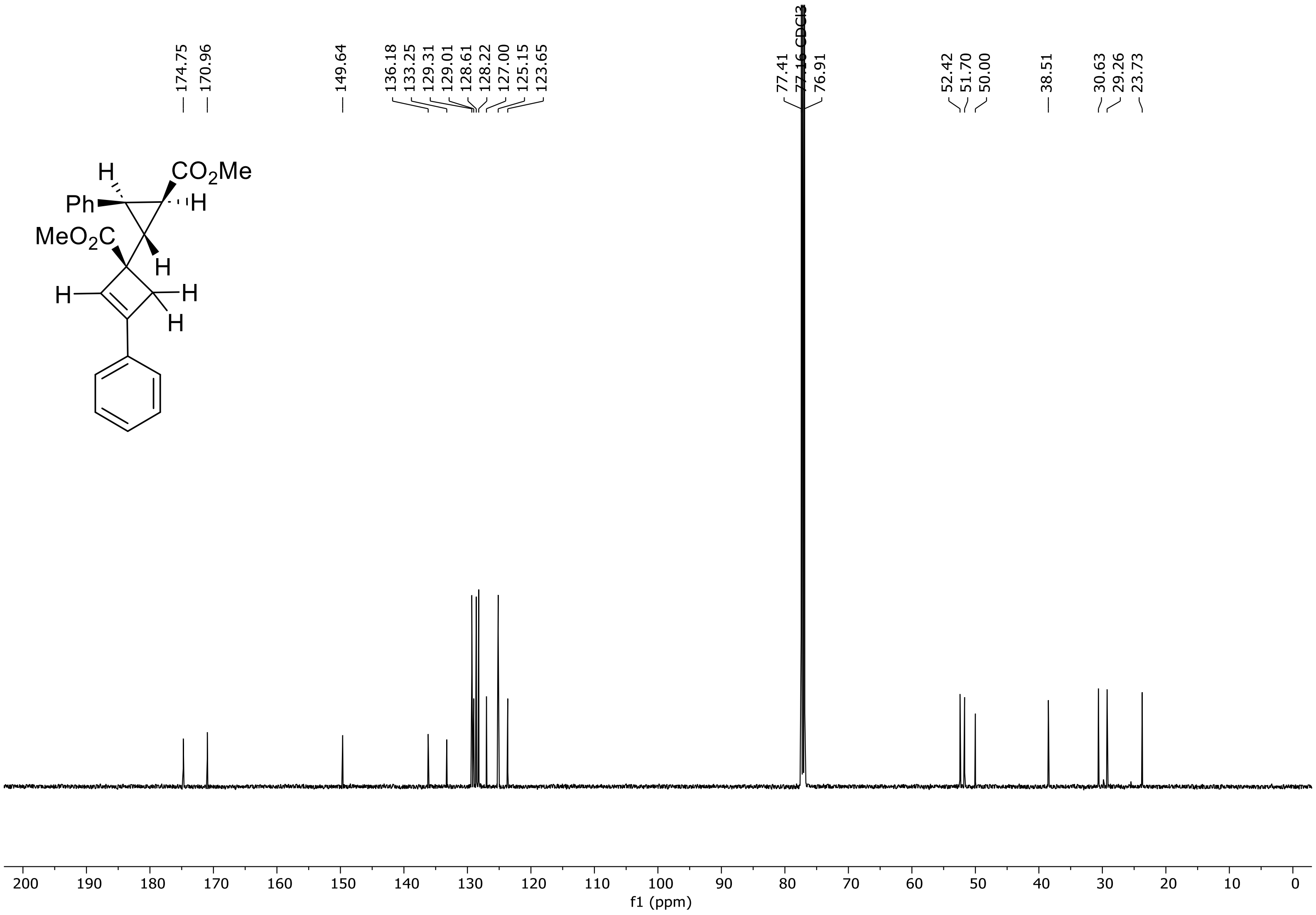

^{1}H NMR (600 MHz, $CDCl_3$, 298 K) spectrum of **3g**.

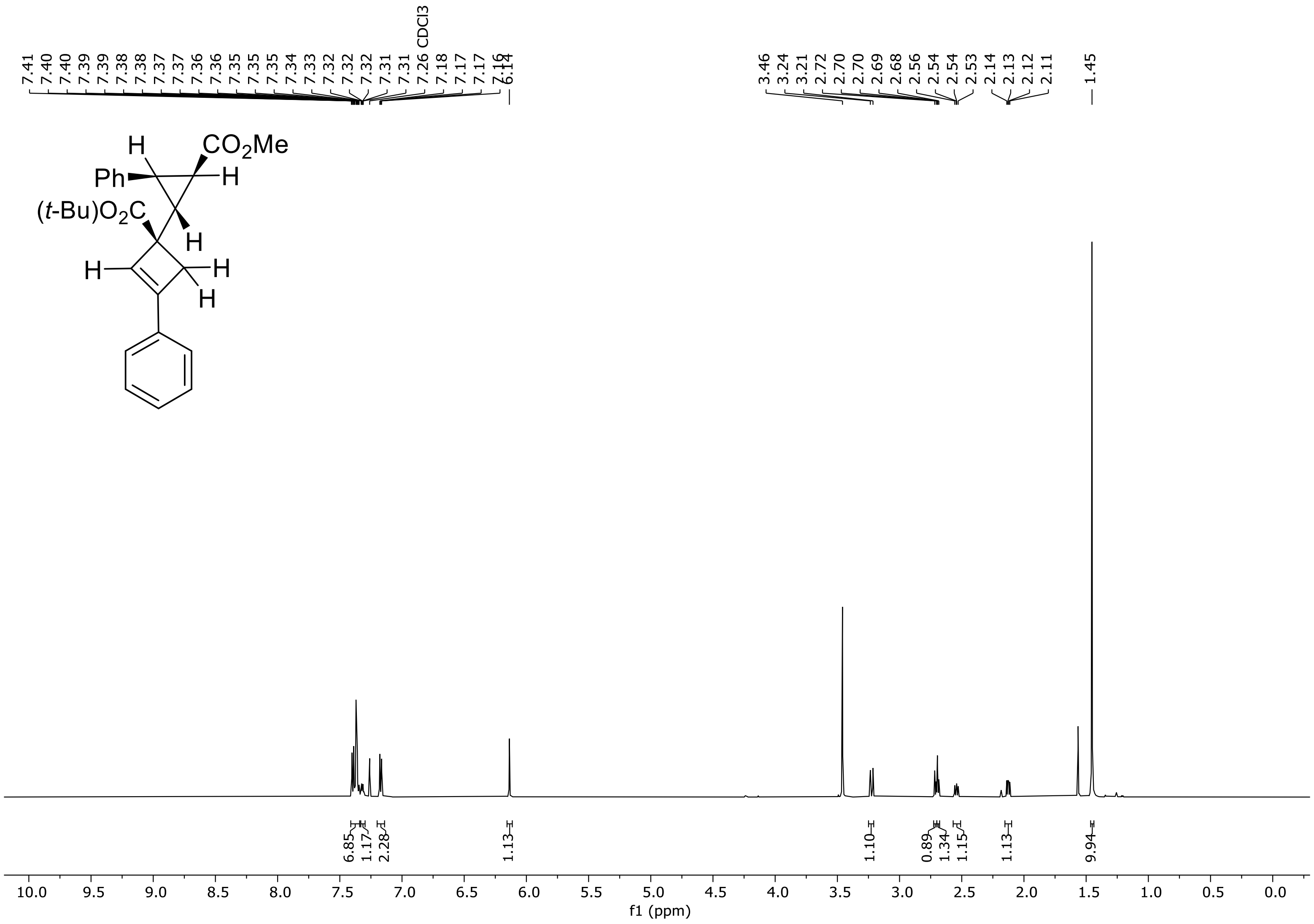

^{13}C NMR (151 MHz, $CDCl_3$, 298 K) spectrum of **3g**.

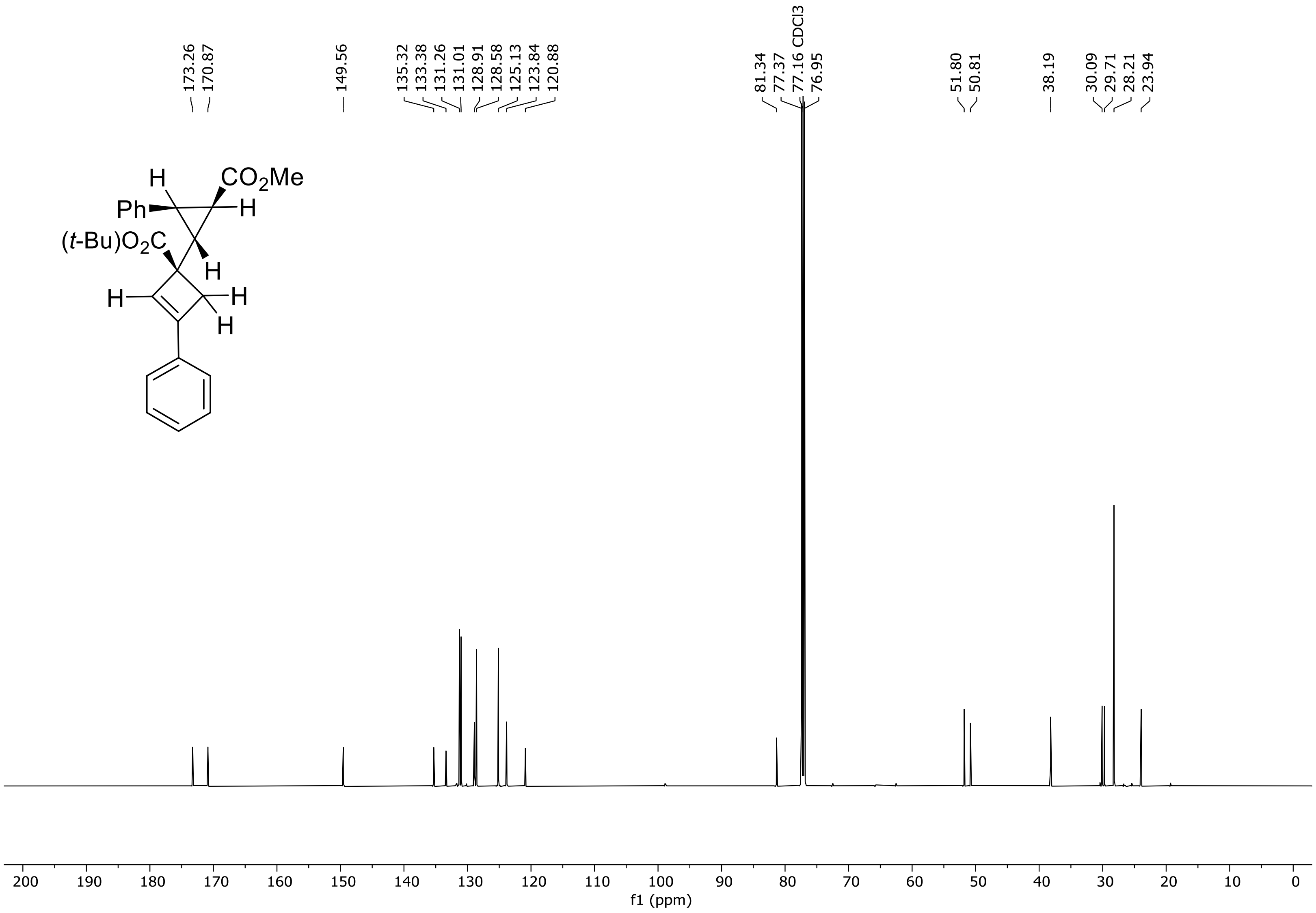

^{1}H NMR (600 MHz, $CDCl_3$, 298 K) spectrum of **3h**.

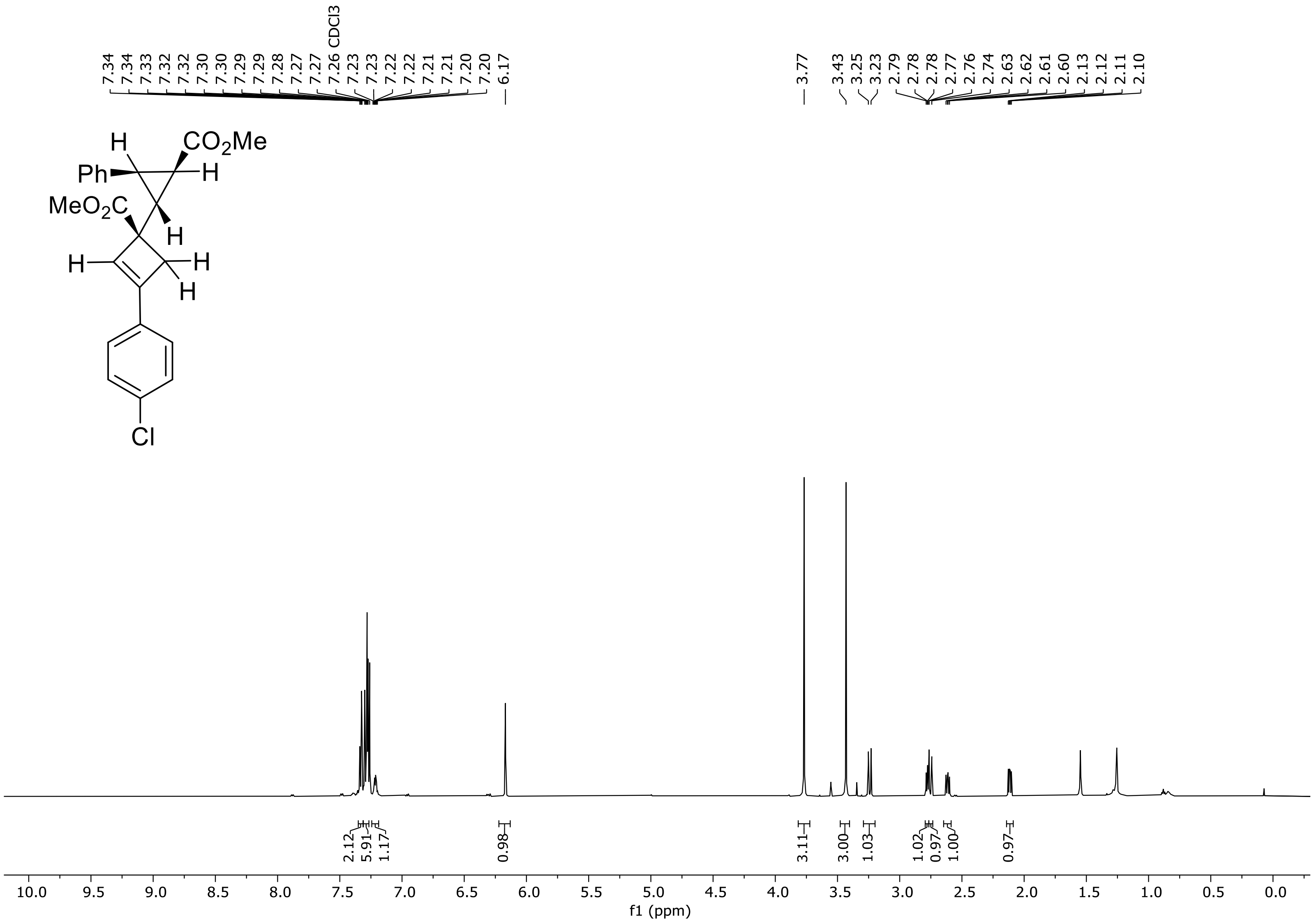

^{13}C NMR (151 MHz, $CDCl_3$, 298 K) spectrum of **3h**.

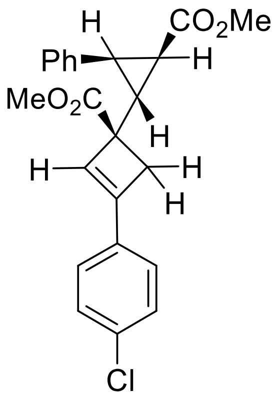

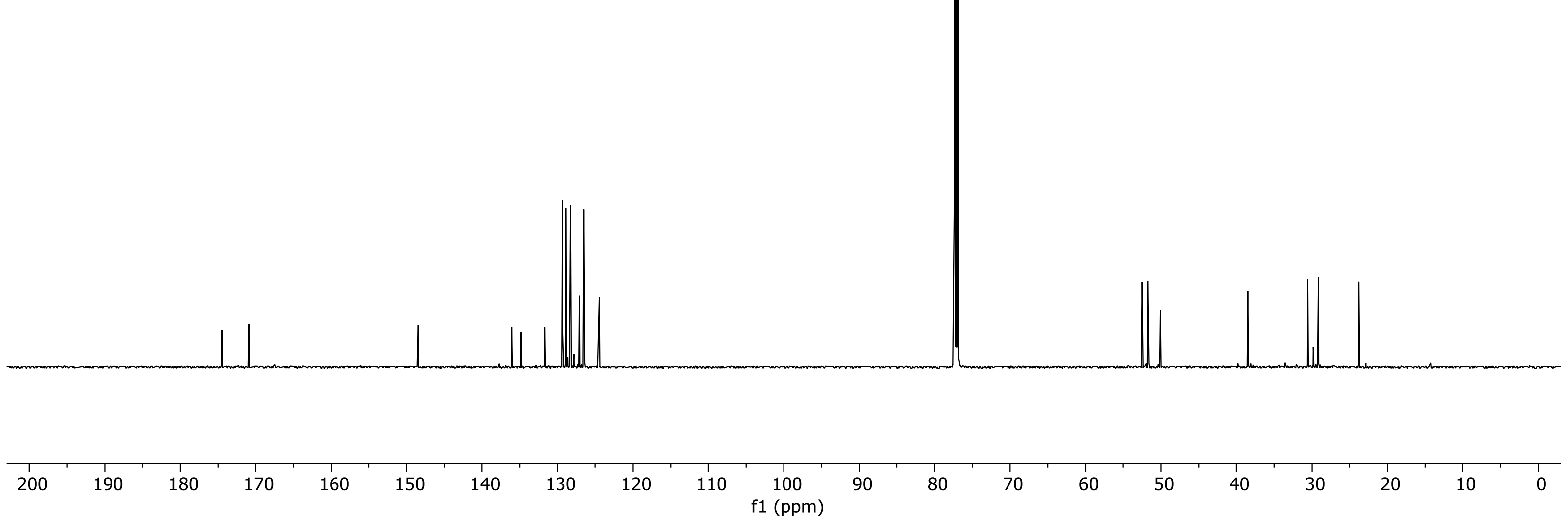

^{1}H NMR (600 MHz, $CDCl_3$, 298 K) spectrum of **3i**.

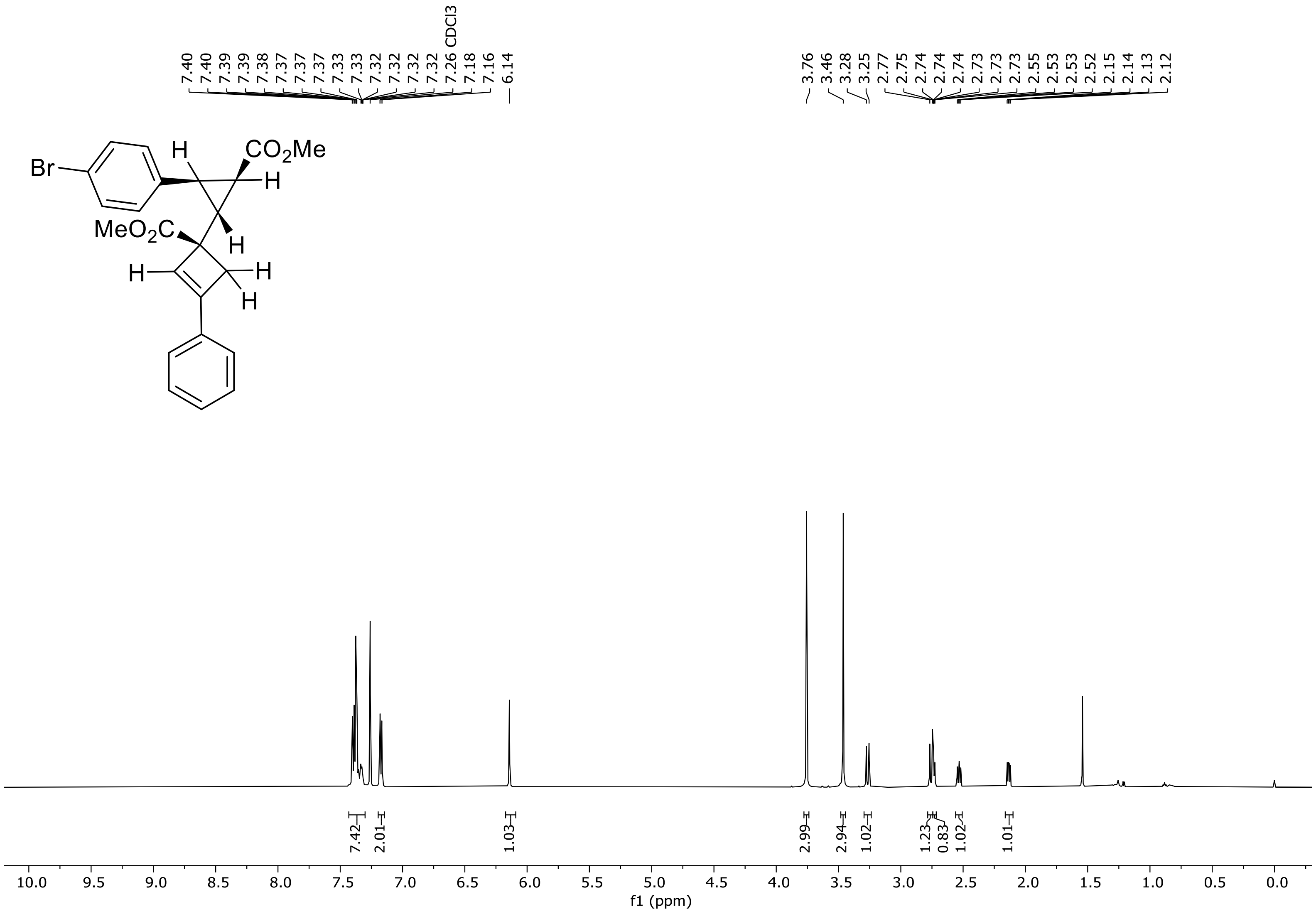

^{13}C NMR (151 MHz, $CDCl_3$, 298 K) spectrum of **3i**.

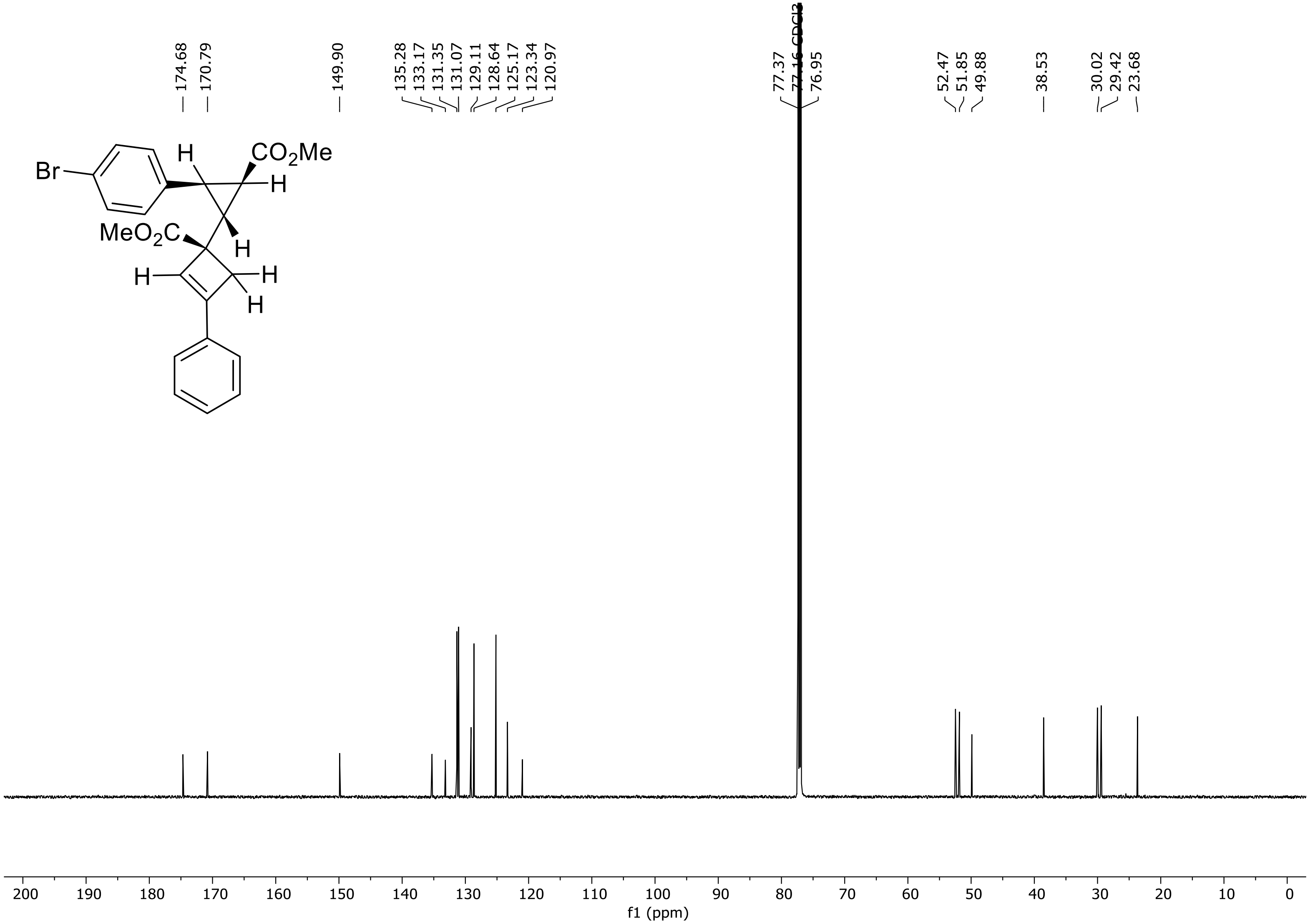

^{1}H NMR (600 MHz, $CDCl_3$, 298 K) spectrum of **3j**.

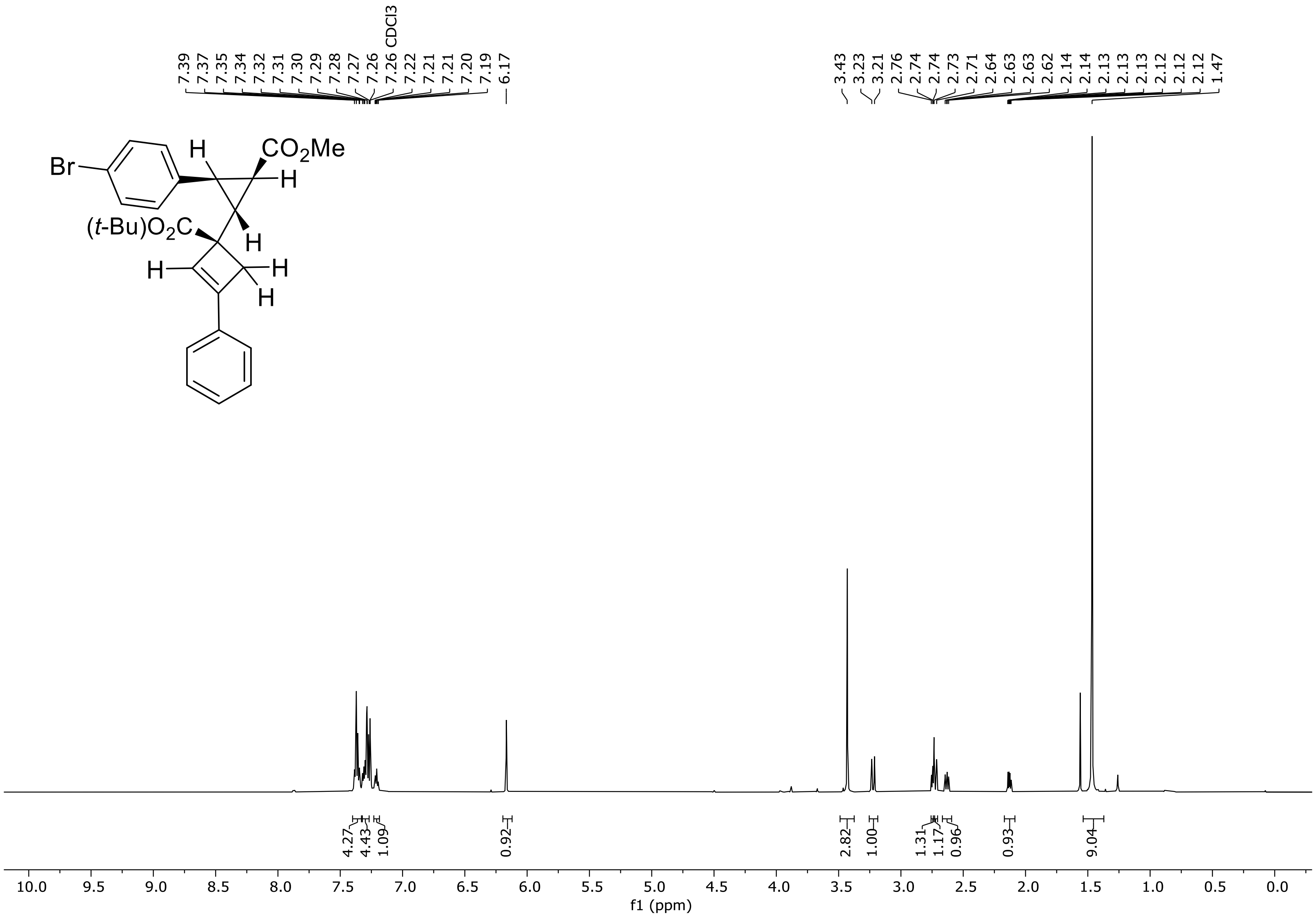

^{13}C NMR (151 MHz, $CDCl_3$, 298 K) spectrum of **3j**.

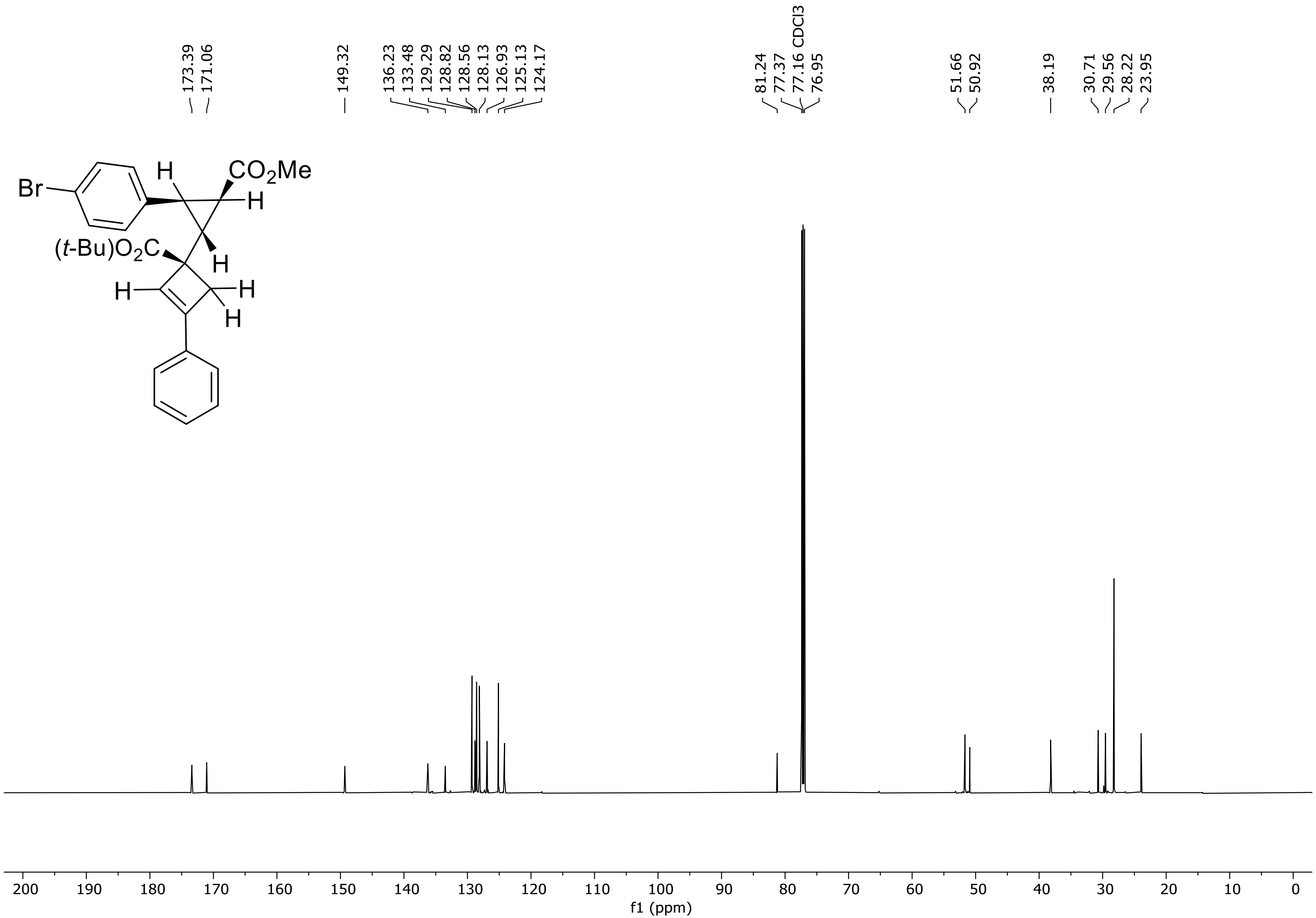

^{1}H NMR (600 MHz, $CDCl_3$, 298 K) spectrum of **3k**.

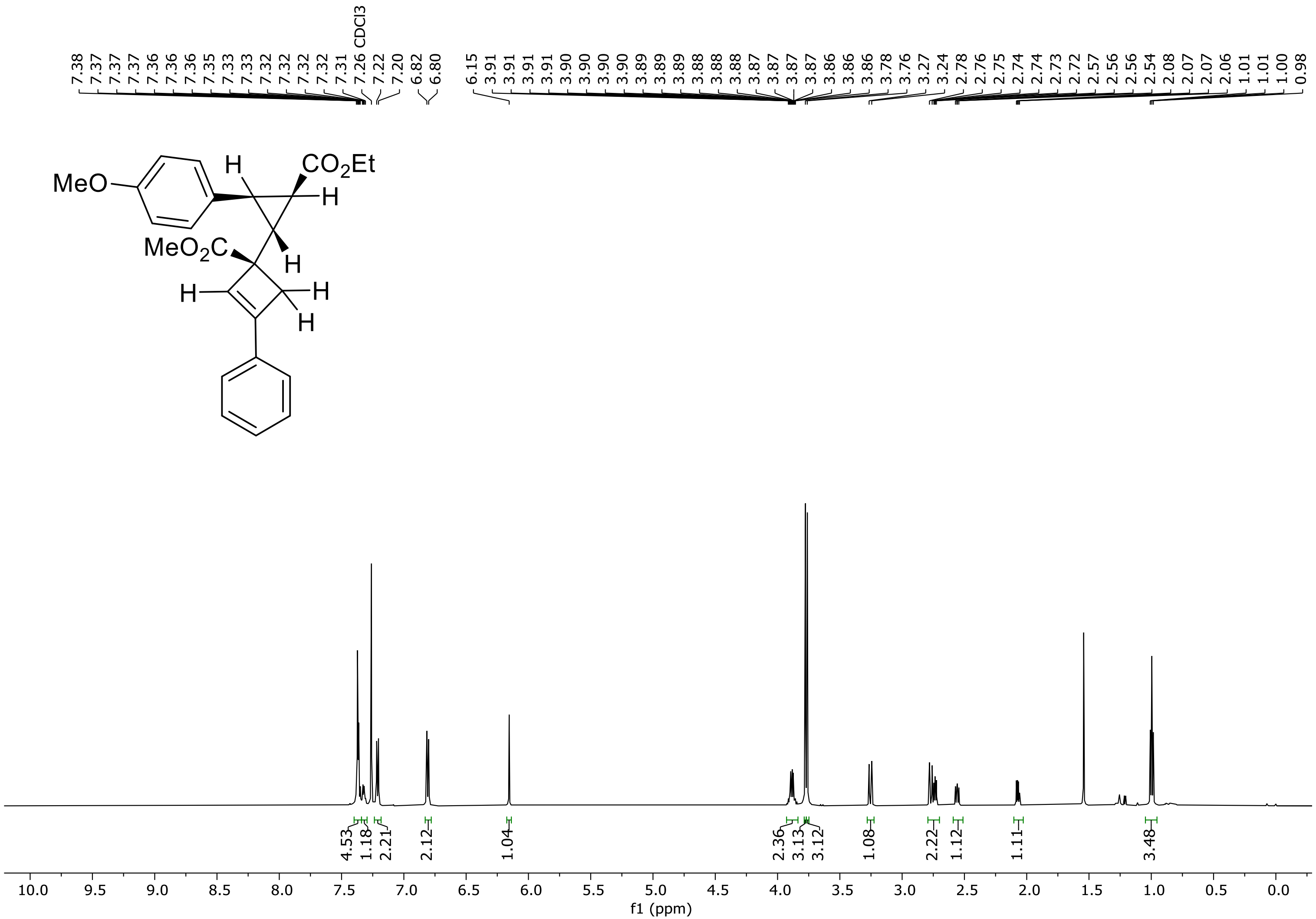

^{13}C NMR (151 MHz, $CDCl_3$, 298 K) spectrum of **3k**.

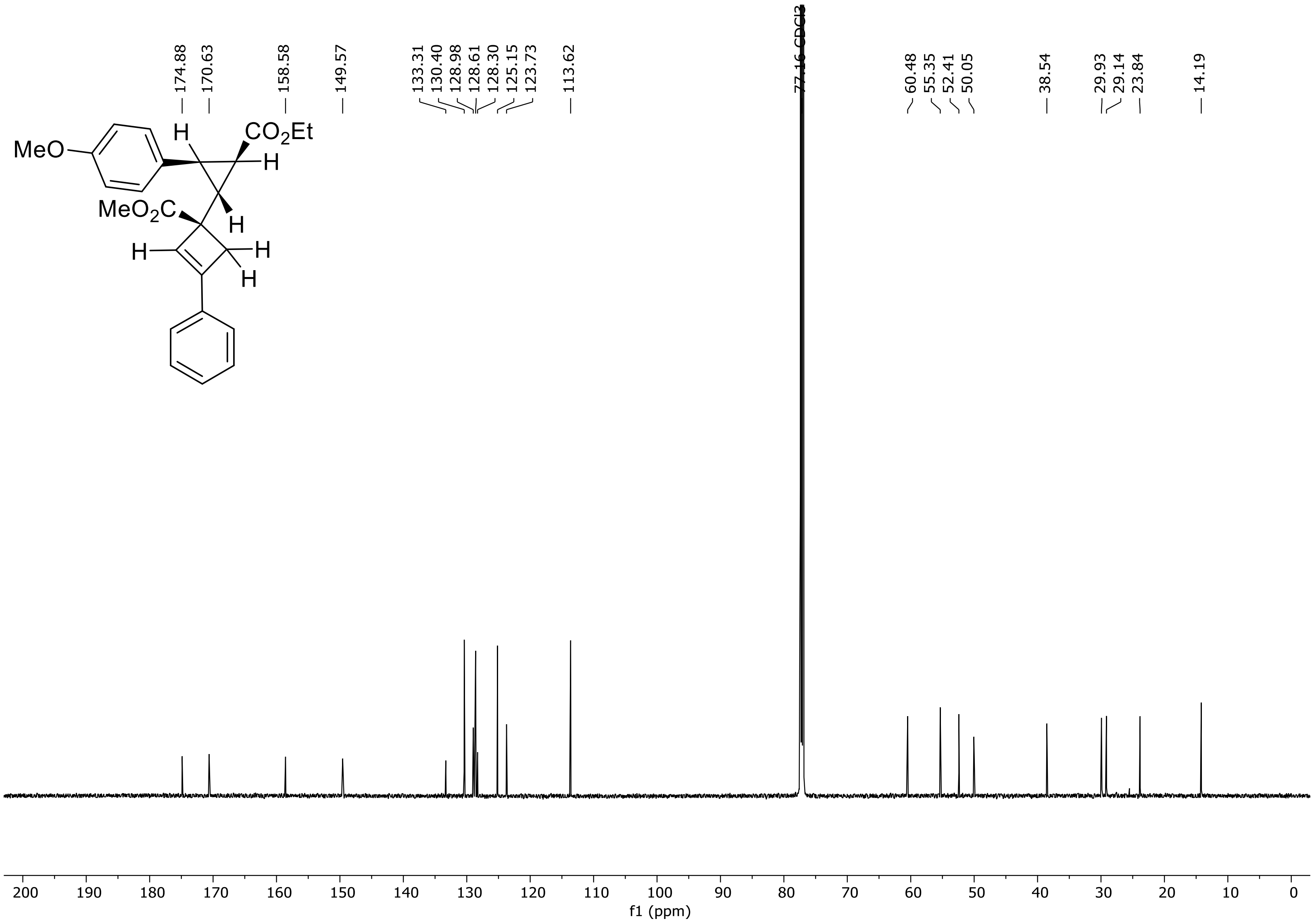

^{1}H NMR (500 MHz, $CDCl_3$, 298 K) spectrum of **3l**.

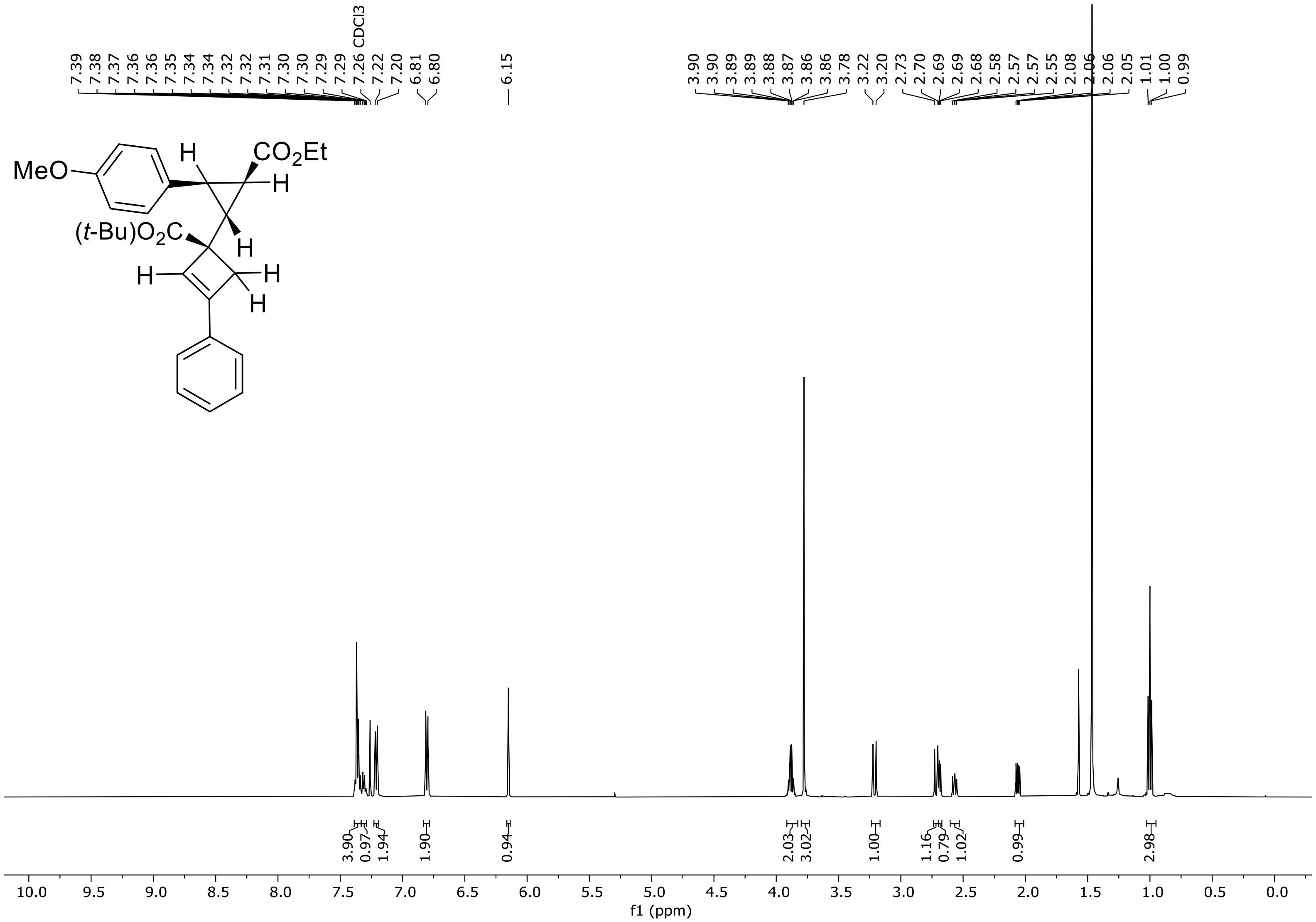

^{13}C NMR (126 MHz, $CDCl_3$, 298 K) spectrum of **3l**.

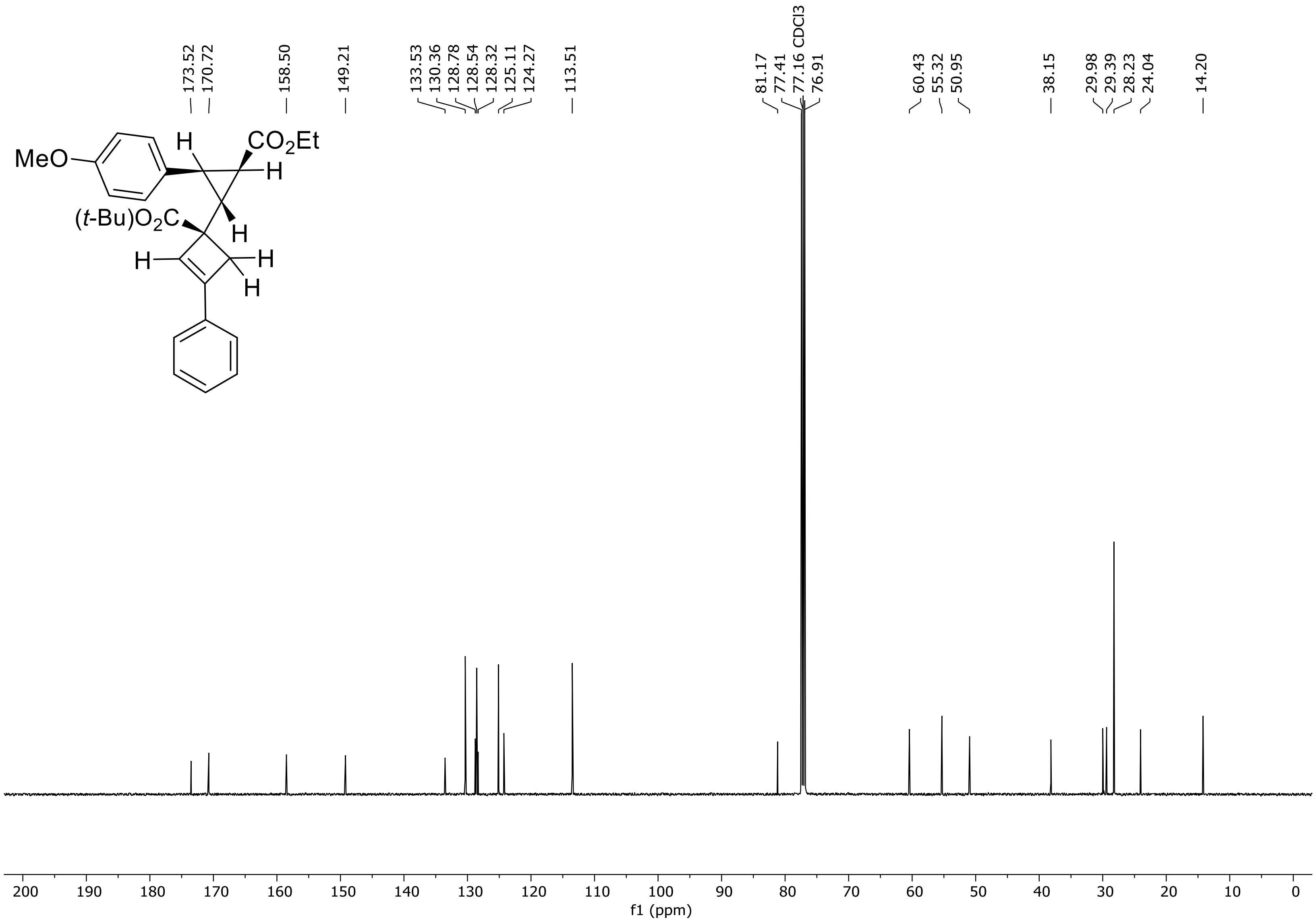

^{1}H NMR (600 MHz, $CDCl_3$, 298 K) spectrum of **3m**.

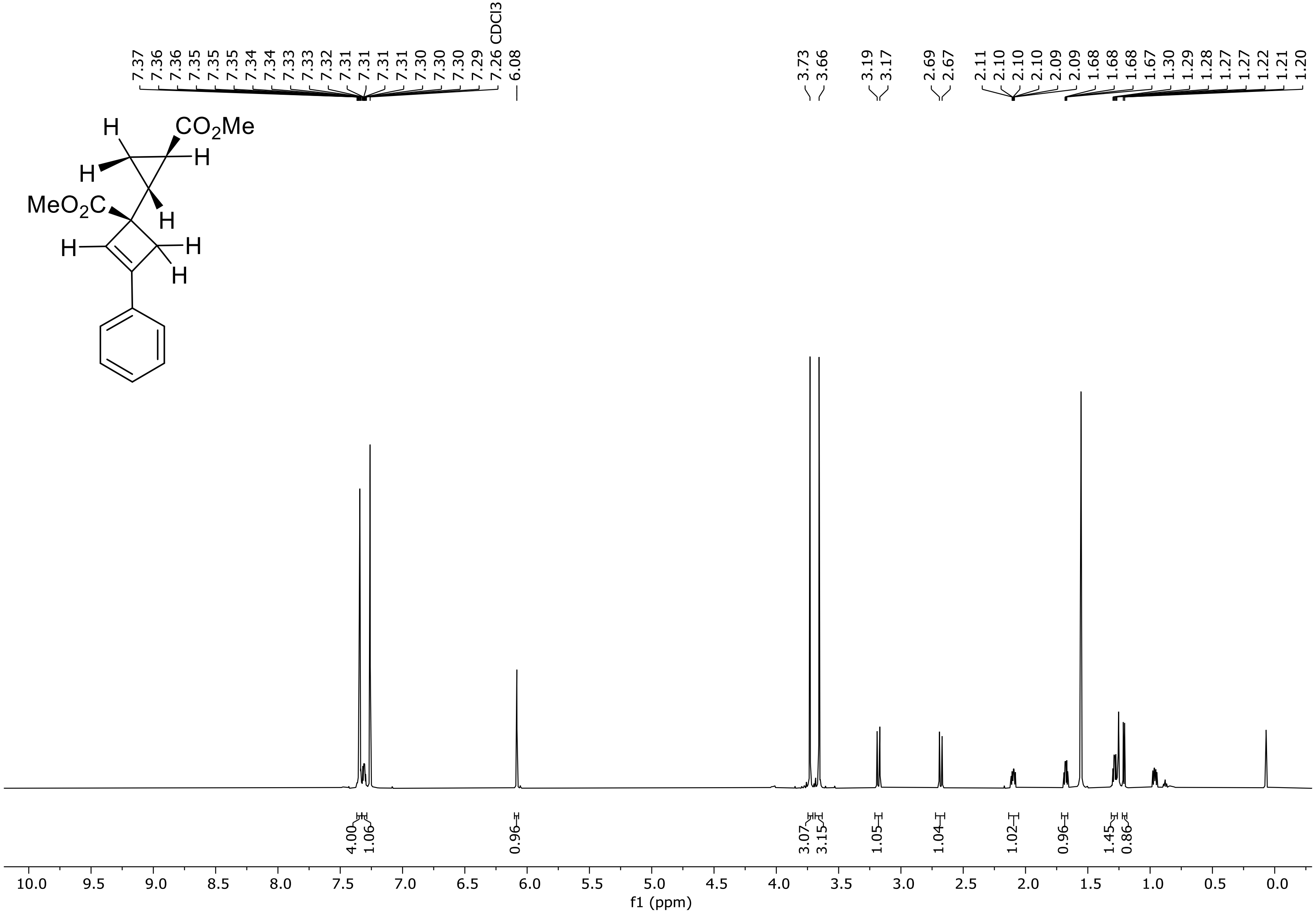

^{13}C NMR (151 MHz, $CDCl_3$, 298 K) spectrum of **3m**.

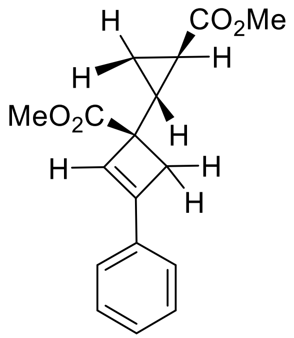

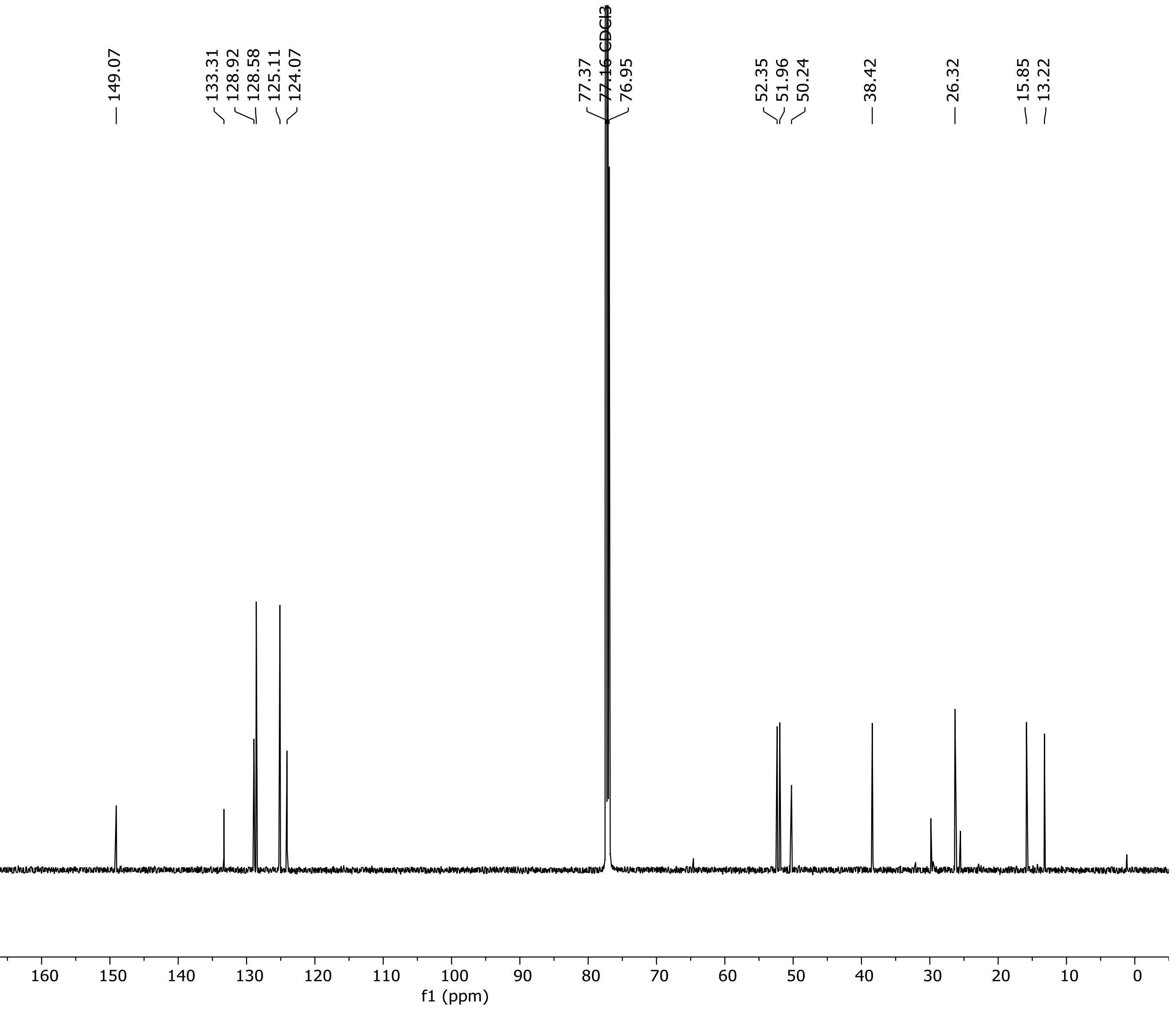

^{1}H NMR (500 MHz, $CDCl_3$, 298 K) spectrum of **3n**.

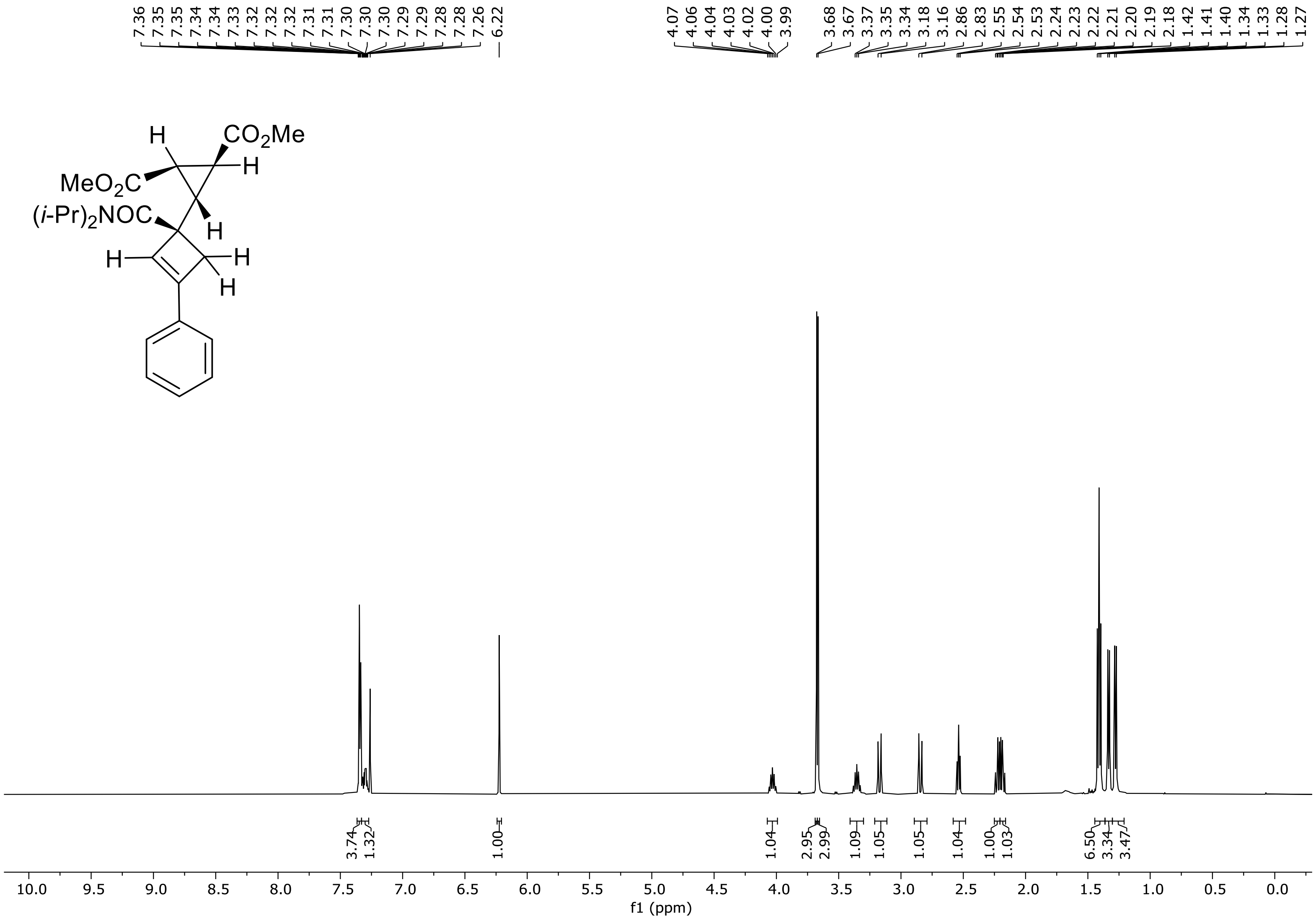

^{13}C NMR (126 MHz, $CDCl_3$, 298 K) spectrum of **3n**.

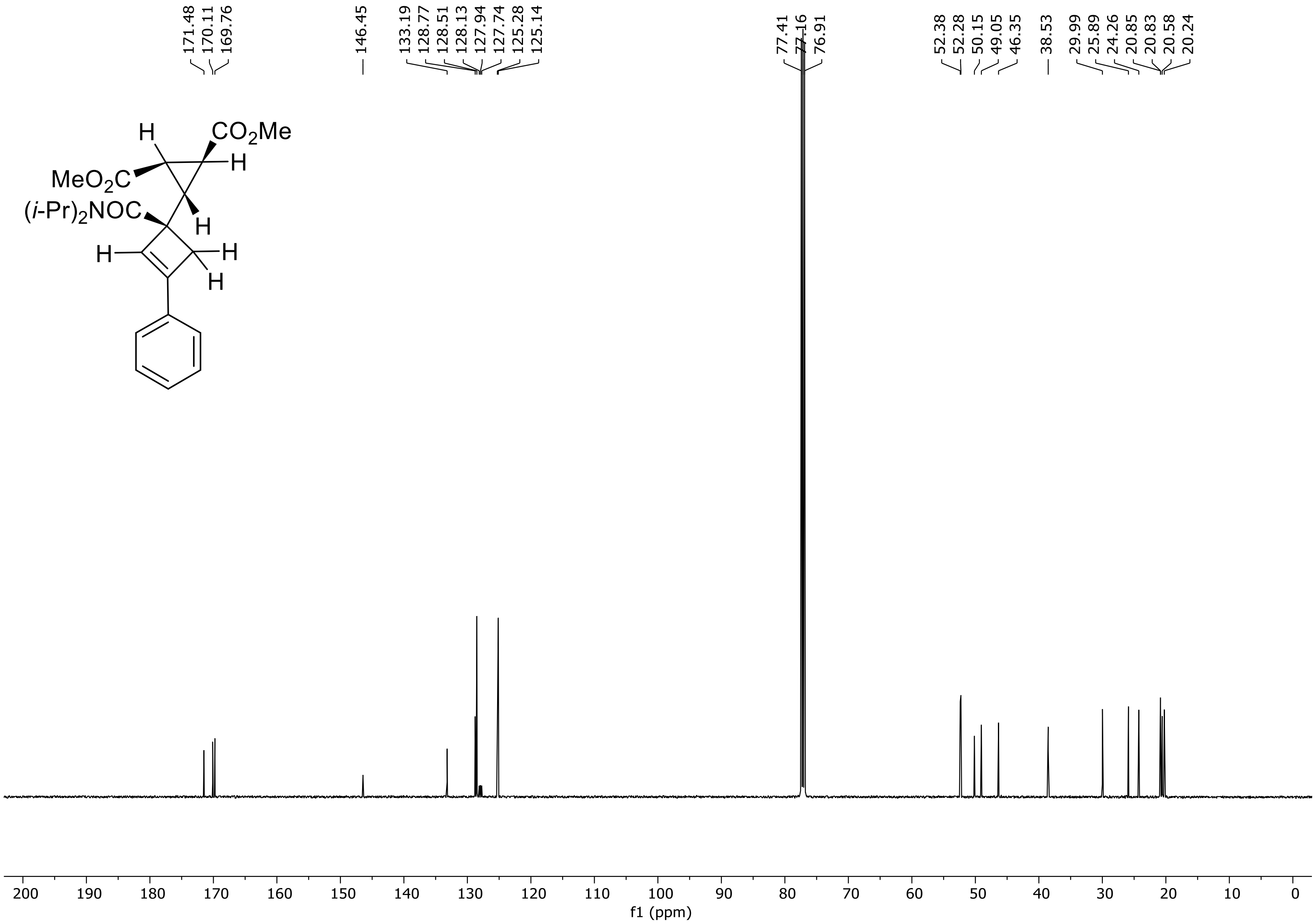

^1H NMR (600 MHz, $CDCl_3$, 298 K) spectrum of **3o**.

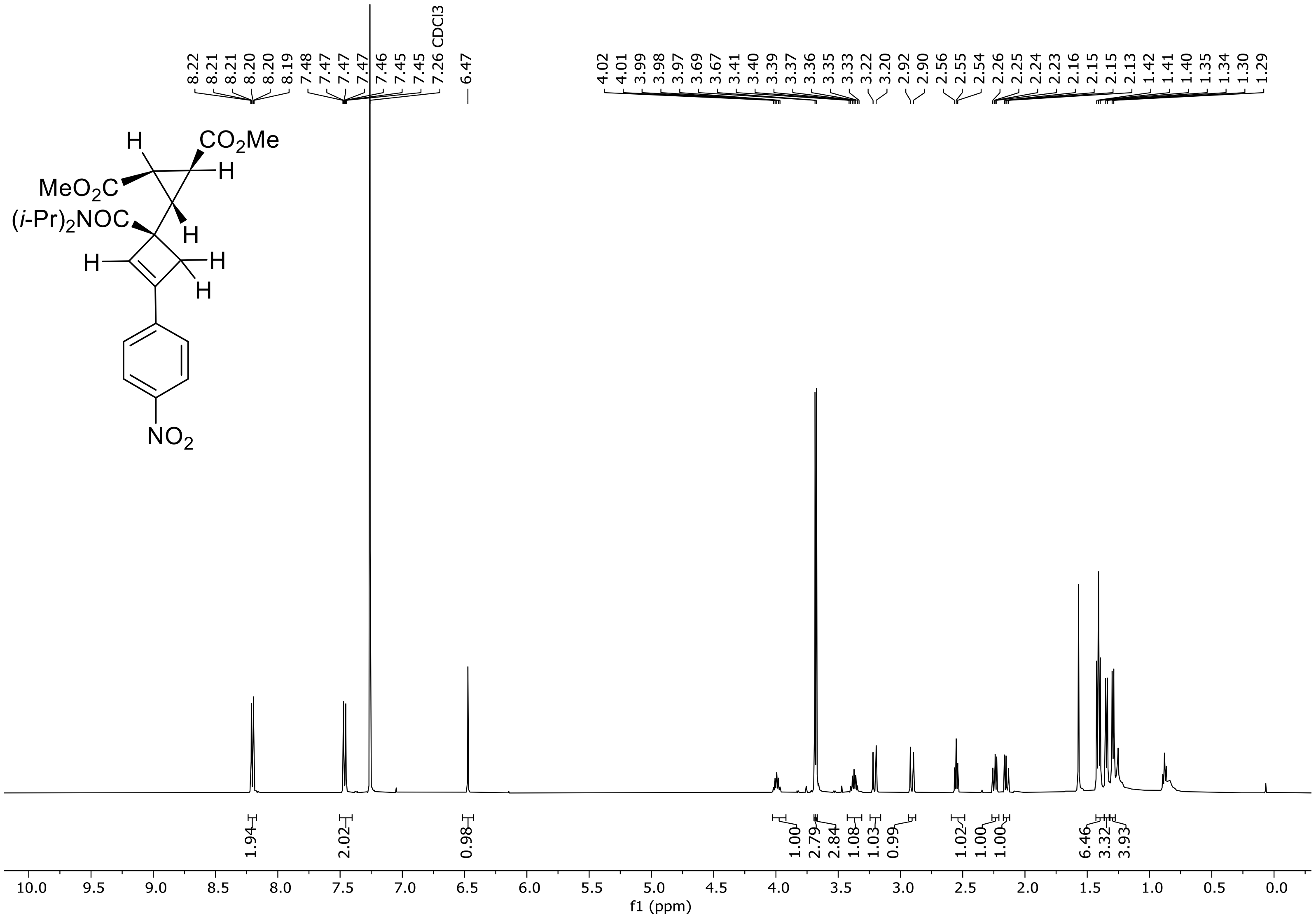

^{13}C NMR (151 MHz, $CDCl_3$, 298 K) spectrum of **3o**.

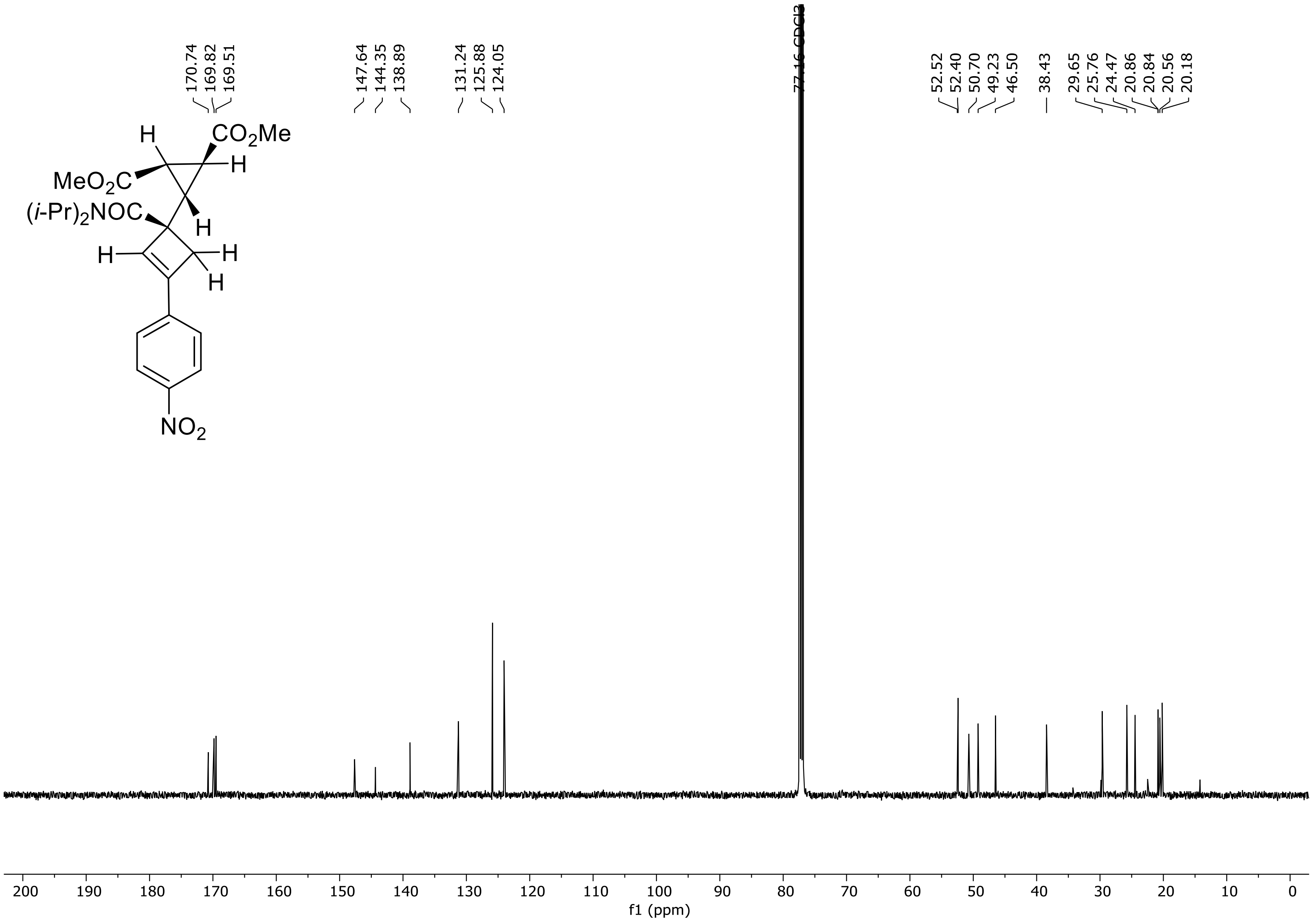

^{1}H NMR (500 MHz, $CDCl_3$, 298 K) spectrum of **3p**.

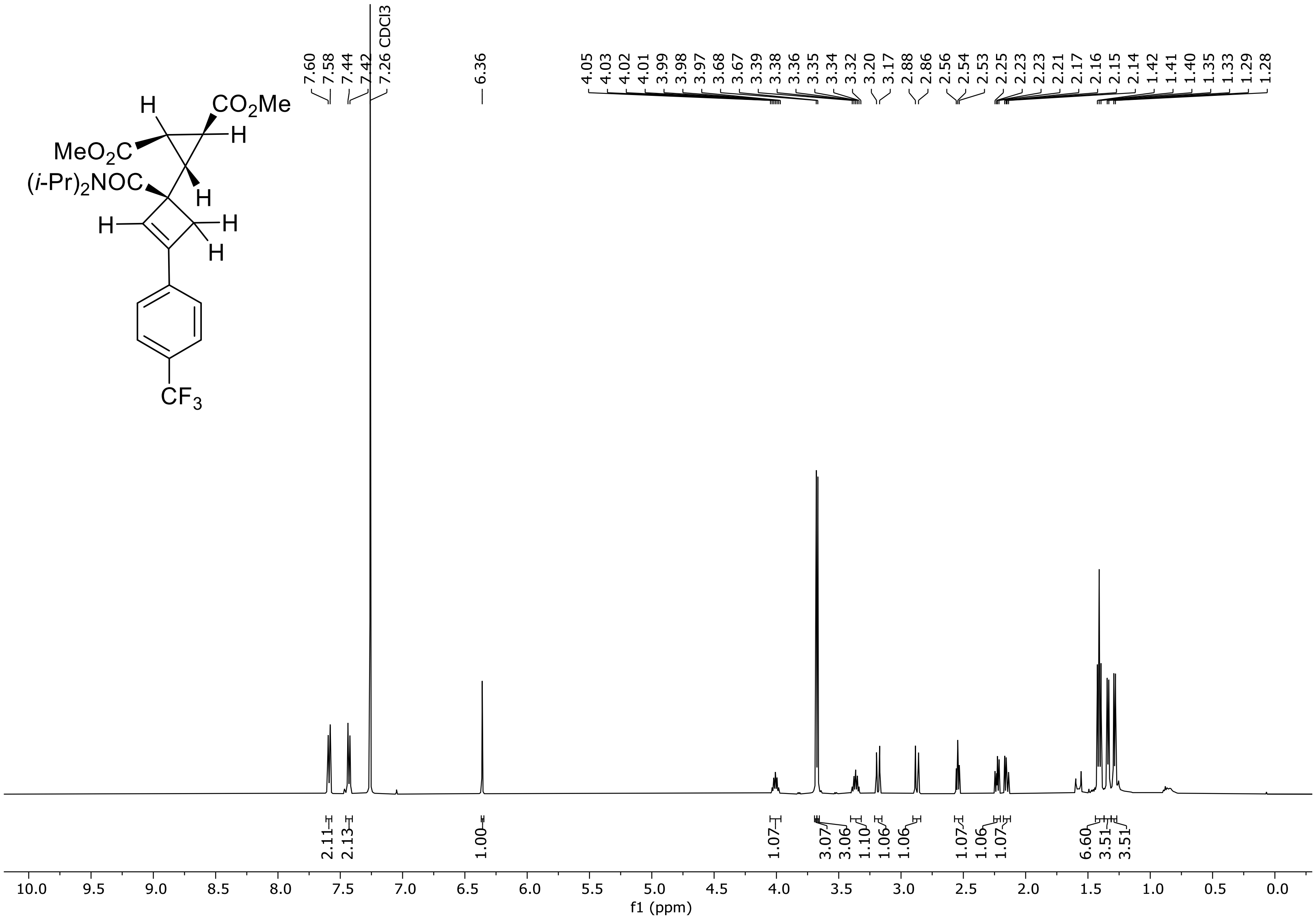

^{13}C NMR (126 MHz, $CDCl_3$, 298 K) spectrum of **3p**.

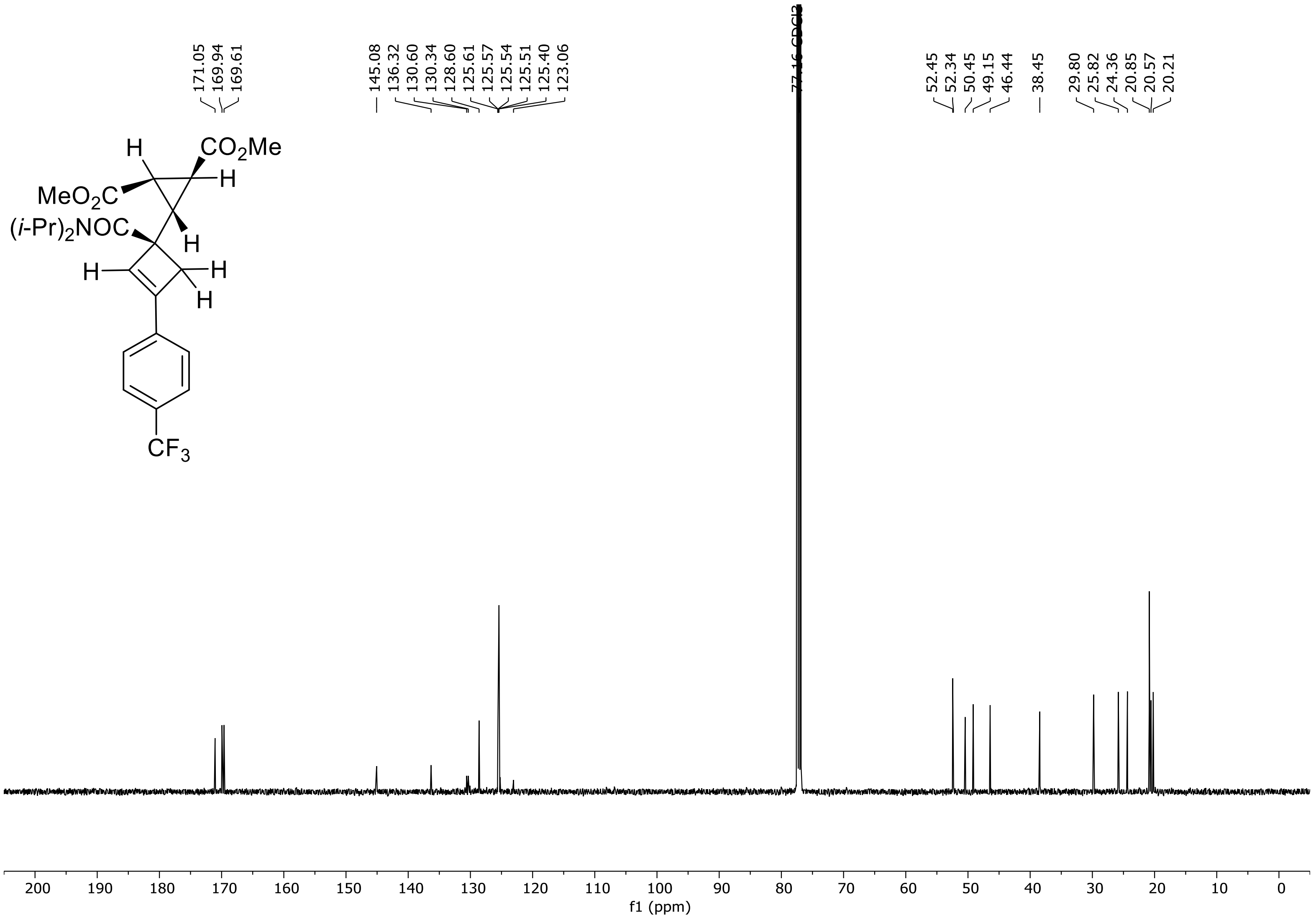

^{19}F NMR (471 MHz, $CDCl_3$, 298 K) spectrum of **3p**.

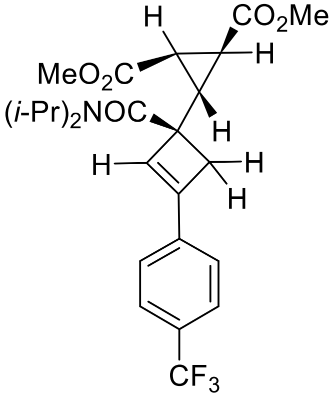

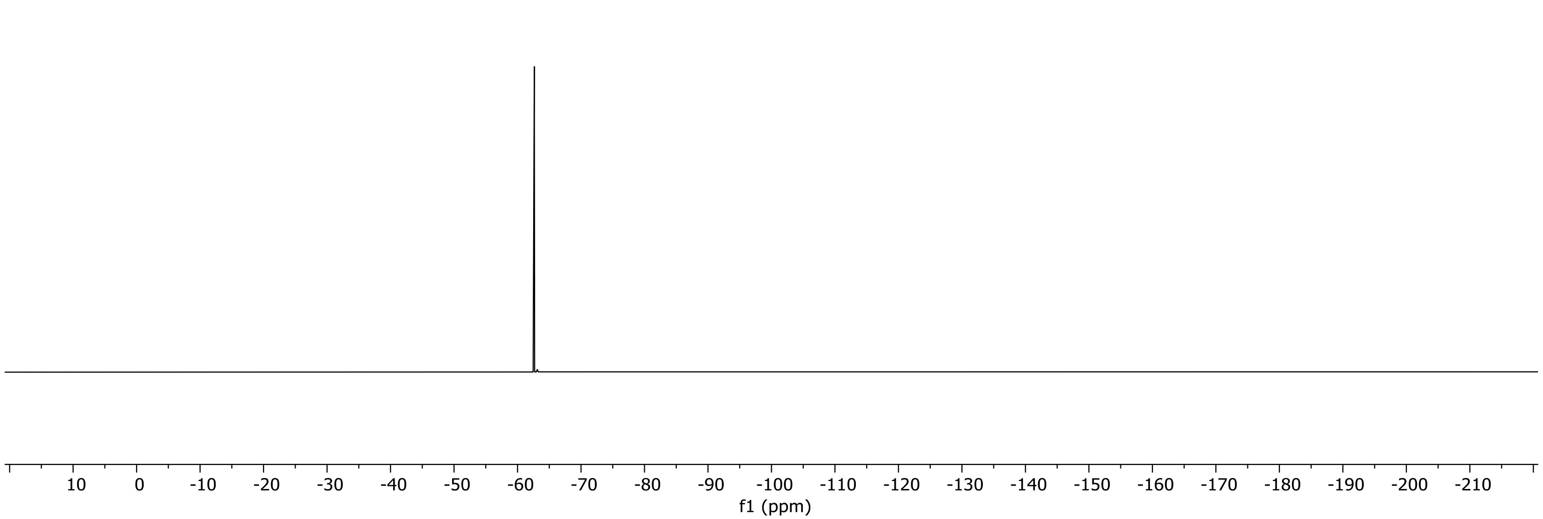

^{1}H NMR (500 MHz, $CDCl_3$, 298 K) spectrum of **3q**.

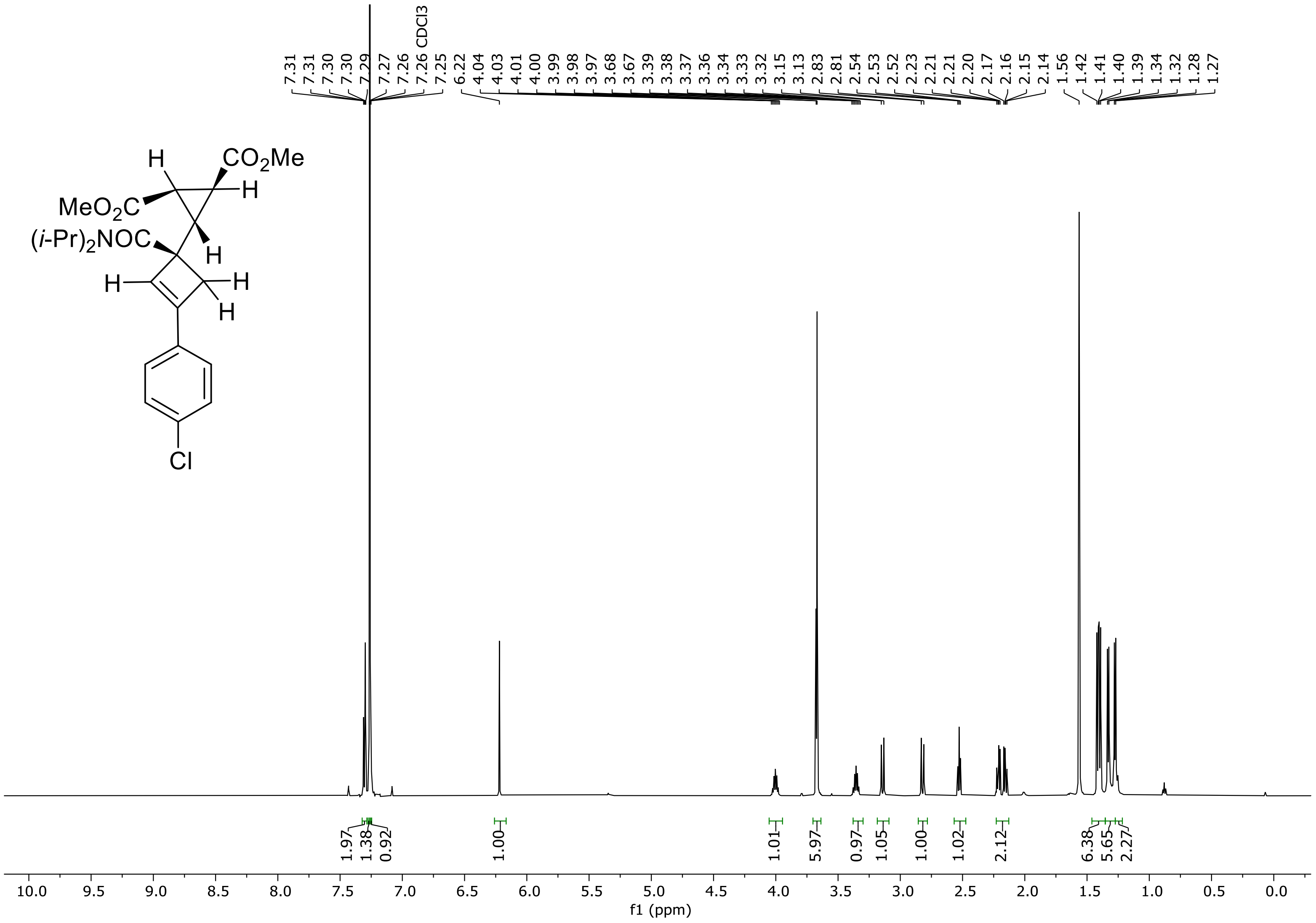

^{13}C NMR (126 MHz, $CDCl_3$, 298 K) spectrum of **3q**.

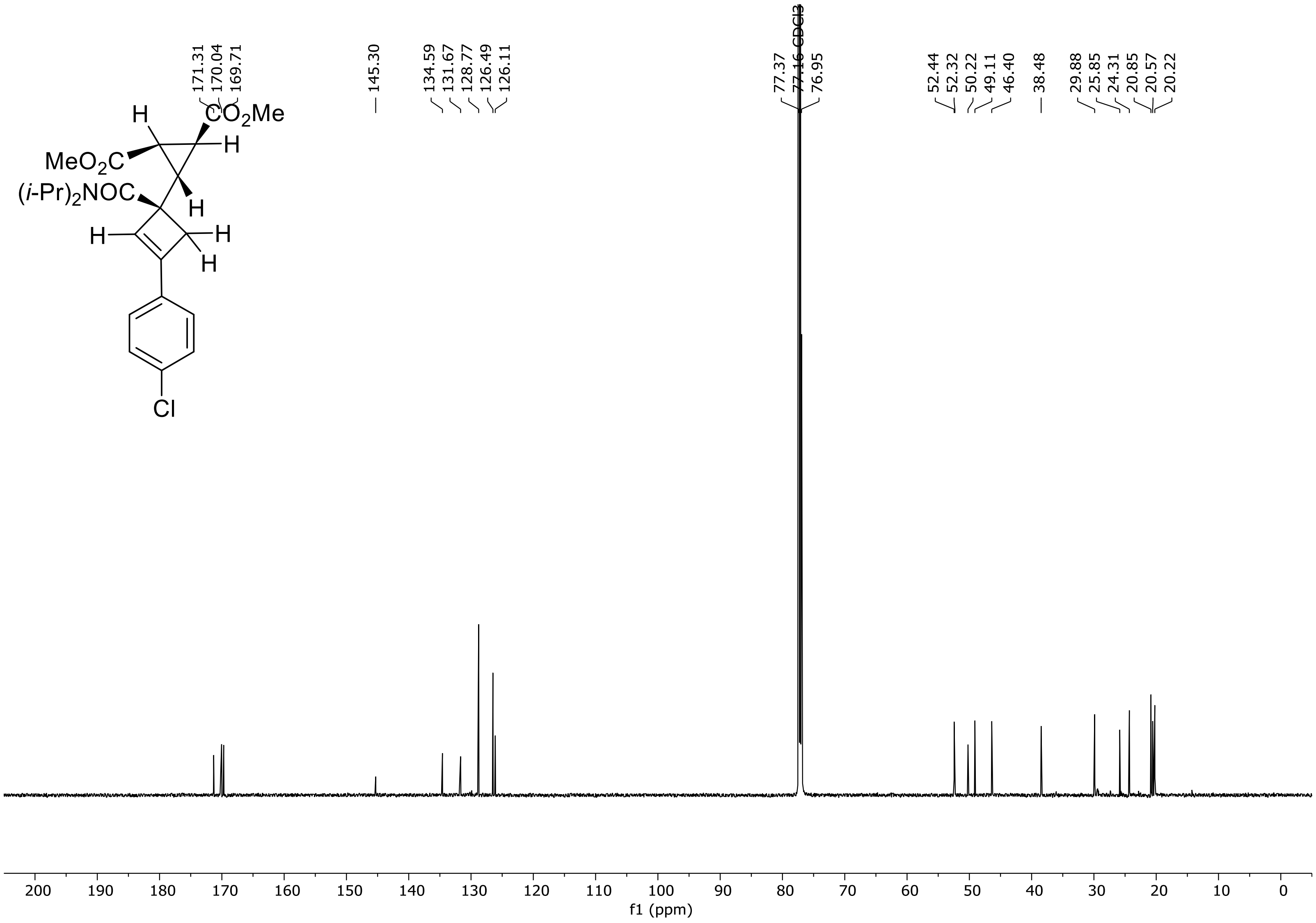

^{1}H NMR (500 MHz, $CDCl_3$, 298 K) spectrum of **3r**.

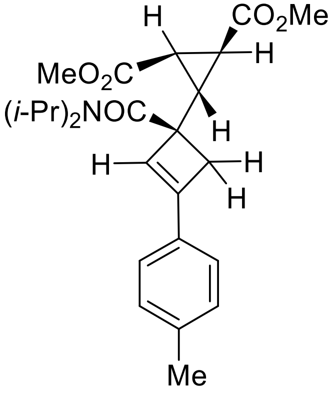

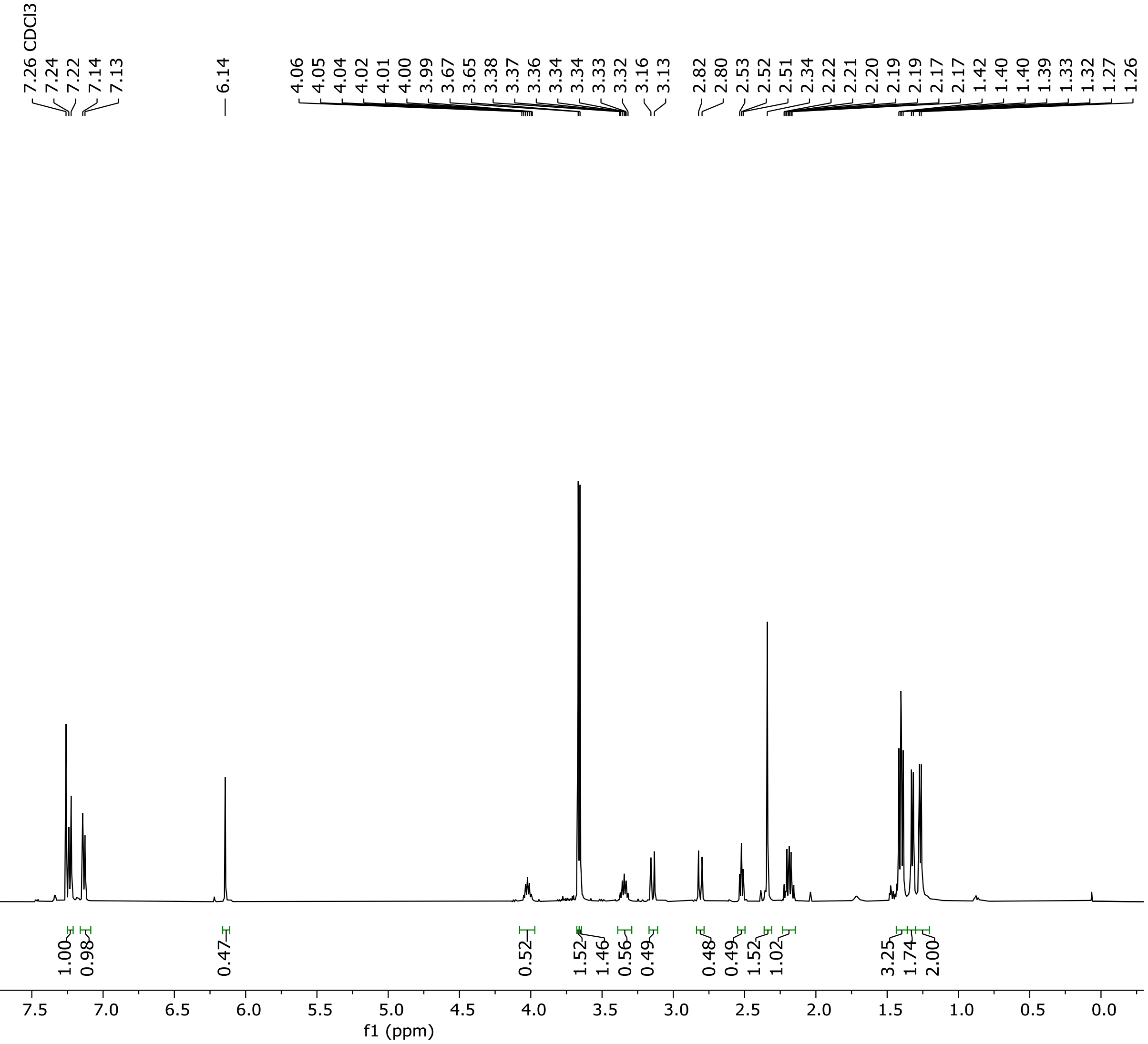

^{13}C NMR (126 MHz, $CDCl_3$, 298 K) spectrum of **3r**.

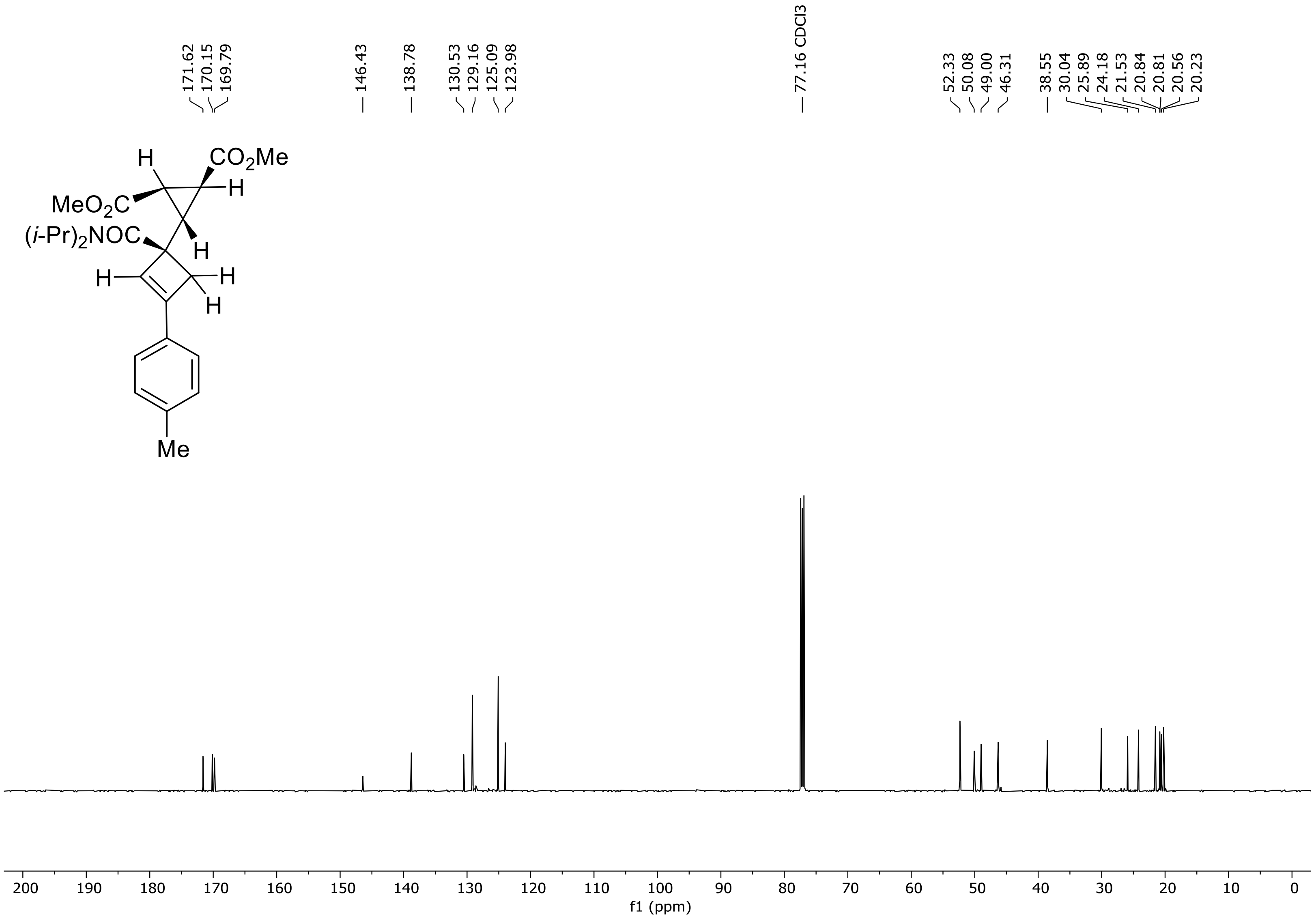

^1H NMR (500 MHz, $CDCl_3$, 298 K) spectrum of **3s**.

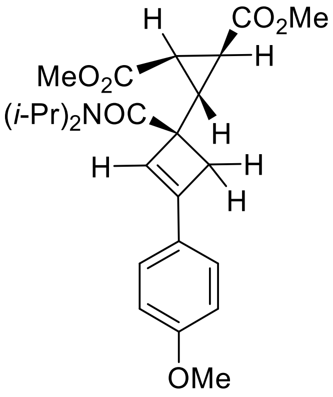

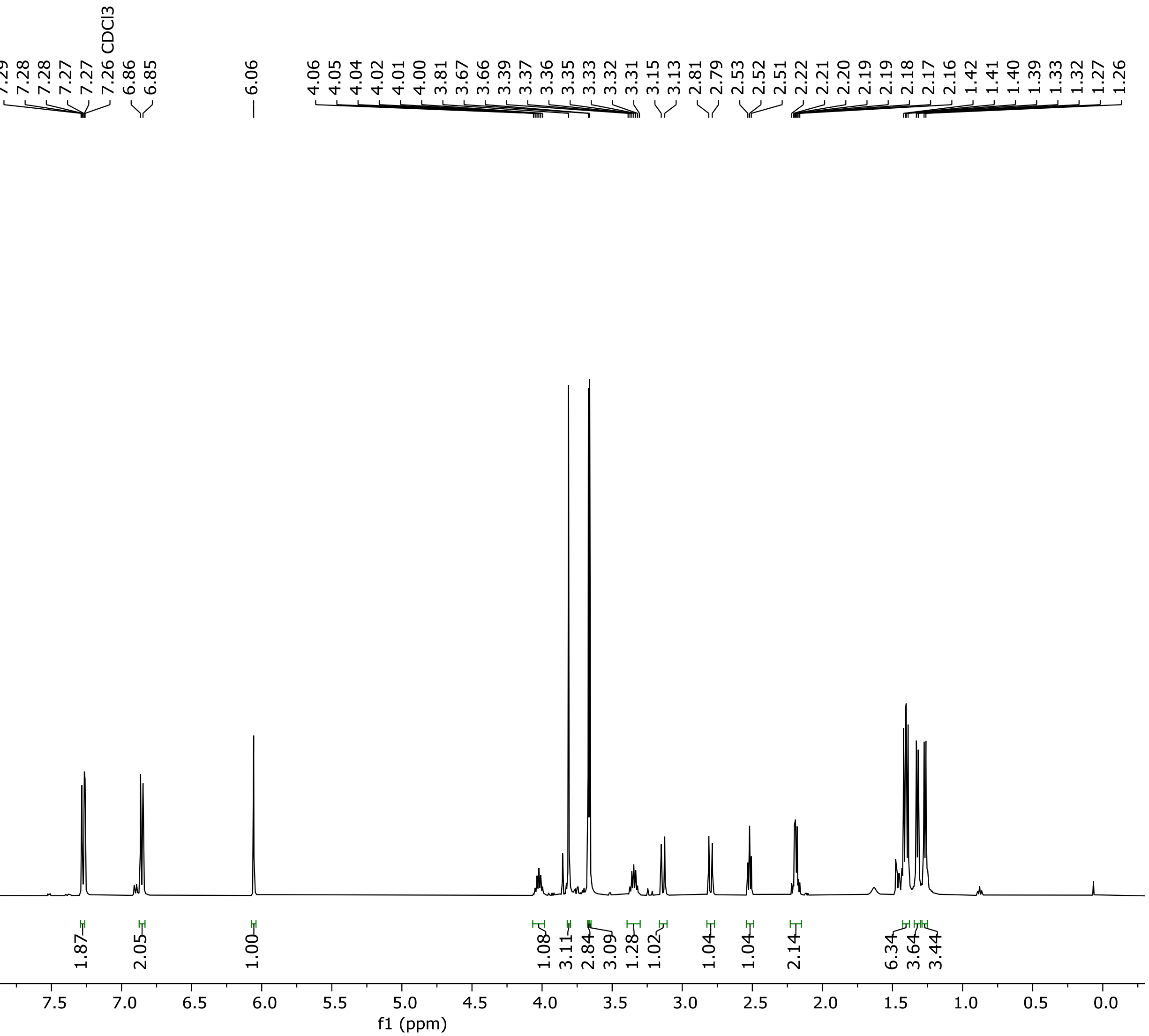

^{13}C NMR (126 MHz, $CDCl_3$, 298 K) spectrum of **3s**.

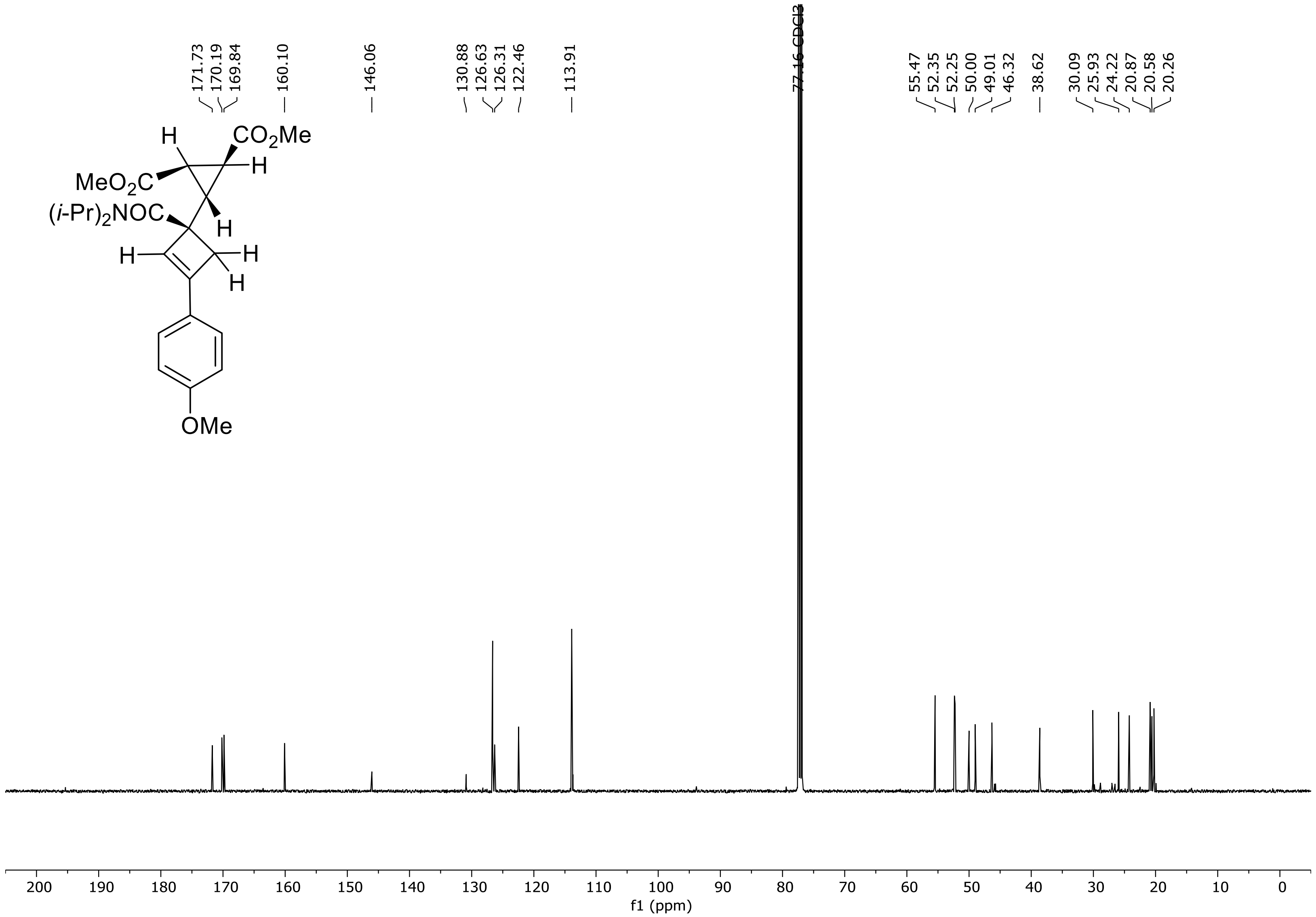

^{1}H NMR (500 MHz, $CDCl_3$, 298 K) spectrum of **3t**.

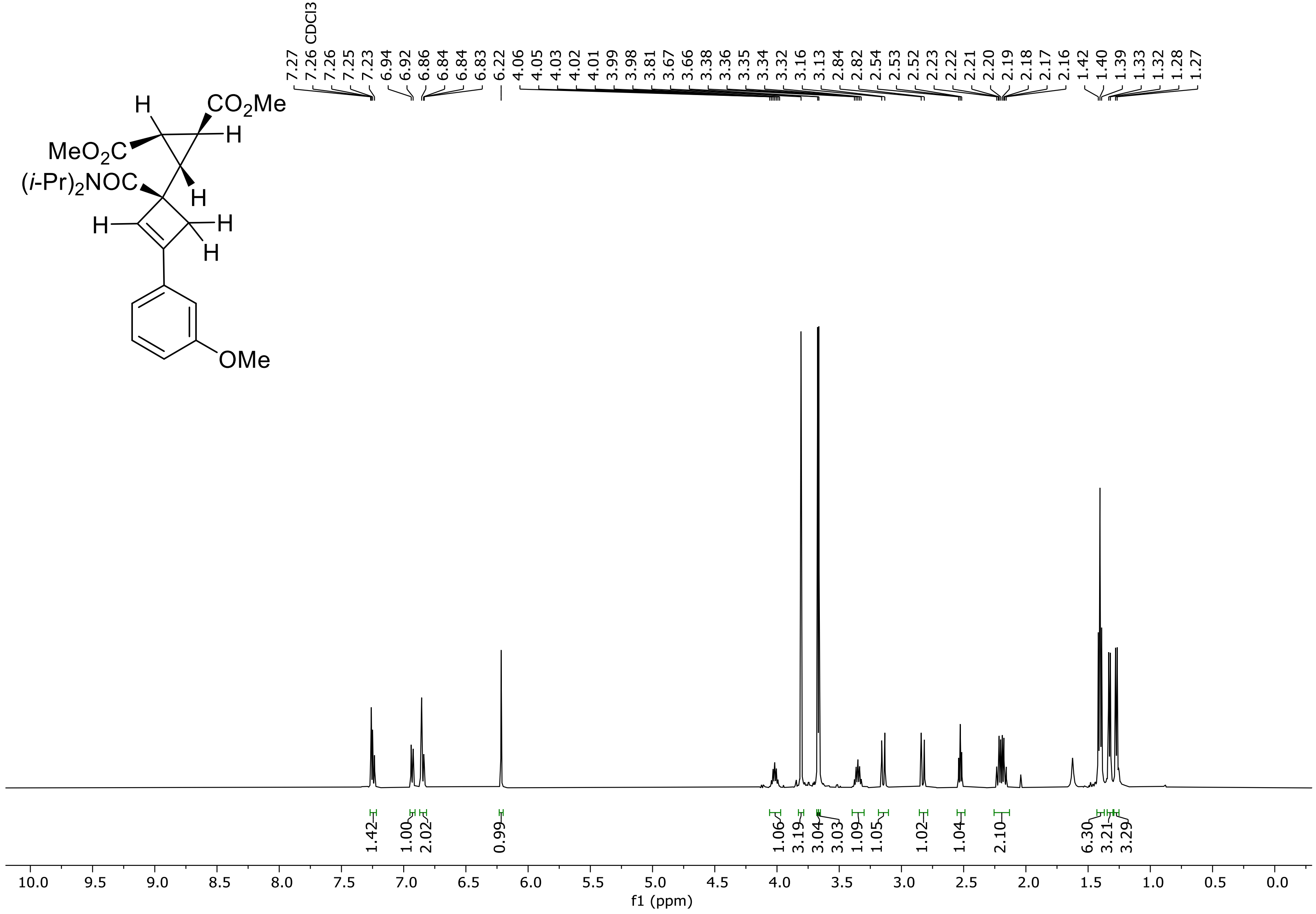

^{13}C NMR (126 MHz, $CDCl_3$, 298 K) spectrum of **3t**.

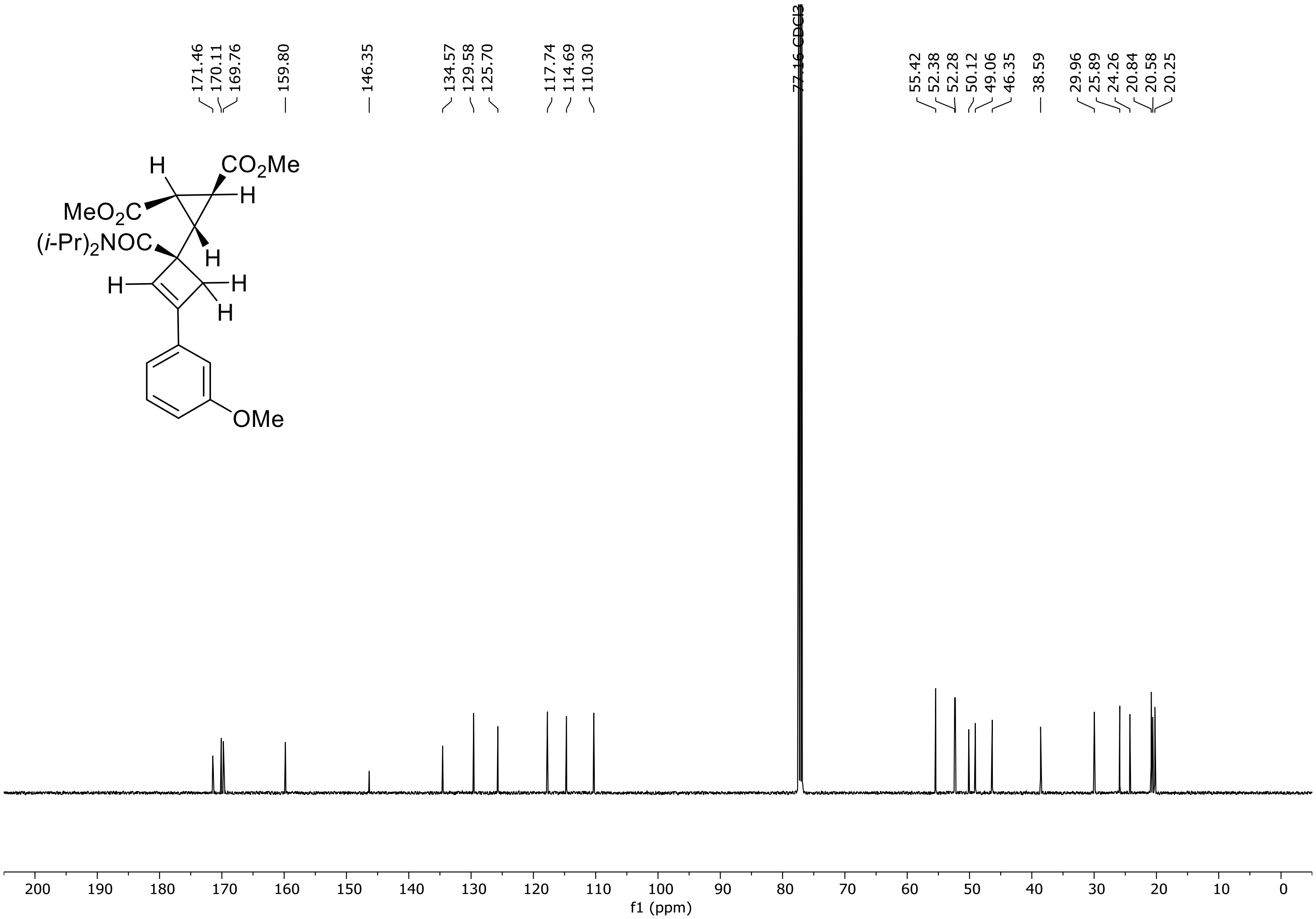

^{1}H NMR (500 MHz, $CDCl_3$, 298 K) spectrum of **3u**.

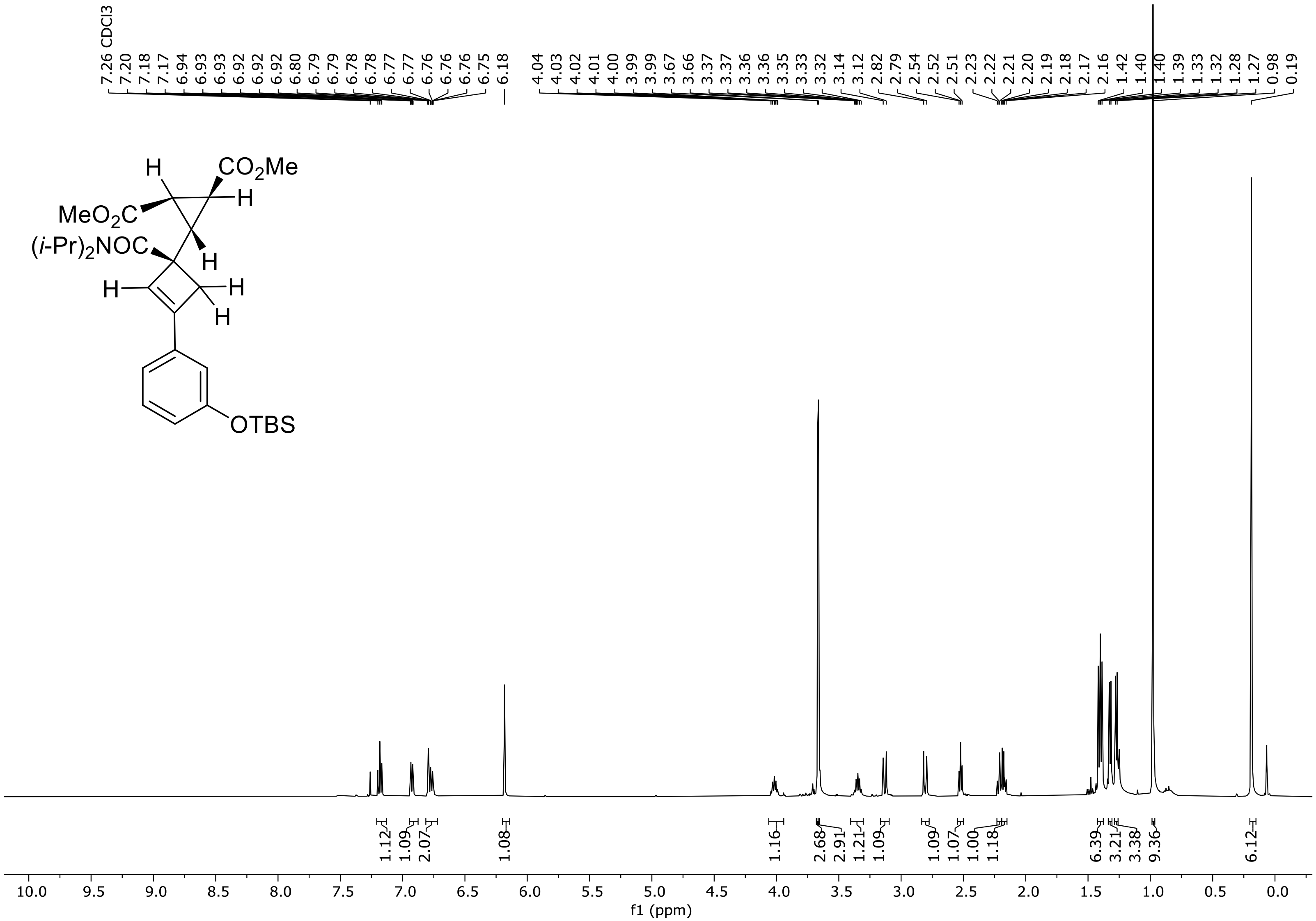

^{13}C NMR (126 MHz, $CDCl_3$, 298 K) spectrum of **3u**.

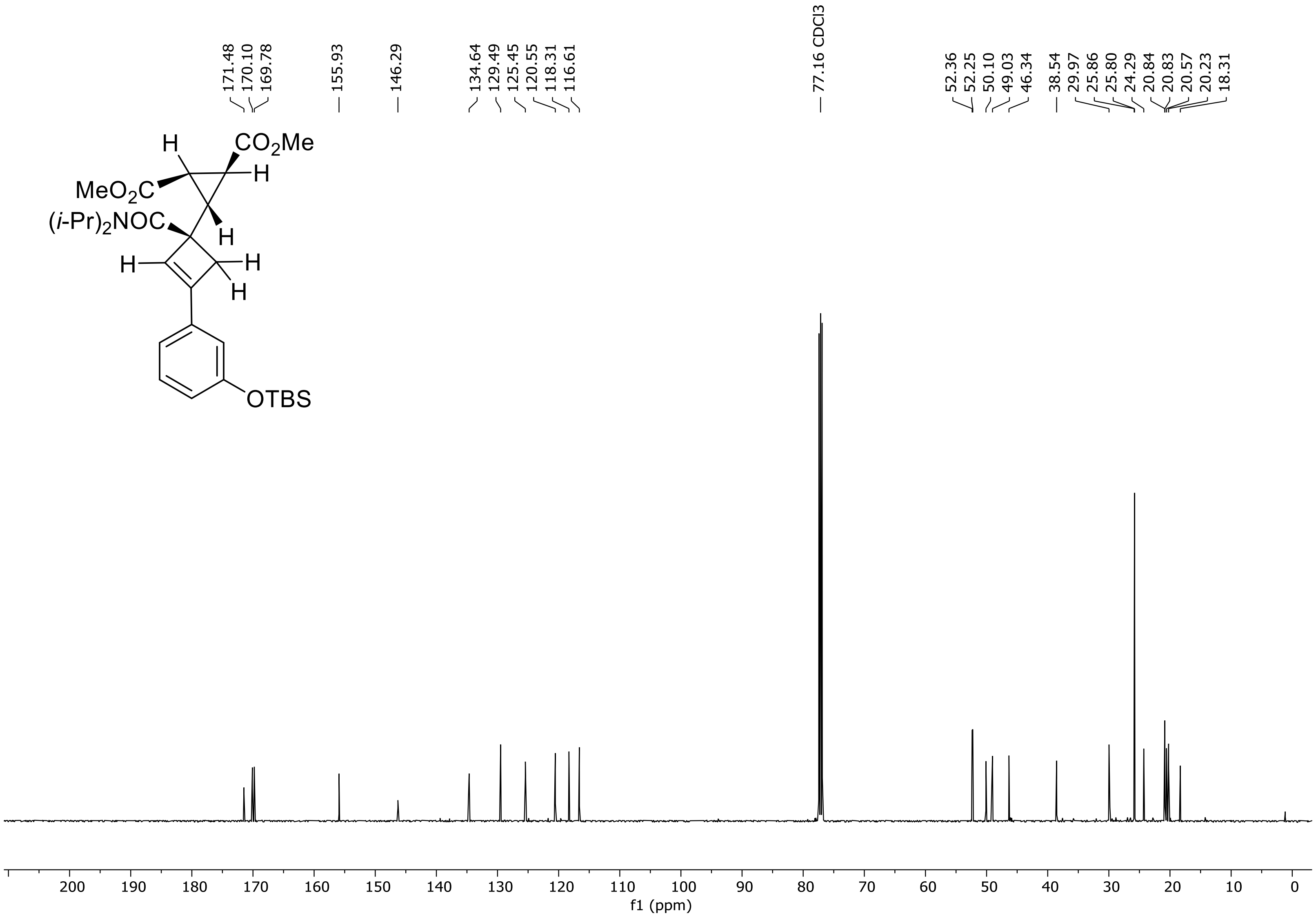

^1H NMR (500 MHz, $CDCl_3$, 298 K) spectrum of **3v**.

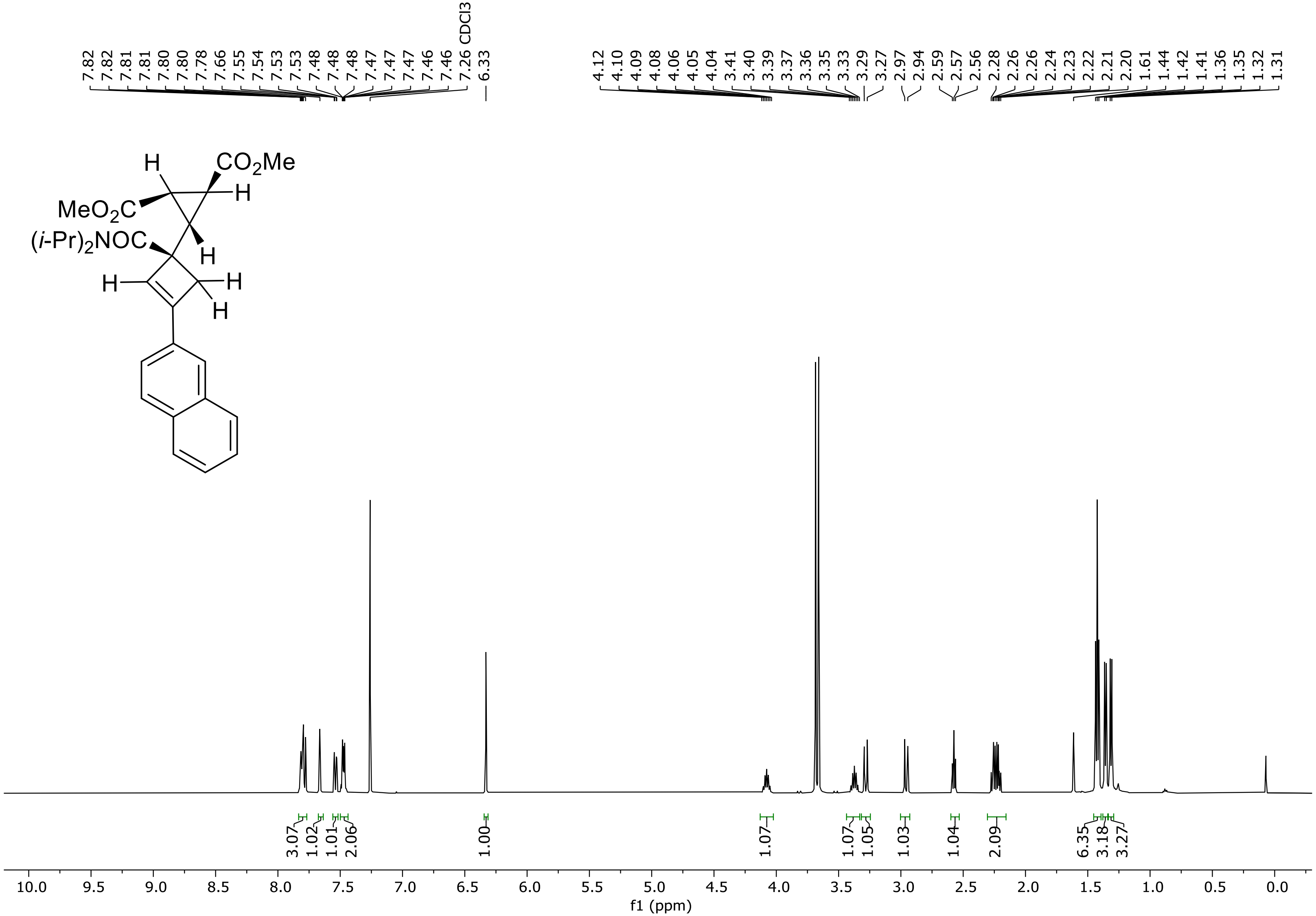

^{13}C NMR (126 MHz, $CDCl_3$, 298 K) spectrum of **3v**.

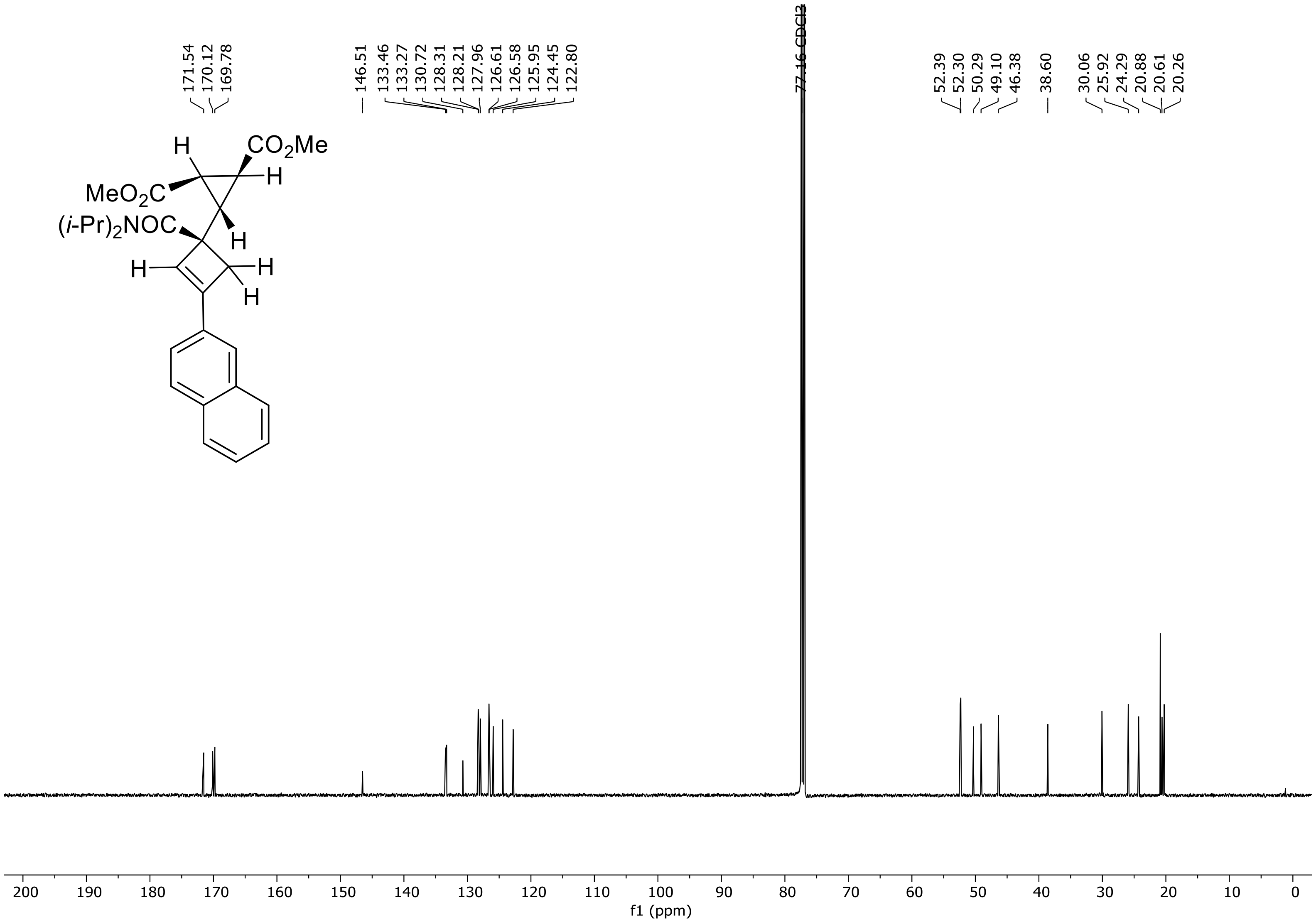

^{1}H NMR (500 MHz, $CDCl_3$, 298 K) spectrum of **3w**.

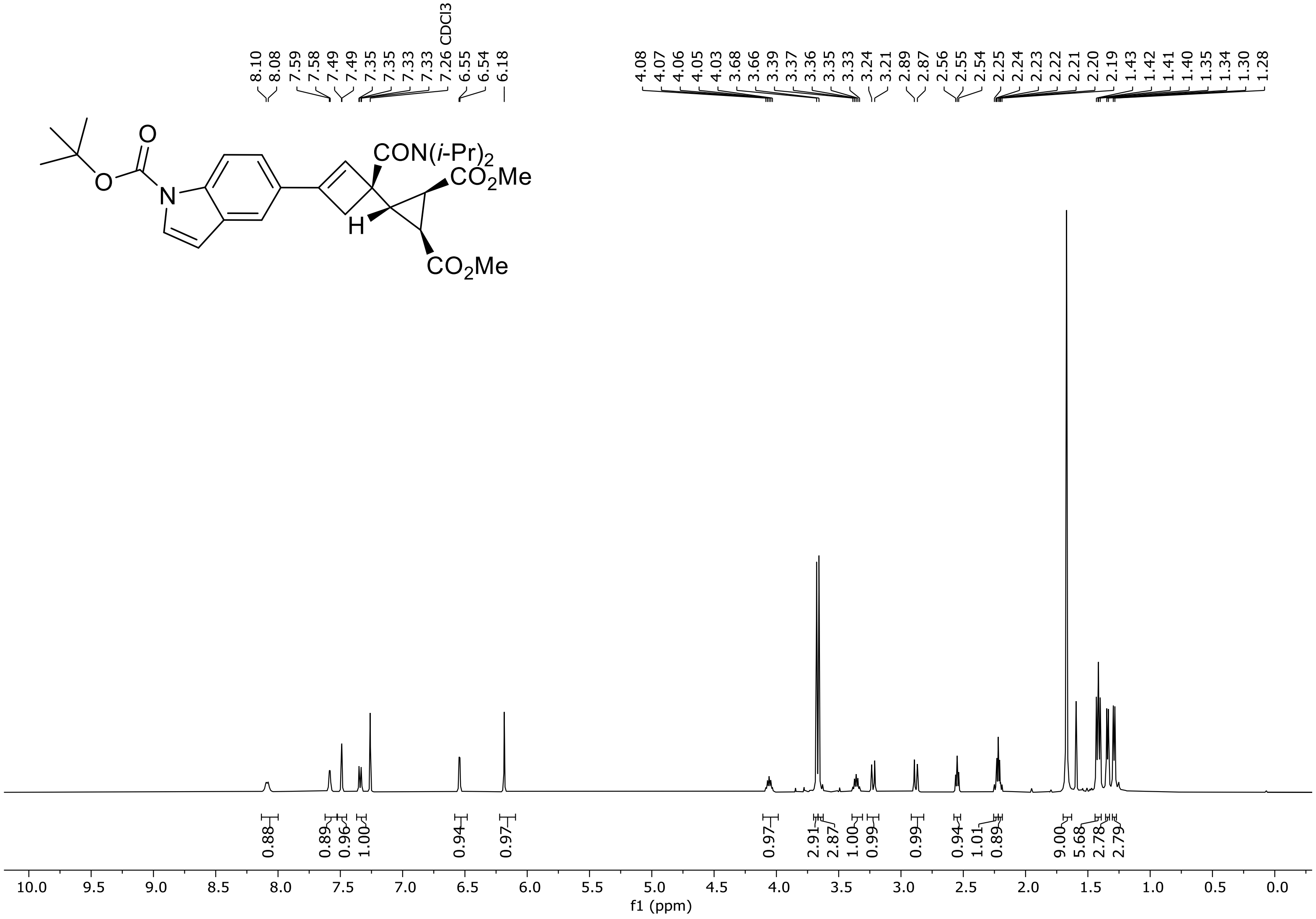

^{13}C NMR (126 MHz, $CDCl_3$, 298 K) spectrum of **3w**.

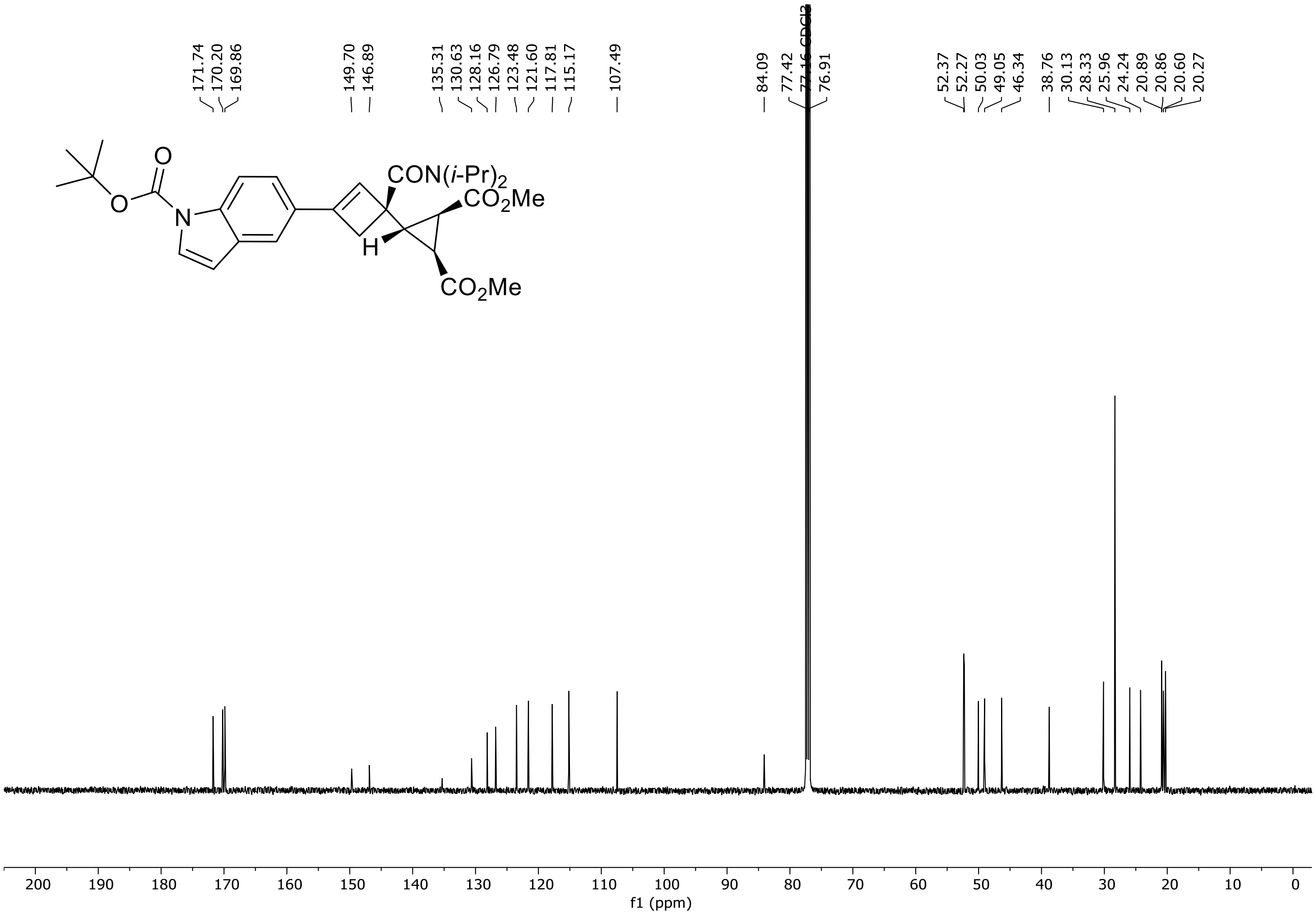

^{1}H NMR (500 MHz, $CDCl_3$, 298 K) spectrum of **3x**.

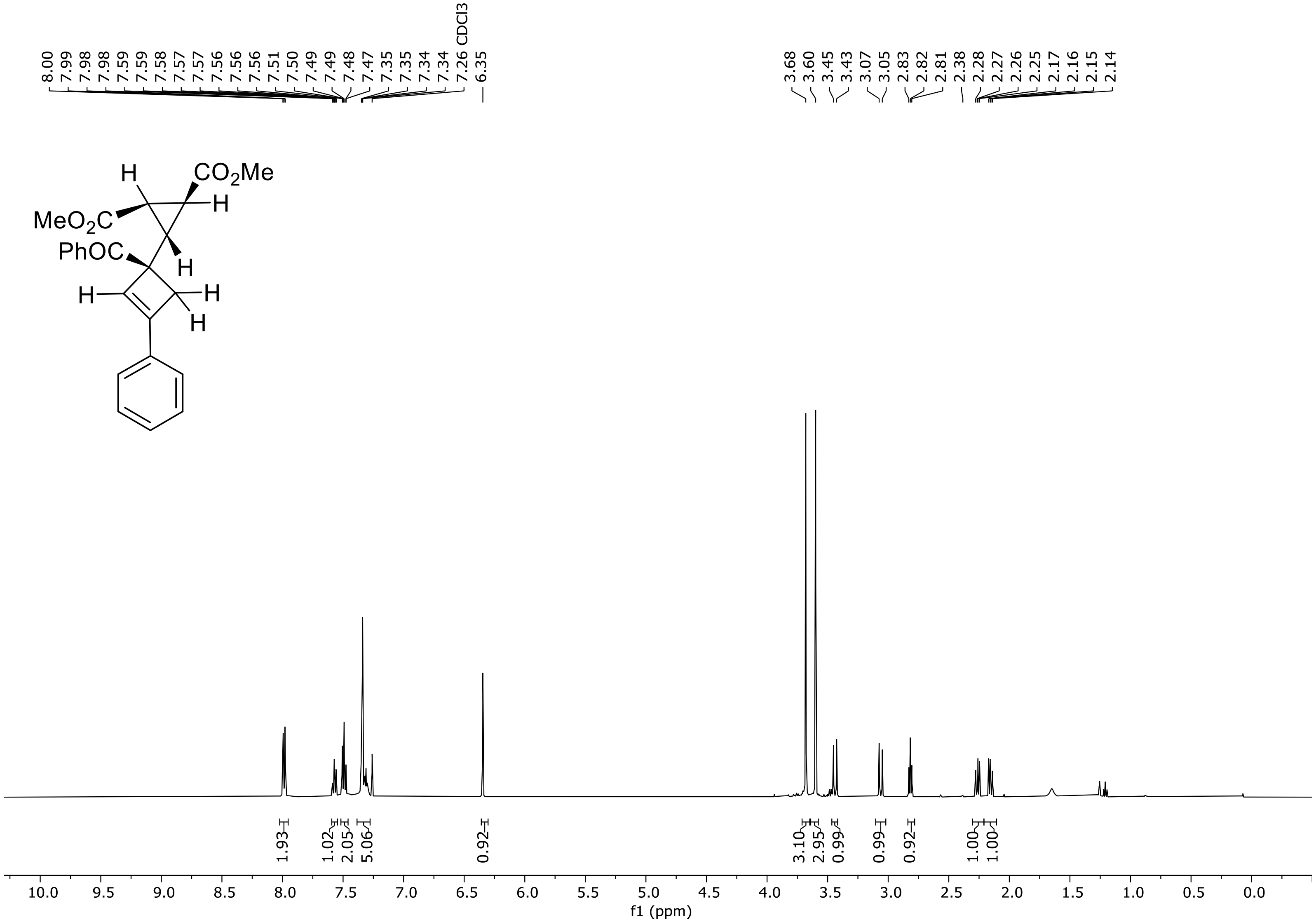

^{13}C NMR (126 MHz, $CDCl_3$, 298 K) spectrum of **3x**.

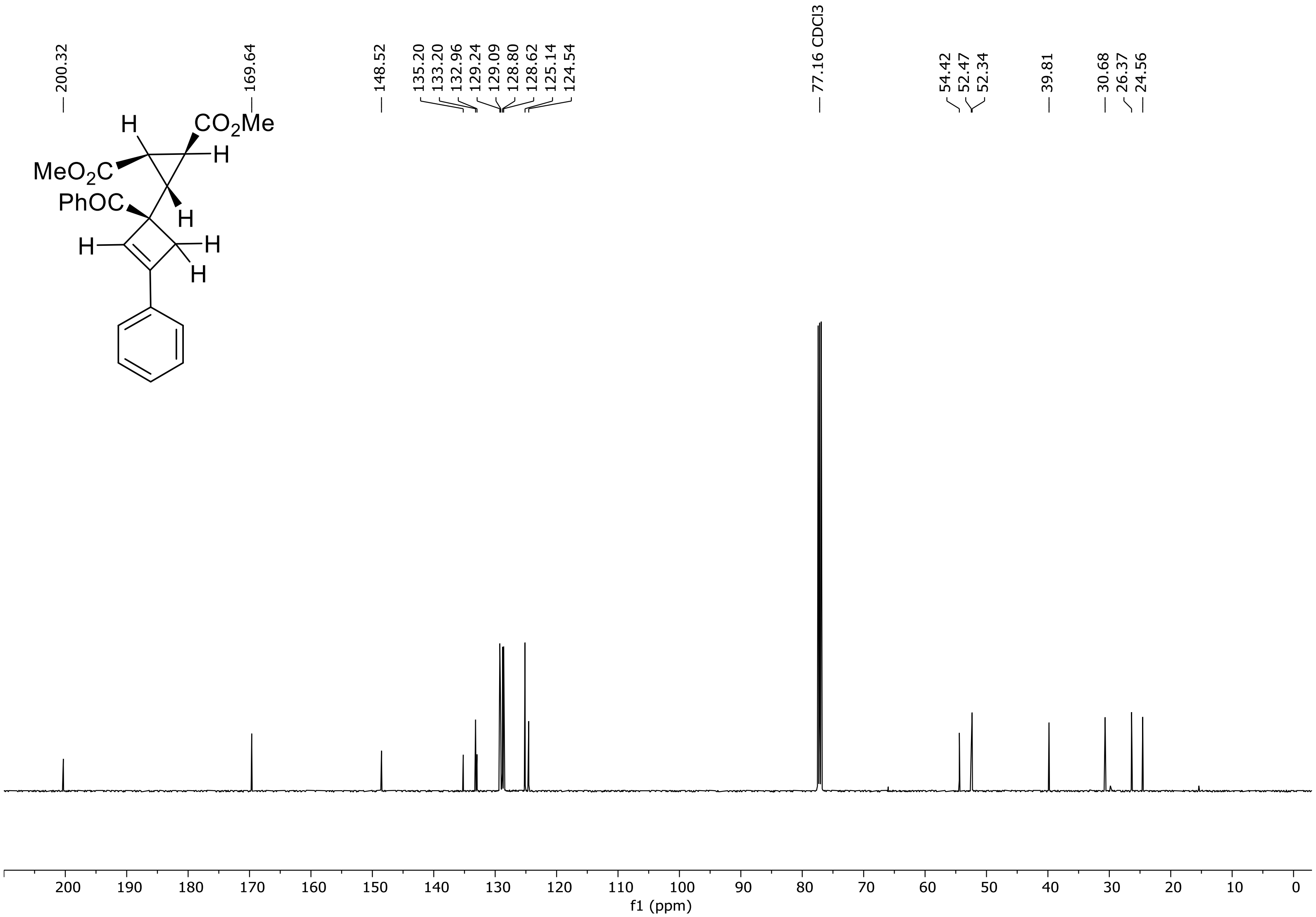

^{1}H NMR (700 MHz, $CDCl_3$, 298 K) spectrum of **3y**.

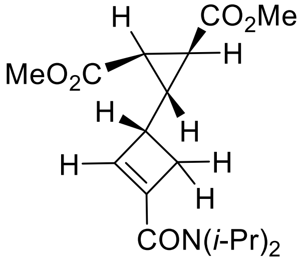

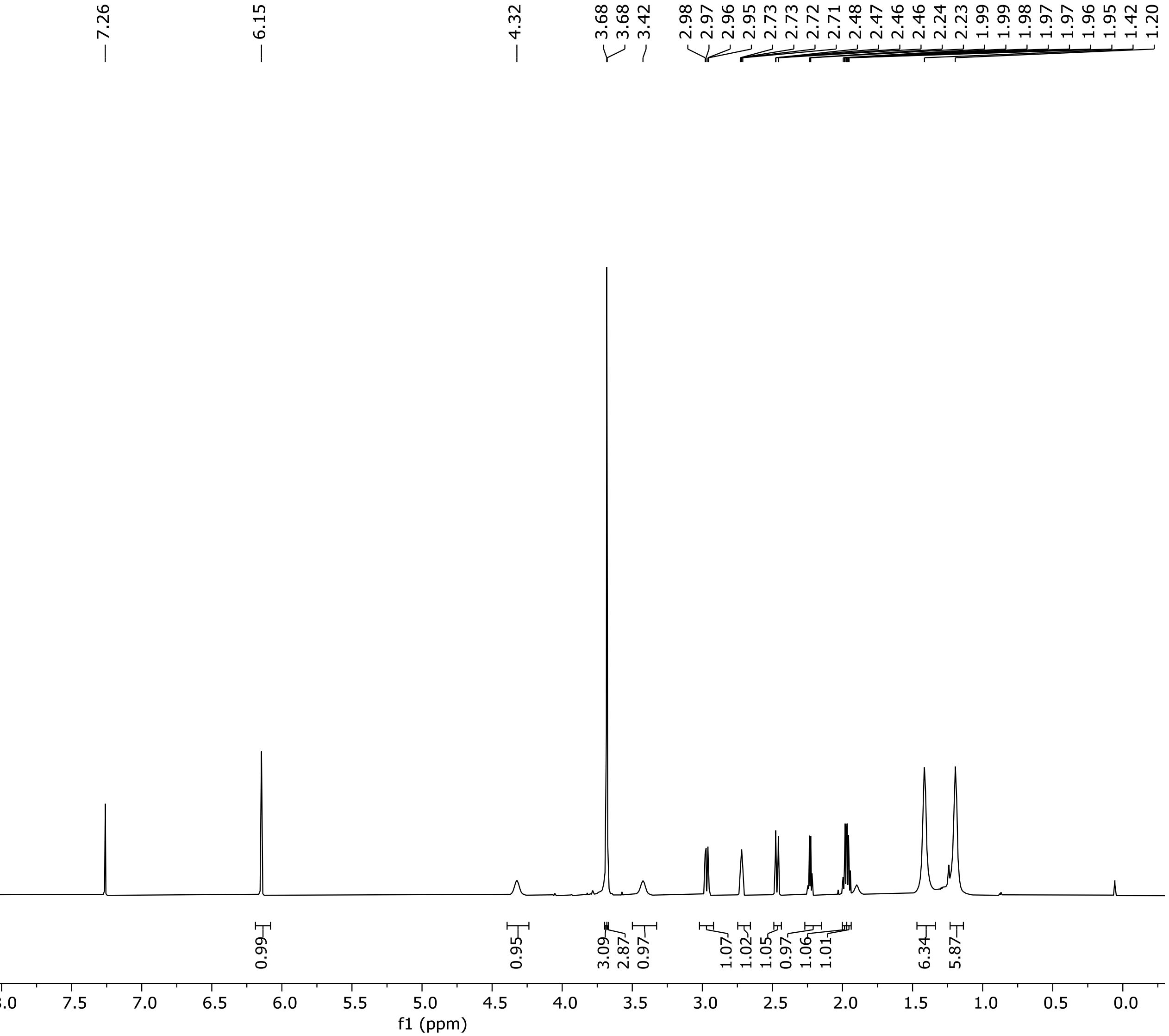

^{13}C NMR (176 MHz, $CDCl_3$, 298 K) spectrum of **3y**.

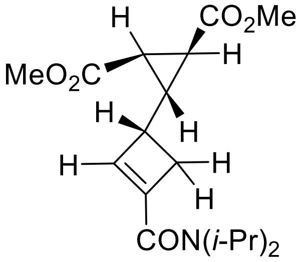

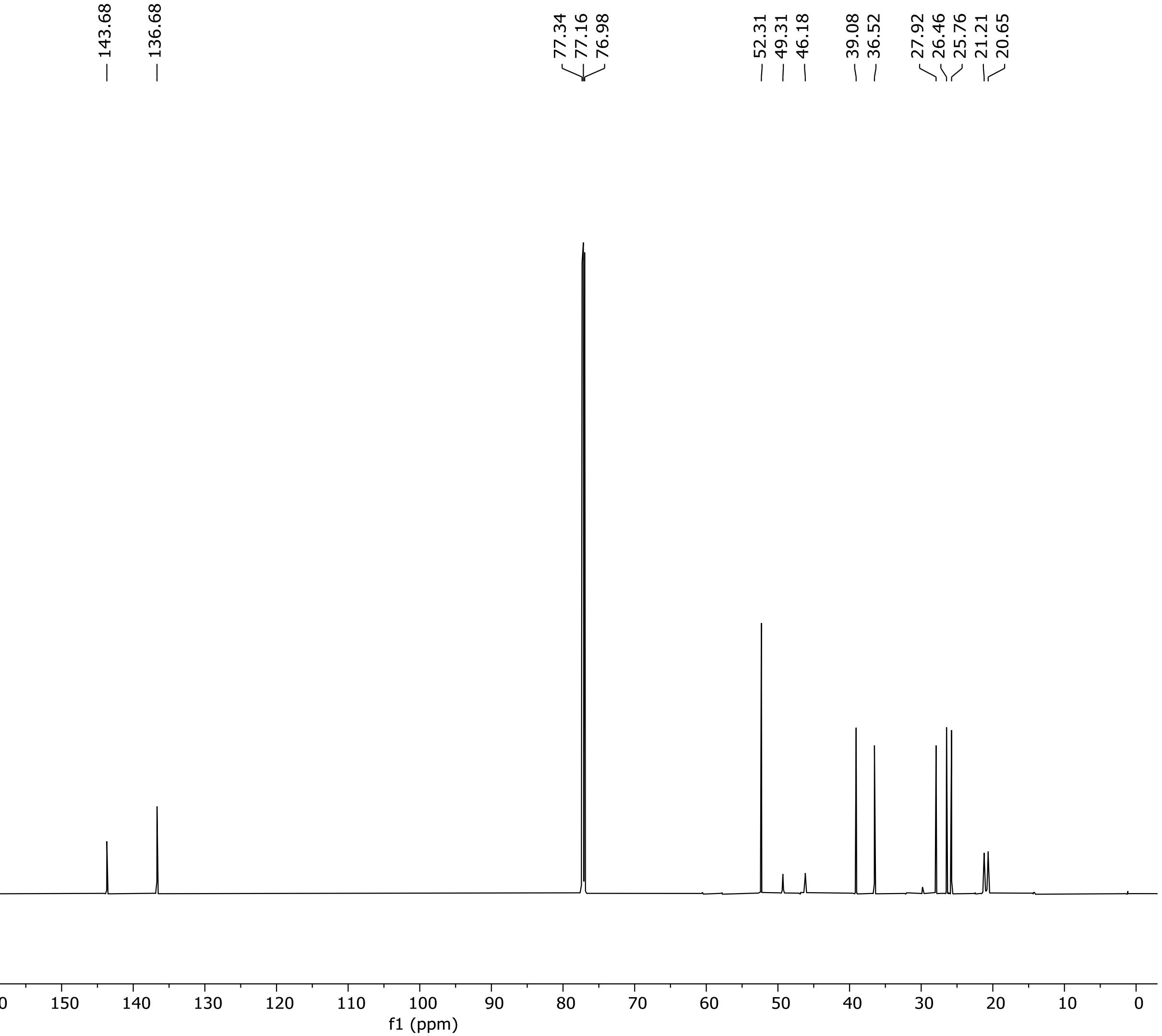

COSY (^1H–^1H) NMR (700 MHz, $CDCl_3$, 298 K) spectrum of **3y**.

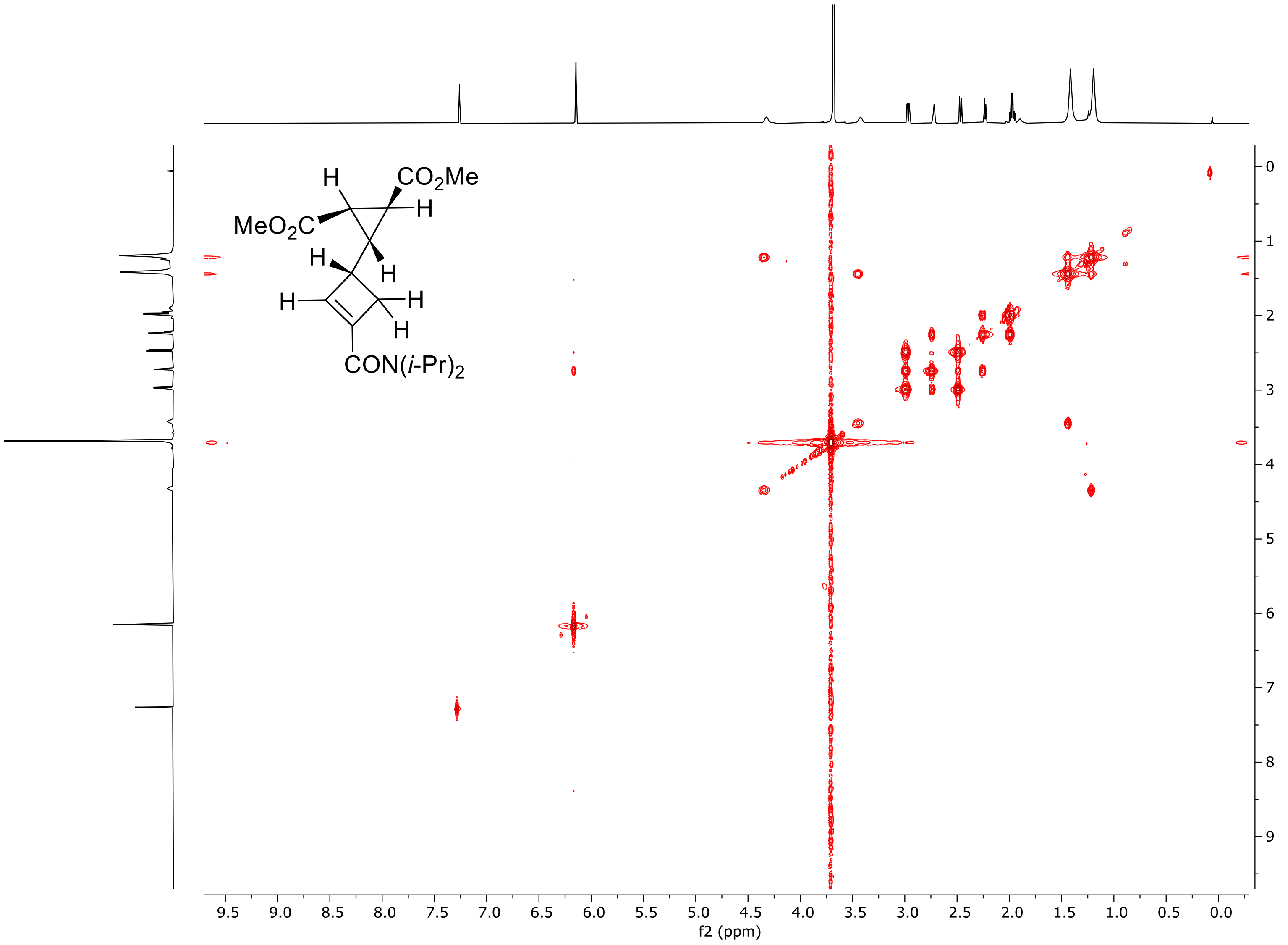

HSQC (^{1}H–^{13}C) NMR (700 MHz, $CDCl_3$, 298 K) spectrum of **3y**.

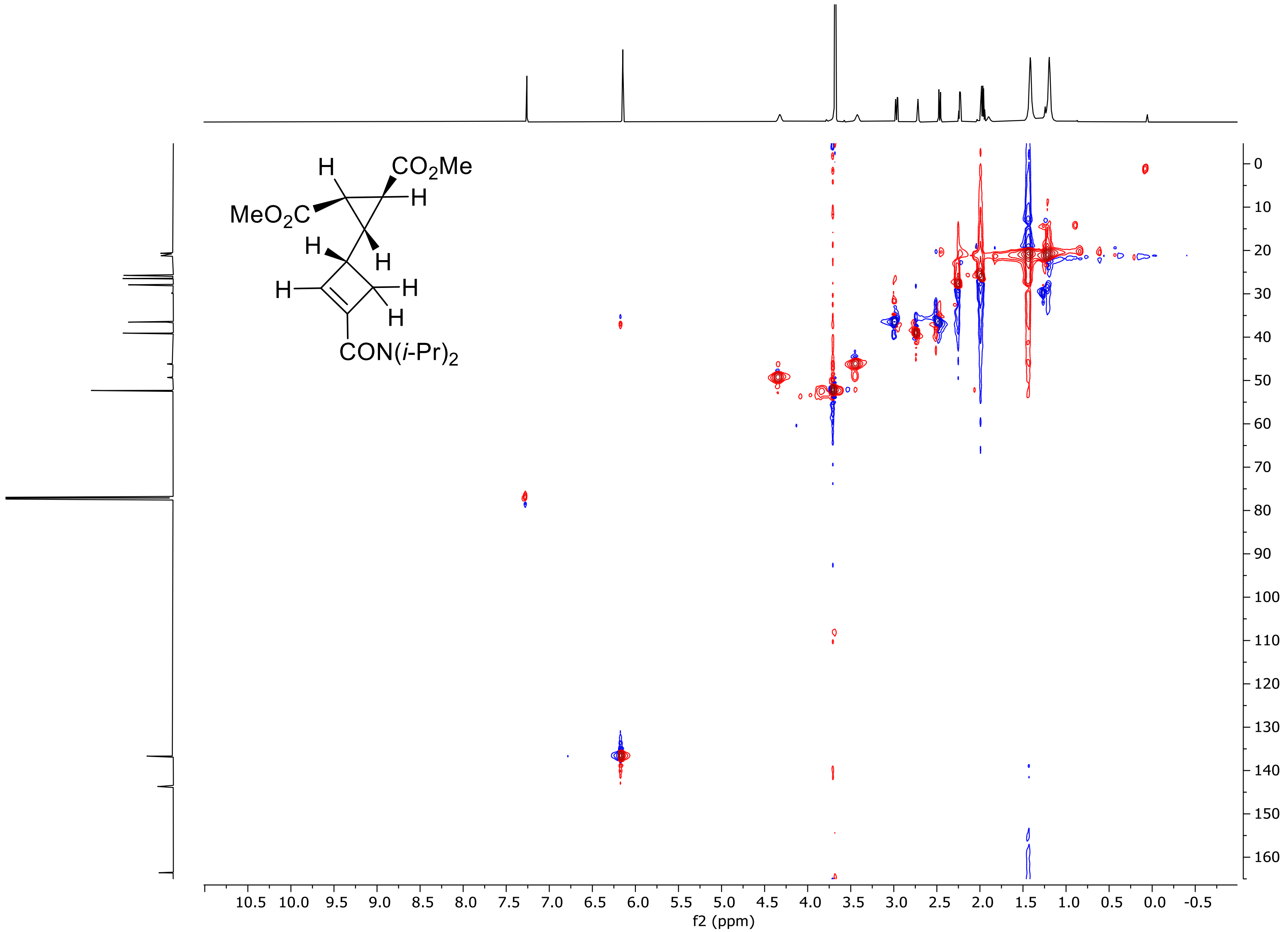

^{1}H NMR (500 MHz, $CDCl_3$, 298 K) spectrum of **3z**.

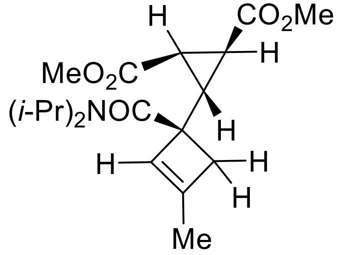

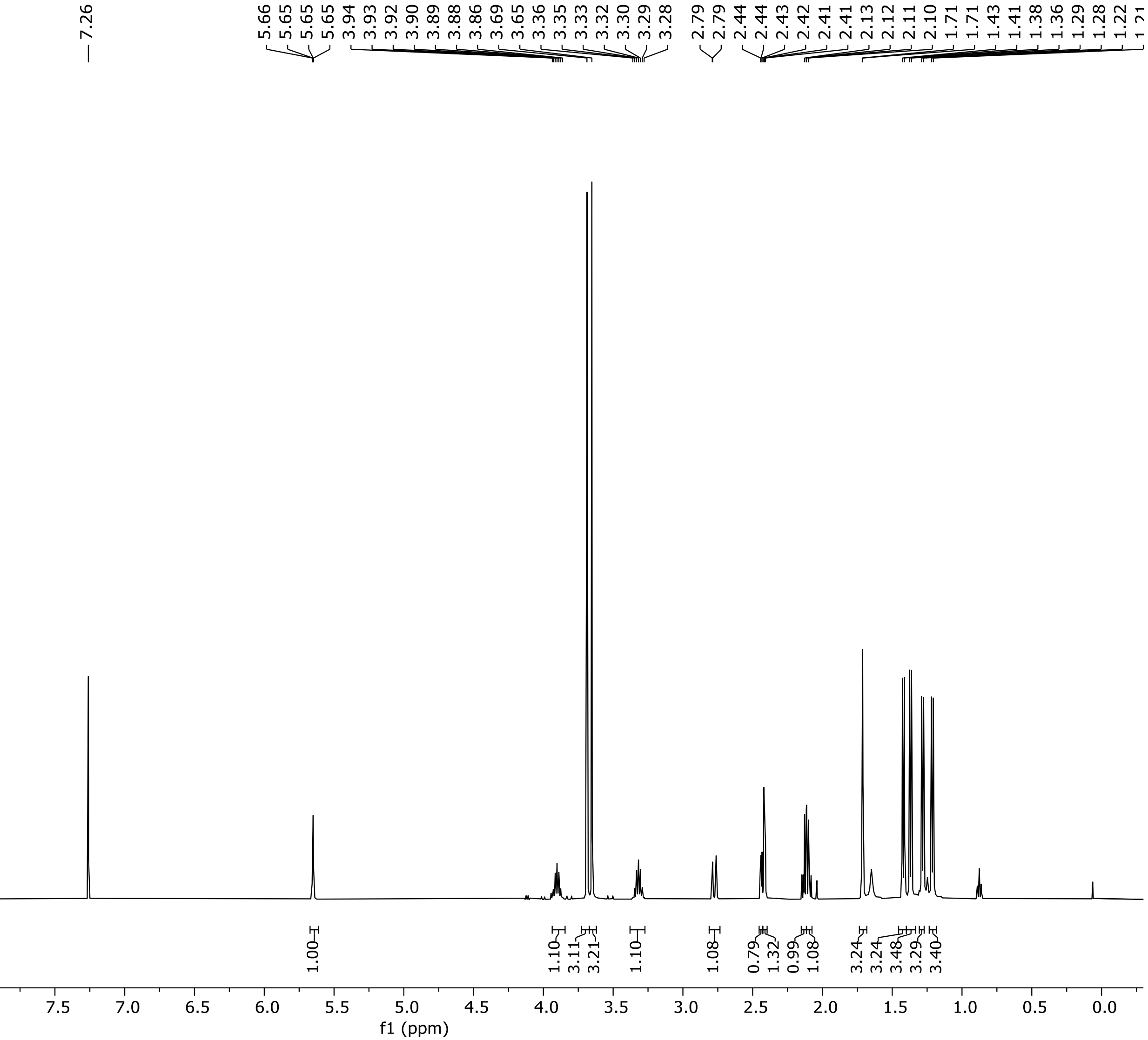

^{13}C NMR (126 MHz, $CDCl_3$, 298 K) spectrum of **3z**.

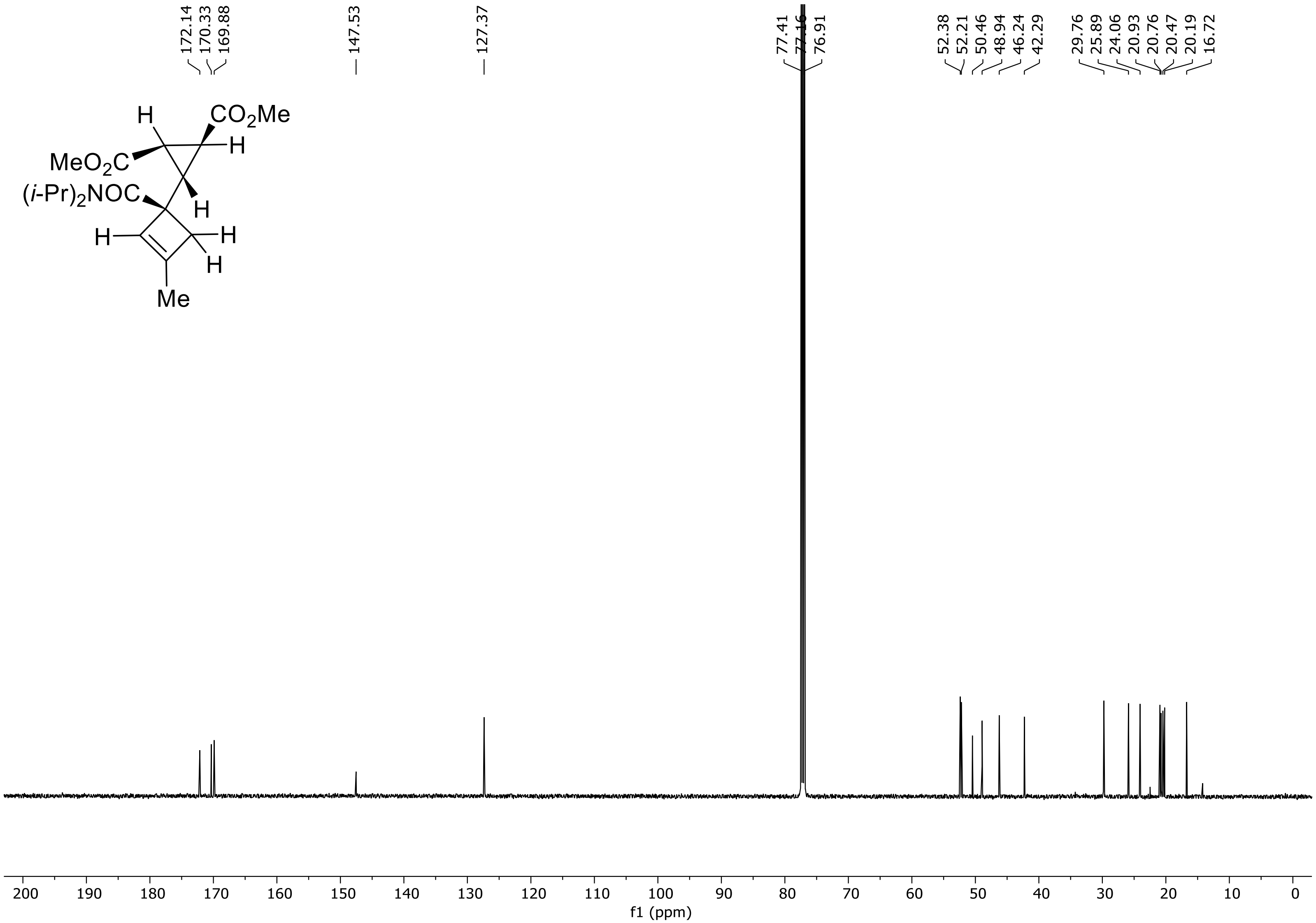

COSY (^{1}H–^{1}H) NMR (500 MHz, $CDCl_3$, 298 K) spectrum of **3z**.

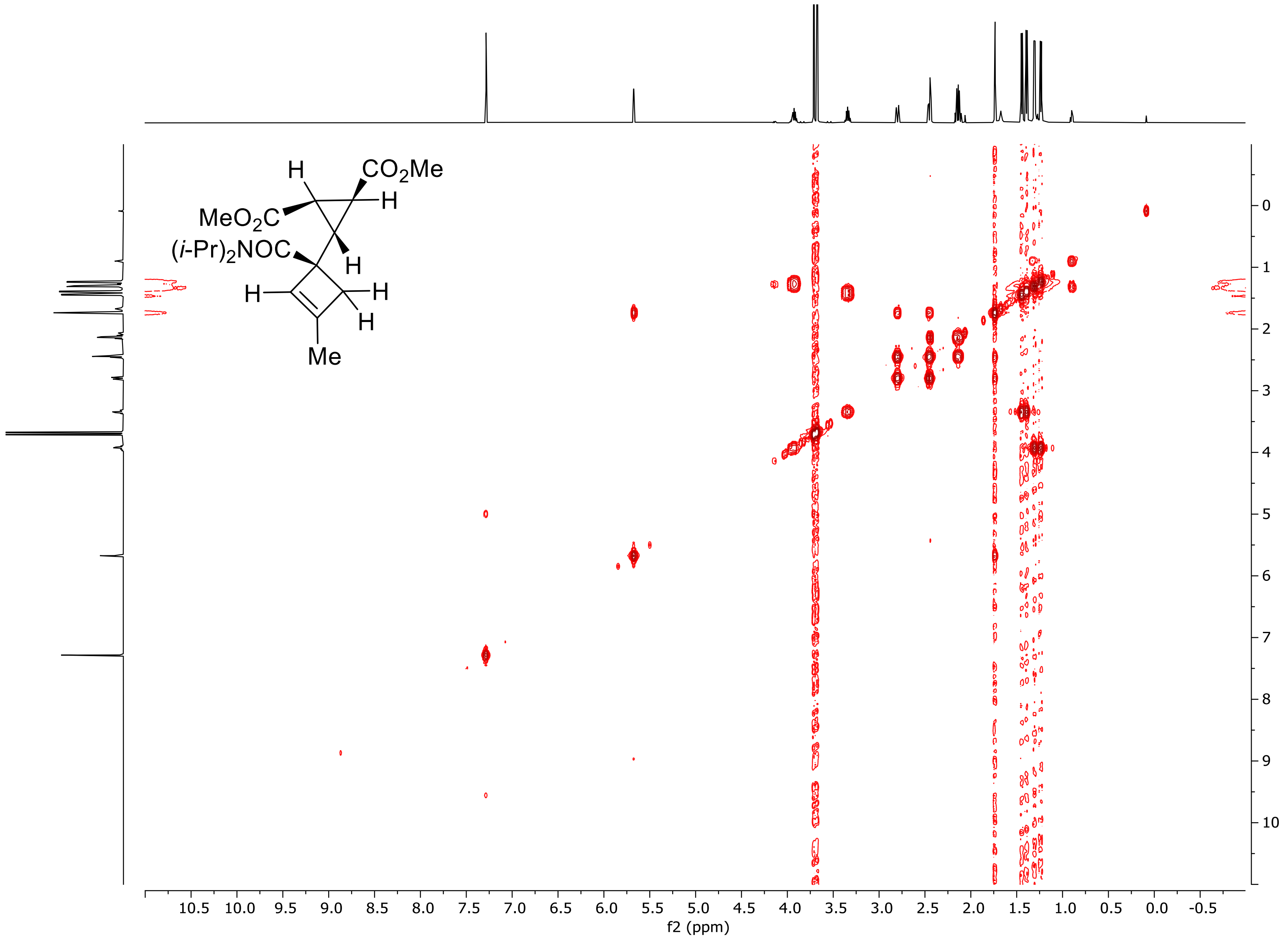

HSQC (^1H–^{13}C) NMR (500 MHz, $CDCl_3$, 298 K) spectrum of **3z**.

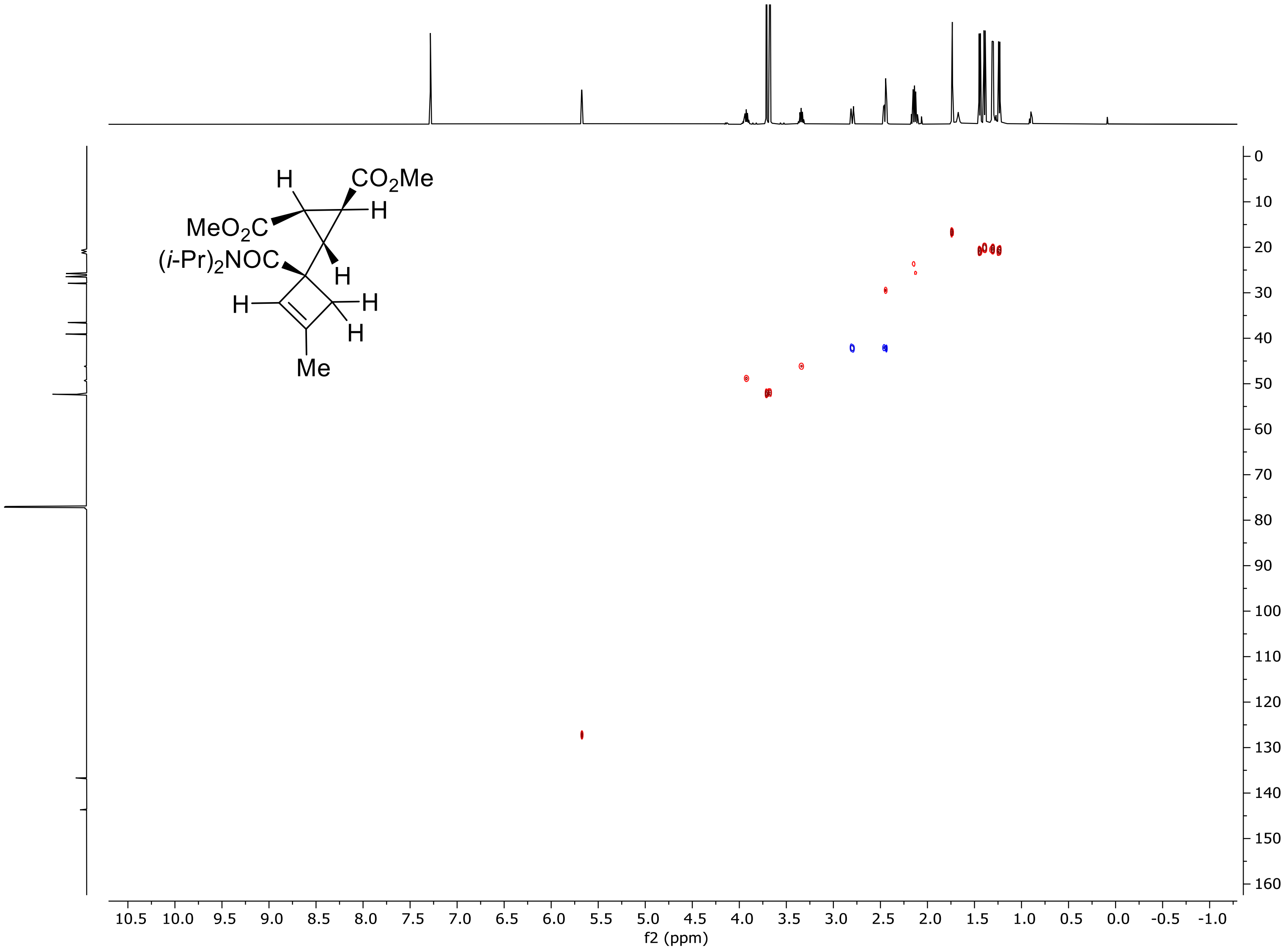

^{1}H NMR (500 MHz, $CDCl_3$, 298 K) spectrum of **3z'** (minor isomer).

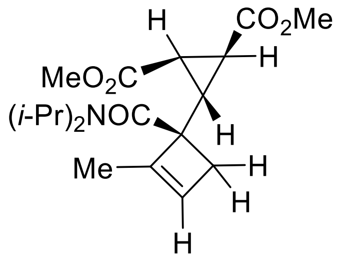

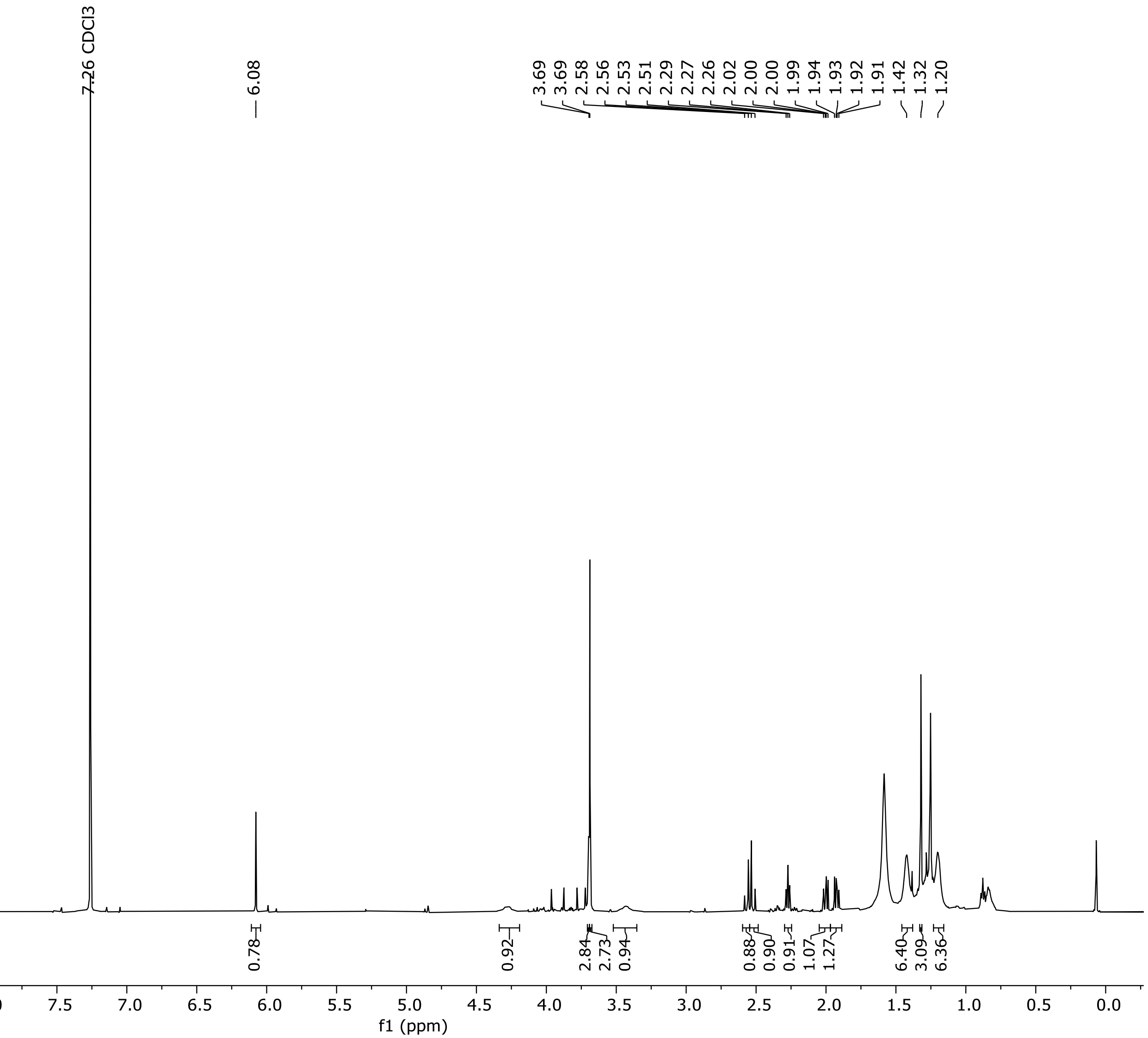

^{13}C NMR (126 MHz, $CDCl_3$, 298 K) spectrum of **3z'** (minor isomer).

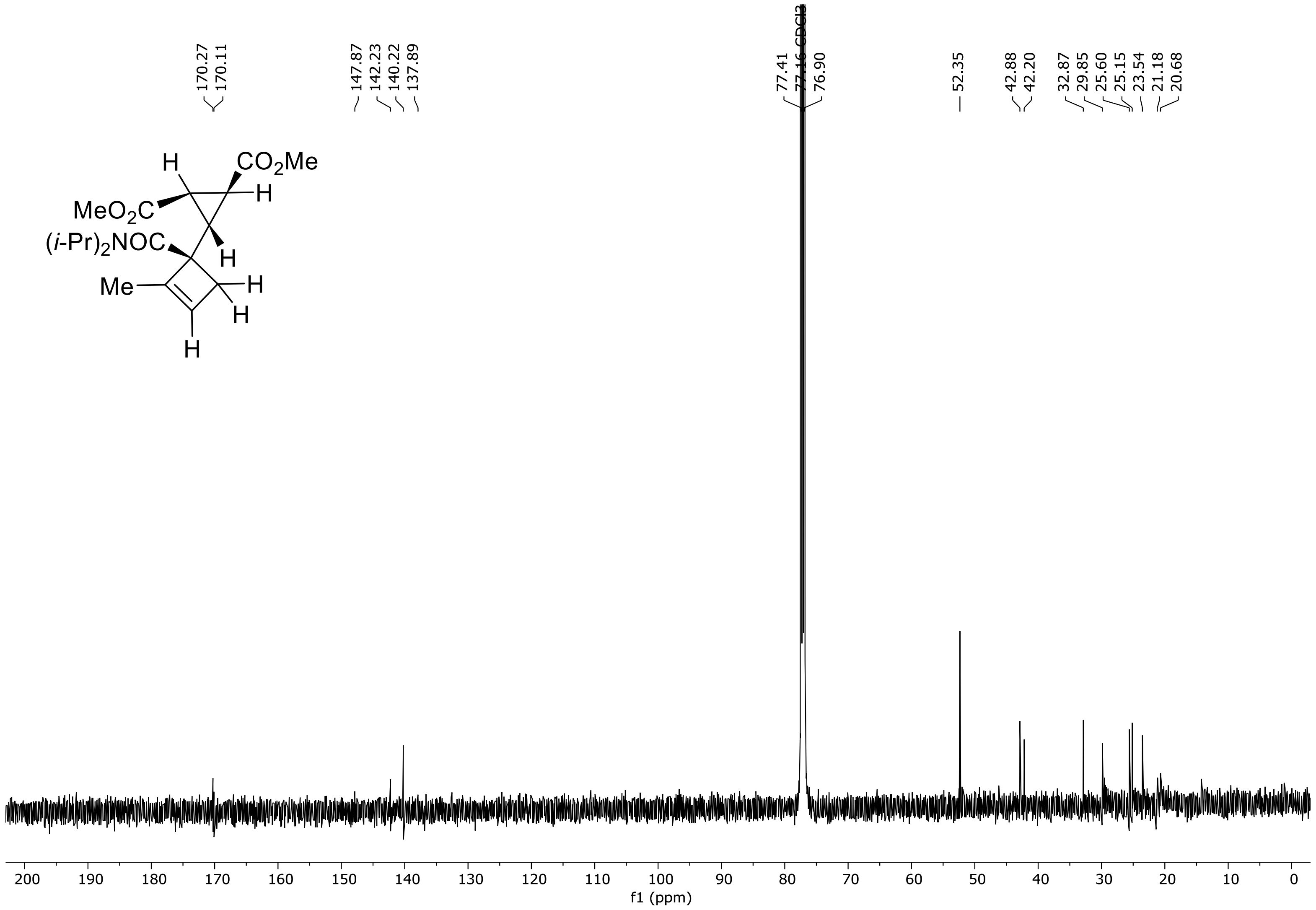

^{1}H NMR (500 MHz, $CDCl_3$, 298 K) spectrum of **3aa**.

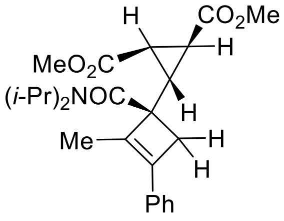

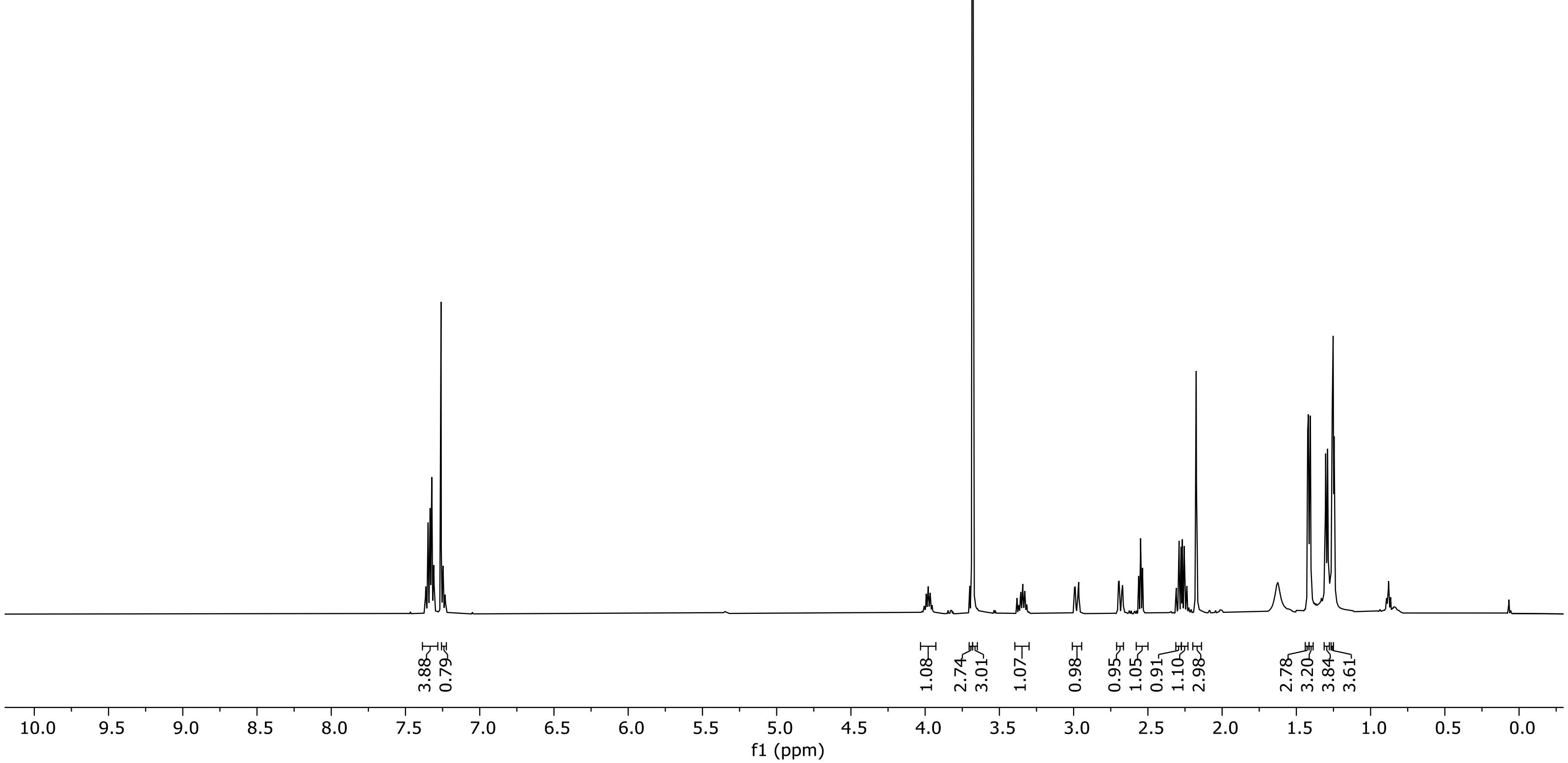

^{13}C NMR (126 MHz, $CDCl_3$, 298 K) spectrum of **3aa**.

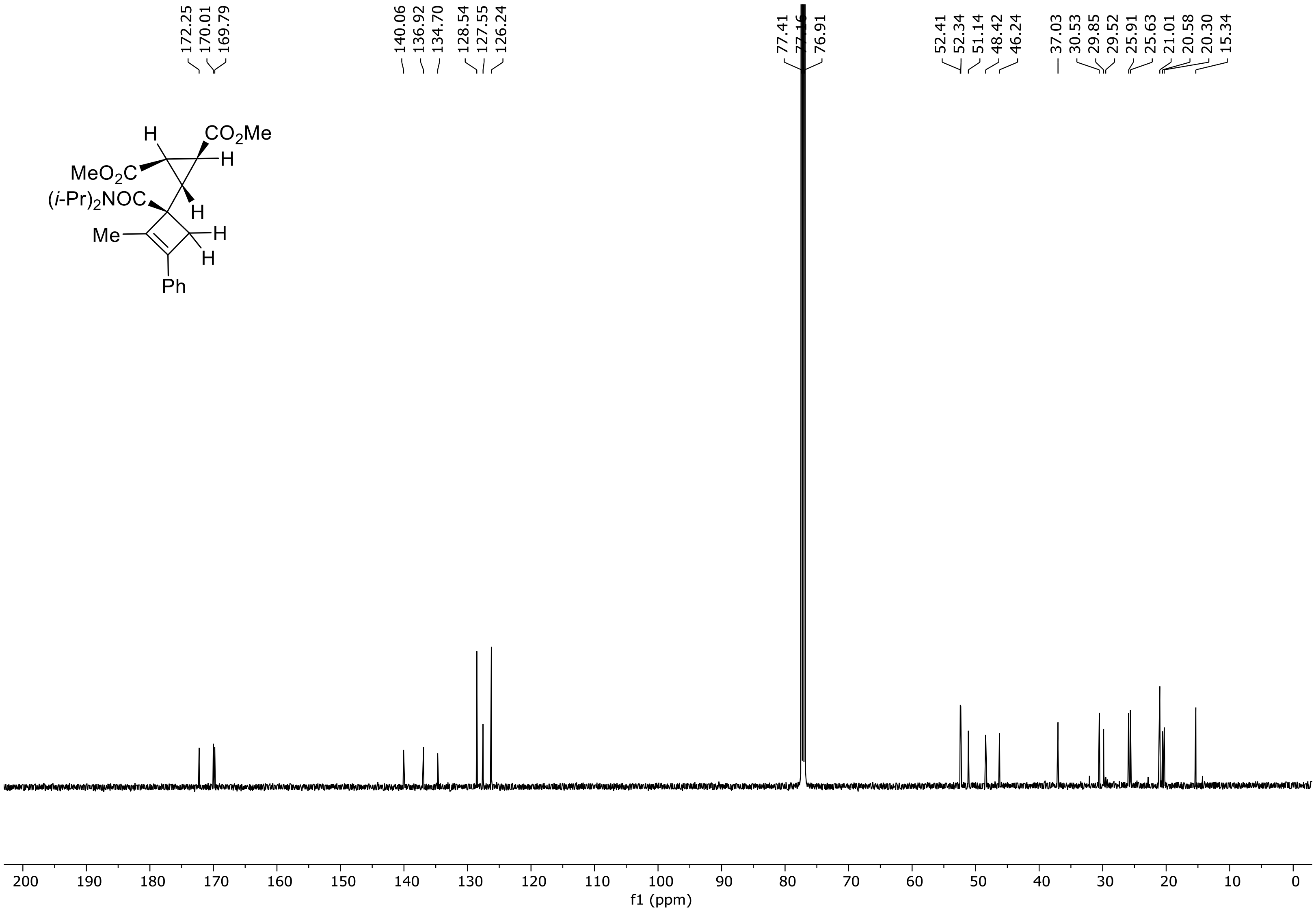

^{1}H NMR (500 MHz, $CDCl_3$, 298 K) spectrum of **3ab**.

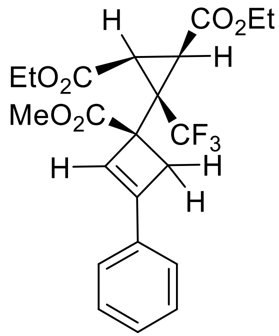

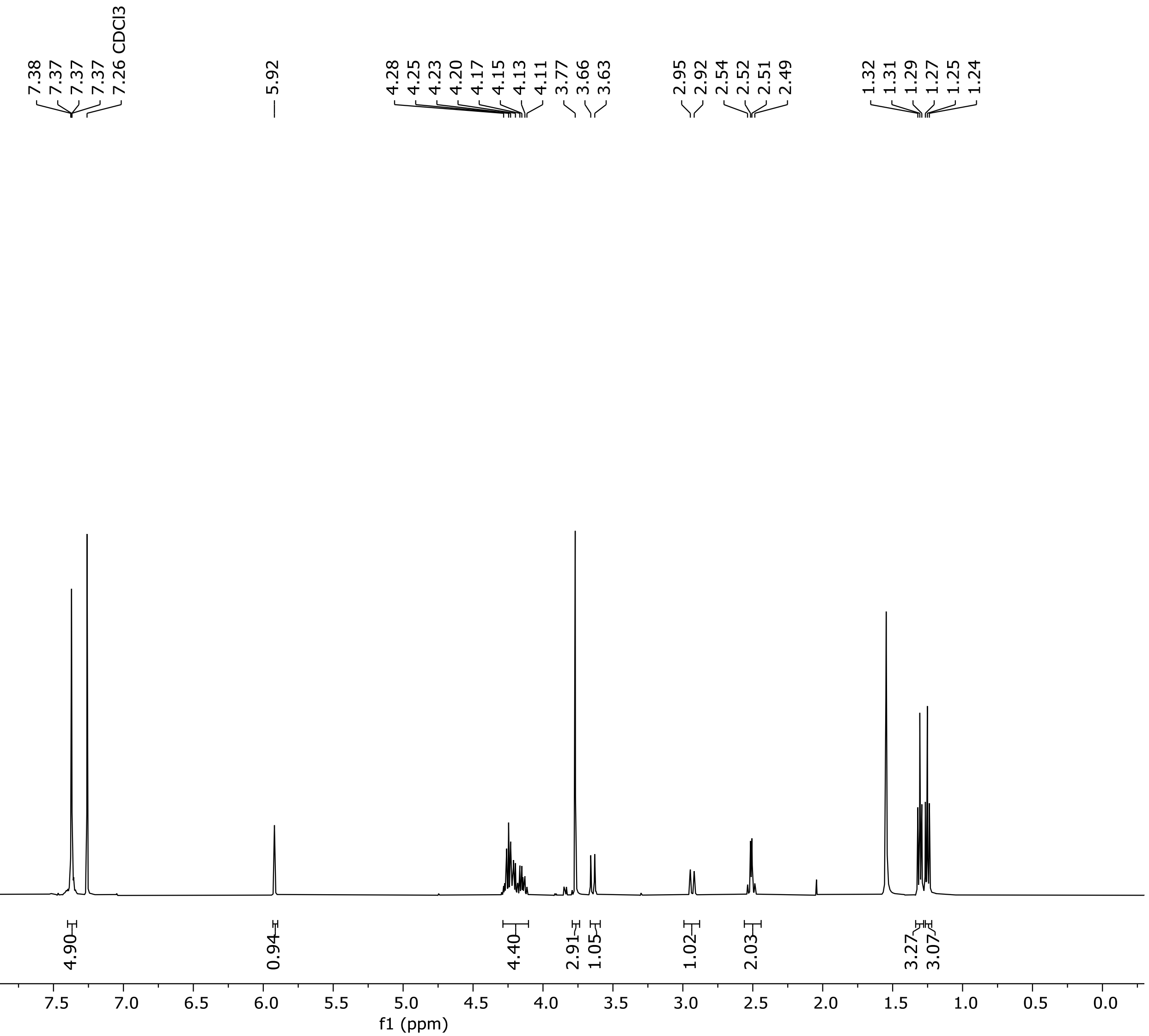

^{13}C NMR (126 MHz, $CDCl_3$, 298 K) spectrum of **3ab**.

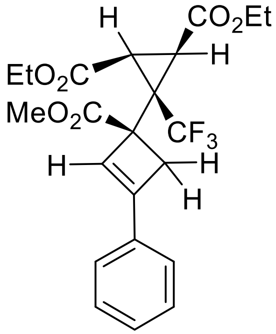

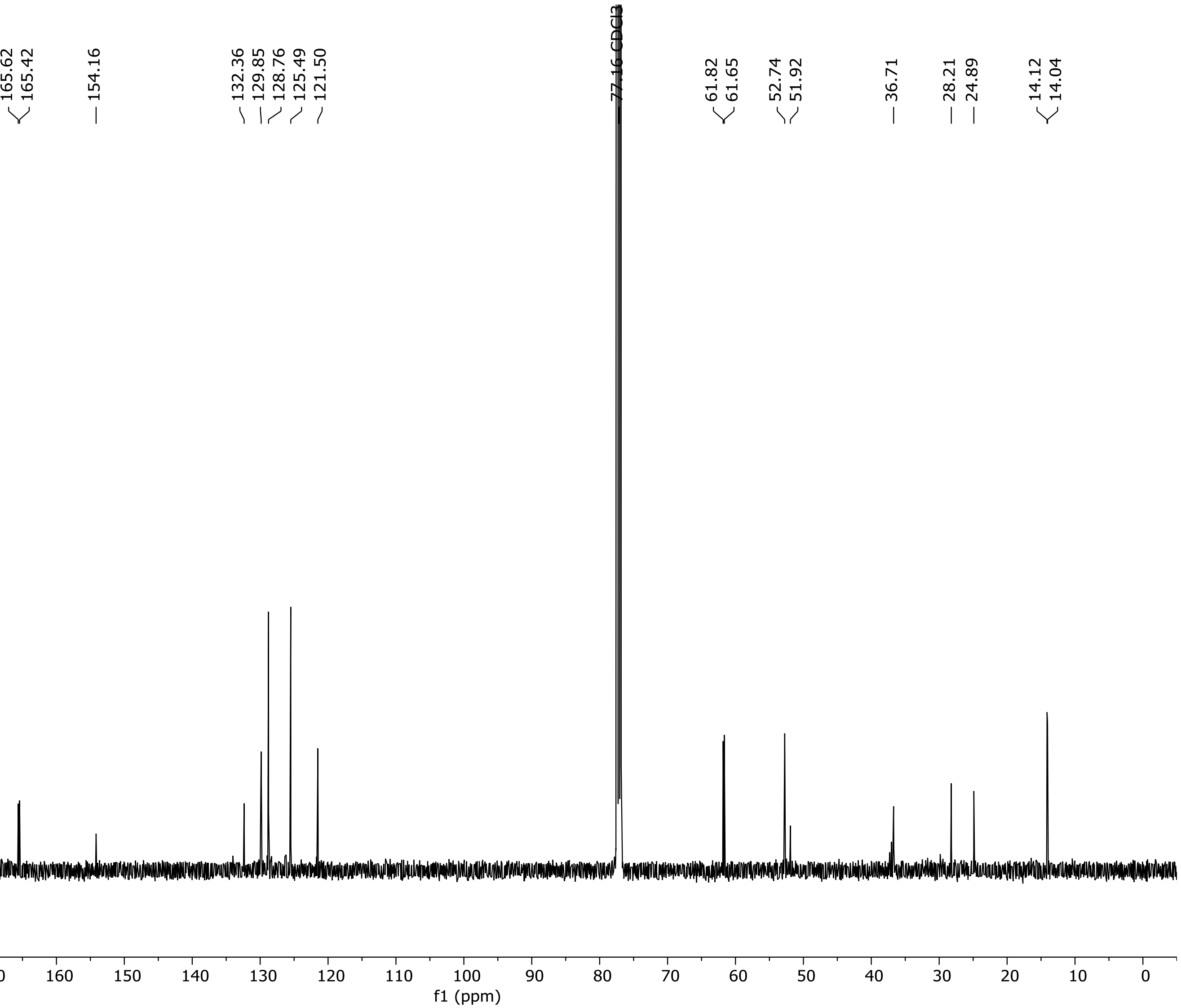

^{19}F NMR (377 MHz, $CDCl_3$, 298 K) spectrum of **3ab**.

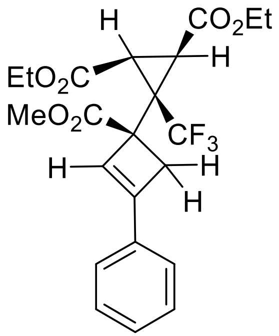

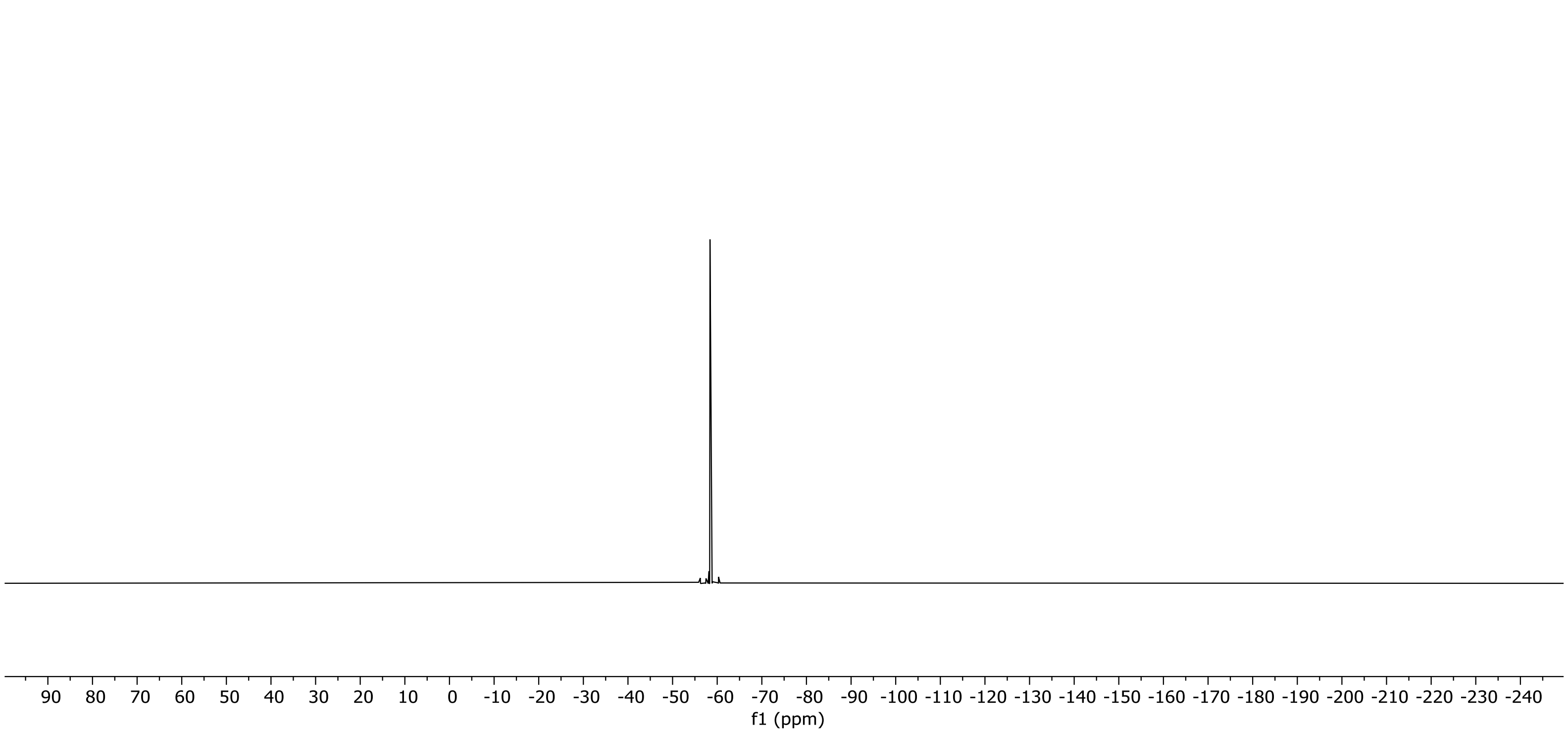

COSY (^{1}H–^{1}H) NMR (500 MHz, $CDCl_3$, 298 K) spectrum of **3ab**.

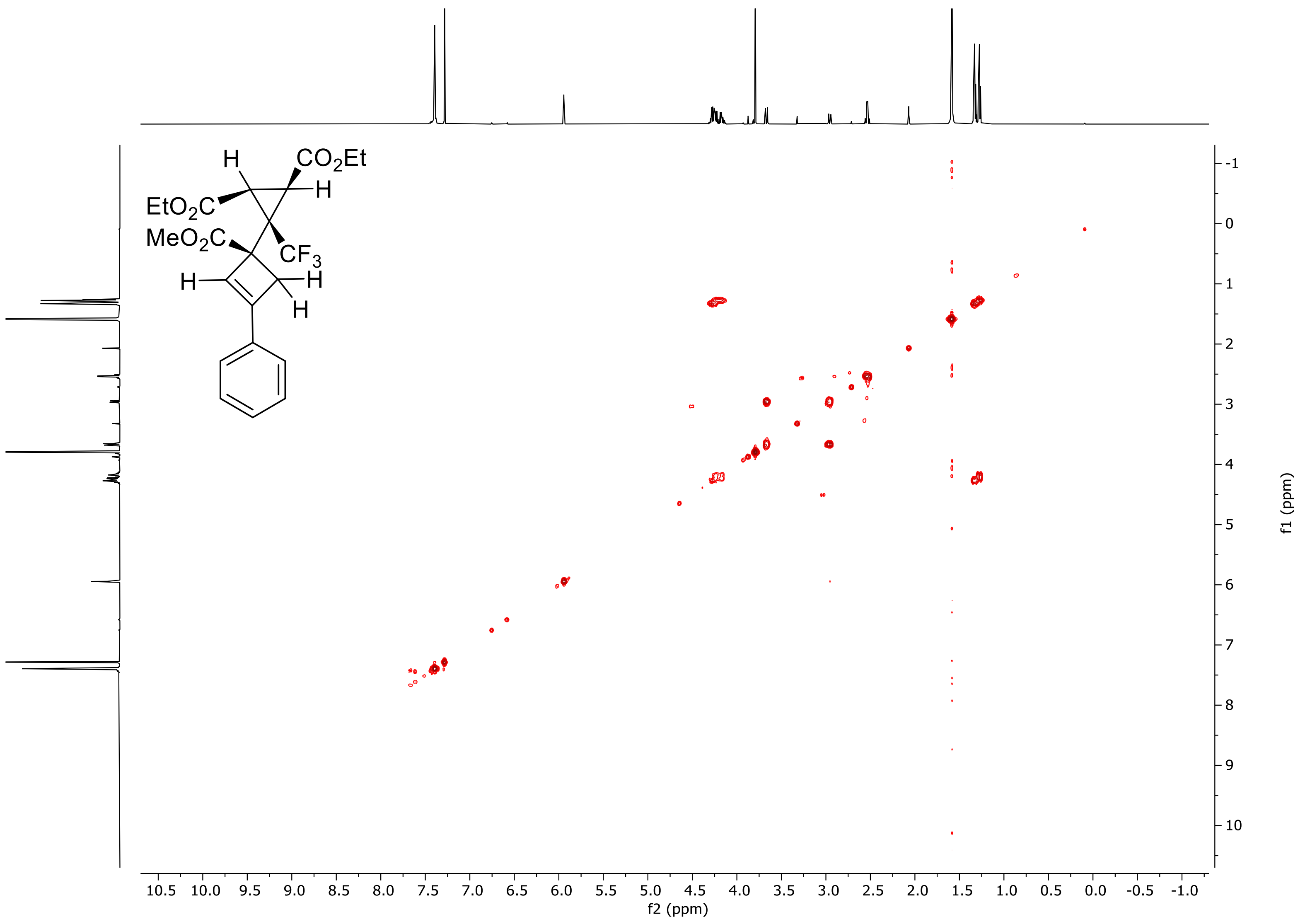

HSQC (^{1}H–^{13}C) NMR (500 MHz, $CDCl_3$, 298 K) spectrum of **3ab**.

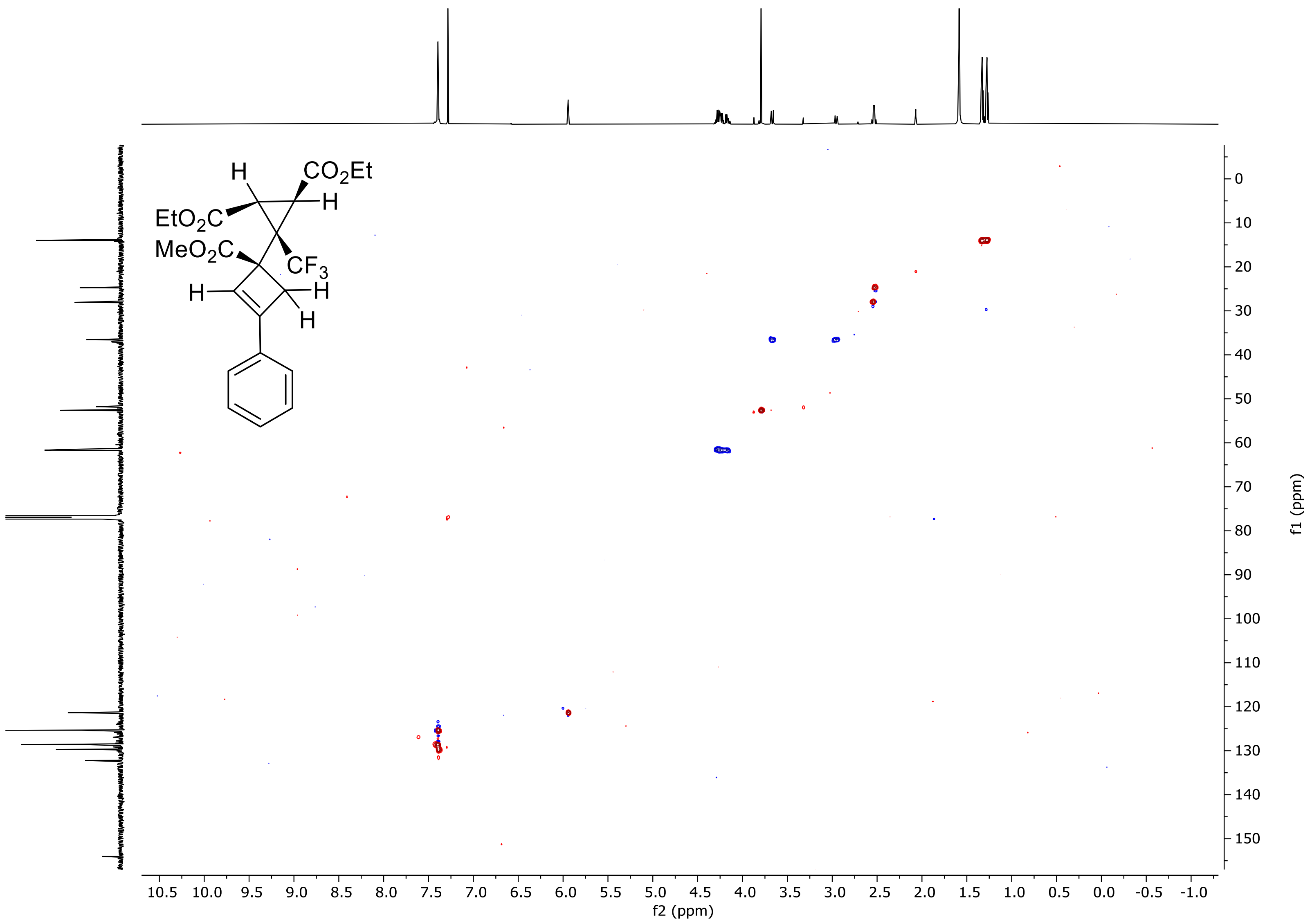

^{1}H NMR (500 MHz, $CDCl_3$, 298 K) spectrum of **3ab'**.

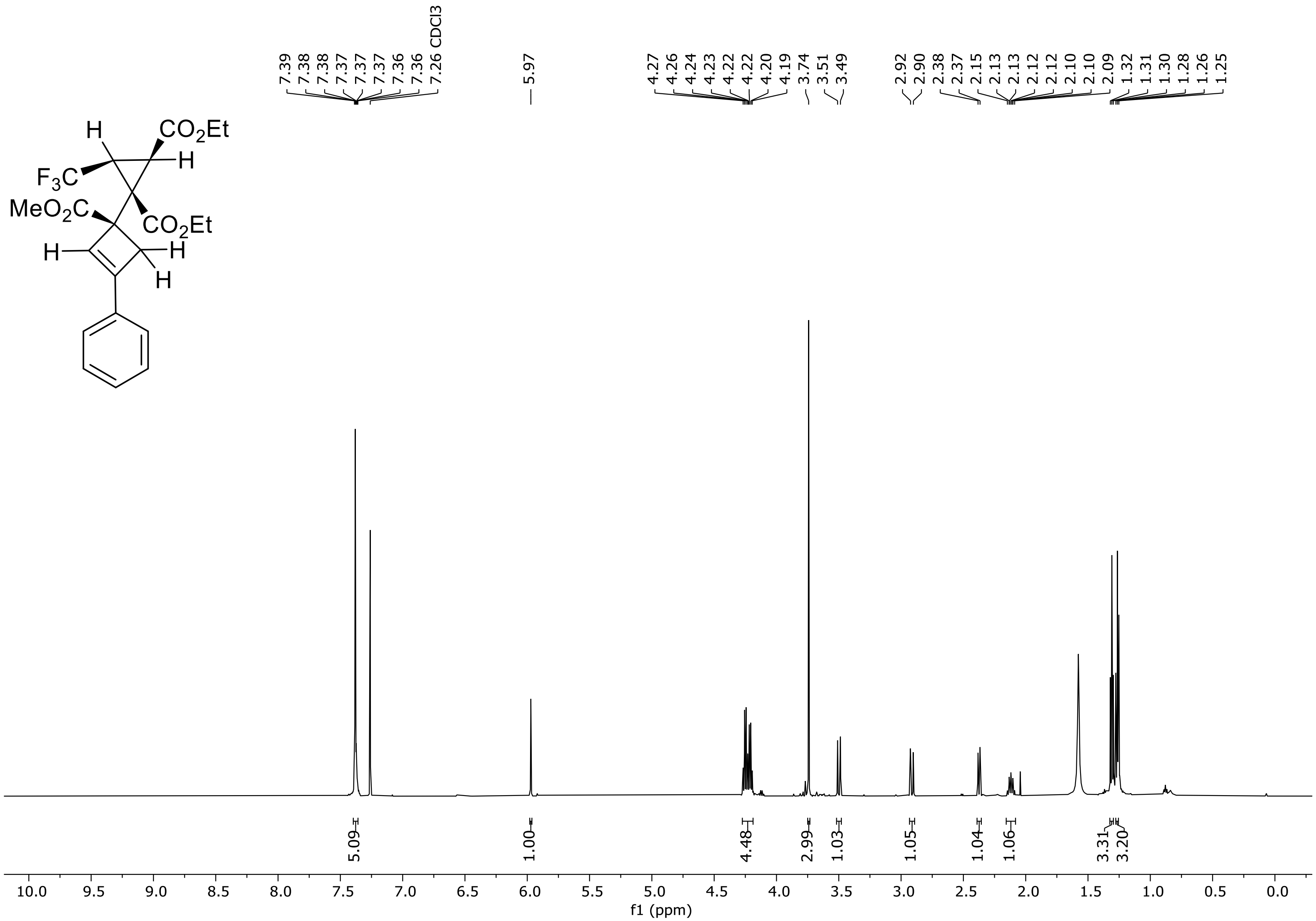

^{13}C NMR (126 MHz, $CDCl_3$, 298 K) spectrum of **3ab'**.

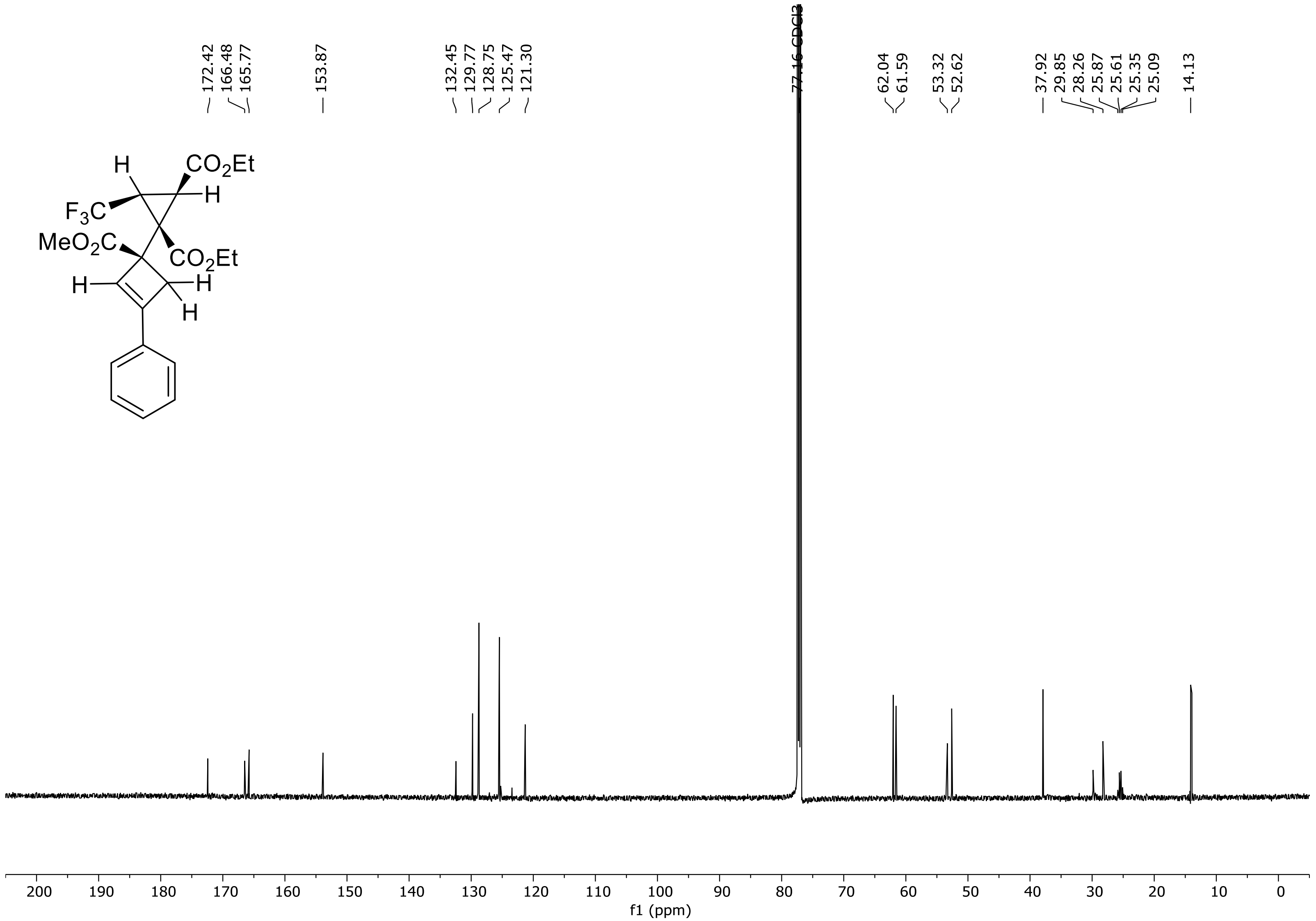

^{19}F NMR (377 MHz, $CDCl_3$, 298 K) spectrum of **3ab'**.

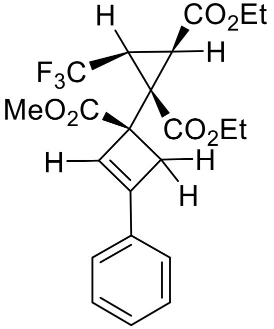

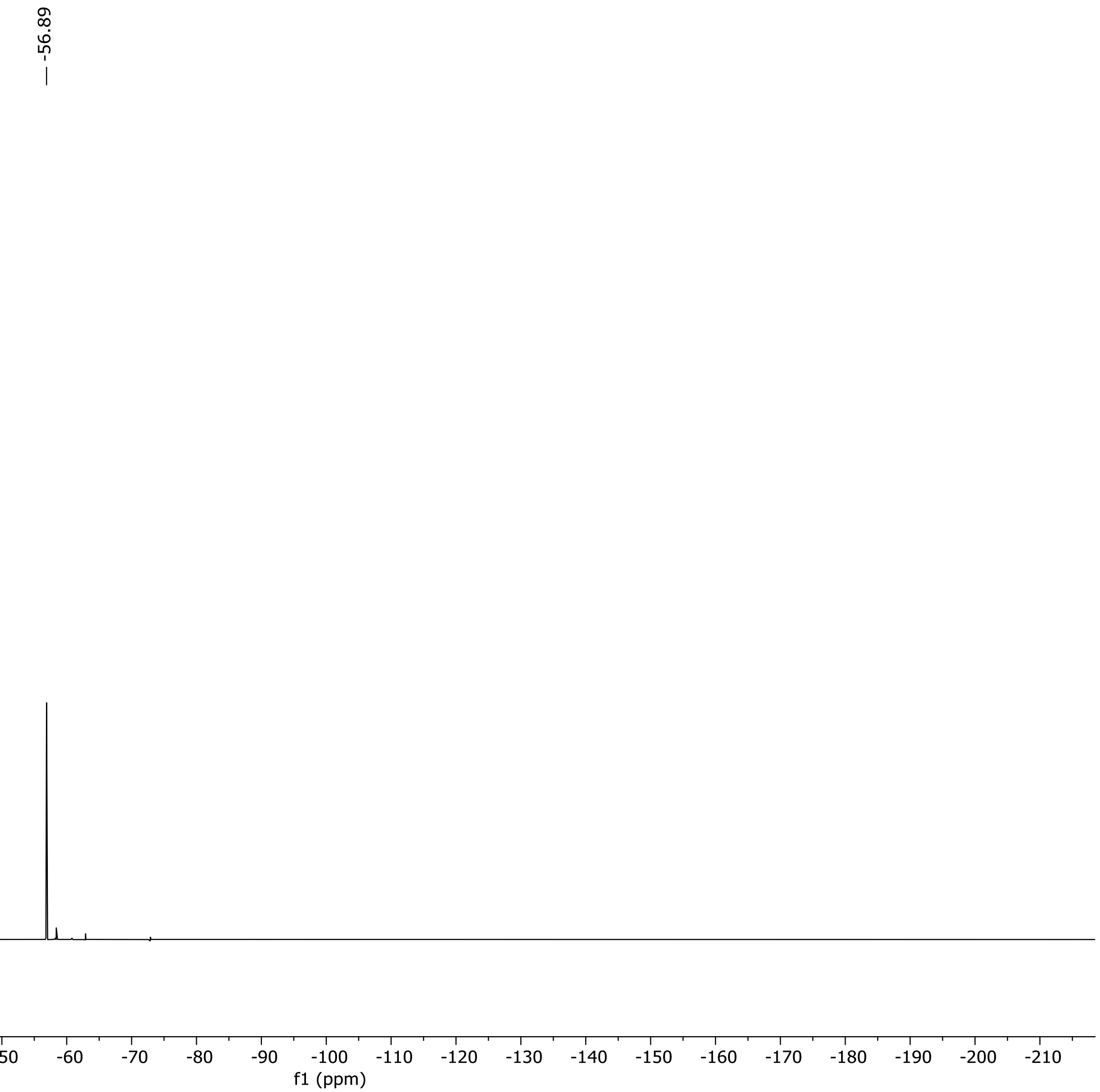

COSY (^1H–^1H) NMR (500 MHz, $CDCl_3$, 298 K) spectrum of **3ab'**.

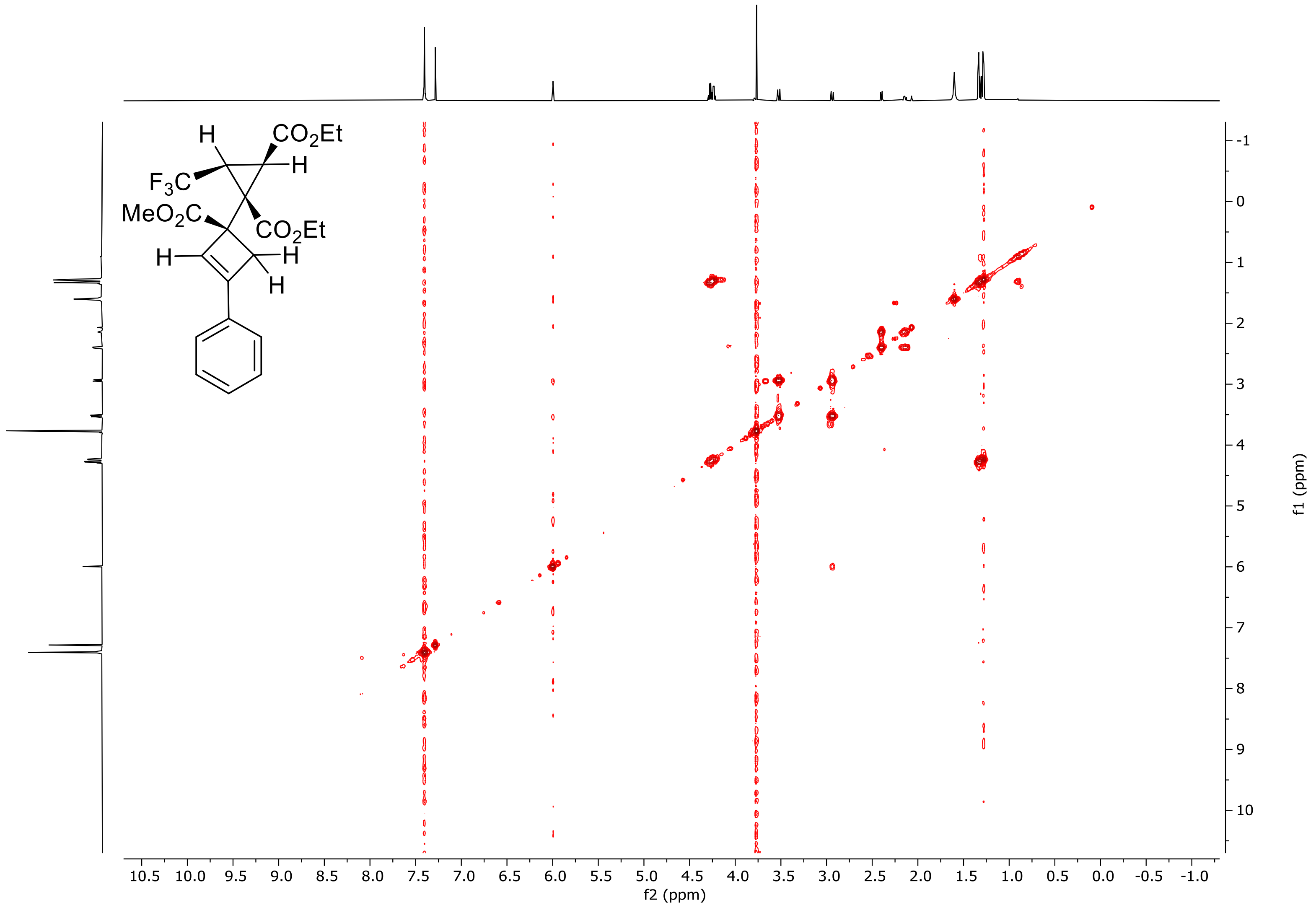

HSQC (^1H–^{13}C) NMR (500 MHz, $CDCl_3$, 298 K) spectrum of **3ab'**.

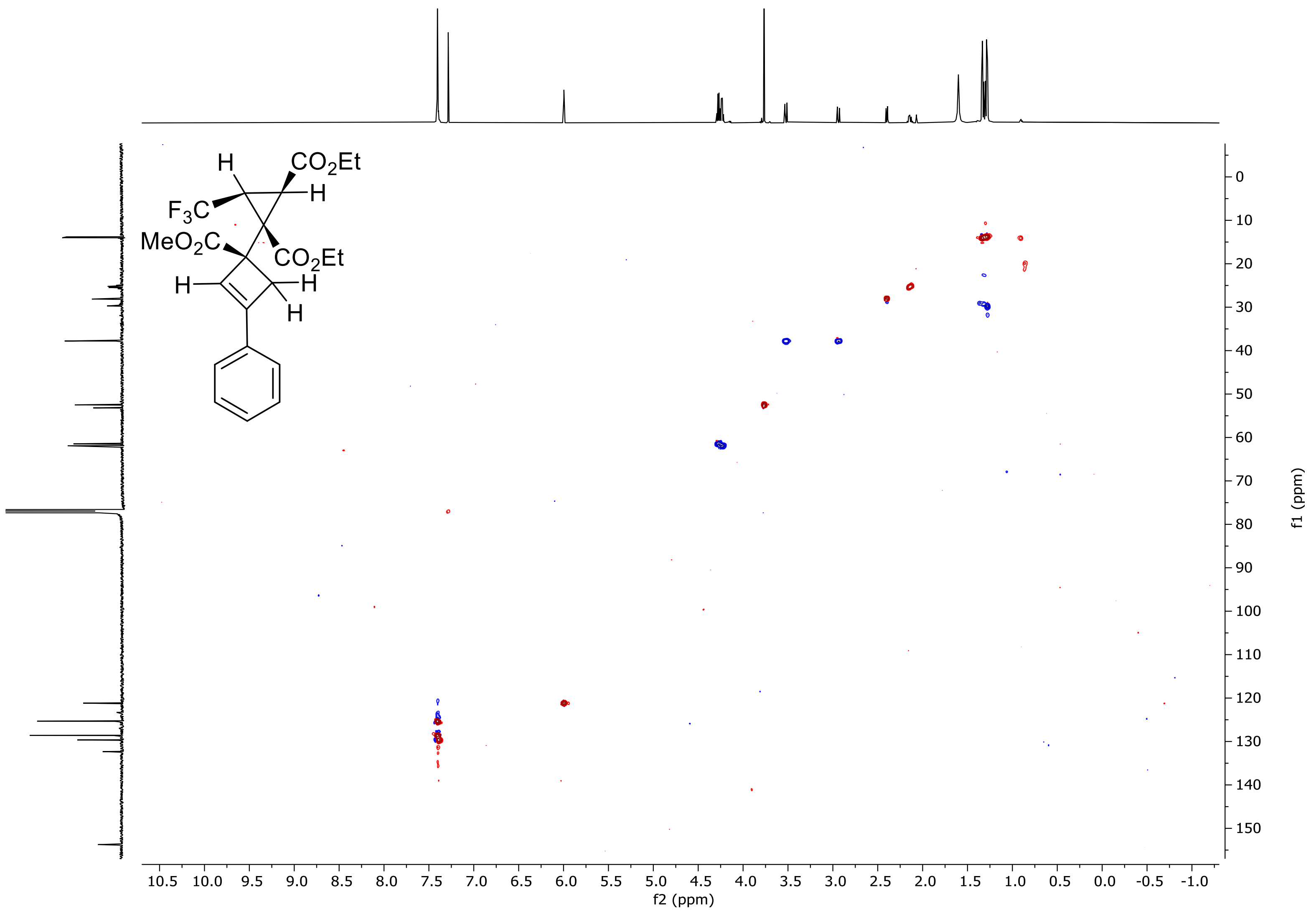

^{1}H NMR (400 MHz, C_6D_6, 298 K) spectrum of **7**.

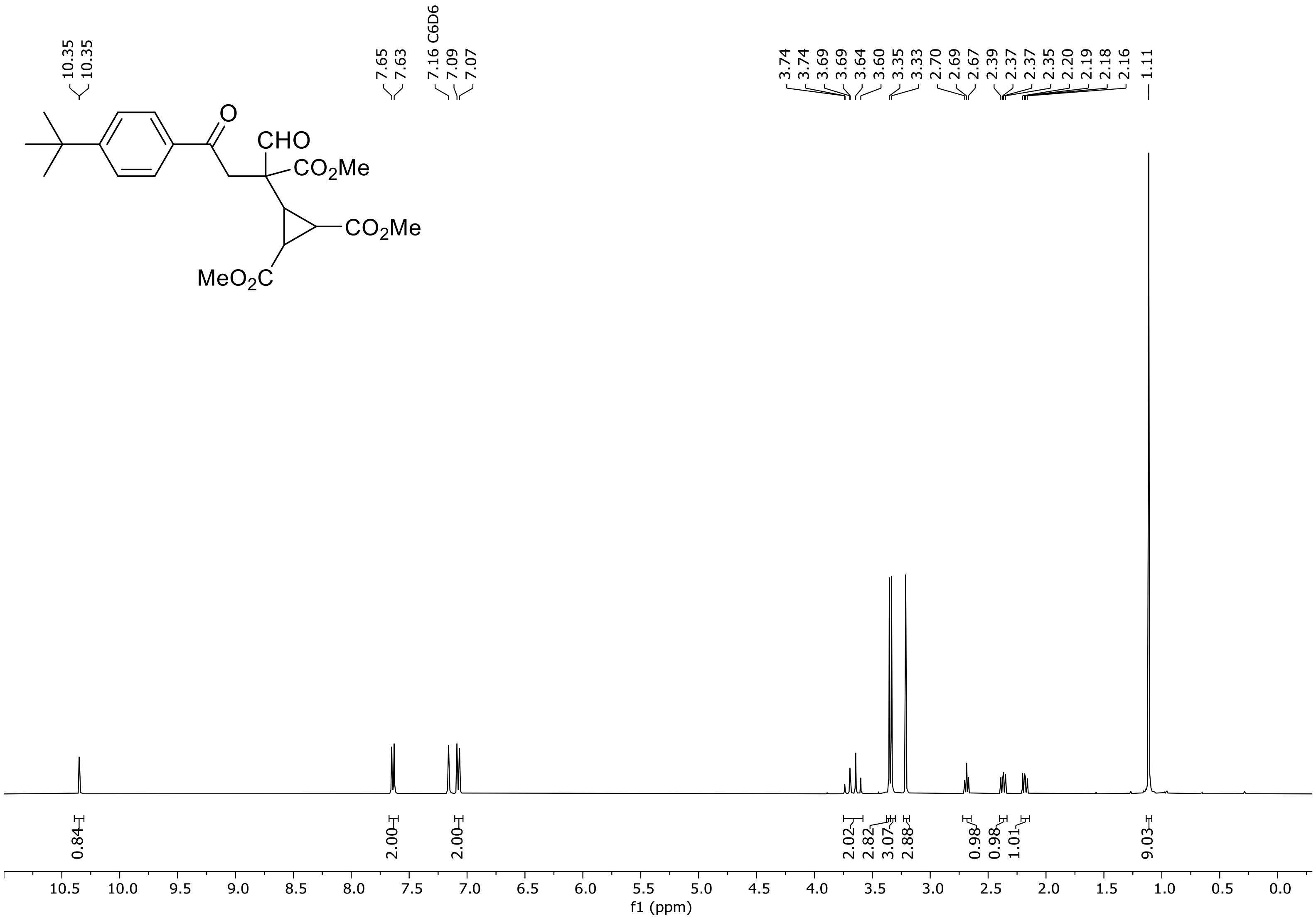

^{13}C NMR (100 MHz, C_6D_6, 298 K) spectrum of **7**.

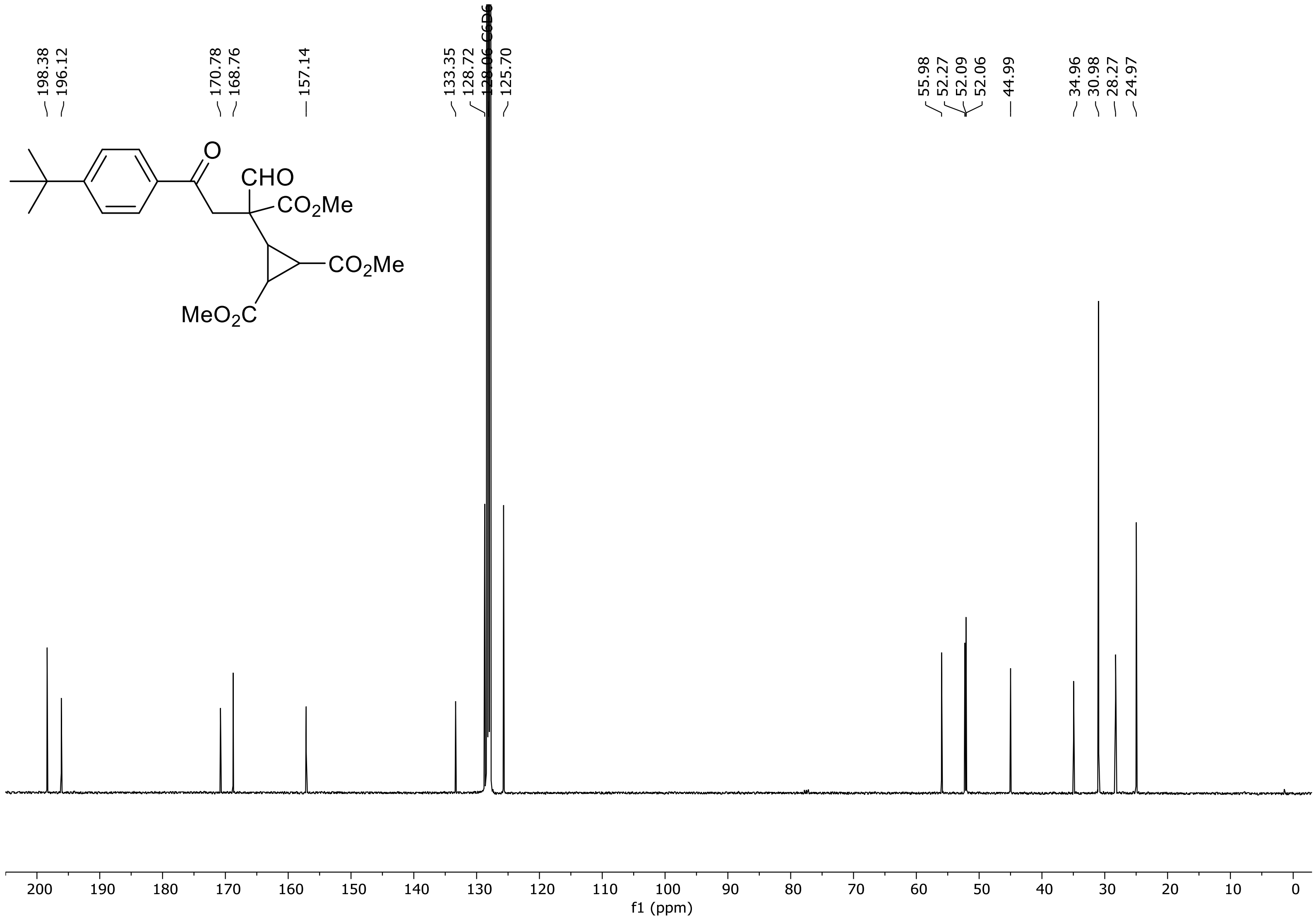

COSY (^1H–^1H) NMR (400 MHz, C_6D_6, 298 K) spectrum of **7**.

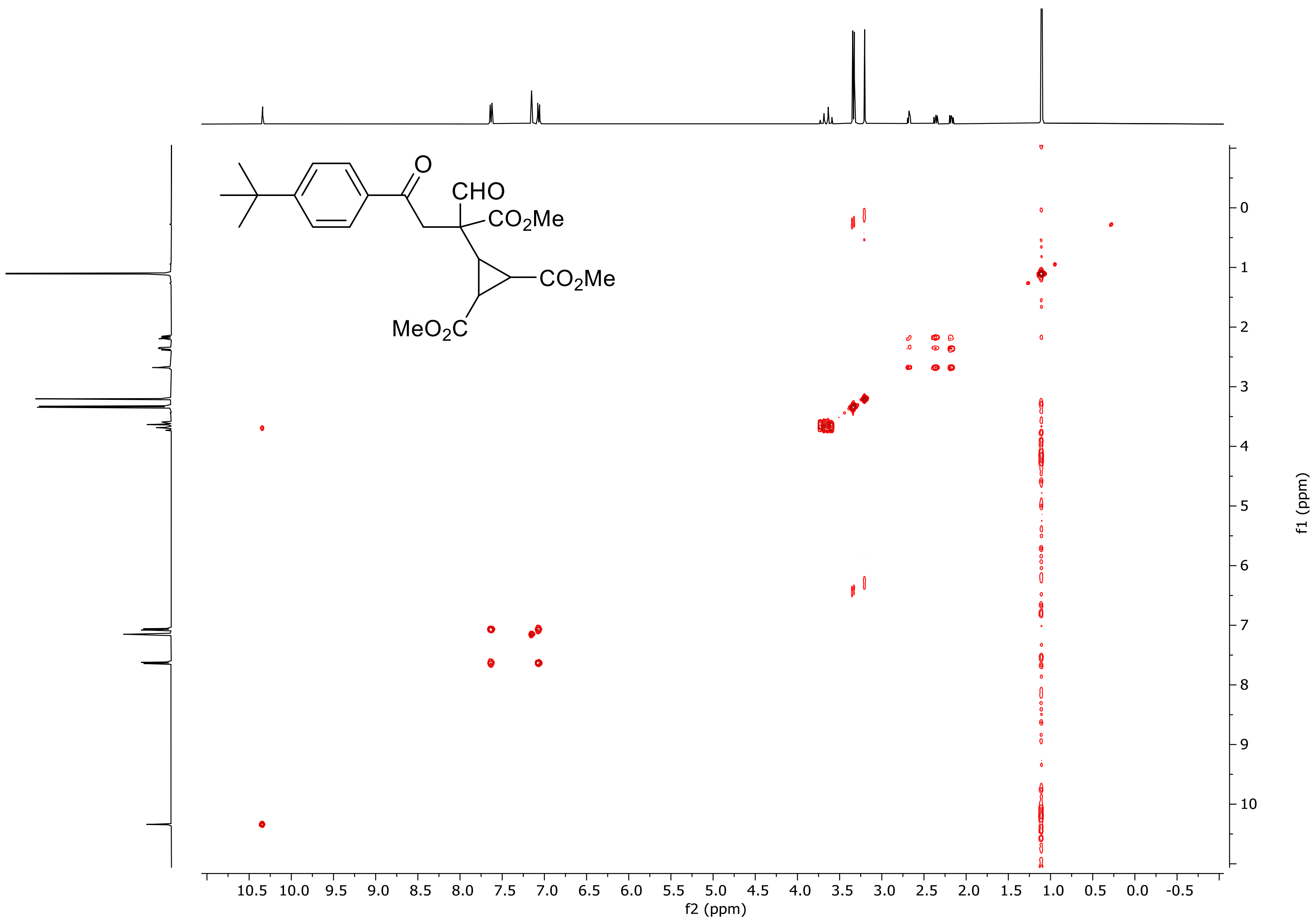

HSQC ($^{1}H–^{13}C$) NMR (400 MHz, $CDCl_3$, 298 K) spectrum of **7**.

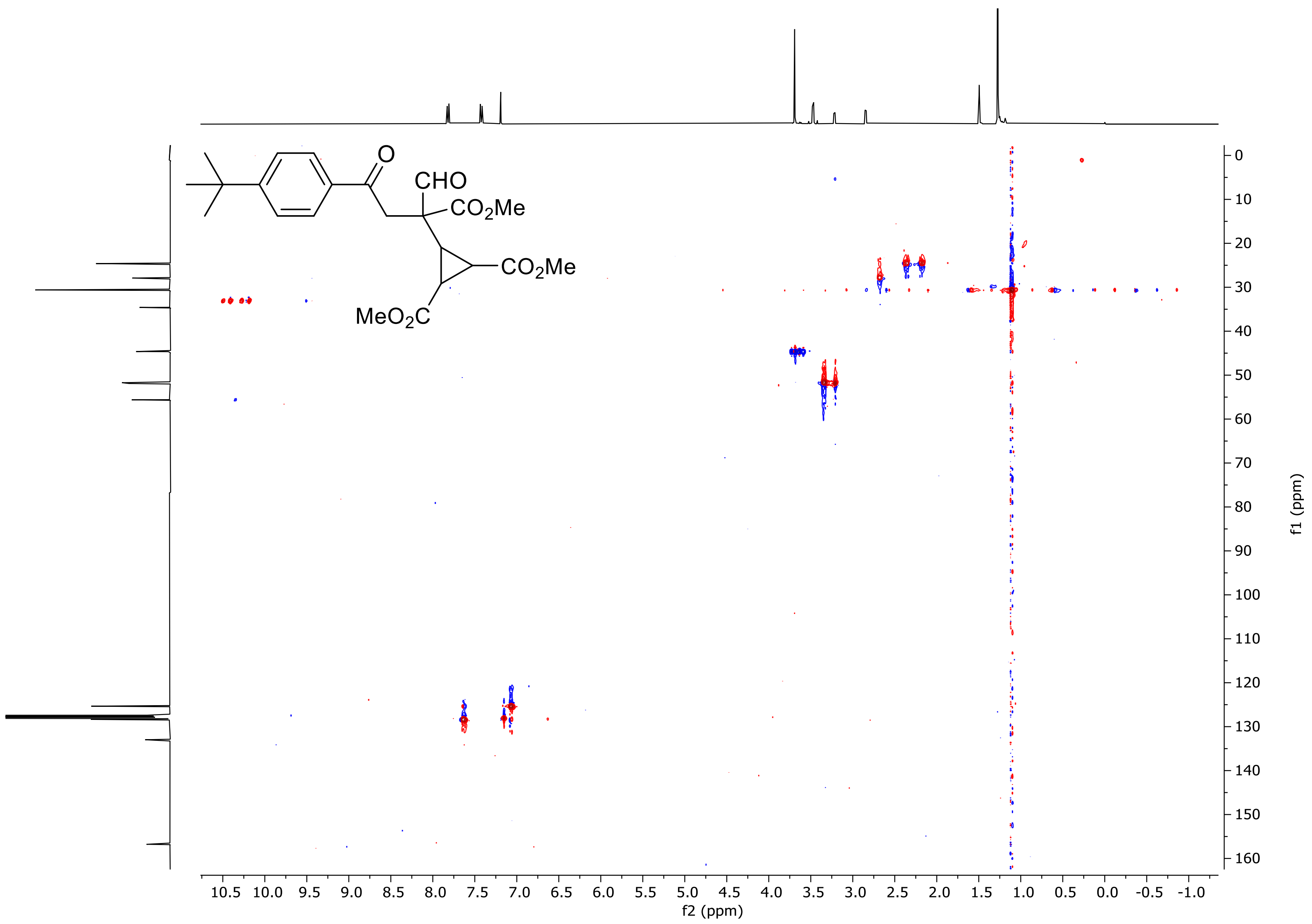

^{1}H NMR (500 MHz, $CDCl_3$, 298 K) spectrum of **D-3n**.

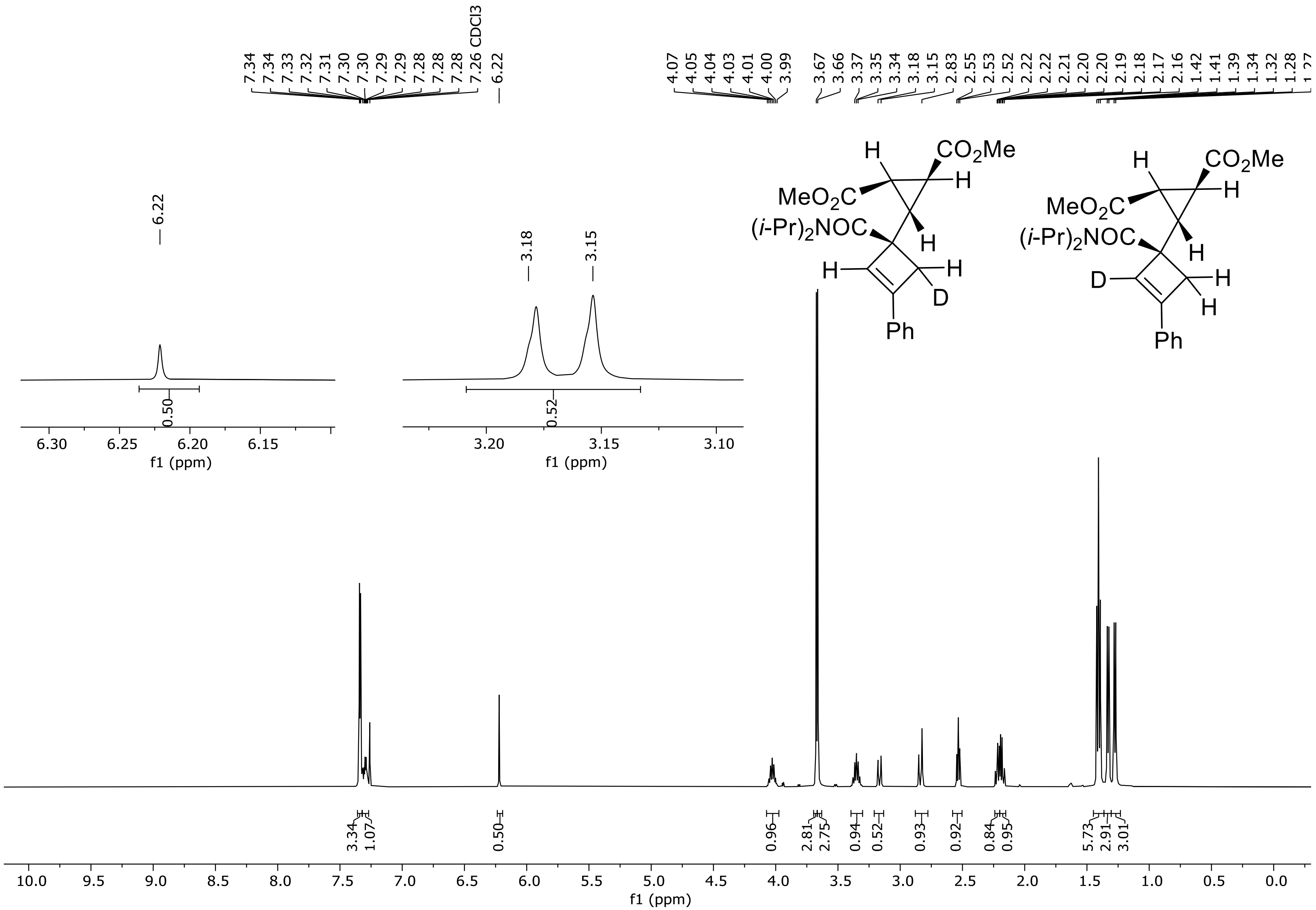

^{13}C NMR (126 MHz, $CDCl_3$, 298 K) spectrum of **D-3n**.

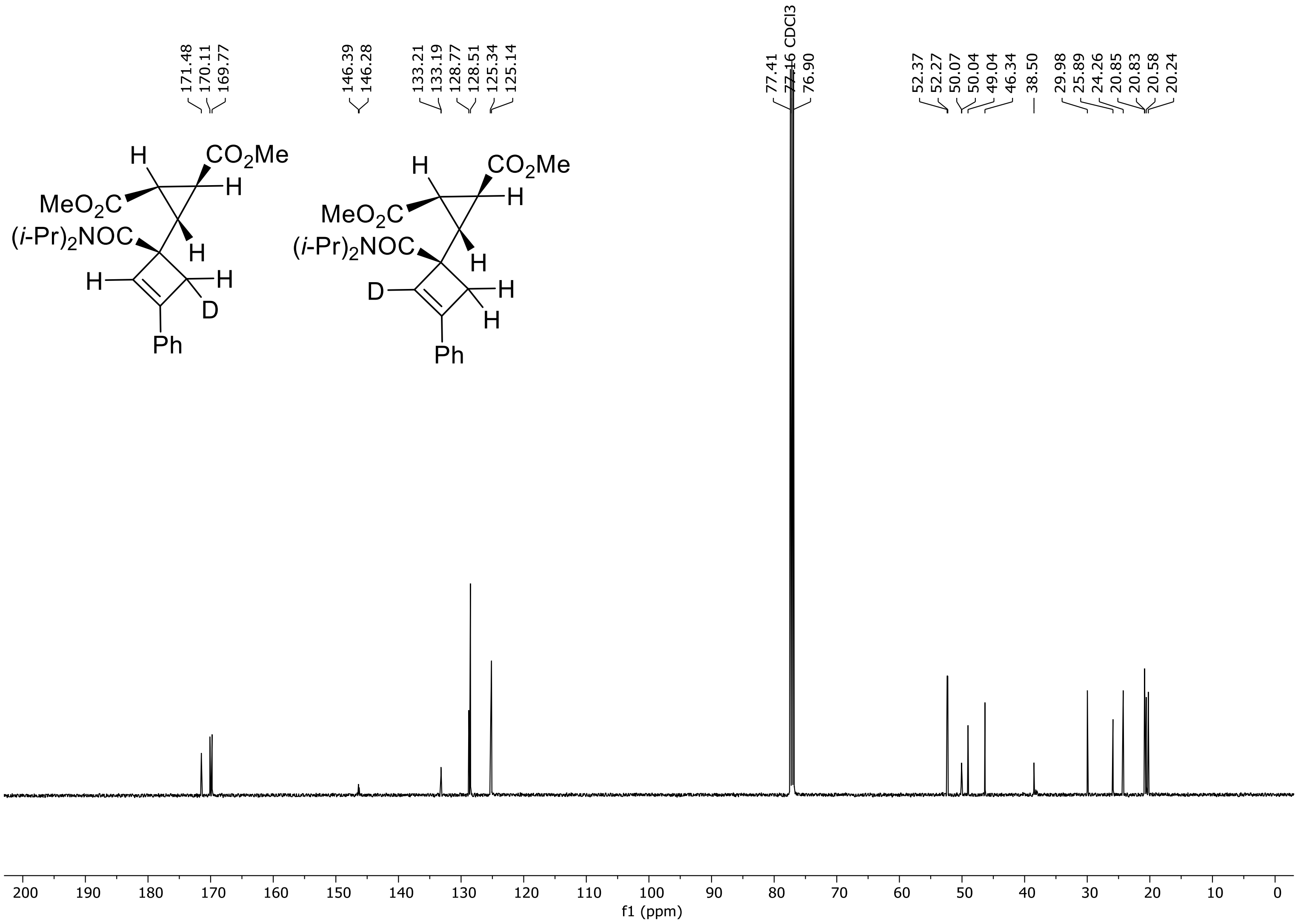

6 Optimization of the Alder-ene reaction (Aryne Part):

To an oven-dried screw-capped test tube equipped with a magnetic stir bar, were added KF and 18-crown-6 in a glove box. Then the BCB **1a** (38 mg, 0.2 mmol, 1.0 equiv) was added outside the glove box under a nitrogen atmosphere, followed by the addition of THF (1.0 mL). To the stirring solution, aryne precursors **4a** was added, and the reaction mixture was allowed to stir at 25 °C for 4 h. After completion of the reaction, all the volatiles were removed *in vacuo,* and the crude residue was pre-adsorbed on silica gel and purified by flash column chromatography (Pet. ether/EtOAc = 97/03) on silica gel to afford the arylated cyclobutene derivative **5a**.

Optimization of the Reaction Conditions[a]

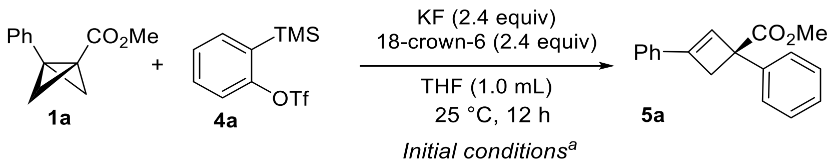

entry	**1a** (equiv)	**4a** (equiv)	fluoride source (equiv)	time	solvent	yield of **5a** (%)[b]
1	1.0	1.2	KF (2.4), 18-crown-6 (2.4)	12 h	THF	85
2	1.0	1.2	CsF (2.4)	12 h	CH_3CN	82
3	1.0	1.5	KF (3.0), 18-crown-6 (3.0)	12 h	THF	96
4	1.0	1.5	KF (3.0), 18-crown-6 (3.0)	4 h	THF	98

[a] Initial conditions: **1a** (0.2 mmol), **4a** (0.24 mmol, KF (2.4 equiv), 18-crown-6 (2.4 equiv), THF (1.0 mL), 25 °C for 12 h. [b] Isolated yield.

7 Synthesis and characterization of Alder-ene products (Aryne Part):

7.1 General reaction procedure d:

To an oven-dried screw-capped test tube equipped with a magnetic stir bar, were added KF (35 mg, 0.6 mmol, 3.0 equiv) and 18-crown-6 (158 mg, 0.6 mmol, 3.0 equiv) in a glove box. Then the BCBs 1 (0.2 mmol, 1.0 equiv) was added outside the glove box under a nitrogen atmosphere, followed by the addition of THF (1.0 mL). To the stirring solution, aryne precursors 4 (0.3 mmol, 1.5 equiv) was added, and the reaction mixture was allowed to stir at 25 °C for 4 h. After completion of the reaction, all the volatiles were removed in vacuo, and the crude residue was pre-adsorbed on silica gel and purified by flash column chromatography (Pet. ether/EtOAc = 97/03) on silica gel to afford the arylated cyclobutene derivatives.

Synthesis and characterization of **5a:**

Synthesized in accordance with *General Procedure d* using BCB-**1a** (38 mg, 0.2 mmol, 1.0 equiv), aryne precursor **4a** (89 mg, 73 µL, 0.3 mmol, 1.5 equiv), KF (35 mg, 0.6 mmol, 3.0 equiv) and 18-crown-6 (158 mg, 0.6 mmol, 3.0 equiv). All volatiles were removed *in vacuo* and the crude compound was purified *via* column chromatography using silica gel and pet. ether/ethyl acetate (97:3 v/v; R_f = 0.4). The desired product **5a** was obtained as a white solid. Yield: 52 mg, 0.2 mmol, 98%.

The 1.0 mmol scale reaction was performed in accordance with General Procedure d using BCB-**1a** (188 mg, 1.0 mmol, 1.0 equiv), aryne precursor **4a** (447 mg, 364 µL, 1.5 mmol, 1.5 equiv), KF (174 mg, 3.0 mmol, 3.0 equiv) and 18-crown-6 (792 mg, 3.0 mmol, 3.0 equiv). All volatiles were removed in vacuo and the crude compound was purified via column chromatography using silica gel and pet. ether/ethyl acetate (97:3 v/v; R_f = 0.4). The desired product **5a** was obtained as a white solid. Yield: 259 mg, 0.98 mmol, 98%.

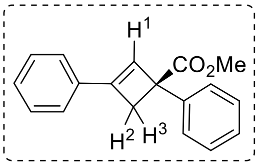

^{1}H NMR (400 MHz, $CDCl_3$, 298 K) δ: 7.50 (d, J = 8.3 Hz, 2H, ArH), 7.46–7.37 (m, 6H, ArH), 7.35–7.29 (m, 2H, ArH), 6.78 (s, 1H, H1), 3.79 (d, J = 12.9 Hz, 1H, H2), 3.73 (s, 3H, CO_2Me), 3.08 (d, J = 12.9 Hz, 1H, H3); ^{13}C NMR (100 MHz, 298 K) δ: 174.1, 147.8, 141.1, 136.6, 128.7, 128.5, 128.4, 127.2, 127.1, 126.8, 125.0, 54.7, 52.4, 41.7; IR υ_{max} (cm^{-1}): 3058, 3027, 2947, 2355, 1953, 1728, 1440, 1248, 1106; HRMS (ESI+) $[M+Na]^+$ calculated for $[C_{18}H_{16}NaO_2]^+$: 287.1043, found: 287.1049.

Synthesis and characterization of **5b:**

Synthesized in accordance with *General Procedure d* using BCB-**1f** (51 mg, 0.2 mmol, 1.0 equiv), aryne precursor **4a** (89 mg, 73 µL, 0.3 mmol, 1.5 equiv), KF (35 mg, 0.6 mmol, 3.0 equiv) and 18-crown-6 (158 mg, 0.6 mmol, 3.0 equiv). All volatiles were removed *in vacuo* and the crude compound was purified *via* column chromatography using silica gel and pet. ether/ethyl acetate (97:3 v/v; R_f = 0.4). The desired product **5b** was obtained as a colorless oil. Yield: 62 mg, 0.19 mmol, 93%.

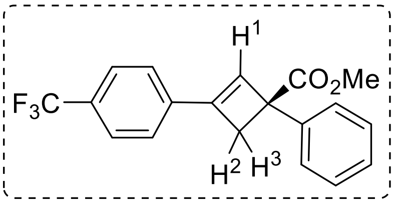

^{1}H NMR (400 MHz, $CDCl_3$, 298 K) δ: 7.61 (d, J = 8.2 Hz, 2H, ArH), 7.50–7.44 (m, 4H, ArH), 7.40–7.36 (m, 2H, ArH), 7.32–7.28 (m, 1H, ArH), 6.87 (s, 1H, H1), 3.77–3.74 (m, 1H, H2), 3.71 (s, 3H, CO_2Me), 3.07 (d, J = 12.9 Hz, 1H, H3); ^{13}C NMR (100 MHz, 298 K) δ: 173.8, 146.5, 140.6, 136.8 (unresolved quartet), 130.4 (q, $J_{C–F}$ = 32.4 Hz), 130.2, 128.6, 127.3, 126.8, 125.6 (q, $J_{C–F}$ = 3.7 Hz), 125.3, 124.2 (q, $J_{C–F}$ = 272.2 Hz), 55.0, 52.6, 41.6; ^{19}F NMR (377 MHz, 298 K) δ: -62.57. IR υ_{max} (cm^{-}

[1]): 3058, 2951, 2356, 1926, 1731, 1613, 1325, 1120; HRMS (ESI+) $[M+H]^+$ calculated for $[C_{19}H_{16}F_3O_2]^+$: 333.1097, found 333.1100.

Synthesis and characterization of **5c:**

Synthesized in accordance with *General Procedure d* using BCB-**1c** (41 mg, 0.2 mmol, 1.0 equiv), aryne precursor **4a** (89 mg, 73 µL, 0.3 mmol, 1.5 equiv), KF (35 mg, 0.6 mmol, 3.0 equiv) and 18-crown-6 (158 mg, 0.6 mmol, 3.0 equiv). All volatiles were removed *in vacuo* and the crude compound was purified *via* column chromatography using silica gel and pet. ether/ethyl acetate (97:3 v/v; R_f = 0.4). The desired product **5c** was obtained as a colorless oil. Yield: 56 mg, 0.2 mmol, 99%.

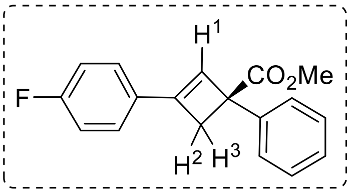

^{1}H NMR (400 MHz, $CDCl_3$, 298 K) δ: 7.45–7.28 (m, 7H, ArH), 7.04 (t, J = 8.6 Hz, 2H, ArH), 6.67 (s, 1H, H1), 3.73-3.70 (m, 4H, H2 and CO_2Me), 3.01 (d, J = 12.9 Hz, 1H, H3). ^{13}C NMR (100 MHz, 298 K) δ: 174.1, 163.0 (d, $J_{C–F}$ = 248.0 Hz), 146.7, 141.1, 130.0 (d, $J_{C–F}$ = 3.3 Hz), 128.5, 127.2, 127.0 (d, $J_{C–F}$ = 8.3 Hz), 126.8, 126.7 (d, $J_{C–F}$ = 2.4 Hz), 115.6 (d, $J_{C–F}$ = 21.7 Hz), 54.6, 52.5, 41.7. ^{19}F NMR (377 MHz, 298 K) δ: -112.05 (ArF). IR υ_{max} (cm^{-1}): 3055, 2950, 2357, 1729, 1597, 1501, 1233, 1105; HRMS (ESI+) $[M+Na]^+$ calculated for $[C_{18}H_{15}FNaO_2]^+$: 305.0948; found 305.0951.

Synthesis and characterization of **5d:**

Synthesized in accordance with *General Procedure d* using BCB-**1d** (44 mg, 0.2 mmol, 1.0 equiv), aryne precursor **4a** (89 mg, 73 µL, 0.3 mmol, 1.5 equiv), KF (35 mg, 0.6 mmol, 3.0 equiv) and 18-crown-6 (158 mg, 0.6 mmol, 3.0 equiv). All volatiles were removed *in vacuo* and the crude compound was purified *via* column chromatography using silica gel and pet. ether/ethyl acetate (97:3 v/v; R_f = 0.4). The desired product **5d** was obtained as a colorless oil. Yield: 56 mg, 0.19 mmol, 93%.

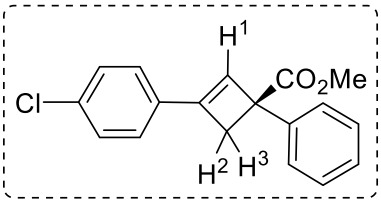

^{1}H NMR (400 MHz, $CDCl_3$, 298 K) δ: 7.46–7.26 (m, 9H, ArH), 6.74 (s, 1H, H1), 3.73–3.70 (m, 1H, H2), 3.71 (s, 3H, CO_2Me), 3.02 (d, J = 12.9 Hz, 1H, H3); ^{13}C NMR (100 MHz, 298 K) δ: 174.0, 146.6, 140.9, 134.4, 132.1, 128.8, 128.5, 127.9, 127.2, 126.8, 126.4, 54.7, 52.5, 41.6; IR υ_{max} (cm^{-1}): 3057, 2950, 2357, 1898, 1729, 1592, 1250, 1098; HRMS (ESI+) $[M+Na]^+$ calculated for $[C_{18}H_{15}ClNaO_2]^+$: 321.0653, found 321.0654.

Synthesis and characterization of **5e:**

Synthesized in accordance with *General Procedure d* using BCB-**1g** (53 mg, 0.2 mmol, 1.0 equiv), aryne precursor **4a** (89 mg, 73 µL, 0.3 mmol, 1.5 equiv), KF (35 mg, 0.6 mmol, 3.0 equiv) and 18-crown-6 (158 mg, 0.6 mmol, 3.0 equiv). All volatiles were removed *in vacuo* and the crude compound was purified *via* column chromatography using silica gel and pet. ether/ethyl acetate (97:3 v/v; R_f = 0.4). The desired product **5e** was obtained as a yellow liquid. Yield: 64 mg, 0.19 mmol, 94%.

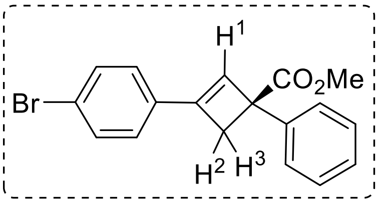

^{1}H NMR (400 MHz, $CDCl_3$, 298 K) δ: 7.50–7.28 (m, 9H, ArH), 6.76 (s, 1H, H1), 3.77–3.72 (m, 1H, H2), 3.74 (s, 3H, CO_2Me), 3.02 (d, *J* = 13.2 Hz, 1H, H3); ^{13}C NMR (100 MHz, 298 K) δ: 173.9, 146.7, 140.8, 131.7, 128.5, 128.1, 127.2, 126.8, 126.6, 125.1, 122.7, 54.8, 52.5, 41.6; IR υ_{max} (cm^{-1}): 3029, 2948, 2356, 1893, 1728, 1589, 1485, 1249; HRMS (ESI+) $[M+Na]^+$ calculated for $[C_{18}H_{15}BrNaO_2]^+$: 365.0148, found 365.0153.

Synthesis and characterization of **5f:**

Synthesized in accordance with *General Procedure d* using BCB-**1l** (40 mg, 0.2 mmol, 1.0 equiv), aryne precursor **4a** (89 mg, 73 µL, 0.3 mmol, 1.5 equiv), KF (35 mg, 0.6 mmol, 3.0 equiv) and 18-crown-6 (158 mg, 0.6 mmol, 3.0 equiv). All volatiles were removed *in vacuo* and the crude compound was purified *via* column chromatography using silica gel and pet. ether/ethyl acetate (97:3 v/v; R_f = 0.4). The desired product **5f** was obtained as a colorless oil. Yield: 52 mg, 0.19 mmol, 94%.

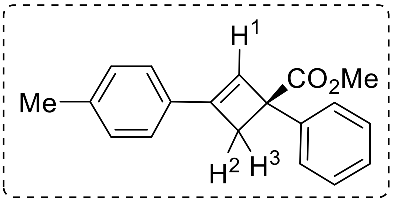

^{1}H NMR (400 MHz, $CDCl_3$, 298 K) δ: 7.46–7.44 (m, 2H, ArH), 7.38–7.26 (m, 5H, ArH), 7.16 (d, *J* = 8.0 Hz, 2H, ArH), 6.66 (s, 1H, H1), 3.74–3.70 (m, 1H, H2), 3.70 (s, 3H, CO_2Me), 3.01 (d, *J* = 13.0 Hz, 1H, H3), 2.36 (s, 3H, ArMe); ^{13}C NMR (100 MHz, 298 K) δ: 174.3, 147.8, 141.3, 138.7, 131.0, 129.2, 128.4, 127.1, 126.8, 126.0, 125.0, 54.7, 52.4, 41.8, 21.5; IR υ_{max} (cm^{-1}): 3025, 2949, 2358, 1902, 1729, 1606, 1248, 1106; HRMS (ESI+) $[M+Na]^+$ calculated for $[C_{19}H_{18}NaO_2]^+$: 301.1199, found 301.1204.

Synthesis and characterization of **5g:**

Synthesized in accordance with *General Procedure d* using BCB-**1m** (54 mg, 0.2 mmol, 1.0 equiv), aryne precursor **4a** (89 mg, 73 µL, 0.3 mmol, 1.5 equiv), KF (35 mg, 0.6 mmol, 3.0 equiv) and 18-crown-6 (158 mg, 0.6 mmol, 3.0 equiv). All volatiles were removed *in vacuo* and the crude compound was purified *via* column chromatography using silica gel and pet.

ether/ethyl acetate (97:3 v/v; R_f = 0.4). The desired product **5g** was obtained as a colorless oil. Yield: 64 mg, 0.18 mmol, 92%.

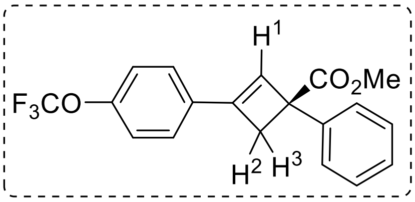

^{1}H NMR (400 MHz, $CDCl_3$, 298 K) δ 7.44–7.35 (m, 6H, ArH), 7.30–7.26 (m, 1H, ArH), 7.20 (d, *J* = 8.2 Hz, 1H, ArH), 6.74 (s, 1H, H1), 3.73–3.70 (m, 4H, H2 and CO_2Me, 3.02 (d, *J* = 12.9 Hz, 1H, H3); ^{13}C NMR (100 MHz, 298 K) δ: 174.0, 149.3 (q, $J_{C–F}$ = 1.8 Hz),146.5, 140.8, 132.0, 128.5, 128.2, 127.3, 126.8, 126.6, 121.1 (unresolved quartet), 120.5 (q, $J_{C–F}$ = 157.2 Hz), 54.7, 52.6, 41.7; ^{19}F NMR (377 MHz, 298 K) δ: -57.81. IR $υ_{max}$ (cm^{-1}): 3059, 2358, 1728, 1501, 1438, 1249, 1207; HRMS (ESI+) $[M+H]^+$ calculated for $[C_{19}H_{16}F_3O_3]^+$: 349.1046, found 349.1053.

Synthesis and characterization of **5h:**

Synthesized in accordance with *General Procedure d* using BCB-**1h** (41 mg, 0.2 mmol, 1.0 equiv), aryne precursor **4a** (89 mg, 73 µL, 0.3 mmol, 1.5 equiv), KF (35 mg, 0.6 mmol, 3.0 equiv) and 18-crown-6 (158 mg, 0.6 mmol, 3.0 equiv). All volatiles were removed *in vacuo* and the crude compound was purified *via* column chromatography using silica gel and pet. ether/ethyl acetate (97:3 v/v; R_f = 0.4). The desired product **5h** was obtained as a colorless oil. Yield: 56 mg, 0.2 mmol, 99%.

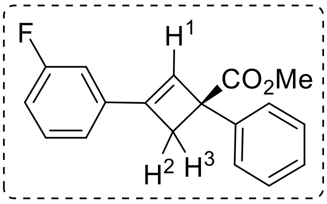

^{1}H NMR (400 MHz, $CDCl_3$, 298 K) δ: 7.45–7.43 (m, 2H, ArH), 7.39–7.26 (m, 4H, ArH), 7.18-7.16 (m, 1H, ArH), 7.10–7.07 (m, 1H, ArH), 7.02-6.97 (m, 1H, ArH), 6.77 (s, 1H, H1), 3.73–3.70 (m, 1H, H2), 3.71 (s, 3H, CO_2Me), 3.02 (d, *J* = 12.8 Hz, 1H, H3); ^{13}C NMR (100 MHz, 298 K) δ: 173.9, 163.1 (d, $J_{C–F}$ = 246.7 Hz), 146.8 (d, $J_{C–F}$ = 2.5 Hz), 140.8, 135.8 (d, $J_{C–F}$ = 7.7 Hz), 130.1 (d, $J_{C–F}$ = 8.3 Hz), 128.8, 128.5, 127.2, 126.8, 120.9 (d, $J_{C–F}$ = 2.9 Hz), 115.6 (d, *J* = 21.4 Hz), 111.9 (d, $J_{C–F}$ = 21.6 Hz), 54.8, 52.5, 41.7; ^{19}F NMR (377 MHz, 298 K) δ: -113.12; IR $υ_{max}$ (cm^{-1}): 3062, 2950, 2358, 1945, 1729, 1584, 1250, 1182; HRMS (ESI+) $[M+Na]^+$ calculated for $[C_{18}H_{15}FNaO_2]^+$: 305.0948, found 305.0952.

Synthesis and characterization of **5i:**

Synthesized in accordance with *General Procedure d* using BCB-**1i** (40 mg, 0.2 mmol, 1.0 equiv), aryne precursor **4a** (89 mg, 73 µL, 0.3 mmol, 1.5 equiv), KF (35 mg, 0.6 mmol, 3.0 equiv) and 18-crown-6 (158 mg, 0.6 mmol, 3.0 equiv). All volatiles were removed *in vacuo* and the crude compound was purified *via* column chromatography using silica gel and pet. ether/ethyl acetate (97:3 v/v; R_f = 0.5). The desired product **5i** was obtained as a colorless oil. Yield: 55 mg, 0.2 mmol, 99%.

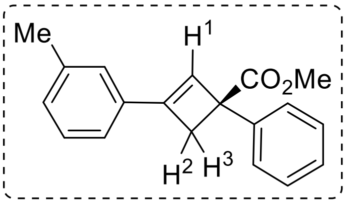

^{1}H NMR (400 MHz, $CDCl_3$, 298 K) δ: 7.47-7.44 (m, 2H, ArH), 7.39–7.34 (m, 2H, ArH), 7.30–7.21 (m, 4H, ArH), 7.13 (d, *J* = 7.0 Hz, 1H, ArH), 6.72 (s, 1H, H1), 3.75-3.72 (m, 1H, H2), 3.71 (s, 3H, CO_2Me), 3.03 (d, *J* = 13.2 Hz, 1H, H3), 2.37 (s, 3H, ArMe); ^{13}C NMR (100 MHz, 298 K) δ: 174.3, 147.9, 141.2, 138.1, 133.6, 129.5, 128.5, 128.4, 127.1, 127.0, 126.8, 125.7, 122.2, 54.7, 52.4, 41.8, 21.5; IR υ_{max} (cm^{-1}): 3026, 2947, 2357, 1729, 1594, 1439, 1246, 1105; HRMS (ESI+) $[M+Na]^+$ calculated for $[C_{19}H_{18}NaO_2]^+$: 301.1199, found 301.1205.

Synthesis and characterization of **5j:**

Synthesized in accordance with *General Procedure d* using BCB-**1j** (44 mg, 0.2 mmol, 1.0 equiv), aryne precursor **4a** (89 mg, 73 µL, 0.3 mmol, 1.5 equiv), KF (35 mg, 0.6 mmol, 3.0 equiv) and 18-crown-6 (158 mg, 0.6 mmol, 3.0 equiv). All volatiles were removed *in vacuo* and the crude compound was purified *via* column chromatography using silica gel and pet. ether/ethyl acetate (97:3 v/v; R_f = 0.4). The desired product **5j** was obtained as a colorless oil. Yield: 55 mg, 0.19 mmol, 94%.

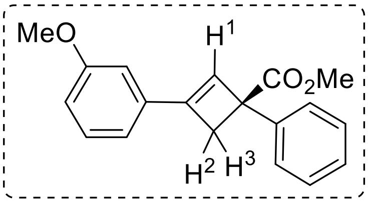

^{1}H NMR (400 MHz, $CDCl_3$, 298 K) δ: 7.47–7.44 (m, 2H, ArH), 7.39–7.34 (m, 2H, ArH), 7.31-7.26 (m, 2H, ArH), 7.03-7.00 (m, 1H, ArH), 6.94–6.93 (m, 1H, ArH), 6.88-6.85 (m, 1H, ArH), 6.74 (s, 1H, H1), 3.83 (s, 3H, OMe), 3.73 (d, *J* = 13.0 Hz, 1H, H2), 3.71 (s, 3H, CO_2Me), 3.02 (d, *J* = 13.0 Hz, 1H, H3); ^{13}C NMR (100 MHz, 298 K) δ: 174.2, 159.8, 147.7, 141.1, 135.0, 129.6, 128.5, 127.6, 127.1, 126.8, 117.7, 114.6, 110.2, 55.4, 54.7, 52.5, 41.8; IR υ_{max} (cm^{-1}): 3057, 2949, 2358, 1942, 1729, 1589, 1247, 1039; HRMS (ESI+) $[M+H]^+$ calculated for $[C_{19}H_{19}O_3]^+$: 295.1329, found 295.1332.

Synthesis and characterization of **5k:**

Synthesized in accordance with *General Procedure d* using BCB-**1k** (40 mg, 0.2 mmol, 1.0 equiv), aryne precursor **4a** (89 mg, 73 µL, 0.3 mmol, 1.5 equiv), KF (35 mg, 0.6 mmol, 3.0 equiv) and 18-crown-6 (158 mg, 0.6 mmol, 3.0 equiv). All volatiles were removed *in vacuo* and the crude compound was purified *via* column chromatography using silica gel and pet. ether/ethyl acetate (97:3 v/v; R_f = 0.5). The desired product **5k** was obtained as a colorless oil. Yield: 52 mg, 0.19 mmol, 95%.

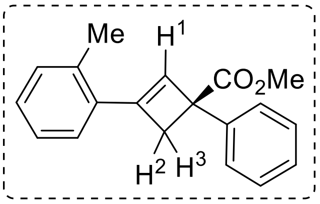

^{1}H NMR (400 MHz, $CDCl_3$, 298 K) δ: 7.49-7.46 (m, 2H, ArH), 7.39–7.36 (m, 2H, ArH), 7.31–7.26 (m, 1H, ArH), 7.22–7.20 (m, 4H, ArH), 6.62 (s, 1H, H1), 3.84 (d, *J* = 12.5 Hz, 1H, H2), 3.72 (s, 3H, CO_2Me), 3.11 (d, *J* = 13.0 Hz, 1H, H3), 2.50 (s, 3H, ArMe); ^{13}C NMR (100

MHz, 298 K) δ: 174.3, 147.7, 141.3, 137.4, 132.2, 130.8, 130.7, 128.5, 128.5, 127.1, 127.0, 126.78, 126.0, 54.9, 52.5, 43.2, 22.0; IR υ_{max} (cm^{-1}): 3022, 2949, 2356, 1729, 1602, 1443, 1252, 1109; HRMS (ESI+) $[M+Na]^+$ calculated for $[C_{19}H_{18}NaO_2]^+$: 301.1199; found 301.1203.

Synthesis and characterization of **5l:**

Synthesized in accordance with *General Procedure d* using BCB-**1o** (53 mg, 0.2 mmol, 1.0 equiv), aryne precursor **4a** (89 mg, 73 µL, 0.3 mmol, 1.5 equiv), KF (35 mg, 0.6 mmol, 3.0 equiv) and 18-crown-6 (158 mg, 0.6 mmol, 3.0 equiv). All volatiles were removed *in vacuo* and the crude compound was purified *via* column chromatography using silica gel and pet. ether/ethyl acetate (97:3 v/v; R_f = 0.5). The desired product **5l** was obtained as a white sticky solid. Yield: 64 mg, 0.19 mmol, 94%.

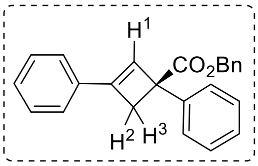

^{1}H NMR (400 MHz, $CDCl_3$, 298 K) δ: 7.48-7.45 (m, 2H, ArH), 7.44–7.29 (m, 11H, ArH), 7.28–7.25 (m, 2H, ArH), 6.77 (s, 1H, H1), 5.17 (s, 2H, CO_2CH_2Ph), 3.78 (d, *J* = 12.9 Hz, 1H, H2), 3.07 (d, *J* = 12.9 Hz, 1H, H3); ^{13}C NMR (100 MHz, 298 K) δ: 173.5, 147.9, 141.1, 136.1, 133.6, 128.7, 128.6, 128.5, 128.5, 128.1, 127.8, 127.1, 127.1, 126.9, 125.1, 66.7, 54.8, 41.6; IR υ_{max} (cm^{-1}): 2916, 2687, 2357, 1724, 1450, 1209, 1020; HRMS (ESI+) $[M+Na]^+$ calculated for $[C_{24}H_{20}NaO_2]^+$: 363.1356**,** found: 363.1360.

Synthesis and characterization of **5m:**

Synthesized in accordance with *General Procedure d* using BCB-**1n** (43 mg, 0.2 mmol, 1.0 equiv), aryne precursor **4a** (89 mg, 73 µL, 0.3 mmol, 1.5 equiv), KF (35 mg, 0.6 mmol, 3.0 equiv) and 18-crown-6 (158 mg, 0.6 mmol, 3.0 equiv). All volatiles were removed *in vacuo* and the crude compound was purified *via* column chromatography using silica gel and pet. ether/ethyl acetate (97:3 v/v; R_f = 0.5). The desired product **5m** was obtained as a white solid. Yield: 58 mg, 0.2 mmol, 99%.

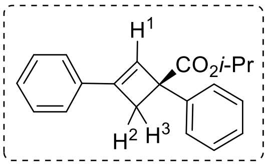

^{1}H NMR (400 MHz, $CDCl_3$, 298 K) δ: 7.46–7.25 (m, 10H, ArH), 6.72 (s, 1H, H1), 5.07–4.98 (m, 1H, CO_2*i*-Pr), 3.70 (d, *J* = 13.0 Hz, 1H, H2), 3.00 (d, *J* = 13.0 Hz, 1H, H3), 1.22 (d, *J* = 6.2 Hz, 3H, CO_2*i*-Pr), 1.18 (d, *J* = 6.2 Hz, 3H, CO_2*i*-Pr); ^{13}C NMR (100 MHz, 298 K) δ: 173.2, 147.7, 141.5, 133.8, 128.6, 128.5, 128.4, 127.6, 127.0, 126.8, 125.1, 68.5, 55.0, 41.7, 21.8, 21.7; IR υ_{max} (cm^{-1}): 3059, 2977, 2930, 2357, 1720, 1449, 1247, 1099; HRMS (ESI+) $[M+Na]^+$ calculated for $[C_{20}H_{20}NaO_2]^+$: 315.1356, found: 315.1362.

Synthesis and characterization of **5n:**

Synthesized in accordance with *General Procedure d* using BCB-**1b** (46 mg, 0.2 mmol, 1.0 equiv), aryne precursor **4a** (89 mg, 73 µL, 0.3 mmol, 1.5 equiv), KF (35 mg, 0.6 mmol, 3.0 equiv) and 18-crown-6 (158 mg, 0.6 mmol, 3.0 equiv). All volatiles were removed *in vacuo* and the crude compound was purified *via* column chromatography using silica gel and pet. ether/ethyl acetate (97:3 v/v; R_f = 0.5). The desired product **5n** was obtained as a white sticky solid. Yield: 60 mg, 0.2 mmol, 98%.

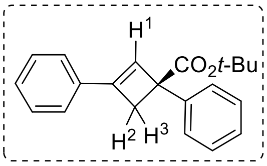

^{1}H NMR (400 MHz, $CDCl_3$, 298 K) δ: 7.46–7.41 (m, 4H, ArH), 7.37–7.25 (m, 6H, ArH), 6.71 (s, 1H, H1), 3.67 (d, *J* = 12.8 Hz, 1H, H2), 2.98 (d, *J* = 12.8 Hz, 1H, H3) 1.43 (s, 9H, CO_2*t*-Bu); ^{13}C NMR (100 MHz, 298 K) δ: 173.0, 147.5, 141.8, 133.9, 128.5, 128.5, 128.3, 127.9, 126.8, 126.7, 125.0, 81.0, 55.6, 41.5, 28.0; IR υ_{max} (cm^{-1}): 3059, 2928, 2357, 1950, 1720, 1488, 1369, 1107; HRMS (ESI+) $[M+Na]^+$ calculated for $[C_{21}H_{22}NaO_2]^+$: 329.1512, found: 329.1518.

Synthesis and characterization of **5o:**

Synthesized in accordance with *General Procedure d* using BCB-**1ab** (49 mg, 0.2 mmol, 1.0 equiv), aryne precursor **4a** (89 mg, 73 µL, 0.3 mmol, 1.5 equiv), KF (35 mg, 0.6 mmol, 3.0 equiv) and 18-crown-6 (158 mg, 0.6 mmol, 3.0 equiv). All volatiles were removed *in vacuo* and the crude compound was purified *via* column chromatography using silica gel and pet. ether/ethyl acetate (80:20 v/v; R_f = 0.3). The desired product **5o** was obtained as a white solid. Yield: 58 mg, 0.18 mmol, 91%.

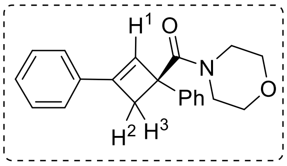

^{1}H NMR (400 MHz, $CDCl_3$, 298 K) δ: 7.45–7.28 (m, 10H, ArH), 6.65 (s, 1H, H1), 3.87-3.60 (m, 5H, H2 and morpholine CH_2), 3.43–3.24 (m, 4H, morpholine CH_2), 2.95 (d, *J* = 13.1 Hz, 1H, H3); ^{13}C NMR (100 MHz, 298 K) δ: 171.5, 148.2, 141.7, 133.5, 129.0, 128.7, 128.5, 127.5, 127.2, 125.3, 125.1, 66.9, 66.1, 55.5, 46.6, 42.5, 42.3; IR υ_{max} (cm^{-1}): 3048, 2909, 2851, 2371, 1620, 1431, 1245, 1113; HRMS (ESI+) $[M+H]^+$ calculated for $[C_{21}H_{22}NO_2]^+$: 320.1645, found: 320.1651.

Synthesis and characterization of **5p:**

Synthesized in accordance with *General Procedure* ***d*** using BCB-**1p** (38 mg, 0.2 mmol, 1.0 equiv), aryne precursor **4a** (89 mg, 73 µL, 0.3 mmol, 1.5 equiv), KF (35 mg, 0.6 mmol, 3.0 equiv) and 18-crown-6 (158 mg, 0.6 mmol, 3.0 equiv). All volatiles were removed *in vacuo* and the crude compound was purified *via* column chromatography using silica gel and pet. ether/ethyl acetate (97:3 v/v; R_f = 0.5). The desired product **5p** was obtained as a colorless liquid. Yield: 46 mg, 0.17 mmol, 87%.

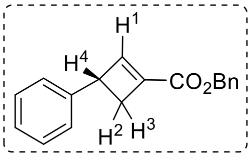

^{1}H NMR (400 MHz, $CDCl_3$, 298 K) δ: 7.45–7.32 (m, 7H, ArH), 7.28–7.24 (m, 3H, ArH), 7.06 (d, *J* = 2.2 Hz, 1H, H1), 5.26 (s, 2H, CO_2CH_2Ph), 3.99-3.97 (m, 1H, H4), 3.27 (dd, *J* = 13.4, 4.6 Hz, 1H, H2), 2.67 (dd, *J* = 13.4, 1.9 Hz, 1H, H3); ^{13}C NMR (100 MHz, 298 K) δ: 162.3, 148.7, 141.0, 138.6, 136.0, 128.7, 128.7, 128.4, 126.9, 66.2, 43.9, 38.7; IR $υ_{max}$ (cm^{-1}): 3031, 2931, 2359, 1714, 1605, 1225, 1110, 960, 743; HRMS (ESI+) $[M+Na]^+$ calculated for $[C_{18}H_{16}NaO_2]^+$: 287.1043, found: 287.1045.

Synthesis and characterization of **5q:**

Synthesized in accordance with *General Procedure d* using BCB-**1aa** (28 mg, 0.2 mmol, 1.0 equiv), aryne precursor **4a** (89 mg, 73 μL, 0.3 mmol, 1.5 equiv), KF (35 mg, 0.6 mmol, 3.0 equiv) and 18-crown-6 (158 mg, 0.6 mmol, 3.0 equiv). All volatiles were removed *in vacuo* and the crude compound was purified *via* column chromatography using silica gel and pet. ether/ethyl acetate (90:10 v/v; R_f = 0.3). The desired product **5q** was obtained as a colorless oil. Yield: 30 mg, 0.14 mmol, 69%.

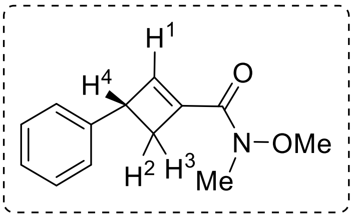

^{1}H NMR (400 MHz, $CDCl_3$, 298 K) δ: 7.33–7.21 (m, 5H, ArH), 6.87 (s, 1H, H1), 3.95 (d, *J* = 5.0 Hz, 1H, H4), 3.73 (s, 3H, OMe), 3.33-3.28 (m, 1H, H2), 3.28 (s, 3H, Me), 2.68 (d, *J* = 13.4 Hz, 1H, H3); ^{13}C NMR (100 MHz, 298 K) δ: 162.9, 146.4, 141.7, 139.5, 128.6, 126.9, 126.8, 61.6, 44.2, 40.0; IR $υ_{max}$ (cm^{-1}): 3020, 2938, 2346, 1661, 1599, 1441, 1250; HRMS (ESI+) [M+H]+ calculated for $[C_{13}H_{16}NO_2]$+: 218.1176, found 218.1181.

Synthesis and characterization of **5r:**

Synthesized in accordance with *General Procedure d* using BCB-**1a** (38 mg, 0.2 mmol, 1.0 equiv), aryne precursor **4b** (100 mg, 0.3 mmol, 1.5 equiv), KF (35 mg, 0.6 mmol, 3.0 equiv) and 18-crown-6 (158 mg, 0.6 mmol, 3.0 equiv). All volatiles were removed *in vacuo* and the crude compound was purified *via* column chromatography using silica gel and pet. ether/ethyl acetate (97:3 v/v; R_f = 0.4). The desired product **5r** was obtained as a colorless liquid. Yield: 56 mg, 0.19 mmol, 96%.

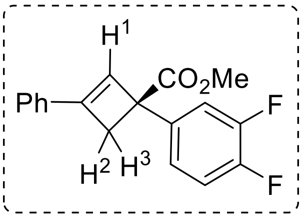

^{1}H NMR (400 MHz, $CDCl_3$, 298 K) δ: 7.41–7.23 (m, 6H, ArH), 7.15–7.09 (m, 2H, ArH), 6.64 (s, 1H, H1), 3.72–3.69 (m, 1H, H2), 3.71 (s, 3H, CO_2Me), 2.96 (d, *J* = 12.9 Hz, 1H); ^{13}C NMR (100 MHz, 298 K) δ: 173.6, 150.3 (dd, $J_{C–F}$ = 248.4 Hz, 12.6 Hz), 149.7 (dd, $J_{C–F}$ = 248.3 Hz, 13.2 Hz), 148.3, 138.2 (dd, $J_{C–F}$ = 5.2 Hz, 4.1 Hz), 133.3, 129.1, 128.6, 126.4, 125.1, 123.0 (dd, $J_{C–F}$ = 6.3 Hz, 3.6 Hz), 116.7 (dd, $J_{C–F}$ = 94.6 Hz, 18.0

Hz), 115.3 (d, $J_{C\text{–}F}$ = 21.5 Hz), 54.0, 52.6, 41.8; ^{19}F NMR (377 MHz, 298 K) δ: –137.4 (d, J = 21.3 Hz, ArF), –140.0 (d, J = 21.3 Hz, ArF); IR $υ_{max}$ (cm^{-1}): 3063, 2951, 2358, 1886, 1731, 1515, 1245, 1110; HRMS (ESI+) $[M+H]^+$ calculated for $[C_{18}H_{15}F_2O_2]^+$: 301.1035, found 301.1038.

Synthesis and characterization of **5s:**

Synthesized in accordance with *General Procedure d* using BCB-**1a** (38 mg, 0.2 mmol, 1.0 equiv), aryne precursor **4c** (98 mg, 0.3 mmol, 1.5 equiv), KF (35 mg, 0.6 mmol, 3.0 equiv) and 18-crown-6 (158 mg, 0.6 mmol, 3.0 equiv). All volatiles were removed *in vacuo* and the crude compound was purified *via* column chromatography using silica gel and pet. ether/ethyl acetate (97:3 v/v; R_f = 0.5). The desired product **5s** was obtained as a colorless oil. Yield: 57 mg, 0.2 mmol, 99%.

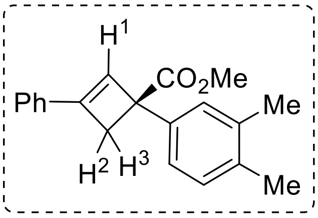

^{1}H NMR (400 MHz, $CDCl_3$, 298 K) δ: 7.43–7.28 (m, 5H, ArH), 7.22–7.13 (m, 3H, ArH), 6.73 (s, 1H, H1), 3.74–3.71 (m, 1H, H2), 3.71 (s, 3H, CO_2Me), 3.02 (d, J = 12.9 Hz, 1H, H3), 2.29 (s, 3H, ArMe), 2.27 (s, 3H, ArMe); ^{13}C NMR (100 MHz, 298 K) δ: 174.4, 147.6, 138.6, 136.7, 135.5, 133.8, 129.7, 128.6, 128.5, 128.0, 127.4, 125.0, 124.2, 54.4, 52.4, 41.7, 20.0, 19.5; IR $υ_{max}$ (cm^{-1}): 3020, 2946, 2358, 1953, 1728, 1497, 1443, 1234, 1106; HRMS (ESI+) $[M+Na]^+$ calculated for $[C_{20}H_{20}NaO_2]^+$: 315.1356, found 315.1363.

Synthesis and characterization of **5t:**

Synthesized in accordance with *General Procedure d* using BCB-**1a** (38 mg, 0.2 mmol, 1.0 equiv), aryne precursor **4d** (107 mg, 0.3 mmol, 1.5 equiv), KF (35 mg, 0.6 mmol, 3.0 equiv) and 18-crown-6 (158 mg, 0.6 mmol, 3.0 equiv). All volatiles were removed *in vacuo* and the crude compound was purified *via* column chromatography using silica gel and pet. ether/ethyl acetate (90:10 v/v; R_f = 0.3). The desired product **5t** was obtained as a white solid. Yield: 58 mg, 0.18 mmol, 89%.

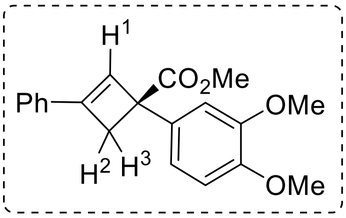

^{1}H NMR (400 MHz, $CDCl_3$, 298 K) δ: 7.41–7.27 (m, 5H, ArH), 6.98–6.96 (m, 2H, ArH), 6.84 (d, J = 8.6 Hz, 1H, ArH), 6.70 (s, 1H, H1), 3.90 (s, 3H, ArOMe), 3.87 (s, 3H, ArOMe), 3.72–3.68 (m, 4H, H2 and CO_2Me), 3.00 (d, J = 13.0 Hz, 1H, H3); ^{13}C NMR (100 MHz, 298 K) δ: 174.4, 148.9, 148.2, 147.8, 133.8, 133.6, 128.7, 128.5, 127.1, 125.0, 119.1, 110.0, 110.1, 56.0, 56.0, 54.3, 52.4, 41.7; IR $υ_{max}$ (cm^{-1}): 3007, 2947, 2837, 2358, 1726, 1451, 1246, 1153; HRMS (ESI+) $[M+Na]^+$ calculated for $[C_{20}H_{20}NaO_4]^+$: 347.1254, found 347.1257.

Synthesis and characterization of **5u:**

Synthesized in accordance with *General Procedure d* using BCB-**1a** (38 mg, 0.2 mmol, 1.0 equiv), aryne precursor **4e** (101 mg, 0.3 mmol, 1.5 equiv), KF (35 mg, 0.6 mmol, 3.0 equiv) and 18-crown-6 (158 mg, 0.6 mmol, 3.0 equiv). All volatiles were removed *in vacuo* and the crude compound was purified *via* column chromatography using silica gel and pet. ether/ethyl acetate (97:3 v/v; R_f = 0.4). The desired product **5u** was obtained as a sticky white solid. Yield: 60 mg, 0.2 mmol, 98%.

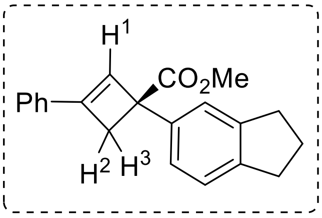

^{1}H NMR (400 MHz, $CDCl_3$, 298 K) δ: 7.43–7.40 (m, 2H, ArH), 7.38–7.28 (m, 4H, ArH), 7.23 (d, *J* = 1.3 Hz, 2H, ArH), 6.73 (s, 1H, H1), 3.73 (d, *J* = 12.8 Hz, 1H, H2), 3.71 (s, 3H, CO_2Me), 3.02 (d, *J* = 12.8 Hz, 1H, H3), 2.95–2.89 (m, 4H, $ArCH_2$), 2.09 (quint, *J* = 7.4 Hz, 2H, indane CH_2); ^{13}C NMR (100 MHz, 298 K) δ: 174.5, 147.6, 144.6, 143.2, 139.1, 133.8, 128.6, 128.5, 127.5, 125.0, 124.7, 124.3, 122.8, 54.7, 52.4, 41.8, 33.0, 32.7, 25.6; IR υ_{max} (cm^{-1}): 3019, 2947, 2356, 1728, 1579, 1438, 1236, 1104; HRMS (ESI+) $[M+Na]^+$ calculated for $[C_{21}H_{20}NaO_2]^+$: 327.1356, found 327.1359.

Synthesis and characterization of **5v:**

Synthesized in accordance with *General Procedure d* using BCB-**1a** (38 mg, 0.2 mmol, 1.0 equiv), aryne precursor **4f** (103 mg, 0.3 mmol, 1.5 equiv), KF (35 mg, 0.6 mmol, 3.0 equiv) and 18-crown-6 (158 mg, 0.6 mmol, 3.0 equiv). All volatiles were removed *in vacuo* and the crude compound was purified *via* column chromatography using silica gel and pet. ether/ethyl acetate (95:5 v/v; R_f = 0.3). The desired product **5v** was obtained as a yellow liquid. Yield: 61 mg, 0.2 mmol, 98%.

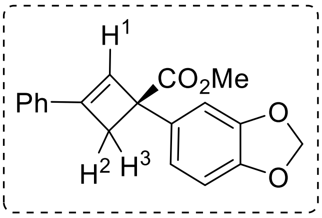

^{1}H NMR (400 MHz, $CDCl_3$, 298 K) δ: 7.40–7.28 (m, 5H, ArH), 6.94 (s, 1H, ArH), 6.89 (d, J = 7.8, 1H, ArH), 6.78 (d, *J* = 8.6 Hz, 1H, ArH), 6.66 (s, 1H, H1), 5.95 (s, 2H, sesamol CH_2), 3.70–3.67 (m, 4H, H2 and CO_2Me), 2.96 (d, *J* = 12.86 Hz, 1H, H3); ^{13}C NMR (100 MHz, 298 K) δ: 174.2, 147.8, 147.8, 146.7, 135.1, 133.6, 128.8, 128.5, 127.1, 125.1, 120.1, 108.2, 107.6, 101.2, 54.4, 52.5, 41.7; IR υ_{max} (cm^{-1}): 3018, 2893, 2357, 1727, 1610, 1491, 1241, 1037; HRMS (ESI+) $[M+Na]^+$ calculated for $[C_{19}H_{16}NaO_4]^+$: 331.0941, found 331.0946.

Synthesis and characterization of **5w:**

Synthesized in accordance with *General Procedure d* using BCB-**1a** (38 mg, 0.2 mmol, 1.0 equiv), aryne precursor **4g** (98 mg, 0.3 mmol, 1.5 equiv), KF (35 mg, 0.6 mmol, 3.0 equiv) and 18-crown-6 (158 mg, 0.6 mmol, 3.0 equiv). All volatiles were removed *in vacuo* and the crude compound was purified *via* column chromatography using silica gel and pet. ether/ethyl acetate (97:3 v/v; R_f = 0.4). The desired product **5w** was obtained as a white solid. Yield: 57 mg, 0.19 mmol, 97%.

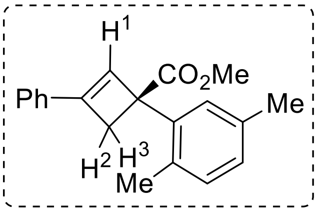

^{1}H NMR (400 MHz, $CDCl_3$, 298 K) δ: 7.43 (d, J = 7.3 Hz, 2H, ArH), 7.38–7.30 (m, 3H, ArH), 7.17 (s, 1H, ArH), 7.08–7.01 (m, 2H, ArH), 6.80 (s, 1H, H1), 3.90 (d, *J* = 12.6 Hz, 2H, H2), 3.68 (s, 3H, CO_2Me), 2.84 (d, *J* = 12.6 Hz, 1H, H3), 2.34 (s, 3H, $ArCH_3$), 2.26 (s, 3H, $ArCH_3$); ^{13}C NMR (100 MHz, 298 K) δ: 174.4, 147.8, 139.4, 135.2, 133.7, 133.2, 130.8, 128.8, 128.6, 128.5, 128.1, 127.1, 125.1, 54.8, 52.6, 40.1, 21.2, 19.2; IR υ_{max} (cm^{-1}): 2926, 2866, 2356, 1727, 1443, 123, 1107; HRMS (ESI+) $[M+Na]^+$ calculated for $[C_{20}H_{20}NaO_2]^+$: 315.1356, found 315.1358.

Synthesis and characterization of **5x:**

Synthesized in accordance with *General Procedure d* using BCB-**1a** (38 mg, 0.2 mmol, 1.0 equiv), aryne precursor **4h** (99 mg, 0.3 mmol, 1.5 equiv), KF (35 mg, 0.6 mmol, 3.0 equiv) and 18-crown-6 (158 mg, 0.6 mmol, 3.0 equiv). All volatiles were removed *in vacuo* and the crude compound was purified *via* column chromatography using silica gel and pet. ether/ethyl acetate (90:10 v/v; R_f = 0.5). The desired product **5x** was obtained as a colorless liquid. Yield: 58 mg, 0.19 mmol, 97%.

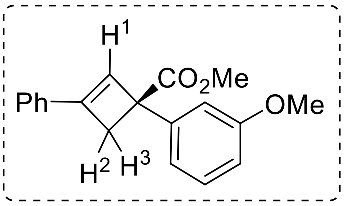

^{1}H NMR (400 MHz, $CDCl_3$, 298 K) δ: 7.41–7.26 (m, 6H, ArH), 7.04–6.99 (m, 2H, ArH), 7.23 (d, *J* = 8.0 Hz, 1H, ArH), 6.70 (s, 1H, H1), 3.82 (s, 3H, ArOMe), 3.74–3.70 (m, 4H, H2 and CO_2Me), 3.02 (d, *J* = 13.0 Hz, 1H, H3); ^{13}C NMR (100 MHz, 298 K) δ: 174.1, 159.7, 147.9, 142.7, 133.6, 129.5, 128.7, 128.5, 127.0, 125.1, 119.2, 112.8, 112.4, 55.4, 54.7, 52.5, 41.7; IR υ_{max} (cm^{-1}): 3010, 2948, 2837, 2358, 1729, 1594, 1438, 1241; HRMS (ESI+) $[M+Na]^+$ calculated for $[C_{19}H_{18}NaO_3]^+$: 317.1148, found 317.1154.

Synthesis and characterization of **5y** and **5y’**:

Synthesized in accordance with *General Procedure d* using BCB-**1a** (38 mg, 0.2 mmol, 1.0 equiv), aryne precursor **4i** (94 mg, 0.3 mmol, 1.5 equiv), KF (35 mg, 0.6 mmol, 3.0 equiv) and 18-crown-6 (158 mg, 0.6 mmol, 3.0 equiv). All volatiles were removed *in vacuo* and the crude compound was purified *via* column chromatography using silica gel and pet. ether/ethyl acetate (97:3 v/v; R_f = 0.4). The inseparable (1:1) regioisomeric mixture of **5y** and **5y’** was obtained as a colorless liquid. Yield: 56 mg, 0.19 mmol, 93%.

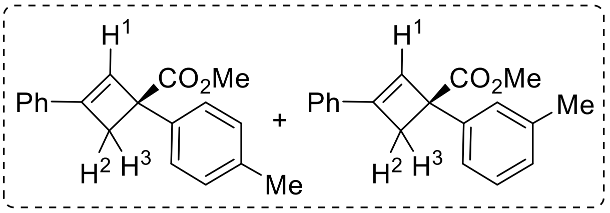

^{1}H NMR (400 MHz, $CDCl_3$, 298 K) of one isomer δ: 7.43–7.09 (m, 9H, ArH), 6.73 (s, 1H, H1), 3.75–3.70 (m, 4H, H2 and CO_2Me), 3.02 (d, *J* = 13.0 Hz, 1H, H3), 2.38 (s, 3H, ArMe); ^{13}C NMR (100 MHz, 298 K) of the isomer δ: 174.4, 147.7, 138.1, 136.8, 133.7, 128.7, 128.5, 128.4, 127.5, 127.3, 125.1, 123.9, 54.7, 52.5, 41.7, 21.6; Representative peaks for another isomer: ^{1}H NMR (400 MHz, $CDCl_3$, 298 K) δ: 6.72 (s, 1H, H1), 3.01 (d, *J* = 13.0 Hz, 1H, H3), 2.36 (s, 3H, ArMe); ^{13}C NMR (100 MHz, 298 K) δ: 174.3, 138.2, 125.0, 54.4, 52.4, 41.7, 21.2; IR $υ_{max}$ (cm^{-1}): 3025, 2948, 2357, 1728, 1601, 1440, 1242, 1106; HRMS (ESI+) $[M+Na]^+$ calculated for $[C_{19}H_{18}NaO_2]^+$: 301.1199, found 301.1204.

Synthesis and characterization of **5z** and **5z’**:

Synthesized in accordance with *General Procedure d* using BCB-**1a** (38 mg, 0.2 mmol, 1.0 equiv), aryne precursor **4j** (100 mg, 0.3 mmol, 1.5 equiv), KF (35 mg, 0.6 mmol, 3.0 equiv) and 18-crown-6 (158 mg, 0.6 mmol, 3.0 equiv). All volatiles were removed *in vacuo* and the crude compound was purified *via* column chromatography using silica gel and pet. ether/ethyl acetate (97:3 v/v; R_f = 0.4). The inseparable (1:1.5) regioisomeric mixture of **5z** and **5z’** was obtained as a colorless liquid. Yield: 47 mg, 0.16 mmol, 79%.

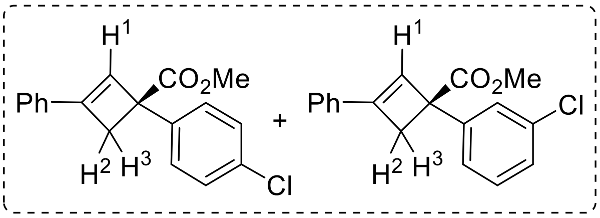

^{1}H NMR (400 MHz, $CDCl_3$, 298 K) of major isomer δ: 7.42–7.23 (m, 9H, ArH), 6.67 (s, 1H, H1), 3.74–3.70 (m, 4H, H2 and CO_2Me), 2.96 (d, *J* = 13.0 Hz, 1H, H3); ^{13}C NMR (100 MHz, 298 K) of major isomer δ: 173.9, 148.1, 139.7, 133.4, 133.1, 129.7, 128.9, 128.6, 128.3, 127.2, 126.7, 125.1, 54.2, 52.6, 41.8; Representative peaks for minor isomer: ^{1}H NMR (400 MHz, $CDCl_3$, 298 K) δ: 2.99 (d, *J* = 13.0 Hz, 1H, H3); ^{13}C NMR (100 MHz, 298 K) δ: 173.7, 148.2, 143.2, 134.4, 129.0, 128.6, 126.5, 54.4, 52.4, 41.7; IR $υ_{max}$ (cm^{-1}): 3026, 2949, 2356, 1729, 1477, 1250, 1103; HRMS (ESI+) $[M+Na]^+$ calculated for $[C_{18}H_{15}ClNaO_2]^+$: 321.0653, found 321.0655.

Synthesis and characterization of **5aa:**

Synthesized in accordance with *General Procedure d* using BCB-**1a** (38 mg, 0.2 mmol, 1.0 equiv), aryne precursor **4k** or **4l** (104 mg, 0.3 mmol, 1.5 equiv), KF (35 mg, 0.6 mmol, 3.0 equiv) and 18-crown-6 (158 mg, 0.6 mmol, 3.0 equiv). All volatiles were removed *in vacuo* and the crude compound was purified *via* column chromatography using silica gel and pet. ether/ethyl acetate (97:3 v/v; R_f = 0.4). The desired product **5aa** was obtained as a colorless oil. Yield: 61 mg, 0.19 mmol, 97% from **4k** or Yield: 60 mg, 0.19 mmol, 97% from **4l**.

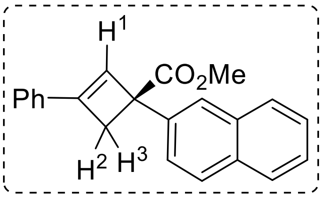

^{1}H NMR (400 MHz, $CDCl_3$, 298 K) δ: 7.87–7.82 (m, 4H, ArH), 7.61–7.58 (m, 1H, ArH), 7.51–7.44 (m, 4H, ArH), 7.40–7.28 (m, 3H, ArH), 6.85 (s, 1H, H1), 3.82 (d, *J* = 12.9 Hz, 1H, H2), 3.72 (s, 3H, CO_2Me), 3.11 (d, *J* = 12.9 Hz, 1H, H3); ^{13}C NMR (100 MHz, 298 K) δ: 174.2, 148.0, 138.6, 133.6, 133.3, 132.5, 128.8, 128.6, 128.3, 128.0, 127.7, 127.2, 126.3, 126.0, 125.5, 125.1, 125.0, 54.9, 52.5, 41.7; IR υ_{max} (cm^{-1}): 3055, 2950, 2358, 1947, 1728, 1593, 1245, 1108; HRMS (ESI+) $[M+H]^+$ calculated for $[C_{22}H_{19}O_2]^+$: 315.1380, found 315.1381.

Synthesis and characterization of **5ab:**

Synthesized in accordance with *General Procedure d* using BCB-**1a** (38 mg, 0.2 mmol, 1.0 equiv), aryne precursor **4m** (120 mg, 0.3 mmol, 1.5 equiv), KF (35 mg, 0.6 mmol, 3.0 equiv) and 18-crown-6 (158 mg, 0.6 mmol, 3.0 equiv). All volatiles were removed *in vacuo* and the crude compound was purified *via* column chromatography using silica gel and pet. ether/ethyl acetate (97:3 v/v; R_f = 0.4). The desired product **5ab** was obtained as a white solid. Yield: 71 mg, 0.19 mmol, 97%.

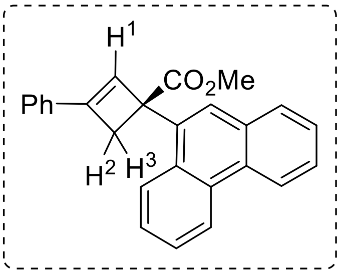

^{1}H NMR (400 MHz, $CDCl_3$, 298 K) δ: 8.76 (d, *J* = 7.8 Hz, 1H, ArH), 8.67 (d, *J* = 8.2 Hz, 1H, ArH), 8.04 (d, *J* = 7.6 Hz, 1H, ArH), 7.90 (d, *J* = 7.7 Hz, 1H, ArH), 7.78 (s, 1H, ArH), 7.70-7.57 (m, 4H, ArH), 7.48–7.46 (m, 2H, ArH), 7.39–7.30 (m, 3H, ArH), 7.00 (s, 1H, H1), 4.15 (d, J = 12.8 Hz, 1H, H2), 3.62 (s, 3H, CO_2Me), 3.03 (d, J = 12.9 Hz, 1H, H3); ^{13}C NMR (100 MHz, 298 K) δ: 174.7, 149.0, 136.1, 133.6, 131.6, 131.0, 130.6, 130.2, 128.9, 128.8, 128.6, 127.0, 127.0, 126.9, 126.9, 126.8, 126.5, 125.2, 124.9, 123.5, 122.6, 55.0, 52.8, 40.3; IR υ_{max} (cm^{-1}): 3430, 2925, 2358, 1958, 1722, 1642, 1438, 1244; HRMS (ESI+) $[M+Na]^+$ calculated for $[C_{26}H_{20}NaO_2]^+$: 387.1356; found 387.1362.

Synthesis and characterization of **7a:**

Synthesized in accordance with *Literature Procedure*[2] using BCB-**1a** (38 mg, 0.2 mmol, 1.0 equiv), $Ga(OTf)_3$ (5 mg, 5 mol %), in 1.2 mL THF for 20 min. All volatiles were removed *in vacuo* and the crude compound was purified *via* column chromatography using silica gel and pet. ether/ethyl acetate (97:3 v/v; R_f = 0.6). The desired product **7a** was obtained as a colorless oil. Yield: 21 mg, 0.11 mmol, 58%.

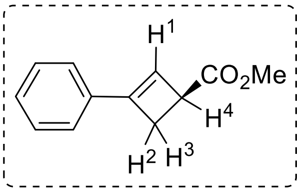

^{1}H NMR (400 MHz, $CDCl_3$, 298 K) δ: 7.38–7.28 (m, 5H, ArH), 6.29 (s, 1H, H1), 3.72 (s, 3H, CO_2Me), 3.67–3.66 (m, 1H, H4), 3.10-3.00 (m, 2H, H2, H3); ^{13}C NMR (100 MHz, 298 K) δ: 174.1, 148.4, 133.9, 128.5, 124.8, 124.6, 52.0, 41.4, 32.8.

8 Mechanistic Experiments:

To get insight into the mechanism of the Alder-ene reaction of BCB and aryne, we performed a few experiments. As the acid-catalyzed rearrangement of BCB **1a** to cyclobutene **7a** is a known process,[12] there was a possibility that the arylated cyclobutene **5a** was forming *insitu via* the rearrangement-arylation sequence under optimized reaction conditions.

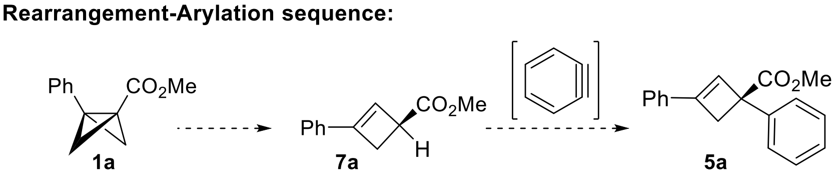

So to rule out this possibility, the BCB **1a** (38 mg, 0.2 mmol, 1.0 equiv), KF (35 mg, 0.6 mmol, 3.0 equiv), and 18-crown-6 (158 mg, 0.6 mmol, 3.0 equiv) in THF (1.0 mL) were stirred under optimized reaction conditions in the absence of the aryne precursor **4a**, and even after 12 h, the BCB **1a** was intact, and the rearranged cyclobutene **7a** was not observed.

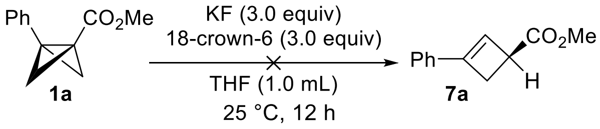

Moreover, the cyclobutene **7a** was separately prepared following the literature procedure[2] and reacted with aryne precursor **4a** under the optimized reaction conditions:

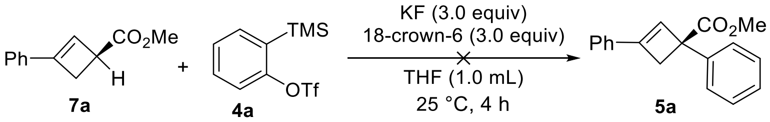

To an oven-dried screw-capped test tube equipped with a magnetic stir bar, were added KF (18 mg, 0.3 mmol, 3.0 equiv) and 18-crown-6 (79 mg, 0.3 mmol, 3.0 equiv) in a glove box.

Then THF (0.5 mL) and cyclobutene **7a** (19 mg, 0.1 mmol, 1.0 equiv) were added outside the glove box under a nitrogen atmosphere. To the stirring solution, aryne precursors **4a** (44 mg, 36 μL, 0.15 mmol, 1.5 equiv) was added, and the reaction mixture was allowed to stir at 25 °C for 4 h. After 4 h, the arylated cyclobutene **5a** was not formed (a complex mixture of products was observed).

These experiments indicate that the present aryne reaction is not proceeding via the rearrangement-arylation sequence and, indeed, an ene process.

Further, the Aryne-ene reaction could also operate via concerted, stepwise radical, or stepwise ionic pathways. So to explore these possibilities, the reaction was carried out in the presence of radical scavengers like TEMPO and BHT or in the presence of D_2O.

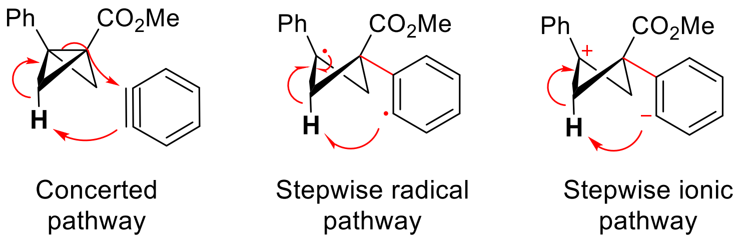

Reaction in the presence of TEMPO

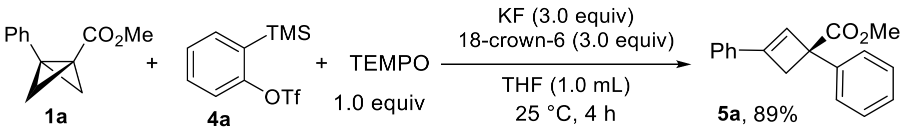

To an oven-dried screw-capped test tube equipped with a magnetic stir bar, were added KF (35 mg, 0.6 mmol, 3.0 equiv) and 18-crown-6 (158 mg, 0.6 mmol, 3.0 equiv) in a glove box. Then the BCB **1a** (38 mg, 0.2 mmol, 1.0 equiv) and TEMPO (31 mg, 0.2 mmol, 1.0 equiv) was added outside the glove box under a nitrogen atmosphere, followed by the addition of THF (1.0 mL). To the stirring solution, aryne precursors **4a** (89 mg, 73 μL, 0.3 mmol, 1.5 equiv) was added and the reaction mixture was allowed to stir at 25 °C for 4 h. After 4 h, the cyclobutene **5a** was formed in 89% (^{1}H NMR analysis of the crude reaction mixture using CH_2Br_2 as internal standard).

Reaction in the presence of BHT

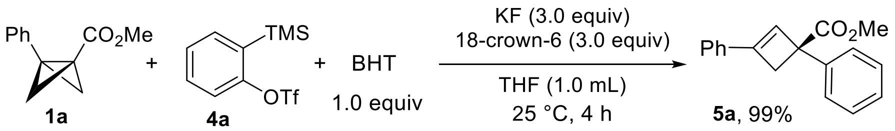

To an oven-dried screw-capped test tube equipped with a magnetic stir bar, were added KF (35 mg, 0.6 mmol, 3.0 equiv) and 18-crown-6 (158 mg, 0.6 mmol, 3.0 equiv) in a glove box. Then the BCB **1a** (38 mg, 0.2 mmol, 1.0 equiv) and BHT (44 mg, 0.2 mmol, 1.0 equiv) were

added outside the glove box under a nitrogen atmosphere, followed by the addition of THF (1.0 mL). To the stirring solution, aryne precursors **4a** (89 mg, 73 µL, 0.3 mmol, 1.5 equiv) was added, and the reaction mixture was allowed to stir at 25 °C for 4 h. After 4 h, the cyclobutene **5a** was formed in 99% (^{1}H NMR analysis of the crude reaction mixture using CH_2Br_2 as internal standard).

These reactions indicate that the present reaction is not proceeding via the radical pathway.

Reaction in the presence of D_2O

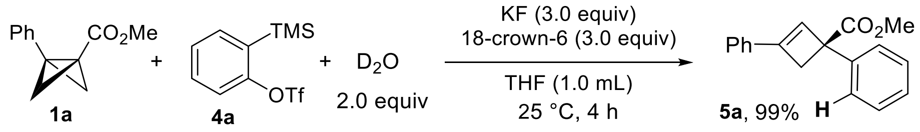

To an oven-dried screw-capped test tube equipped with a magnetic stir bar, were added KF (35 mg, 0.6 mmol, 3.0 equiv) and 18-crown-6 (158 mg, 0.6 mmol, 3.0 equiv) in a glove box. Then the BCB **1a** (38 mg, 0.2 mmol, 1.0 equiv) was added outside the glove box under a nitrogen atmosphere, followed by the addition of THF (1.0 mL) and D_2O (8 mg, 0.4 mmol, 2.0 equiv). To the stirring solution, aryne precursors **4a** (89 mg, 73 µL, 0.3 mmol, 1.5 equiv) was added, and the reaction mixture was allowed to stir at 25 °C for 4 h. After 4 h, the cyclobutene **5a** was isolated in 99% yield without any deuterium incorporation at the C2-position.

This reaction indicates that the present ene reaction is likely proceeding via a concerted process

9 NMR Spetcra (Aryne Part):

^{1}H NMR (400 MHz, $CDCl_3$, 298 K) spectrum of **5a**.

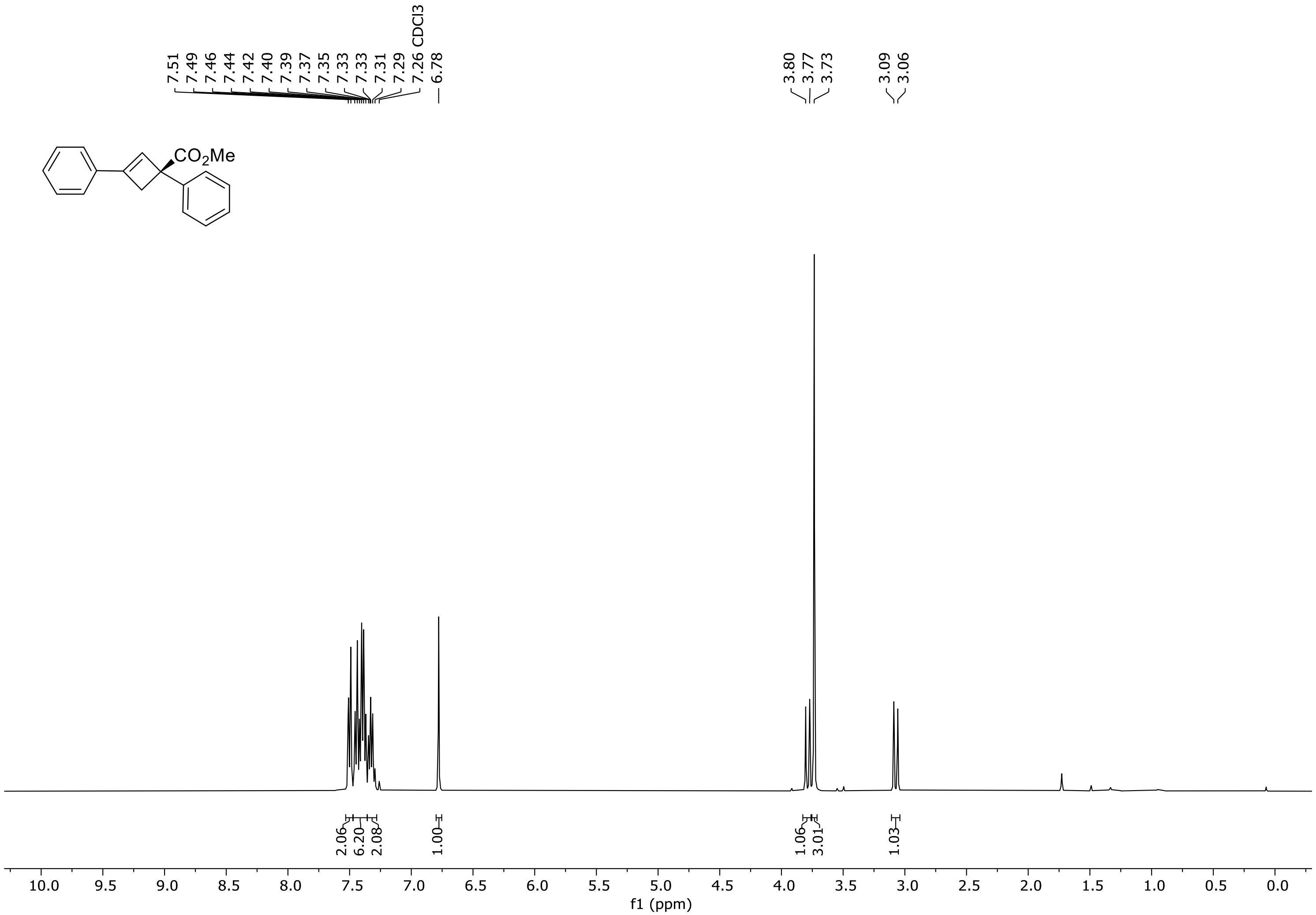

^{13}C NMR (100 MHz, $CDCl_3$, 298 K) spectrum of **5a**.

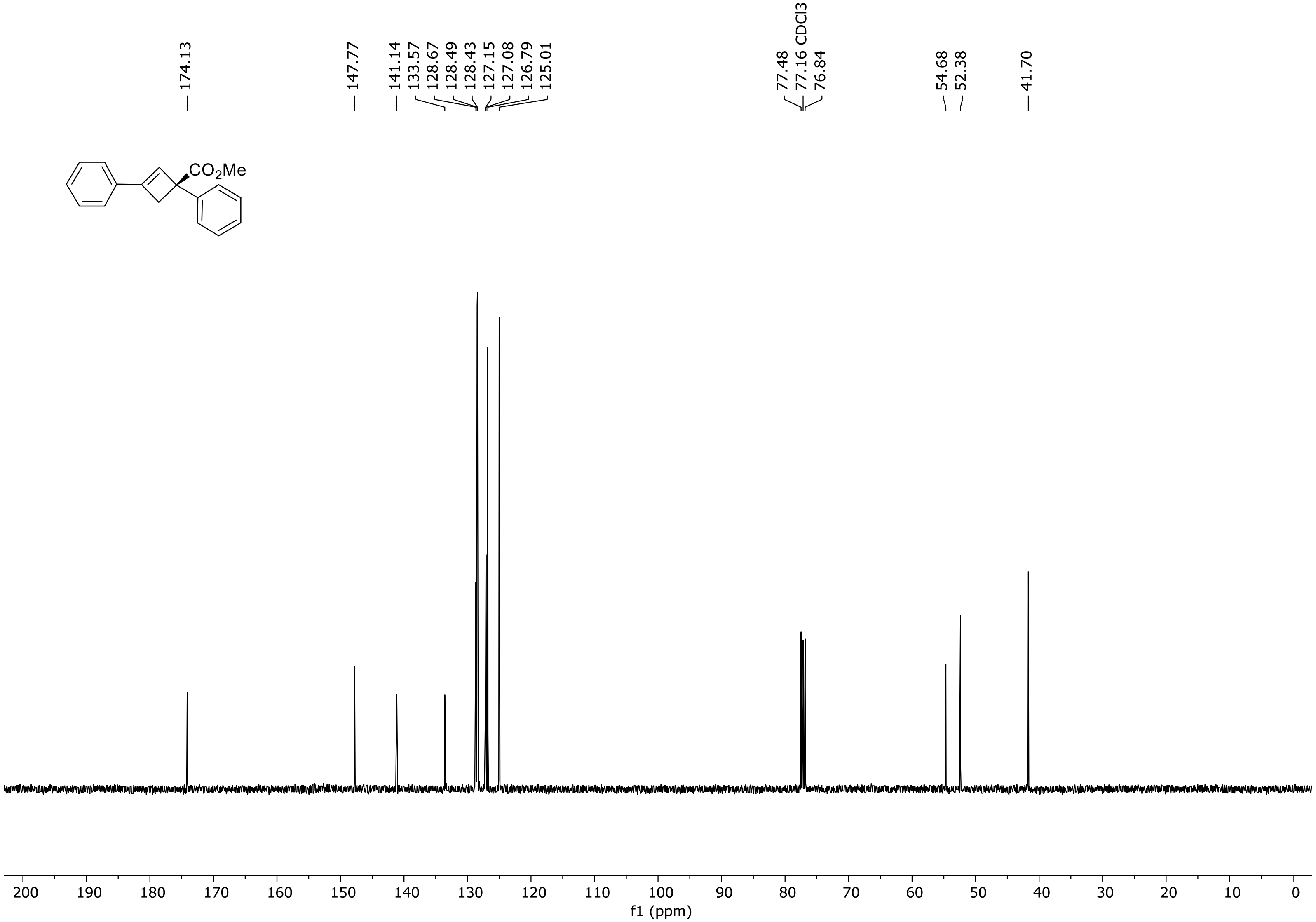

COSY (^1H–^1H) NMR (400 MHz, $CDCl_3$, 298 K) spectrum of **5a.**

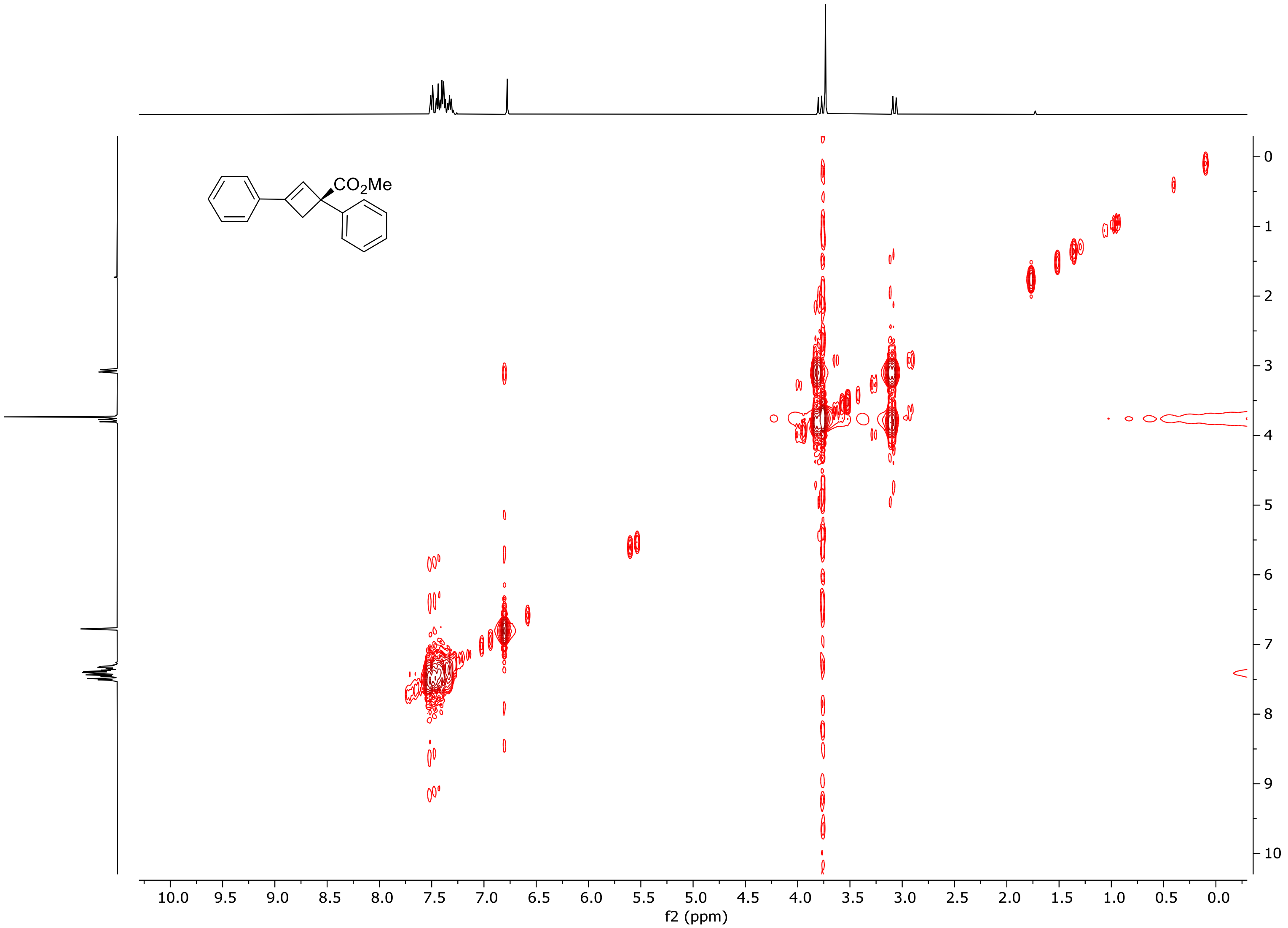

HSQC (^1H–^{13}C) NMR (400 MHz, $CDCl_3$, 298 K) spectrum of **5a**.

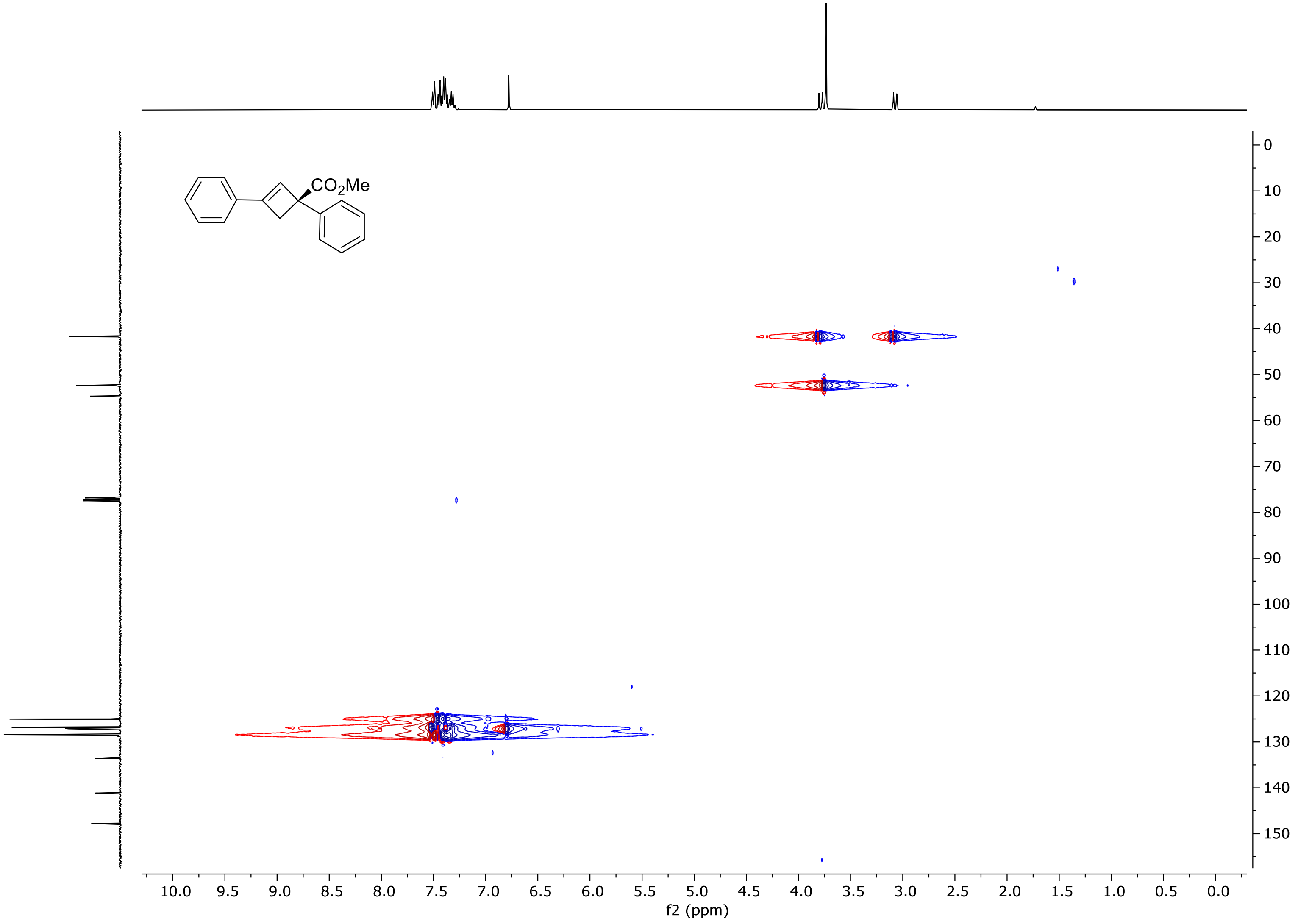

^{1}H NMR (400 MHz, $CDCl_3$, 298 K) spectrum of **5b**.

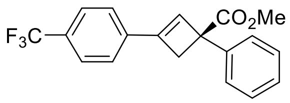

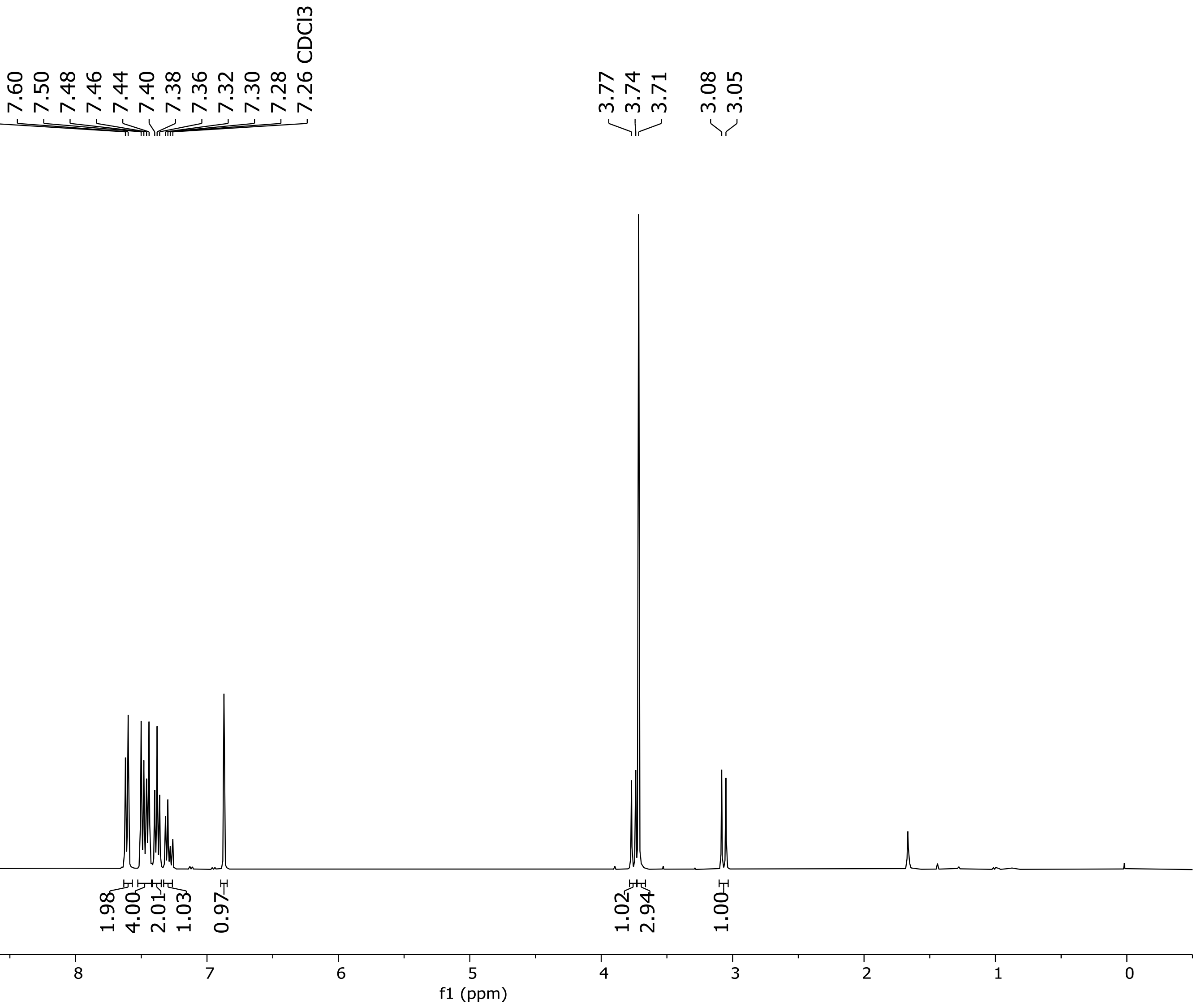

^{13}C NMR (100 MHz, $CDCl_3$, 298 K) spectrum of **5b**.

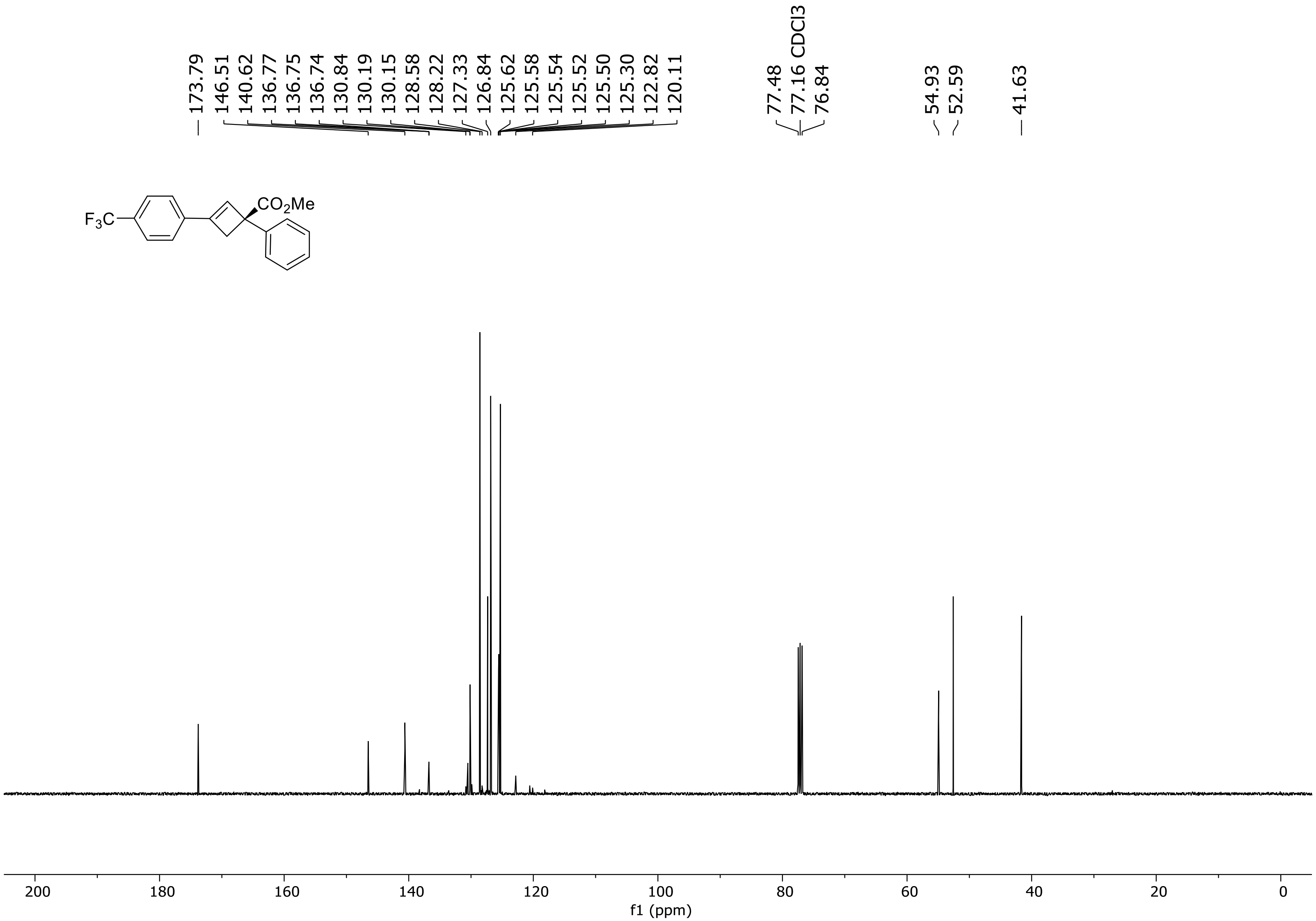

^{19}F NMR (377 MHz, $CDCl_3$, 298 K) spectrum of **5b**.

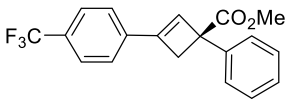

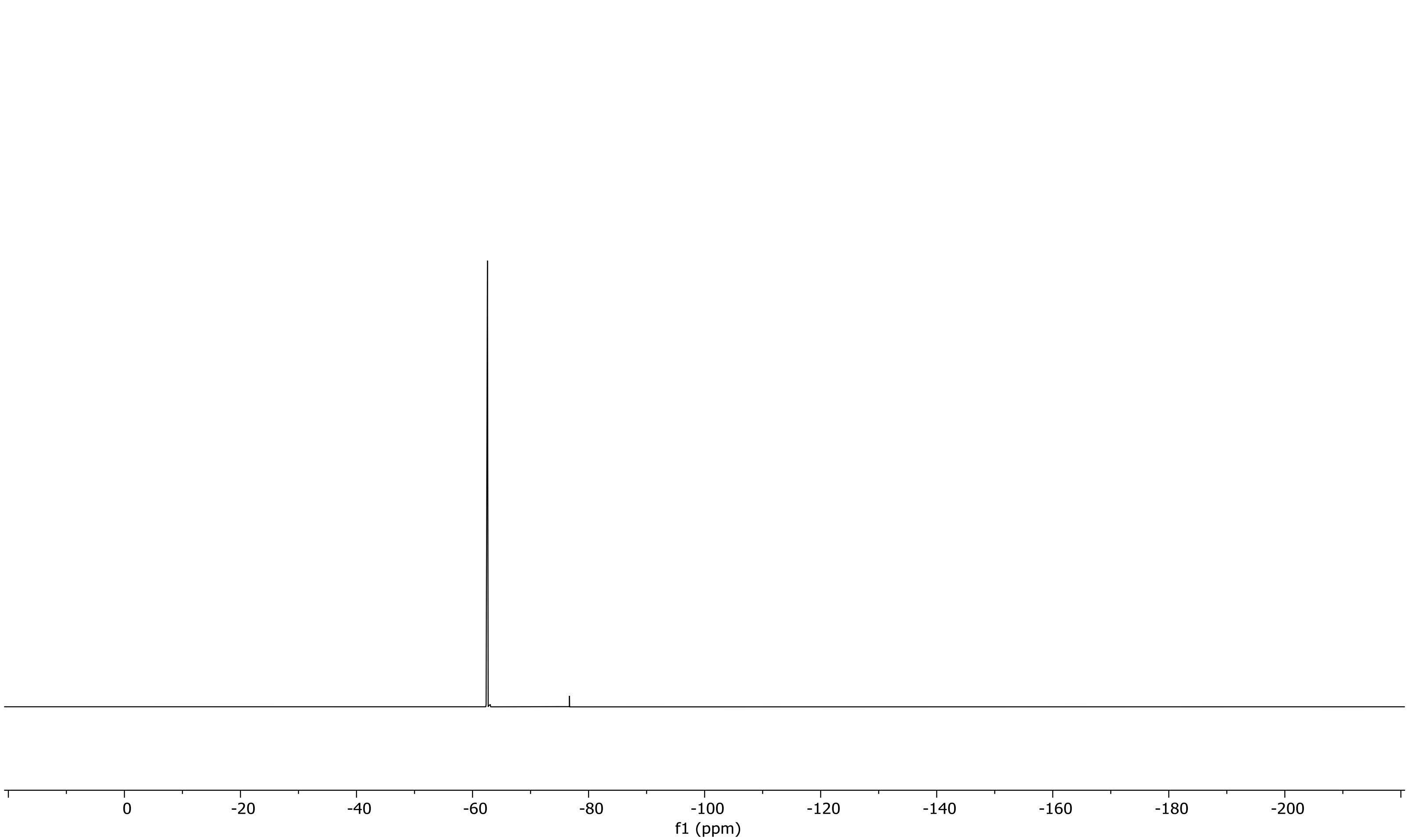

^{1}H NMR (400 MHz, $CDCl_3$, 298 K) spectrum of **5c**.

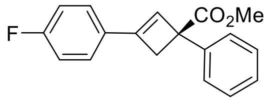

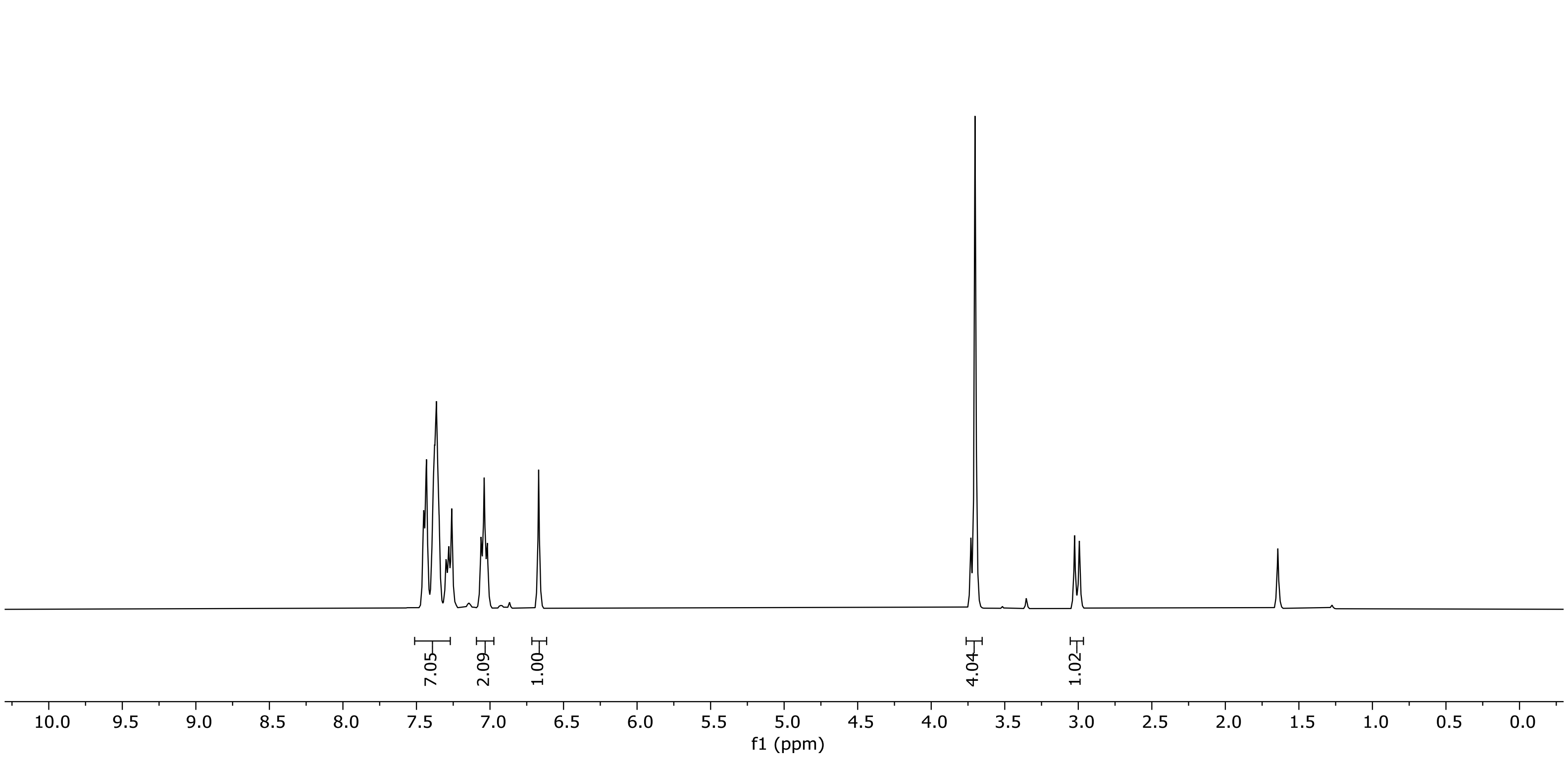

^{13}C NMR (100 MHz, $CDCl_3$, 298 K) spectrum of **5c**.

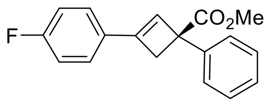

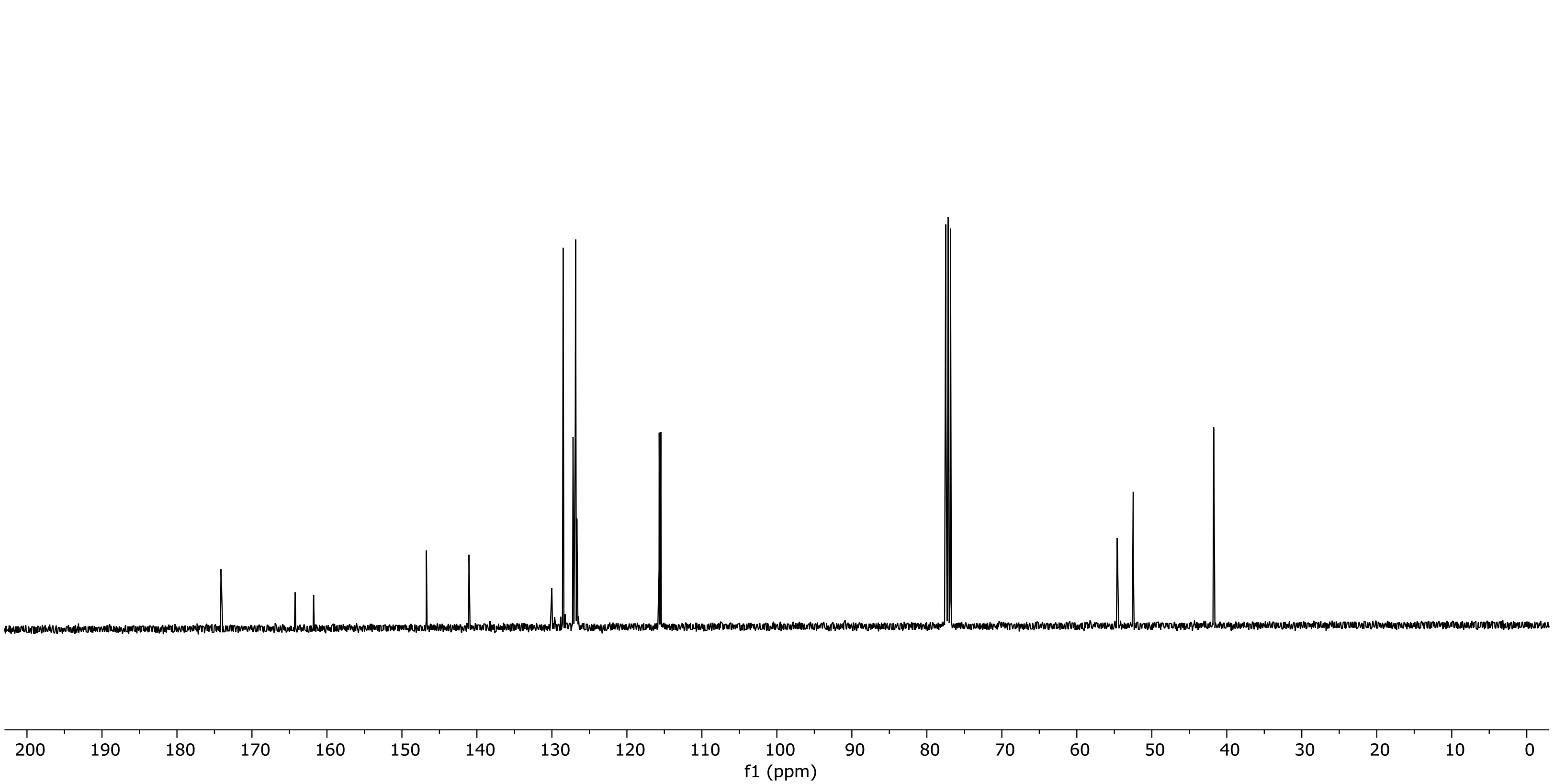

^{19}F NMR (377 MHz, $CDCl_3$, 298 K) spectrum of **5c.**

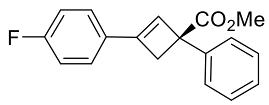

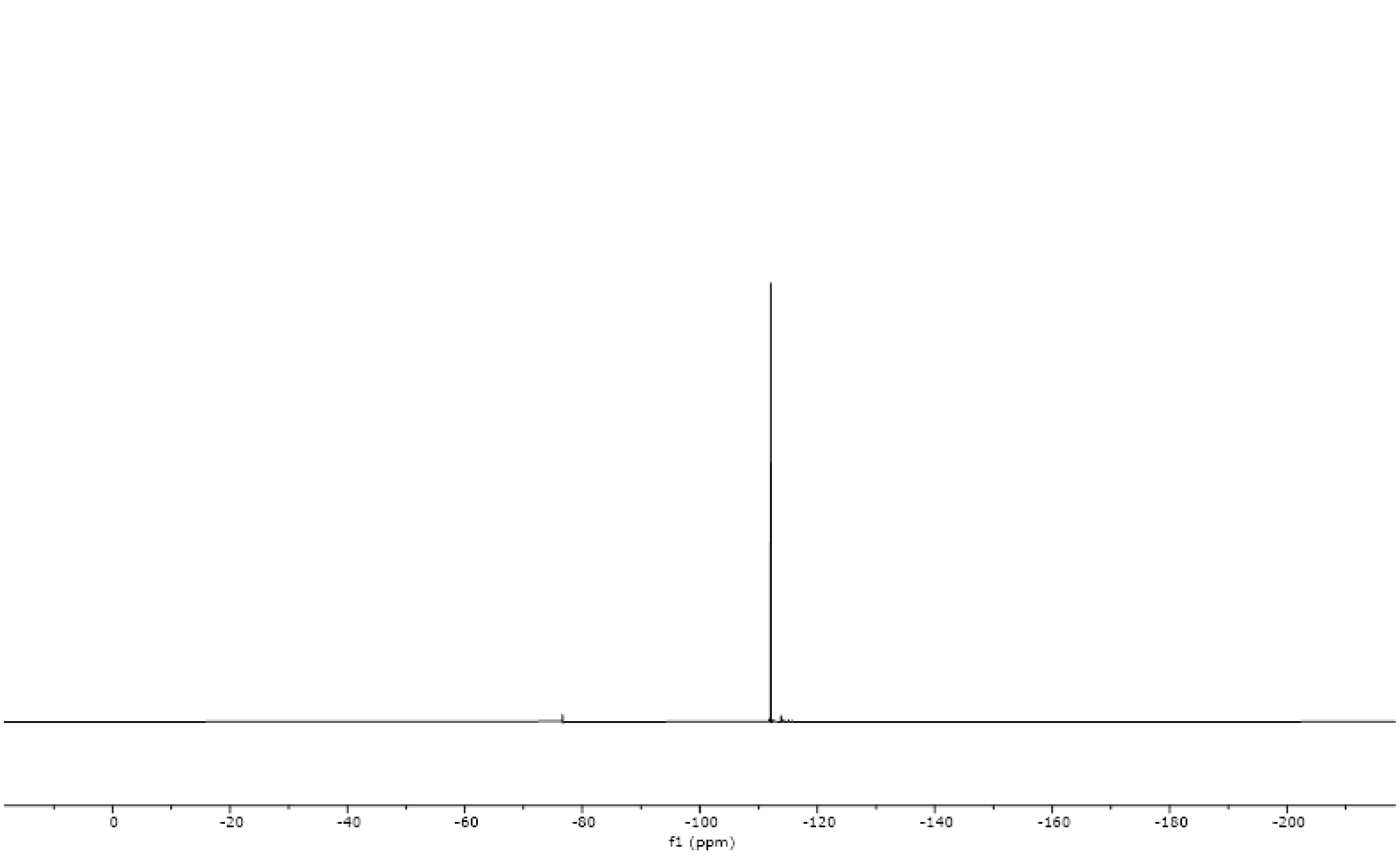

^{1}H NMR (400 MHz, $CDCl_3$, 298 K) spectrum of **5d**.

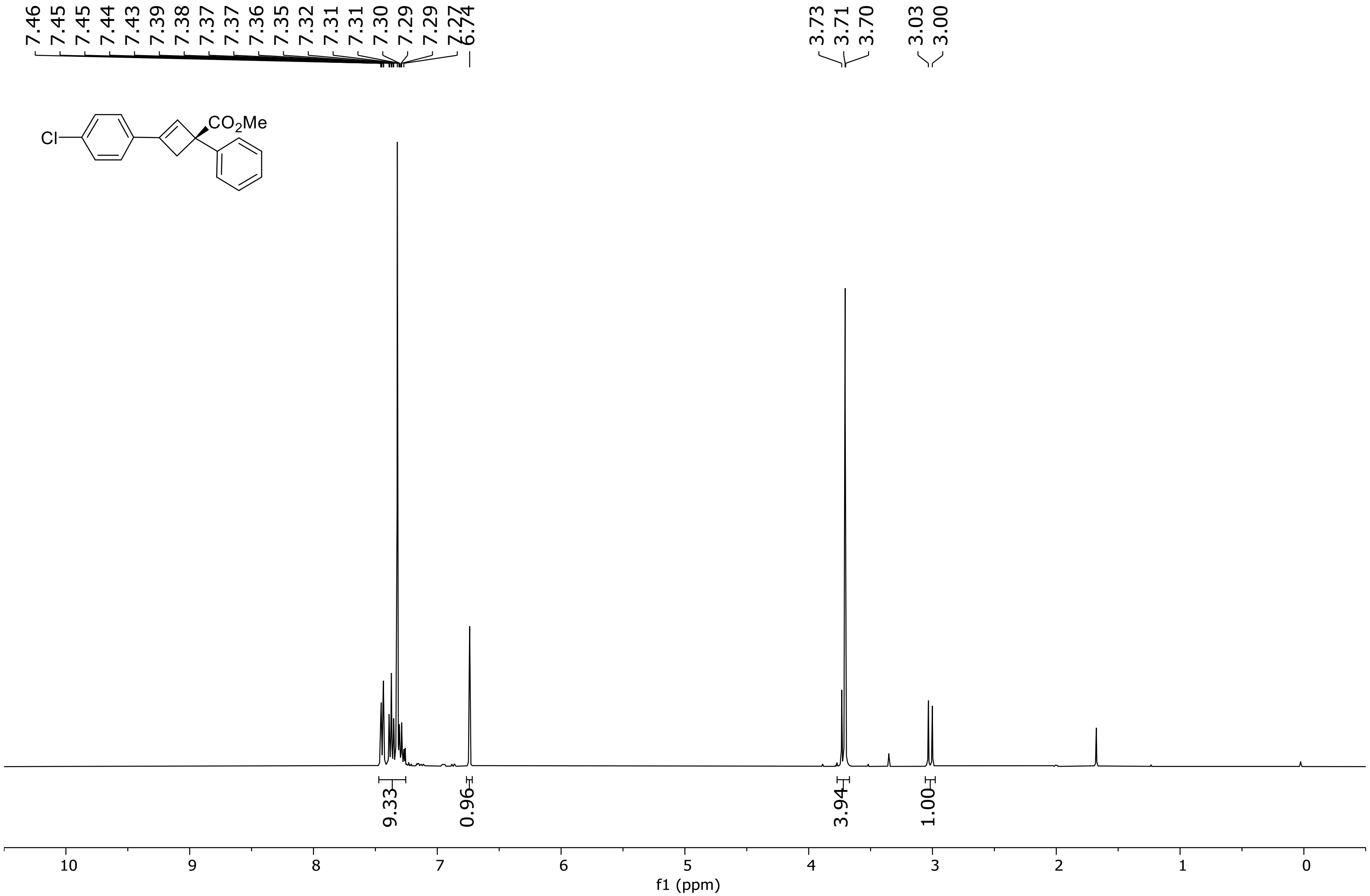

^{13}C NMR (100 MHz, $CDCl_3$, 298 K) spectrum of **5d**.

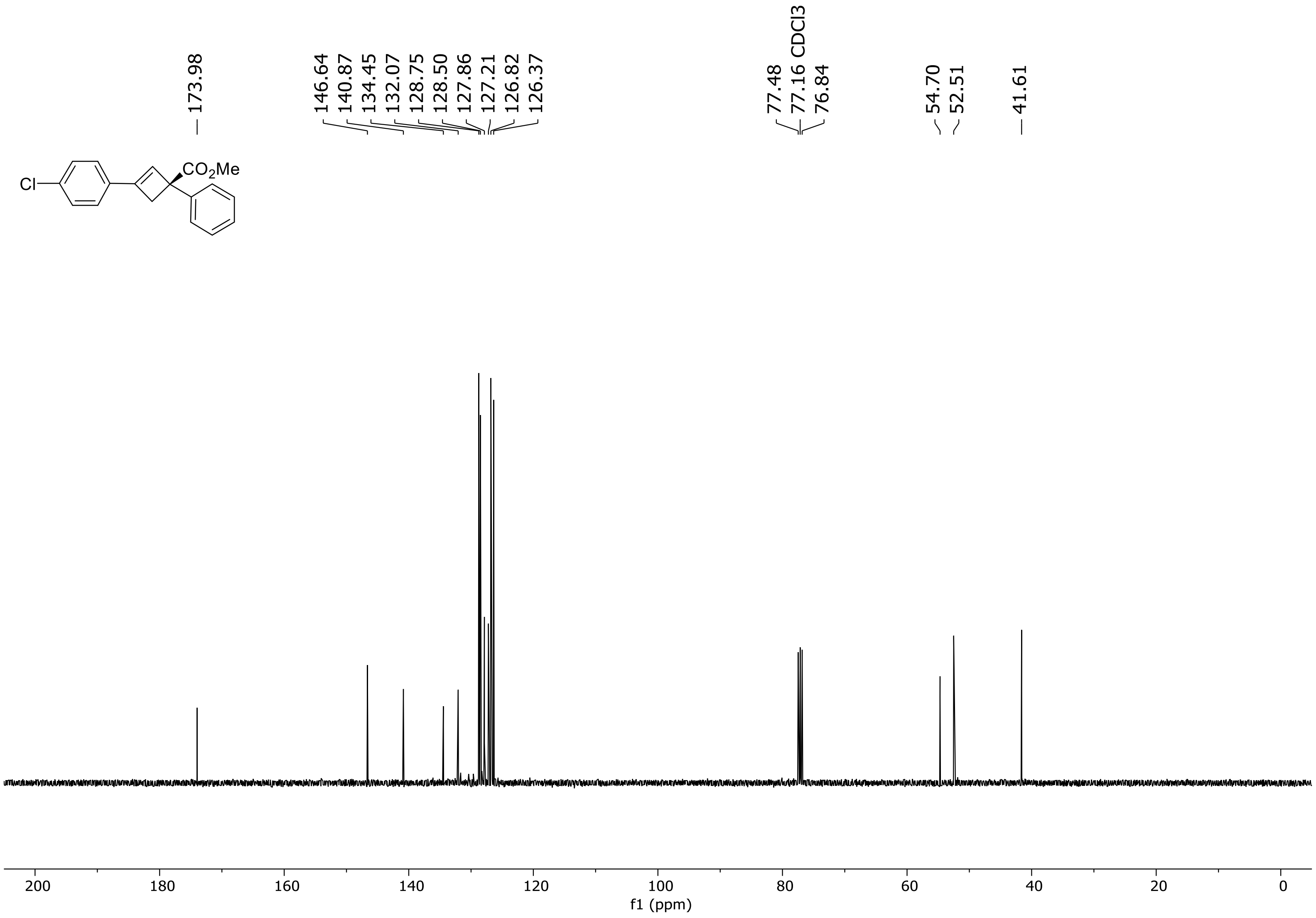

^{1}H NMR (400 MHz, $CDCl_3$, 298 K) spectrum of **5e**.

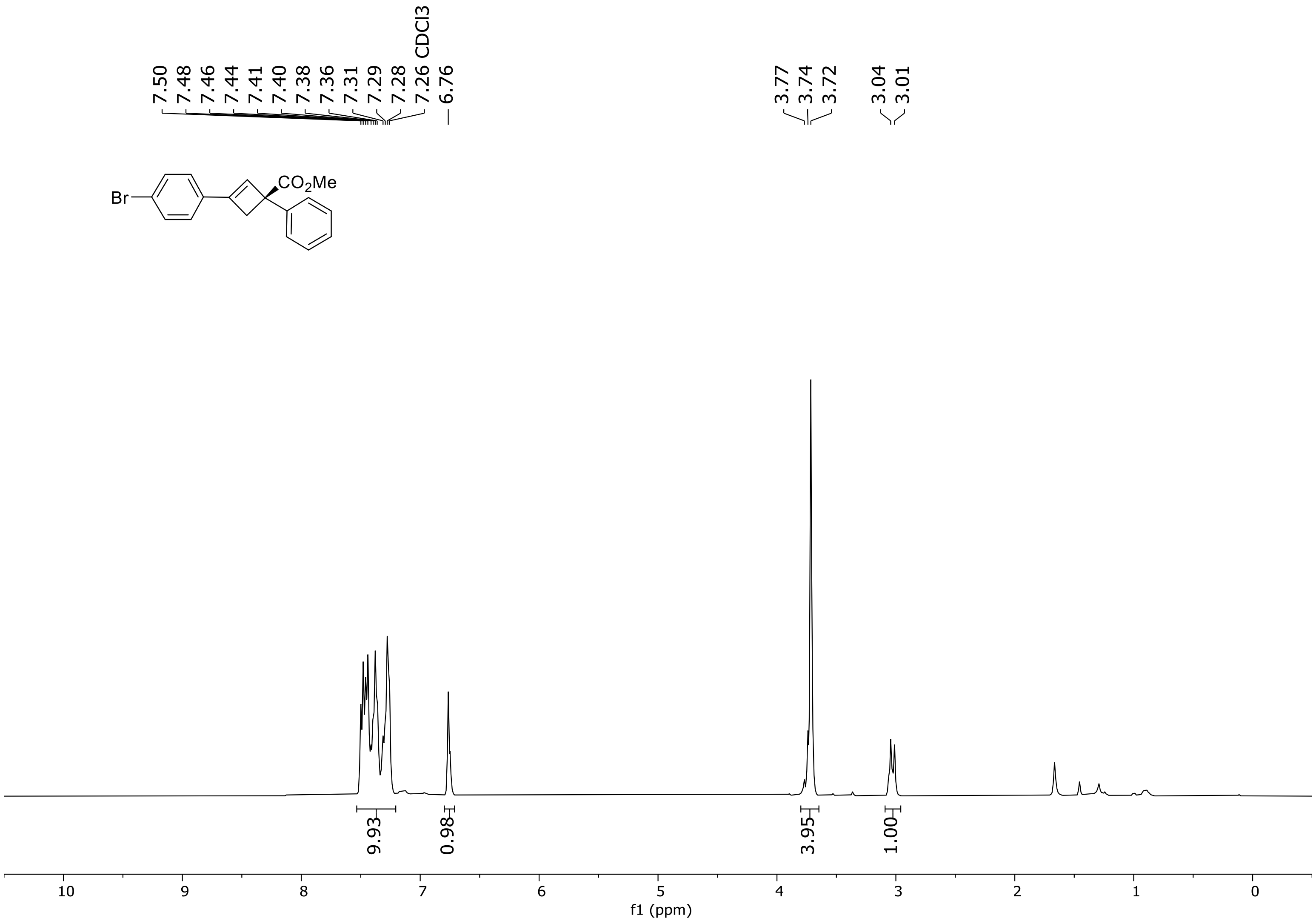

^{13}C NMR (100 MHz, $CDCl_3$, 298 K) spectrum of **5e**.

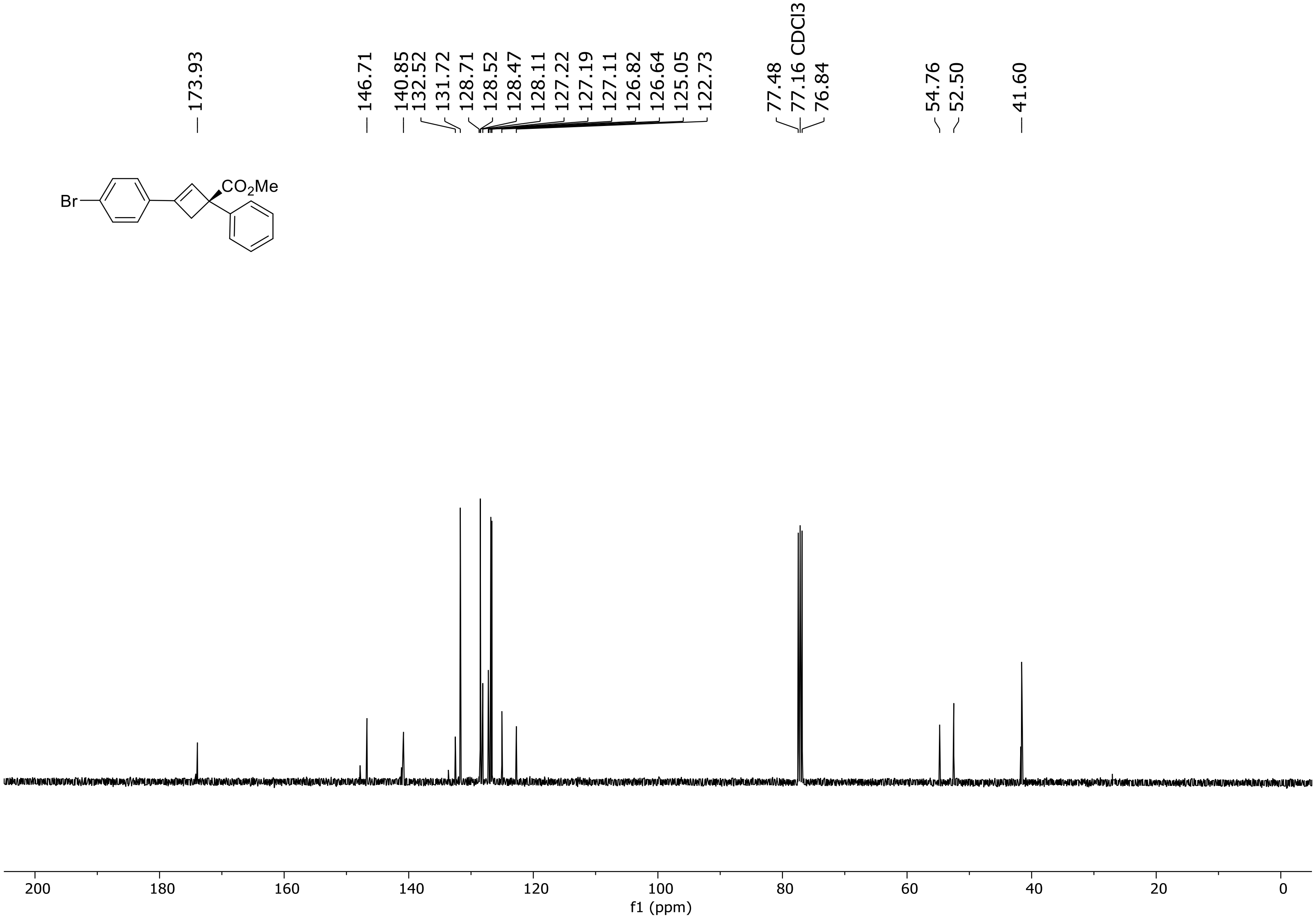

^{1}H NMR (400 MHz, $CDCl_3$, 298 K) spectrum of **5f**.

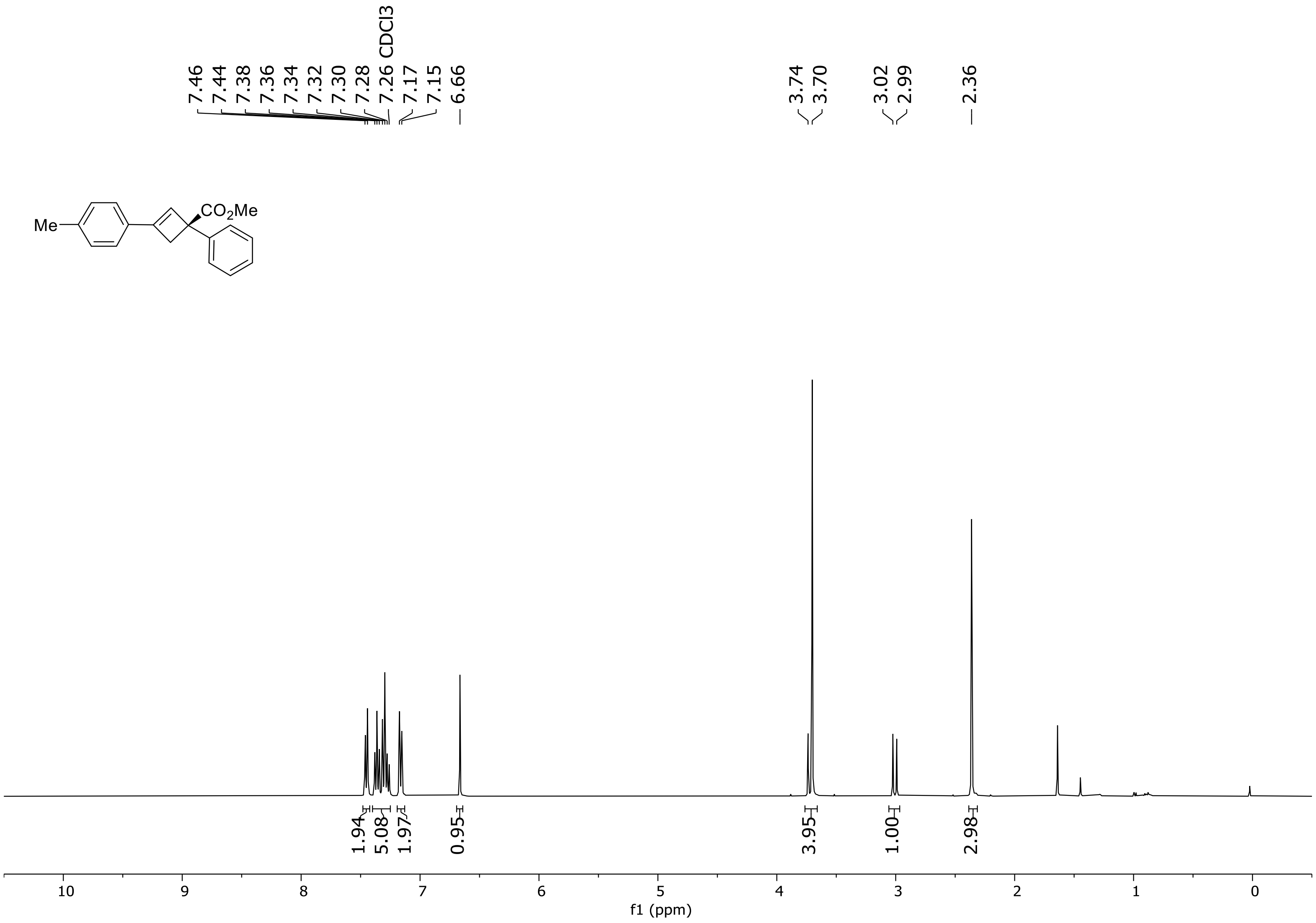

^{13}C NMR (100 MHz, $CDCl_3$, 298 K) spectrum of **5f**.

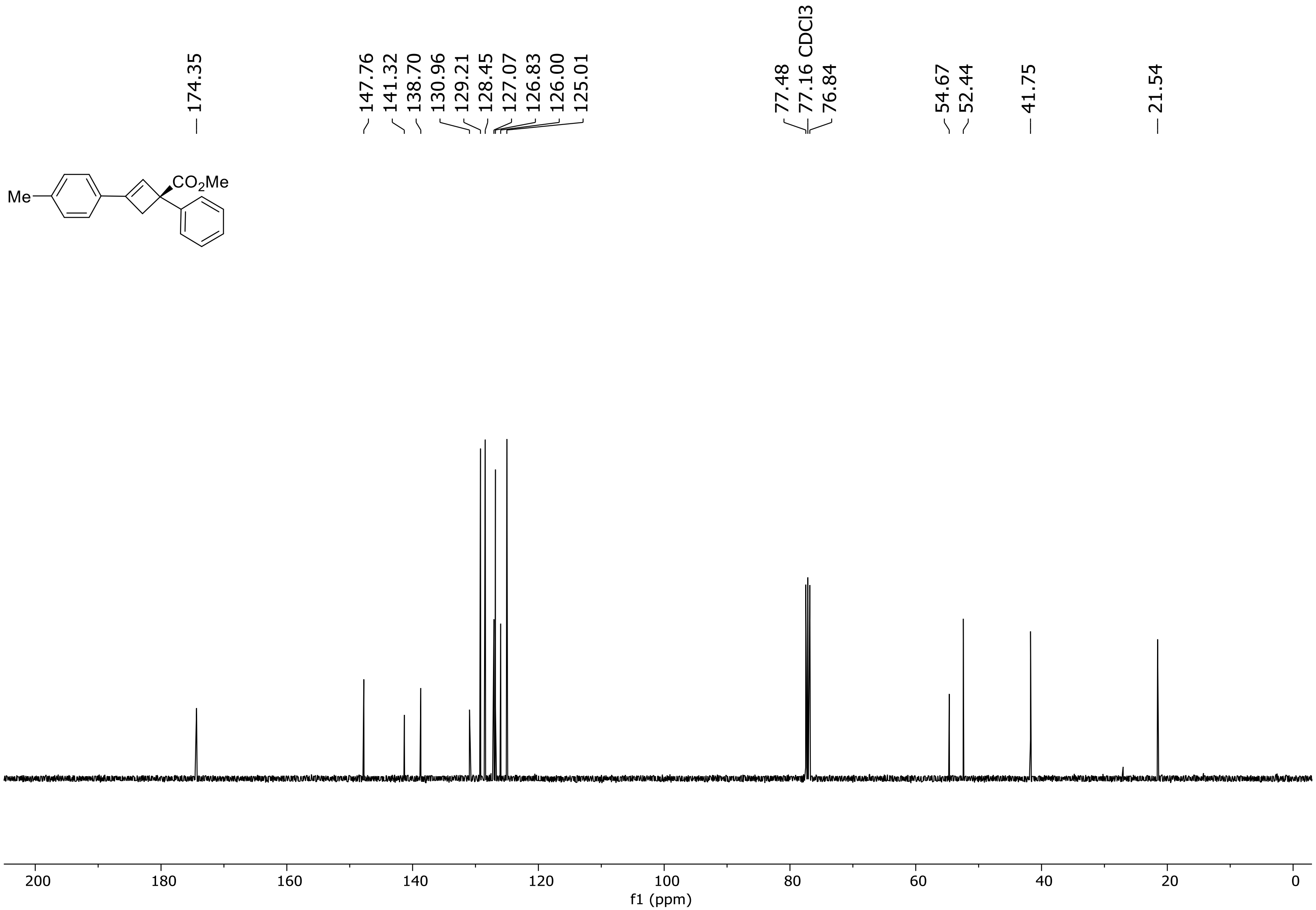

^{1}H NMR (400 MHz, $CDCl_3$, 298 K) spectrum of **5g**.

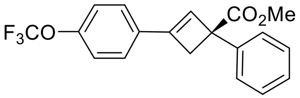

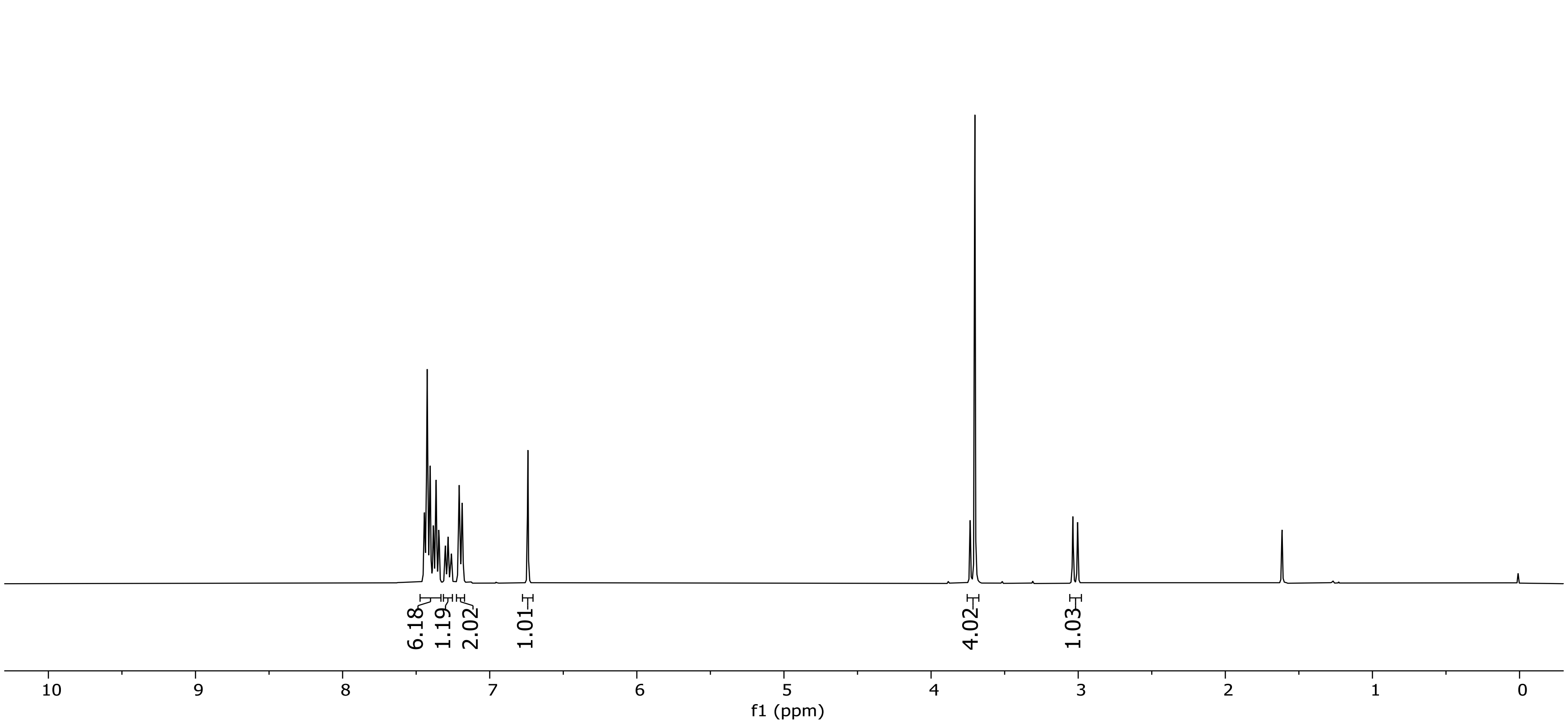

^{13}C NMR (100 MHz, $CDCl_3$, 298 K) spectrum of **5g**.

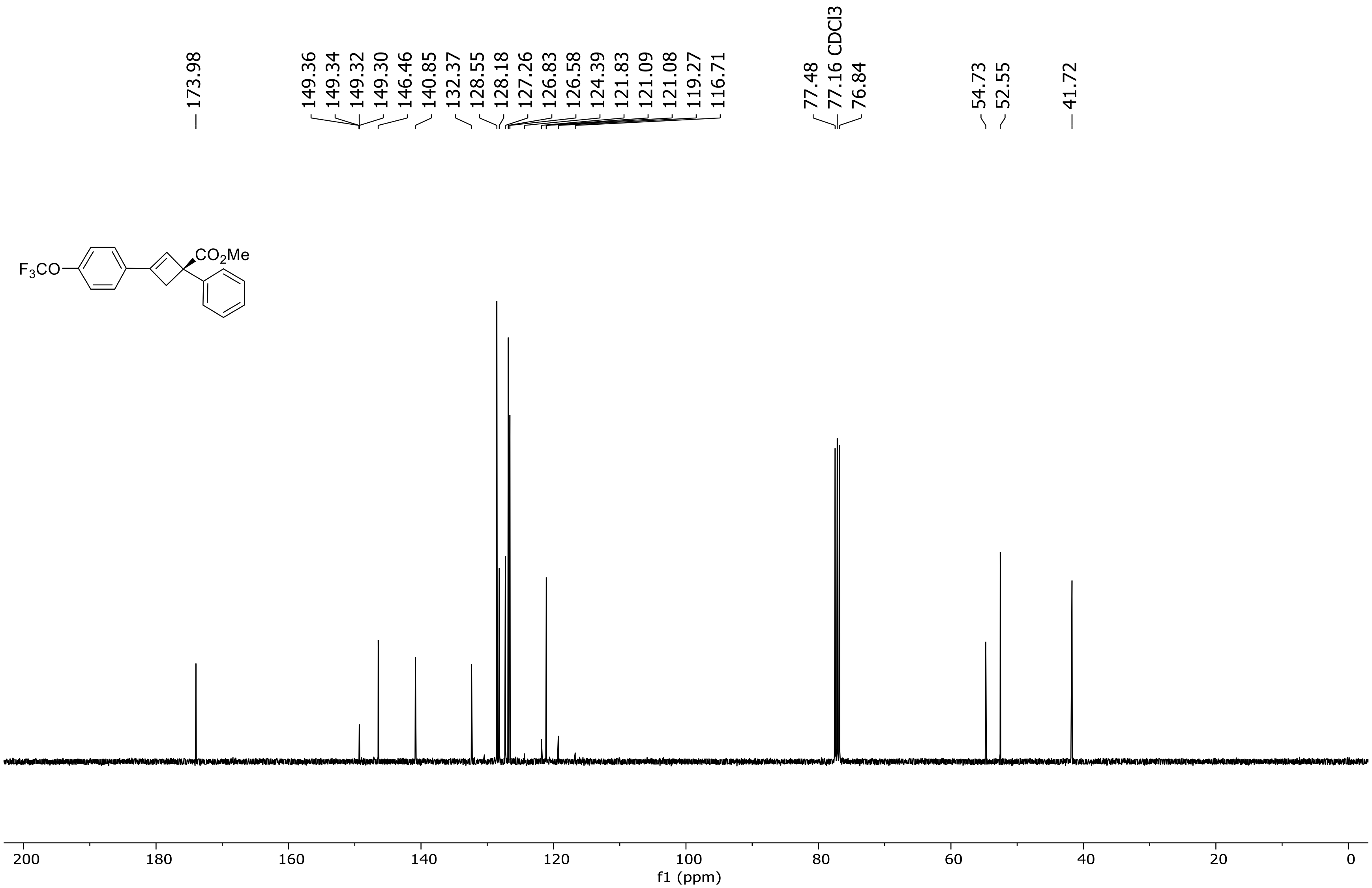

^{19}F NMR (377 MHz, $CDCl_3$, 298 K) spectrum of **5g**.

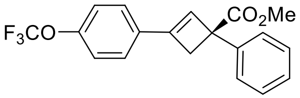

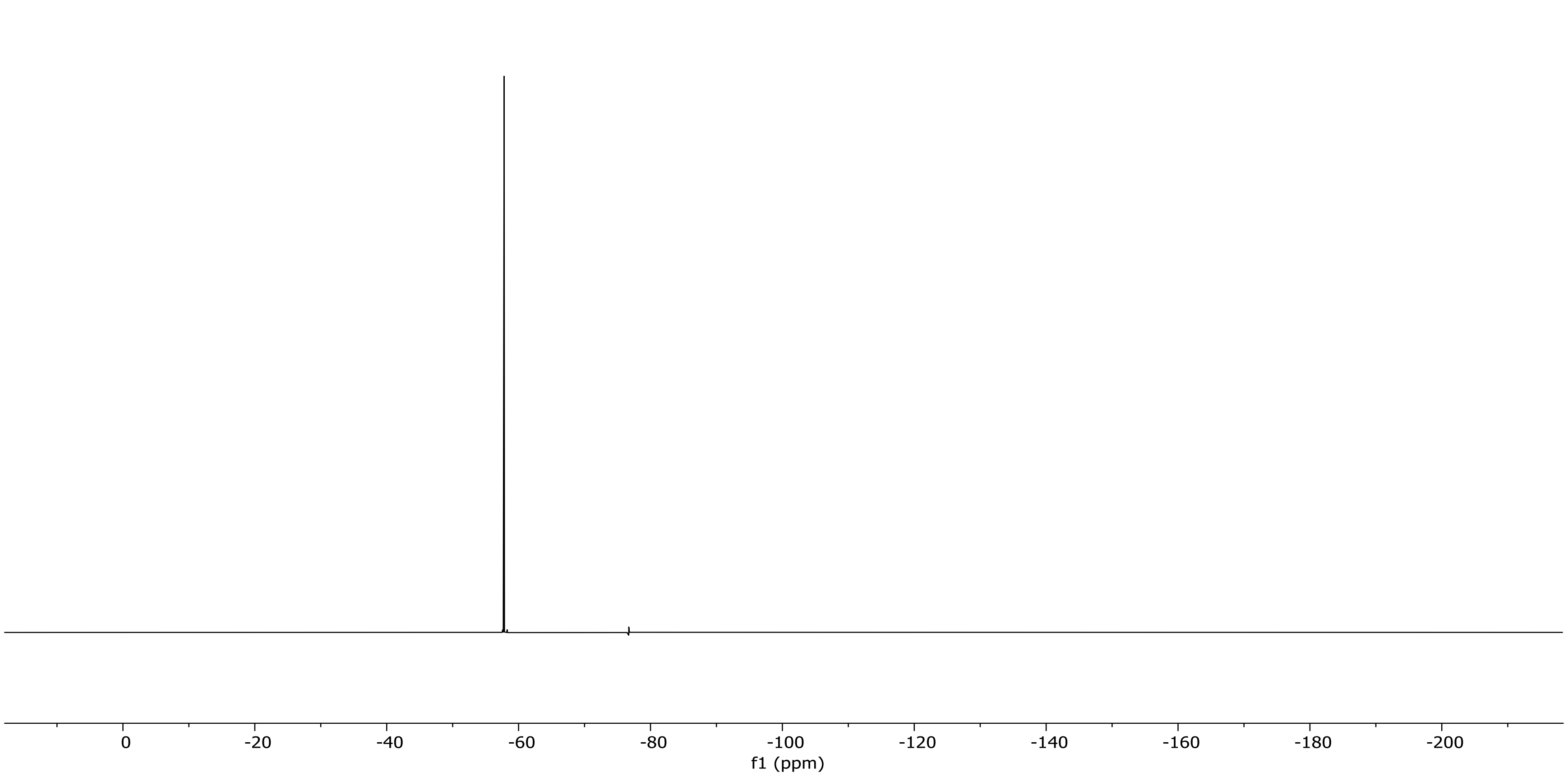

^{1}H NMR (400 MHz, $CDCl_3$, 298 K) spectrum of **5h**.

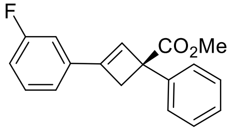

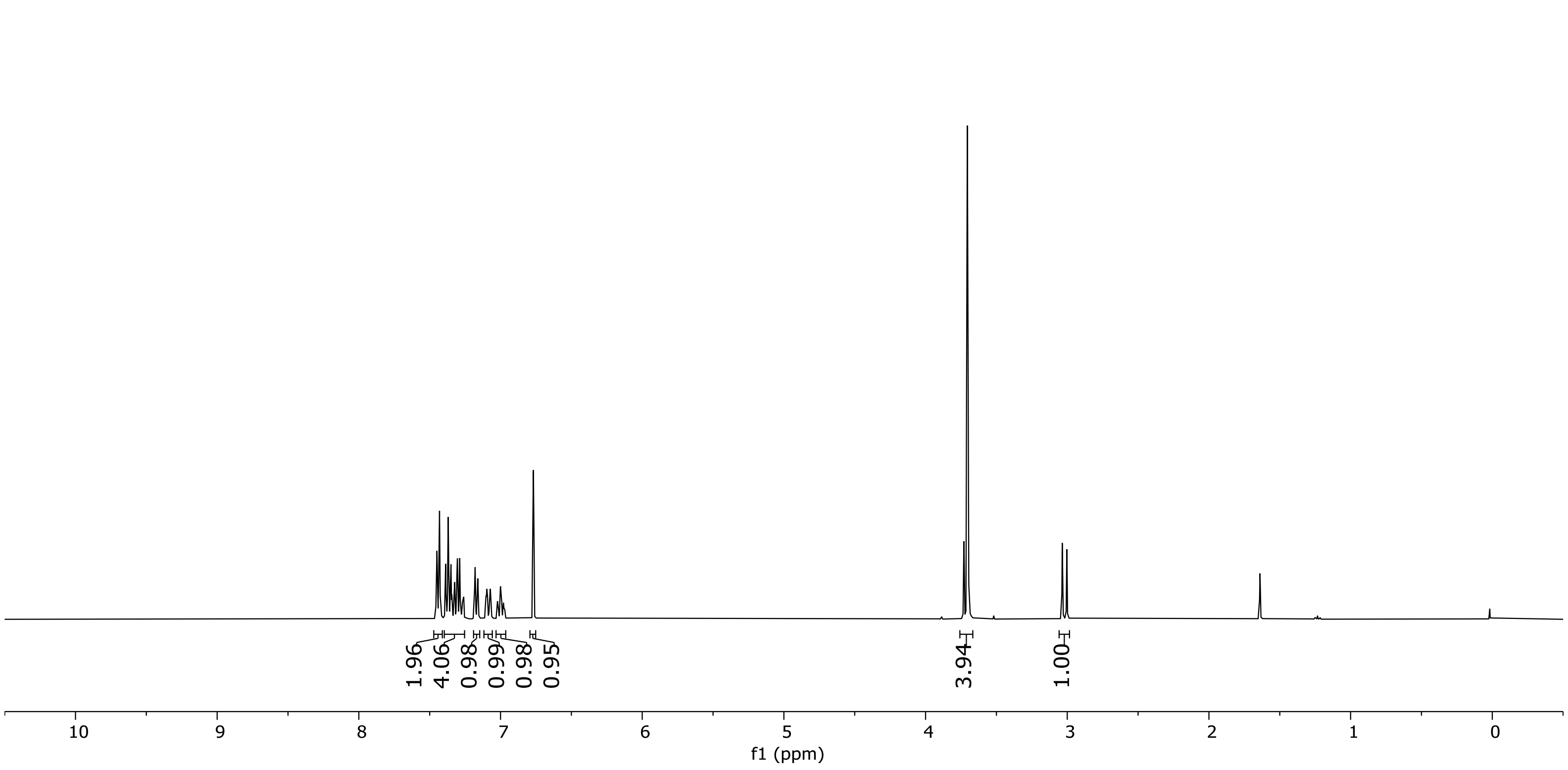

^{13}C NMR (100 MHz, $CDCl_3$, 298 K) spectrum of **5h**.

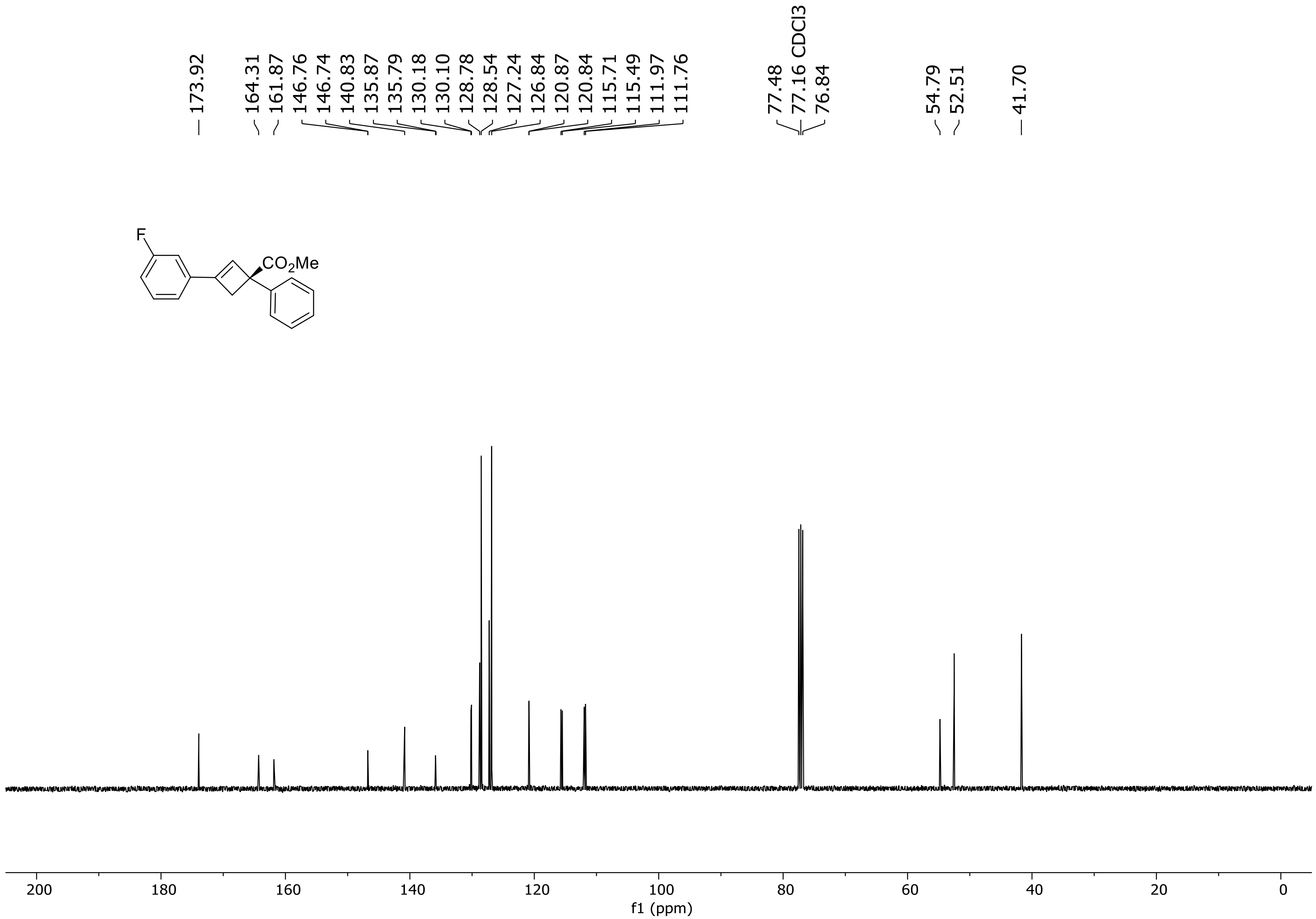

^{19}F NMR (377 MHz, $CDCl_3$, 298 K) spectrum of **5h**.

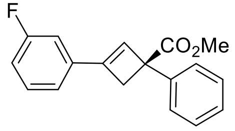

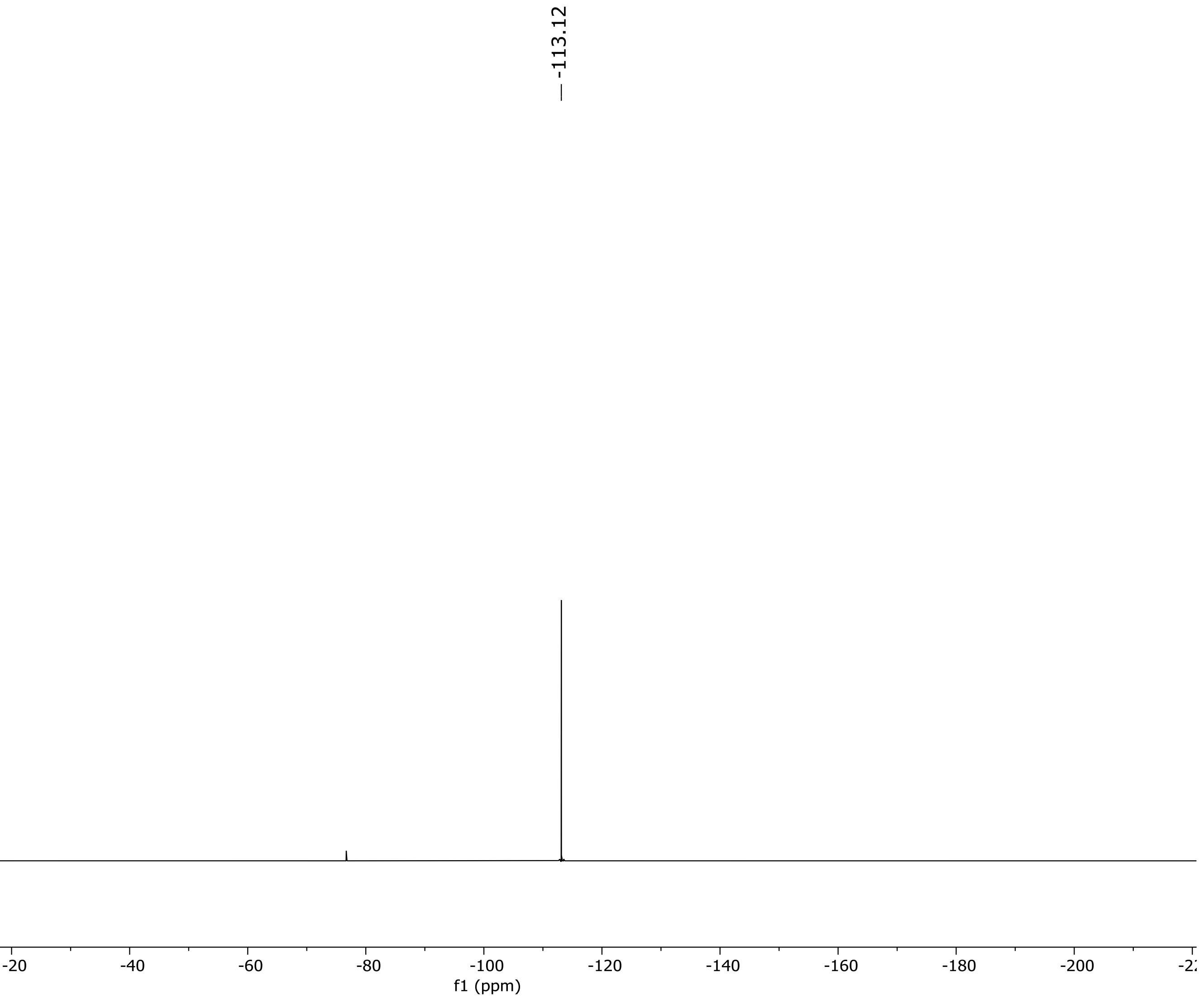

^{1}H NMR (400 MHz, $CDCl_3$, 298 K) spectrum of **5i**.

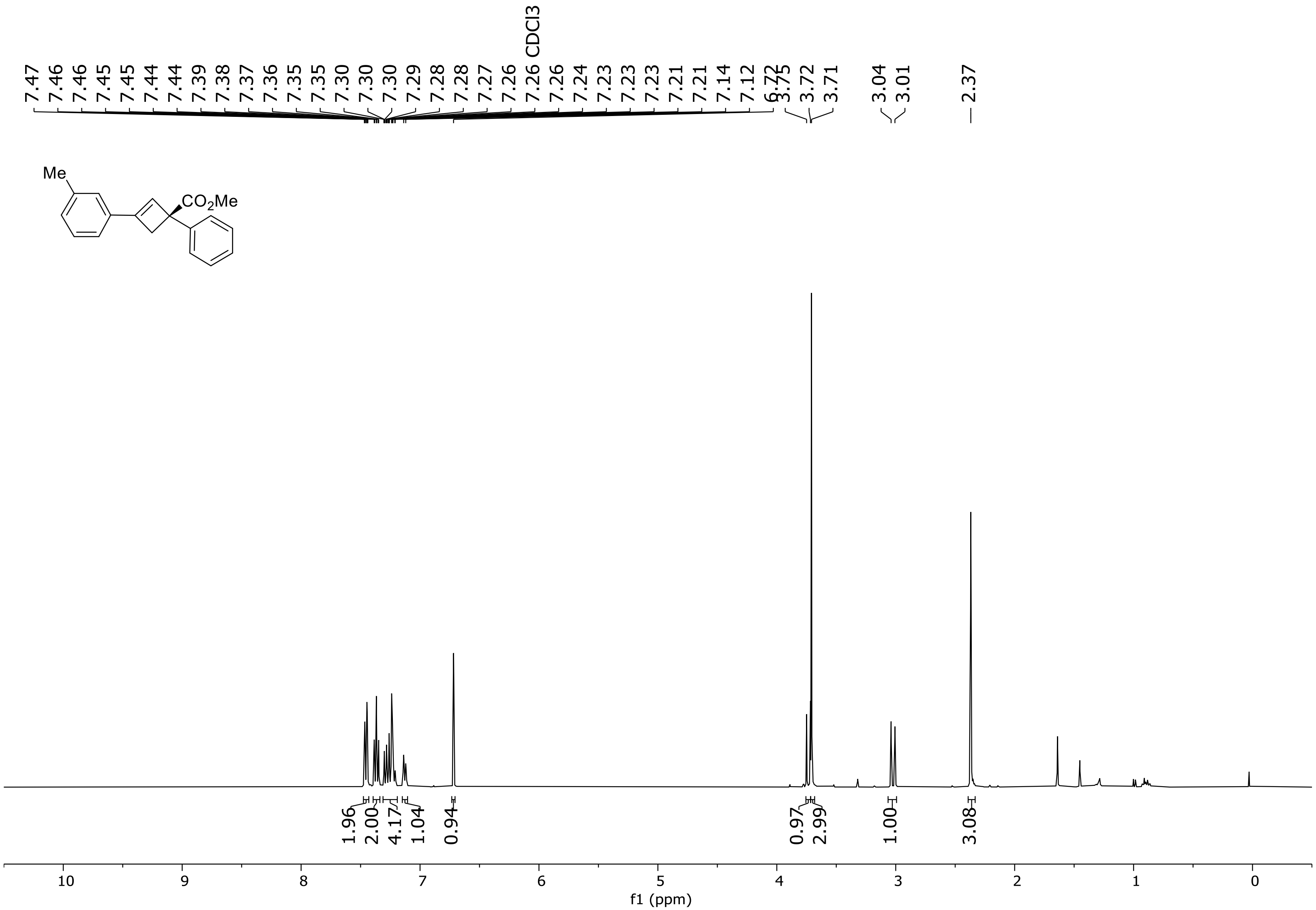

^{13}C NMR (100 MHz, $CDCl_3$, 298 K) spectrum of **5i**.

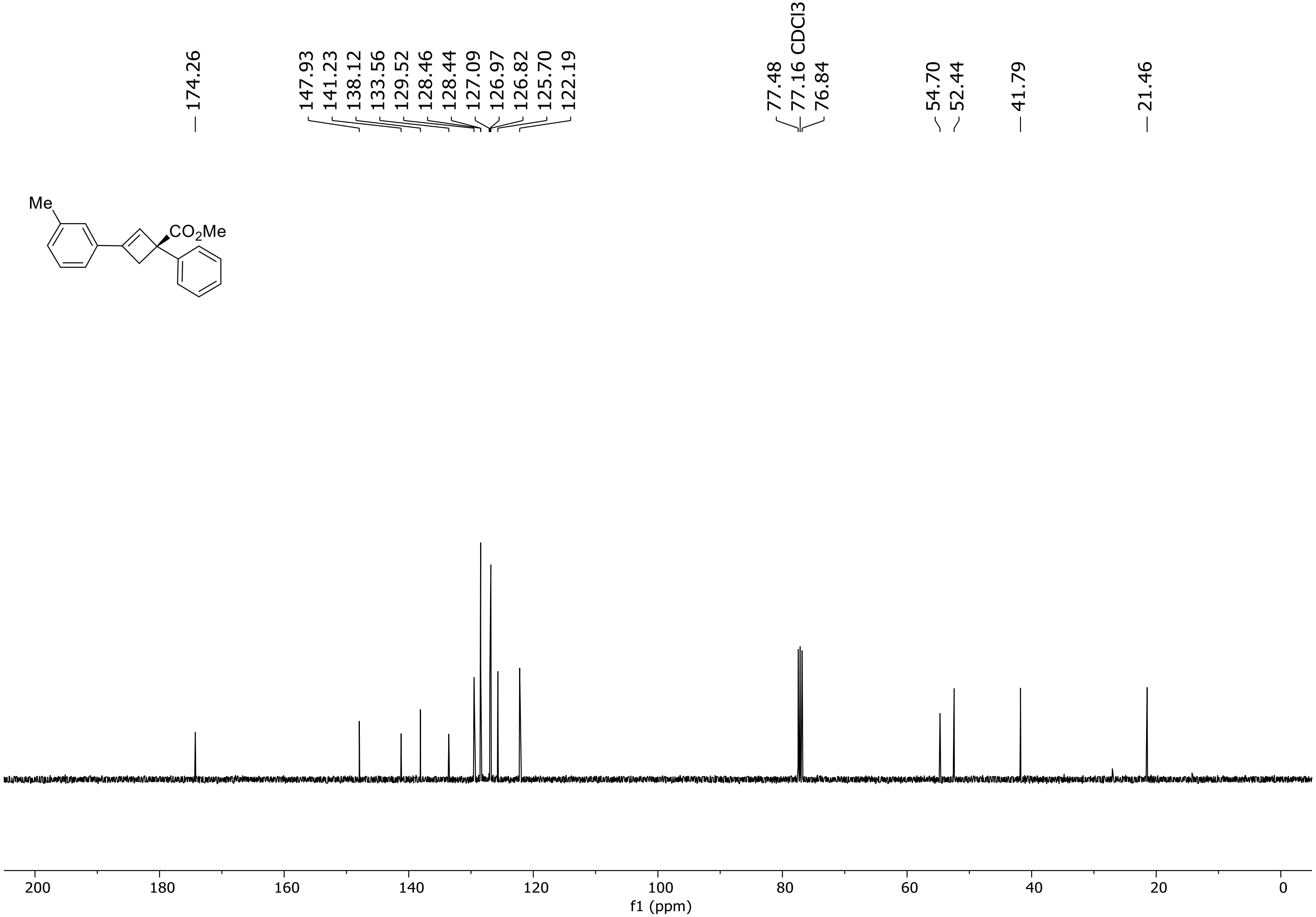

^{1}H NMR (400 MHz, $CDCl_3$, 298 K) spectrum of **5j**.

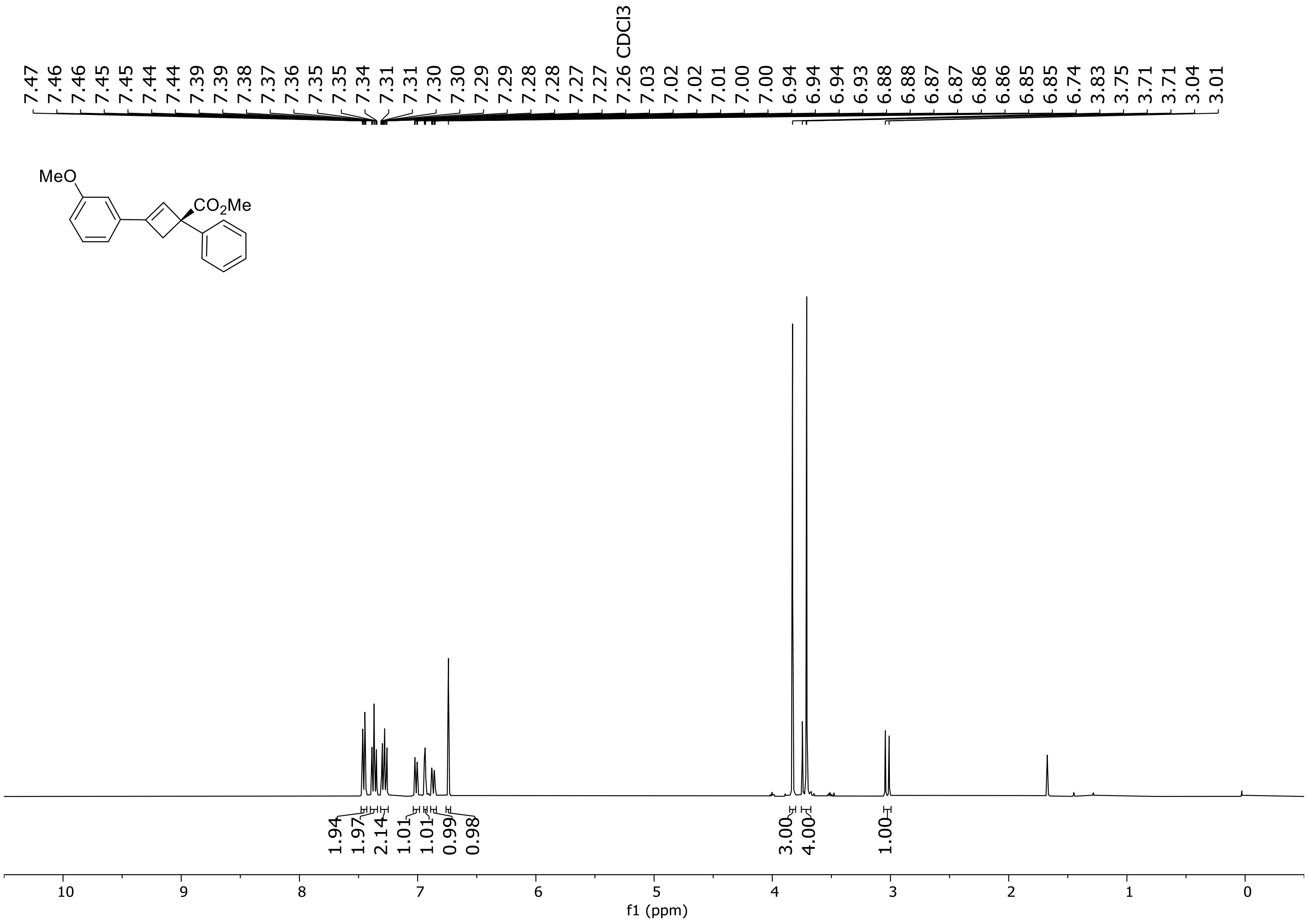

^{13}C NMR (100 MHz, $CDCl_3$, 298 K) spectrum of **5j**.

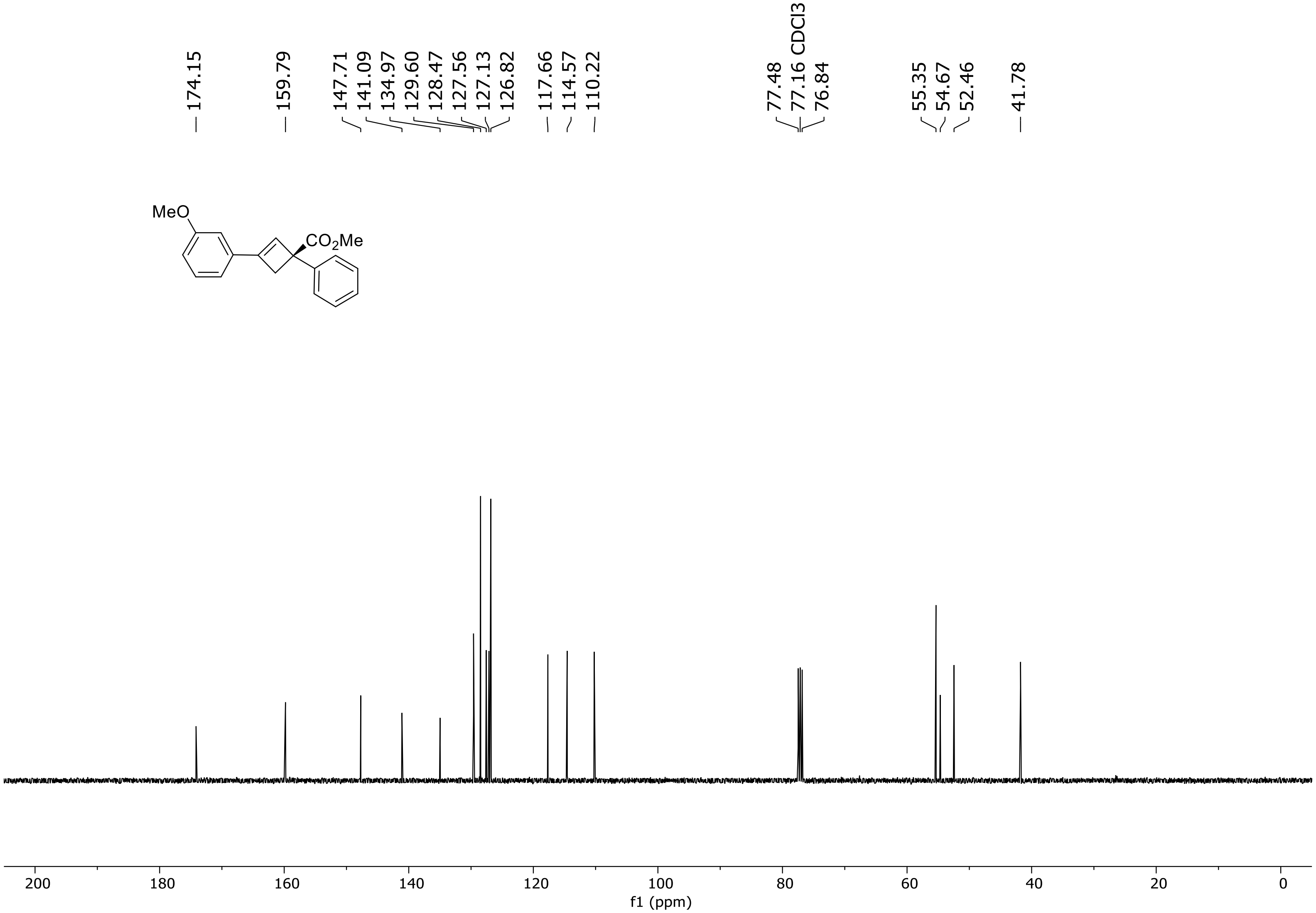

^{1}H NMR (400 MHz, $CDCl_3$, 298 K) spectrum of **5k**.

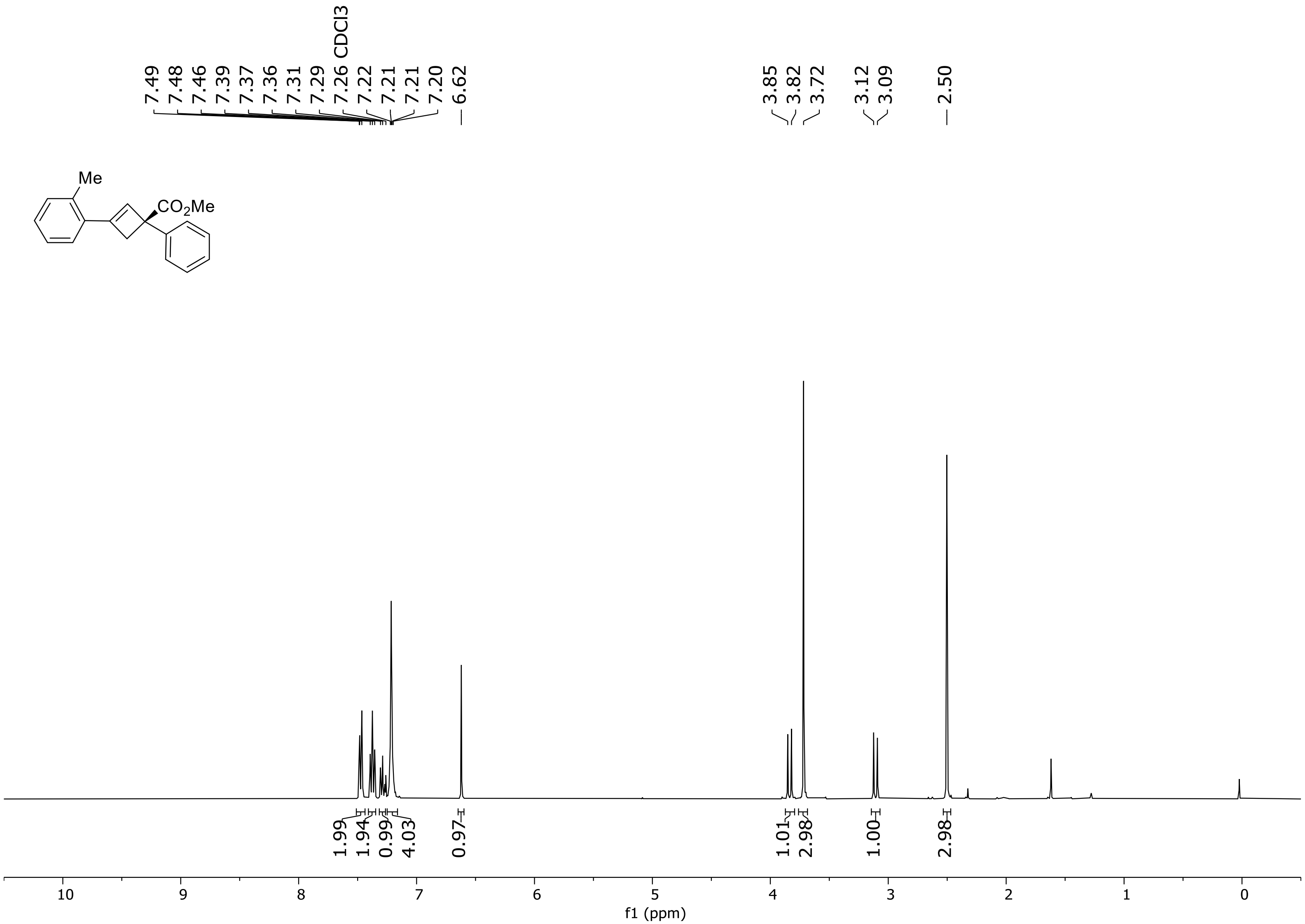

^{13}C NMR (100 MHz, $CDCl_3$, 298 K) spectrum of **5k**.

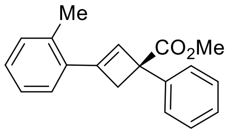

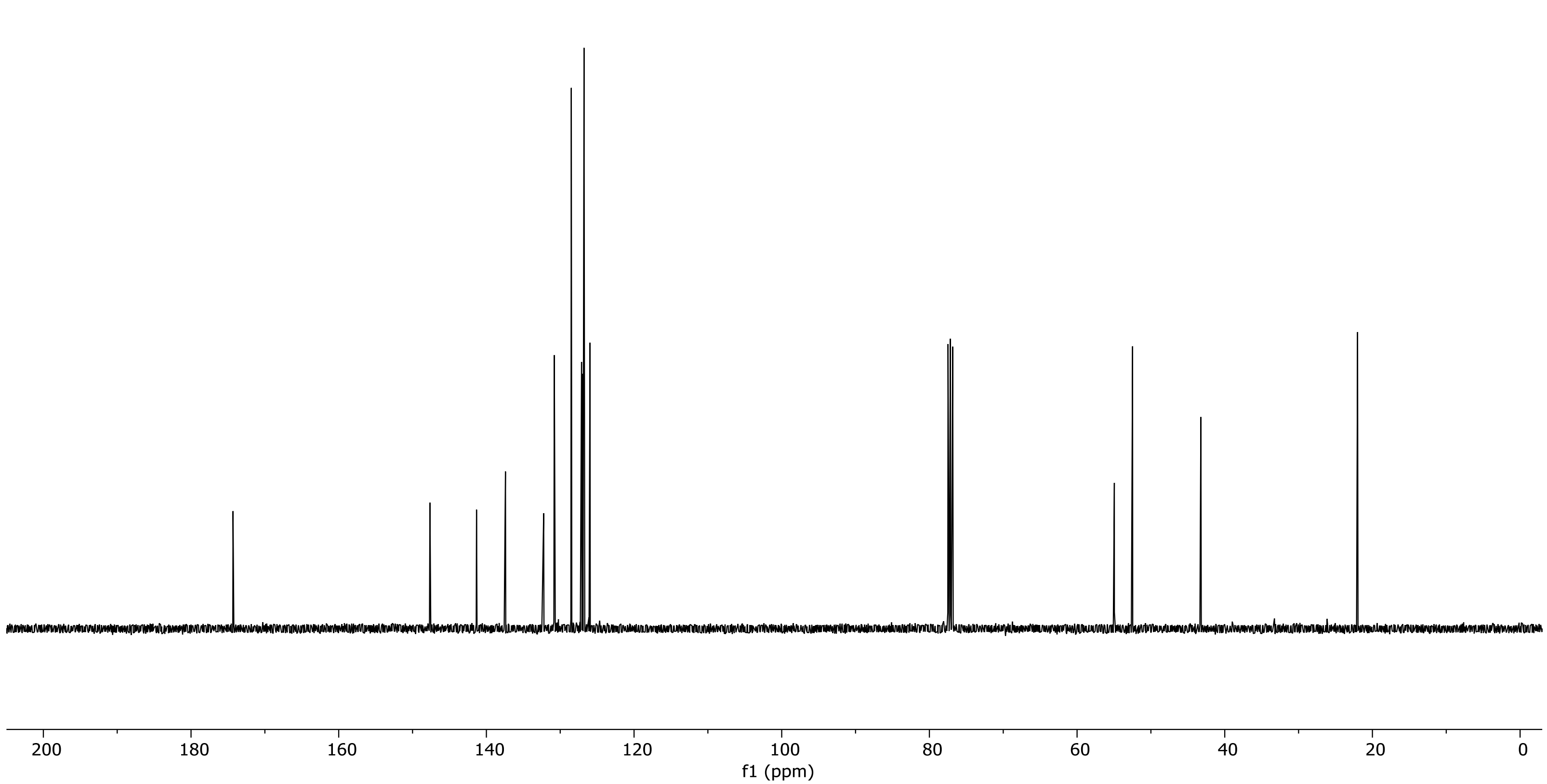

^1H NMR (400 MHz, $CDCl_3$, 298 K) spectrum of **5l**.

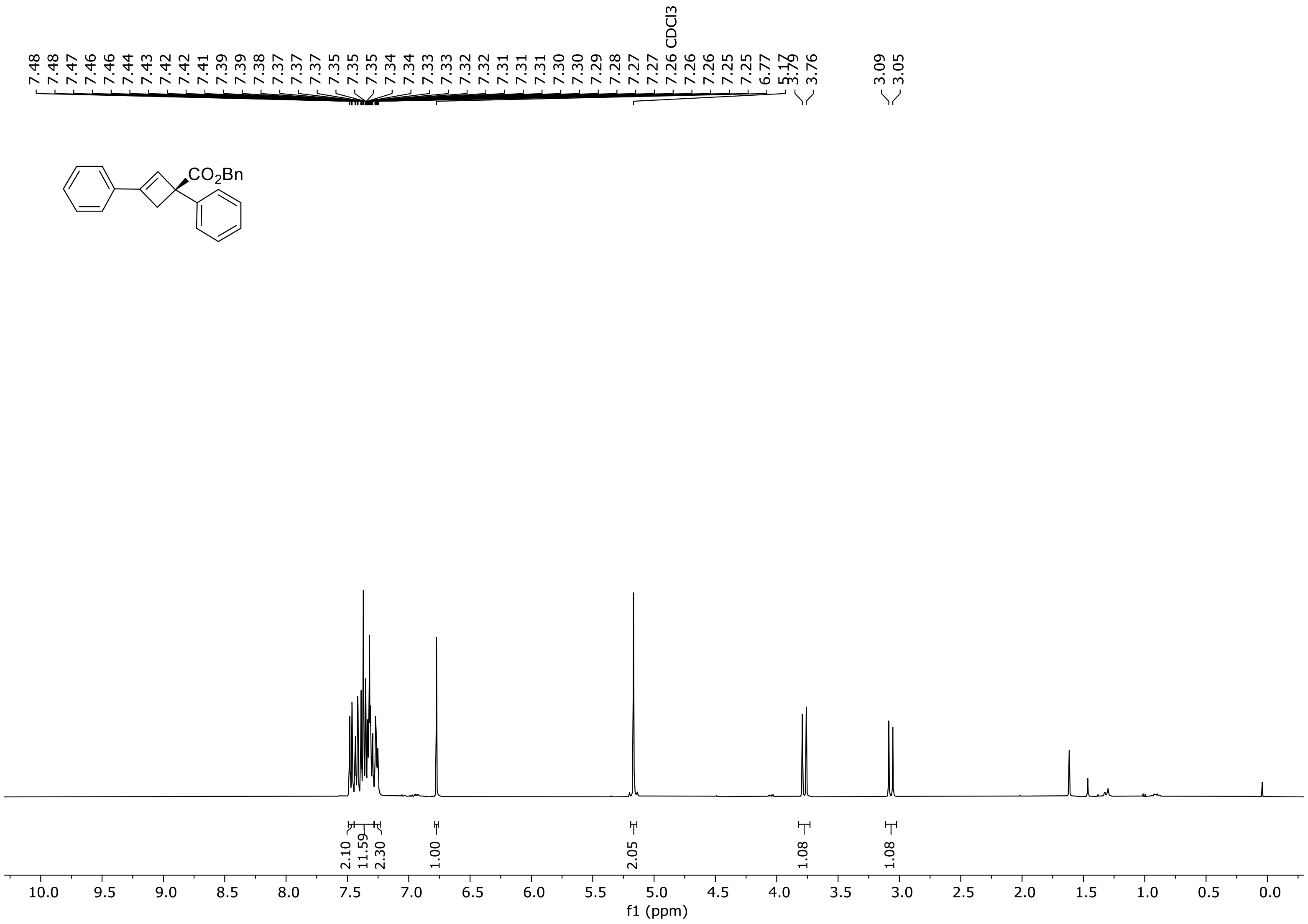

^{13}C NMR (100 MHz, $CDCl_3$, 298 K) spectrum of **5l**.

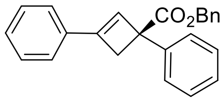

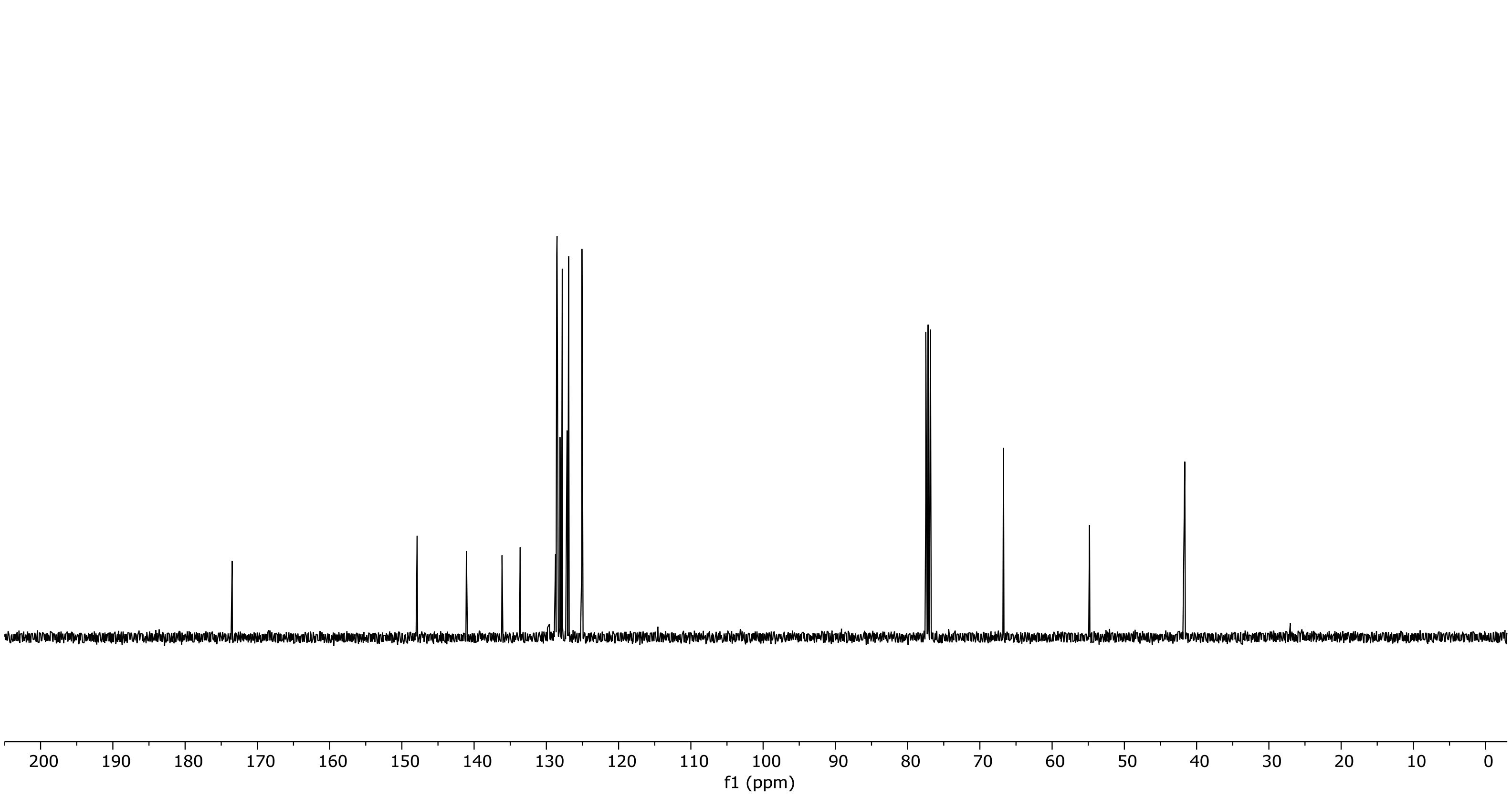

^{1}H NMR (400 MHz, $CDCl_3$, 298 K) spectrum of **5m**.

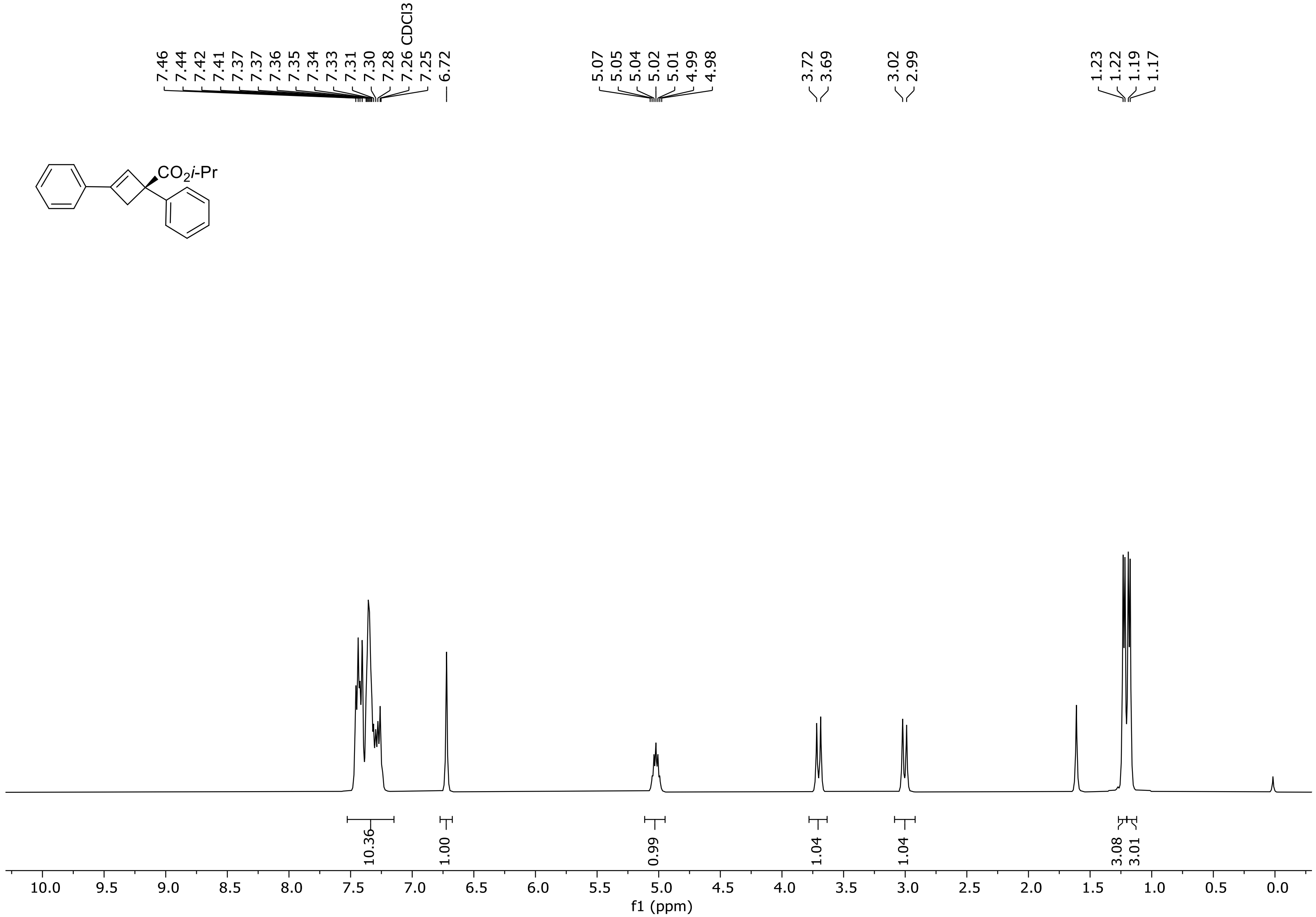

^{13}C NMR (100 MHz, $CDCl_3$, 298 K) spectrum of **5m**.

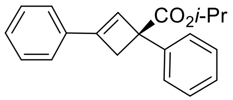

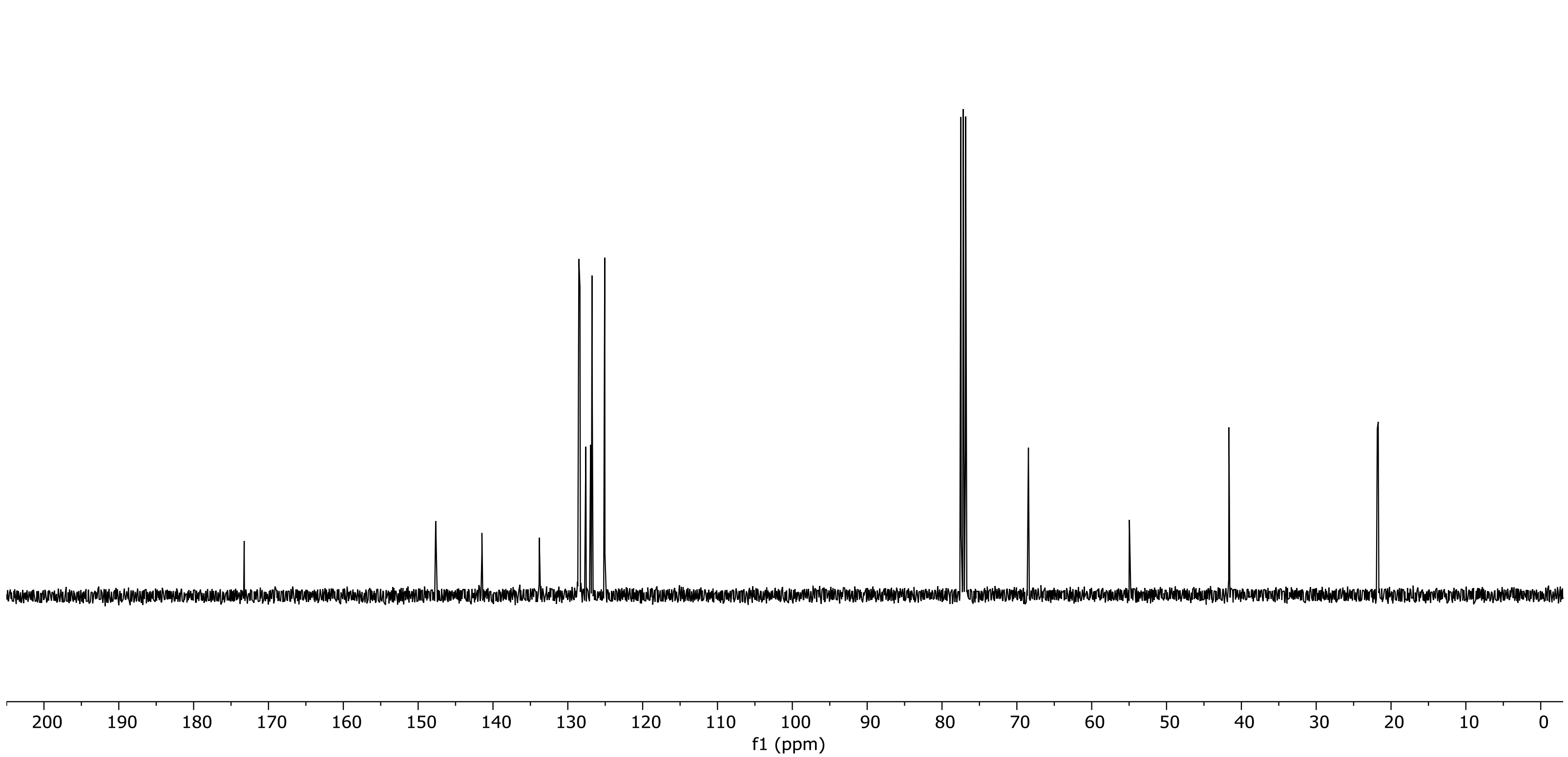

^{1}H NMR (400 MHz, $CDCl_3$, 298 K) spectrum of **5n**.

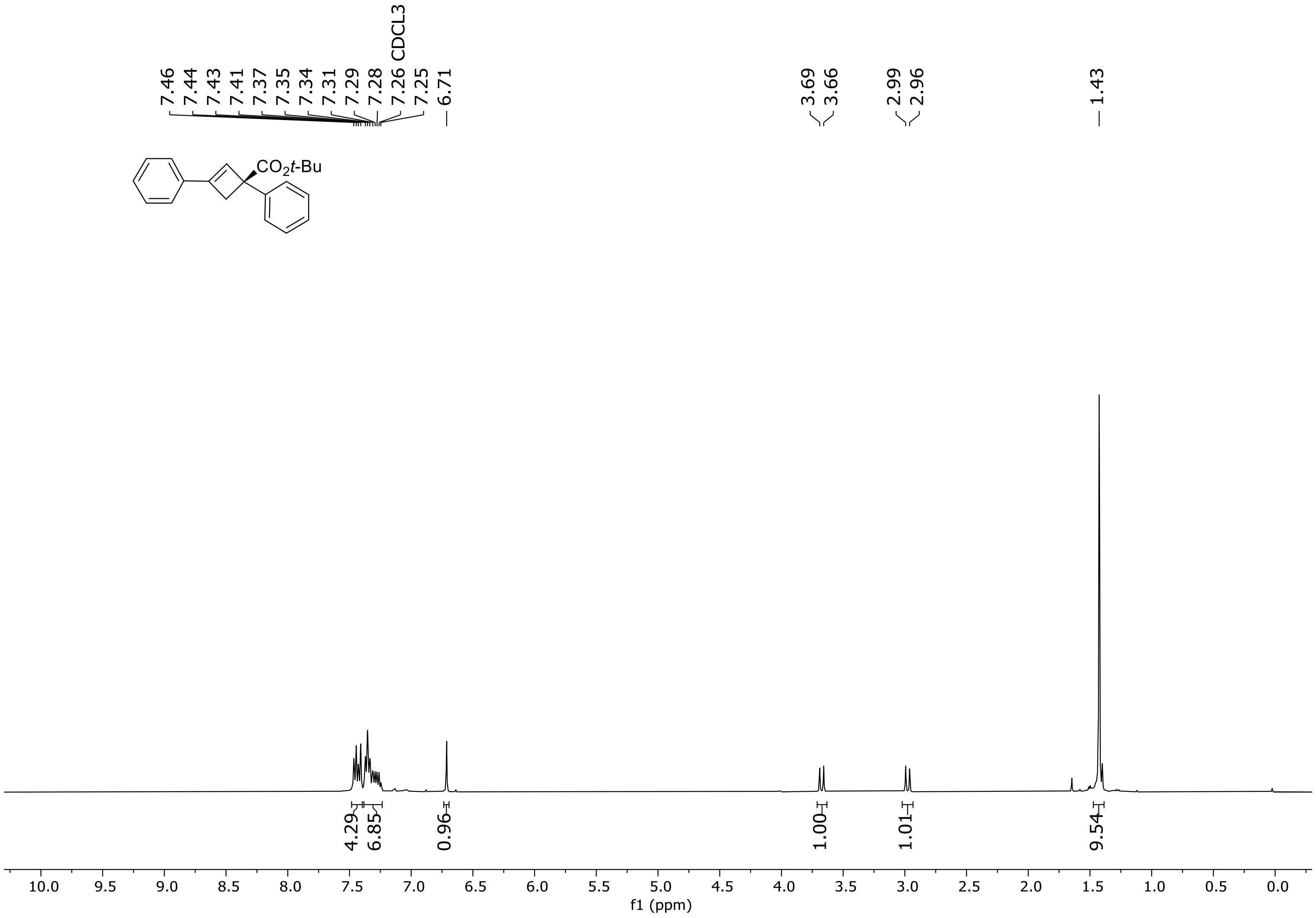

^{13}C NMR (100 MHz, $CDCl_3$, 298 K) spectrum of **5n**.

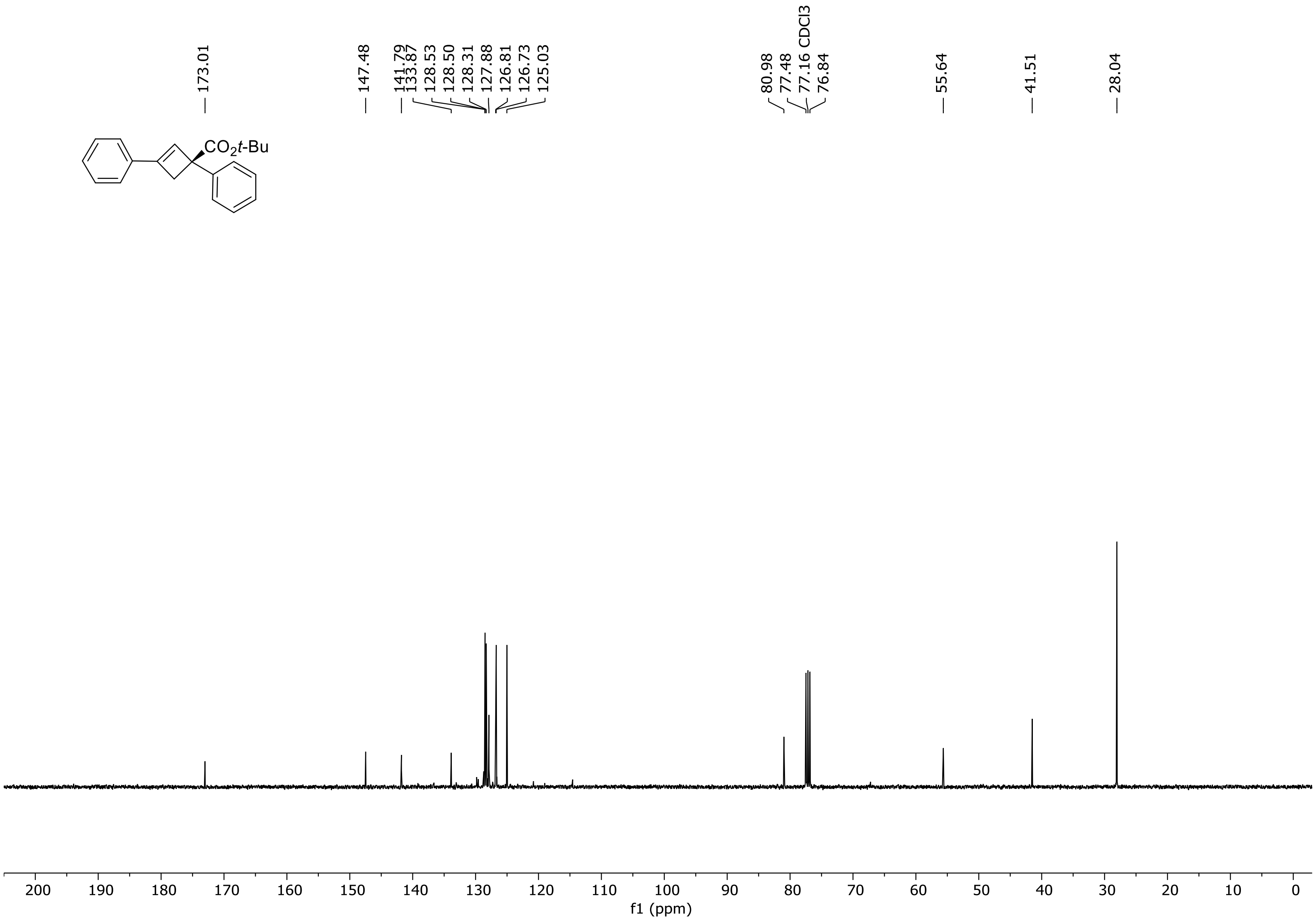

^{1}H NMR (400 MHz, $CDCl_3$, 298 K) spectrum of **5o**.

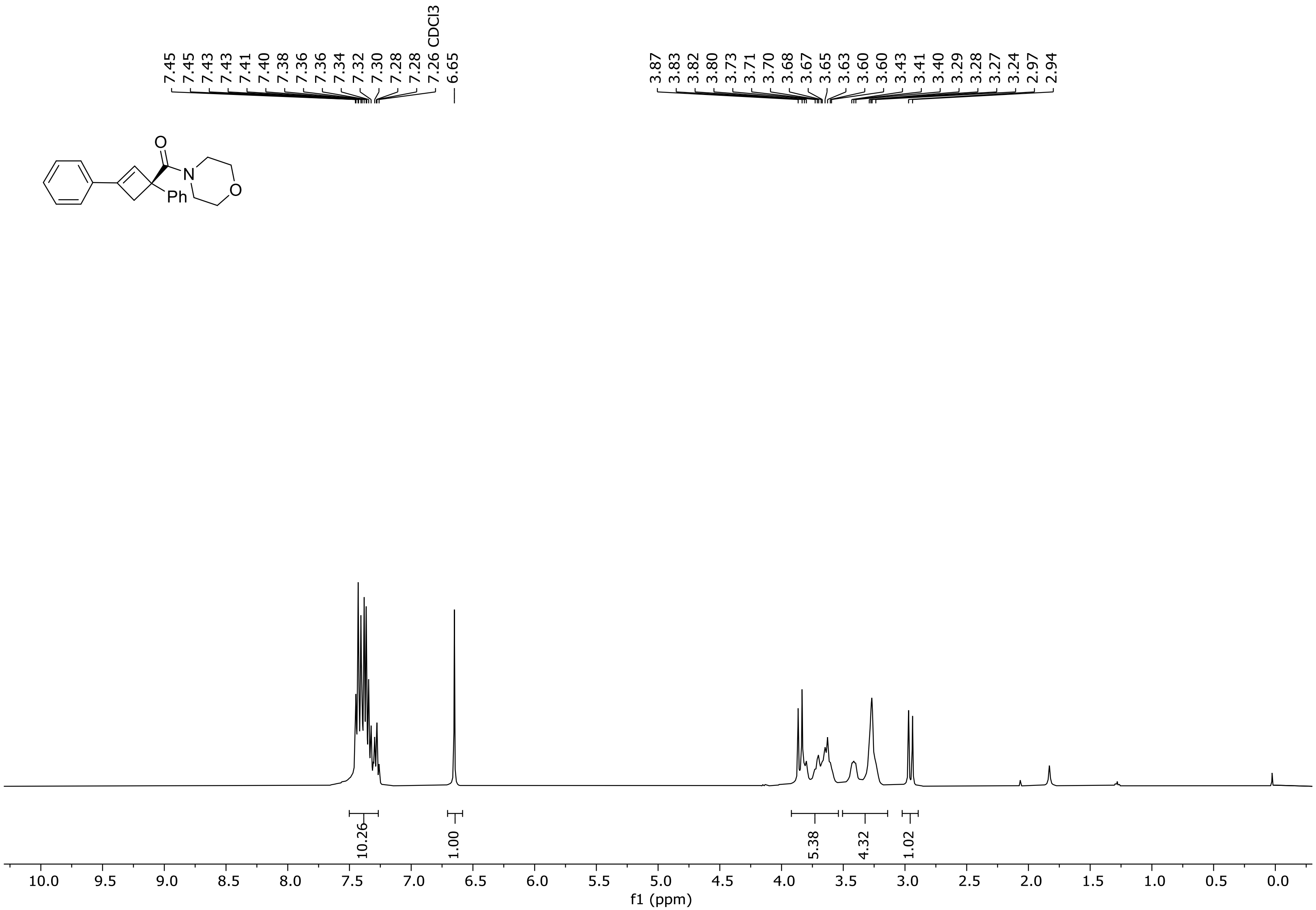

^{13}C NMR (100 MHz, $CDCl_3$, 298 K) spectrum of **5o**.

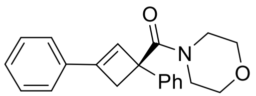

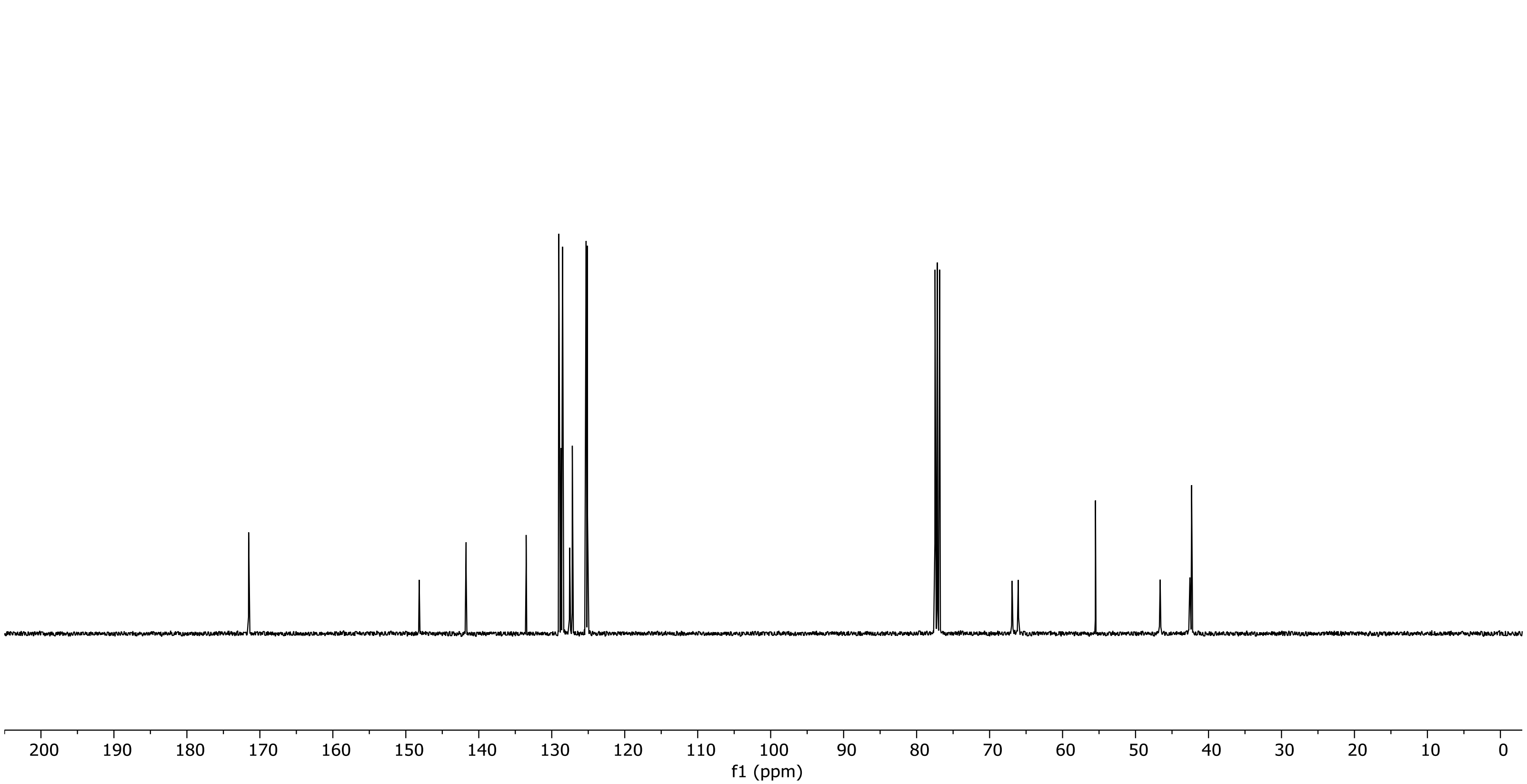

^{1}H NMR (400 MHz, $CDCl_3$, 298 K) spectrum of **5p**.

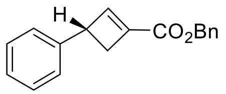

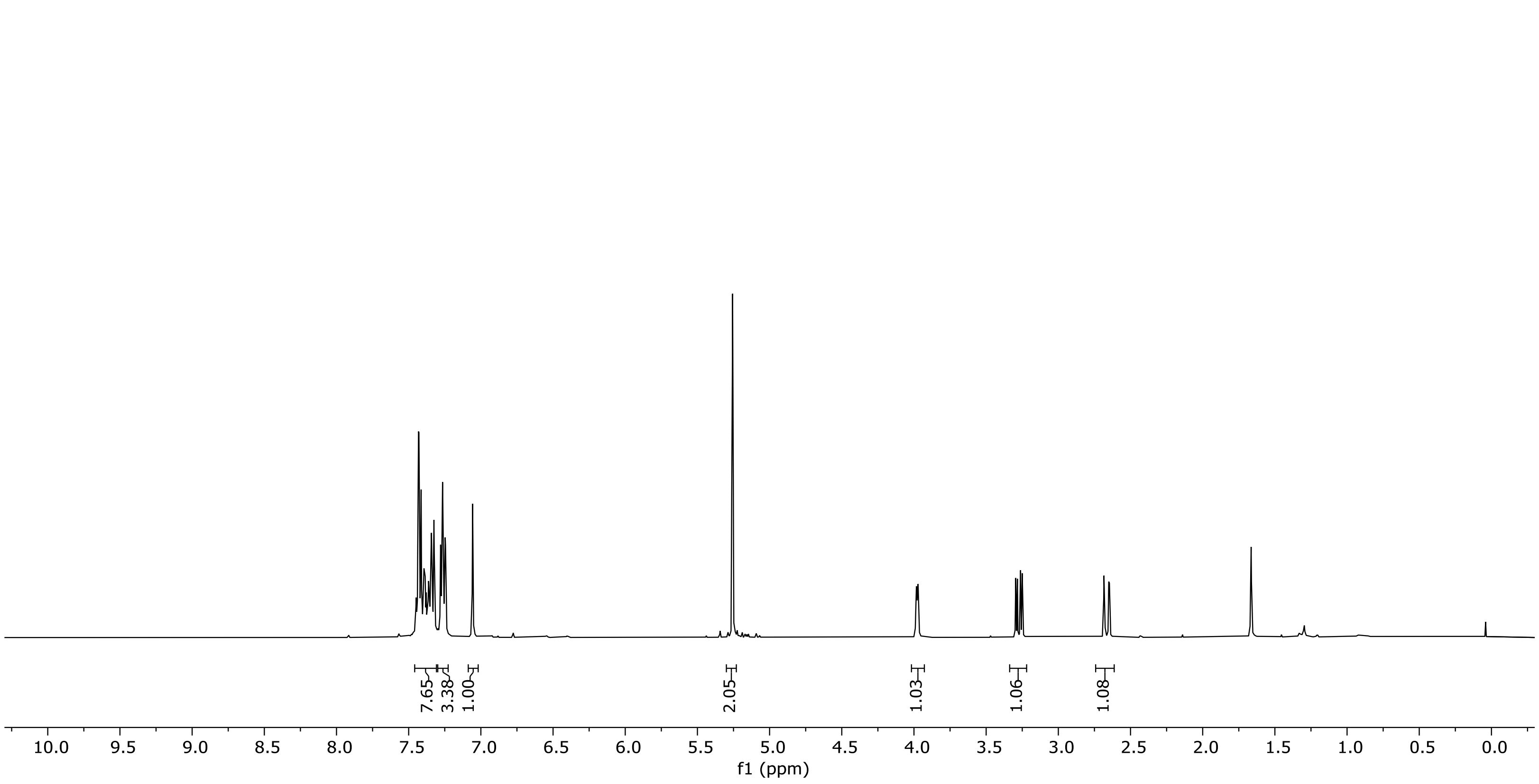

^{13}C NMR (100 MHz, $CDCl_3$, 298 K) spectrum of **5p**.

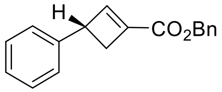

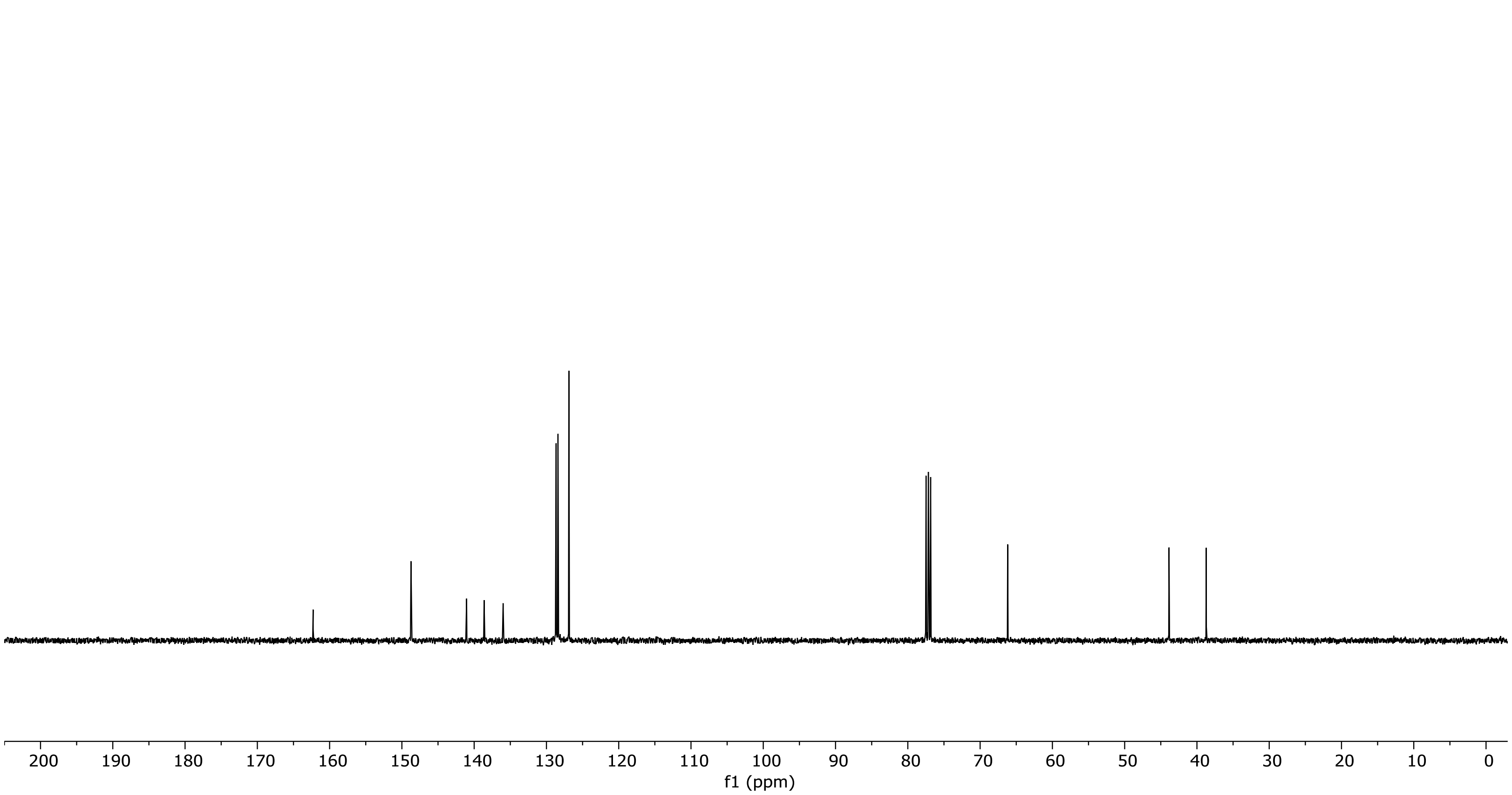

COSY (^{1}H–^{1}H) NMR (400 MHz, $CDCl_3$, 298 K) spectrum of **5p**.

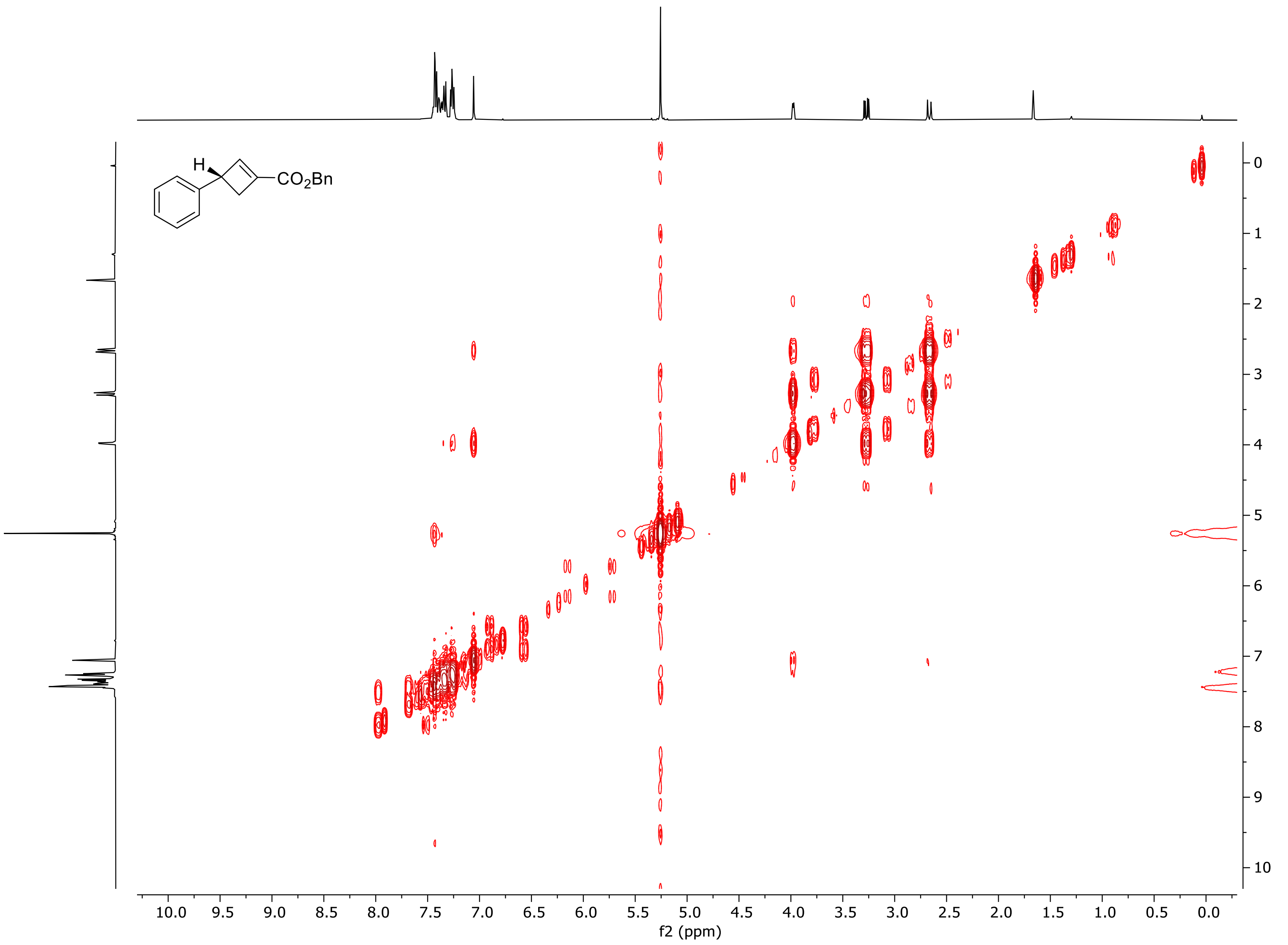

HSQC (^1H–^{13}C) NMR (400 MHz, $CDCl_3$, 298 K) spectrum of **5p**.

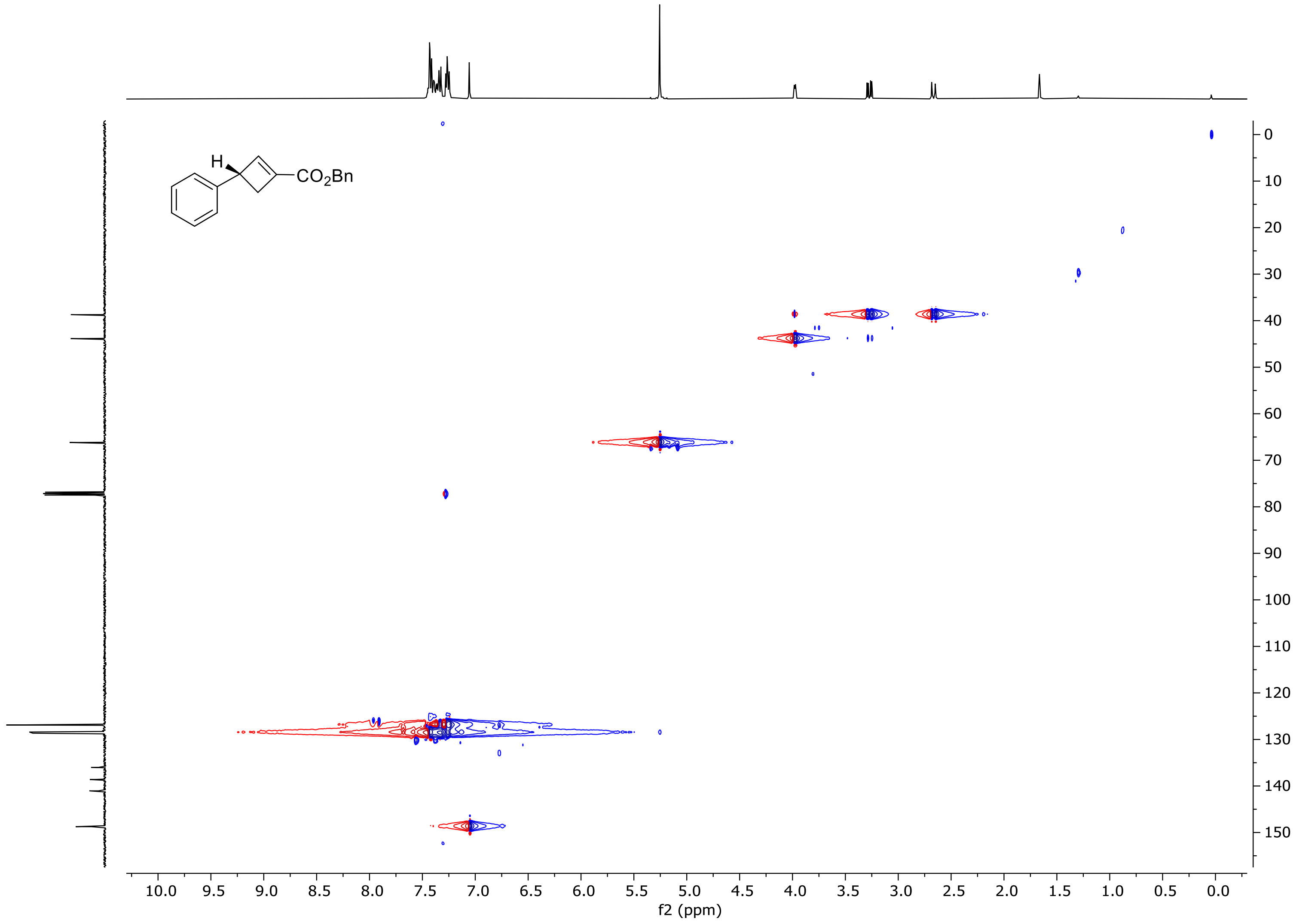

^{1}H NMR (400 MHz, $CDCl_3$, 298 K) spectrum of **5q**.

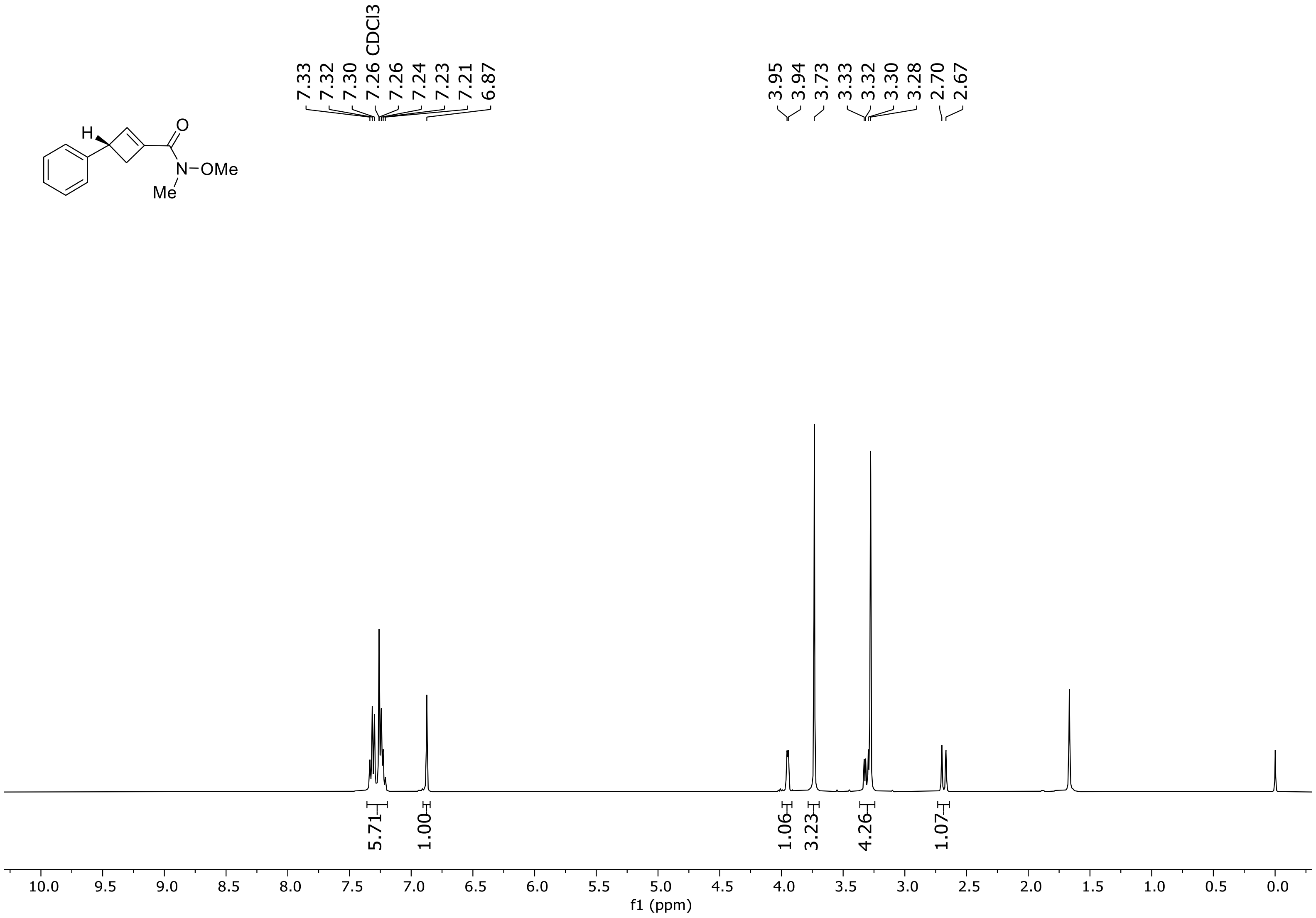

^{13}C NMR (100 MHz, $CDCl_3$, 298 K) spectrum of **5q**.

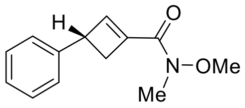

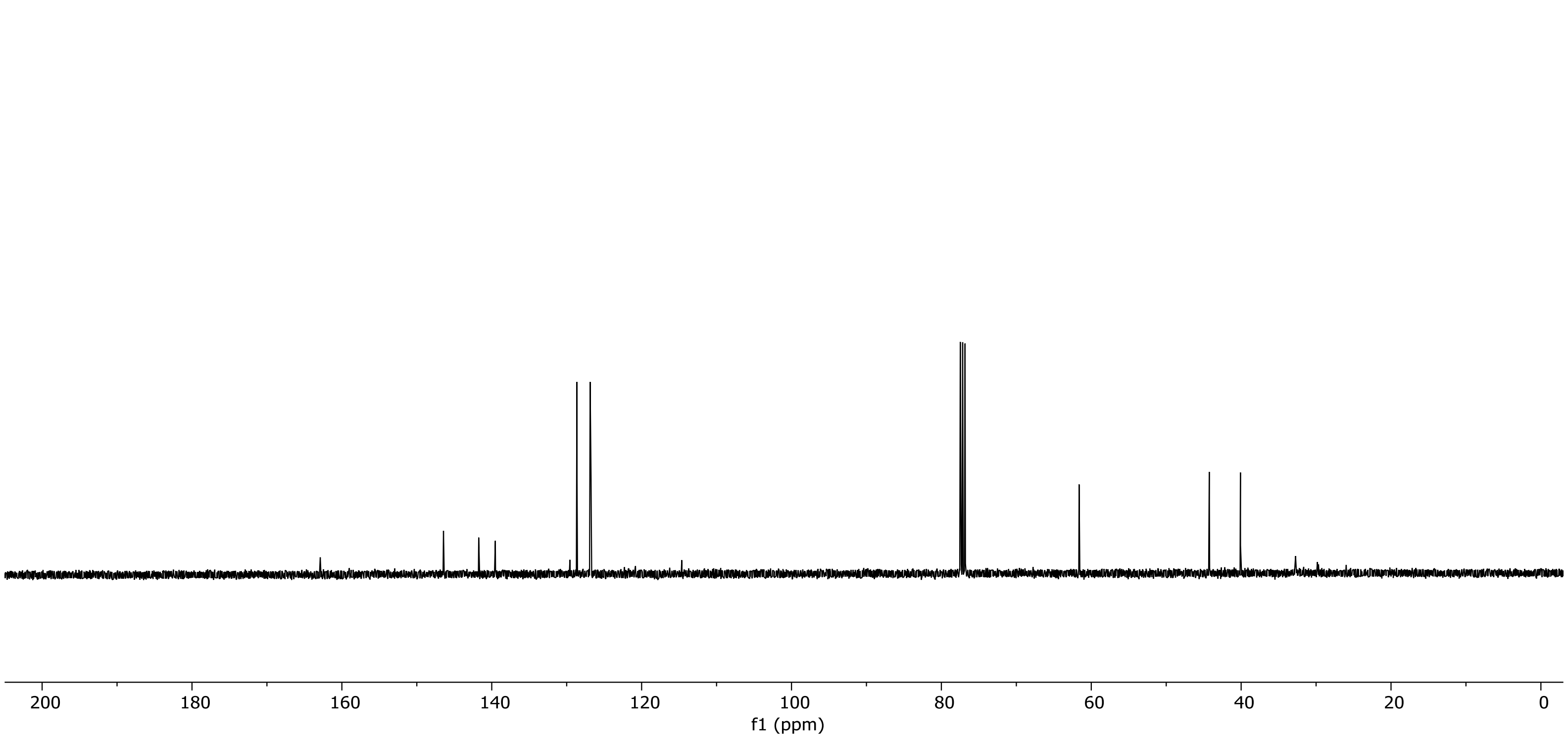

^{1}H NMR (400 MHz, $CDCl_3$, 298 K) spectrum of **5r**.

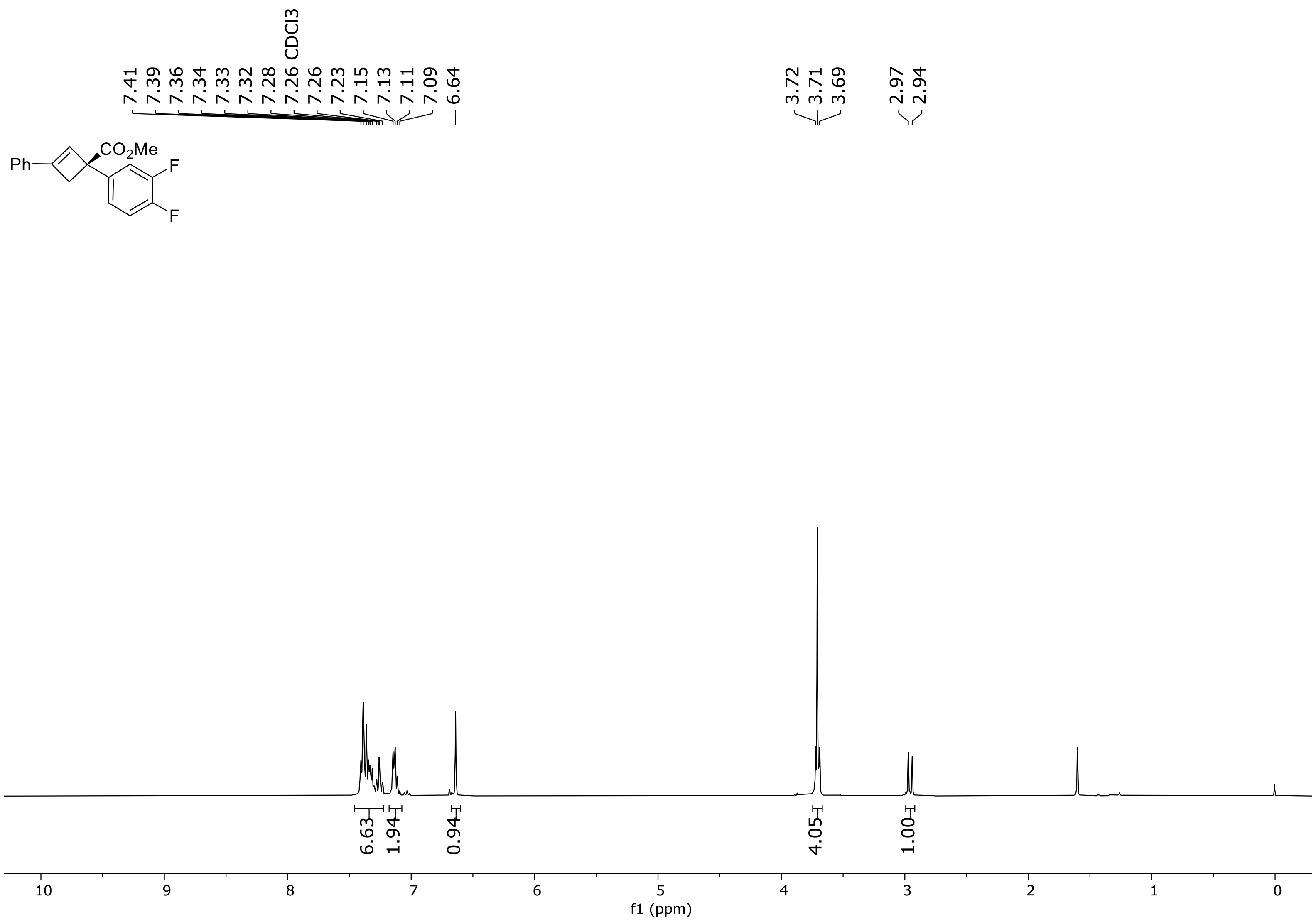

^{13}C NMR (100 MHz, $CDCl_3$, 298 K) spectrum of **5r**.

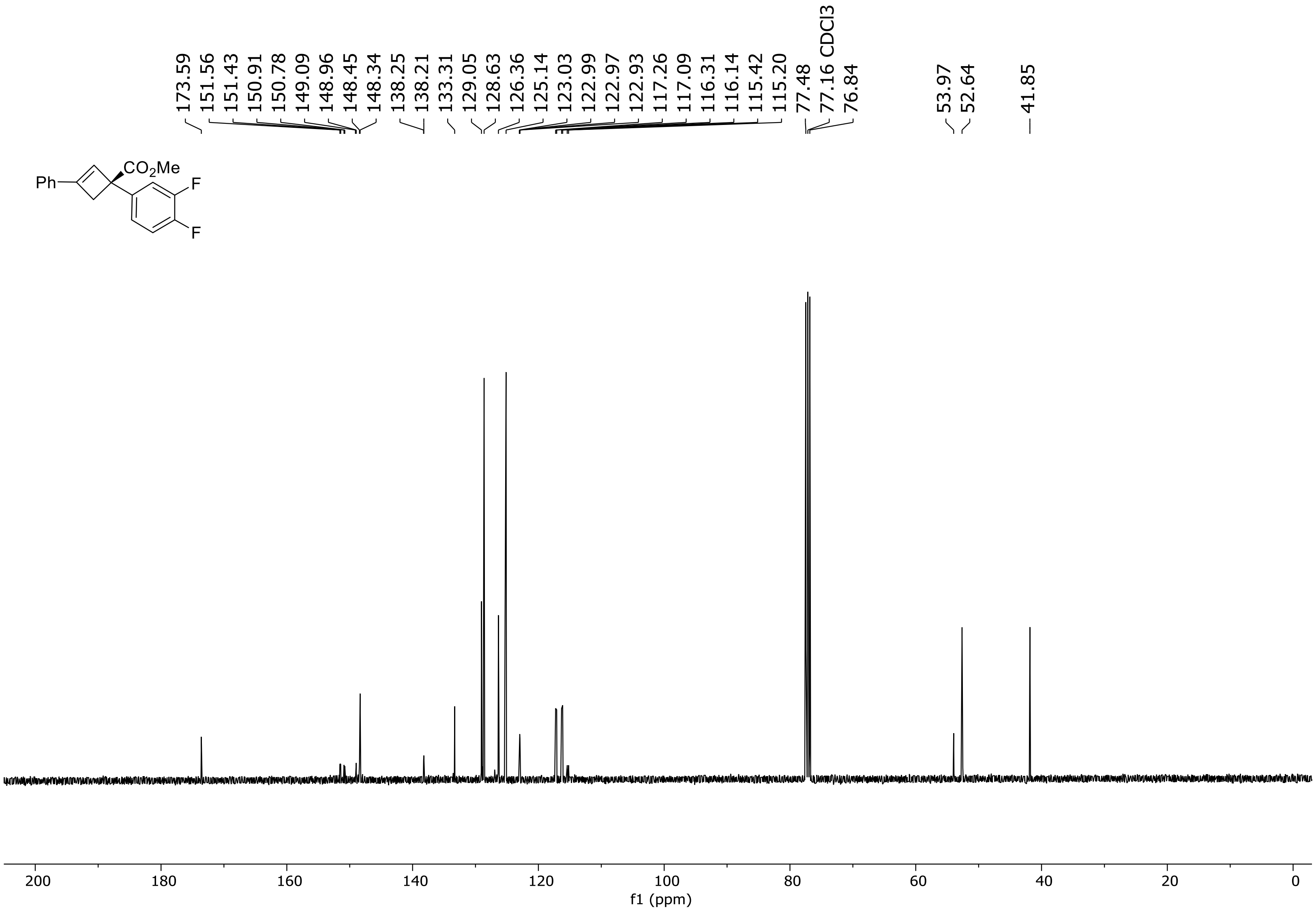

^{19}F NMR (377 MHz, $CDCl_3$, 298 K) spectrum of **5r**.

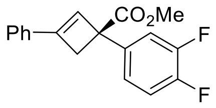

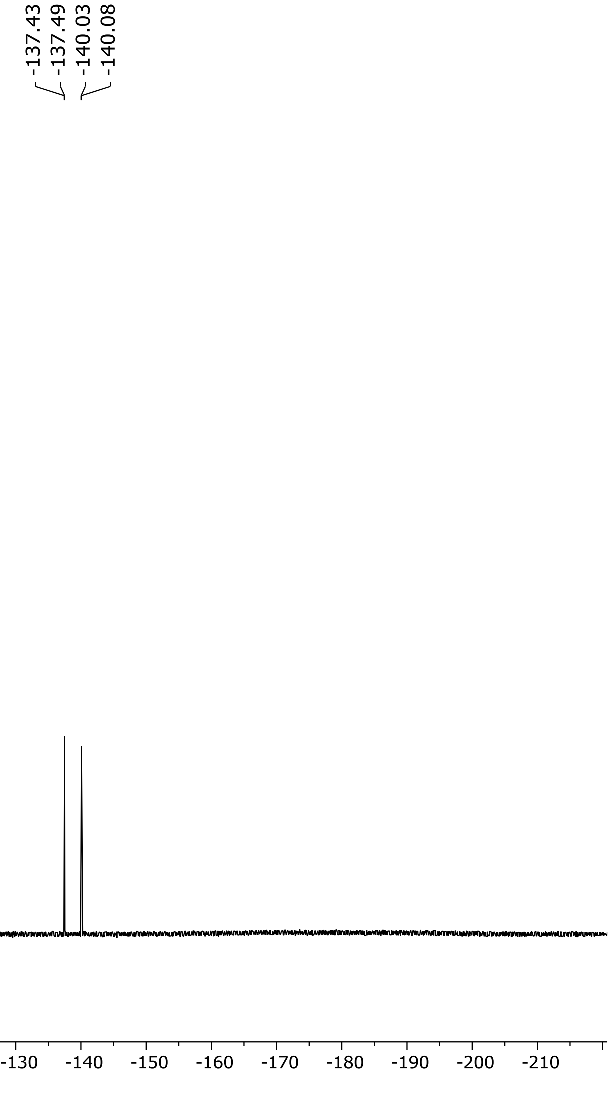

^{1}H NMR (400 MHz, $CDCl_3$, 298 K) spectrum of **5s**.

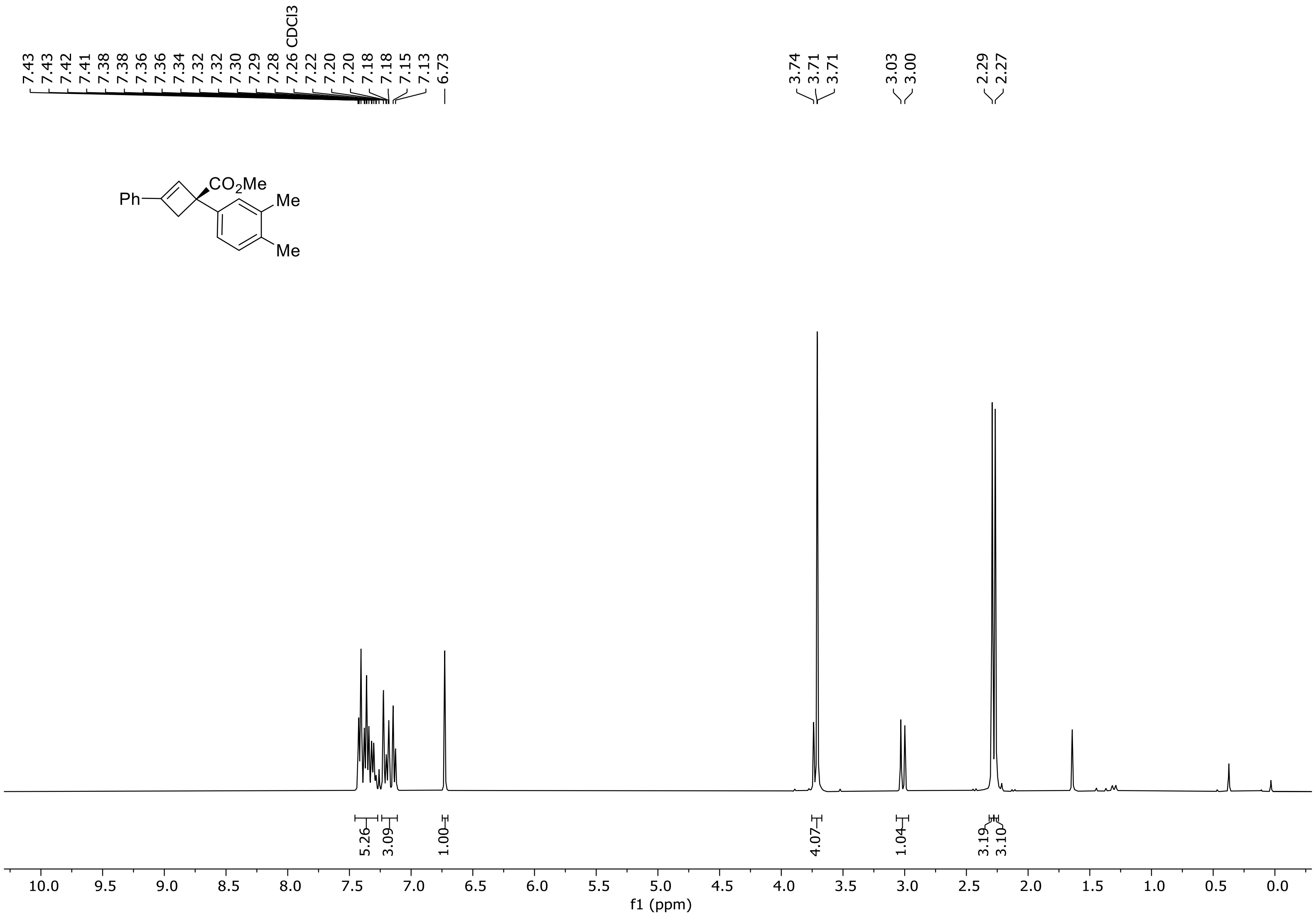

^{13}C NMR (100 MHz, $CDCl_3$, 298 K) spectrum of **5s**.

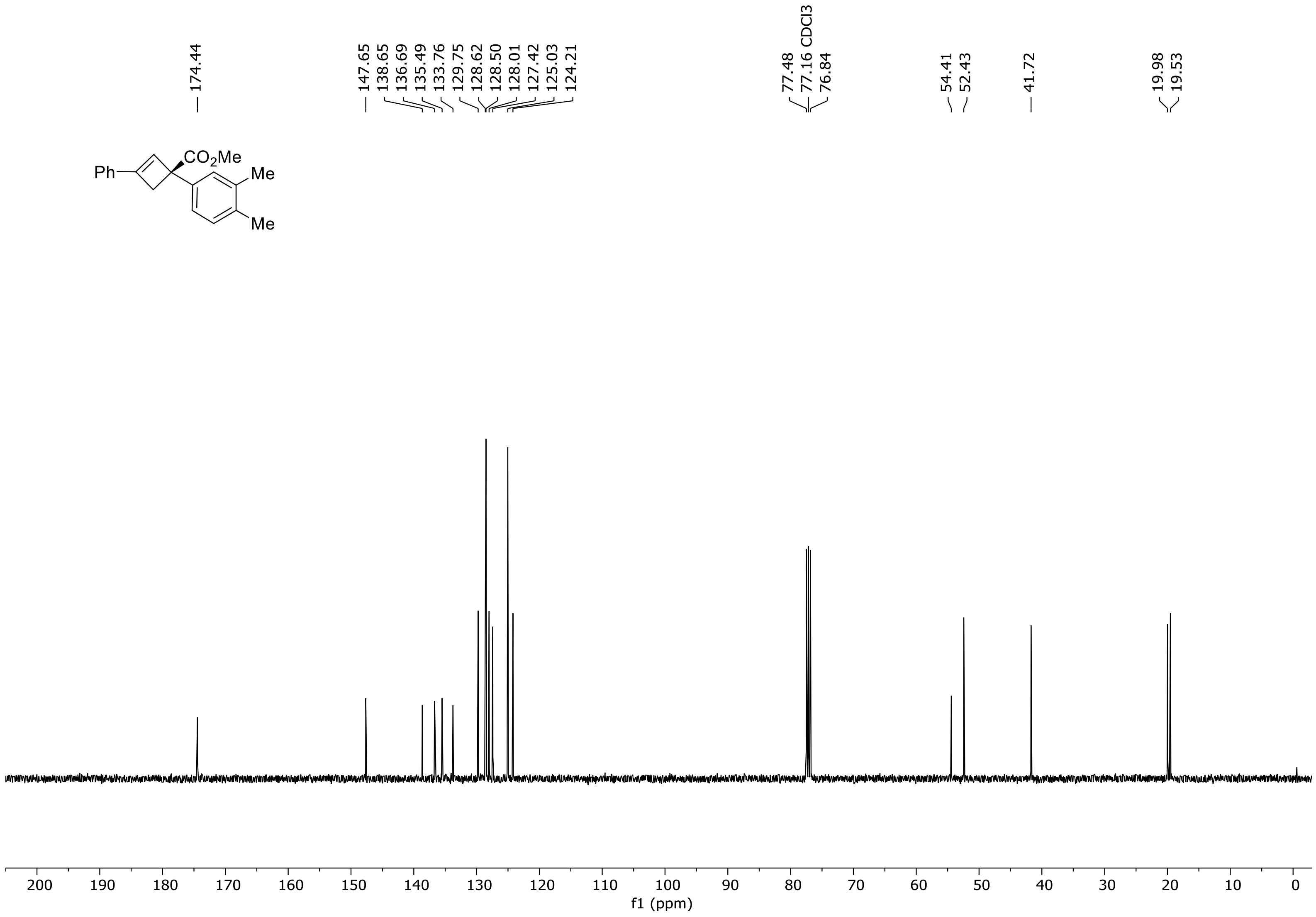

^{1}H NMR (400 MHz, $CDCl_3$, 298 K) spectrum of **5t**.

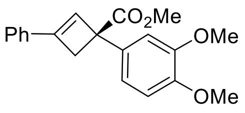

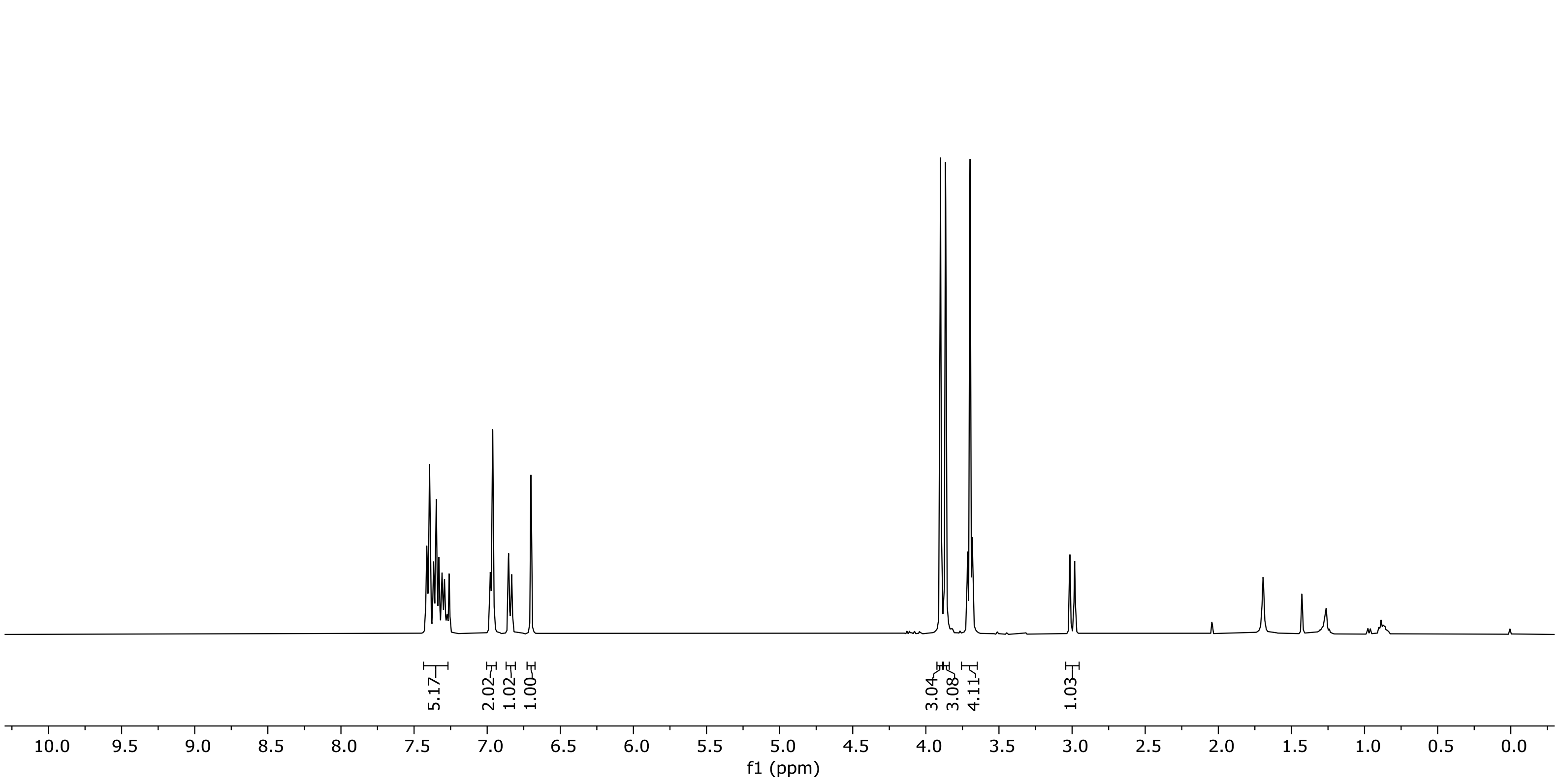

^{13}C NMR (100 MHz, $CDCl_3$, 298 K) spectrum of **5t**.

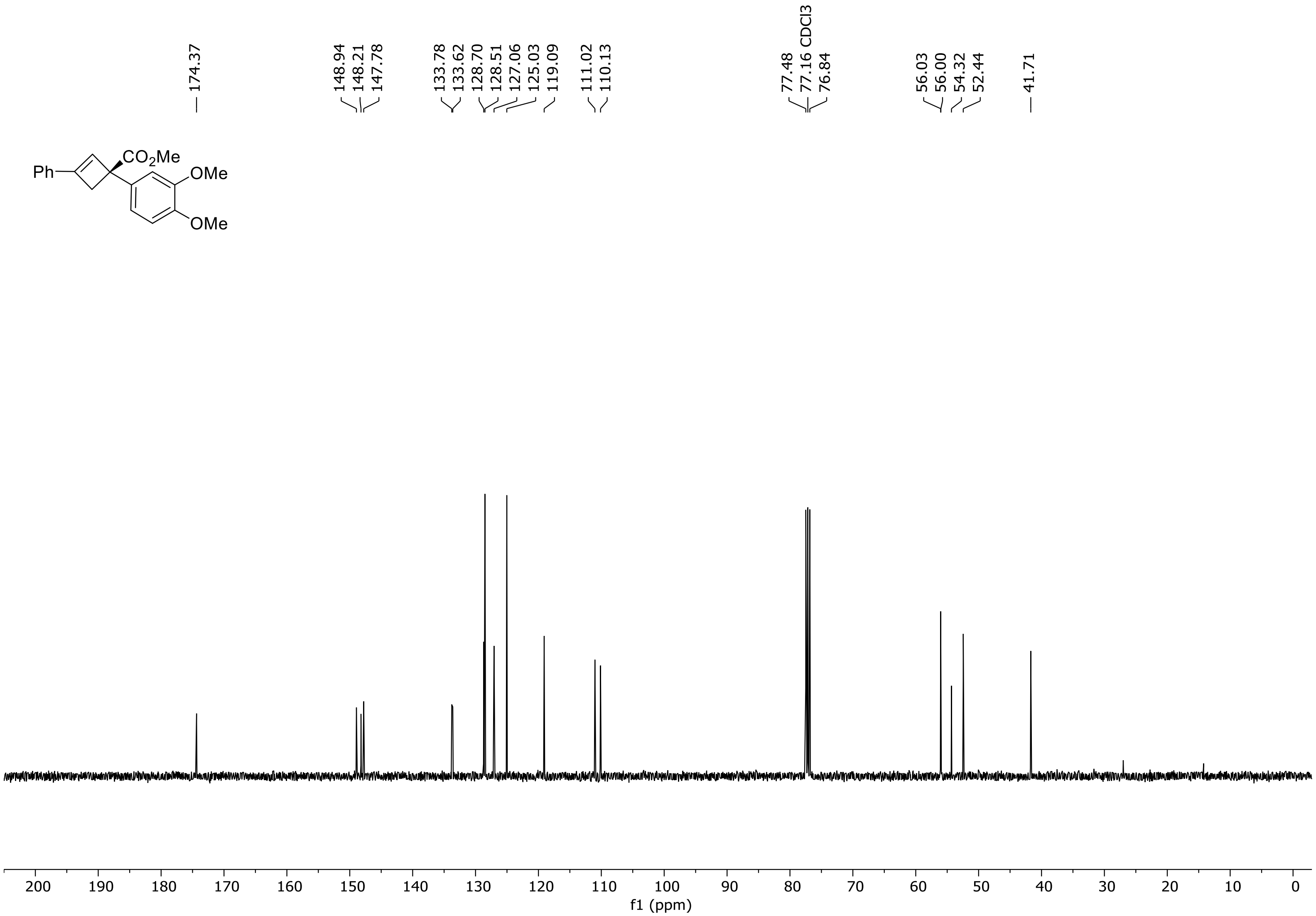

^{1}H NMR (400 MHz, $CDCl_3$, 298 K) spectrum of **5u**.

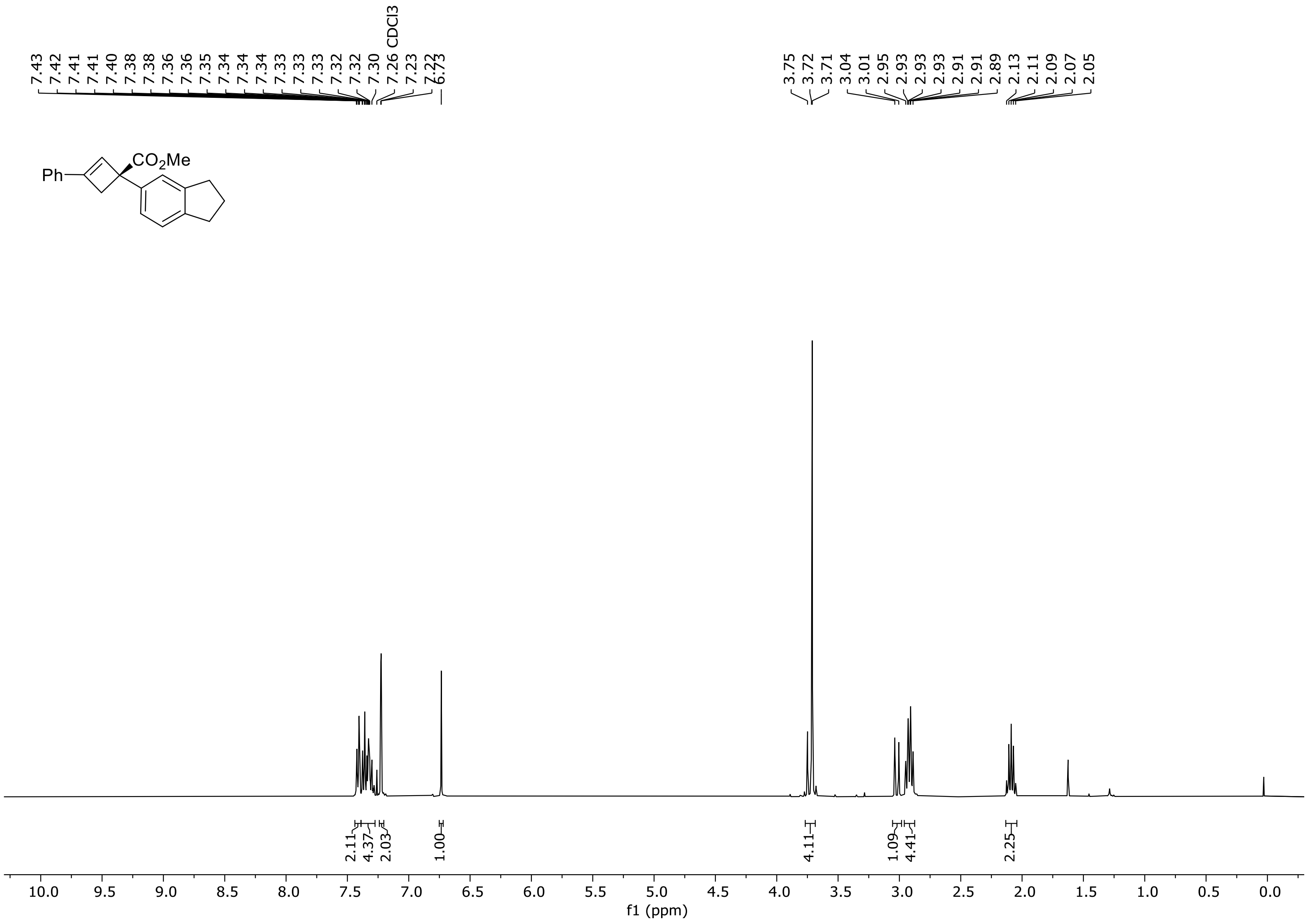

^{13}C NMR (100 MHz, $CDCl_3$, 298 K) spectrum of **5u**.

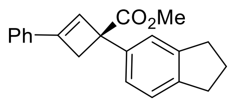

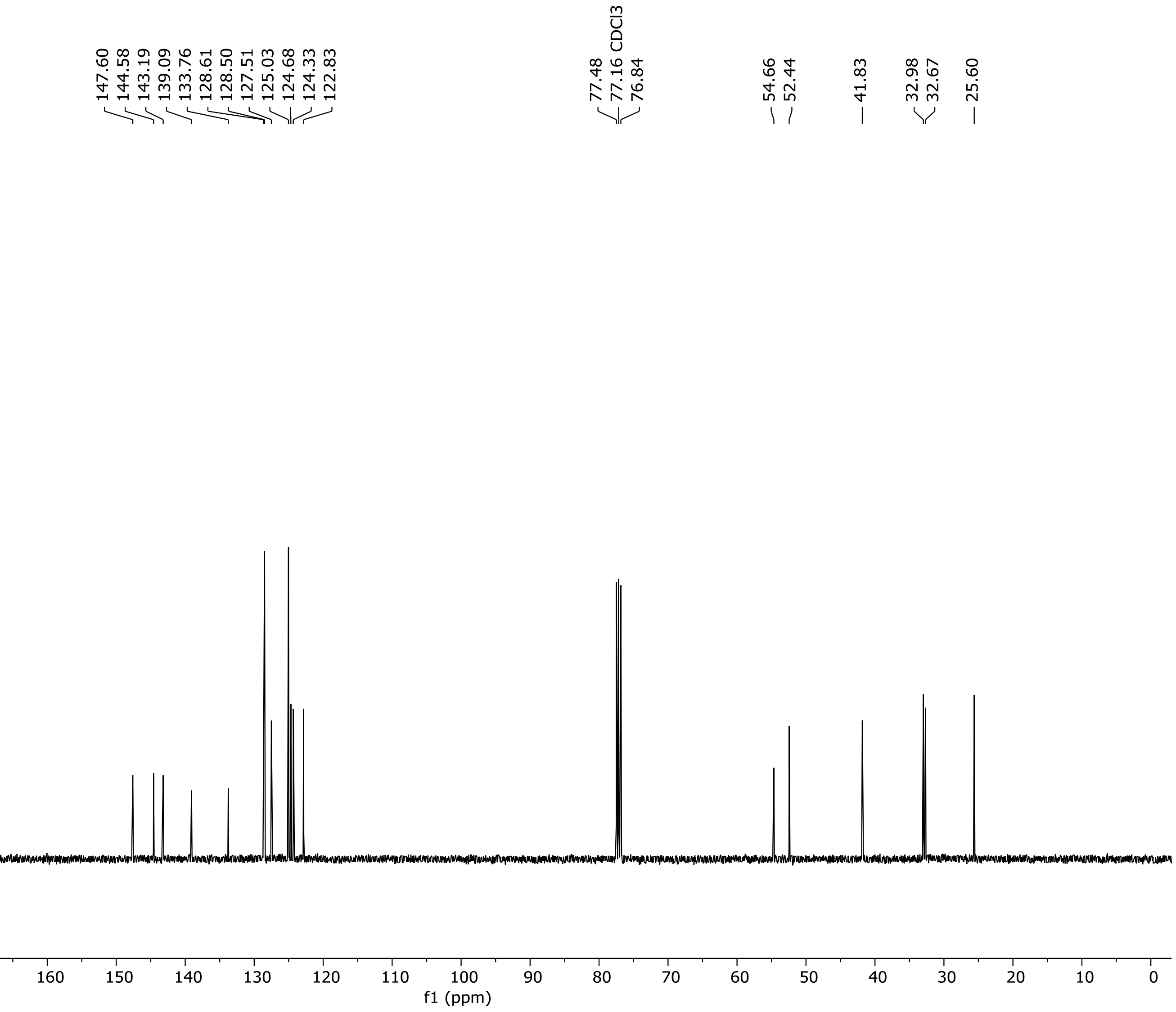

^{1}H NMR (400 MHz, $CDCl_3$, 298 K) spectrum of **5v**.

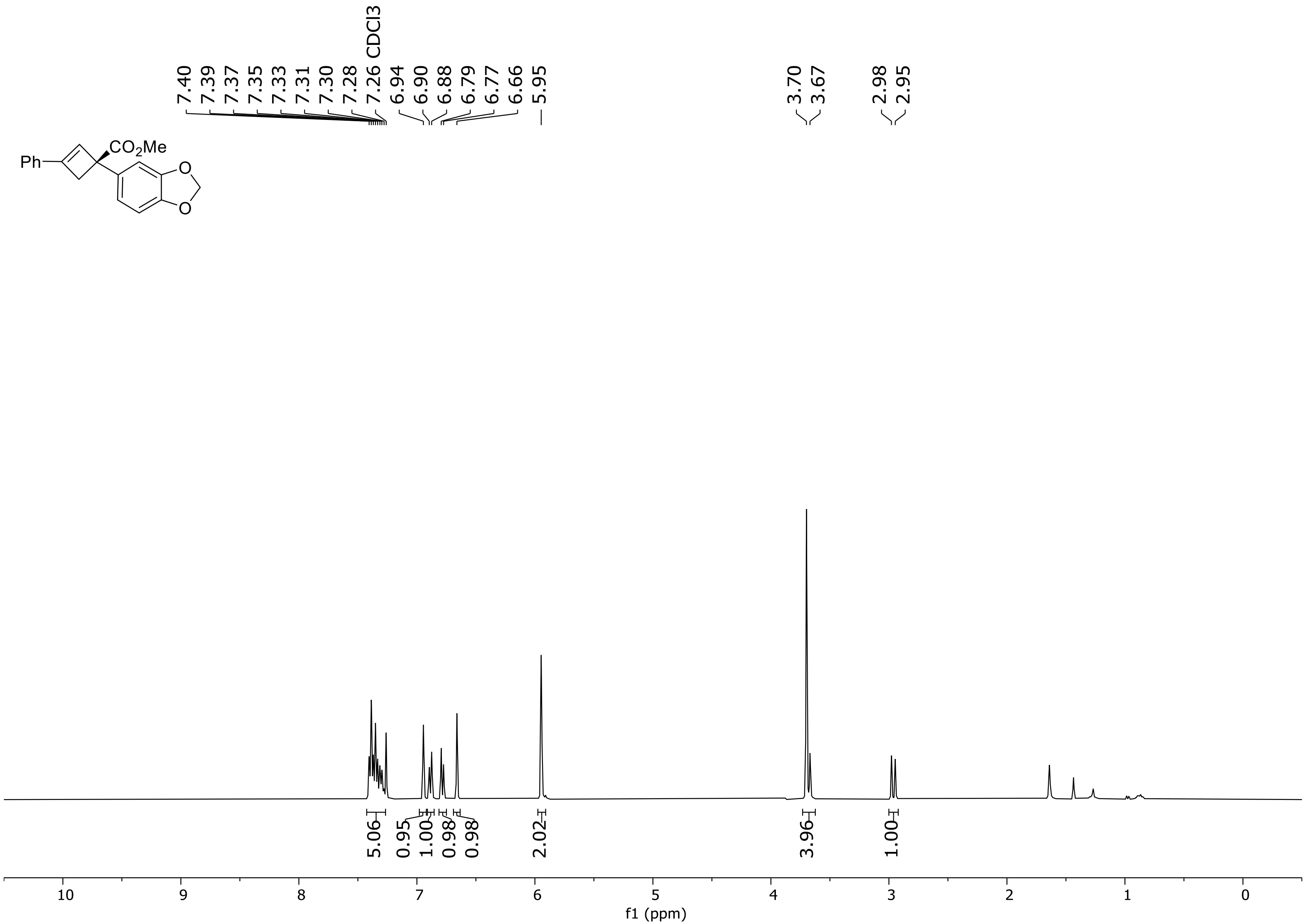

^{13}C NMR (100 MHz, $CDCl_3$, 298 K) spectrum of **5v**.

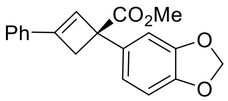

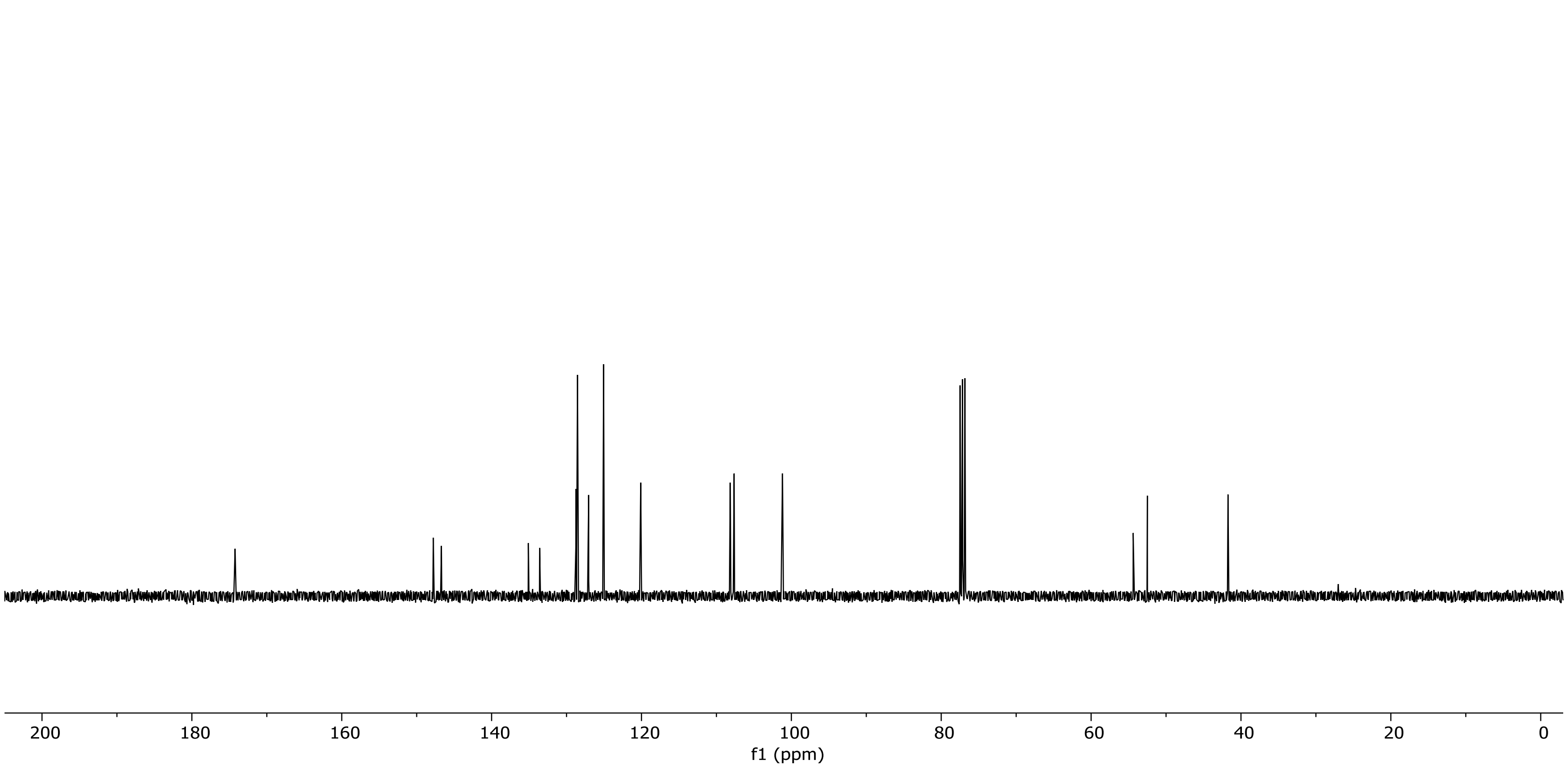

^{1}H NMR (400 MHz, $CDCl_3$, 298 K) spectrum of **5w**.

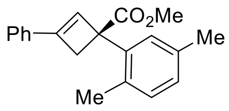

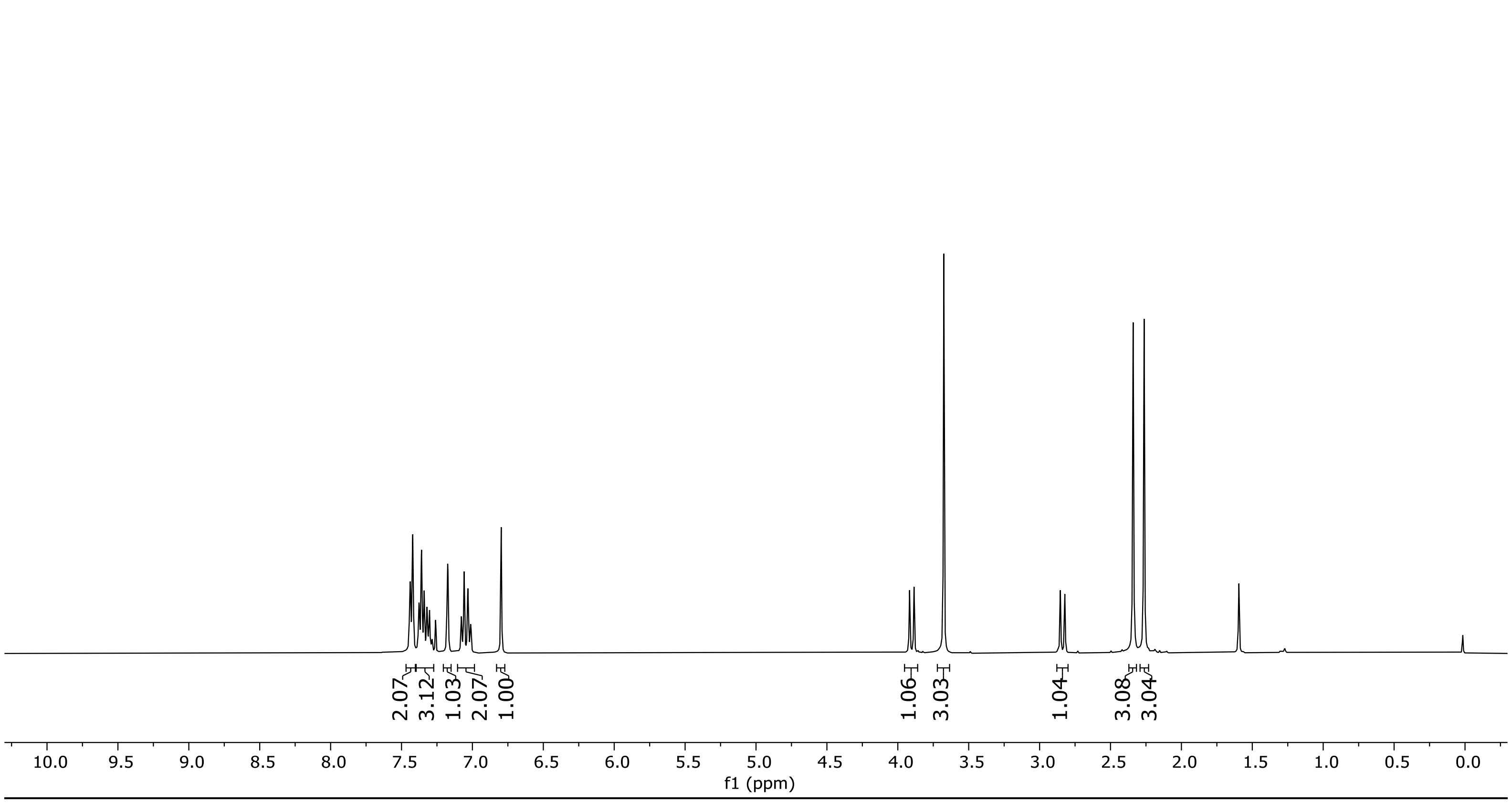

^{13}C NMR (100 MHz, $CDCl_3$, 298 K) spectrum of **5w**.

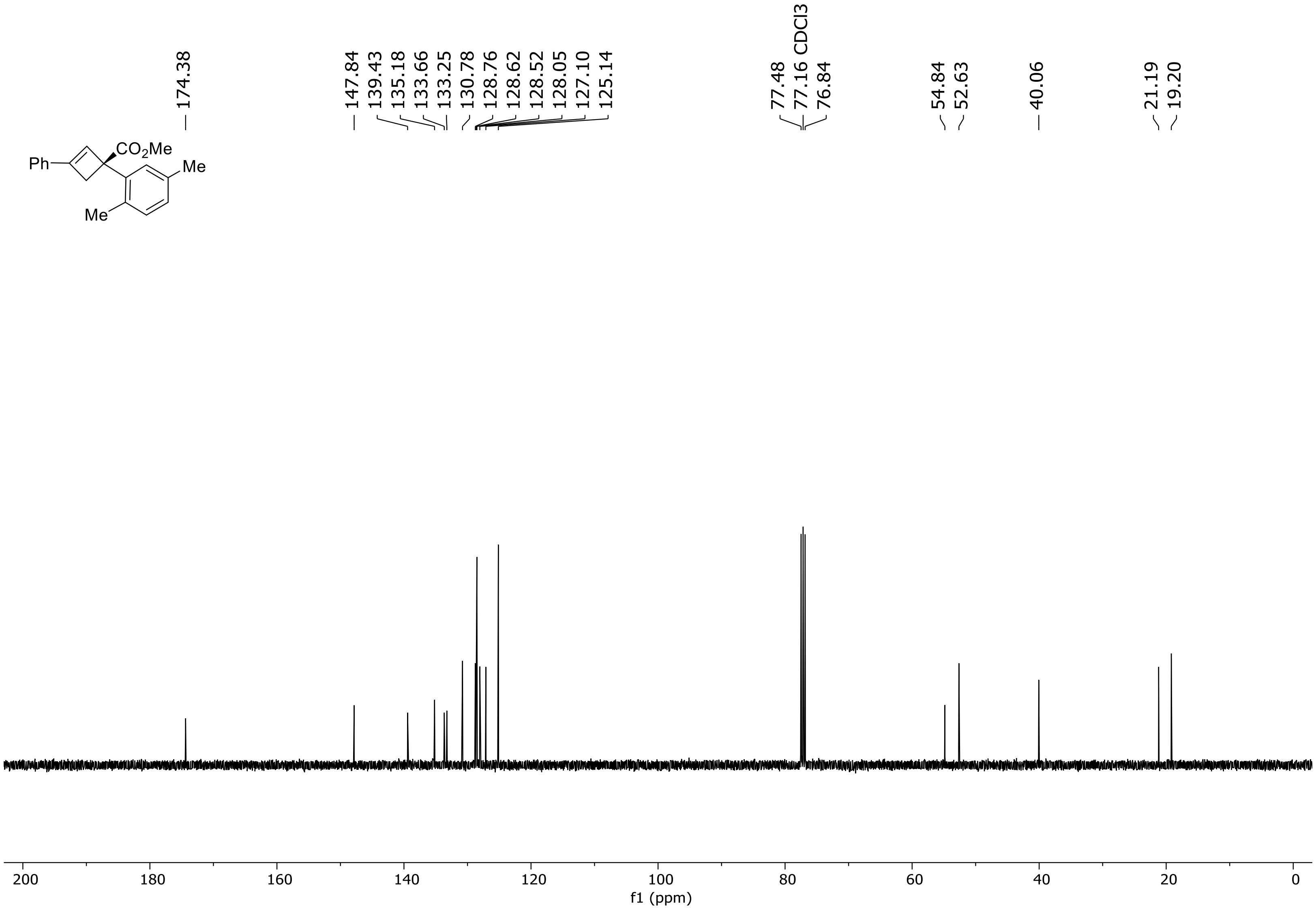

^{1}H NMR (400 MHz, $CDCl_3$, 298 K) spectrum of **5x**.

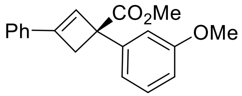

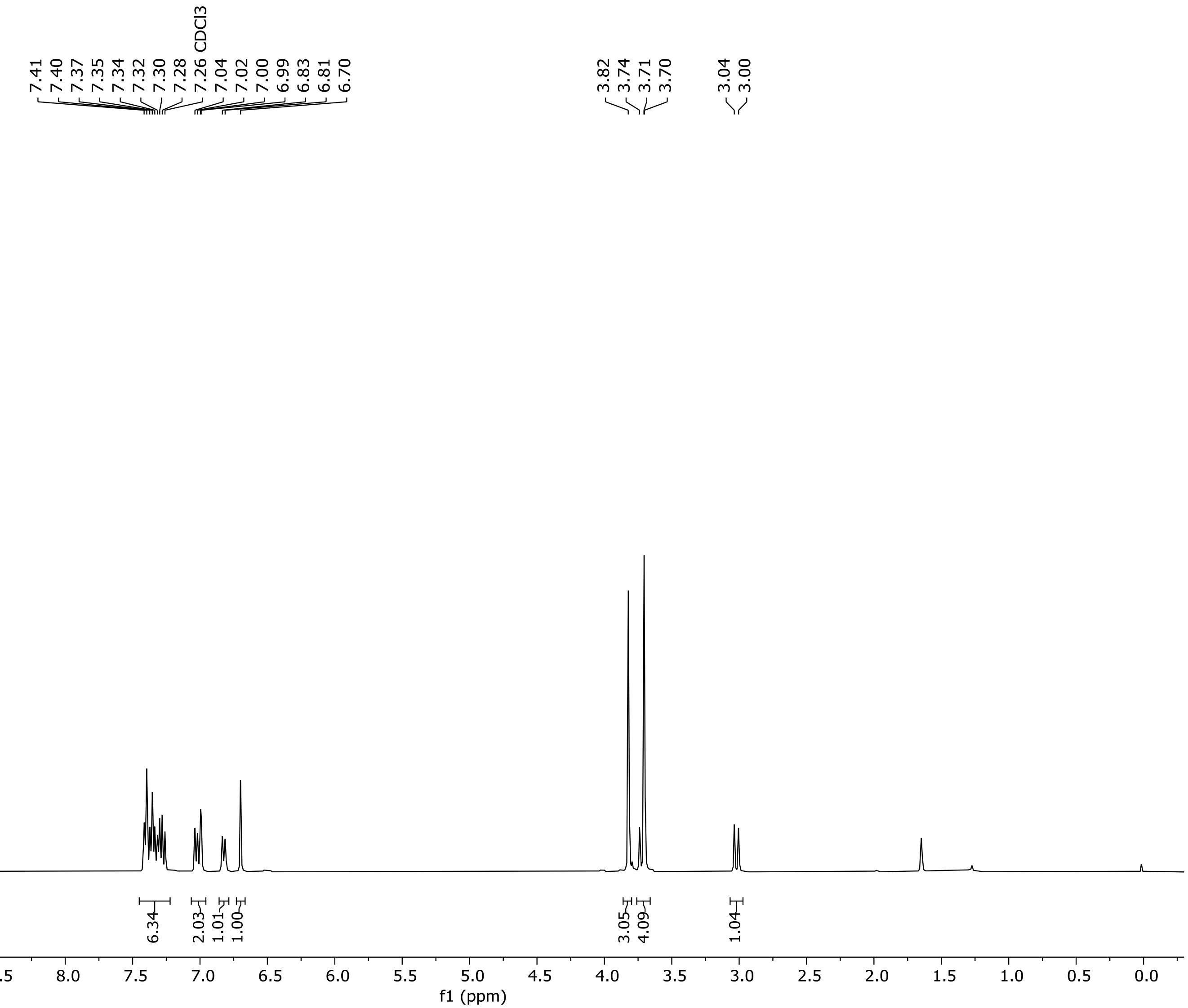

^{13}C NMR (100 MHz, $CDCl_3$, 298 K) spectrum of **5x**.

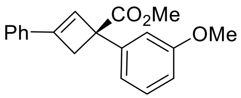

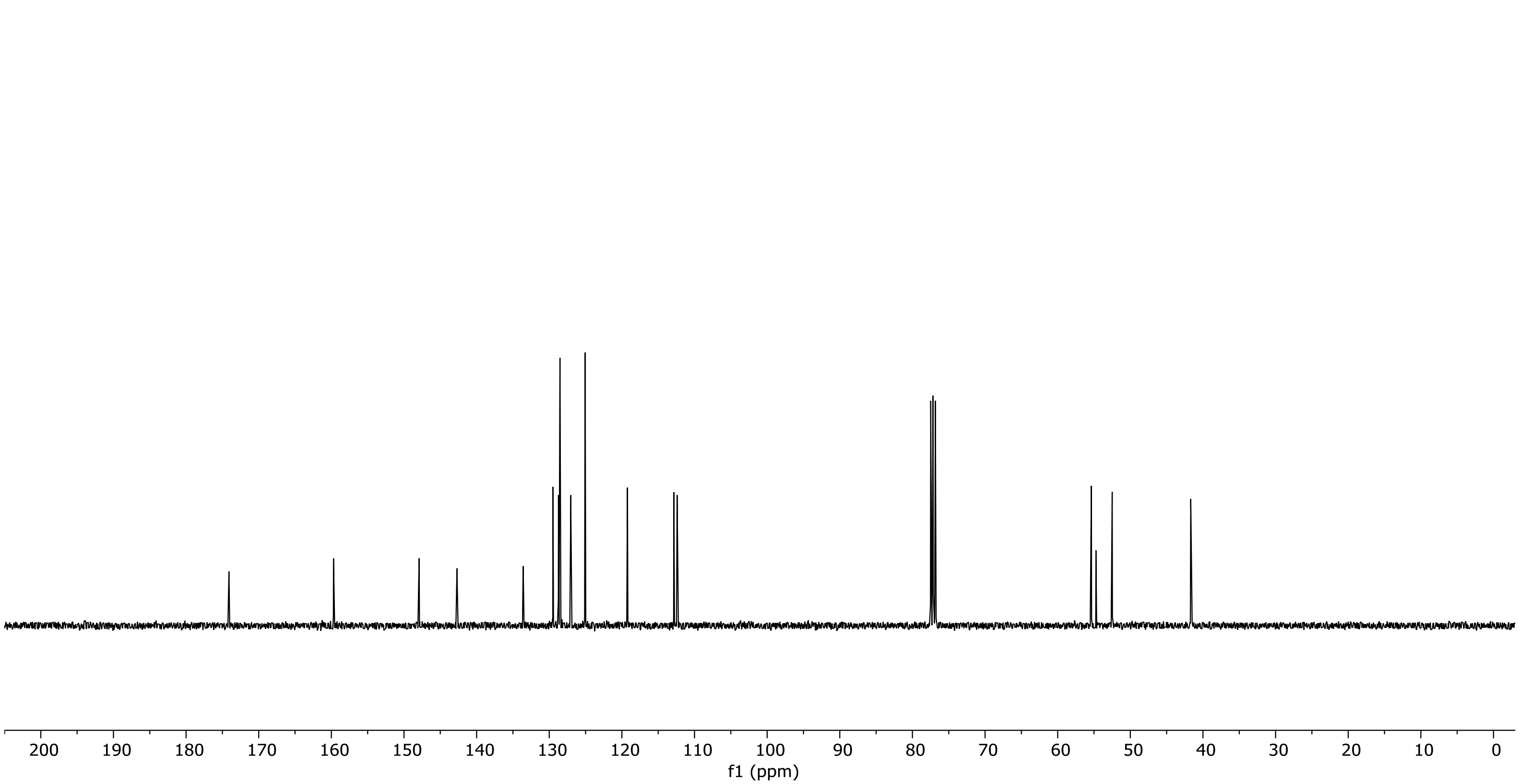

^{1}H NMR (400 MHz, $CDCl_3$, 298 K) spectrum of **5y** and **5y'**.

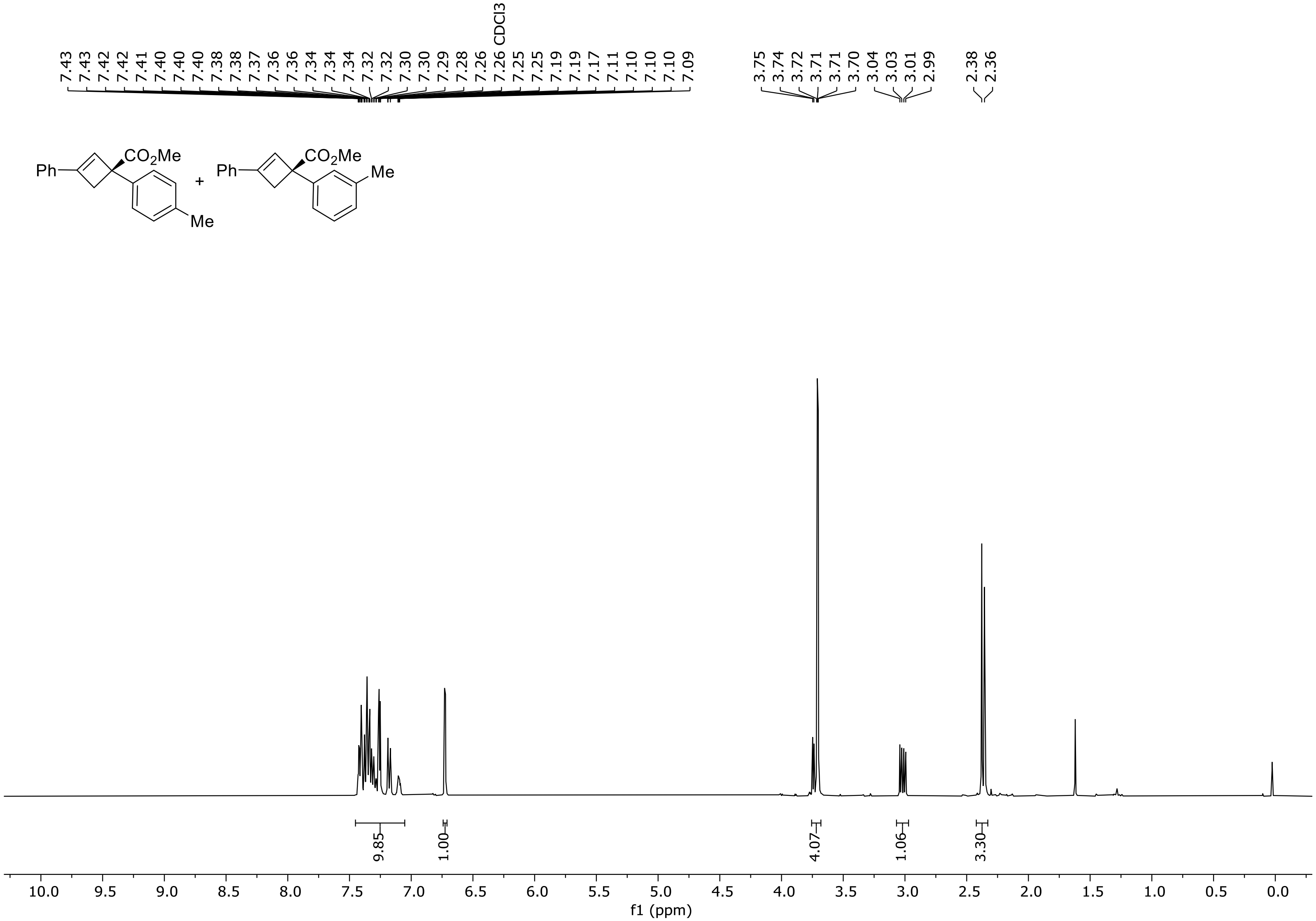

^{13}C NMR (100 MHz, $CDCl_3$, 298 K) spectrum of **5y** and **5y'**.

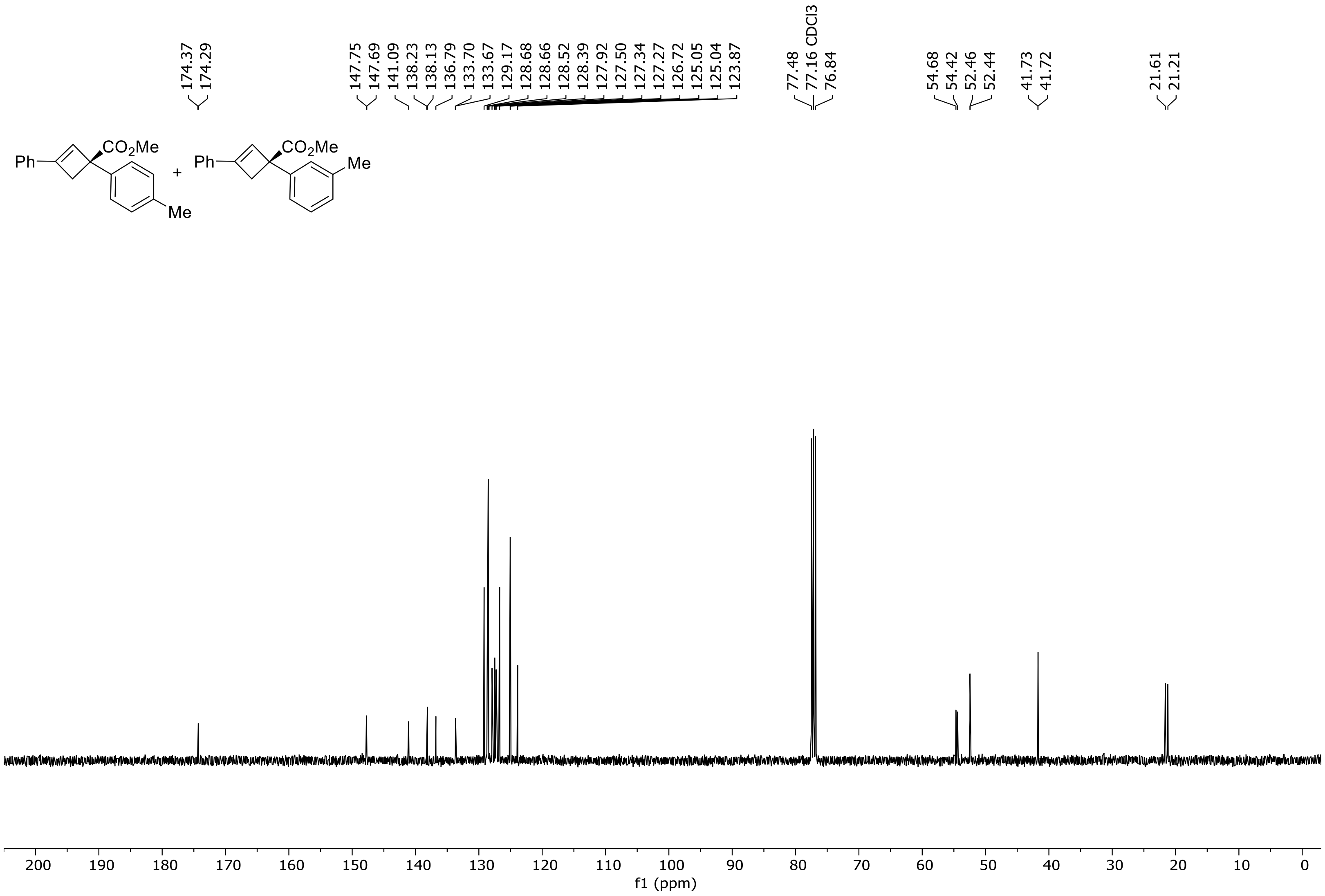

^{1}H NMR (400 MHz, $CDCl_3$, 298 K) spectrum of **5z** and **5z'**.

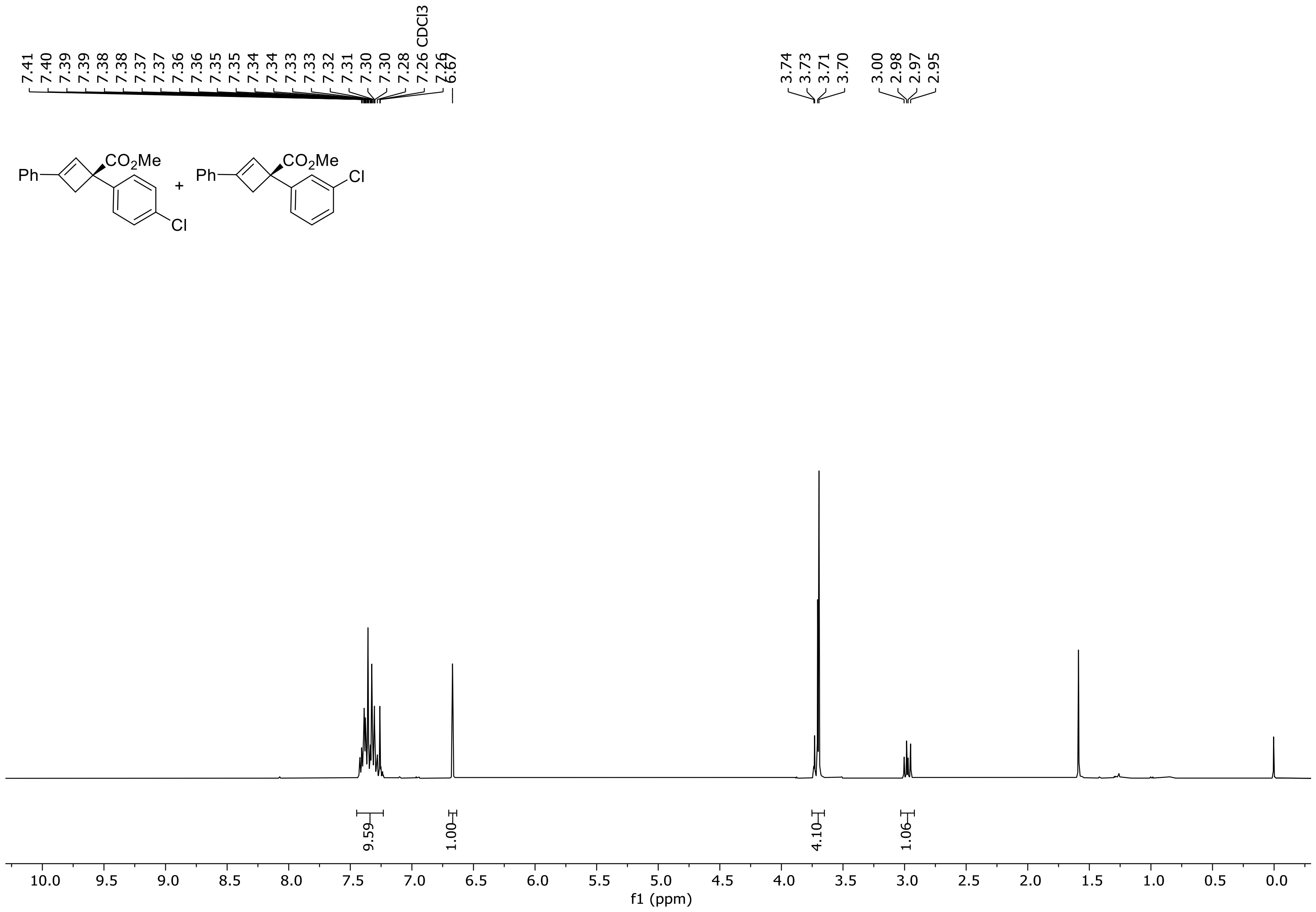

^{13}C NMR (100 MHz, $CDCl_3$, 298 K) spectrum of **5z** and **5z’**.

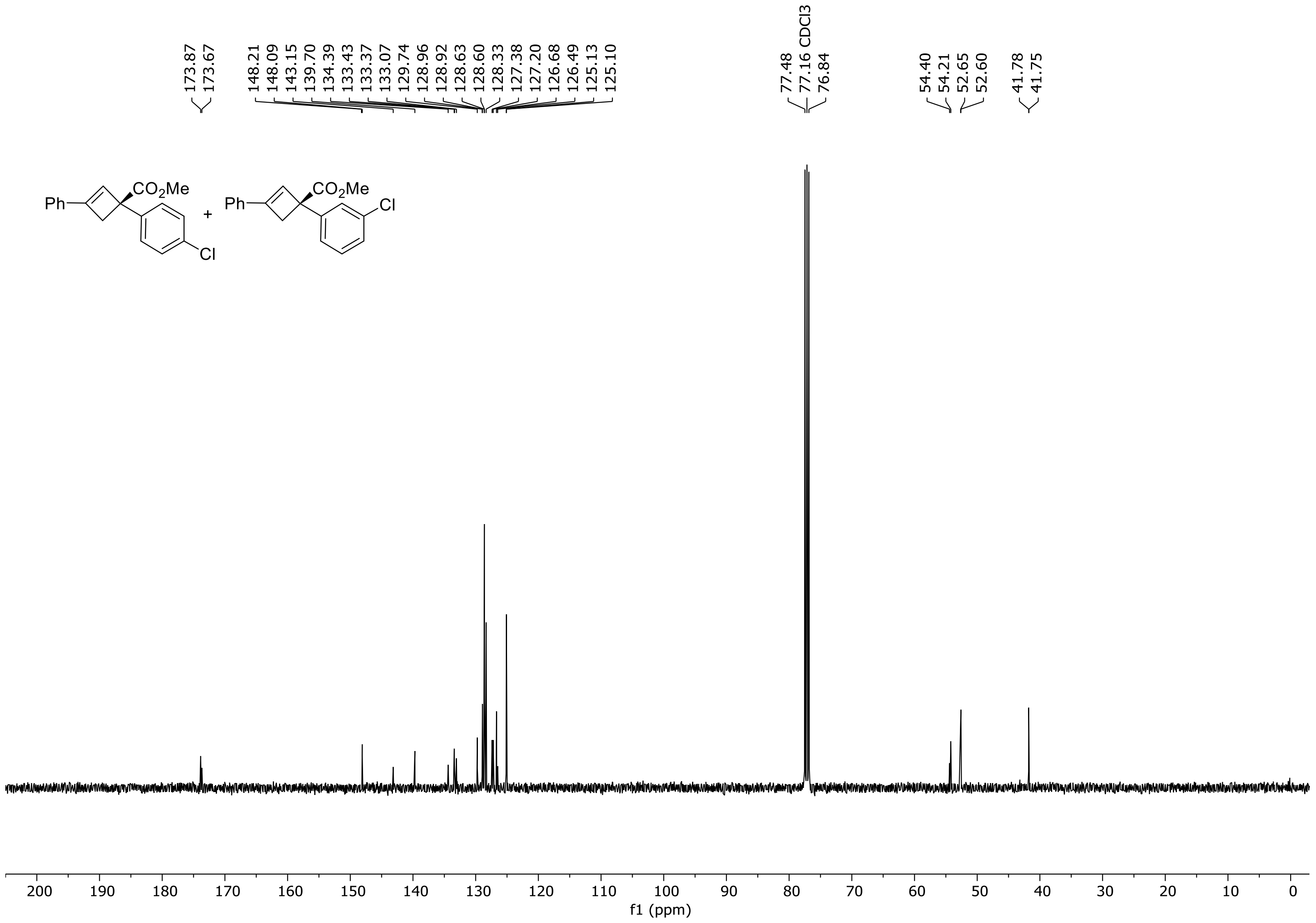

^{1}H NMR (400 MHz, $CDCl_3$, 298 K) spectrum of **5aa**.

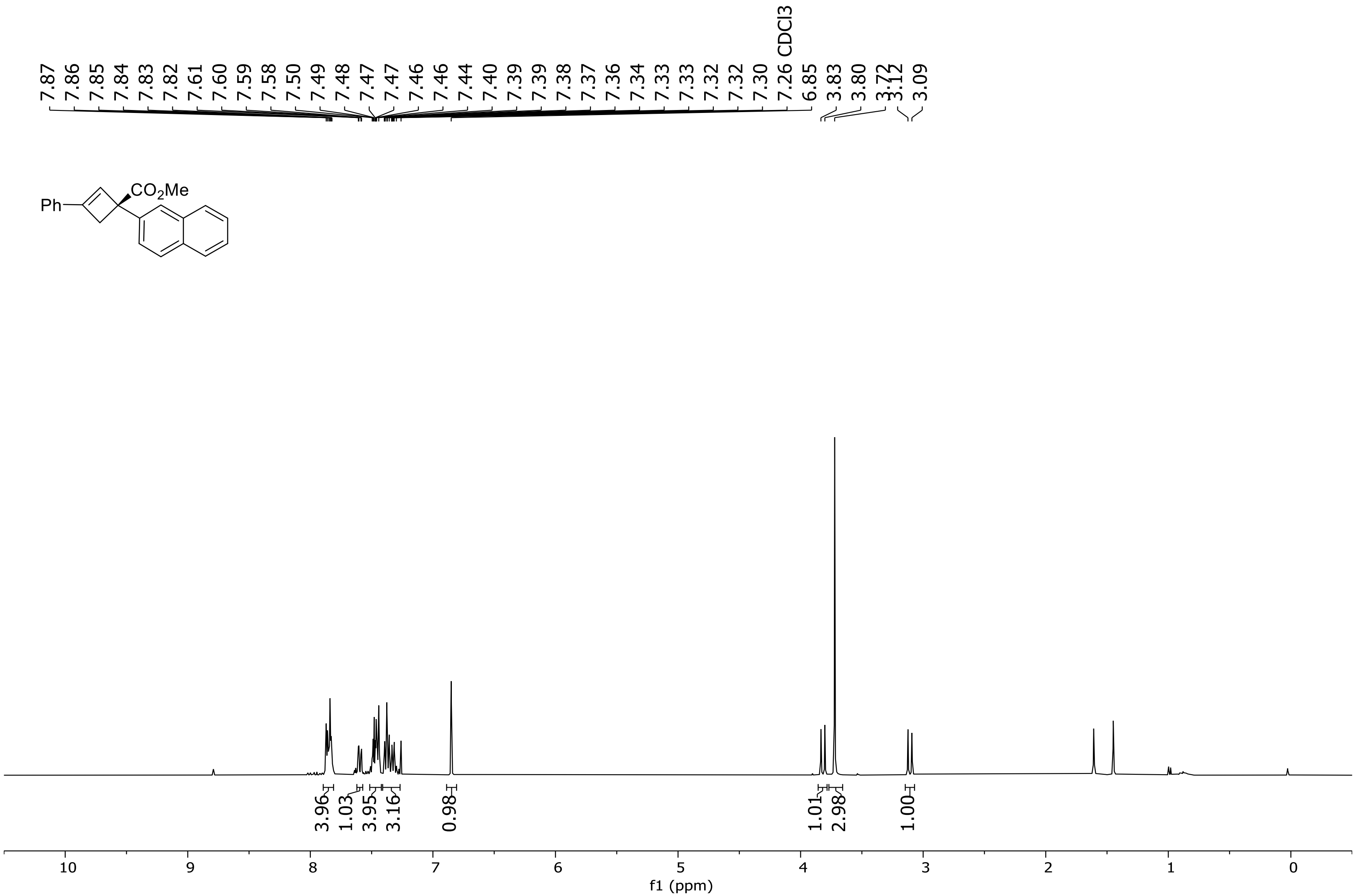

^{13}C NMR (100 MHz, $CDCl_3$, 298 K) spectrum of **5aa**.

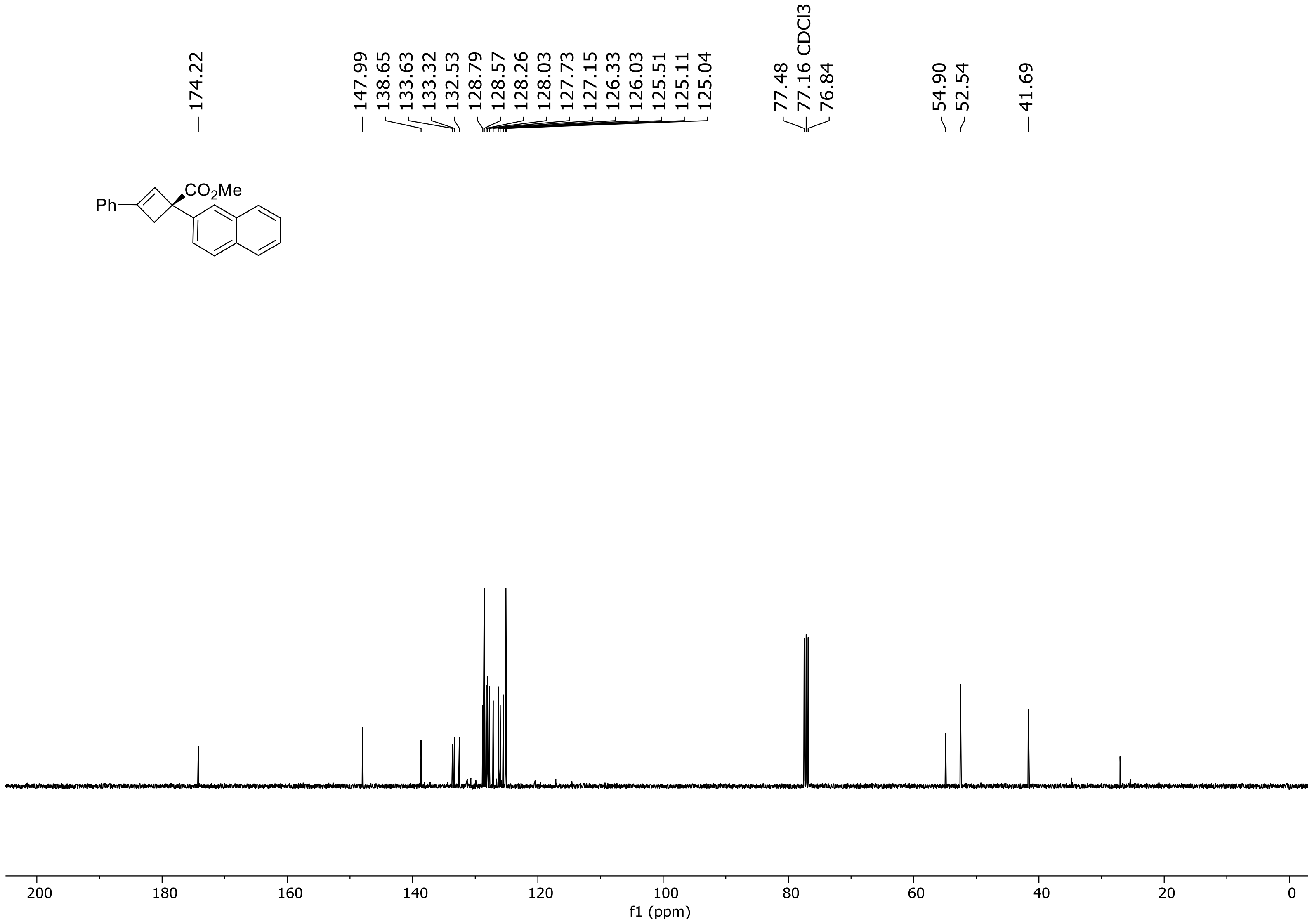

^{1}H NMR (400 MHz, $CDCl_3$, 298 K) spectrum of **5ab**.

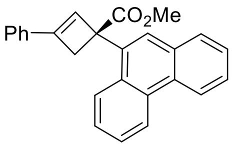

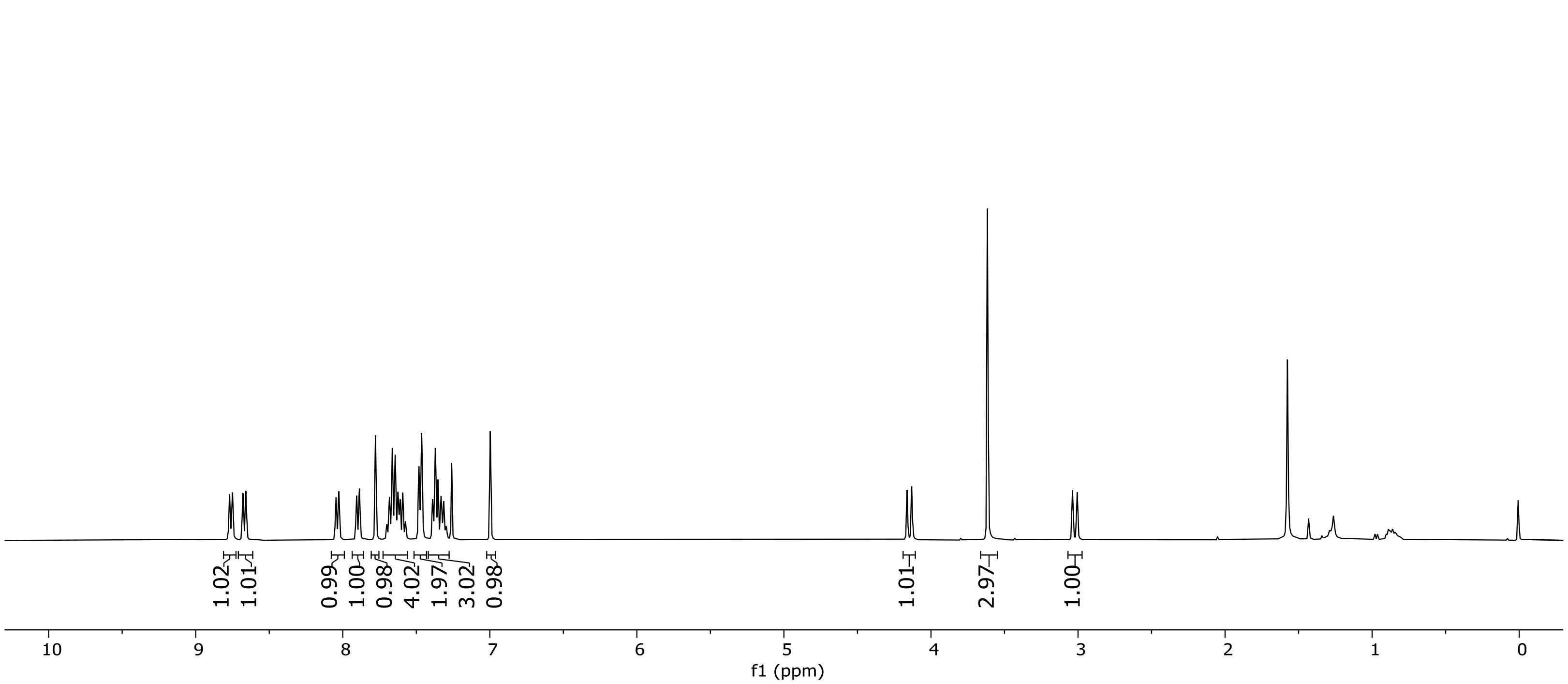

^{13}C NMR (100 MHz, $CDCl_3$, 298 K) spectrum of **5ab**.

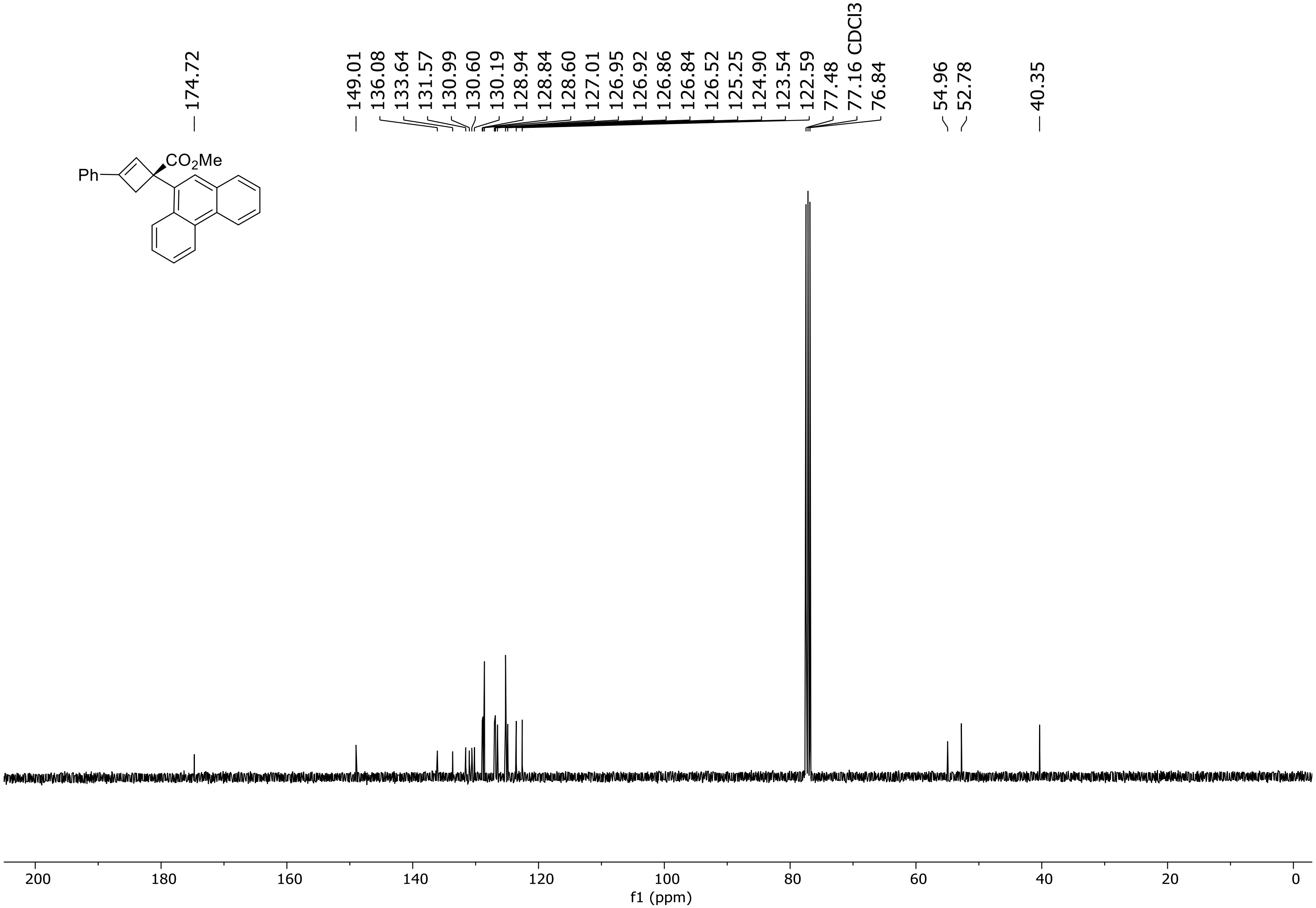

10 Crystallographic Data

10.1 Single crystal X-ray diffraction experimental

Low temperature single crystal X-ray diffraction data for **3i** (recrystallized from $CDCl_3$/pentane at 25 °C) and **3n** (recrystallized from $CDCl_3$/pentane at 25 °C) were collected using a Rigaku Oxford Diffraction SuperNova diffractometer at 150 K. **3ab** (recrystallized from diethyl ether/pentane at –40 °C) and **7** (recrystallized from diethyl ether/pentane at 0 °C) were collected using a Rigaku Synergy-DW diffractometer (EP/V028995/1) at 100 K. The single crystal was affixed to a cryoloop using Fomblin® Y oil. Raw frame data were reduced using CrysAlisPro and the structure was solved using 'Superflip' [8] before refinement with CRYSTALS. [9] Refinement was by full-matrix least-squares with anisotropic atomic displacement parameters freely refined for all non-hydrogen atoms. In general, hydrogen atoms were visible in the difference Fourier map. Hydrogens bound to carbon were positioned at calculated positions and refined separately with restraints before being included in the refinement using a riding model. [10]

Single crystal of **5l** and **5n** (recrystallized from EtOAc/n-hexane at 25 °C) was mounted and the diffraction data was collected at 173 K on a Bruker D8 Quest diffractometer using SMART/SAINT software. Intensity data were collected using MoKα radiation (λ=0.71073 A°). The single crystal was affixed to a Hampton Research cryoloop using Paratone-N oil. Data collection and reduction was performed using Bruker APEX4 and Bruker SAINT, respectively. The structure was solved by direct methods using the SHELX-97 and refined by full-matrix leasts-quares on F2. Empirical absorption corrections were applied with SADABS. All Nonhydrogen atoms were refined anisotropically and hydrogen atoms were included in geometric positions. Structure was drawn using Olex-2[11] and Mercury-3.

Final refinement statistics are given in the table below and further details, are documented in the CIF; Crystallographic data have been deposited with the Cambridge Crystallographic Data Centre CCDC 2280531 (**3i**), 2280532 (**3n**), 2307983 (**3ab**) 2266042 (**5l**), 2266045 (**5n**) and 2307984 (**7**) can be obtained via www.ccdc.cam.ac.uk/data_request/cif.

10.2 Solid-state structures

Solid-state structure of compound **3i**, Thermal ellipsoids drawn at 50% probability. Carbon: black; oxygen: red; bromine dark red.

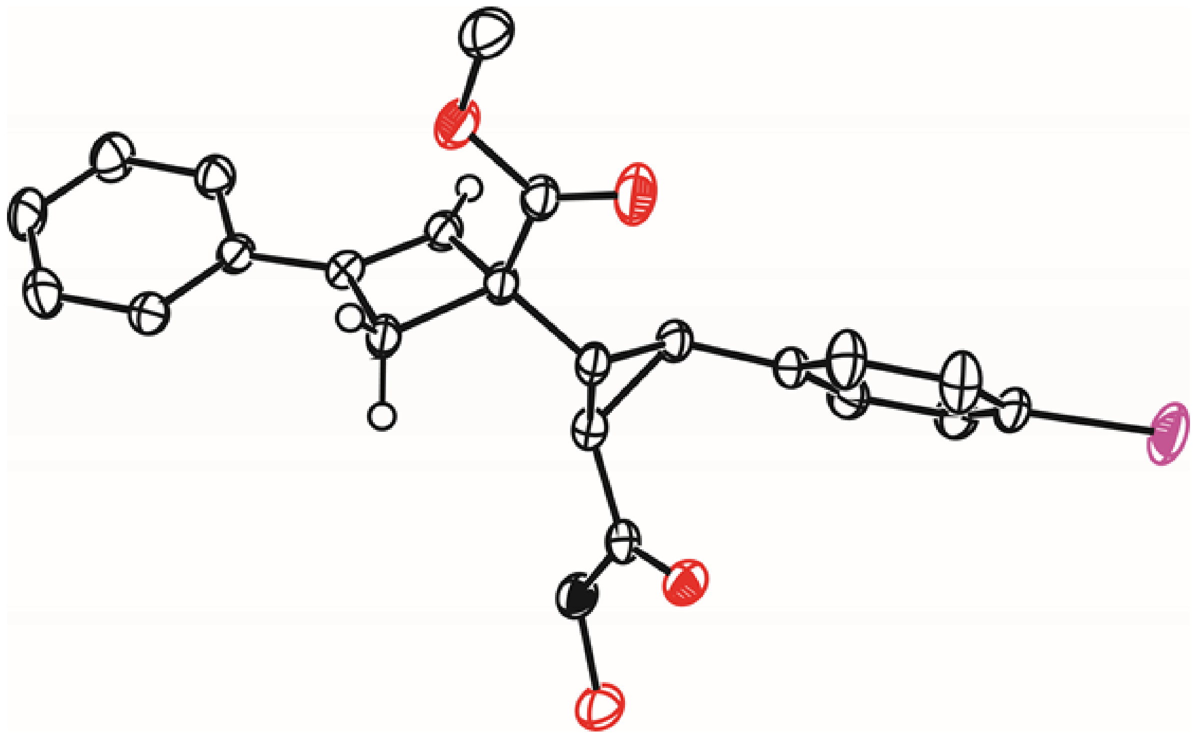

Solid-state structure of compound **3n**, Thermal ellipsoids drawn at 50% probability. Carbon: black; oxygen: red; nitrogen: blue.

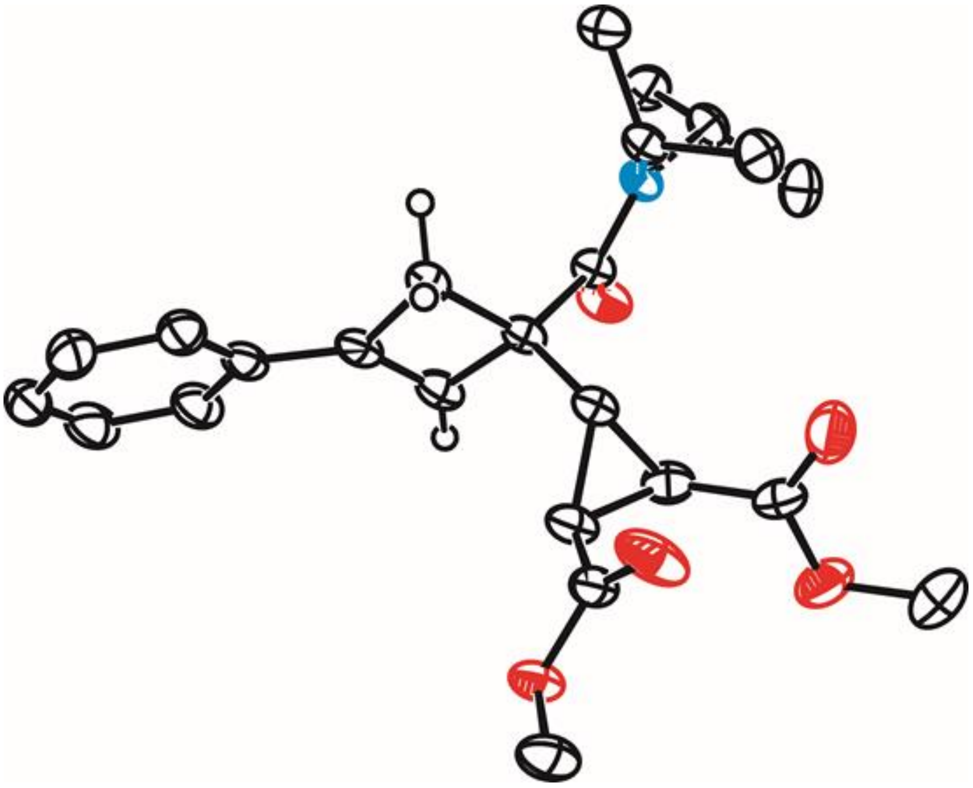

Solid-state structure of compound **3ab**, Thermal ellipsoids drawn at 50% probability. Carbon: black; oxygen: red; fluorine green.

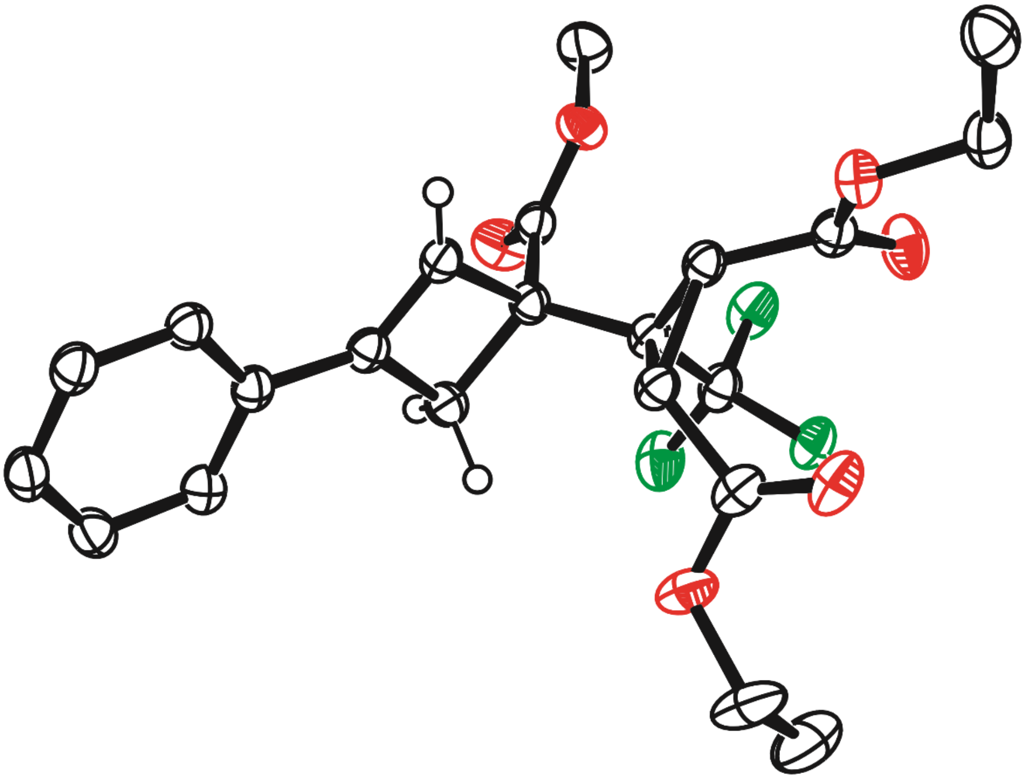

Solid-state structure of compound **5m**, Thermal ellipsoids drawn at 50% probability. Carbon: black; oxygen: red..

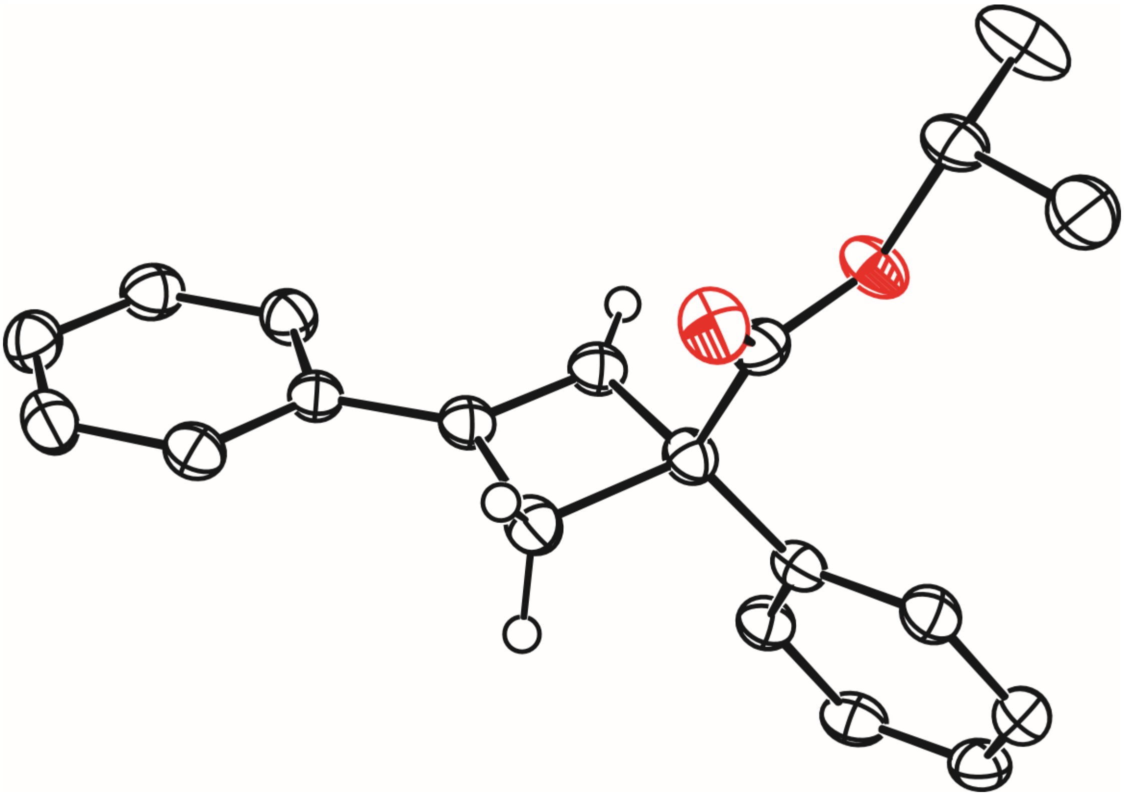

Solid-state structure of compound **5o**, Thermal ellipsoids drawn at 50% probability. Carbon: black; oxygen: red; nitrogen: blue.

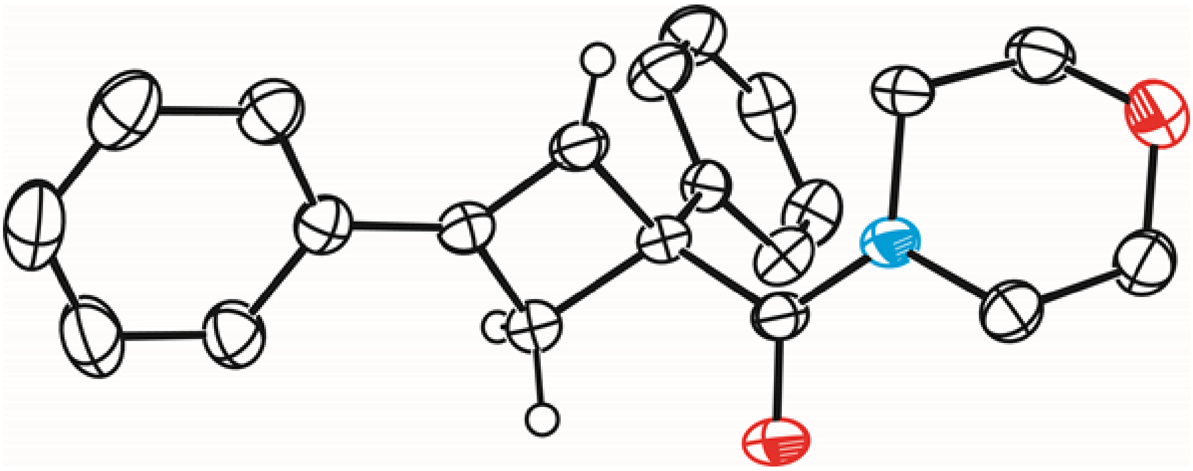

Solid-state structure of compound **7**, Thermal ellipsoids drawn at 50% probability. Carbon: black; oxygen: red.

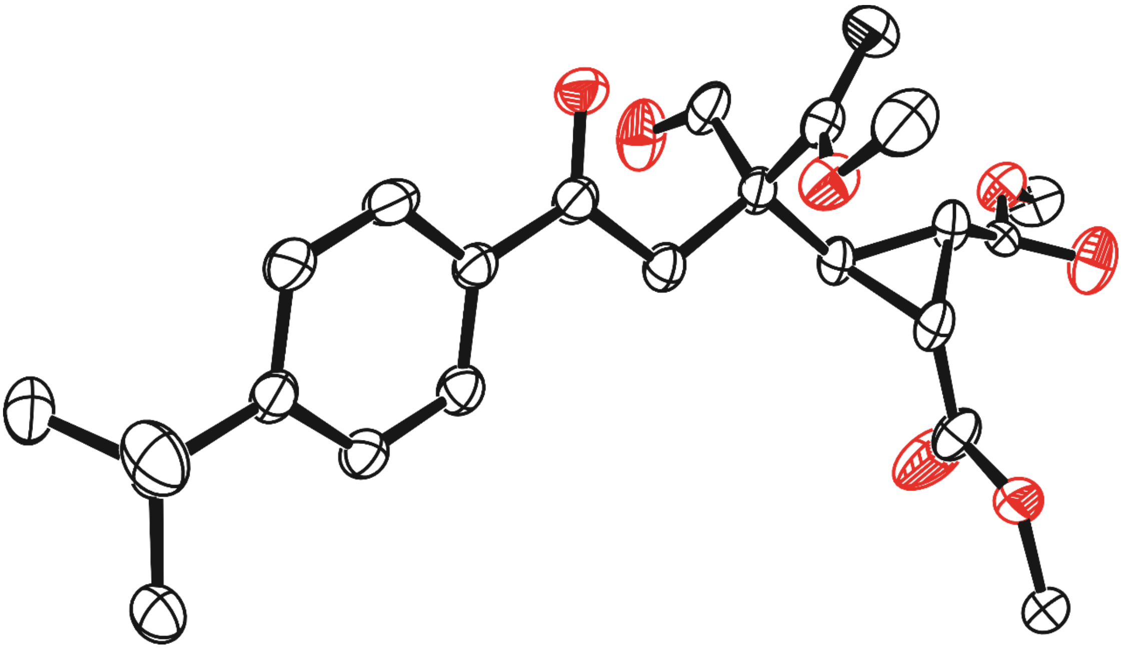

10.3 X-ray refinement data

Crystal data and structure refinement for compound **3i**. [CCDC: 2280531]

Identification code	7719	
Empirical formula	C23H21BrO4	
Formula weight	441.32	
Temperature	150 K	
Wavelength	1.54184 Å	
Crystal system	Triclinic	
Space group	P -1	
Unit cell dimensions	a = 9.6522(4) Å	a= 75.533(3)°.
	b = 10.3769(4) Å	b= 80.034(3)°.
	c = 10.9237(3) Å	g = 73.104(3)°.
Volume	1007.72(7) $Å^3$	
Z	2	
Density (calculated)	1.454 Mg/m^3	
Absorption coefficient	2.996 mm^{-1}	
F(000)	452	
Crystal size	0.250 x 0.200 x 0.180 mm^3	
Theta range for data collection	4.204 to 76.133°.	
Index ranges	-8<=h<=12, -11<=k<=12, -10<=l<=13	
Reflections collected	9832	
Independent reflections	4121 [R(int) = 0.022]	
Completeness to theta = 73.088°	99.6 %	
Absorption correction	Semi-empirical from equivalents	
Max. and min. transmission	0.58 and 0.44	
Refinement method	Full-matrix least-squares on F^2	
Data / restraints / parameters	4121 / 0 / 254	
Goodness-of-fit on F^2	1.0053	
Final R indices [I>2sigma(I)]	R1 = 0.0308, wR2 = 0.0820	
R indices (all data)	R1 = 0.0317, wR2 = 0.0829	
Extinction coefficient	19(2)	
Largest diff. peak and hole	0.59 and -0.91 e.$Å^{-3}$	

Crystal data and structure refinement for compound **3n**. [CCDC: 2280532]

Identification code	7715	
Empirical formula	C24H31NO5	
Formula weight	413.51	
Temperature	150 K	
Wavelength	1.54184 Å	
Crystal system	Monoclinic	
Space group	P 2_1/c	
Unit cell dimensions	a = 7.2538(2) Å	a= 90°.
	b = 10.6413(2) Å	b= 92.839(2)°.
	c = 29.3223(7) Å	g = 90°.
Volume	2260.61(9) $Å^3$	
Z	4	
Density (calculated)	1.215 Mg/m^3	
Absorption coefficient	0.685 mm^{-1}	
F(000)	888.000	
Crystal size	0.25 x 0.21 x 0.09 mm^3	
Theta range for data collection	4.421 to 76.392°.	
Index ranges	-9<=h<=8, -12<=k<=13, -36<=l<=36	
Reflections collected	17467	
Independent reflections	4698 [R(int) = 0.034]	
Completeness to theta = 74.865°	99.7 %	
Absorption correction	Semi-empirical from equivalents	
Max. and min. transmission	0.94 and 0.80	
Refinement method	Full-matrix least-squares on F^2	
Data / restraints / parameters	4698 / 16 / 304	
Goodness-of-fit on F^2	0.9999	
Final R indices [I>2sigma(I)]	R1 = 0.0390, wR2 = 0.0989	
R indices (all data)	R1 = 0.0440, wR2 = 0.1047	
Extinction coefficient	38(7)	
Largest diff. peak and hole	0.27 and -0.26 e.$Å^{-3}$	

Crystal data and structure refinement for compound **3ab**. [CCDC: 2307983]

Identification code	7804	
Empirical formula	C22H23F3O6	
Formula weight	440.41	
Temperature	100 K	
Wavelength	1.54184 Å	
Crystal system	Monoclinic	
Space group	P 2_1/c	
Unit cell dimensions	a = 11.20910(10) Å	a= 90°.
	b = 12.58740(10) Å	b= 98.7239(7)°.
	c = 15.39480(10) Å	g = 90°.
Volume	2146.98(3) Å^3	
Z	4	
Density (calculated)	1.362 Mg/m^3	
Absorption coefficient	0.989 mm^{-1}	
F(000)	920	
Crystal size	0.10 x 0.09 x 0.03 mm^3	
Theta range for data collection	3.990 to 74.740°.	
Index ranges	-14<=h<=13, -15<=k<=14, -19<=l<=19	
Reflections collected	41496	
Independent reflections	4374 [R(int) = 0.020]	
Completeness to theta = 74.740°	99.5 %	
Absorption correction	Semi-empirical from equivalents	
Max. and min. transmission	0.97 and 0.90	
Refinement method	Full-matrix least-squares on F^2	
Data / restraints / parameters	4374 / 0 / 281	
Goodness-of-fit on F^2	0.9993	
Final R indices [I>2sigma(I)]	R1 = 0.0359, wR2 = 0.0937	
R indices (all data)	R1 = 0.0389, wR2 = 0.0959	
Extinction coefficient	14(3)	
Largest diff. peak and hole	0.54 and -0.28 e.Å^{-3}	

Crystal data and structure refinement for compound **5m**. [CCDC: 2266042]

Identification code	2266042
Empirical formula	$C_{20}H_{20}O_2$
Formula weight	292.36
Temperature/K	173(2)
Crystal system	monoclinic
Space group	$P2_1/n$
a/Å	9.8999(8)
b/Å	13.6322(10)
c/Å	11.7650(10)
α/°	90
β/°	97.057(3)
γ/°	90
Volume/Å^3	1575.7(2)
Z	4
ρ_{calc}g/cm^3	1.232
μ/mm^{-1}	0.078
F(000)	624.0
Crystal size/mm^3	0.316 × 0.171 × 0.115
Radiation	MoKα (λ = 0.71073)
2Θ range for data collection/°	5.894 to 55.224
Index ranges	-12 ≤ h ≤ 12, -17 ≤ k ≤ 17, -15 ≤ l ≤ 15
Reflections collected	45942
Independent reflections	3655 [R_{int} = 0.0679, R_{sigma} = 0.0289]
Data/restraints/parameters	3655/0/199
Goodness-of-fit on F^2	1.019
Final R indexes [I>=2σ (I)]	R_1 = 0.0438, wR_2 = 0.1024
Final R indexes [all data]	R_1 = 0.0567, wR_2 = 0.1088
Largest diff. peak/hole / e Å^{-3}	0.21/-0.17

Crystal data and structure refinement for compound **5o**. [CCDC: 2266045]

Identification code	2266045
Empirical formula	$C_{21}H_{21}NO_2$
Formula weight	319.39
Temperature/K	173(2)
Crystal system	triclinic
Space group	P-1
a/Å	6.0108(3)
b/Å	11.6565(6)
c/Å	12.5461(7)
α/°	92.131(2)
β/°	93.294(2)
γ/°	100.898(2)
Volume/Å^3	860.73(8)
Z	2
ρ_{calc}g/cm^3	1.232
μ/mm^{-1}	0.079
F(000)	340.0
Crystal size/mm^3	0.353 × 0.216 × 0.214
Radiation	MoKα (λ = 0.71073)
2Θ range for data collection/°	6.514 to 60.732
Index ranges	-8 ≤ h ≤ 8, -16 ≤ k ≤ 16, -17 ≤ l ≤ 17
Reflections collected	29651
Independent reflections	5168 [R_{int} = 0.0429, R_{sigma} = 0.0313]
Data/restraints/parameters	5168/0/217
Goodness-of-fit on F^2	1.027
Final R indexes [I>=2σ (I)]	R_1 = 0.0488, wR_2 = 0.1203
Final R indexes [all data]	R_1 = 0.0665, wR_2 = 0.1301
Largest diff. peak/hole / e Å^{-3}	0.27/-0.17

Crystal data and structure refinement for compound **7**. [CCDC: 2307984]

Identification code	7808	
Empirical formula	C23 H28 O8	
Formula weight	432.48	
Temperature	100 K	
Wavelength	1.54184 Å	
Crystal system	Triclinic	
Space group	P -1	
Unit cell dimensions	a = 9.7832(3) Å	a= 76.376(2)°.
	b = 14.7188(4) Å	b= 81.478(2)°.
	c = 16.6186(4) Å	g = 72.878(3)°.
Volume	2214.60(11) $Å^3$	
Z	4	
Density (calculated)	1.297 Mg/m^3	
Absorption coefficient	0.816 mm^{-1}	
F(000)	920.017	
Crystal size	0.10 x 0.06 x 0.05 mm^3	
Theta range for data collection	2.746 to 74.632°.	
Index ranges	-11<=h<=12, -18<=k<=18, -19<=l<=20	
Reflections collected	49226	
Independent reflections	9013 [R(int) = 0.040]	
Completeness to theta = 73.140°	99.7 %	
Absorption correction	Semi-empirical from equivalents	
Max. and min. transmission	0.96 and 0.76	
Refinement method	Full-matrix least-squares on F^2	
Data / restraints / parameters	9004 / 440 / 657	
Goodness-of-fit on F^2	1.0374	
Final R indices [I>2sigma(I)]	R1 = 0.0866, wR2 = 0.0559	
R indices (all data)	R1 = 0.0985, wR2 = 0.0583	
Largest diff. peak and hole	0.60 and -0.41 e.$Å^{-3}$	

11 Computational Data

11.1 Computational details

All DFT calculations were carried out using Gaussian 16 program[12] and ORCA (v 5.0.3)[13]. Geometry optimization of all structures was carried out using the Gaussian 16 program at the IEFPCM(acetonitrile)-B2PLYP(D3)/def2-SVP level of theory at 298 K without any constraints of freedom. [14,15,16] Each stationary point was confirmed to be either a local minimum or a transition state by vibrational frequency calculations performed at the same level of theory. Natural Bond Orbital (NBO) analysis was performed using the NBO (version 3)32 analysis module implemented in Gaussian 16 program package. Single point electronic energies for each optimized structure at 298 K were obtained using Domain-Based Local Pair Natural Orbital coupled cluster with singles, doublets and perturbative triplets (DLPNO-CCSD(T)[17] method implemented in ORCA (v 5.0.3) program and def2-TZVPP[7] basis set[15] using 'Tight' convergence criteria. For the DLPNO-CCSD(T) calculations, the CPCM method[18] was used to simulate the solvent effects with actonitrile as solvent. Several corrections to the Gibbs free energies of each species were applied using the GoodVibes[19] program package: i) quasi-harmonic corrections by using the keyword '-q'; ii) corrections to a solution-phase standard state of 1 M by using the keyword '-c 1'; iii) entropic symmetry corrections by using the keyword '--ssymm'. The Gibbs free energies (G_{sol}, kcal mol^{-1}) of each species were calculated using the formula below:

$$G_{sol} = E_{elec}\big(DLPNO\text{-}CCSD(T)\big) + [G_{corr}(B2PLYPD3) - E_{elec}(B2PLYPD3)]$$

where $E_{elec}\big(DLPNO\text{-}CCSD(T)\big)$ is the electronic energy calculated at the CPCM(acetonitrile)-DLPNO-CCSD(T)/def2-TZVPP//IEFPCM(acetonitrile)-B2PLYP(D3)/def2-SVP level of theory; $G_{corr}(B2PLYPD3)$ is the corrected Gibbs free energy computed by the GoodVibes program at the IEFPCM(acetonitrile)-B2PLYP(D3)/def2-SVP level of theory; $E_{elec}(B2PLYPD3)$ is the electronic energy calculated at the IEFPCM(acetonitrile)-B2PLYP(D3)/def2-SVP level of theory.

11.2 Evaluation of Level of Theory

The selection of density functional for this study was based on a screening of different functionals (including ωB97xd[20], PBE[21], TPSS[22], M06-2X[23], B2PLYP[15] with D3BJ dispersion[24,25]) combined with def2-SVP basis set.[16]

Optimized structures using those selected functionals all exhibited high geometry consistency. Some of the selected geometry attributes were shown below.

Comparison of Optimized Geometries Using Different Functionals.

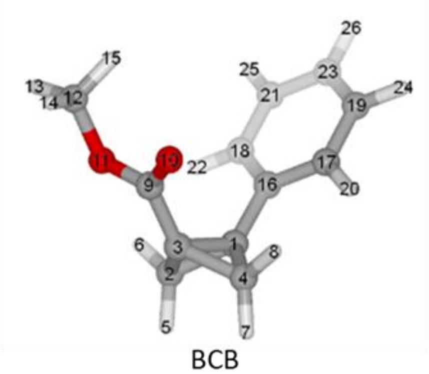

BCB

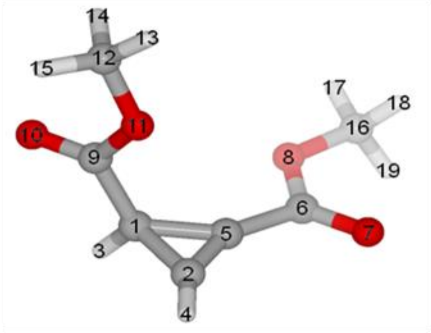

Cyclopropene

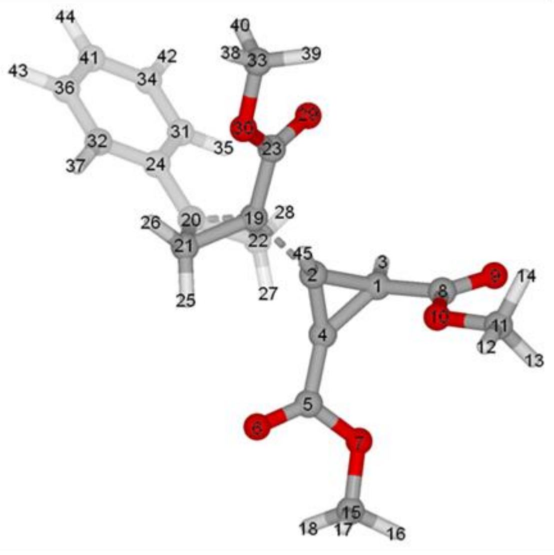

TS_{RSR}

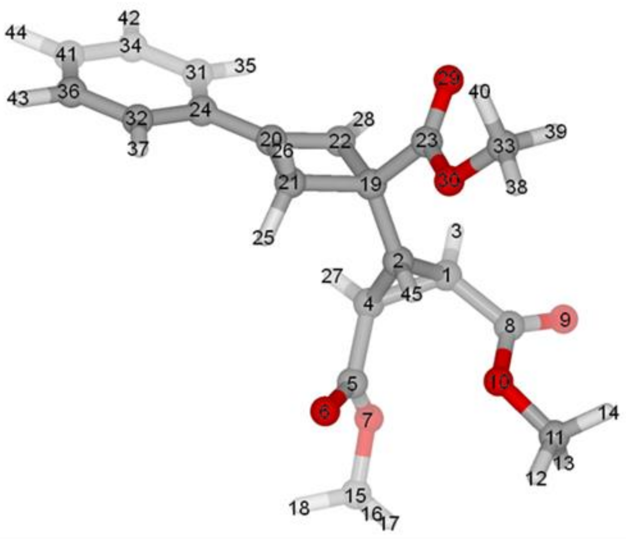

$Product_{RSR}$

	Geometry Attributes	ωB97xd	PBE	TPSS
Cyclopropene (6a)	D(9-1-5-2)/°	106.5	106.7	106.6
	B(2-5)/Å	1.29	1.31	1.30
	A(2-1-5)/°	50.8	51.1	51.0
BCB	A(9-3-1)/°	121.8	123.9	124.8
	A(3-1-16)/°	127.5	129.5	130.0
	B(3-1) /Å	1.52118	1.55	1.55
	D(4-3-1-2)/°	123.7	124.4	124.0
	D(4-1-16-17)/°	17.5	10.4	10.5
TS_{RSR}	B(2-19)/Å	1.97	2.08	2.02
	B(19-20) /Å	1.70	1.72	1.75
	D(22-19-20-21)/°	131.0	131.4	132.1
	D(4-2-19-22)/°	-25.8	-25.6	-25.5
	A(23-19-20)/°	110.8	112.2	111.0
	A(19-20-24)/°	126.9	129.3	129.7
$Product_{RSR}$	D(23-19-21-22)/°	114.5	114.0	113.9
	D(1-2-19-22)/°	46.8	47.1	47.3
	D(5-4-1-8)/°	1.0	1.4	1.3
	D(22-20-24-31)/°	0.1	0.4	0.4
	A(5-4-2)/°	119.7	119.8	119.8
	A(8-1-2)/°	121.1	122.0	122.0
	A(2-19-23)/°	110.8	111.4	111.4
	B(22-20)/Å	1.35	1.36	1.36
	B(19-2)/Å	1.52	1.52	1.53

	Geometry Attributes	M06-2X	B2PLYP(D3)
Cyclopropene 6a	D(9-1-5-2)/°	106.3	106.6
	B(2-5)/Å	1.29	1.30
	A(2-1-5)/°	50.9	51.0
BCB	A(9-3-1)/°	118.8	119.2
	A(3-1-16)/°	125.9	125.8
	B(3-1) /Å	1.51	1.55
	D(4-3-1-2)/°	124.1	125.0
	D(4-1-16-17)/°	20.0	13.9
TS_{RSR}	B(2-19)/Å	1.89	1.96
	B(19-20)/Å	1.68	1.74
	D(22-19-20-21)/°	130.7	132.0
	D(4-2-19-22)/°	-28.7	-25.9
	A(23-19-20)/°	108.7	109.3
	A(19-20-24)/°	125.2	126.9
$Product_{RSR}$	D(23-19-21-22)/°	114.4	114.8
	D(1-2-19-22)/°	44.6	46.8
	D(5-4-1-8)/°	1.4	1.0

D(22-20-24-31)/°	0.1	0.3
A(5-4-2)/°	119.0	119.6
A(8-1-2)/°	120.5	121.0
A(2-19-23)/°	111.1	110.6
B(22-20)/Å	1.35	1.36
B(19-2)/Å	1.51	1.51

All geometries were obtained at the IEFPCM(acetonitrile)-[Functional]/def2-SVP level of theory. A = bond angle; B = bond length; D = dihedral angle.

Energy analysis revealed that M06-2X and B2PLYP(D3) produced reaction energy profiles close to that of DLPNO-CCSD(T) calculations (see below). However, the use of larger basis sets (def2-TZVP and def2-TZVPP) in combination with the M06-2X functional did not improve the accuracy of its energy profile but caused an increased deviation from the DLPNO-CCSD(T) results (see below). We've also attempted basis sets screening using B2PLYP(D3) functional, however, the calculation failed to complete. We hence decided to use CPCM(acetonitrile)-DLPNO-CCSD(T)/def2-TZVPP//B2PLYP(D3)/def2-SVP level of theory as a good compromise between efficient geometry optimizations and accurate energies.

Comparison of Electronic Energies Using Different Functionals.

	ωB97xd	***PBE***	***TPSS***	***DLPNO-CCSD(T)***
SM				
Cyclopropene (Ha)	−571.784716	−571.321613	−572.065100	−571.510223
BCB (Ha)	−614.262008	−613.70845	−614.578396	−613.843955
TS				
TS_{RSR} (Ha)	−1186.033114	−1185.022443	−1186.629995	−1185.337000
$\Delta E_{elec}^{‡}$ (kcal mol^{-1})	8.5	4.8	8.8	10.8
Product				
$Product_{RSR}$ (Ha)	−1186.156204	−1185.120727	−1186.727157	−1185.457977
ΔE_{elec} (kcal mol^{-1})	−68.7	−56.9	−52.5	−65.1

	M06-2X	*B2PLYP(D3)*	*DLPNO-CCSD(T)*
S.M.			
Cyclopropene (Ha)	−571.732303	−570.878173	−571.510223
BCB (Ha)	−614.195775	−613.184339	−613.843955
TS			
TS_{RSR} (Ha)	−1185.911796	−1184.037519	−1185.337000
$\Delta E_{elec}^{‡}$ (kcal mol^{-1})	10.2	15.7	10.8
Product			
$Product_{RSR}$ (Ha)	−1186.025435	−1184.167325	−1185.457977
ΔE_{elec} (kcal mol^{-1})	−61.1	−65.8	−65.1

Electronic energies of each species were obtained at the IEFPCM(acetonitrile)-[Functional]/def2-SVP level of theory or at the CPCM(acetonitrile)-DLPNO-CCSD(T)/def2-TZVPP//IEFPCM(acetonitrile)-B2PLYP(D3)/def2-SVP. All electronic energies were reported in hartree (Ha); relative electronic energies were calculated compared to the sum of starting material electronic energies and were reported in kcal mol^{-1}.

Comparison of Electronic Energies Using M06-2X Combined with Different Basis Sets.

	def2-SVP	*def2-TZVP*	*def2-TZVPP*
SM			
Cyclopropene (Ha)	−571.732303	−572.395355	−572.39839
BCB (Ha)	−614.195775	−614.882876	−614.887543
TS			
TS_{RSR} (Ha)	−1185.911796	−1187.258943	−1187.266662
$\Delta E_{elec}^{‡}$ (kcal mol^{-1})	10.2	12.1	12.1
Product			
$Product_{RSR}$ *(Ha)*	−1186.025435	−1187.36917	−1187.376801
ΔE_{elec} (kcal mol^{-1})	−61.1	−57.1	−57.0

Electronic energies of each species were obtained at the IEFPCM(acetonitrile)-M06-2X/[Basis Set] level of theory. All electronic energies were reported in hartree (Ha); relative electronic energies were calculated compared to the sum of starting material electronic energies and were reported in kcal mol^{-1}.

11.3 Intrinsic Reaction Coordinate (IRC) Calculation

As an effort to locate any intermediate structure before the formation of the final product, we performed IRC calculation on the **TS**$_{RSR}$ structure at IEFPCM(acetonitrile)-B2PLYP(D3)/def2-SVP level of theory using the IRC program package built in the Gaussian 16 program. The plot of system total energies against IRC showed no minima between **TS**$_{\mathbf{RSR}}$ and the product, which suggested that the mechanism is highly concerted. Various molecule properties were also plotted against IRC, showing asynchronicity of the C–C bond formation and H atom shift. Therefore, we concluded that the reaction proceeds through a concerted asynchronous mechanism.

DFT-computed reaction pathways for the reaction of CPCM(acetonitrile)-DLPNO-CCSD(T)/def2-TZVPP//IEFPCM(acetonitrile)-B2PLYP(D3)/def2-SVP level of theory at 298K in acetonitrile

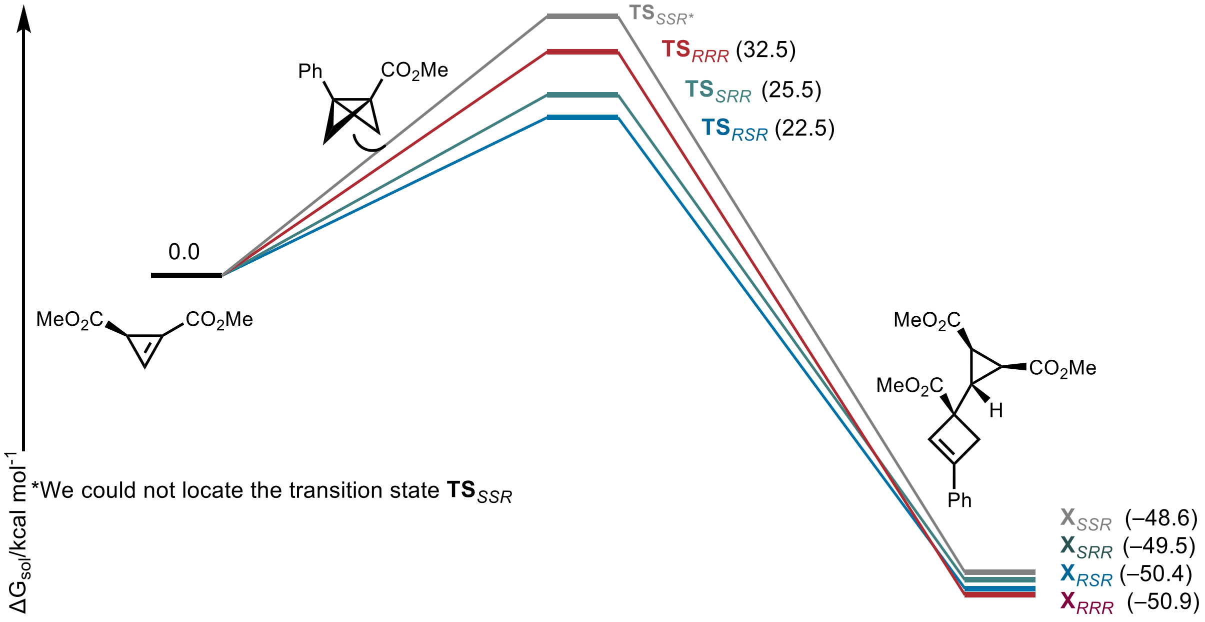

Plot of total energies against reaction coordinate for TS$_{RSR}$.

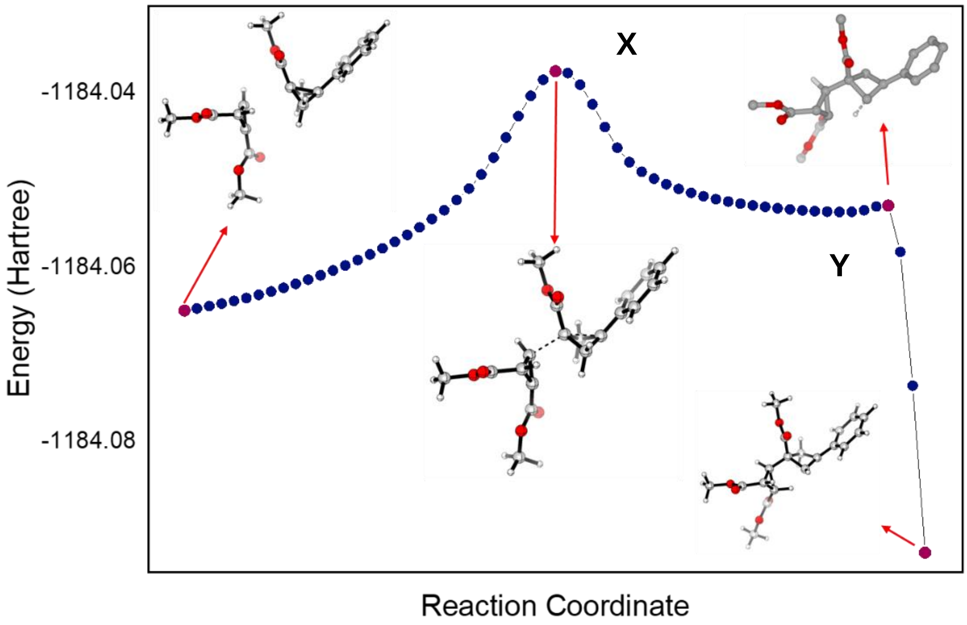

Wiberg Bond Order at stage **X**:
C1–C3 = 0.460
C1–C4= 0.4181
C2–C3 = 1.049
C4–C5 = 1.457
C2–H2 = 0.870
C5–H2 = 0.023

Wiberg Bond Order at stage **Y**:
C1–C3 = 0.104
C1–C4 = 0.929
C2–C3 = 1.323
C4–C5 = 1.023
C2–H2 = 0.511
C5–H2 = 0.322

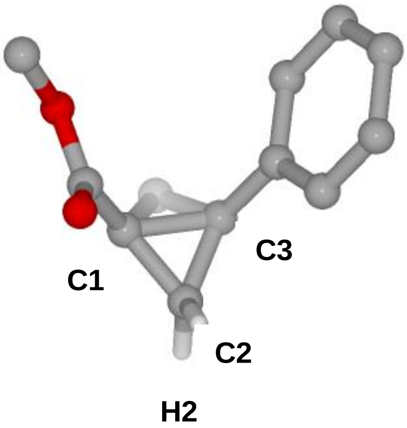

Wiberg Bond Order of BCB (**1a**):

C1–C3 = 0.7548

C2–C3 = 0.9937

C2–H2 = 0.9140

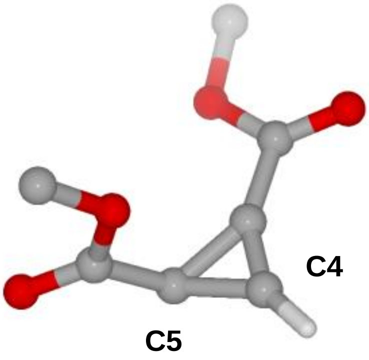

Wiberg Bond Order of cyclopropene (**6a**):

C4–C5 = 1.8772

Plot of different bond lengths against reaction coordinate.

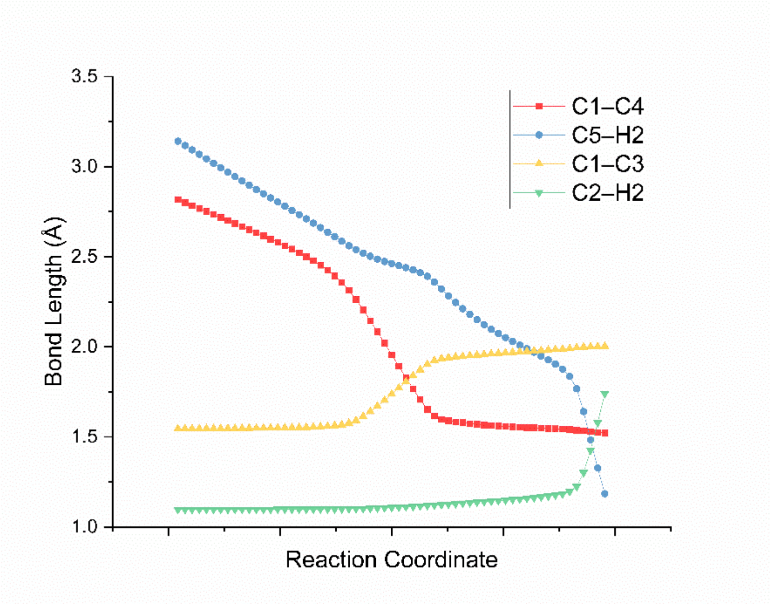

11.4 Cartesian coordinates and total energies for the calculated structures

BCB-1a (IEFPCM(acetonitrile)-ωb97XD/def2-SVP)

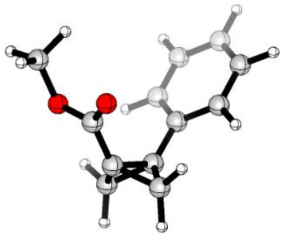

C 0.10644600 -1.44777300 0.50136100
C 1.12770800 -1.36472500 1.58957800
C 1.55214300 -1.10296200 0.17722800
C 0.88088100 -2.26678700 -0.48078900
H 1.47308300 -2.29296300 2.06198300
H 1.08317400 -0.50129300 2.25839500
H 1.20386200 -3.27621300 -0.19729100
H 0.62884400 -2.14498000 -1.53848900
C 1.91807500 0.16234400 -0.46780400
O 1.98248200 0.32649300 -1.66474900
O 2.12637000 1.13230600 0.42487300
C 2.40303000 2.42730300 -0.09212300
H 2.55371200 3.07738100 0.77592700
H 3.30851500 2.41322900 -0.71452300
H 1.56006500 2.79350700 -0.69491400
C -1.08795900 -0.61442000 0.27003900
C -1.86001400 -0.78653600 -0.88786600
C -1.46167200 0.38080600 1.18606000
C -2.96414200 0.02819700 -1.13314300
H -1.60090000 -1.57155700 -1.60098800
C -2.56449400 1.19465700 0.93779800
H -0.88900800 0.52059500 2.10477300
C -3.31902600 1.02429300 -0.22386100
H -3.55342900 -0.12097800 -2.04056300
H -2.83902100 1.96576200 1.66095800
H -4.18557700 1.66073000 -0.41512500

Imaginary Frequencies: 0
Electronic energy: -614.262008 hartree

Cyclopropene (6) (IEFPCM(acetonitrile)-ωb97XD/def2-SVP)

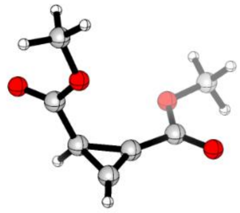

C 0.72965500 1.09022200 -0.79198100
C 0.24305700 2.03490000 0.28383200
H 0.74096700 1.40891600 -1.84227400
H 0.50492500 2.91630600 0.86110400
C -0.55809900 1.06720000 -0.01911600
C -1.81985900 0.34642600 0.20417100
O -2.68201500 0.71596500 0.96131800
O -1.87410600 -0.75790500 -0.52824100
C 1.80956600 0.09239200 -0.50530000
O 2.59404000 -0.30420400 -1.33314600
O 1.80725800 -0.31563000 0.76334200
C 2.79092200 -1.27232500 1.13461100
H 2.62789800 -1.48379800 2.19646000
H 2.67894700 -2.19299200 0.54506600
H 3.80196100 -0.86981400 0.98116900
C -3.04634600 -1.55673500 -0.39558400
H -2.90924700 -2.41134500 -1.06524200
H -3.16264800 -1.90123800 0.64119700
H -3.93759500 -0.98432500 -0.68746100

Imaginary Frequencies: 0
Electronic energy: -571.784716 hartree

TS$_{RSR}$ (IEFPCM(acetonitrile)-ωb97XD/def2-SVP)

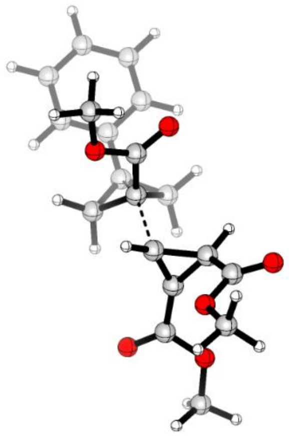

C -2.00965700 0.50588200 0.79754400
C -1.27851200 0.26628700 -0.50947300

H -1.42439300 0.53287900 1.72821100
C -2.04815500 -0.72649900 -0.03601800
C -2.87607100 -1.83341800 -0.42810600
O -2.66859900 -2.56194500 -1.38005600
O -3.92534000 -1.99168900 0.39521400
C -3.17262100 1.44237000 0.85818400
O -3.48034300 2.07741300 1.83939100
O -3.84631500 1.50071600 -0.29221900
C -4.97845300 2.35735900 -0.33232600
H -5.38911500 2.27680900 -1.34426500
H -5.73052900 2.04247000 0.40479500
H -4.68935000 3.39729100 -0.12470700
C -4.81348000 -3.05548500 0.09436800
H -5.59109600 -3.03422400 0.86572700
H -5.26869300 -2.92247200 -0.89777600
H -4.29183100 -4.02336300 0.11580300
C 0.64602100 0.01611400 -0.17973500
C 1.83883900 -1.16941200 0.07883800
C 1.05821700 -1.08576100 -1.16488700
C 0.78544900 -0.87016000 1.04918500
C 1.24396800 1.37403200 -0.18251800
C 3.27839600 -0.94977000 0.22855900
H 0.24364700 -1.81149100 -1.30525300
H 1.56984600 -0.80040400 -2.08764900
H -0.08844000 -1.55243400 1.09678500
H 1.06197400 -0.41837600 2.00494000
O 1.46380500 2.02030400 0.81389300
O 1.47232300 1.80157900 -1.42059900
C 3.83199900 -0.65403500 1.48564100
C 4.12694300 -1.03114800 -0.88838500
C 2.03589000 3.10226900 -1.55801900
C 5.19871400 -0.43104600 1.61712200
H 3.19311800 -0.60260400 2.36839200
C 5.49322900 -0.80641200 -0.75220900
H 3.71867000 -1.28180200 -1.86880900
H 2.13979700 3.27726800 -2.63336900
H 1.37489100 3.85793000 -1.11226900
H 3.01894300 3.14832900 -1.06974800
C 6.03144900 -0.50259800 0.49913500
H 5.61750400 -0.20147200 2.59869300
H 6.14264800 -0.87288800 -1.62708900
H 7.10409200 -0.32774800 0.60475900
H -1.18894000 0.85388000 -1.42357300

Imaginary Frequencies: 1
Electronic energy: -1186.033114 hartree

3a$_{RSR}$ (IEFPCM(acetonitrile)-ωb97XD/def2-SVP)

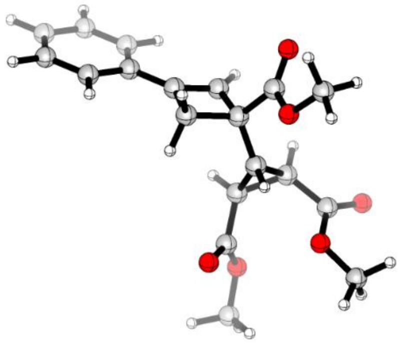

C -1.73806200 0.01361500 1.09270500
C -0.95322600 0.38031200 -0.15097500
H -1.28799700 0.22857300 2.06363500
C -1.04161100 -1.04260100 0.25301100
C -1.81554100 -2.00978900 -0.59505600
O -1.82534500 -2.01455700 -1.80017500
O -2.45999900 -2.89785900 0.15585700
C -3.22200900 0.11506500 1.12148100
O -3.86362500 0.37523400 2.10977400
O -3.77154300 -0.12413300 -0.06993000
C -5.19104500 -0.11345600 -0.14947400
H -5.43865300 -0.33130100 -1.19327200
H -5.61916900 -0.88204000 0.50880300
H -5.58788800 0.86987900 0.13717400
C -3.23539900 -3.87444200 -0.52881000
H -3.67406500 -4.51430300 0.24358300
H -4.02985800 -3.39122600 -1.11476000
H -2.60490000 -4.47136800 -1.20213200
C 0.34833400 1.14927000 -0.04256300
C 2.26331800 0.29623200 0.06412900
C 1.41584600 0.71963700 -1.11677000
C 1.35869600 0.67996200 0.98345200
C 0.08954500 2.64506800 -0.02154500
C 3.59614400 -0.30603900 0.10758800
H 1.06984100 -0.08833100 -1.78041500
H 1.82251500 1.54056800 -1.72748400
H -0.16975200 -1.49817700 0.73457400
H 1.33443300 0.67395700 2.07460500
O 0.56593500 3.41756200 0.77291600
O -0.72361300 3.01615400 -1.00815400
C 4.21222400 -0.61817700 1.33047400
C 4.27856100 -0.58242100 -1.08540000
C -1.03383800 4.40115400 -1.10557900
C 5.47956700 -1.19173500 1.35573300

H 3.69150500 -0.40840300 2.26783600
C 5.54850000 -1.15754600 -1.05904700
H 3.80929400 -0.34453100 -2.04319700
H -1.71442100 4.50326700 -1.95695100
H -1.52188800 4.75411400 -0.18662900
H -0.12261100 4.99078400 -1.27723100
C 6.15154200 -1.46316900 0.16066900
H 5.94884400 -1.42976000 2.31276800
H 6.06917900 -1.36774700 -1.99572300
H 7.14630200 -1.91348800 0.18260100
H -1.56447600 0.65469200 -1.01422000

Imaginary Frequencies: 0
Electronic energy: -1186.156204 hartree

BCB-1a (IEFPCM(acetonitrile)-PBE/def2-SVP)

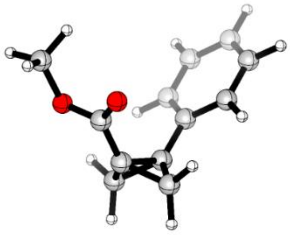

C 0.01334700 -1.42014500 0.45546300
C 1.01098500 -1.38502300 1.58069000
C 1.51039600 -1.12204200 0.18613800
C 0.80568700 -2.24568200 -0.51968300
H 1.30226000 -2.34118500 2.05678700
H 0.97550500 -0.52244700 2.26568700
H 1.07812100 -3.28076200 -0.23740600
H 0.60315300 -2.09689100 -1.59320100
C 2.00084700 0.11825200 -0.43281000
O 2.09428800 0.30570300 -1.63968600
O 2.31910700 1.04854500 0.50273800
C 2.75912700 2.31619500 -0.00623700
H 2.99080400 2.93722700 0.87679800
H 3.66228800 2.19597300 -0.63745500
H 1.96592200 2.79681900 -0.61353900
C -1.16705600 -0.57118200 0.22257700
C -1.85366400 -0.60736200 -1.01492000
C -1.64781900 0.29546400 1.23359300
C -2.96628700 0.21566300 -1.24108500
H -1.51775200 -1.29503200 -1.80521900
C -2.76090000 1.11670200 1.00339300
H -1.14963100 0.31903400 2.21426200
C -3.42366700 1.08439600 -0.23552100
H -3.48518700 0.17251200 -2.21111200
H -3.11812800 1.78370400 1.80313000
H -4.30049300 1.72565900 -0.41273900

Imaginary Frequencies: 0

Electronic energy: -613.708450 hartree

Cyclopropene (6) (IEFPCM(acetonitrile)-PBE/def2-SVP)

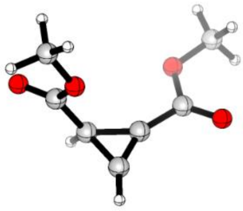

C 0.72944400 1.04784400 -0.82903700
C 0.24770400 2.03601600 0.21707000
H 0.72721600 1.32804200 -1.89995000
H 0.51496400 2.95017200 0.75717300
C -0.56606800 1.04473600 -0.04067300
C -1.83512300 0.34772600 0.21506800
O -2.68784000 0.73356000 0.99915000
O -1.92406600 -0.77146400 -0.53275000
C 1.82704200 0.06813800 -0.52231100
O 2.60513200 -0.37215100 -1.35403000
O 1.85436700 -0.27421700 0.78783800
C 2.87184100 -1.21167600 1.17048500
H 2.74831000 -1.37321500 2.25569000
H 2.75097400 -2.16873100 0.62462200
H 3.88116800 -0.80709800 0.95569300
C -3.12816600 -1.53898100 -0.36278300
H -3.03312900 -2.40822300 -1.03592500
H -3.23304800 -1.87386400 0.68831900

H -4.01724000 -0.93573000 -0.63421400

Imaginary Frequencies: 0

Electronic energy: -571.321613 hartree

TS$_{RSR}$ (IEFPCM(acetonitrile)-PBE/def2-SVP)

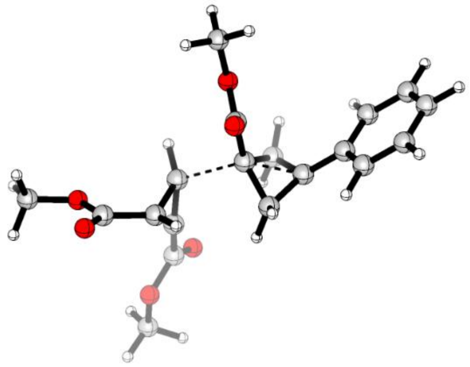

C -2.08734100 0.50927700 0.74483600
C -1.35833200 0.31997600 -0.58343700
H -1.48535200 0.50787000 1.67585200
C -2.11877900 -0.70008100 -0.14282400
C -2.81342900 -1.92513900 -0.47055500
O -2.52772800 -2.67500000 -1.40264500
O -3.83884400 -2.16295000 0.39434000
C -3.27431400 1.41796500 0.87118100
O -3.57110500 2.02823000 1.88746900
O -3.99030900 1.48814800 -0.27828200
C -5.14567000 2.33801300 -0.24522800
H -5.59892700 2.28028500 -1.25046900
H -5.86832300 1.99155200 0.52064600
H -4.86076000 3.38391100 -0.01315500
C -4.59577400 -3.35270800 0.14003300
H -5.36947600 -3.39876600 0.92678300
H -5.07260400 -3.31713200 -0.86061900
H -3.94954900 -4.25280400 0.18659700
C 0.67194400 0.05824700 -0.22031600
C 1.91319700 -1.10595200 0.00686400
C 1.12551900 -0.97730400 -1.25119200
C 0.81924800 -0.86273400 0.97809200
C 1.18944500 1.45179000 -0.15485100
C 3.34565300 -0.90446300 0.19103900
H 0.35055100 -1.74556600 -1.44584900
H 1.63792600 -0.62014400 -2.15956900
H -0.01407200 -1.60299200 1.01309600
H 1.06998200 -0.41926700 1.95550400
O 1.39590300 2.06840900 0.88064000
O 1.35491400 1.96533600 -1.39531700
C 3.89281100 -0.70098700 1.48494200
C 4.22653300 -0.93344400 -0.92189500
C 1.82789300 3.32206300 -1.45439000
C 5.27004000 -0.51720000 1.65397900
H 3.23230000 -0.69629400 2.36409000
C 5.60282400 -0.74846600 -0.74553100
H 3.82655100 -1.11499400 -1.93024600
H 1.88430800 3.57927600 -2.52621700
H 1.12825200 4.00299900 -0.93070100
H 2.82809800 3.40810900 -0.98553400
C 6.12985100 -0.53630100 0.54103300
H 5.67880000 -0.36106500 2.66375500
H 6.27251300 -0.77550200 -1.61840100
H 7.21263300 -0.39420900 0.67752600
H -1.21341600 0.93204200 -1.48341500

Imaginary Frequencies: 1

Electronic energy: -1185.022443 hartree

3a$_{RSR}$ (IEFPCM(acetonitrile)-PBE/def2-SVP)

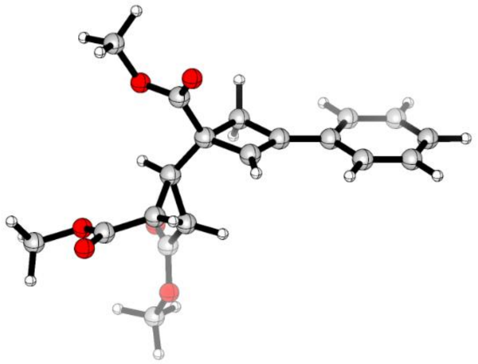

C -1.78060500 0.00310700 1.09548100
C -0.98122600 0.35770900 -0.15541000
H -1.31696500 0.22002400 2.06960500
C -1.07208500 -1.07075800 0.25937500
C -1.82439000 -2.05398400 -0.59994800
O -1.97829800 -1.96817100 -1.80626200
O -2.27068800 -3.08712500 0.14563300
C -3.26525200 0.14322000 1.15055100
O -3.88336900 0.48656100 2.14627700
O -3.85681100 -0.15119700 -0.03145600
C -5.28915300 -0.05095700 -0.06786500
H -5.58854400 -0.33984000 -1.09035400
H -5.74925700 -0.73263500 0.67469500
H -5.61513300 0.98546300 0.14958900
C -2.97410500 -4.11813500 -0.56736200
H -3.23558500 -4.88547600 0.18193600
H -3.89245200 -3.71125700 -1.03600500
H -2.33507900 -4.55557400 -1.35994100
C 0.32158100 1.14132500 -0.05611500
C 2.26547800 0.30522900 0.06027200
C 1.40604900 0.69989500 -1.12981500
C 1.33977200 0.69606800 0.98142900
C 0.06796400 2.64888400 -0.06436300
C 3.60848100 -0.26856800 0.11395400
H 1.06382400 -0.12680100 -1.78730100
H 1.80199900 1.52272200 -1.76100800
H -0.20599800 -1.51820900 0.77745900
H 1.31626100 0.71686300 2.08144600
O 0.60471100 3.45381000 0.67833800
O -0.81567300 2.99545800 -1.02643000
C 4.23921100 -0.55328100 1.35103800
C 4.30809800 -0.55224500 -1.08326200
C -1.10328100 4.39899400 -1.14527700
C 5.52629000 -1.10284100 1.38518800
H 3.70983700 -0.33915000 2.29239700
C 5.59786900 -1.10316200 -1.04645200
H 3.83198400 -0.33707300 -2.05251400
H -1.84620900 4.49157800 -1.95648700
H -1.51671800 4.79620600 -0.19713300
H -0.18576400 4.96674200 -1.39816500
C 6.21106800 -1.38018900 0.18667000
H 6.00313500 -1.31824600 2.35379400
H 6.12808100 -1.31774800 -1.98700800
H 7.22302900 -1.81234400 0.21649900
H -1.60000800 0.61819300 -1.02883900

Imaginary Frequencies: 0

Electronic energy: -1185.120727 hartree

BCB-1a (IEFPCM(acetonitrile)-TPSS/def2-SVP)

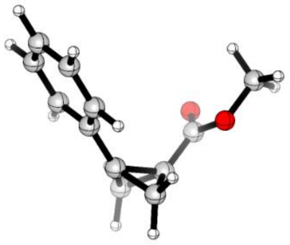

C -0.00439500 -1.40126000 0.46427500
C 0.99058500 -1.36861900 1.59077300
C 1.49481500 -1.12111600 0.19566800
C 0.78206100 -2.24755700 -0.49663000
H 1.27011500 -2.31947400 2.07177600
H 0.96151000 -0.50584900 2.26676000
H 1.04316900 -3.27592900 -0.20079200
H 0.58247100 -2.11437300 -1.56702200

C 2.01988600 0.09949300 -0.42942600
O 2.13685200 0.27239600 -1.63612100
O 2.34780500 1.03462800 0.49772500
C 2.83019400 2.28924200 -0.02688200
H 3.05723300 2.90911600 0.85093000
H 3.73663300 2.12817700 -0.63298800
H 2.05780600 2.76893400 -0.65022600
C -1.18461700 -0.55152600 0.22325600
C -1.87897200 -0.60945100 -1.00747500
C -1.65863400 0.33070300 1.22200300
C -2.99345200 0.20752100 -1.23943700
H -1.54867600 -1.30559700 -1.78499700
C -2.77346900 1.14590400 0.98600500
H -1.15537200 0.37259600 2.19326900
C -3.44448700 1.09165400 -0.24628700
H -3.51639300 0.14771200 -2.20017700
H -3.12386200 1.82285800 1.77279700
H -4.31898100 1.72571000 -0.42721500

Imaginary Frequencies: 0

Electronic energy: -614.578396 hartree

Cyclopropene (6a) (IEFPCM(acetonitrile)-TPSS/def2-SVP)

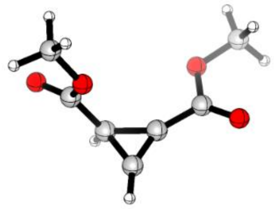

C 0.72956100 1.04910900 -0.82795900
C 0.24660200 2.03163500 0.22095400
H 0.72512000 1.33230600 -1.89206900
H 0.51014700 2.94048100 0.76030800
C -0.56452400 1.04313700 -0.03817600
C -1.83357700 0.34673600 0.21531500
O -2.68497100 0.73027900 1.00117300
O -1.92794400 -0.76919600 -0.53490700
C 1.82764000 0.07024200 -0.52237900
O 2.60742100 -0.36790700 -1.35303600
O 1.85600600 -0.27551600 0.78616300
C 2.88137500 -1.21767100 1.16638500
H 2.75198400 -1.37729700 2.24523800
H 2.75427800 -2.16397700 0.61582100
H 3.87997100 -0.80333900 0.95203700
C -3.14182300 -1.53643800 -0.36258100
H -3.04358900 -2.39701600 -1.03681500
H -3.23565300 -1.86767200 0.68412700
H -4.01787400 -0.92526400 -0.63314200

Imaginary Frequencies: 0

Electronic energy: -572.065100 hartree

TS$_{RSR}$ (IEFPCM(acetonitrile)-TPSS/def2-SVP)

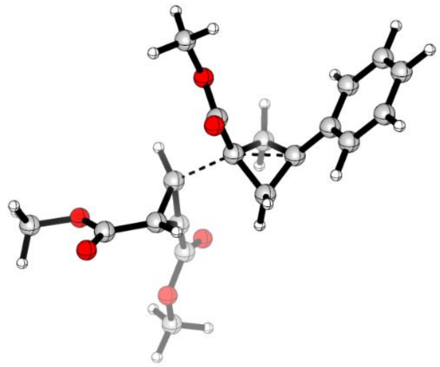

C -2.07169700 0.49229000 0.76474000
C -1.33531900 0.30652700 -0.56095200
H -1.47647800 0.49026200 1.69305300
C -2.10103500 -0.71554000 -0.12158700
C -2.82973200 -1.90885700 -0.47227200
O -2.58080100 -2.64324700 -1.42774500

O -3.84951300 -2.14973300 0.40169700
C -3.25610000 1.40432200 0.88167500
O -3.56209300 2.01377700 1.89539300
O -3.96196600 1.48090900 -0.27310200
C -5.11997500 2.34047500 -0.24519300
H -5.55543700 2.28494800 -1.25204600
H -5.84160700 1.98657300 0.50922400
H -4.82462800 3.37541400 -0.00666100
C -4.63904800 -3.32011300 0.11910500
H -5.39705500 -3.36598400 0.91320400
H -5.12160400 -3.23559500 -0.86906400
H -4.00911200 -4.22535400 0.13354000
C 0.64676700 0.06861900 -0.22256200
C 1.91761900 -1.10775900 0.00438000
C 1.12546200 -0.96798100 -1.24854000
C 0.82466600 -0.85884600 0.97252300
C 1.18494000 1.45568300 -0.15695600
C 3.34792000 -0.90741200 0.18691100
H 0.35876500 -1.73665600 -1.44172300
H 1.63147500 -0.61277600 -2.15459700
H -0.00445400 -1.59692100 1.00561200
H 1.07314800 -0.42431700 1.94826200
O 1.40173500 2.06970700 0.87731600
O 1.34918700 1.97207700 -1.39550600
C 3.89714400 -0.71877800 1.48188300
C 4.22689100 -0.93114100 -0.92732500
C 1.83234000 3.33400800 -1.45073700
C 5.27400500 -0.54554600 1.65056600
H 3.24042500 -0.71692100 2.35683400
C 5.60268700 -0.75645800 -0.75063900
H 3.82642900 -1.09870500 -1.93176200
H 1.88577700 3.58443500 -2.51831000
H 1.13416700 4.00562700 -0.92614500
H 2.82804400 3.40257200 -0.98410600
C 6.13123800 -0.55993100 0.53676300
H 5.68342600 -0.40169500 2.65580000
H 6.26846300 -0.77884400 -1.61958600
H 7.20952500 -0.42622500 0.67261400
H -1.23429500 0.92086600 -1.45926400

Imaginary Frequencies: 1

Electronic energy: -1186.629995 hartree

3a$_{RSR}$ (IEFPCM(acetonitrile)-TPSS/def2-SVP)

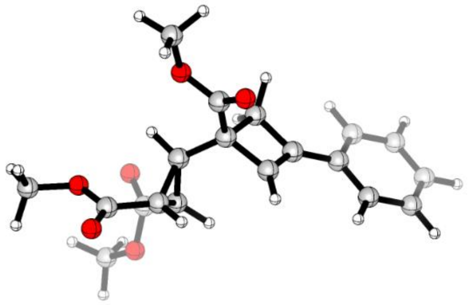

C -1.78522600 -0.00029500 1.09508900
C -0.98950600 0.34914700 -0.15906600
H -1.31931100 0.21577300 2.06174700
C -1.07355600 -1.07797500 0.26345600
C -1.81884100 -2.06950300 -0.59159700
O -2.02515100 -1.96326500 -1.78789000
O -2.19306100 -3.13944300 0.14173600
C -3.26845400 0.14341200 1.15425400
O -3.88168500 0.49359700 2.15030600
O -3.86689800 -0.15287100 -0.02226000
C -5.30674300 -0.04288600 -0.05058800
H -5.60387600 -0.33670000 -1.06599000
H -5.75531600 -0.71636600 0.69724000
H -5.61315800 0.99431600 0.16008800
C -2.87853200 -4.18791900 -0.57918100
H -3.07599500 -4.97520600 0.16040300
H -3.82215200 -3.80457000 -1.00023000
H -2.24346000 -4.56928600 -1.39498500
C 0.31239900 1.14025800 -0.06550600
C 2.26217100 0.31588500 0.05501600
C 1.40038700 0.69959400 -1.13883600
C 1.33584500 0.70537600 0.97103800
C 0.05162300 2.64628700 -0.08058400
C 3.61027900 -0.25038700 0.11329200
H 1.06447700 -0.12836700 -1.78852300
H 1.78863800 1.52001700 -1.76839200
H -0.21389900 -1.51728500 0.78633100
H 1.31153400 0.73067000 2.06538600
O 0.60648200 3.45992400 0.63844100
O -0.85974500 2.98481600 -1.01881500
C 4.24167500 -0.52361800 1.35143800
C 4.31205000 -0.53719200 -1.08052500
C -1.15302500 4.39531400 -1.13828600
C 5.53086100 -1.06487700 1.38959600
H 3.71422300 -0.30821100 2.28697700
C 5.60398700 -1.07979600 -1.03960500

H 3.83865300 -0.33218200 -2.04698200
H -1.91338700 4.47316000 -1.92665600
H -1.53955100 4.78598300 -0.18329200
H -0.24328400 4.95053900 -1.41849700
C 6.21751200 -1.34538600 0.19436400
H 6.00596000 -1.27038900 2.35500000
H 6.13325900 -1.29588700 -1.97393700
H 7.22694300 -1.76924300 0.22737600
H -1.60927300 0.59855000 -1.02780800

Imaginary Frequencies: 0

Electronic energy: -1186.727157 hartree

BCB-1a (IEFPCM(acetonitrile)-M06-2X/def2-SVP)

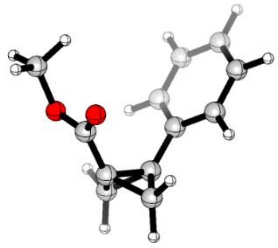

C 0.20813100 -1.47923100 0.50027200
C 1.24457300 -1.35719400 1.56580400
C 1.62302300 -1.06796900 0.15051200
C 0.99269500 -2.24865400 -0.50857500
H 1.64190400 -2.27427600 2.01451600
H 1.17624500 -0.49954400 2.23829500
H 1.36898200 -3.24192400 -0.24121800
H 0.71120900 -2.11821700 -1.55686500
C 1.84283900 0.23646900 -0.48561300
O 1.83921700 0.42504700 -1.67795000
O 1.97976300 1.21181200 0.41612200
C 2.09007500 2.53146700 -0.10554600
H 2.20195400 3.19437500 0.75698600
H 2.96407100 2.61140400 -0.76456900
H 1.18823100 2.79408800 -0.67417400
C -1.00583300 -0.67009300 0.28479400
C -1.79643600 -0.87089100 -0.85557400
C -1.37173200 0.33523800 1.19320000
C -2.91596700 -0.07379400 -1.09100000
H -1.53998700 -1.66539100 -1.55791800
C -2.48952300 1.13112200 0.95358100
H -0.78204000 0.49445500 2.09696700
C -3.26499000 0.93213800 -0.19021500
H -3.52123300 -0.24465700 -1.98257300
H -2.75981400 1.90945600 1.66884800
H -4.14249800 1.55370500 -0.37351400

Imaginary Frequencies: 0

Electronic energy: -614.195775 hartree

Cyclopropene (6a) (IEFPCM(acetonitrile)-M06-2X/def2-SVP)

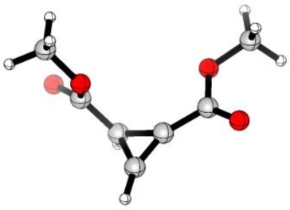

C 0.73008400 1.10351200 -0.78491500
C 0.25382400 2.03726100 0.30011100
H 0.74632400 1.42969000 -1.83103800
H 0.52687900 2.91154600 0.88288100
C -0.55162800 1.07692600 -0.01261100
C -1.81434700 0.34586800 0.19994500
O -2.68485800 0.70669400 0.94669200
O -1.85600800 -0.75876700 -0.53305900
C 1.80153700 0.09725400 -0.49957700
O 2.59194900 -0.29584400 -1.31981800
O 1.78586200 -0.32522500 0.76565800
C 2.77127700 -1.28929100 1.11790900
H 2.61303100 -1.51984400 2.17515100
H 2.65549200 -2.19469400 0.50815800
H 3.77849300 -0.88132900 0.96274200
C -3.03250200 -1.55321600 -0.39794500

H -2.89927200 -2.41151600 -1.06145300
H -3.14890100 -1.88499200 0.64169000
H -3.91708200 -0.97361300 -0.69142400

Imaginary Frequencies: 0

Electronic energy: -571.732303 hartree

TS_RSR_ (IEFPCM(acetonitrile)- M06-2X/def2-SVP)

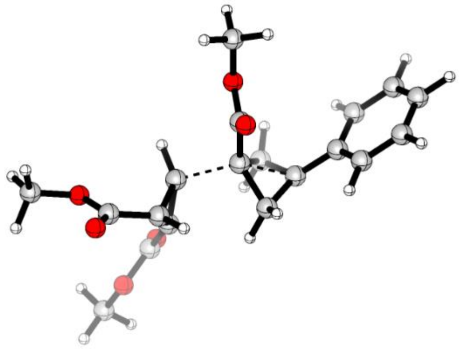

C -1.94750000 0.47819200 0.84576100
C -1.20891400 0.19144600 -0.44272600
H -1.37775100 0.51399100 1.78532000
C -2.02507600 -0.76865100 0.04786700
C -2.98699000 -1.74232200 -0.39298500
O -2.87184100 -2.46637600 -1.36130400
O -4.06565600 -1.78074500 0.41184400
C -3.08195900 1.45011500 0.84963600
O -3.40340600 2.11873400 1.80055100
O -3.72191300 1.49587700 -0.32249500
C -4.82783600 2.38574600 -0.39876000
H -5.21921500 2.30579300 -1.41695900
H -5.59909800 2.10071100 0.32886900
H -4.50861600 3.41578900 -0.19259500
C -5.07531500 -2.70932800 0.05013800
H -5.86530000 -2.61589500 0.80161400
H -5.47520600 -2.48297300 -0.94759400
H -4.67830400 -3.73355000 0.04420300
C 0.63773800 -0.07834800 -0.12662100
C 1.82243300 -1.23317800 0.18349800
C 1.02418900 -1.24894700 -1.04398500
C 0.79575400 -0.87723800 1.15995300
C 1.28842600 1.25719500 -0.22465400
C 3.25916500 -0.96336100 0.27904800
H 0.18855600 -1.96184300 -1.09710300
H 1.51448400 -1.03065800 -1.99473000
H -0.07998400 -1.54828100 1.25552800
H 1.09720200 -0.35734600 2.07145900
O 1.52444600 1.96121700 0.72341400
O 1.55166100 1.57888000 -1.48630600
C 3.83340300 -0.57467000 1.50110500
C 4.07723600 -1.08158500 -0.85728600
C 2.18306800 2.84144600 -1.69429500
C 5.19481300 -0.29920900 1.57908400
H 3.21589800 -0.49484400 2.39605700
C 5.43791900 -0.80275300 -0.77385300
H 3.65034900 -1.40541700 -1.80701400
H 2.32135400 2.93976600 -2.77407000
H 1.54796000 3.65030000 -1.31212500
H 3.15153500 2.86688000 -1.17847300
C 5.99851600 -0.40833400 0.44243800
H 5.63205200 0.00042500 2.53206900
H 6.06517200 -0.89891900 -1.66067200
H 7.06579500 -0.19221200 0.50651100
H -1.14762400 0.77028500 -1.36610500

Imaginary Frequencies: 1

Electronic energy: -1185.911796 hartree

3a$_{RSR}$ (IEFPCM(acetonitrile)-M06-2X/def2-SVP)

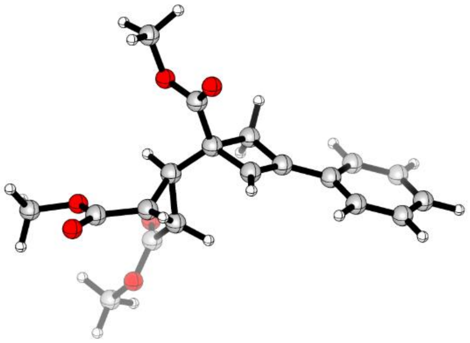

C -1.70982300 0.00756100 1.10713000
C -0.95095700 0.39669700 -0.14242300
H -1.24713000 0.20079100 2.07556000
C -1.04175000 -1.03191400 0.23219600
C -1.86429100 -1.95649400 -0.61888000
O -1.87499300 -1.95420000 -1.82127400
O -2.56272700 -2.81061000 0.12534000
C -3.19442700 0.09259800 1.14738600
O -3.83775500 0.29717400 2.14426400
O -3.74668100 -0.09949900 -0.05383000
C -5.16948400 -0.10859500 -0.10452000
H -5.43715200 -0.27232800 -1.15209100
H -5.56533100 -0.91793400 0.52300900
H -5.57016500 0.84982400 0.24868800
C -3.39339500 -3.72333600 -0.58478500
H -3.88511300 -4.34377000 0.16947800
H -4.13965500 -3.17368400 -1.17412400
H -2.79188400 -4.34509400 -1.26014400
C 0.36356000 1.14053600 -0.03472900
C 2.26464900 0.26504200 0.07093300
C 1.42170300 0.69595800 -1.11029900
C 1.36520300 0.65755400 0.99163500
C 0.14193800 2.64121500 -0.02508600
C 3.59331000 -0.34755300 0.11080800
H 1.05912500 -0.10988600 -1.76685200
H 1.83646100 1.51208600 -1.72088400
H -0.16379100 -1.50959900 0.67846900
H 1.34366000 0.65671100 2.08293600
O 0.66691900 3.41213600 0.73568000
O -0.69408400 3.02235400 -0.99053000
C 4.21004300 -0.66192500 1.33261500
C 4.26625900 -0.62946200 -1.08569800
C -0.94707100 4.42041500 -1.09128400
C 5.47253100 -1.24569800 1.35279400
H 3.69238800 -0.44517900 2.26927300
C 5.53179900 -1.21494900 -1.06395800
H 3.79152000 -0.38742800 -2.03903400
H -1.65093700 4.54681400 -1.91845000
H -1.38225700 4.79754300 -0.15698100
H -0.01487600 4.96262800 -1.29593100
C 6.13671300 -1.52386200 0.15415600
H 5.94426200 -1.48645900 2.30660400
H 6.04683500 -1.43057500 -2.00116200
H 7.12677100 -1.98186300 0.17240400
H -1.57722400 0.69584700 -0.98593400

Imaginary Frequencies: 0

Electronic energy: -1186.025435 hartree

BCB-1a (IEFPCM(acetonitrile)-M06-2X/def2-TZVP)

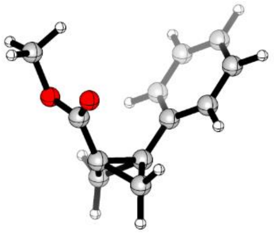

C 0.14678400 -1.46437000 0.48218800
C 1.16973800 -1.38180200 1.56093800
C 1.57837900 -1.09267500 0.15460600
C 0.92150800 -2.25183800 -0.51563600
H 1.53171200 -2.30647500 1.99993600
H 1.11482700 -0.53902500 2.23761300
H 1.26567600 -3.24464500 -0.24328700
H 0.66027100 -2.11845400 -1.55828400
C 1.86371400 0.19632000 -0.46661600
O 1.86412800 0.39413600 -1.65959500

O 2.07857600 1.15364700 0.43993000
C 2.28421200 2.46846300 -0.08335300
H 2.44227900 3.10843600 0.77846500
H 3.15667200 2.48189400 -0.73398500
H 1.40829100 2.79142400 -0.64330700
C -1.04451800 -0.63331200 0.26384800
C -1.80529200 -0.78308800 -0.89674300
C -1.43069000 0.32736400 1.20152100
C -2.91149300 0.02351800 -1.12446000
H -1.53766200 -1.54221600 -1.62006000
C -2.53599000 1.13240000 0.97051400
H -0.86927700 0.44293000 2.11941000
C -3.27949500 0.98649600 -0.19439100
H -3.49038600 -0.10634800 -2.02973700
H -2.82076900 1.87299300 1.70671200
H -4.14440900 1.61236300 -0.37065100

Imaginary Frequencies: 0

Electronic energy: -614.882876 hartree

Cyclopropene (6a) (IEFPCM(acetonitrile)-M06-2X/def2-TZVP)

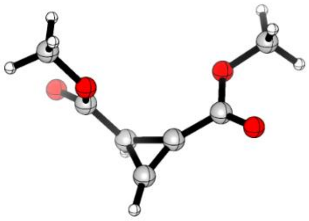

C 0.73081800 1.09561500 -0.77557700
C 0.24009900 2.01573700 0.31158700
H 0.74579700 1.43258900 -1.80679000
H 0.50358300 2.88044800 0.89329000
C -0.55104800 1.05925700 -0.00904000
C -1.81380100 0.33923700 0.20137500
O -2.67203500 0.70462400 0.96111000
O -1.87471300 -0.75429100 -0.54523400
C 1.80987900 0.10469300 -0.49367900
O 2.61535200 -0.25665100 -1.31612500
O 1.78810400 -0.34306300 0.76128800
C 2.79408300 -1.30040800 1.10094700
H 2.62719900 -1.54953300 2.14363300
H 2.69523300 -2.18600100 0.47595000
H 3.78405800 -0.86899300 0.96486800
C -3.06865900 -1.53468300 -0.40569200
H -2.94899600 -2.37791900 -1.07685200
H -3.17306000 -1.87274800 0.62338800
H -3.93569800 -0.93948000 -0.68532900

Imaginary Frequencies: 0

Electronic energy: -572.395355 hartree

TS$_{RSR}$ (IEFPCM(acetonitrile)- M06-2X/def2-TZVP)

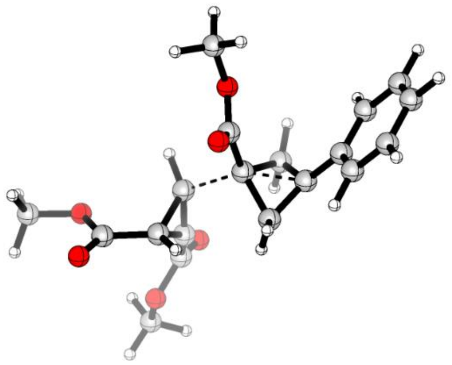

C -1.95652600 0.46352100 0.83090400
C -1.22595900 0.19469100 -0.46411600
H -1.38378100 0.47255300 1.75632900
C -2.04352100 -0.76284000 0.00706700
C -2.98226700 -1.75125000 -0.41862700
O -2.87887800 -2.44911600 -1.41036100
O -4.03101400 -1.84426100 0.42250900
C -3.06884000 1.45277200 0.85953700
O -3.36150600 2.11110500 1.82874200
O -3.72027100 1.53427400 -0.30255300

C -4.80748700 2.45992300 -0.34529500
H -5.20902000 2.40102900 -1.35165100
H -5.56609100 2.18230000 0.38453200
H -4.45394900 3.46761500 -0.13391600
C -5.02429100 -2.80406800 0.06964900
H -5.78274800 -2.74572000 0.84427000
H -5.45737500 -2.56783500 -0.90122600
H -4.59398000 -3.80400200 0.03650100
C 0.62438000 -0.07059500 -0.14624400
C 1.82518500 -1.21647700 0.17653200
C 1.03279800 -1.24502200 -1.05148600
C 0.78899300 -0.86867100 1.14258000
C 1.26530600 1.26383700 -0.24786700
C 3.25455200 -0.94538000 0.28279900
H 0.21517700 -1.96454100 -1.09945200
H 1.51899800 -1.03552000 -1.99478000
H -0.06842200 -1.54694400 1.23528900
H 1.07425000 -0.35213300 2.04936000
O 1.46020700 1.98721600 0.69627300
O 1.56018100 1.57183800 -1.50624700
C 3.81858200 -0.57312500 1.50733200
C 4.07956500 -1.06976800 -0.83985500
C 2.17292500 2.85177900 -1.70864300
C 5.17554700 -0.31414400 1.60007200
H 3.19828000 -0.49199300 2.38947400
C 5.43589600 -0.80837300 -0.74182100
H 3.66225300 -1.38239100 -1.78753500
H 2.33813000 2.93352400 -2.77736400
H 1.51004900 3.64093300 -1.35994300
H 3.11680800 2.89921400 -1.16877300
C 5.98564200 -0.42684100 0.47624600
H 5.60311600 -0.02660400 2.55119500
H 6.06623500 -0.90766200 -1.61527700
H 7.04601300 -0.22490700 0.55156800
H -1.16657100 0.78481700 -1.36808600

Imaginary Frequencies: 1

Electronic energy: -1187.258943 hartree

3a$_{RSR}$ (IEFPCM(acetonitrile)-M06-2X/def2-TZVP)

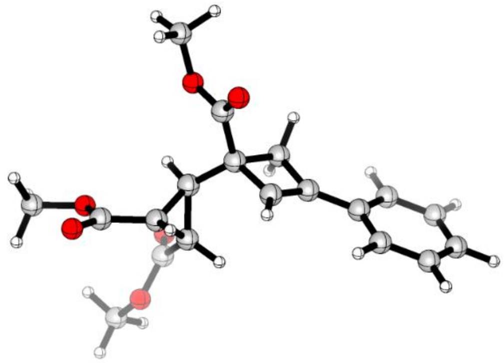

C -1.72190500 0.01236900 1.07651700
C -0.95312800 0.38297800 -0.17139600
H -1.25901100 0.21430400 2.03198600
C -1.04610200 -1.03768500 0.21994800
C -1.85576200 -1.98355000 -0.61270200
O -1.92065700 -1.96070800 -1.81341400
O -2.46842000 -2.88869600 0.14616600
C -3.19937700 0.11980900 1.12027700
O -3.82514900 0.37473100 2.11865700
O -3.76661200 -0.10325700 -0.06582100
C -5.19631200 -0.06876900 -0.09940000
H -5.47246200 -0.28040500 -1.12703500
H -5.60315400 -0.82456700 0.57001600
H -5.55419700 0.91452000 0.19909500
C -3.27015800 -3.84725000 -0.55129700
H -3.69141900 -4.49321300 0.21164000
H -4.06001900 -3.33889100 -1.10212000
H -2.65501500 -4.42053000 -1.24220700
C 0.35271100 1.13307900 -0.04700500
C 2.25598100 0.27156900 0.06496300
C 1.42658600 0.71569200 -1.11888100
C 1.34957500 0.64077900 0.97661800
C 0.12594600 2.62999100 -0.02114900
C 3.58312300 -0.33164100 0.10939300
H 1.08262400 -0.07357100 -1.78947100
H 1.83845200 1.54088700 -1.70182200
H -0.18545300 -1.50556300 0.68421600
H 1.31412500 0.62154200 2.05806200
O 0.65472100 3.39179600 0.74723300
O -0.71196500 3.02080700 -0.98037800
C 4.18176700 -0.66705000 1.32698700
C 4.27070000 -0.58230400 -1.07737900
C -0.95671500 4.42817800 -1.06661600
C 5.44133700 -1.24062500 1.35260300
H 3.65310800 -0.47513700 2.25290300
C 5.53407100 -1.15790400 -1.05098000
H 3.81078800 -0.32411100 -2.02387300
H -1.65448000 4.55804100 -1.88707300

H -1.38850900 4.79056800 -0.13570700
H -0.02718800 4.95776400 -1.26706600
C 6.12103600 -1.48775600 0.16280100
H 5.89791100 -1.49717500 2.29973300
H 6.05935600 -1.34845400 -1.97775600
H 7.10563300 -1.93641400 0.18511800
H -1.56668900 0.66356200 -1.01798800

Imaginary Frequencies: 0

Electronic energy: -1187.369170 hartree

BCB-1a (IEFPCM(acetonitrile)-M06-2X/def2-TZVPP)

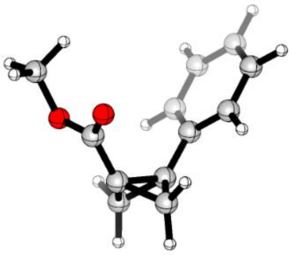

C 0.14951500 -1.47219500 0.45720400
C 1.16130500 -1.39860400 1.54688300
C 1.58196500 -1.08699200 0.14881000
C 0.93905800 -2.23995900 -0.54455700
H 1.52555500 -2.32577200 1.97686800
H 1.09354700 -0.56548800 2.23335600
H 1.28563300 -3.23395700 -0.28253900
H 0.68837600 -2.09396000 -1.58726400
C 1.86679100 0.21152500 -0.45248900
O 1.87599100 0.42505400 -1.64277100
O 2.07073300 1.15759100 0.46820300
C 2.27567400 2.47959500 -0.03628600
H 2.42485300 3.10894700 0.83400300
H 3.15238600 2.50468400 -0.67962200
H 1.40360000 2.80676700 -0.59845200
C -1.04449100 -0.64372100 0.24469600
C -1.76282700 -0.72989400 -0.94920600
C -1.47604000 0.25228200 1.22523700
C -2.87019400 0.07763600 -1.16605000
H -1.46143800 -1.43903000 -1.70813000
C -2.58294900 1.05840700 1.00517900
H -0.94960000 0.31463000 2.16794100
C -3.28282400 0.97740400 -0.19224600
H -3.41509900 -0.00202700 -2.09712900
H -2.90294100 1.74783500 1.77490900
H -4.14855600 1.60329200 -0.36044300

Imaginary Frequencies: 0

Electronic energy: -614.887543 hartree

Cyclopropene (6a) (IEFPCM(acetonitrile)-M06-2X/def2-TZVPP)

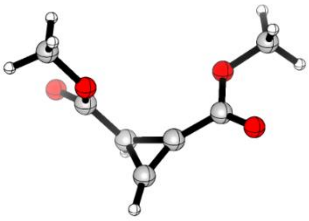

C 0.73094900 1.09578200 -0.77524800
C 0.23972200 2.01586800 0.31176600
H 0.74609900 1.43269100 -1.80577200
H 0.50286800 2.88001200 0.89328900
C -0.55116100 1.05928400 -0.00919100
C -1.81387300 0.33912900 0.20106300
O -2.67241000 0.70456200 0.96042100
O -1.87450100 -0.75471200 -0.54516500
C 1.80992600 0.10483500 -0.49345900
O 2.61532000 -0.25664100 -1.31591700
O 1.78826700 -0.34296000 0.76153300
C 2.79410300 -1.30027400 1.10047300
H 2.62808900 -1.55019100 2.14238400
H 2.69544100 -2.18502400 0.47545900
H 3.78351600 -0.86930700 0.96442500
C -3.06834200 -1.53467200 -0.40533400
H -2.94968000 -2.37774600 -1.07573200
H -3.17282000 -1.87231400 0.62313300

H -3.93487000 -0.93982800 -0.68457600

Imaginary Frequencies: 0

Electronic energy: -572.398390 hartree

TS$_{RSR}$ (IEFPCM(acetonitrile)- M06-2X/def2-TZVPP)

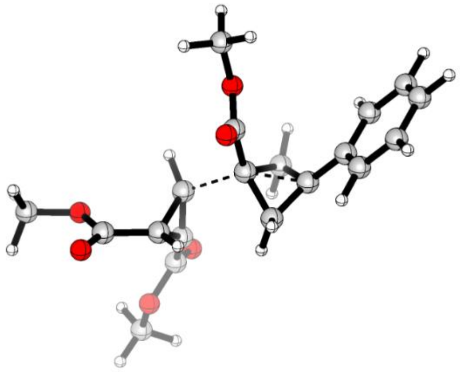

C -1.95625000 0.46325500 0.82990200
C -1.22653600 0.19570400 -0.46578300
H -1.38284400 0.47184300 1.75420600
C -2.04299600 -0.76266200 0.00545100
C -2.98076500 -1.75238800 -0.41958800
O -2.87745600 -2.44975400 -1.41163000
O -4.02849400 -1.84732300 0.42261200
C -3.06862800 1.45228500 0.86044500
O -3.35973000 2.11068300 1.83006400
O -3.72200400 1.53381000 -0.30058400
C -4.80859600 2.45986300 -0.34119600
H -5.21267500 2.40133700 -1.34580900
H -5.56529200 2.18339700 0.39001300
H -4.45442900 3.46685100 -0.13106800
C -5.02005900 -2.80871000 0.07042100
H -5.77804900 -2.75224200 0.84466900
H -5.45396700 -2.57365500 -0.89961100
H -4.58850300 -3.80735300 0.03691200
C 0.62466100 -0.06866700 -0.14867300
C 1.82423100 -1.21546800 0.17458600
C 1.03347000 -1.24242200 -1.05451500
C 0.78781300 -0.86742800 1.13996000
C 1.26517500 1.26597900 -0.24914800
C 3.25369400 -0.94551200 0.28250300
H 0.21643400 -1.96137100 -1.10468100
H 1.52155600 -1.03254200 -1.99594700
H -0.07052100 -1.54383200 1.23130300
H 1.07317800 -0.35208400 2.04654000
O 1.46095400 1.98824300 0.69567200
O 1.55862900 1.57582000 -1.50745000
C 3.81662900 -0.57404800 1.50769300
C 4.07983200 -1.07043800 -0.83915900
C 2.17027400 2.85623800 -1.70831800
C 5.17366300 -0.31633800 1.60208100
H 3.19549600 -0.49247400 2.38846300
C 5.43624300 -0.81029800 -0.73955200
H 3.66331000 -1.38213700 -1.78679800
H 2.33401500 2.94064200 -2.77634700
H 1.50811200 3.64400900 -1.35735700
H 3.11431200 2.90335000 -1.17011200
C 5.98488200 -0.42955900 0.47918800
H 5.60015400 -0.02946100 2.55317000
H 6.06713500 -0.90988800 -1.61179300
H 7.04472900 -0.22873600 0.55572600
H -1.16776700 0.78618500 -1.36873000

Imaginary Frequencies: 1

Electronic energy: -1187.266662 hartree

3a$_{RSR}$ (IEFPCM(acetonitrile)-M06-2X/def2-TZVPP)

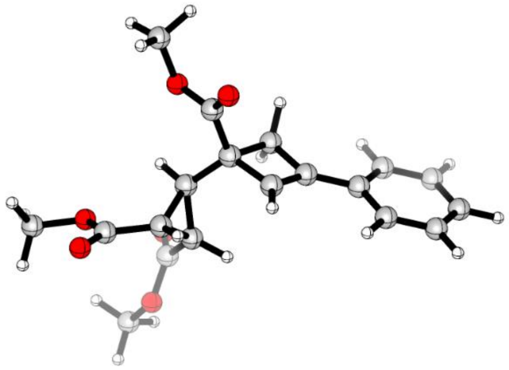

C -1.72135300 0.01111600 1.07681200
C -0.95379000 0.38308000 -0.17138800
H -1.25744200 0.21212200 2.03120500
C -1.04583900 -1.03793900 0.21871500
C -1.85522000 -1.98354800 -0.61442100
O -1.92476600 -1.95681500 -1.81477400
O -2.46185400 -2.89355900 0.14353900
C -3.19864800 0.11925100 1.12272600
O -3.82272200 0.37723500 2.12137800
O -3.76795200 -0.10656300 -0.06188700
C -5.19738200 -0.06923700 -0.09316700
H -5.47610100 -0.28401800 -1.11872900
H -5.60458700 -0.82104200 0.57937400
H -5.55263000 0.91519300 0.20209700
C -3.26223100 -3.85236900 -0.55453700
H -3.67730400 -4.50353100 0.20637100
H -4.05632000 -3.34559100 -1.09936400
H -2.64821800 -4.41948100 -1.25041200
C 0.35171200 1.13379400 -0.04770800
C 2.25519400 0.27245800 0.06492600
C 1.42581000 0.71556100 -1.11921300
C 1.34882000 0.64255700 0.97618800
C 0.12542900 2.63081000 -0.02378600
C 3.58236900 -0.33057900 0.10991500
H 1.08225900 -0.07377800 -1.78879500
H 1.83759900 1.53976100 -1.70229700
H -0.18541400 -1.50551900 0.68215000
H 1.31370100 0.62450600 2.05707300
O 0.65849600 3.39406300 0.74017000
O -0.71697900 3.02008500 -0.97974000
C 4.18104500 -0.66499000 1.32769400
C 4.26999900 -0.58223100 -1.07654700
C -0.96059800 4.42725000 -1.06782500
C 5.44058400 -1.23846600 1.35383300
H 3.65269100 -0.47249700 2.25294500
C 5.53333300 -1.15772800 -1.04969000
H 3.81033600 -0.32503000 -2.02272900
H -1.66186800 4.55664400 -1.88444500
H -1.38700300 4.79212500 -0.13622100
H -0.03222400 4.95528700 -1.27384200
C 6.12026600 -1.48653400 0.16430900
H 5.89687500 -1.49407800 2.30064600
H 6.05832900 -1.34893300 -1.97577600
H 7.10431300 -1.93488500 0.18699200
H -1.56777900 0.66365200 -1.01674800

Imaginary Frequencies: 0

Electronic energy: -1187.376801 hartree

BCB-1a (IEFPCM(actonitrile)-DLPNO-CCSD(T)/def2-TZVPP//B2PLYP(D3)/def2-SVP)

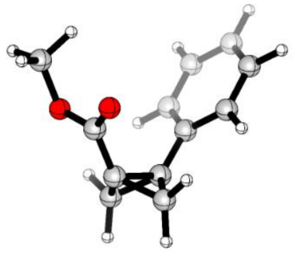

C 0.17278100 -1.50201800 0.45108000
C 1.19217500 -1.39656900 1.54098000
C 1.62578600 -1.07104800 0.14389500
C 0.99658500 -2.22825700 -0.56587900
H 1.57894000 -2.32110600 1.98600400
H 1.10278200 -0.55790200 2.23429000
H 1.36160700 -3.23355400 -0.32485400
H 0.74664600 -2.07079500 -1.61776900

C 1.87126800 0.24458800 -0.45179200
O 1.89134700 0.46689400 -1.64730000
O 2.01837600 1.19465400 0.48719100
C 2.16887200 2.53318600 0.00523800
H 2.28547200 3.16178600 0.89285500
H 3.05376100 2.61533900 -0.63854400
H 1.28241300 2.84041100 -0.56454700
C -1.03055800 -0.68391800 0.24004600
C -1.72302300 -0.72827800 -0.98471500
C -1.50080500 0.17684100 1.25014800
C -2.83836000 0.08470900 -1.20073100
H -1.39307700 -1.40901100 -1.77006800
C -2.61572100 0.98872800 1.02982800
H -0.99668200 0.20621500 2.21655800
C -3.28757500 0.94973000 -0.19708300
H -3.36213600 0.03740300 -2.15716300
H -2.96505500 1.65098100 1.82403100
H -4.16099600 1.58168400 -0.36601900

Imaginary Frequencies: 0

Electronic energy (B2PLYP(D3)/def2-SVP): -613.184339 hartree

Electronic energy (DLPNO-CCSD(T)/def2-TZVPP): -613.843955 hartree

Corrected Gibbs Free Energy (B2PLYP(D3)/def2-SVP): -613.008145 hartree

Cyclopropene (6a) (IEFPCM(actonitrile)-DLPNO-CCSD(T)/def2-TZVPP//B2PLYP(D3)/def2-SVP)

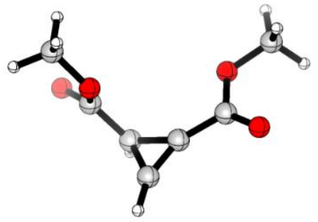

C 0.73151400 1.08979400 -0.79832200
C 0.25098400 2.04266600 0.27577600
H 0.73871800 1.40337000 -1.84903800
H 0.51743800 2.92467200 0.84953400
C -0.55723500 1.07009300 -0.02248300
C -1.81701600 0.34959400 0.20322400
O -2.68723100 0.71848600 0.96161000
O -1.86571800 -0.76322300 -0.53302800
C 1.80631200 0.09031100 -0.50807500
O 2.59151600 -0.32188200 -1.33567700
O 1.80084700 -0.30547800 0.77386800
C 2.79115700 -1.27009600 1.14142000
H 2.63547800 -1.47309900 2.20492300
H 2.66881800 -2.19026900 0.55595600
H 3.79963000 -0.87109400 0.97338500
C -3.04932400 -1.55963900 -0.38907100
H -2.91543400 -2.41779300 -1.05309300
H -3.16235500 -1.89242300 0.65031800
H -3.93595000 -0.98293200 -0.68099000

Imaginary Frequencies: 0

Electronic energy (B2PLYP(D3)/def2-SVP): -570.878173 hartree

Electronic energy (DLPNO-CCSD(T)/def2-TZVPP): -571.510223 hartree

Corrected Gibbs Free Energy (B2PLYP(D3)/def2-SVP): -570.768357 hartree

TS$_{RSR}$ (IEFPCM(actonitrile)-DLPNO-CCSD(T)/def2-TZVPP//B2PLYP(D3)/def2-SVP)

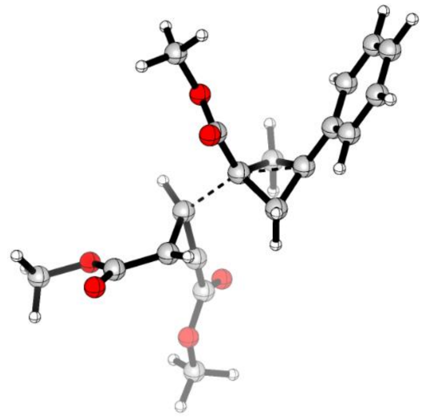

C -1.99948900 0.50424200 0.78761200
C -1.27304600 0.26603100 -0.52904100
H -1.40474400 0.51580500 1.71131200
C -2.06414400 -0.71812600 -0.05825500
C -2.91665600 -1.81455800 -0.43761000
O -2.73651700 -2.55378100 -1.39294700
O -3.96200600 -1.94392400 0.40881400
C -3.15944800 1.43996200 0.86630100
O -3.45968100 2.07805800 1.85450200
O -3.84732800 1.49462500 -0.28651000
C -4.98510300 2.35989200 -0.30156600
H -5.41006300 2.28389000 -1.30678200
H -5.72172800 2.04171100 0.44744400
H -4.68786500 3.39541800 -0.09126100
C -4.87727200 -2.99804400 0.11137300
H -5.64531900 -2.96106300 0.89013100
H -5.33472500 -2.85361900 -0.87650700
H -4.37041400 -3.97218400 0.12596900
C 0.63609300 -0.01054500 -0.20235500
C 1.87109600 -1.20328100 0.06207000
C 1.08252800 -1.09170500 -1.18667300
C 0.79515000 -0.89453200 1.01986600
C 1.24967100 1.34255900 -0.18910300
C 3.29574700 -0.96309000 0.22252500
H 0.28879300 -1.83690400 -1.34087700
H 1.58991700 -0.78470200 -2.10412100
H -0.06053800 -1.59764500 1.07043300
H 1.05634300 -0.43447900 1.97538500
O 1.48210200 1.97918900 0.81762900
O 1.47242000 1.78344800 -1.43295300
C 3.84243200 -0.69357600 1.49580900
C 4.15595000 -0.99817300 -0.89639000
C 2.04692400 3.09244700 -1.54521300
C 5.20794900 -0.45209300 1.63967600
H 3.19671400 -0.68063600 2.37383400
C 5.52020500 -0.75323200 -0.74527200
H 3.75414800 -1.22764900 -1.88354600
H 2.14376800 3.28678100 -2.61685700
H 1.39275300 3.83831400 -1.07706400
H 3.03083900 3.11917800 -1.06052900
C 6.05027000 -0.47645300 0.52106800
H 5.61897100 -0.24517200 2.62891700
H 6.17488400 -0.78306600 -1.61754300
H 7.11861200 -0.28725400 0.63705600
H -1.19541900 0.85599400 -1.44260700

Imaginary Frequencies: 1

Electronic energy (B2PLYP(D3)/def2-SVP): -1184.037519 hartree

Electronic energy (DLPNO-CCSD(T)/def2-TZVPP): -1185.336998 hartree

Corrected Gibbs Free Energy (B2PLYP(D3)/def2-SVP): -1183.732753 hartree

3a$_{RSR}$ (IEFPCM(actonitrile)-DLPNO-CCSD(T)/def2-TZVPP//B2PLYP(D3)/def2-SVP)

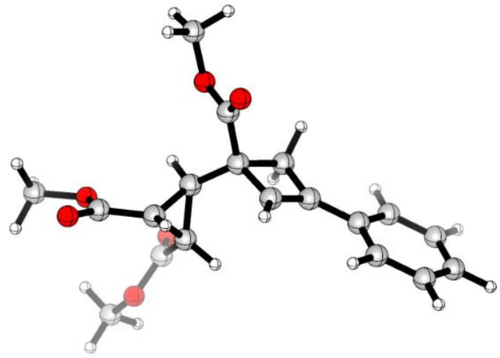

C -1.73849300 0.01394400 1.09678700
C -0.95349000 0.38727600 -0.14847700
H -1.28759700 0.22679100 2.06680500
C -1.03874400 -1.03892700 0.24985200
C -1.81733100 -1.99985800 -0.59952500
O -1.83287100 -2.00193800 -1.81019600
O -2.46475200 -2.89246900 0.15874400
C -3.22115800 0.10494400 1.12358600
O -3.87455500 0.35127000 2.11472700
O -3.76350400 -0.12938800 -0.08257000
C -5.19276600 -0.12548100 -0.15448900
H -5.44054700 -0.32975000 -1.19993100
H -5.61009200 -0.90455500 0.49593300
H -5.59053500 0.85045000 0.14989000
C -3.24196300 -3.86919500 -0.54269000
H -3.67734600 -4.51632200 0.22403900
H -4.03449600 -3.37943400 -1.12304400
H -2.60844700 -4.45333400 -1.22190400
C 0.34682600 1.15607700 -0.03441100
C 2.26718300 0.29522500 0.06727900
C 1.41753000 0.72825300 -1.11425900
C 1.35554500 0.68028100 0.99328600
H -1.56720000 0.66484100 -1.00714200
C 0.08316500 2.64904400 -0.00676300
C 3.59500200 -0.30898400 0.10812100
H 1.06804100 -0.07395600 -1.78189700
H 1.82284300 1.55483800 -1.71696700
H -0.16581500 -1.49257100 0.72879800
H 1.33207300 0.67412200 2.08419800
O 0.51961100 3.41974200 0.82044800
O -0.69431600 3.02508100 -1.03171500
C 4.21067400 -0.63486400 1.33339400
C 4.28211900 -0.57745300 -1.08981000
C -1.00441800 4.42074300 -1.11550900
C 5.47960700 -1.21268200 1.35560400
H 3.68738100 -0.43239700 2.26993200
C 5.55387200 -1.15705300 -1.06548800
H 3.81478900 -0.32968900 -2.04480300
H -1.64392300 4.53545300 -1.99531500
H -1.53328500 4.75166200 -0.21282100
H -0.08704000 5.01187100 -1.23047300
C 6.15574600 -1.47588400 0.15598700
H 5.94632100 -1.46044900 2.31060100
H 6.07583700 -1.36005200 -2.00220300
H 7.14868500 -1.92834400 0.17595800

Imaginary Frequencies: 0

Electronic energy (B2PLYP(D3)/def2-SVP): -1184.167325 hartree

Electronic energy (DLPNO-CCSD(T)/def2-TZVPP): -1185.457977 hartree

Corrected Gibbs Free Energy (B2PLYP(D3)/def2-SVP): -1183.857872 hartree

TS*RRR* (IEFPCM(actonitrile)-DLPNO-CCSD(T)/def2-TZVPP//B2PLYP(D3)/def2-SVP)

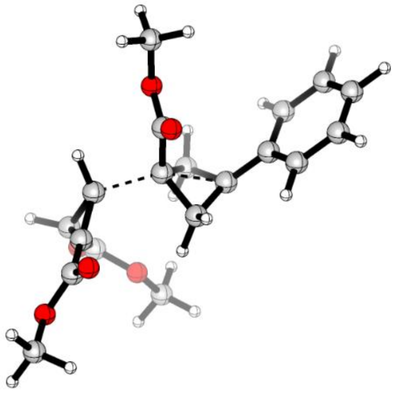

C 2.49533700 0.61421500 0.37414800
C 1.44611600 0.41713400 1.21238600
C 2.62652900 -0.57785200 1.23734100
C 2.57222100 -1.98572100 0.73445300
O 2.95422100 -2.93973400 1.38087100
O 2.10609400 -2.10042300 -0.52234900
C 3.17014500 1.55421500 -0.47163900
O 2.66436600 2.55927100 -0.95118000
O 4.45087400 1.18673800 -0.71616000
C 5.19332600 2.05997000 -1.56465700
H 6.19026300 1.61774100 -1.65828600
H 4.72241200 2.14104100 -2.55374200
H 5.26739200 3.06559200 -1.12883800
C 2.06403200 -3.42526800 -1.06379200
H 1.65957400 -3.32368000 -2.07513900
H 3.07166700 -3.85802900 -1.09610800
H 1.41727900 -4.07307000 -0.45782300
C -0.26885300 0.04909100 0.48791800
C -1.38108400 -0.81045900 -0.61484300
C -0.78674900 -1.39625000 0.61642000
C -0.23004400 0.02067600 -1.03316200
C -1.10787200 1.14219400 1.07553200
C -2.78253800 -0.52209000 -0.83529500
H -0.00628800 -2.14373200 0.46790500
H -1.45171500 -1.62674600 1.45255300
H 3.27655400 -0.54465300 2.12421800
H 0.68604100 -0.48649500 -1.37018500
O -1.29287500 2.21884800 0.54900600
O -1.58135700 0.79924400 2.27838700
C -3.17896600 0.39250600 -1.83772400
C -3.77926000 -1.15387400 -0.05623000
C -2.38740900 1.78185600 2.94354200
C -4.52826400 0.67503400 -2.04091900
H -2.42530300 0.87385300 -2.46074100
C -5.12593200 -0.86144200 -0.26200200
H -3.49426800 -1.87990800 0.70532400
H -2.67109000 1.33812100 3.90175100
H -1.81378300 2.70342100 3.10117700
H -3.27966400 2.00762700 2.34637800
C -5.50545200 0.05476500 -1.25180600
H -4.82187000 1.38184700 -2.81844600
H -5.88568300 -1.35373900 0.34692300
H -6.56114200 0.27877600 -1.41294800
H 1.10526800 0.95965200 2.09805200
H -0.42792600 0.95862700 -1.55864400

Imaginary Frequencies: 1

Electronic energy (B2PLYP(D3)/def2-SVP): -1184.021741 hartree

Electronic energy (DLPNO-CCSD(T)/def2-TZVPP): -1185.321329 hartree

Corrected Gibbs Free Energy (B2PLYP(D3)/def2-SVP): -1183.716795 hartree

3a$_{RRR}$ (IEFPCM(actonitrile)-DLPNO-CCSD(T)/def2-TZVPP//B2PLYP(D3)/def2-SVP)

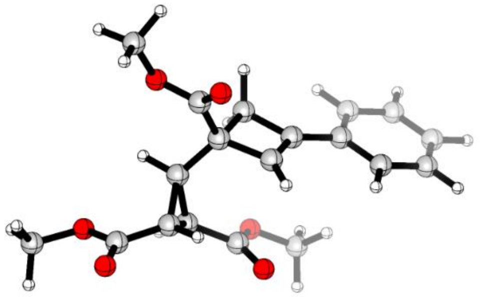

C -2.15919400 -0.89515800 0.61585100
C -1.50106600 0.00593200 -0.40685100
H -1.76599000 -0.86040000 1.63274800
C -1.22089900 -1.47132800 -0.41751100
C 0.07154600 -2.07211000 0.04653300
O 0.34374000 -2.38015400 1.18648400
O 0.89363500 -2.26312200 -0.99409500
C -3.61870300 -1.16625600 0.55866300
O -4.33217100 -1.28672200 1.53209900
O -4.06626500 -1.25710700 -0.70398200
C -5.46645200 -1.50515500 -0.86550100
H -5.64233000 -1.54812300 -1.94410200
H -5.74559100 -2.45633500 -0.39501300
H -6.05492800 -0.69598000 -0.41485400
C 2.15698200 -2.87355100 -0.69873800
H 2.68846300 -2.93854300 -1.65244400
H 2.72243200 -2.25714800 0.00988800
H 2.00820000 -3.87450500 -0.27429000
C -0.48805600 1.06038700 -0.00712200
C 1.59904400 0.79216700 0.06130800
C 0.67842300 1.23362700 -1.06129500
C 0.60344800 0.67925800 0.97289400
C -1.19595500 2.36105600 0.32659800
C 3.03717500 0.55149600 0.07089800
H 0.60275000 0.55048000 -1.91995600
H 0.82313900 2.26340800 -1.42084400
H -1.66995700 -2.00725400 -1.25581200
H 0.56577300 0.39902400 2.02556400
O -1.03430900 2.99855800 1.34431200
O -2.02299400 2.73589400 -0.65890600
C 3.69269100 0.11290200 1.23922000
C 3.78801600 0.72328800 -1.10601300
C -2.73946700 3.95972700 -0.45680300
C 5.06231600 -0.14905800 1.22533700
H 3.11928800 -0.02898300 2.15727100
C 5.16076800 0.45990200 -1.11781900
H 3.28978800 1.06060700 -2.01693300
H -3.35915200 4.09696700 -1.34747000
H -3.36828100 3.89288900 0.43991600
H -2.04159200 4.79899100 -0.34380700
C 5.80106200 0.02244900 0.04610300
H 5.55879700 -0.49025000 2.13545000
H 5.73150400 0.59511300 -2.03819200
H 6.87245500 -0.18498600 0.03715000
H -2.15792500 0.32879000 -1.21608600

Imaginary Frequencies: 0

Electronic energy (B2PLYP(D3)/def2-SVP): -1184.167785 hartree

Electronic energy (DLPNO-CCSD(T)/def2-TZVPP): -1185.459822 hartree

Corrected Gibbs Free Energy (B2PLYP(D3)/def2-SVP): -1183.857225 hartree

TS$_{SRR}$ (IEFPCM(actonitrile)-DLPNO-CCSD(T)/def2-TZVPP//B2PLYP(D3)/def2-SVP)

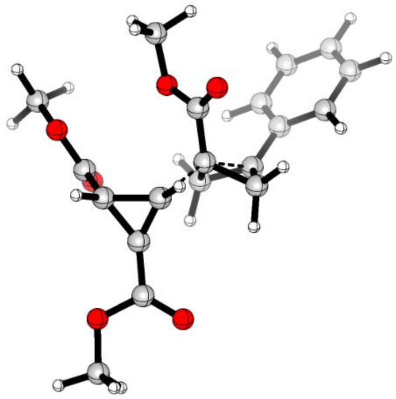

C -2.61247000 -0.53080200 0.33614600
C -1.59566000 -0.22404700 1.17577500
C -2.43791100 0.91675000 0.60709800
C -1.85642800 1.83986400 -0.42281700
O -1.57171300 1.55003800 -1.56577900
O -1.69025400 3.07253500 0.08480000
C -3.57602700 -1.56239400 0.06108600
O -3.37949300 -2.76240700 0.19081100
O -4.74697900 -1.05532300 -0.38683500
C -5.76127700 -2.01159400 -0.69120000
H -6.62537000 -1.43707200 -1.03944200
H -5.42563000 -2.70226700 -1.47645800
H -6.03144700 -2.59614800 0.19848700
C -1.13837000 4.04477300 -0.80676400
H -1.03986100 4.96765600 -0.22751600
H -0.15776000 3.71536500 -1.17213300
H -1.80300300 4.20352100 -1.66590700
C 0.21006800 -0.44112200 0.51497600
C 1.31271000 -1.44949800 -0.38435500
C 0.25703500 -0.62327600 -0.99499100
C 0.56377000 -1.90142800 0.81076900
C 0.98237700 0.62241900 1.20789600
C 2.74943600 -1.26579900 -0.49534800
H -3.10022800 1.44613200 1.30719600
H 1.11705800 -2.11662300 1.72876100
O 1.34156600 0.54475300 2.36502500
O 1.18310400 1.68879800 0.42346800
C 3.62538700 -1.91159100 0.40409200
C 3.29247600 -0.43869100 -1.50326500
C 1.86030300 2.79722900 1.02947500
C 5.00318600 -1.72076000 0.30616100
H 3.22419200 -2.57206000 1.17301000
C 4.67064900 -0.25293400 -1.59488800
H 2.63163200 0.05396600 -2.21625600
H 1.98588700 3.54147000 0.23848000
H 1.25578300 3.20831800 1.84767600
H 2.83636500 2.48333700 1.41874400
C 5.52994800 -0.88913700 -0.68961900
H 5.67038700 -2.22554100 1.00641200
H 5.07902300 0.38877600 -2.37706600
H 6.60858200 -0.74259200 -0.76510200
H -1.35918800 -0.44467200 2.21829800
H -0.29584500 -2.56330400 0.63910900
H -0.67027900 -1.12439800 -1.32493800
H 0.53463900 0.22120900 -1.62463300

Imaginary Frequencies: 1

Electronic energy (B2PLYP(D3)/def2-SVP): -1184.034163 hartree

Electronic energy (DLPNO-CCSD(T)/def2-TZVPP): -1185.333737 hartree

Corrected Gibbs Free Energy (B2PLYP(D3)/def2-SVP): -1183.727881 hartree

3a$_{SRR}$ (IEFPCM(actonitrile)-DLPNO-CCSD(T)/def2-TZVPP//B2PLYP(D3)/def2-SVP)

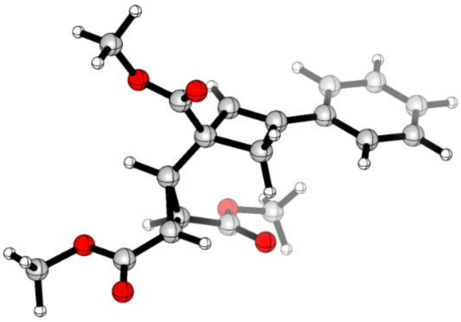

C -2.10414200 -0.95199400 0.70733700
C -1.55844200 0.05038800 -0.28235400
H -1.60360900 -1.02338900 1.67296700
C -1.29663600 -1.42158600 -0.48241500
C 0.04759600 -2.03839900 -0.26386900
O 0.57303400 -2.25171100 0.80902900
O 0.61421400 -2.33900500 -1.43814900
C -3.56382700 -1.22605800 0.78332000
O -4.16227600 -1.46177000 1.81151200
O -4.15101200 -1.17688900 -0.42251000
C -5.56161200 -1.41771900 -0.45343700
H -5.85779700 -1.33789000 -1.50318100
H -5.78922800 -2.41933700 -0.06757400
H -6.09213400 -0.67111500 0.15081800
C 1.93473400 -2.89335700 -1.37624700
H 2.21916400 -3.10631000 -2.41067100
H 2.62865900 -2.16913500 -0.93097300
H 1.93932600 -3.81447800 -0.78038200
C -0.49698400 1.08841000 0.06026000
C 1.59133500 0.80063900 0.11342100
C 0.71220300 1.08761300 -0.87653100
C 0.53303300 0.80018800 1.20531900
C -1.15070200 2.45834900 0.17526900
C 3.02010100 0.51633100 0.17605800
H -1.84631000 -1.86250100 -1.31489900
H 0.60335800 1.62069200 1.93106400
O -1.10716800 3.18839200 1.14095900
O -1.77178700 2.78456600 -0.96767800
C 3.60142700 0.11741400 1.39340400
C 3.83362100 0.59903100 -0.97213600
C -2.41121000 4.06578600 -1.00139800
C 4.96246200 -0.19314300 1.46278400
H 2.97898100 0.04537000 2.28710300
C 5.19119500 0.28856900 -0.90078000
H 3.39377200 0.90705800 -1.92273200
H -2.86228700 4.15132300 -1.99403600
H -3.18284500 4.13117200 -0.22402300
H -1.67606400 4.86537900 -0.84549100
C 5.76039900 -0.10964100 0.31709800
H 5.40068500 -0.50319500 2.41304300
H 5.81112800 0.35575600 -1.79657800
H 6.82286300 -0.35333000 0.37027800
H -2.29979600 0.43088800 -0.98623500
H 0.40295100 -0.15125500 1.73168600
H 0.78183700 1.23069000 -1.95609500

Imaginary Frequencies: 0

Electronic energy (B2PLYP(D3)/def2-SVP): -1184.164918 hartree

Electronic energy (DLPNO-CCSD(T)/def2-TZVPP): -1185.456308 hartree

Corrected Gibbs Free Energy (B2PLYP(D3)/def2-SVP): -1183.854183 hartree

3a$_{SSR}$ (IEFPCM(actonitrile)-DLPNO-CCSD(T)/def2-TZVPP//B2PLYP(D3)/def2-SVP)

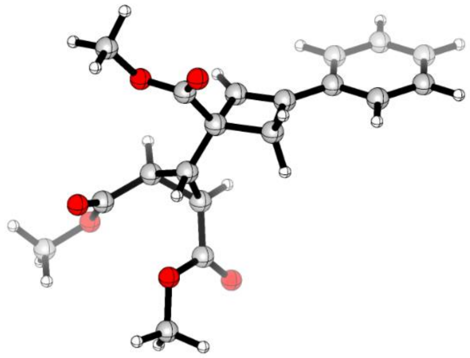

C 1.02599600 -1.10398200 0.23788900
C 0.93374700 0.24652600 -0.44795300
H 0.15178200 -1.44299800 0.79469900
C 1.73730300 0.12241000 0.79170500
C 3.23067000 0.25213800 0.72805000
O 3.83734300 0.99435200 -0.01082700
O 3.81464600 -0.54324600 1.63304100
C 1.78360400 -2.22572400 -0.37373200
O 1.47499600 -3.39399600 -0.27192600
O 2.86243000 -1.80080700 -1.05076700
C 3.68752200 -2.80943600 -1.64235500
H 4.50518200 -2.27603200 -2.13529300
H 4.08103200 -3.48287200 -0.87062300
H 3.11541500 -3.39360200 -2.37373100
C 5.24526100 -0.50791500 1.67106200
H 5.54377800 -1.19441200 2.46850300
H 5.65980800 -0.83733800 0.70953900
H 5.60251600 0.50697100 1.88649700
C -0.38281700 0.99717200 -0.47380300
C -2.30527100 0.23657700 -0.06698800
C -1.33613400 0.79384800 0.70040500
C -1.50358600 0.35063300 -1.35359300
C -0.12623000 2.46631500 -0.76622400
C -3.64055500 -0.29722900 0.17762000
H 1.31061800 0.50520100 1.72271400
H -1.91730500 1.01798800 -2.12083100
O -0.55047700 3.09041200 -1.71412600
O 0.63690900 3.00608500 0.19439500
C -4.39602200 -0.81350600 -0.89120200
C -4.19696600 -0.30952100 1.47269100
C 0.96168400 4.39273200 0.04646300
C -5.67666100 -1.32917300 -0.67269700
H -3.97471900 -0.80884800 -1.89826300
C -5.47479500 -0.82421500 1.68851000
H -3.62032600 0.08709900 2.31058900
H 1.58547800 4.64934100 0.90743200
H 1.51336300 4.56042500 -0.88721200
H 0.05001900 5.00360400 0.03951800
C -6.21919300 -1.33603100 0.61634800
H -6.25206400 -1.72651000 -1.51062200
H -5.89513900 -0.82840500 2.69573600
H -7.21895700 -1.73867200 0.78782900
H 1.52149200 0.32883300 -1.36651900
H -1.20784000 -0.60557600 -1.81061700
H -1.24626100 1.04367500 1.75916200

Imaginary Frequencies: 0

Electronic energy (B2PLYP(D3)/def2-SVP): -1184.167072 hartree

Electronic energy (DLPNO-CCSD(T)/def2-TZVPP): -1185.456657 hartree

Corrected Gibbs Free Energy (B2PLYP(D3)/def2-SVP): -1183.856081 hartree

BCB$_{CF3}$ (IEFPCM(actonitrile)-DLPNO-CCSD(T)/def2-TZVPP//B2PLYP(D3)/def2-SVP)

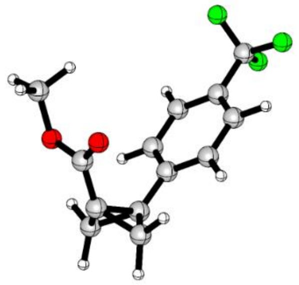

C 1.92433 -1.52625 0.39053
C 2.93164 -1.14657 1.43114
C 3.14661 -0.65026 0.03479
C 2.86473 -1.91741 -0.70754
H 3.61200 -1.92245 1.80101
H 2.63538 -0.40462 2.17513
H 3.53688 -2.76692 -0.54088
H 2.51089 -1.80530 -1.73510
C 2.93316 0.70000 -0.49773
O 2.80853 0.96090 -1.67841
O 2.83689 1.60947 0.48527
C 2.53736 2.94756 0.07463
H 2.50896 3.54419 0.99091
H 3.31293 3.32594 -0.60310
H 1.56609 2.98599 -0.43503
C 0.52141 -1.10805 0.28081
C -0.20589 -1.34094 -0.90014
C -0.11768 -0.44427 1.34726
C -1.52424 -0.90093 -1.02565
H 0.25686 -1.87880 -1.72736
C -1.43226 -0.00338 1.22373
H 0.41491 -0.27727 2.28315
C -2.13889 -0.22748 0.03370
H -2.07398 -1.08710 -1.94765
H -1.91114 0.51145 2.05801
C -3.55620 0.25523 -0.06848
F -4.11352 -0.02796 -1.25508
F -3.63998 1.58941 0.10073
F -4.33956 -0.29609 0.87921

Imaginary Frequencies: 0

Electronic energy (B2PLYP(D3)/def2-SVP): -949.512745 hartree

Electronic energy (DLPNO-CCSD(T)/def2-TZVPP): -950.521177 hartree

Corrected Gibbs Free Energy (B2PLYP(D3)/def2-SVP): -949.334398 hartree

TS$_{CF3}$ (IEFPCM(actonitrile)-DLPNO-CCSD(T)/def2-TZVPP//B2PLYP(D3)/def2-SVP)

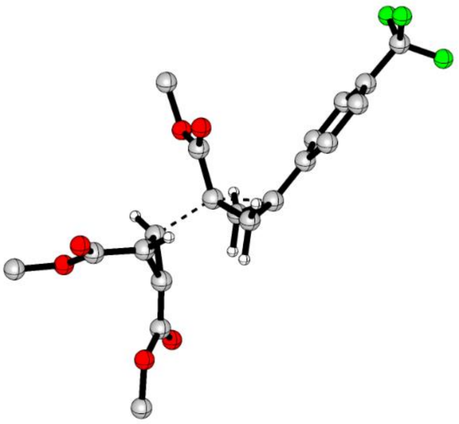

C 3.15381 0.48800 -0.82138
C 2.47548 0.25589 0.52331
H 2.52268 0.50144 -1.72051
C 3.24537 -0.73224 0.02286
C 4.10499 -1.83455 0.37246
O 3.95054 -2.57279 1.33253
O 5.11981 -1.96786 -0.50789
C 4.31613 1.41619 -0.94651
O 4.58124 2.05153 -1.94613
O 5.04772 1.46610 0.17879

C 6.19115 2.32401 0.15033
H 6.65637 2.24110 1.13695
H 6.89382 2.00392 -0.62974
H 5.89197 3.36215 -0.04355
C 6.03957 -3.02731 -0.24278
H 6.78270 -2.99082 -1.04526
H 6.52805 -2.88774 0.73076
H 5.52751 -3.99878 -0.24482
C 0.57442 -0.00502 0.28075
C -0.69240 -1.18302 0.07220
C 0.15294 -1.07874 1.28470
C 0.34408 -0.88612 -0.93262
C -0.03179 1.35536 0.29116
C -2.11744 -0.91490 -0.02379
H 0.94076 -1.83629 1.40499
H -0.30682 -0.76223 2.22380
H 1.18446 -1.60336 -1.02245
H 0.04847 -0.42111 -1.87568
O -0.30675 1.98734 -0.70710
O -0.19197 1.80115 1.54135
C -2.71566 -0.62885 -1.26960
C -2.92711 -0.93312 1.13236
C -0.75115 3.11544 1.67671
C -4.07829 -0.35817 -1.35439
H -2.11272 -0.62236 -2.17691
C -4.28902 -0.65941 1.04698
H -2.48918 -1.16994 2.10168
H -0.80293 3.30984 2.75135
H -0.10953 3.85520 1.18243
H -1.75322 3.14957 1.23154
C -4.86646 -0.36945 -0.19622
H -4.52924 -0.13291 -2.32071
H -4.90389 -0.67002 1.94689
H 2.44994 0.84553 1.44032
C -6.34757 -0.13091 -0.29440
F -6.83857 0.43764 0.81867
F -7.02423 -1.28150 -0.47413
F -6.66056 0.66882 -1.32633

Imaginary Frequencies: 1

Electronic energy (B2PLYP(D3)/def2-SVP): -1520.495316 hartree

Electronic energy (DLPNO-CCSD(T)/def2-TZVPP): -1522.012344 hartree

Corrected Gibbs Free Energy (B2PLYP(D3)/def2-SVP): -1520.185314 hartree

Product$_{CF3}$ (IEFPCM(actonitrile)-DLPNO-CCSD(T)/def2-TZVPP//B2PLYP(D3)/def2-SVP)

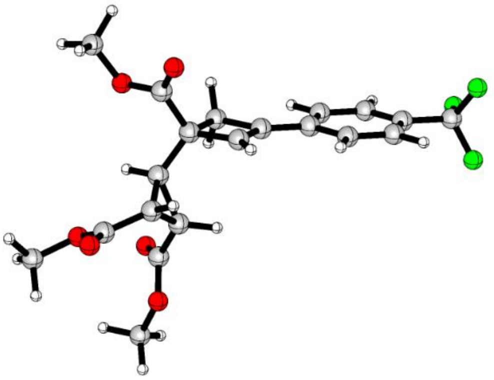

C -2.92928 -0.15388 1.10211
C -2.22864 0.35085 -0.14691
H -2.51340 0.12763 2.07025
C -2.06933 -1.07097 0.24498
C -2.68113 -2.14648 -0.60395
O -2.70201 -2.14804 -1.81451
O -3.16644 -3.13608 0.15466
C -4.40628 -0.31272 1.13942
O -5.08357 -0.18509 2.13678
O -4.91085 -0.62709 -0.06472
C -6.32144 -0.86092 -0.12740
H -6.53961 -1.09710 -1.17269
H -6.59805 -1.70262 0.51998
H -6.87364 0.03335 0.18674
C -3.77280 -4.22780 -0.54596
H -4.09228 -4.93887 0.22115
H -4.63736 -3.87592 -1.12342
H -3.05288 -4.69786 -1.22759
C -1.07686 1.32898 -0.04164
C 0.96173 0.80852 0.01890
C 0.03171 1.10280 -1.14398
C 0.01543 1.02137 0.96493
C -1.59235 2.75398 0.01477
C 2.37323 0.43967 0.03603

H -0.18641 0.25984 -1.81697
H 0.27974 1.99339 -1.74033
H -1.12927 -1.37388 0.71572
H 0.01263 0.99861 2.05579
O -1.29314 3.57064 0.85865
O -2.42513 3.00882 -1.00310
C 3.05240 0.20850 1.25040
C 3.07926 0.30779 -1.17166
C -2.97494 4.33033 -1.06044
C 4.39758 -0.14285 1.25257
H 2.51803 0.30589 2.19662
C 4.43059 -0.04529 -1.17414
H 2.56707 0.48310 -2.11891
H -3.62458 4.34858 -1.94003
H -3.55369 4.54492 -0.15325
H -2.17514 5.07492 -1.16058
C 5.08944 -0.27035 0.03698
H 4.91346 -0.31900 2.19765
H 4.96852 -0.14475 -2.11622
H -2.88748 0.52475 -0.99941
C 6.54167 -0.65110 0.07132
F 7.08222 -0.73595 -1.15292
F 6.72750 -1.84292 0.67102
F 7.27180 0.24189 0.76695

Imaginary Frequencies: 0

Electronic energy (B2PLYP(D3)/def2-SVP: -1520.363074 hartree

Electronic energy (DLPNO-CCSD(T)/def2-TZVPP):): -1522.012344 hartree

Corrected Gibbs Free Energy (B2PLYP(D3)/def2-SVP): -1520.057711hartree

BCB$_{OMe}$ (IEFPCM(actonitrile)-DLPNO-CCSD(T)/def2-TZVPP//B2PLYP(D3)/def2-SVP)

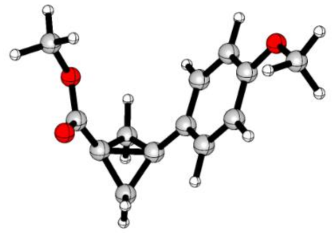

C 1.26419 -1.53744 0.35274
C 2.36751 -1.28107 1.32842
C 2.54845 -0.78333 -0.07455
C 2.08340 -2.00332 -0.80821
H 2.99156 -2.12484 1.64721
H 2.19295 -0.52725 2.09875
H 2.67773 -2.91875 -0.70271
H 1.67090 -1.83710 -1.80648
C 2.43251 0.58676 -0.57446
O 2.25223 0.88395 -1.74035
O 2.49161 1.48678 0.42336
C 2.28811 2.85156 0.04725
H 2.38507 3.43563 0.96716
H 3.04103 3.16802 -0.68575
H 1.28863 2.98814 -0.38567
C -0.10330 -0.99984 0.34442
C -0.94915 -1.19849 -0.75774
C -0.59877 -0.24769 1.43017
C -2.23724 -0.65419 -0.80052
H -0.60671 -1.79810 -1.60205
C -1.87580 0.29946 1.39811
H 0.02053 -0.09279 2.31419
C -2.70856 0.10610 0.28050
H -2.85949 -0.83536 -1.67508
H -2.25935 0.88119 2.23735
O -3.93613 0.67558 0.34377
C -4.81549 0.50676 -0.75343
H -5.73500 1.04412 -0.49821
H -4.39306 0.93060 -1.67781
H -5.05183 -0.55560 -0.92138

Imaginary Frequencies: 0

Electronic energy (B2PLYP(D3)/def2-SVP: -727.410246 hartree

Electronic energy (DLPNO-CCSD(T)/def2-TZVPP):): -728.201962 hartree

Corrected Gibbs Free Energy (B2PLYP(D3)/def2-SVP): -727.203537 hartree

TS$_{OMe}$ (IEFPCM(actonitrile)-DLPNO-CCSD(T)/def2-TZVPP//B2PLYP(D3)/def2-SVP)

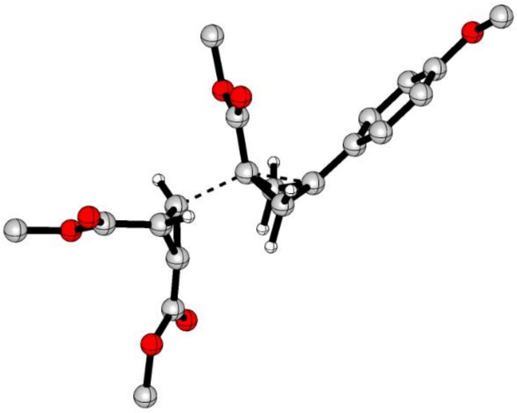

C 2.61471 0.50670 -0.80966
C 1.97089 0.29704 0.55289
H 1.96379 0.51568 -1.69485
C 2.71207 -0.70511 0.05016
C 3.53622 -1.84116 0.36796
O 3.38702 -2.57747 1.33121
O 4.51605 -2.01353 -0.54686
C 3.78123 1.42252 -0.97541
O 4.02409 2.04693 -1.98807
O 4.54656 1.47806 0.12773
C 5.69385 2.32759 0.05647
H 6.18765 2.25214 1.02979
H 6.37163 1.99506 -0.74035
H 5.39640 3.36586 -0.13977
C 5.39654 -3.11278 -0.31581
H 6.11379 -3.10540 -1.14242
H 5.92308 -2.99987 0.64128
H 4.84340 -4.06149 -0.30365
C 0.00353 0.02911 0.32554
C -1.24461 -1.16420 0.14598
C -0.38978 -1.03255 1.34980
C -0.21695 -0.87349 -0.87128
C -0.59335 1.38447 0.32201
C -2.67124 -0.93422 0.05408
H 0.40753 -1.77907 1.47727
H -0.84902 -0.70832 2.28668
H 0.62964 -1.58314 -0.95883
H -0.52723 -0.42618 -1.81845
O -0.87072 2.01114 -0.68072
O -0.75154 1.84796 1.56952
C -3.29147 -0.70150 -1.19004
C -3.48303 -0.93612 1.21389
C -1.30948 3.16275 1.68687
C -4.66117 -0.46491 -1.28620
H -2.69680 -0.71237 -2.10369
C -4.84541 -0.69893 1.12849
H -3.03813 -1.13495 2.18925
H -1.35602 3.37506 2.75860
H -0.67188 3.89632 1.17798
H -2.31435 3.19181 1.24709
C -5.45089 -0.45586 -0.12172
H -5.10277 -0.29132 -2.26546
H -5.47585 -0.70047 2.01820
H 1.92816 0.89993 1.45970
O -6.77874 -0.23596 -0.10360
C -7.45014 0.01111 -1.33023
H -8.50406 0.16330 -1.07664
H -7.05998 0.91360 -1.82417
H -7.36001 -0.84601 -2.01427

Imaginary Frequencies: 1

Electronic energy (B2PLYP(D3)/def2-SVP: -1298.266433 hartree

Electronic energy (DLPNO-CCSD(T)/def2-TZVPP):): -1299.697494 hartree

Corrected Gibbs Free Energy (B2PLYP(D3)/def2-SVP): -1297.930862 hartree

Product$_{OMe}$ (IEFPCM(actonitrile)-DLPNO-CCSD(T)/def2-TZVPP//B2PLYP(D3)/def2-SVP)

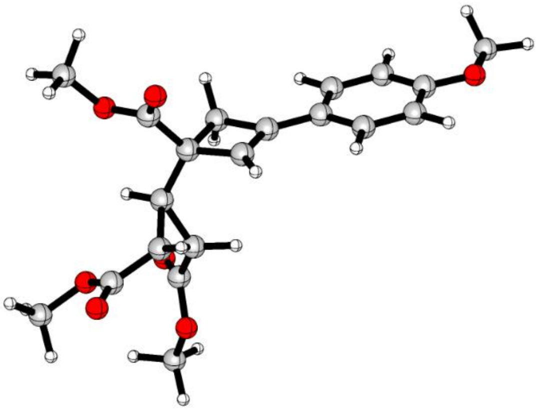

C -2.40288 -0.09425 1.08436
C -1.64518 0.36242 -0.15027
H -2.00592 0.18940 2.05978
C -1.55120 -1.05674 0.26980
C -2.17569 -2.12485 -0.57854
O -2.16393 -2.14687 -1.78912
O -2.71504 -3.08519 0.18184
C -3.88473 -0.19856 1.07868
O -4.58765 -0.02357 2.05101
O -4.36401 -0.52169 -0.13372
C -5.77977 -0.70345 -0.23567
H -5.97444 -0.95833 -1.28125
H -6.10909 -1.51681 0.42321
H -6.30660 0.21899 0.03810
C -3.33908 -4.16701 -0.51815
H -3.70193 -4.85515 0.25064
H -4.17630 -3.79611 -1.12338
H -2.61811 -4.67135 -1.17370
C -0.45890 1.29681 -0.02117
C 1.55437 0.69117 0.13389
C 0.68172 0.99572 -1.07109
C 0.57967 0.97112 1.03463
C -0.92109 2.74092 -0.02706
C 2.94333 0.26011 0.20567
H 0.45353 0.14663 -1.73342
H 0.99103 1.85951 -1.67864
H -0.63694 -1.38260 0.77480
H 0.53256 0.97813 2.12481
O -0.62978 3.57231 0.80531
O -1.69898 2.99789 -1.08863
C 3.57997 0.02417 1.44454
C 3.68807 0.06364 -0.96774
C -2.19417 4.33635 -1.20569
C 4.90294 -0.38908 1.49896
H 3.02428 0.16775 2.37329
C 5.02272 -0.35315 -0.92778
H 3.21886 0.23869 -1.93795
H -2.80392 4.35429 -2.11351
H -2.80319 4.59884 -0.33141
H -1.36306 5.04799 -1.29008
C 5.63932 -0.58254 0.31200
H 5.39999 -0.57305 2.45232
H 5.56518 -0.49403 -1.86080
H -2.27060 0.54492 -1.02585
O 6.92039 -0.98657 0.46885
C 7.71049 -1.20021 -0.68807
H 7.81798 -0.27690 -1.27798
H 8.69638 -1.51877 -0.33358
H 7.28284 -1.98790 -1.32739

Imaginary Frequencies: 0

Electronic energy (B2PLYP(D3)/def2-SVP: -1298.394144 hartree

Electronic energy (DLPNO-CCSD(T)/def2-TZVPP):): -1299.816662 hartree

Corrected Gibbs Free Energy (B2PLYP(D3)/def2-SVP): -1298.053924 hartree

12 References

1 (a) Wang, M.; Huang, Y.; Li, C.; Lu, P. Diastereoselective Synthesis of 1,1,3,3-Tetrasubstituted Cyclobutanes Enabled by Cycloaddition of Bicyclo[1.1.0]Butanes. *Org. Chem. Front*., **2022**, *9*, 2149. DOI: 10.1039/d2qo00167e; (b) Livingstone, K.; Siebold, K.; Meyer, S.; Martín-Heras, V.; Daniliuc, C. G.; Gilmour, R. Skeletal Ring Contractions via I(I)/I(III) Catalysis: Stereoselective Synthesis of *cis-α,α*-Difluorocyclopropanes. *ACS Catal.,* **2022**, *12*, 14507. DOI: 10.1021/acscatal.2c04511.

2 Dhake, K.; Woelk, K. J.; Becica, J.; Un, A.; Jenny, S. E.; Leitch, D. C. Beyond Bioisosteres: Divergent Synthesis of Azabicyclohexanes and Cyclobutenyl Amines from Bicyclobutanes. *Angew. Chem., Int. Ed.,* **2022**, *61*. DOI: e202204719.

3 Kleinmans, R.; Pinkert, T.; Dutta, S.; Paulisch, T. O.; Keum, H.; Daniliuc, C. G.; Glorius, F.; Intermolecular [2*π*+2*σ*]-Photocycloaddition Enabled by Triplet Energy Transfer. *Nature*, **2022**, *605*, 477. DOI: 10.1038/s41586-022-04636-x

4 McNamee, R. E.; Thompson, A. L.; Anderson, E. A. Synthesis and Applications of Polysubstituted Bicyclo[1.1.0]Butanes. *J. Am. Chem. Soc.* **2021**, *143* (50), 21246. DOI: 10.1021/jacs.1c11244; (b) McNamee, R. E.; Haugland, M. M.; Nugent, J.; Chan, R.; Christensen, K. E.; Anderson, E. A. Synthesis of 1,3-Disubstituted Bicyclo[1.1.0]Butanesviadirected Bridgehead Functionalization. *Chem. Sci.,* **2021**, *12* (21), 7480. DOI: 10.1039/d1sc01836a.

5 (a) Dasgupta, A.; Babaahmadi, R.; Pahar, S.; Stefkova, K.; Gierlichs, L.; Yates, B. F.; Ariafard, A.; Melen, R. L. Tris(Pentafluorophenyl)Borane-Catalyzed Carbenium Ion Generation and Autocatalytic Pyrazole Synthesis—A Computational and Experimental Study. *Angew. Chem. Int. Ed.,* **2021**, *60* (46), 24395. DOI: 10.1002/anie.202109744; (b) Dasgupta, A., Pahar, S., Babaahmadi, R., Gierlichs, L., Yates, B. F., Ariafard, A., and Melen, R. L. Borane Catalyzed Selective Diazo Cross-Coupling Towards Pyrazoles *Adv. Synth. Catal*., **2022**, *364*, 773, DOI: 0.1002/adsc.202101312.

6 Supurgibekov, M. B.; Prakash, G. K. S.; Nikolaev, V. A. Two-Stage Synthesis of 3-(Perfluoroalkyl) -Substituted Vinyldiazocarbonyl Compounds and Their Nonfluorinated Counterparts: A Comparative Study. *Synthesis,* **2013**, 45, 1215.

7 (a) Himeshima, Y.; Sonoda, T.; Kobayashi, H. Fluoride-induced 1,2-Elimination of *o*-Trimethylphenyl Triflate to Benzyne under Mild Conditions. *Chem. Lett.,* **1983**, 1211. DOI: 10.1246/cl.1983.1211; For a modified procedure, see: (b) Peña, D.; Cobas, A.; Pérez, D.; Guitián, E. An Efficient Procedure for the Synthesis of ortho-Trialkylsilylaryl Triflates: Easy Access to Precursors of Functionalized Arynes. *Synthesis,* **2002**, 1454. DOI: 10.1055/s-2002-33110

8 Palatinus, L.; Chapuis, G., *SUPERFLIP* - A Computer Program for the Solution of Crystal Structures by Charge Flipping in Arbitrary Dimensions**.** *J. Appl. Cryst*., **2007**, *40*, 786. DOI: 10.1107/S0021889807029238.

9 Parois, P.; Cooper, R. I.; Thompson, A. L.; Crystal Structures of Increasingly Large Molecules: Meeting the Challenges with Crystal Software**.** *Chem. Cent. J.,* **2015**, 9:30. DOI: 10.1186/s13065-015-0105-4.

10 R. I. Cooper, A. L. Thompson and D. J. Watkin. Crystals Enhancements: Dealing with Hydrogen Atoms in Refinement. *J. Appl. Cryst*., **2010**, *43*, 1100. DOI: 10.1107/S0021889810025598.

11 Dolomanov, O.V., Bourhis, L.J., Gildea, R. J, Howard, J.A.K. & Puschmann, H.; *OLEX2*: A Complete Structure Solution, Refinement and Analysis Program. *J. Appl. Cryst*., **2009,** *42*, 339. DOI: 10.1107/S0021889808042726.

12 *Gaussian 16, Rev. C.01*; Gaussian, Inc.: Wallingford CT, 2016. (accessed).

13 Neese, F.; Wennmohs, F.; Becker, U.; Riplinger, C. The ORCA Quantum Chemistry Program Package. *J. Chem. Phys.,* **2020**, *152* (22), 224108. DOI: 10.1063/5.0004608.

14 Tomasi, J.; Mennucci, B.; Cammi, R. Quantum Mechanical Continuum Solvation Models. *Chem. Rev.,* **2005**, *105* (8), 2999. DOI: 10.1021/cr9904009.

15 Grimme, S.; Semiempirical Hybrid Density Functional with Perturbative Second-order Correlation. *J. Chem. Phys.,* **2006**, *124* (3), 034108. DOI: 10.1063/1.2148954.

16 Weigend, F.; Ahlrichs, R. Balanced Basis Sets of Split Valence, Triple zeta Valence and Quadruple zeta Valence Quality for H to Rn: Design and Assessment of Accuracy. *Phys. Chem. Chem. Phys*., **2005**, *7* (18), 3297. DOI: 10.1039/B508541A.

17 Liakos, D. G.; Guo, Y.; Neese, F. Comprehensive Benchmark Results for the Domain Based Local Pair Natural Orbital Coupled Cluster Method (DLPNO-CCSD(T)) for Closed- and Open-Shell Systems. *J. Phys. Chem. A.,* **2020**, *124* (1), 90. DOI: 10.1021/acs.jpca.9b05734.

18 Barone, V.; Cossi, M. Quantum Calculation of Molecular Energies and Energy Gradients in Solution by a Conductor Solvent Model. *J. Phys. Chem. A,* **1998**, *102* (11), 1995. DOI: 10.1021/jp9716997.

19 Luchini, G.; Alegre-Requena, J.; Funes-Ardoiz, I.; Paton, R. GoodVibes: Automated Thermochemistry for Heterogeneous Computational Chemistry Data [version 1; peer review: 2 approved with reservations]. *F1000Research,* **2020**, *9* (291). DOI: 10.12688/f1000research.22758.1.

20 Chai, J.-D.; Head-Gordon, M. Long-range Corrected Hybrid Density Functionals with Damped Atom–atom Dispersion Corrections. *Phys. Chem. Chem. Phys.,* **2008**, *10* (44), 6615. DOI: 10.1039/B810189B.

21 Perdew, J. P.; Burke, K.; Ernzerhof, M. Generalized Gradient Approximation Made Simple [Phys. Rev. Lett. 77, 3865 (1996)]. *Phys. Rev. Lett.,* **1997**, *78* (7), 1396. DOI: 10.1103/PhysRevLett.78.1396.

22 Tao, J.; Perdew, J. P.; Staroverov, V. N.; Scuseria, G. E. Climbing the Density Functional Ladder: Nonempirical Meta--Generalized Gradient Approximation Designed for Molecules and Solids. *Phys. Rev. Lett.,* **2003**, *91* (14), 146401. DOI: 10.1103/PhysRevLett.91.146401.

23 Zhao, Y.; Truhlar, D. G. The M06 Suite of Density Functionals for Main Group Thermochemistry, Thermochemical Kinetics, Noncovalent Interactions, Excited States, and Transition Elements: Two New Functionals and Systematic Testing of Four M06-Class Functionals and 12 other Functionals. *Theor. Chem. Account,* **2008**, *120* (1), 215. DOI: 10.1007/s00214-007-0310-x.

24 Grimme, S.; Ehrlich, S.; Goerigk, L. Effect of the Damping Function in Dispersion Corrected Density Functional theory. *J. Comput. Chem.,* **2011**, *32* (7), 1456. DOI: 10.1002/jcc.21759.

25 Goerigk, L.; Grimme, S. Efficient and Accurate Double-Hybrid-Meta-GGA Density Functionals—Evaluation with the Extended GMTKN30 Database for General Main Group Thermochemistry, Kinetics, and Noncovalent Interactions. *J. Chem. Theory Comput.,* **2011**, *7* (2), 291. DOI: 10.1021/ct100466k.
